# Chromosome‐Level Genome and Organ‐Specific Transcriptome of *Alnus glutinosa* Uncover Lineage‐Specific Innovations in Root Nodule Symbiosis

**DOI:** 10.1111/pce.70440

**Published:** 2026-02-12

**Authors:** Zijian Liu, Xiaoxiao Zhao, Xiuli li, Yong Feng, Linnan Wu, Zhen Wu, Yao Zhong, Qingcheng Qiu, Bo Song, Hang Zhao, Hongbing Liu, Shifeng Cheng

**Affiliations:** ^1^ Shenzhen Branch, Guangdong Laboratory of Lingnan Modern Agriculture, Genome Analysis Laboratory of the Ministry of Agriculture and Rural Affairs, Agricultural Genomics Institute at Shenzhen Chinese Academy of Agricultural Sciences Shenzhen China; ^2^ Microbial Processes and Interactions (MiPI), TERRA Teaching and Research Centre, Gembloux Agro‐Bio Tech University of Liège Gembloux Belgium; ^3^ School of Biological Sciences The University of Hong Kong Hong Kong, SAR China; ^4^ State Key Laboratory of Agrobiotechnology The Chinese University of Hong Kong Hong Kong China

**Keywords:** genetic variation, genome, nutrients/nitrogen, symbiosis

## Abstract

*Alnus glutinosa* is one of only three lineages within the order Fagales capable of establishing root nodule symbiosis (RNS). Although a fragmented genome assembly of *A. glutinosa* was previously available, its limited quality, combined with the lack of comprehensive transcriptomic resources, has constrained in‐depth comparative and functional genomic analyses. In this study, we present a 505 Mb chromosome‐level genome assembly of *A. glutinosa*, anchored to 14 pseudochromosomes, representing the most complete and high‐quality genomic resource for this species to date. Whole‐genome alignment and synonymous substitution rate (Ks) analysis confirm *Alnus* and *Betula* as sister genera with shared genomic architectures and evolutionary histories. Functional enrichment analyses of nodule‐enhanced genes reveal significant associations with photosynthesis and sugar metabolism, while expanded gene families are enriched in terpenoid biosynthesis and malate transport pathways, likely critical to RNS in *A. glutinosa*. Phylogenetic analysis indicated that *Alnus* has retained non‐symbiotic class 1 haemoglobin (nsHB1), but lost nsHB2 haemoglobin, suggesting a lineage‐specific adaptation in symbiotic oxygen regulation. Further comparative analysis of nsHB1 protein sequences across nodulating taxa highlights evolutionary patterns within the *Alnus* lineage. Through a targeted phylogenetic survey of known RNS‐related genes, we identified PAV in *RPG* and copy number variation in *AGO5*, both of which may underlie *Alnus*‐specific RNS adaptations. Weighted gene co‐expression network analysis identified a nodule‐specific module comprising 231 genes significantly enriched in sugar‐related metabolic pathways. Notably, the *bZIP* ortholog shows conserved nodule‐specific expression across species from Cucurbitales, Rosales and Fabales, suggesting deep evolutionary conservation within the nitrogen‐fixing clade. Together, these findings provide a high‐resolution view of *Alnus*‐specific RNS adaptations and uncover conserved regulatory modules potentially critical for RNS. These works establish a foundational genomic framework for future efforts aimed at engineering RNS capacity into non‐nodulating crops.

## Summary statement

We provide a chromosome‐level genome and organ transcriptomes for the actinorhizal tree *Alnus glutinosa*, uncovering key lineage‐specific and conserved regulators associated with root nodule symbiosis.

## 1 Introduction

Root nodule symbiosis (RNS) is a specialised form of mutualism in which certain plants accommodate nitrogen‐fixing bacteria within root‐derived organs known as nodules. In legumes, the symbionts are typically *Rhizobium* species, while in non‐legume actinorhizal plants, such as *Alnus* species, the microsymbionts are *Frankia* spp (Huisman and Geurts 2020). Within nodules, differentiated bacteroids reduce atmospheric nitrogen (N_2_) to ammonia via nitrogenase, while the plant host supplies carbon sources and maintains the low‐oxygen environment required for nitrogen fixation (Poole et al. 2018). Phylogenetic studies have shown that all extant nodulators are restricted to a monophyletic nitrogen‐fixing clade comprising four orders: Fabales, Rosales, Cucurbitales and Fagales (Soltis et al. 1995). Within this clade, RNS is most prevalent in Fabaceae, while non‐legume nodulators are sparsely distributed, with nodulators and non‐nodulators often coexisting within the same order (Doyle 2016).

**Figure 1:**
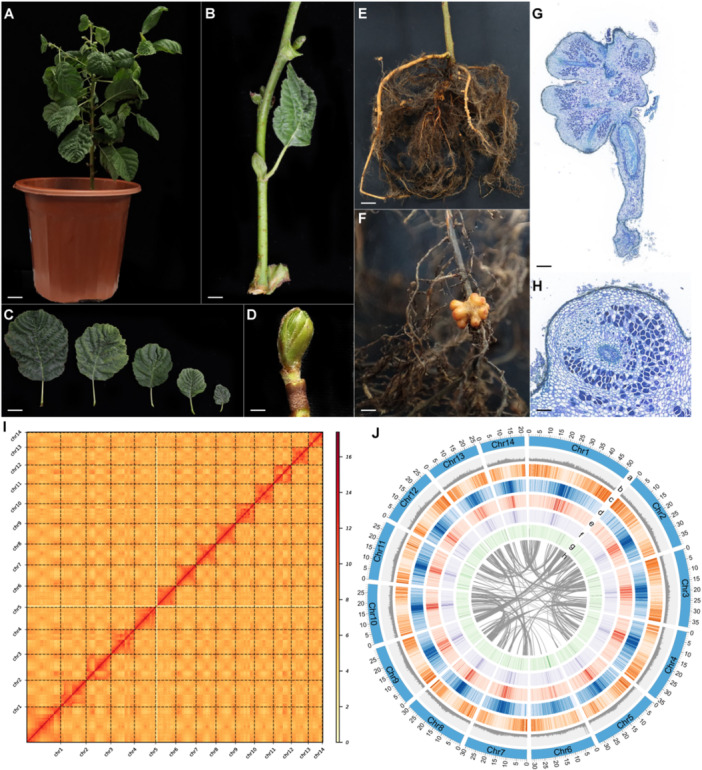
Overview of the phenotype, chromosome structure and genomic landscape of *Alnus glutinosa*. (A–H) Phenotype of *A. glutinosa*. (A) Whole plant. (B) Stem and stipule. (C) Leaves are in different stages. (D) Budding. (E) Root. (F) Nodule. (G) Nodule section stained with Trypan blue. (H) Magnified views of the nodule in (G). For A, scale bar = 3.0 cm. For B, scale bar = 0.5 cm. For C, scale bar = 2 cm. For D, scale bar = 0.2 cm. For E, scale bar = 2 cm. For F, scale bar = 0.2 cm. For G, scale bar = 0.5 mm. For H, scale bar = 0.2 mm. (I) Chromosome structure of *A. glutinosa*. Hi‐C contact map of the 14 assembled pseudochromosomes (*n* = 14). (J) Genomic landscape of *A. glutinosa*. Genomic landscape of *A. glutinosa*. Different tracks in the circos represent a, chromosome ideogram (unit, Mb); b, GC content; c, gene density along the chromosomes; d, repeat ratio of each bin; e, percentage of LTR_Copia among TEs; f, percentage of LTR_Gypsy among TEs; g, percentage of Helitron among TEs; h, intra‐genome collinear blocks connected by curved lines. All density or ratio information was determined in non‐overlapping 100 kb windows.

*Alnus glutinosa*, a deciduous actinorhizal tree of the order Fagales, is one of only a few non‐legume species capable of establishing RNS. The tree exhibits smooth, greenish stems and terminal buds that are ovoid, resinous and reddish‐brown in coloration (Figure 1A,B,D). Its leaves display a rounded to broadly ovate morphology with a slightly notched apex, presenting a glossy dark green surface and sticky texture when young (Figure 1C). The root system develops irregularly lobed, reddish‐brown symbiotic nodules when infected by its microsymbiont *Frankia alni* (Figure 1E,F) (Benson and Silvester 1993). Internally, these nodules show a characteristic spongy parenchyma resulting from bacterial colonisation (Figure 1G,H), facilitating nitrogen fixation under nutrient‐poor conditions such as glacial moraines, volcanic deposits and degraded lands (Huss‐Danell 1997; Põlme et al. 2014). Understanding RNS in this lineage not only provides evolutionary insights into actinorhizal symbiosis but also has potential agronomic value for developing nitrogen‐fixing crops (Rogers and Oldroyd 2014).

Although significant advances have been made in deciphering the molecular basis of legume RNS through studies on *Medicago truncatula*, *Lotus japonicus* and other models, non‐legume RNS remains poorly understood due to limitations such as the lack of stable transformation systems (Oldroyd 2013; Roy et al. 2020). Nevertheless, comparative studies suggest deep evolutionary homology between legume and non‐legume RNS. A key example is the conserved use of the Common Symbiosis Signalling Pathway, originally co‐opted from arbuscular mycorrhizal signalling (Maillet et al. 2011; Zhu et al. 2006). However, differences also exist. For instance, legumes employ Nod factor receptors for rhizobial recognition (Radutoiu et al. 2003), whereas actinorhizal plants like *Alnus* appear to utilise alternative recognition systems (Bhattacharyya et al. 2024). Previous studies on *A. glutinosa* have provided some initial insights into its RNS mechanisms. Early comparative transcriptomic studies using root and nodule microarrays identified strong up‐regulation of genes involved in carbon‐nitrogen exchange, pathogen defence and stress responses in nodules (Hocher et al. 2011). Based on EST transcriptome data and qPCR analysis, further research identified *AgZF1*, a transcription factor (TF) specific to non‐legume RNS in *A. glutinosa* (Diédhiou et al. 2014).

The draft genome of *A. glutinosa* was included in a broader phylogenomic study on nodulators, where conservation of the *NIN* TF supported a single origin model for RNS within the nitrogen‐fixing clade (Griesmann et al. 2018). However, the previously published genome of *A. glutinosa* remained at the scaffold level, and no organ‐specific transcriptomic resources were available, thereby limiting the resolution of genomic and functional analyses. More recently, chromosome‐level assemblies have become available for the nodulators *Alnus rubra* and for two closely related non‐nodulators, *Betula pendula* and *Betula platyphylla* (Hixson 2023; Salojärvi et al. 2017; Chen et al. 2021). Despite these advances, research into the genetic basis of RNS in *Alnus* remains limited, underscoring a critical gap in our understanding of non‐legume RNS.

In this study, we report a high‐quality chromosome‐level genome assembly of *A. glutinosa*, generated using PacBio HiFi long‐read and Hi‐C sequencing technologies. The final assembly achieves an N50 of ~31 Mb and exhibits 98.7% BUSCO completeness. We also mapped the PacBio HiFi raw reads to the final assembly, achieving a 99.99% mapping rate. To our knowledge, among the most complete chromosome‐level assemblies reported so far. We further generated organ‐specific transcriptomes from mature nodules, roots, leaves, buds and stems, enabling the identification of candidate RNS‐related genes using weighted gene co‐expression network analysis (WGCNA). Leveraging these genomic and transcriptomic resources, we investigated the evolution of gene families and symbiosis‐related pathways through orthogroup reconstruction, divergence time estimation and analysis of presence–absence and copy number variations (CNVs). Collectively, our study provides new insights into the genomic basis of actinorhizal RNS, elucidates lineage‐specific adaptations in *A. glutinosa*, and contributes to a broader understanding of RNS evolution across the nitrogen‐fixing clade.

## 2 Results

### 2.1 Genome Sequencing, Assembly and Annotation of *Alnus glutinosa*

The seeds of *A. glutinosa* were sourced from France and cultivated under controlled greenhouse conditions in Shenzhen, China. Once the plants reached an appropriate growth stage, their roots were inoculated with *Frankia alni* to induce the formation of nodules. As a characteristic feature of actinorhizal plants, *A. glutinosa* exhibited the typical nodule morphology. Histological analysis through sectioning and staining of the nodules revealed distinct infected cells, confirming the integrity of the nodules (Tu et al. 2024). The genome of *A. glutinosa* was sequenced using PacBio HiFi and Hi‐C technology, releasing 38.01 Gb and 55.72 Gb of data, respectively (Table S1). Based on prior genome survey analyses (Griesmann et al. 2018), the estimated genome size of *A. glutinosa* is approximately 461 Mb.

The PacBio HiFi sequencing data were assembled using the hifiasm assembler, which yielded two high‐quality haplotype‐resolved contig assemblies of *A. glutinosa*. From these assemblies, the higher‐quality haplotype‐resolved contig set was selected for further analysis (Table S2). Hi‐C reads were aligned to the *A. glutinosa* haplotype‐resolved contig assembly using Juicer, capturing inter‐ and intra‐chromosomal interactions to generate a contact frequency matrix. This matrix was processed with 3D‐DNA (v180419) to cluster and orient contigs into preliminary chromosome‐scale scaffolds based on Hi‐C interaction patterns. Due to potential misjoins and misorientations in the initial scaffolding, we performed manual curation by visualising the assembly in Juicebox and adjusting discordant regions (Figure 1I). Finally, a second iteration of 3D‐DNA refinement was applied to the curated scaffolds, yielding a chromosome‐level assembly with improved continuity. We additionally quantified Hi‐C contact probability as a function of genomic distance, and the smooth monotonic distance‐decay profile provides a quantitative quality metric supporting correct chromosome‐scale scaffolding (Figure S19). The final scaffolded genome of *A. glutinosa* spans approximately 505 Mb, with 453 Mb of sequences anchored to 14 pseudochromosomes (Table S3). The assembly exhibits a contig N50 length of ~30.8 Mb, and the completeness assessed using Benchmarking Universal Single‐Copy Orthologs (BUSCO) reached 98.7% when evaluated against both the *embryophyta_odb10* and *eudicots_odb10* databases. (Tables S2, S4). These results surpass those of the previously best‐assembled *A. glutinosa* genome (Christenhusz et al. 2024), establishing this assembly as the most complete genome of *A. glutinosa* to date.

Before gene prediction, repetitive sequences within the chromosome‐level assembly were annotated using a de novo approach through the EDTA pipeline, which identified 45.63% of the genome as repetitive sequences. Among these, Copia, Gypsy and Helitron elements comprised 9.73%, 7.78% and 3.64%, respectively (Figure 1J) (Table S5). For gene prediction, the MAKER pipeline annotated 28,979 protein‐coding genes, with a completeness of 91.2% according to the BUSCO evaluation against the eudicots_odb10 database (Table S6), confirming the high quality of the gene predictions. Functional annotation of the predicted gene set was performed using the eggNOG‐mapper, which assigned functional categories to 24 789 genes (Table S7). The predicted gene count for *A. glutinosa* is approximately half that of the closely related *A. rubra*, which has undergone extensive gene duplication (Hixson 2023). This suggests that *A. glutinosa* possesses a streamlined genome, making it an ideal candidate for comparative genomics and future molecular studies.

### 2.2 The Expansion of Gene Families Reveals the Evolutionary Trajectory of RNS in *A. glutinosa*

**Figure 2:**
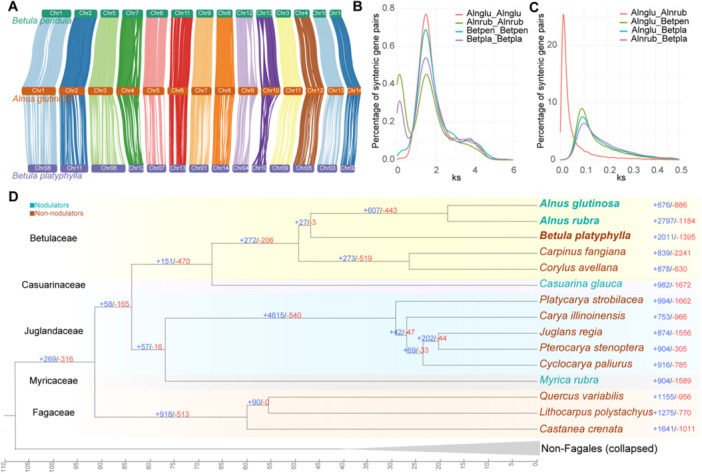
Phylogenetic inference of *Alnus glutinosa* and other representative species in Fagales. (A) Whole‐genome synteny comparison between *A. glutinosa* and two chromosome‐scale assembled relatives, *Betula pendula* and *Betula platyphylla*. (B) Synonymous substitution (Ks) of paralogous gene pairs in intragenomic collinear regions of selected plant genomes. (C) Ks distributions calculated from orthologous gene pairs in intergenomic collinear regions between different species. Alnglu, *A. glutinosa*; Betpla, *B. platyphylla*; Alnrub, *A. rubra*; Betpen, *B. pendula*. (D) Gene family expansion and contraction. The phylogenetic tree of 20 plant species shows the number of expanded and contracted gene families, represented in blue and red, respectively. Species names in cyan indicate nodulators, while those in brown represent non‐nodulators. The background colour corresponds to the family each species belongs to. The horizontal line at the bottom is the timeline (million years ago, Mya). For clarity, non‐Fagales taxa were collapsed into a single clade, and *Alnus* and *Betula* were highlighted in bold. [Color figure can be viewed at wileyonlinelibrary.com]

Despite their close phylogenetic relationship in the Betulaceae family, *Alnus* and *Betula* exhibit striking differences in RNS: *Alnus* species universally retain the RNS trait, whereas *Betula* species appear to have either lost this trait or never acquired it (Tedersoo et al. 2018). To assess genomic conservation within Betulaceae, we conducted whole‐genome alignments using MUMmer4, with *A. glutinosa* serving as the reference, and *B. pendula* and *B. platyphylla* as comparators. The comparative analysis revealed strong synteny conservation across all three species, with *A. glutinosa* exhibiting slightly higher collinearity with *B. pendula* (Figure 2A; Figure S1A,B) (Table S8). To explore the possibility of lineage‐specific whole‐genome duplication (WGD) events in *Alnus* and *Betula*, we analysed the distribution of synonymous substitution rates (Ks) among paralogous gene pairs within collinear blocks (Maere et al. 2005). The Ks distribution for *A. glutinosa* showed a prominent peak at Ks ≈ 1.5 (Table S9), which corresponds to the ancient γ hexaploidization event shared by core eudicots around 130 million years ago (Mya) (Jiao et al. 2012), suggesting the absence of recent lineage‐specific WGD events. In contrast, minor secondary peaks at lower Ks values were detected in *A. rubra* and *B. platyphylla* (Figure 2B), indicative of recent small‐scale gene duplications or expansions in these species. Additionally, the Ks distribution of orthologous gene pairs between *Alnus* and *Betula* revealed a unimodal peak centred at ~0.1 (Table S9; Figure 2C). Based on the estimated divergence time of ~46.91 Mya between *Alnus* and *Betula* (Figure S2), we calculated a synonymous substitution rate of ~1.06 × 10^−9^ substitutions per site per year, consistent with reported rates in the Juglandaceae (Ding et al. 2023), indicating conserved molecular evolutionary dynamics among closely related lineages.

To enable broader comparative genomic analyses, we selected *A. glutinosa*, *A. rubra*, 13 additional Fagales species (each represented by the species with the highest‐quality genome assembly within its genus), four extensively studied legumes capable of RNS (*Medicago truncatula*, *Lotus japonicus*, *Glycine max* and *Phaseolus vulgaris*), and the outgroup *Arabidopsis thaliana* for the reconstruction of orthologous groups (OGs) utilising the Orthofinder pipeline (Table S10). Given the relatively poor assembly and gene prediction quality of *B. pendula*, it was excluded from orthology inference to avoid potential biases (Gaudet and Dessimoz 2017). In total, 42 389 OGs were identified across the included Fagales and legume species (Table S11). Among these, 12 594 and 10 036 one‐to‐one orthologous gene pairs were found between *A. glutinosa* versus *B. platyphylla* and *A. rubra* versus *B. platyphylla*, respectively. Notably, *A. glutinosa* has more one‐to‐one orthologs with other species than *A. rubra*, suggesting a closer phylogenetic relationship. A robust phylogenetic tree reconstructed from single‐copy orthologs confirmed the sister‐group relationship between *Alnus* and *Betula* (Figure 2D). Based on the orthogroups, we performed gene family expansion and contraction analyses along each branch of the phylogeny. In *A. glutinosa*, 676 gene families were expanded, while 886 were contracted (Figure 2D). Of these, 35 expanded and 8 contracted gene families were statistically significant (*p* < 0.01) (Table S12). GO and KEGG enrichment analyses of the significantly expanded gene families revealed enrichment in terpenoid metabolic processes and malate transport (Table S12, S13) (Figure S3A,B). Previous studies have shown that genes involved in terpenoid biosynthesis can enhance RNS efficiency in soybean (Ali et al. 2021), suggesting that the expansion of these pathways in *A. glutinosa* may represent evolutionary adaptations to optimise RNS. To determine whether this enrichment is specific to *A. glutinosa* rather than a general feature of non‐nodulating Fagales, we included an additional non‐nodulating control, *Quercus variabilis*, and compared copy numbers of representative terpenoid‐related OGs. Several OGs show pronounced *Alnus*‐biased expansions relative to both *Betula* and *Quercus* (Figure S18), including Germacrene D synthase (OG 37ICK) and a UDP‐glycosyltransferase family (OG 37T4R).

To explore organ‐specific gene expression, RNA‐seq data from three biological replicates across five *A. glutinosa* organs were aligned to the reference genome, with mapping rates exceeding 90% for all samples (Table S15). Principal component analysis (PCA) and sample‐to‐sample distance heatmaps based on variance‐stabilised counts showed clustering primarily by organ, with tight replicate grouping and no obvious outliers (Figure S13), indicating limited technical variation. Differential gene expression analysis between roots and nodules was conducted using DESeq. 2, identified 3092 genes with significantly enhanced expression in nodules (Table S16; Figure S4). Gene Ontology (GO) and Kyoto Encyclopedia of Genes and Genomes (KEGG) enrichment analyses of these nodule‐enhanced genes revealed significant enrichment in functions related to sugar metabolism and photosynthesis (Tables S17, S18) (Figure S5A,B). These findings underscore the critical role of photosynthesis‐related pathways in RNS, likely through regulating carbon allocation to nodules and thereby influencing RNS efficiency in *A. glutinosa*. To highlight conserved versus lineage‐specific components of the nodule transcriptional programme, we compared nodule‐enhanced genes across *Alnus*, legumes and other actinorhizals at the orthogroup level. This analysis revealed a shared core of 452 nodule‐enhanced orthogroups across all three lineages, together with substantial lineage‐specific sets (1223 Alnus‐specific, 3030 legume‐specific and 1832 other‐actinorhizal‐specific orthogroups) and additional pairwise overlaps (Figure S21).

### 2.3 Lineage‐Specific Adaptation of Haemoglobin 1 in *Alnus*

**Figure 3:**
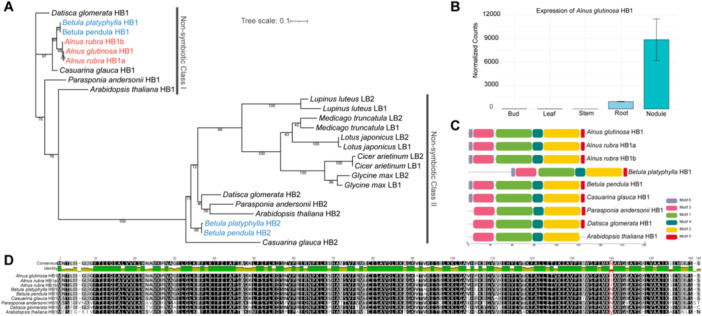
*Alnus*‐specific adaptations in non‐symbiotic class 1 haemoglobins. (A) Phylogenetic analysis of non‐symbiotic class 1 haemoglobins (nsHB1) and non‐symbiotic class 2 haemoglobins (nsHB2). Haemoglobin genes from *Alnus glutinosa* and *Alnus rubra* are shown in red, while those from *Betula pendula* and *Betula platyphylla* are shown in blue. Haemoglobin sequences from other Fagales and legume species were retrieved from UniProt (reviewed entries only), and their classifications into Class 1 and Class 2 are well established. (B) Expression profile of the *A. glutinosa* nsHB1 gene across different organs. The x‐axis represents five organs (bud, leaf, stem, root and nodule), and the *y*‐axis shows read counts normalised using DESeq. 2. (C) Conserved motif analysis of nsHB1 proteins from Alnus and other reference species. A total of six conserved motifs were identified, each shown in different colours. (D) Multiple sequence alignment of nsHB1 proteins from Alnus and reference species. Red boxes highlight amino acid residues that are conserved in *Alnus* but variable among other species, suggesting *Alnus*‐specific sequence features. [Color figure can be viewed at wileyonlinelibrary.com]

Haemoglobins are widely distributed in plant species capable of RNS, where they play a pivotal role in oxygen transport and regulation. By maintaining a microaerobic environment within the nodule, plant haemoglobins protect the oxygen‐sensitive bacterial nitrogenase enzyme, thus enabling efficient nitrogen fixation (Appleby et al. 1988). Plant haemoglobins are generally classified into two main types: non‐symbiotic class 1 haemoglobin (nsHB1) and non‐symbiotic class 2 haemoglobin (nsHB2). In legumes, the leghemoglobins specifically utilised for RNS belong to nsHB2 and are highly specialised for oxygen buffering in nodules (Larrainzar et al. 2020). However, previous molecular studies have shown that some non‐legume nodulators, including *Alnus firma*, *Parasponia andersonii* and *Casuarina glauca*, employ nsHB1 (Kortt et al. 1988; Sasakura et al. 2006; Sturms et al. 2010). In spite of the presence of haemoglobin in the nodules of *A. glutinosa*, the specific type of haemoglobin involved in its symbiosis has remained unresolved (Suharjo and Tjepkema 1995). To clarify this, we identified haemoglobin genes from the *A. glutinosa* genome and constructed a maximum‐likelihood phylogenetic tree using homologous sequences from representative plant species (Table S19, Figure 3). The resulting phylogeny revealed that both *A. glutinosa* and *A. rubra* cluster exclusively with nsHB1, confirming their recruitment of nsHB1 rather than nsHB2. Transcriptomic data from *A. glutinosa* nodules further support the active expression of nsHB1, highlighting its functional involvement in symbiosis (Figure 3B). Interestingly, the phylogenetic tree also revealed that both *Alnus* species have lost nsHB2, which remains conserved in most other plant lineages. This suggests a unique, lineage‐specific loss of nsHB2 in *Alnus*. Motif analysis across nsHB1 sequences from multiple species reveals that, compared to nsHB1 in *A. thaliana*, nsHB1 in non‐legume nodulators possesses a unique motif structure, while an additional distinct motif structure has emerged specifically in Fagales (Figure 3C, Figure S6A,B). This suggests that the nsHB1 is likely to undergo lineage‐specific adaptations through the acquisition of novel motif structures. Multiple sequence alignment of nsHB1 proteins further revealed a putative *Alnus*‐specific amino acid substitution: isoleucine (Ile) at position 140 (Figure 3D). Structural modelling further suggests that the Fagales‐specific N‐terminal motif forms an exposed extension, and that the *Alnus*‐specific Ile substitution contributes to a local hydrophobic surface patch (Figure S16), which may alter the local physicochemical environment and thereby influence nsHB1 function. Notably, a similar Ile substitution is also observed in *P. andersonii*, another non‐legume nodulator. Previous biochemical studies have demonstrated that the isoleucine side chain can interact with the proximal histidine residue within the same subunit, contributing to structural heterogeneity and potentially modulating oxygen‐binding affinity (Kakar et al. 2011). This parallel amino acid change across distantly related non‐legume lineages may reflect convergent evolution driven by the functional constraints of RNS. Taken together, our results demonstrate that *Alnus* species uniquely recruit nsHB1 for RNS and have undergone both gene loss and protein‐level adaptations in their haemoglobin repertoire. These findings provide compelling evidence for lineage‐specific molecular innovation in haemoglobin function, offering important insights into the evolutionary flexibility of RNS‐related proteins in non‐legume lineages such as Fagales and *Alnus*.

### 2.4 Presence–Absence and CNVs of RNS‐Related Genes Underpin the RNS of *A. glutinosa*

**Figure 4:**
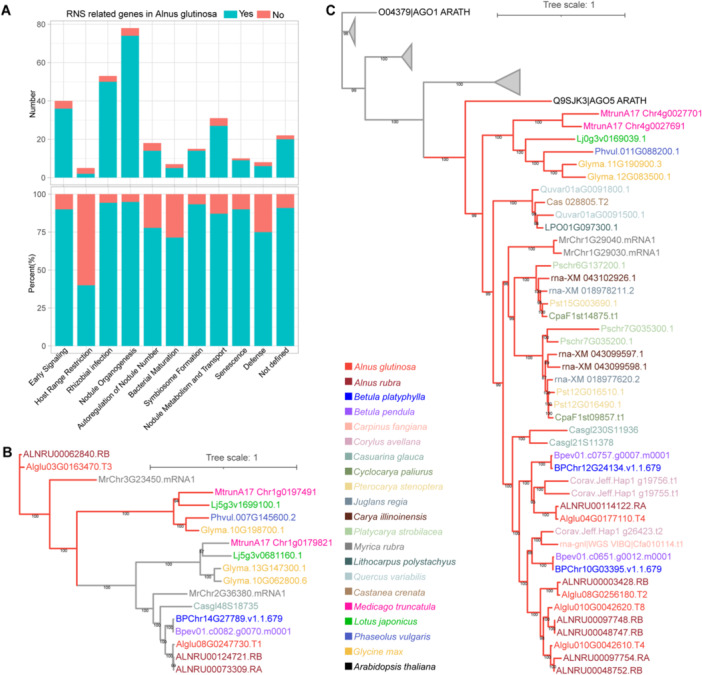
Evolutionary dynamics of RNS‐related genes in *Alnus glutinosa* and Fagales. (A) Overview of the presence and absence of orthologs of 361 experimentally validated reference RNS‐related genes in *A. glutinosa*. Blue bars indicate the presence of orthologs, while red bars indicate their absence. The *y*‐axis shows both the number and proportion of genes present or absent. (B) Phylogenetic analysis of *RPG* homologues in representative Fagales species and four legume reference species. Red branches indicate the orthologous clade of *RPG*, while grey branches represent paralogous clades. (C) Phylogenetic analysis of the *AGO* gene family in representative Fagales species and four legume reference species. Red branches represent the orthologous clade of *AGO5*, while grey compressed branches represent orthologous clades of other *AGO* genes. [Color figure can be viewed at wileyonlinelibrary.com]

Compared to the extensive studies on the molecular mechanisms of the RNS in legumes, research on actinorhizal RNS has been relatively limited (Van den Bulcke et al. 2019). However, due to the deep evolutionary homology between actinorhizal and legume RNS, it is likely that both symbiotic systems co‐opt a shared set of core genes, enabling informative cross‐lineage comparative analyses. This evolutionary conservation provides a valuable opportunity to investigate RNS‐related genes in *Alnus* using knowledge derived from model legumes. We compiled a comprehensive set of 361 experimentally validated RNS‐related genes from four well‐characterised legume species—*Medicago truncatula*, *Lotus japonicus*, *Glycine max* and *Phaseolus vulgaris* (Table S20). Using the orthogroups inferred by OrthoFinder, we constructed maximum‐likelihood phylogenetic trees for each group with IQ‐TREE2, focusing specifically on orthogroups containing known RNS reference genes (Dataset S1). To improve accessibility of the curated phylogenies, we provide a consolidated summary table indexing the 275 orthogroup trees and their key annotations (Table S28). To evaluate the overall robustness of orthogroup phylogenies used for ortholog assignment, we quantified gene‐tree concordance across curated RNS‐related orthogroups using gene concordance factors (gCF), which showed a broad but generally moderate‐to‐high support across internal branches (Figure S15). Within these phylogenies, *A. glutinosa* genes clustering in the same clades as reference genes were identified as putative orthologs. Notably, approximately 89% of RNS‐related genes were conserved in *A. glutinosa*, underscoring a high degree of evolutionary conservation. Nevertheless, lineage‐specific gene losses were observed at each symbiotic stage (Table S21). Key genes such as *NIN*, known to be essential in legume nodulation, were retained, further supporting their central role across diverse RNS lineages (J. Liu and Bisseling 2020). In contrast, gene loss was most prominent at the host range restriction stage, where over 50% of genes were absent in *A. glutinosa* (Figure 4A). This pattern is consistent with the broad host compatibility of *Frankia*, suggesting that *A. glutinosa* may rely on a more generalised recognition system, rendering certain host‐specific genes redundant and prone to loss (Suman 2021).

To assess whether the presence–absence variation (PAV) of key RNS‐related genes may underlie the retention of RNS in *Alnus* but not in the closely related *Betula*, we examined orthogroups containing RNS‐related genes present in *Alnus* but absent in *Betula*. Candidate orthologs were initially identified from phylogenetic clades containing known RNS‐related genes (Table S22). To mitigate potential false negatives from automated orthology assignments, we extended the candidate pool by retrieving the top five BLASTP hits of each RNS‐related gene in *B. pendula* and other representative Fagales genomes. After manual curation, the *RPG* (Rhizobium‐directed Polar Growth) gene emerged as the only gene with robust phylogenetic and genomic support for presence in *Alnus* and absence in *Betula* (Figure 4B). *RPG* encodes a leucine‐rich repeat protein critical for infection thread development during rhizobial entry. Loss‐of‐function mutations in *RPG* are known to severely impair rhizobial infection and subsequent nodule development (X. Li et al. 2023; Arrighi et al. 2008). Consistent with its presumed function, transcriptomic analysis revealed that the putative *AgluRPG* (Alglu03G0163470) in *A. glutinosa* is exclusively expressed in nodules, supporting its identification as a true ortholog (Figure S7A). In contrast, the *RPG* paralog (Alglu08G0247730) exhibited low expression in nodules and was broadly expressed across all five examined organs, consistent with its classification as a paralog. To further address whether nodule‐enriched expression reflects symbiosis induction rather than constitutive root development, we performed a cross‐study validation using two canonical markers, *AgluHB1* and *AgluRPG*. Integrating public RNA‐seq data from uninoculated roots revealed that both genes are virtually undetectable in non‐symbiotic roots and remain at basal levels in inoculated roots, whereas they show robust expression exclusively in nodules (Figure S17) (Y. Zhang et al. 2024). These findings suggest that retention of RPG may contribute to the maintenance of RNS in Alnus, although causal roles remain to be tested experimentally. However, its presence alone may not fully explain the capability, as *RPG* has been independently lost in other RNS‐capable species, such as *Casuarina glauca* (Griesmann et al. 2018), indicating the existence of alternative evolutionary routes for RNS. qRT–PCR further supported strong nodule‐enriched expression of RPG, whereas transcript levels in roots were extremely low (Table S24; Figure S14A).

In addition to PAV, gene CNV is recognised as a major driver of evolutionary innovation, often conferring functional plasticity (Prunier et al. 2017). To determine whether CNV has contributed to RNS evolution in *Alnus*, we identified orthogroups where *Alnus* possesses more gene copies than *Betula*. To validate these duplications and exclude spurious orthogroup assignments, we performed genome‐wide searches using *A. thaliana* homologues as queries and reconstructed expanded phylogenies with broader candidate sets to ensure robust clustering. Among these, we identified the *AGO5* (Argonaute5) gene family as being notably expanded in *Alnus* compared to all representative species of both Fagales and Fabales (Figure 4C). AGO5 is a key protein involved in RNA silencing pathways and has been demonstrated to play an essential role in the rhizobial infection process during the RNS. This protein facilitates plant‐microbe interactions and has been shown to be indispensable for RNS establishment in model legume species, including *Phaseolus vulgaris* and *Glycine max* (Reyero‐Saavedra et al. 2017). All four *AGO5* gene copies identified in *A. glutinosa* were actively expressed in nodules (Figure S8A–D), indicating that they have retained functional roles rather than becoming pseudogenes. To further assess the functional relevance of the duplicated AGO5 genes, we performed qRT–PCR analysis across different organs (Table S23). All four AGO5 copies exhibited detectable expression in *A. glutinosa* (Table S24; Figure S9A–D). Although transcript abundance varied among duplicates, all copies showed markedly higher expression in nodules compared with roots, suggesting that these duplicates are transcriptionally active and potentially involved in symbiotic nodulation. We further examined whether its paralogs show stage‐dependent regulation. By integrating published early‐stage RNA‐seq data (0 and 22 dpi) with our mature‐stage samples (12 wpi), we generated a developmental heatmap (Figure S22) revealing that *AGO5* paralogs are expressed in mature roots and nodules.

Together, these results highlight that both the retention of key RNS‐related genes such as *RPG* and the expansion of regulatory gene families such as *AGO5* may have contributed to the unique symbiotic competence of *A. glutinosa*. These genomic features provide new insights into the evolutionary mechanisms that underlie the presence or absence of RNS across closely related species within Fagales.

### 2.5 Two TFs Emerge as Candidates for RNS Regulation in *A. glutinosa*

**Figure 5:**
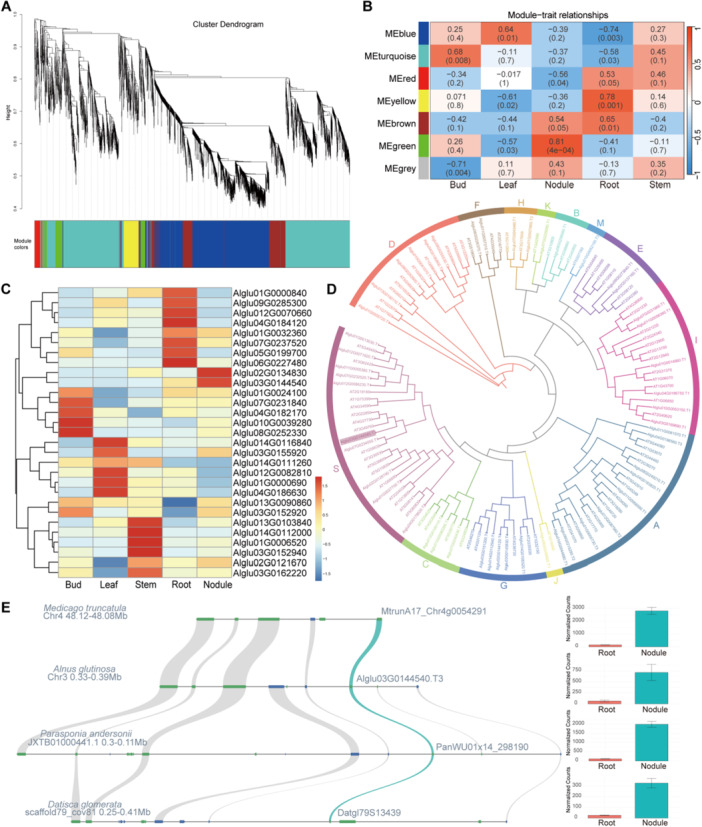
Identification of the nodule‐specific module and functional characterisation of the *AglubZIP‐TF1* (Alglu03G0144540) in *Alnus glutinosa*. (A) All genes in *A. glutinosa* were clustered based on their expression profiles across five organs (bud, leaf, stem, root and nodule) and assigned to distinct co‐expression modules. A total of seven modules were identified. (B) Correlation between the seven co‐expression modules and organ‐specific traits. The numbers in each box represent the correlation coefficient and the corresponding *p* value. (C) Expression profiles (FPKM values) of all transcription factors in the green module across the five organs. (D) Phylogenetic tree of all bZIP protein sequences from *A. glutinosa* and *Arabidopsis thaliana*. Groups were classified based on known *A. thaliana* bZIP subfamilies. The *AglubZIP‐TF1* (Alglu03G0144540) is highlighted in purple. (E) Microsynteny analysis of the *AglubZIP‐TF1* and its orthologs in three representative nodulators (*Datisca glomerata*, *Parasponia andersonii* and *Medicago truncatula*), along with their expression profiles in roots and nodules. [Color figure can be viewed at wileyonlinelibrary.com]

To identify candidate regulators of RNS in *A. glutinosa*, we performed a WGCNA, which groups genes with similar expression patterns into modules and enables the discovery of key genes associated with specific biological traits (J. Liu et al. 2016). The analysis was based on normalised fragments per kilobase of transcript per million mapped reads (FPKM) values obtained from transcriptomes of five organs—buds, leaves, stems, roots and nodules at 12 weeks post‐inoculation. One stem sample identified as an outlier by PCA was excluded to improve network quality. Using an optimal soft‐thresholding power, we constructed a signed co‐expression network and identified seven distinct gene modules (Figure 5A; Figure S10A–C). Module‐trait association analysis revealed that the green module was strongly and significantly correlated with nodule tissue ( | *r* | > 0.7; *p* < 0.05) (Figure 5B). This nodule‐specific module (NSM) comprises 231 genes, representing a candidate set of genes potentially involved in nodule function, although their expression levels varied across tissues (Table S25; Figure S11). Functional enrichment analysis of genes in the NSM revealed significant overrepresentation of GO terms related to starch metabolism. This suggests that sugar‐related metabolic pathways play a central role in the RNS of *A. glutinosa*, consistent with prior observations in legumes (Figure S12A,B) (Reid et al. 2011). Functional mining of the NSM revealed a synchronised carbon network. We identified a sugar transporter and a Molybdate transporter strictly co‐expressed with Sucrose Synthase (*SUS4*), glycolysis enzymes (PFK, PFP) and a complete starch cycling pathway (e.g., *AMY5, PWD1, PHO1*) (Figure S20, Table S26). This transcriptional coupling suggests that nodules are genetically programmed as active carbon sinks to fuel nitrogen fixation (Udvardi and Poole 2013; Gordon et al. 1999; Tejada‐Jiménez et al. 2013). Similar WGCNA‐based studies in soybean have also shown that modules enriched for sugar metabolism are closely associated with nodulation, further supporting a deeply conserved role of sugar pathways in RNS across legumes and non‐legumes (Piya et al. 2023). Among the 29 TFs identified within the NSM, a GOLDEN2‐LIKE (*AgluGLK‐TF1*; Alglu02G0134830) and a bZIP (*AglubZIP‐TF1*; Alglu03G0144540) were significantly upregulated in nodules compared to roots (Figure 5C), suggesting that they may serve as key regulators in the RNS of *A. glutinosa*. Consistent with the RNA‐seq profile, qRT–PCR validated nodule‐enhanced expression of the bZIP TF; transcripts in roots were below the detection limit (ND) (Table S24; Figure S14B).

GLK is a type of light‐mediated TF that regulates the expression of photosynththat regulates the expression of photosynthesis‐related genes, controls chloroplast development and differentiation and maintains chloroplast function (Waters et al. 2008). *AgluGLK‐TF1* elevated expression in nodules points to a potential dual role in coordinating photosynthetic activity and supporting the energy demands of RNS. Moreover, nodule‐enhanced genes in *A. glutinosa* were also significantly enriched in chloroplast‐ and photosynthesis‐related GO terms, underscoring a close functional relationship between photosynthesis and RNS in this species. Phylogenetic analysis of the *AglubZIP‐TF1* indicated that it belongs to the Group S clade, a subgroup broadly induced by sugar signals in *A. thaliana* (Figure 5D) (Dröge‐Laser et al. 2018). Intriguingly, this bZIP TF was specifically expressed in the nodules of multiple representative species across Rosales, Cucurbitales and Fabales, suggesting a conserved role, subject to functional validation. (Figure 5E). To further investigate the regulatory role of *AglubZIP‐TF1* in *A. glutinosa*, we selected the *A. thaliana bZIP11* gene (*AtbZIP11*), one of the closest orthologs with characterised cis‐regulatory binding motifs, to identify potential target genes within the NSM of *A. glutinosa*. Notably, binding sites for the *AtbZIP11* were detected in the upstream regions of 81 genes within the NSM—approximately 35% of the entire gene set (Table S27). Together, these results highlight the critical regulatory roles of *AgluGLK‐TF1* and *AglubZIP‐TF1* in the RNS of *A. glutinosa*, bridging sugar signalling, photosynthesis and symbiotic gene networks. These findings not only deepen our understanding of transcriptional regulation in *A. glutinosa* RNS but also support the hypothesis of conserved regulatory architectures underlying RNS across legumes and non‐legumes. These findings are summarised in Figure S23 as a conservative working model. The NSM (WGCNA Green) highlights bZIP and GLK together with coordinated carbon metabolism genes (MFS, SUS4, PFK/PFP, AMY5/ISA3/PHO1/2/DPE2/PWD1 and a molybdate transporter), while nsHB1 recruitment, RPG retention (PAV/retained), and AGO5 CNV expansion are included as additional candidate components of RNS in *A. glutinosa*.

## 3 Discussion

As one of the few non‐legume nodulators, *Alnus glutinosa* has long been a focal point in studies on the evolution of root nodule symbiosis (Zitzer and Dawson 1992). Research on RNS in legumes is extensive, but research on the RNS of non‐legumes remains limited, largely due to the lack of high‐quality reference genomes and transcriptome data. In earlier work, a draft genome of *A. glutinosa* was assembled and used as a representative of nodulating Fagales species to support the hypothesis of a single origin of RNS (Griesmann et al. 2018). However, these earlier assemblies were limited by the sequencing technologies of the time, resulting in fragmented genomes. Moreover, the absence of organ‐specific transcriptome data has hindered deeper comparative and functional genomic analyses. In this study, we generated a high‐quality, chromosome‐scale genome assembly of *A. glutinosa* using PacBio HiFi sequencing combined with Hi‐C scaffolding. We also produced organ‐specific transcriptomes from leaves, stems, shoot apices, roots and mature nodules (12 weeks post‐inoculation). The resulting genome assembly has a scaffold N50 of 31 Mb, with 89.81% of the assembled sequence anchored to 14 pseudochromosomes. BUSCO analysis indicated that 98.70% of conserved plant orthologs are complete, underscoring the high completeness and quality of the assembly.

To explore the evolutionary trajectory of RNS, we conducted large‐scale comparative genomic analyses. We selected the high‐quality genome assemblies from each sequenced genus within Fagales, along with four well‐characterised legume species, to construct orthogroups, infer species phylogeny and analyse gene family expansions and contractions. The resulting phylogeny reaffirmed the close sister‐group relationship between the genera *Alnus* and *Betula* (Y.‐Y. Yang et al. 2021). As nodulating and non‐nodulating sister taxa, their comparison provides a powerful framework for identifying lineage‐specific adaptations related to RNS in *Alnus*. Gene family evolution analysis revealed expansions in functions related to terpenoid metabolism and malate transport. In legumes, malate is known to be a key carbon source for nodules (Booth et al. 2021), and overexpression of terpenoid biosynthesis genes has been shown to promote nodulation in soybean (Ali et al. 2021; 2022). These gene expansions in *A. glutinosa* suggest potential adaptive mechanisms enhancing RNS and nitrogen fixation efficiency.

Functional enrichment analysis of nodule‐enhanced genes in *A. glutinosa* revealed significant overrepresentation of sugar metabolism and photosynthesis‐related pathways. Previous studies have demonstrated that light intensity significantly influences nitrogen fixation efficiency in this species (Wheeler and Bowes 1974; Dawson and Gordon 1979). While only a few genes have been identified as shared between RNS and photosynthesis, such as *CRY1* (T. Wang et al. 2021), our findings reveal a much broader overlap in functional terms, suggesting a deeper integration of carbon and nitrogen metabolism during RNS. This highlights an underexplored aspect of RNS biology and points to the need for future work to dissect the regulatory networks underlying this integration.

In legumes, maintenance of a low‐oxygen environment within nodules is typically achieved via leghemoglobins, belonging to the nsHB2. In contrast, previous studies have shown that *Alnus firma* recruits the nsHB1 (Sasakura et al. 2006). Here, we provide genome‐level phylogenetic evidence for the exclusive recruitment of nsHB1 in *A. glutinosa*, and transcriptome data confirmed its specific expression in nodules, consistent with reports in other non‐legume nodulators like *Datisca glomerata* and *Casuarina glauca* (Jokipii‐Lukkari et al. 2009; Jacobsen‐Lyon et al. 1995). nsHB2 is proposed to be non‐essential for RNS, as evidenced by its absence of expression in *Parasponia andersonii* (Van Velzen et al. 2018). However, other studies suggest it may improve oxygen diffusion and enhance symbiosis efficiency (Gupta et al. 2011). Our study reveals convergent evolution of nsHB1 recruitment across diverse non‐legume nodulators and lineage‐specific loss of nsHB2 in *Alnus*. Whether nsHB2 is generally dispensable across non‐legumes requires broader functional evidence. Although direct mutational validation is not yet feasible in *Alnus*, these structural inferences provide a testable hypothesis for future heterologous mutagenesis assays.

PAV and copy number variation (CNV) analyses of RNS‐related genes offer key insights into trait evolution. We compiled a comprehensive list of RNS‐related genes and manually examined orthogroups across Fagales and legumes. Genes present in *Alnus* but absent in *Betula* were identified as potential PAV genes, while those with higher copy numbers in *Alnus* compared to *Betula* were considered potential CNV genes. To ensure accuracy, we applied a ‘BLAST+Phylogeny’ pipeline to refine ortholog assignments and excluded pseudogenes based on expression evidence. This analysis identified the *RPG* in *Alnus* representing a PAV gene and the *AGO5* exhibiting *Alnus*‐specific CNV. *RPG* has been widely studied for its essential role in infection thread formation and its recurrent loss in non‐nodulators. The loss of *RPG* in *Casuarina glauca* likely reflects its reliance on intercellular entry rather than the root‐hair infection pathway used by *Alnus (*Griesmann et al. 2018; Svistoonoff et al. 2014). Importantly, we found *RPG* to be a single‐copy gene in *Alnus*, confirming it functions as a non‐redundant, essential component of the intracellular infection machinery, distinct from the evolutionary route taken by *Casuarina*. Although *AGO5* is also crucial for RNS (Sánchez‐Correa et al. 2022), its evolutionary history has been less explored. Notably, *Alnus* exhibits the highest copy number of *AGO5* among all surveyed Fagales and legumes. Consistent with the genomic evidence of gene family expansion, our qRT–PCR analysis confirmed that all four AGO5 duplicates are transcriptionally active in nodules. This observation highlights the potential dosage effects of AGO5 duplicates in sustaining effective RNS in *A. glutinosa*.

To further explore potential RNS regulators, we performed WGCNA using organ‐specific transcriptomes. This approach has previously been used to identify NSM in *Glycine max* (Piya et al. 2023). In *A. glutinosa*, we identified an NSM of 231 genes that showed a strong correlation with nodule samples. GO enrichment analyses highlighted sugar‐related metabolism as the predominant functional category, reinforcing its central role in RNS. Among 29 transcription TFs in this NSM, only *AgluGLK‐TF1* and *AglubZIP‐TF1* exhibited nodule‐specific high expression. The GLK, typically associated with chloroplast development and photosynthesis, has been shown to promote root photosynthesis in *A. thaliana* (Kobayashi et al. 2013), implying a possible role in enhancing carbon use efficiency within nodules. However, its precise function in nodules remains uncharacterised. In *A. thaliana*, *bZIP* genes are typically classified into 13 groups (H. Li et al. 2020), and phylogenetic analysis placed the *AglubZIP‐TF1* within the group S, a clade known for sugar responsiveness in *A. thaliana* (Weltmeier et al. 2009). Since sugar‐related metabolism is the predominant function of the NSM, the *AglubZIP‐TF1* may serve as a key regulatory factor within this module. This hypothesis is further supported by the presence of TF binding sites for the group S *A. thaliana bZIP11* gene in the upstream regions of 35% of the NSM genes. Furthermore, this *bZIP* gene shows conserved nodule‐specific expression in *Medicago truncatula*, *Datisca glomerata* and *Parasponia andersonii*—representatives of Fabales, Cucurbitales and Rosales—highlighting it as a deeply conserved regulator of RNS within the nitrogen‐fixing clade. The strict co‐expression of sugar and molybdate transporters with sucrose and starch metabolism enzymes provides molecular evidence for active carbon allocation. This suggests that the host plant coordinately regulates the import of carbon and nitrogenase cofactors to support nitrogen fixation.

In conclusion, our study presents the most complete genome assembly to date for *A. glutinosa*, along with the high‐quality multi‐organ transcriptomic dataset. These resources enabled us to identify lineage‐specific adaptations in RNS‐related genes and reveal novel regulatory elements in *A. glutinosa* RNS. Our work not only sheds light on the evolutionary and molecular mechanisms underlying RNS in this non‐legume species but also provides a valuable foundation for establishing *A. glutinosa* as a model for future studies on actinorhizal RNS and Fagales evolution.

## 4 Materials and Methods

### 4.1 Plant Materials and Growth Conditions

Seeds of *Alnus glutinosa* were collected from a mature tree growing along the Rhône River in Lyon, France. Seedlings were cultivated in nutrient‐rich soil under controlled greenhouse conditions at Dapeng New District, Shenzhen, Guangdong Province, China. Plants were maintained at a constant temperature of 21°C with a 16‐h light/8‐h dark photoperiod. At 180 days post‐germination, seedlings were inoculated with *Frankia alni* strain AG, which was originally isolated from nodules of the same *A. glutinosa* source tree. Inoculation was performed by syringe injection at the root zone. Twelve weeks after inoculation, when nodules had reached full maturity, we harvested nodules along with roots, leaves, stems and apical buds from the inoculated plants. All collected tissues were briefly rinsed with 75% ethanol, blotted dry with sterile filter paper and immediately flash‐frozen in liquid nitrogen. Leaf samples were divided into two portions: one for genomic DNA extraction and the other for RNA extraction. RNA was also extracted from roots, nodules, stems and apical buds.

### 4.2 Histological Analysis of Nodule Sections

Freshly harvested nodules were fixed in 5% glutaraldehyde prepared in 0.1 M phosphate buffer (pH 7.2) under vacuum for 1–2 h. After fixation, samples were washed four times with 0.1 M phosphate buffer (15 min each) and rinsed in distilled water for 15 min. Tissues were dehydrated through a graded ethanol series (10%, 30%, 50%, 70%, 90% and 100%), each for 10 min, and embedded in Technovit 7100 resin (Kulzer, Germany) according to the manufacturer's instructions. Semi‐thin sections (5–10 μm) were cut using a rotary microtome. Sections were stained with 0.05% Trypan Blue in 0.1 M phosphate buffer (pH ~6.8) for 1–2 min, briefly rinsed in distilled water, air‐dried and mounted on glass slides using Euparal mounting medium (Carl Roth, Germany). Stained sections were examined under bright‐field microscopy using a Leica DM500 microscope. Digital images were captured using a Leica MC190 HD camera system (Franssen et al. 2015).

### 4.3 Genome and Transcriptome Sequencing

High‐quality genomic DNA was extracted from young leaf tissues of *A. glutinosa* using a modified cetyltrimethylammonium bromide protocol optimised for long‐read sequencing. DNA quantity and purity were assessed using a NanoDrop spectrophotometer (Thermo Fisher Scientific), a Qubit Fluorometer (Thermo Fisher Scientific) and agarose gel electrophoresis to confirm high molecular weight and integrity. A SMRTbell library was constructed following the standard protocol provided by Pacific Biosciences (Menlo Park, CA, USA). Size selection was performed using the SageELF system (Sage Science) to enrich for DNA fragments exceeding 15 kb. The final library was sequenced on the PacBio Sequel II platform using Circular Consensus Sequencing (CCS) mode. Raw subreads were processed using SMRT Link v10.1 to generate high‐fidelity (HiFi) CCS reads.

To facilitate chromosome‐level scaffolding, Hi‐C sequencing was performed on fresh *A. glutinosa* leaf tissues. Tissues were crosslinked in 1% formaldehyde to preserve chromatin conformation, followed by quenching with 0.125 M glycine. Nuclei were isolated, and chromatin was digested with the restriction enzyme MboI. Sticky ends were biotin‐labelled and subjected to proximity ligation under dilute conditions to favour intramolecular ligation. Crosslinks were then reversed, and DNA was purified. Biotin‐labelled ligation products were selectively enriched using streptavidin beads. Library construction was carried out according to Illumina Hi‐C library preparation protocols, and sequencing was performed on the Illumina NovaSeq. 6000 platform by Frasergen Bioinformatics Co. Ltd. (Wuhan, China), generating paired‐end reads.

Total RNA was extracted from nodules, roots, leaves, stems and shoot apices of *A. glutinosa* using TRIzol reagent (Invitrogen, USA), following the manufacturer's protocol. For each tissue type, three biological replicates were collected, each derived from a different individual plant to ensure biological variability. RNA concentration and purity were measured with a NanoDrop spectrophotometer (Thermo Fisher Scientific), and RNA integrity was assessed using an Agilent 2100 Bioanalyzer (Agilent Technologies, USA). Polyadenylated mRNA was isolated using oligo (dT) magnetic beads, fragmented and reverse‐transcribed into first‐strand cDNA, followed by second‐strand synthesis. The resulting double‐stranded cDNA was purified, end‐repaired, adenylated and ligated to Illumina sequencing adapters. After size selection and PCR amplification, libraries were sequenced on the Illumina NovaSeq. 6000 platform to generate 150 bp paired‐end reads.

### 4.4 Genome Assembly and Annotation

PacBio HiFi reads were used for de novo genome assembly, utilising hifiasm (version 0.24.0‐r702) (Cheng et al. 2021) with default parameters, resulting in a primary contig‐level genome assembly of *A. glutinosa*. To further construct chromosome‐scale scaffolds, Hi‐C sequencing data were first processed using Juicer (version 1.6) for read alignment and generation of Hi‐C contact maps. The draft contig assembly was then scaffolded using the 3D‐DNA pipeline (version 190716), which automated misjoin correction, contig ordering and orientation based on Hi‐C contact frequency (Dudchenko et al. 2017). Subsequently, manual curation was conducted using Juicebox Assembly Tools (version 1.11.08) to correct potential misassemblies and refine the final arrangement of contigs, yielding a high‐quality chromosome‐level genome assembly (Durand et al. 2016). To evaluate scaffolding integrity, we analysed Hi‐C contact probability as a function of genomic distance using the final Hi‐C contact map at 50 kb resolution. The.hic file was converted to.cool format with hic2cool (v0.8.3) (Rossini and Paulsen 2024), and distance‐dependent contact decay was computed with HiCExplorer (hicPlotDistVsCounts, v3.7.6) (Wolff et al. 2022) on a log–log scale.

The identification and classification of repetitive sequences in the *A. glutinosa* genome were performed using EDTA (version 1.9.6) with default parameters (Ou et al. 2019). EDTA integrates structure‐based and homology‐based approaches to comprehensively annotate various classes of transposable elements. The final non‐redundant repeat annotation was used for subsequent genome masking and gene prediction.

Gene prediction was performed following the pipeline described with identical parameters and workflows (H. Liu et al. 2024). Briefly, it was conducted through MAKER3 integrating RNA‐seq (HISAT2/StringTie), protein homology (Swiss‐Prot/*Betula pendula*/*Carpinus fangiana*/*Arabidopsis thaliana*) and ab initio predictions (BRAKER3/AUGUSTUS) (Kim et al. 2019; Pertea et al. 2015; Gabriel et al. 2024; Stanke et al. 2006). The gene functional annotation was carried out using their longest protein isoforms as queries. Initial homology searches were performed against the eggNOG v5.0 database employing the fast sequence aligner Diamond with sensitivity‐optimised parameters (Huerta‐Cepas et al. 2019; Buchfink et al. 2021). Subsequent functional classification was achieved through the eggNOG‐mapper v2 online tool (Cantalapiedra et al. 2021), which provides integrated annotations from established GO, KEGG, Pfam and CAZy databases.

### 4.5 Synteny and Synonymous Substitution Rate Value Estimation

Whole‐genome alignments between *Alnus* and *Betula* were conducted using MUMmer4, and dot plots were generated using mummerplot (Marçais et al. 2018). Syntenic blocks among different species were identified using the MCScanX (Y. Wang et al. 2012), with a minimum of five colinear genes required to define a syntenic block. Macrosynteny relationships are visualised using NGenomeSyn between *Alnus* and *Betula* to assess large‐scale chromosomal conservation (He et al. 2023). Syntenic gene pairs, both within and between species, were extracted from the JCVI results and used for synonymous substitution rate (Ks) estimation (Tang et al. 2024). Pairwise protein alignments were first performed using ParaAT (version 2.0) with ClustalW2 as the alignment algorithm, and the corresponding protein alignments were converted to codon‐based CDS alignments to generate axt format files. The KaKs_Calculator (version 2.0) with the YN (Yang‐Nielsen) method was then employed to calculate Ks values for each homologous gene pair (Z. Zhang et al. 2012; Larkin et al. 2007; D. Wang et al. 2010). Gene pairs with Ks values greater than 0.0005 were retained for further analysis. Finally, the distribution of Ks values was plotted using R and the ggplot2 package to illustrate the evolutionary divergence patterns within and between species (Villanueva et al. 2019).

### 4.6 Orthogroup Inference and Species Tree Reconstruction

A total of 20 species were used for comparative analysis, including members of Fagales: *A. glutinosa*, *A. rubra*, *B. platyphylla*, *C. fangiana*, *C. illinoinensis*, *C. crenata*, *C. glauca*, *C. avellana*, *C. paliurus*, *J. regia*, *L. polystachyus*, *M. rubra*, *P. stenoptera*, *Q. variabilis* and legumes: *G. max*, *L. japonicus*, *M. truncatula*, *P. vulgaris*, as well as the outgroup *A. thaliana*. We used OrthoFinder v2.5.4 with default settings for orthogroup inference and species tree reconstruction (Emms and Kelly 2018). An all‐vs‐all sequence similarity search was performed using DIAMOND, and the orthogroups (OGs), which contain putative orthologous and paralogous genes, were inferred using the Markov Clustering algorithm. The species tree was inferred based on the concatenated alignments of single‐copy orthologs identified across the 20 species with FastTree (Price et al. 2009). The phylogenetic tree was visualised and annotated using the iTol software (Letunic and Bork 2021).

### 4.7 Divergence Time Estimation

A total of 122 single‐copy OGs identified by OrthoFinder were selected for molecular clock analysis. The corresponding protein sequence alignments from the MultipleSequenceAlignments directory were converted into codon‐based nucleotide alignments using PAL2NAL (version v14) (Suyama et al. 2006). The codon alignments of all OGs were concatenated into a supermatrix, and alignment gaps were removed. The final alignment contained 35,502 nucleotides for each species. Divergence time estimation was performed using MCMCTREE in the PAML package (version 4.10.7) (Z. Yang 2007). The Markov chain Monte Carlo (MCMC) analysis was run with the following parameters: burn‐in = 2000, sample‐number = 10 and sample‐frequency = 20 000. Two fossil calibration points were used to constrain the analysis:

1. the divergence between *G. max* and *P. vulgaris* was set to 19.0–21.0 million years ago (Mya) (F. Zheng 2016a);
2. the divergence between *C. avellana* and the clade comprising *L. polystachyus* and *Q. variabilis* was constrained to 54.93–81.21 Mya (H. Liu et al. 2023).

### 4.8 Gene Family Expansion and Contraction Analysis

Gene family expansion and contraction were inferred using CAFE (version 5.0) (Mendes et al. 2020). The analysis was based on the inferred OGs, the constructed species phylogeny and divergence time estimates. A random birth‐and‐death model was applied to estimate the changes in gene family size along each branch of the phylogenetic tree. Gene families containing more than 100 gene copies in any single species were excluded from the analysis to reduce potential biases caused by abnormally large gene families. The model was tested under different ‐k values, where ‐k specifies the use of a Gamma distribution model to account for variable rates of gene family evolution. Model comparison based on the final log‐likelihood values (−lnL) indicated that the model with ‐k 2 provided the best fit to the data. Gene families with both family‐wide *p* values and Viterbi *p* values less than 0.05 were considered to have experienced significant expansion or contraction events. To clarify whether terpenoid‐related expansions are *Alnus*‐specific, we compared copy numbers of representative terpenoid‐associated OGs among *A. glutinosa*, *B. platyphylla* and *Q. variabilis*. OG membership was defined based on EggNOG‐mapper annotations and orthology assignment, and copy number was calculated as the number of protein‐coding genes assigned to each OG in each genome.

### 4.9 Differential Gene Expression Analysis Between Roots and Nodules

To assess gene expression in *A. glutinosa* nodules, total RNA was extracted and sequenced from mature nodules, along with root samples for comparison. RNA‐seq reads were aligned to the *A. glutinosa* reference genome using HISAT2 (version 2.0.2) (Kim et al. 2019), with the genome index constructed to include exon and splice site annotations to enhance alignment accuracy. Gene‐level read counts were obtained using featureCounts (version 1.5.0) (Liao et al. 2014). The raw count matrix was imported into DESeq. 2 (Love et al. 2014), where gene‐wise dispersions were estimated under a negative binomial model. Potential hidden technical variation was assessed by PCA and sample‐to‐sample distance heatmaps based on DESeq. 2 variance‐stabilised counts (Figure S13). Prior to differential expression analysis, read counts were normalised using the size factor method implemented in DESeq. 2 to correct for library size differences. Differential expression was assessed using the Wald test, and adjusted *p* values were calculated using the Benjamini–Hochberg procedure to control the false discovery rate at 5%. Genes with an adjusted *p* value ≤ 0.05 and an absolute log₂ fold change > 2 were considered significantly differentially expressed. For cross‐lineage comparisons, raw read count matrices for *Medicago truncatula*, *Lotus japonicus*, *Datisca glomerata* and *Hippophae rhamnoides* were obtained from the supplementary dataset of Libourel et al. (2023) (Libourel et al. 2023). Using the same DESeq. 2 workflow and thresholds described above, we defined nodule‐enhanced genes for each species by comparing nodule samples against the corresponding root controls. Nodule‐enhanced genes were then mapped to OrthoFinder‐inferred orthogroups, and overlaps were evaluated at the orthogroup level. Orthogroups from the two legume species were combined to represent ‘Legumes’, and orthogroups from the two actinorhizal species were combined to represent ‘Other actinorhizals’. Orthogroup overlaps were visualised as a three‐set Venn diagram.

### 4.10 Cross‐Study Expression Validation and Temporal Profiling

RNA‐seq data from uninoculated *A. glutinosa* roots were retrieved from a public dataset (CNP0004055) and used as an external negative control to validate symbiosis‐induced expression. For cross‐study comparisons, expression values were summarised as TPM to account for differences in sequencing depth and library preparation, and TPM values of the marker genes *AgluHB1* and *AgluRPG* were compared across uninoculated roots (external), inoculated roots (this study) and nodules (this study) (Figure S17). To examine the temporal regulation of *AGO5* paralogs, RNA‐seq data from roots at 0 dpi and from mixed root nodule material at 22 dpi were retrieved from the same public database and integrated with our mature samples (12 wpi root and nodule) (Y. Zhang et al. 2024). Gene‐level counts for *AGO5a–AGO5d* were CPM‐normalised within each dataset, and Z‐score standardised per gene across stages for visualisation as a heatmap (Figure S22).

### 4.11 Gene Set Enrichment Analysis

All protein‐coding genes of *A. glutinosa* were used as the background set for functional annotation, and their sequences were submitted to the eggNOG‐mapper online platform to retrieve associated GO terms and KEGG pathway identifiers. Enrichment analysis was then performed to assess the over‐representation of GO terms and KEGG pathways in the target gene set compared to the background set, using Fisher's exact test. GO terms and KEGG pathways with a *p* value less than 0.05 were considered significantly enriched, highlighting the biological processes, molecular functions, cellular components and metabolic pathways potentially associated with the target genes in *A. glutinosa*. The entire enrichment analysis was conducted using the analysis workflow provided by the Gidio Cloud Platform.

### 4.12 Identification and Phylogenetic Analysis of Haemoglobin Genes

The protein sequences of nsHB1 and nsHB2 from *C. glauca*, *P. andersonii*, *D. glomerata*, *A. thaliana* and the protein sequences of class 1 leghemoglobin (LB1) and class 2 leghemoglobin (LB2) from *Vigna unguiculata*, *Medicago truncatula*, *Lupinus luteus*, *Lotus japonicus*, *Glycine max* were downloaded from UniProt as reference sequences (UniProt Consortium 2018). For *A. glutinosa*, *A. rubra*, *B. pendula* and *B. platyphylla*, BLASTP databases were constructed using the complete protein sets of each species (McGinnis and Madden 2004). Reference haemoglobin protein sequences were queried against these databases, and the top‐scoring hit for each query was selected as the putative haemoglobin candidate for *Alnus* and *Betula* species. Multiple sequence alignment of all candidate sequences was performed using MAFFT (v7.505) with default parameters (Katoh 2002). A maximum likelihood phylogenetic tree was then constructed using IQ‐TREE (v2.2.2.7), and branch support was assessed with 1,000 ultrafast bootstrap replicates (Minh et al. 2020). The nsHB1 protein sequences identified through phylogenetic analysis were subsequently subjected to conserved motif discovery using MEME suite (v5.5.2) (Bailey et al. 2009). MEME was run with default parameters, except that the maximum number of motifs was set to 10. nsHB1 protein structures were predicted using AlphaFold (Jumper et al. 2021). Structural visualisation and surface rendering were performed in PyMOL to examine the spatial location of the Fagales‐specific motifs and the focal Ile site (DeLano 2002).

### 4.13 Manual Curation of RNS‐Related Orthogroups

To accurately identify orthologs of reference RNS‐related genes in *Alnus* and *Betula*, a total of 275 OGs containing known RNS‐related genes from four reference species were selected based on OrthoFinder inference results. For PAV analysis, each reference gene suspected of being a PAV case was queried against the protein datasets of other nodulators in Fagales and legumes using BLASTP with an e‐value threshold of 1e^−50^ to retrieve candidate homologues. For CNV analysis, the entire gene family corresponding to each putative CNV reference gene was used as a query and searched against the protein datasets of the 20 species in Fagales and legumes (e‐value < 1e^−50^). All retrieved candidate homologues, along with the corresponding reference sequences, were then subjected to maximum likelihood phylogenetic analysis using IQ‐TREE2, which automatically selected the best‐fit substitution model for each alignment. The presence, absence and CNVs of RNS‐related genes across all species were confirmed by manually inspecting the phylogenetic clades that contained the reference sequences. To quantify tree‐topology consistency across curated RNS‐related orthogroups, we calculated gCF using IQ‐TREE2. The inferred maximum‐likelihood gene trees from the 361 curated RNS‐related orthogroups were used to compute gCF values for internal branches of the species tree. The distribution of gCF values across branches was visualised as a boxplot and density plot (Figure S15).

### 4.14 Quantitative Real‐Time PCR (qRT–PCR)

Gene‐specific primers for *AGO5a*–*AGO5d*, *RPG and bZIP*were designed with Primer3 and, when possible, spanned exon–exon junctions (Untergasser et al. 2012). Total RNA from bud, stem, root and nodule was extracted using FastPure Plant Total RNA Isolation Kit (Polysaccharides & Polyphenolics‐rich) (Vazyme), quantified and integrity‐checked by 1% agarose gel. cDNA was synthesised from 1 µg RNA using HiScript III RT SuperMix (Vazyme). qPCR was performed on QuantStudio 5 with ChamQ SYBR qPCR Master Mix (Vazyme) in 20 µL reactions (10 µL 2× mix, 0.4 µL each primer at 10 µM, 2 µL cDNA [1:10], 7.2 µL water): 94°C 30 s; 45 cycles of 94°C 5 s, 60°C 30 s; melt curve to confirm specificity. Actin2 served as a reference. Relative expression was calculated as 2^−ΔCt^ (ΔCt = Ct_gene − Ct_Actin2) and reported as mean ± SE across biological replicates, following MIQE recommendations (Bustin 2009; Schmittgen and Livak 2008). For RPG and bZIP, fold changes relative to buds were calculated using the 2^−ΔΔCt^ method (ΔΔCt = ΔCt_sample − ΔCt_bud) to enable cross‐organ comparison, and samples below the detection limit were reported as ND.

### 4.15 WGCNA

Transcriptome data derived from *A. glutinosa* roots, leaves, stems, apical buds and nodules were used for WGCNA. Gene expression levels were quantified as FPKM. Prior to network construction, PCA was conducted to assess sample clustering and to detect potential outliers. Based on the PCA results, one biological replicate from the stem samples was excluded from downstream analysis due to its deviation from the expected clustering pattern. The remaining expression data were used to construct a signed weighted co‐expression network using the WGCNA R package (Langfelder and Horvath 2008). The soft‐thresholding power was determined based on the scale‐free topology criterion to ensure an approximate scale‐free network structure. Gene modules were identified using the dynamic tree cut algorithm, and the association between each module and tissue type was calculated. Modules with an absolute correlation coefficient (|*r*|) greater than 0.7 and a *p* value less than 0.05 were considered significantly associated. To investigate the molecular basis of carbon allocation, we screened the nodule‐specific WGCNA module for genes involved in carbohydrate transport and metabolism using eggNOG‐mapper functional annotations. We identified a core set of 12 candidate genes (Table S26) and visualised their expression patterns across tissues using the pheatmap R package based on TMM‐normalised CPM values.

### 4.16 Phylogenetic, Microsynteny and cis‐Regulatory Analysis of bZIP TFs

The protein sequences of all *A. thaliana* bZIP TFs were downloaded from the iTAK database, and all bZIP TFs in the *A. glutinosa* genome were predicted using the iTAK pipeline (Y. Zheng 2016b). All *A. thaliana* bZIP sequences and the predicted *A. glutinosa* bZIP protein sequences were aligned using MAFFT for multiple sequence alignment. A maximum likelihood phylogenetic tree was then constructed with IQ‐TREE2. Microsynteny analysis was performed among *A. glutinosa*, *M. truncatula*, *P. andersonii* and *D. glomerata* to investigate local gene collinearity. Syntenic blocks were defined based on a minimum of four colinear genes, and visualised using the JCVI toolkit (Tang et al. 2024). For expression pattern analysis across roots and nodules in the four species, raw count expression data of *A. glutinosa* and the three other species were normalised using the median‐of‐ratios method implemented in the DESeq. 2 package, and visualised using the ggplot2 package in R. The position weight matrix of *A. thaliana* bZIP11 was retrieved from the JASPAR database (Sandelin 2004). FIMO was employed to scan the 2‐kb upstream promoter regions of genes within the NSM in *A. glutinosa* for potential motif occurrences (Grant et al. 2011).

## Conflicts of Interest

The authors declare no conflicts of interest.

## Supporting information


**Figure S1.** Whole‐genome alignments of *Alnus glutinosa* with *Betula pendula* and *Betula platyphylla*.
**Figure S2.** Time‐calibrated phylogeny reconstructed for 20 taxa.
**Figure S3.** Enrichment analysis of significantly expanded genes in *Alnus glutinosa*.
**Figure S4.** Differentially expressed genes in *Alnus glutinosa* between roots and nodules.
**Figure S5.** Enrichment analysis of nodule‐enhanced genes in *Alnus glutinosa*.
**Figure S6.** Conserved motif structure of nsHB1 in different plant species.
**Figure S7.** RNA‐seq expression profiles of RPG and its paralogs in *Alnus glutinosa*.
**Figure S8.** RNA‐seq expression profiles of AGO5 genes in *Alnus glutinosa*.
**Figure S9.** Expression profiles of AGO5 genes in different organs of *Alnus glutinosa*.
**Figure S10.** Weighted correlation network analysis (WGCNA) of *Alnus glutinosa* organ transcriptomes.
**Figure S11.** Gene expression in the nodule‐specific green module identified by WGCNA in *Alnus glutinosa*.
**Figure S12.** GO and KEGG enrichment analysis of the 231 genes in the nodule‐specific green module identified by WGCNA.
**Figure S13.** Assessment of potential technical variation in organ transcriptomes of *Alnus glutinosa*.
**Figure S14.** qRT–PCR validation of RPG and bZIP expression in different organs of *Alnus glutinosa*.
**Figure S15.** Distribution of gene concordance factors (gCF) across RNS‐related orthogroups.
**Figure S16.** Structural basis of *Alnus* nsHB1 adaptation inferred from modeling and in silico mutagenesis.
**Figure S17.** Cross‐study validation of nodule‐specific expression for representative RNS‐related genes *AgluHB1* and *AgluRPG*.
**Figure S18.** *Alnus*‐biased expansion of terpenoid‐related gene families relative to non‐nodulating Fagales controls.
**Figure S19.** Genome‐wide Hi‐C contact probability decay curve of the genome of *Alnus glutinosa*.
**Figure S20.** Coordinated expression of carbon metabolism and transport genes in *Alnus glutinosa*.
**Figure S21.** Overlap of nodule‐enhanced orthogroups across *Alnus*, legumes, and other actinorhizals.
**Figure S22.** Temporal expression profiles of *AGO5* family genes during *Alnus glutinosa* root nodule development.
**Figure S23.** Working model integrating the nodule‐specific module and key candidate components supporting root nodule symbiosis in *Alnus glutinosa*.

**Table S1.** Summary of sequenced data for *A. glutinosa* genome assembly.
**Table S2.** Statistics of the de novo genome assembly of *A. glutinosa*.
**Table S3.** Statistics of pseudo‐chromosome lengths of *A. glutinosa*.
**Table S4.** Evaluation of genome quality by BUSCO v5.5.0.
**Table S5.** The content of repeat sequence in *A. glutinosa* chromosome‐scale genome.
**Table S6.** Evaluation of genome annotation quality by BUSCO v5.5.0.
**Table S7.** Statistics of functional annotation of *A.glutinosa* genes using eggnog‐mapper.
**Table S8.** Intragenomic collinear blocks identified in *A. glutinosa* genome.
**Table S9.** *Alnus* and *Betula* Ks peak values.
**Table S10.** Information on the genomes used for comparative genomic analysis.
**Table S11.** A total of orthologous groups identified among 20 species by OrthoFinder.
**Table S12.** Significantly expanded gene families in *A. glutinosa*.
**Table S13.** Gene Ontology (GO) Enrichment analyses of significantly expanded genes in *A. glutinosa*.
**Table S14.** Kyoto Encyclopedia of Genes and Genomes (KEGG) Enrichment analyses of significantly expanded genes in *A. glutinosa*.
**Table S15.** Summary of mapping rates of mRNA‐seq data.
**Table S16.** Differentially Expressed Genes (DEGs) in Root vs Nodules in *A. glutinosa*.
**Table S17.** Gene Ontology (GO) Enrichment analyses of nodule‐enhanced genes in *A. glutinosa*.
**Table S18.** Kyoto Encyclopedia of Genes and Genomes (KEGG) Enrichment analyses of nodule‐enhanced genes in *A. glutinosa*.
**Table S19.** Hemoglobin sequences from selected species.
**Table S20.** Collected reference RNS‐related genes.
**Table S21.** *A. glutinosa* genes clustered within the same clade as reference RNS‐related genes.
**Table S22.** *A. glutinosa* genes and RNS‐related genes in the same clade.
**Table S23.** Primer sequences used for qPCR validation of AGO5 genes and the reference gene (Actin2) of *A. glutinosa*.
**Table S24.** Summary of qPCR‐based relative expression levels (2^‐ΔCt) of AGO5 paralogs across four organs of *A.glutinosa*.
Table S24 (continued). Additional qRT–PCR summary for *RPG* and *bZIP* (normalized to Bud within each gene; values shown as fold change vs Bud)
**Table S25.** *A. glutinosa* genes in the green module.
**Table S26.** Detailed annotation and expression profiles of core carbon metabolism genes identified in the nodule‐specific module.
**Table S27.** Potential binding sites of Arabidopsis bZIP11 protein within the 2000 bp upstream regions of genes in the green module of *A. glutinosa*.
**Table S28.** Consolidated summary of curated RNS‐related orthogroup phylogenies in Dataset S1.

**Dataset S1.** 275 phylogenetic trees of nitrogen‐fixing related genes from 20 species.

## Data Availability

The sequencing data presented in this study are accessible at the National Genomics Data Center (NGDC) under BioProject accession PRJCA040994. Comprehensive genome sequencing datasets, including PacBio long reads, Hi‐C reads, and RNA‐seq data, have been deposited in the Genome Sequence Archive (GSA) of NGDC under accession CRA026337. The assembled genome and annotation files are available on Figshare (https://doi.org/10.6084/m9.figshare.29232266).
