# Supplementary material for: Chromosome‐Level Genome and Organ‐Specific Transcriptome of Alnus glutinosa Uncover Lineage‐Specific Innovations in Root Nodule Symbiosis
Source: Plant Cell Environ. 2026 Feb 12;49(6):3003–21. doi: 10.1111/pce.70440 (PMC13136556; doi:10.1111/pce.70440)
Supplement: Supplementary file 1 — Figure S1. Whole‐genome alignments of Alnus glutinosa with Betula pendula and Betula platyphylla. Figure S2. Time‐calibrated phylogeny reconstructed for 20 taxa. Figure S3. Enrichment analysis of significantly expanded genes in Alnus glutinosa. Figure S4. Differentially expressed genes in Alnus glutinosa between roots and nodules. Figure S5. Enrichment analysis of nodule‐enhanced genes in Alnus glutinosa. Figure S6. Conserved motif structure of nsHB1 in different plant species. Figure S7. RNA‐seq expression profiles of RPG and its paralogs in Alnus glutinosa. Figure S8. RNA‐seq expression profiles of AGO5 genes in Alnus glutinosa. Figure S9. Expression profiles of AGO5 genes in different organs of Alnus glutinosa. Figure S10. Weighted correlation network analysis (WGCNA) of Alnus glutinosa organ transcriptomes. Figure S11. Gene expression in the nodule‐specific green module identified by WGCNA in Alnus glutinosa. Figure S12. GO and KEGG enrichment analysis of the 231 genes in the nodule‐specific green module identified by WGCNA. Figure S13. Assessment of potential technical variation in organ transcriptomes of Alnus glutinosa. Figure S14. qRT–PCR validation of RPG and bZIP expression in different organs of Alnus glutinosa. Figure S15. Distribution of gene concordance factors (gCF) across RNS‐related orthogroups. Figure S16. Structural basis of Alnus nsHB1 adaptation inferred from modeling and in silico mutagenesis. Figure S17. Cross‐study validation of nodule‐specific expression for representative RNS‐related genes AgluHB1 and AgluRPG. Figure S18. Alnus‐biased expansion of terpenoid‐related gene families relative to non‐nodulating Fagales controls. Figure S19. Genome‐wide Hi‐C contact probability decay curve of the genome of Alnus glutinosa. Figure S20. Coordinated expression of carbon metabolism and transport genes in Alnus glutinosa. Figure S21. Overlap of nodule‐enhanced orthogroups across Alnus, legumes, and other actinorhizals. Figure S22. Temporal expression p [file PCE-49-3003-s003.docx]

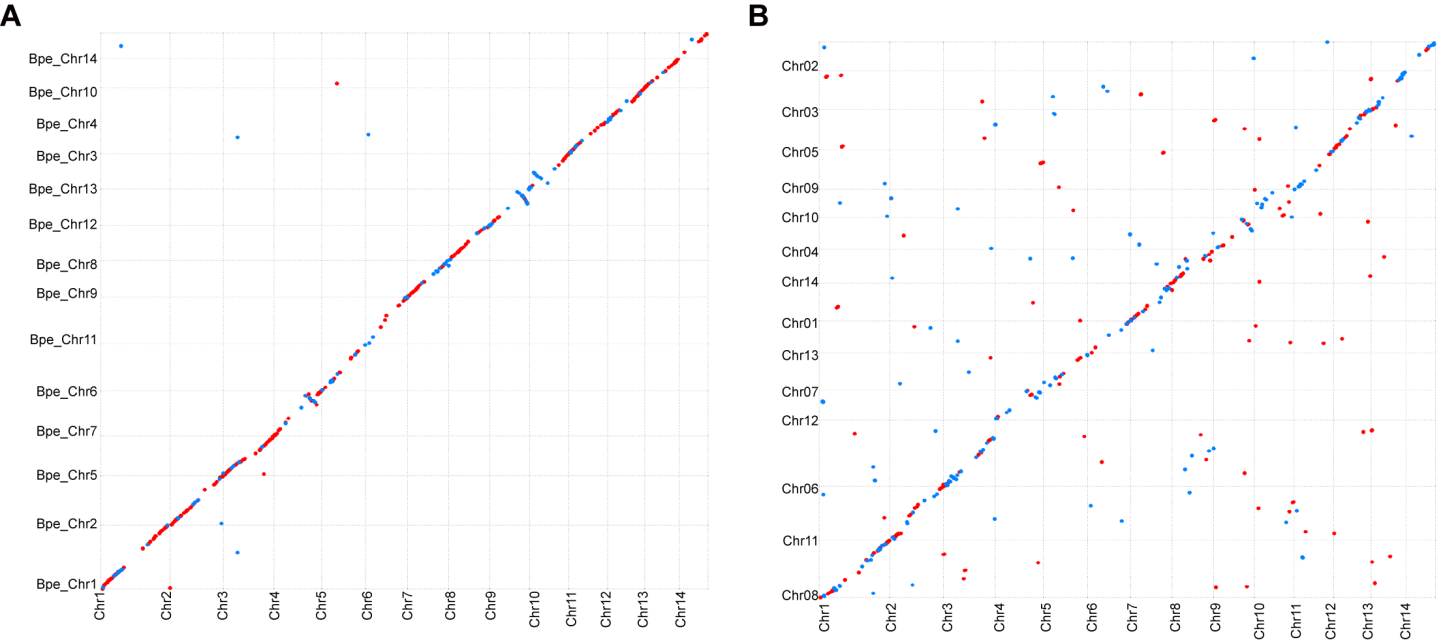


**Figure S1. Whole-genome alignments of *Alnus glutinosa* with *Betula pendula* and *Betula platyphylla*.**

(A) Whole-genome alignment between *A. glutinosa* and *B. pendula*. The x-axis represents the chromosome sequences of *A. glutinosa*, while the y-axis represents the chromosome sequences of *B. pendula.* Red dots indicate that the sequences of the two species at this site are in the same direction, while blue indicates that the sequences of the species are in the opposite direction.

(B) Whole-genome alignment between *A. glutinosa* and *B. platyphylla*. The x-axis represents the chromosome sequences of *A. glutinosa*, and the y-axis represents the chromosome sequences of *B. platyphylla*. Red dots indicate that the sequences of the two species at this site are in the same direction, while blue indicates that the sequences of the species are in the opposite direction.

**
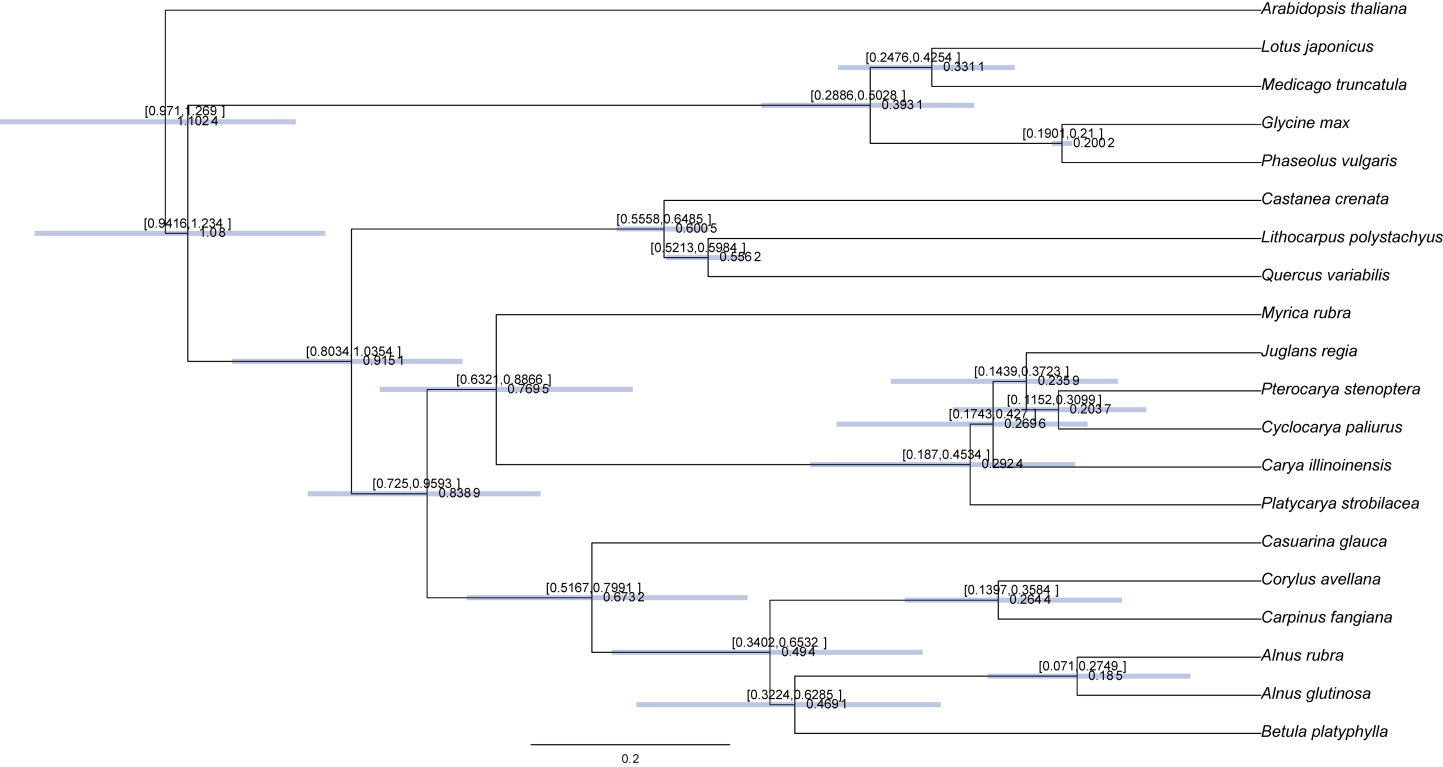
**

**Figure S2. Time-calibrated phylogeny reconstructed for 20 taxa.**

Divergence time estimates (in billion years) are shown at nodes, with bracketed values indicating 95% highest posterior density (HPD) confidence intervals. Corresponding purple bars graphically depict the temporal ranges of 95% HPD intervals for each node.

**
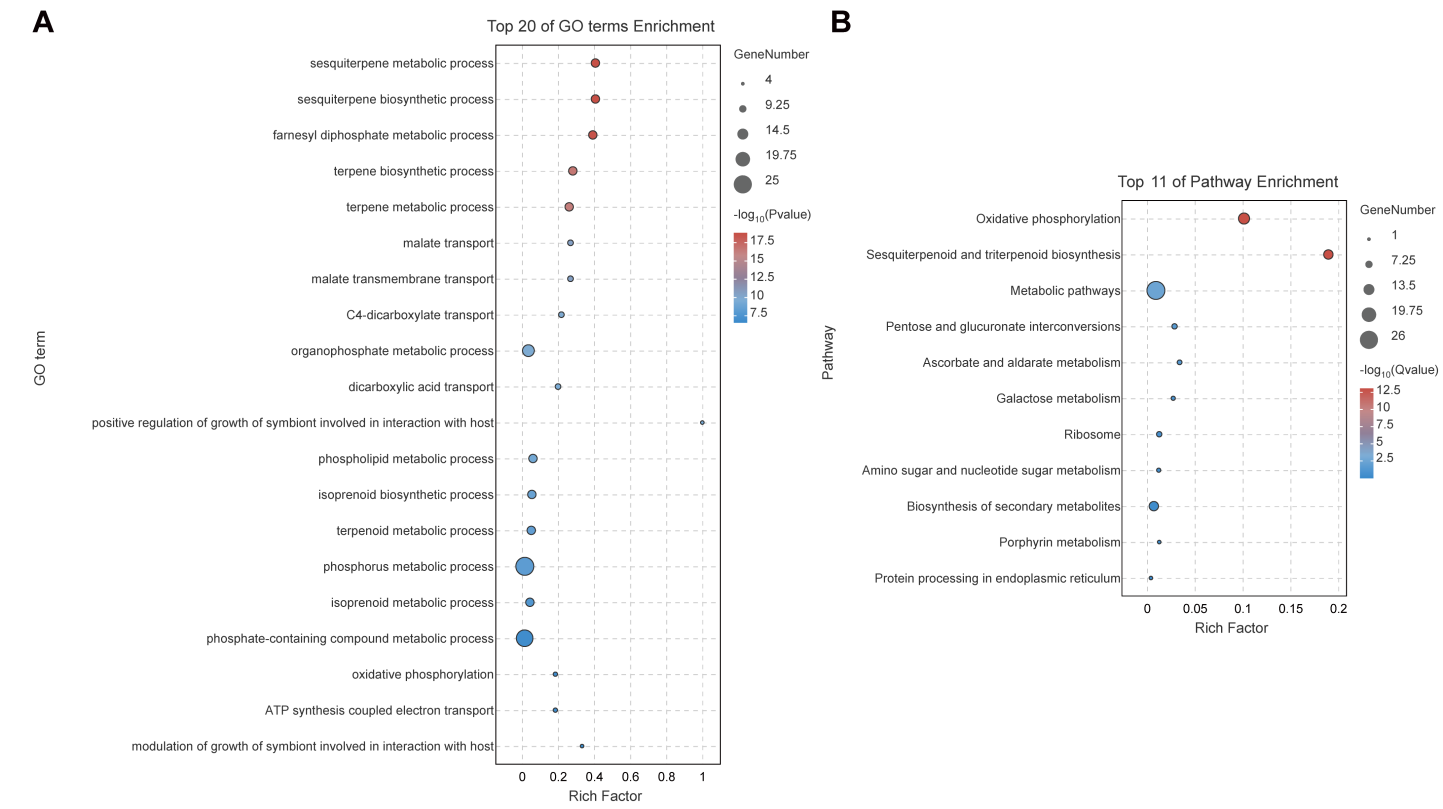
**

**Figure S3. Enrichment analysis of significantly expanded genes in *Alnus glutinosa*.**

(A) GO enrichment analysis of significantly expanded genes in *A. glutinosa*. The top 20 enriched GO terms in the biological process (BP) category are shown.

(B) KEGG pathway enrichment analysis of significantly expanded genes in *A. glutinosa*. The top 11 enriched pathways are shown.

**
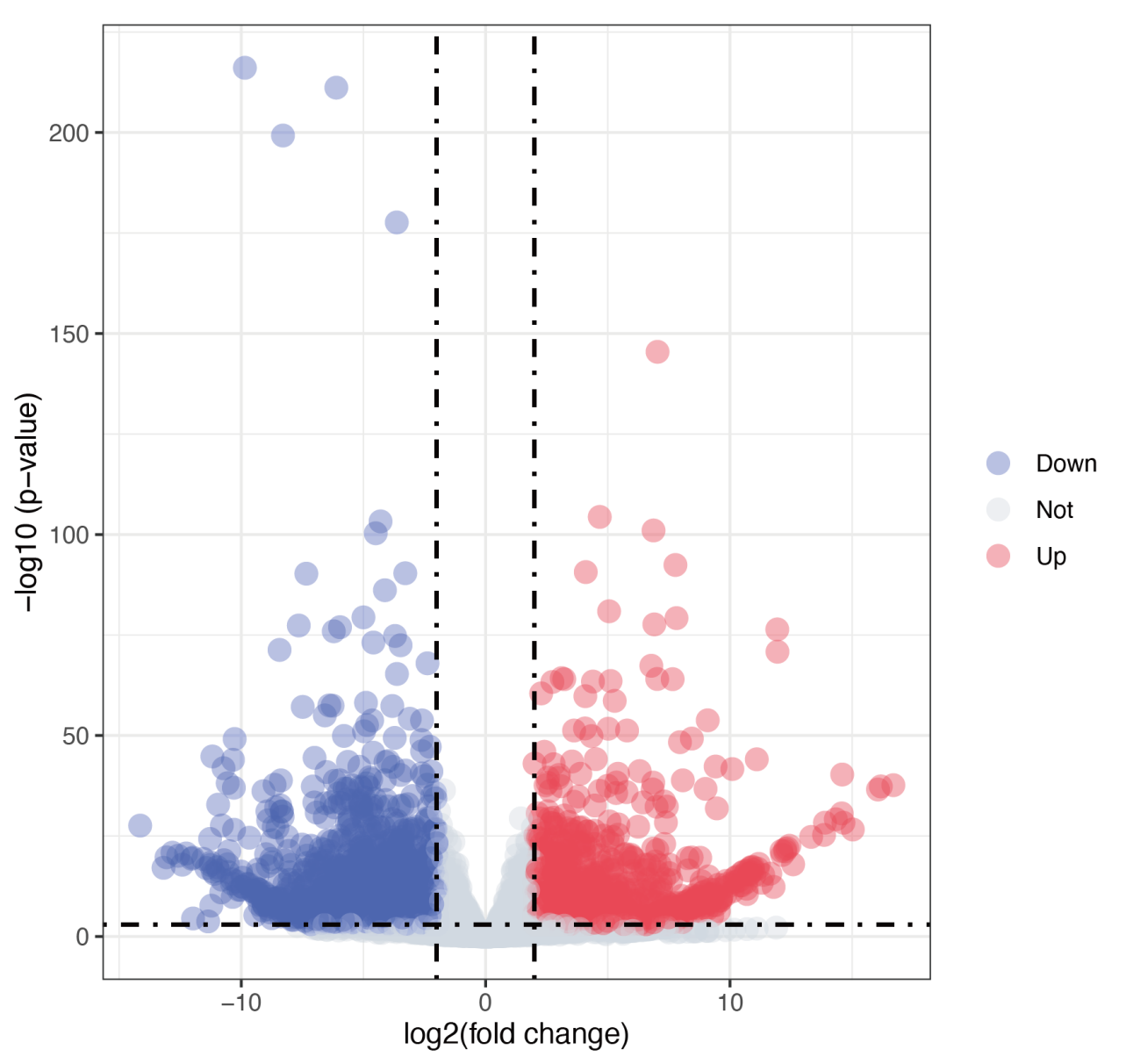
**

**Figure S4. Differentially expressed genes in *Alnus glutinosa* between roots and nodules.**

Blue represents genes enhanced in nodules, red represents genes enhanced in roots, and gray represents genes with no differential expression.

**
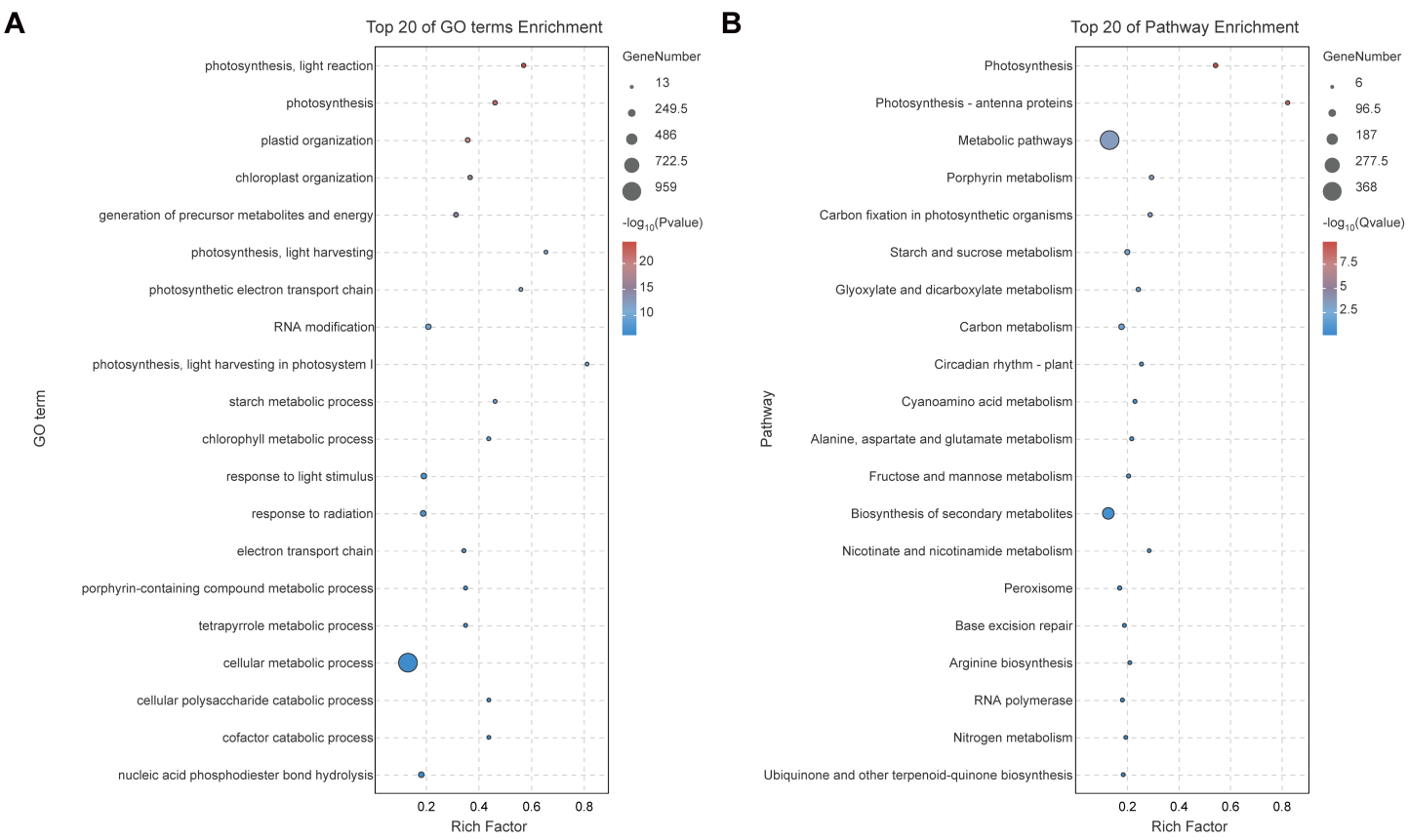
**

**Figure S5. Enrichment analysis of nodule-enhanced genes in *Alnus glutinosa*.**

(A) GO enrichment analysis of nodule-enhanced genes in *A. glutinosa*. The top 20 enriched GO terms in the biological process (BP) category are shown.

(B) KEGG pathway enrichment analysis of nodule-enhanced genes in *A. glutinosa*. The top 20 enriched pathways are shown.

**
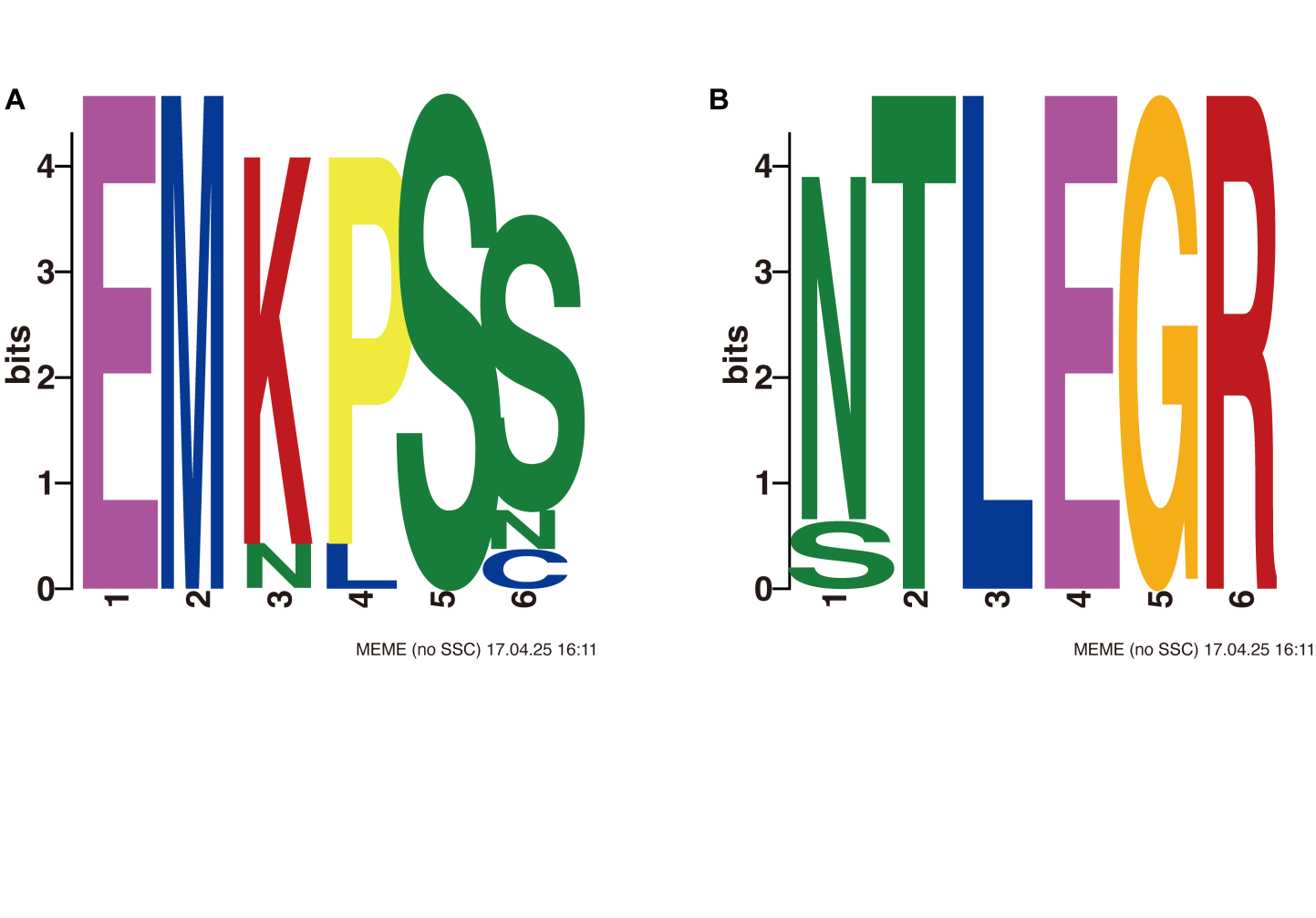
**

**Figure S6. Conserved motif structure of nsHB1 in different plant species.**

(A) Conserved motif structure of nsHB1 in the nitrogen-fixing clade. The logo represents the sequence motifs conserved across different nodulators, with the height of the letters indicating the degree of conservation at each position.

(B) Conserved motif structure of nsHB1 in the Fagales order. This logo illustrates the motif pattern conserved across species within the Fagales order, with the size of each letter reflecting its significance in the motif.

**
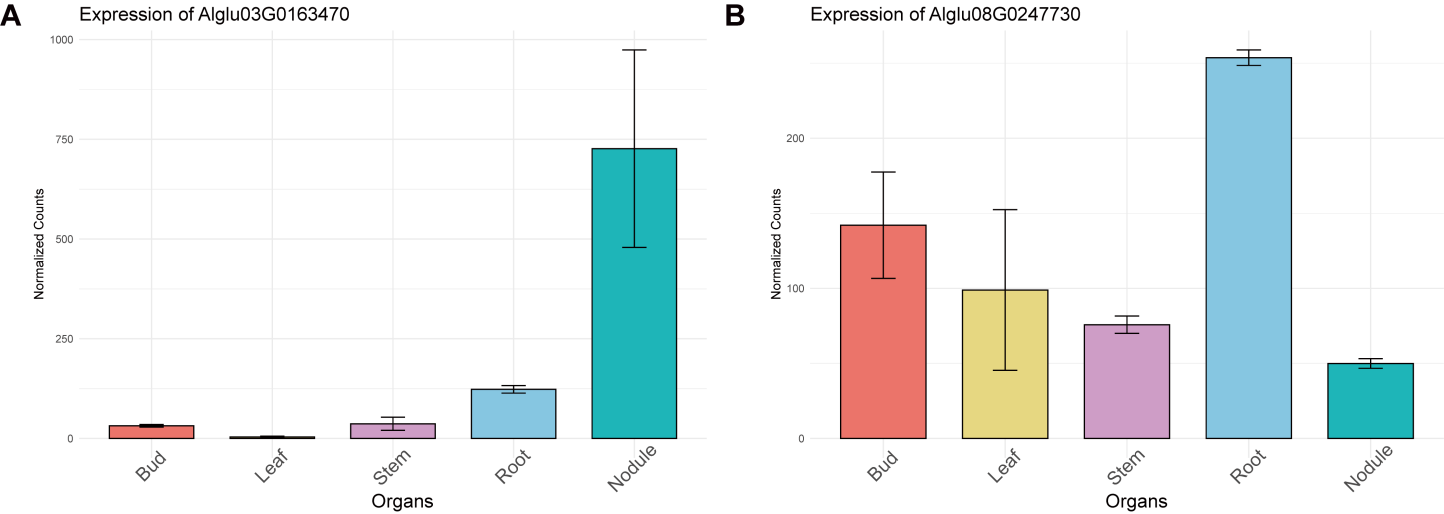
**

**Figure S7. RNA-seq expression profiles of *RPG* and its paralogs in *Alnus glutinosa*.**

(A) Expression of *RPG* gene across different organs in *A. glutinosa*. The expression levels are presented as normalized counts, standardized using DESeq2.

(B) Expression of the paralogous gene of *RPG* across different organs in *A. glutinosa*. The expression levels are presented as normalized counts, standardized using DESeq2.

**
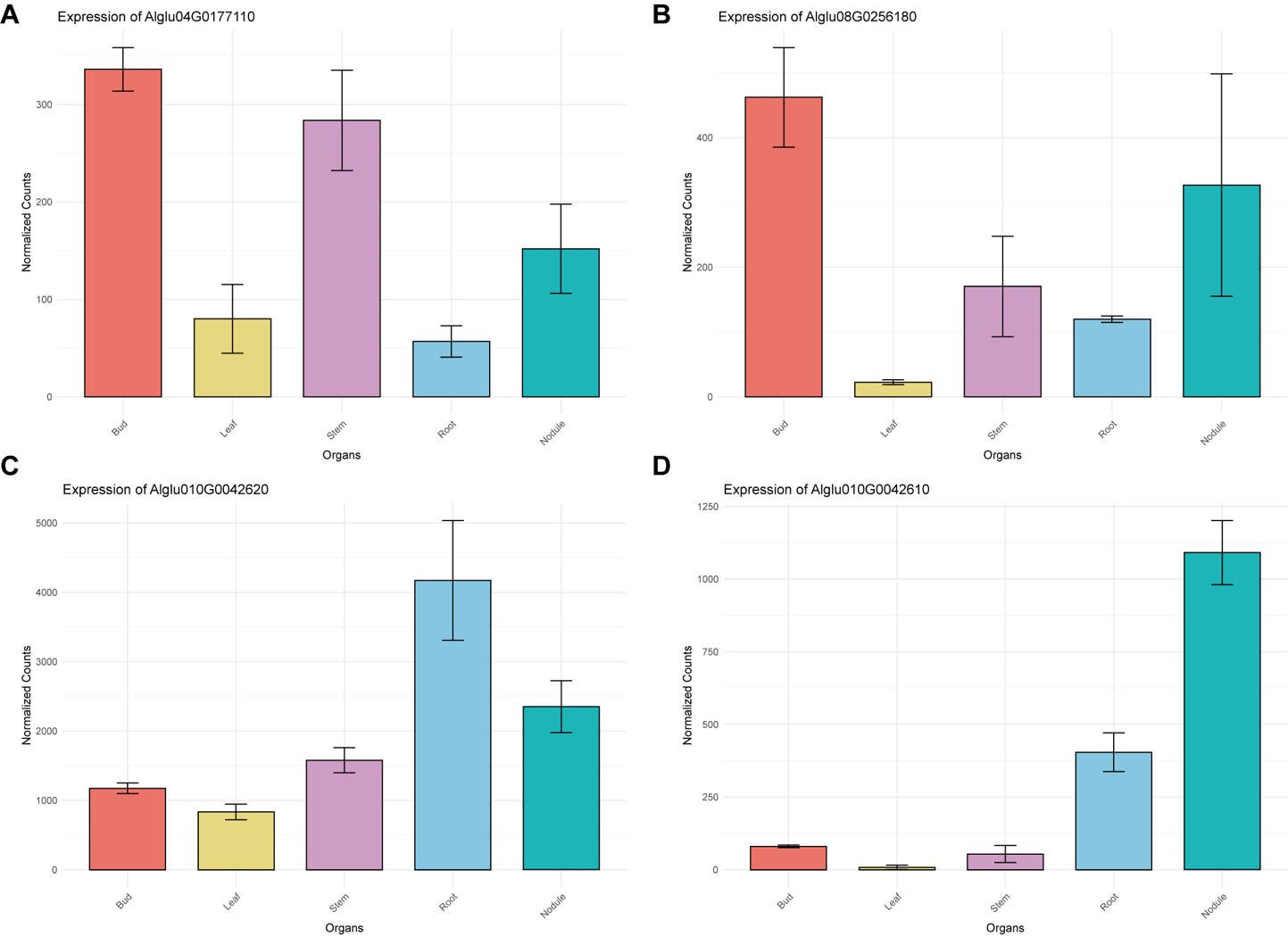
**

**Figure S8. RNA-seq expression profiles of *AGO5* genes in *Alnus glutinosa*.**

(A–D) Organ-specific RNA-seq expression levels of *AGO5a* (Alglu04G0177110), *AGO5b* (Alglu08G0256180), *AGO5c* (Alglu010G0042620), and *AGO5d* (Alglu010G0042610) across bud, leaf, stem, root, and nodule. Expression values are shown as normalized read counts using DESeq2 from three biological replicates per organ.


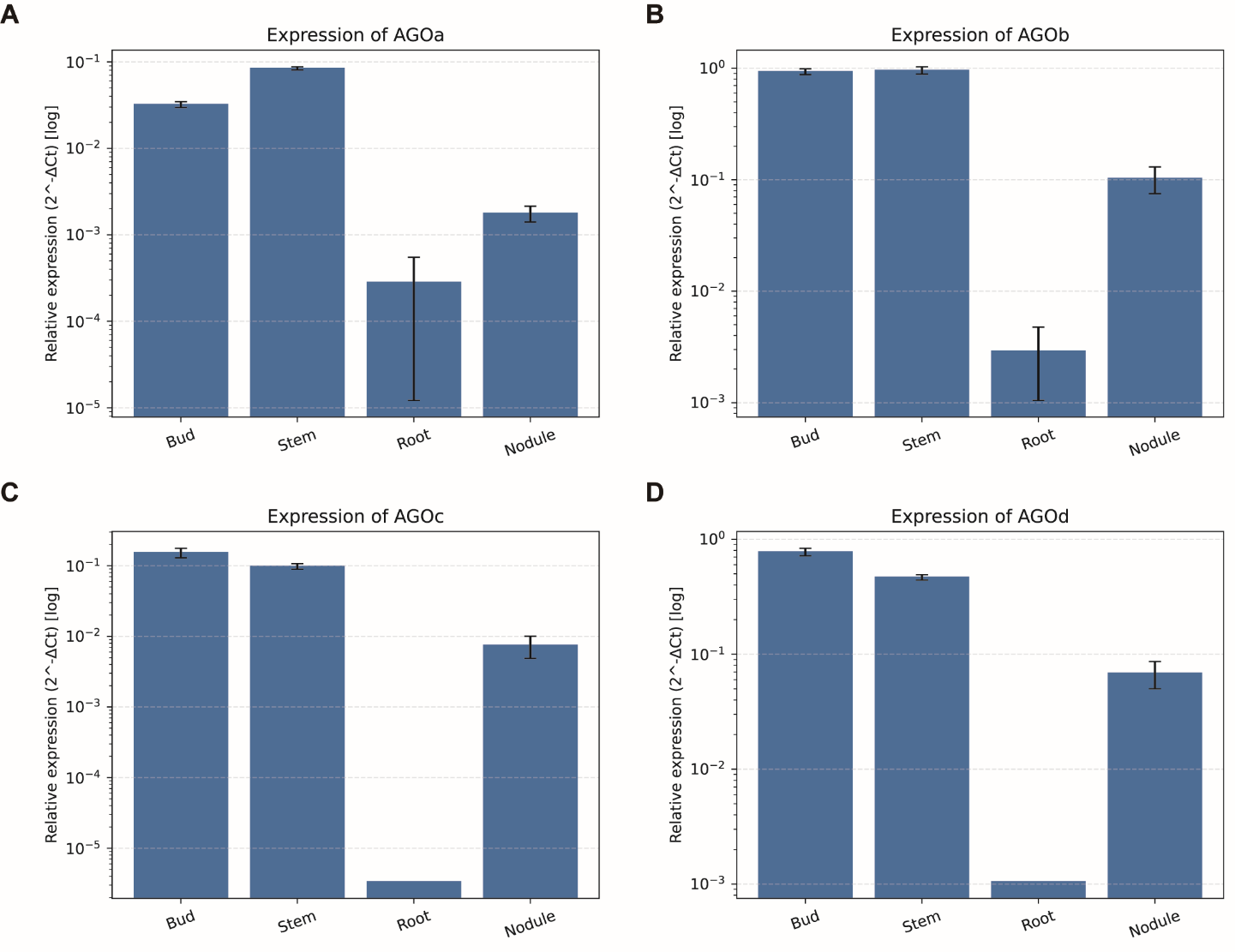


**Figure S9. Expression profiles of *AGO5* genes in different organs of *Alnus glutinosa.***

(A–D) Quantitative RT–PCR analysis of *AGO5a* (Alglu04G0177110), *AGO5b* (Alglu08G0256180), *AGO5c* (Alglu010G0042620), and *AGO5d* (Alglu010G0042610) in bud, stem, root, and nodule. Expression levels are presented as 2^-ΔCt values relative to the internal reference gene *Actin2* and plotted on a logarithmic scale. Bars indicate mean ± SE of three biological replicates (two replicates for roots in *AGO5a/b*).

**
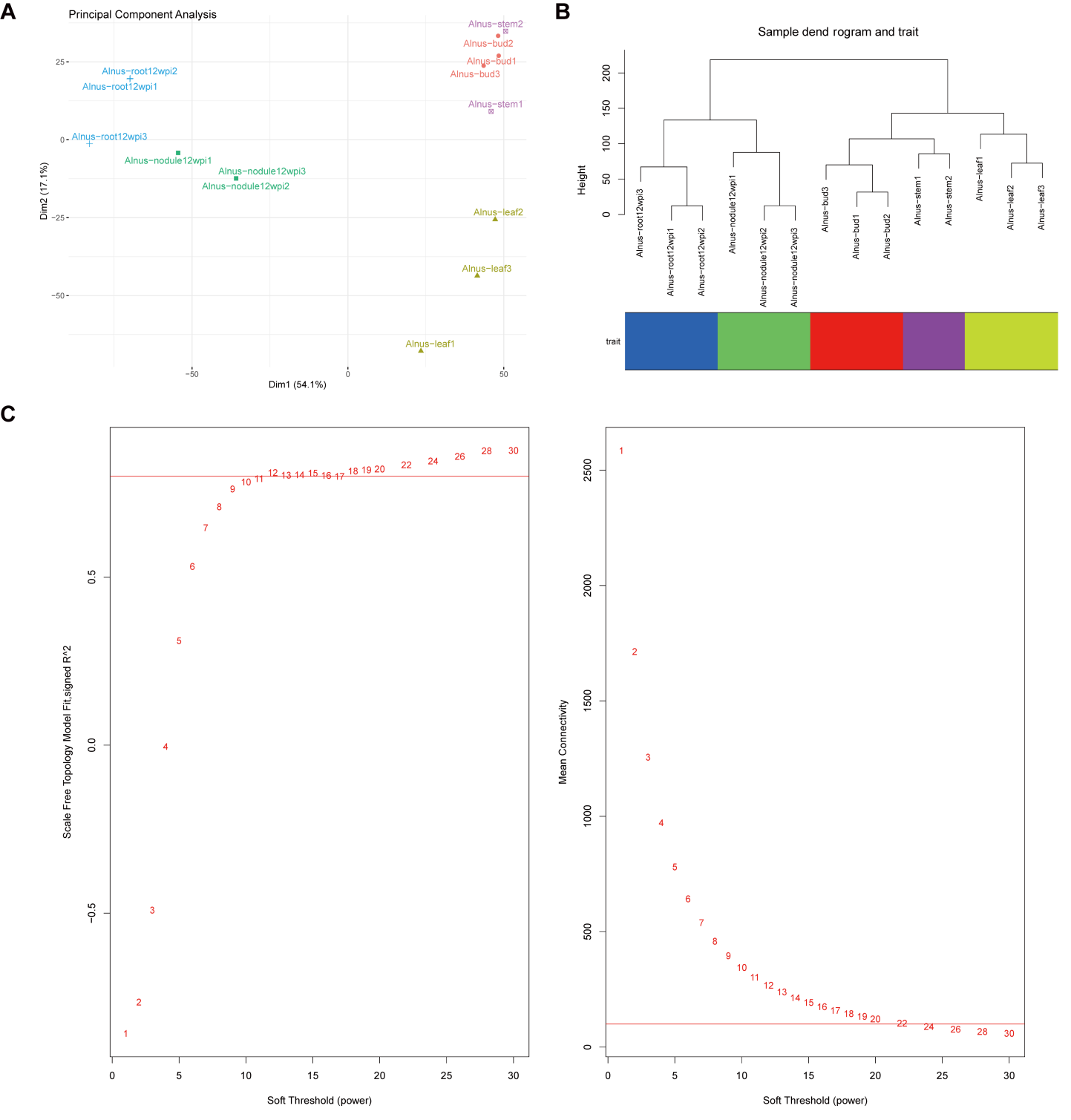
**

**Figure S10. Weighted correlation network analysis (WGCNA) of *Alnus glutinosa* organ transcriptomes.**

(A) Principal component analysis (PCA) of transcriptome samples from five organs of *A. glutinosa* after removing one stem outlier sample in the WGCNA analysis.

(B) Sample dendrogram and trait association. The hierarchical clustering dendrogram represents the relationships between the samples of each organ, with each sample color-coded according to the organ it originates from.

(C) Soft threshold analysis for network construction in WGCNA. Panel (C) displays the scale-free topology model fit (left) and the mean connectivity (right) as a function of the soft thresholding power.

**
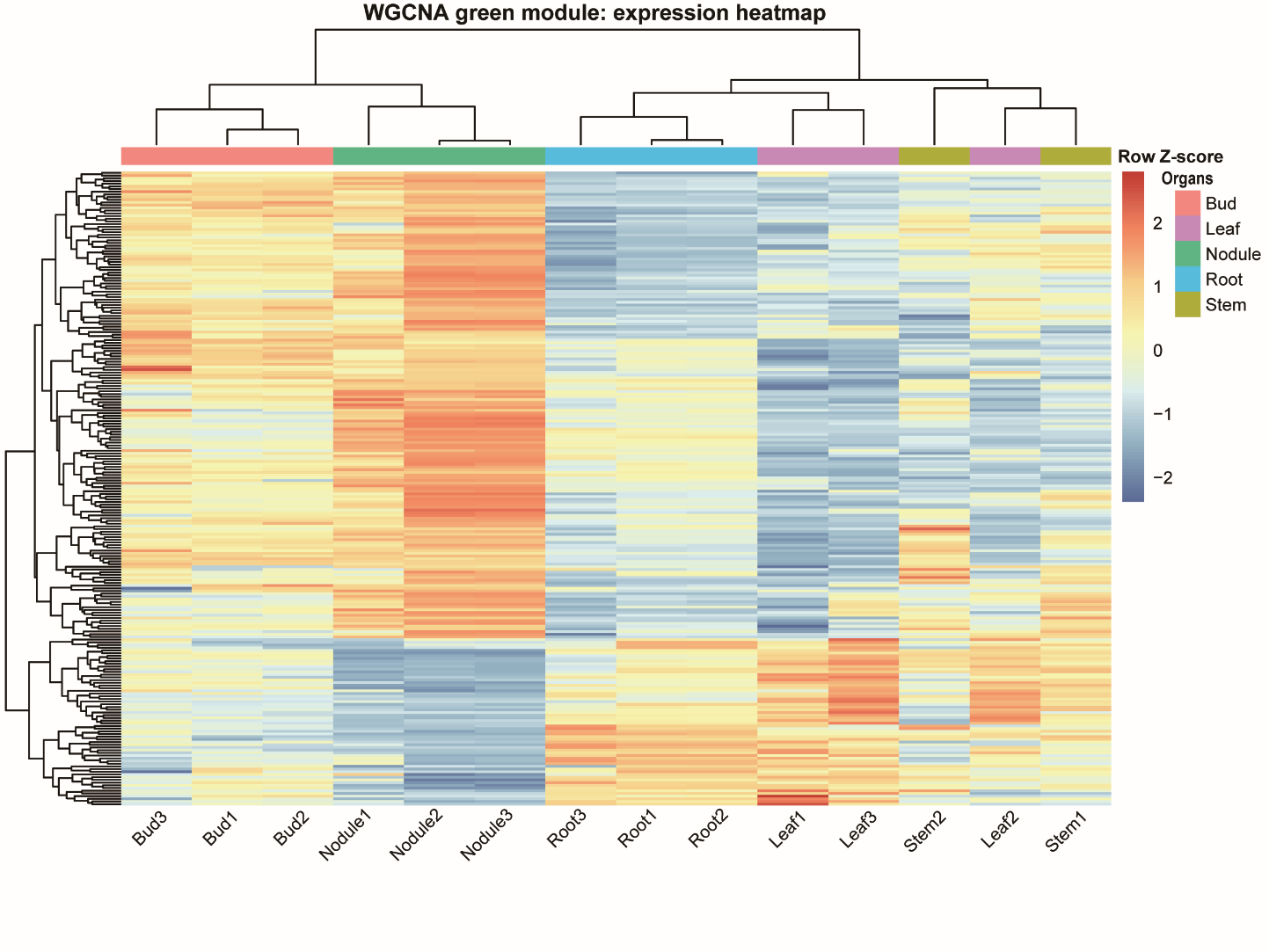
**

**Figure S11. Gene expression in the nodule-specific green module identified by WGCNA in *Alnus glutinosa*.**

Columns are RNA-seq libraries (3 biological replicates per organ: Bud1–3, Leaf1–3, Stem1–3, Root1–3, and Nodule1–3). Each row represents one gene belonging to the “green” co-expression module. Values are row-wise Z-scores of log2(FPKM+1); blue/red indicate lower/higher expression relative to each gene’s mean. Both genes and samples were hierarchically clustered (Pearson correlation distance, complete linkage). The top color bar annotates the organ of each library.


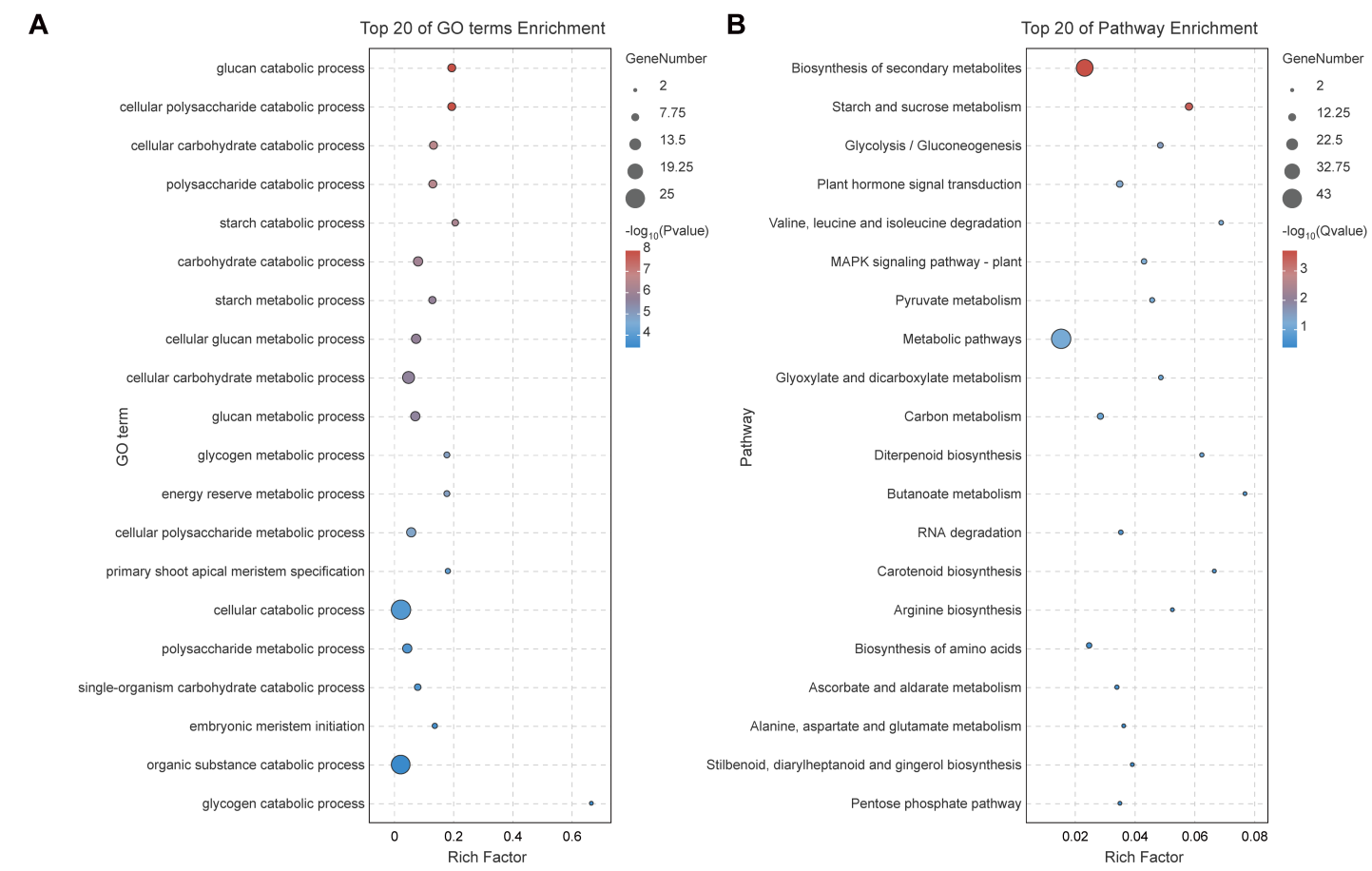


**Figure S12. GO and KEGG enrichment analysis of the 231 genes in the nodule-specific green module identified by WGCNA.**

(A) GO enrichment analysis of the 231 genes in the nodule-specific green module identified by WGCNA. The top 20 enriched GO terms are displayed.

(B) KEGG pathway enrichment analysis of the 231 genes in the nodule-specific green module identified by WGCNA. The top 20 enriched pathways are displayed.

**Figure S13. Assessment of potential technical variation in organ transcriptomes of *Alnus glutinosa*.**

(A) Principal component analysis (PCA) of variance-stabilized expression values (VST; DESeq2) across five organs (bud, leaf, stem, root, and nodule; three biological replicates per organ). PC1 and PC2 explain 79% and 9% of the variance, respectively.

(B) Sample-to-sample distance heatmap computed from VST-transformed counts, with hierarchical clustering of libraries, showing clustering primarily by organ and no obvious outliers.

**Figure S14. qRT–PCR validation of RPG and bZIP expression in different organs of *Alnus glutinosa*.**

(A, B) Relative transcript levels of RPG (A) and AglubZIP-TF1 (B) in bud, stem, root and nodule measured by qRT–PCR. Expression is shown as fold change relative to buds (2^−ΔΔCt) using Actin2 as the reference gene. Bars indicate mean ± SE of biological replicates (n=3), except for RPG in roots where only one biological replicate yielded a valid amplification (n=1) and therefore no SE is shown. For bZIP in roots, transcripts were below the detection limit (ND).

**Figure S15. Distribution of gene concordance factors (gCF) across RNS-related orthogroups.**

(A) Boxplot summarizing gCF values (in %) across internal branches of the species tree computed from the 361 curated RNS-related orthogroup gene trees.

(B) Density plot showing the overall distribution of gCF values across branches.

**Figure S16. Structural basis of *Alnus* nsHB1 adaptation inferred from modeling and in silico mutagenesis.**

(A) The Fagales-specific N-terminal motif (Motif 6; purple sticks) forms an exposed extension projecting from the globular core.

(B) The native Ile site forms a local hydrophobic surface patch (orange).

(C) A virtual substitution to a polar residue introduces a disruptive polar patch (cyan).

**Figure S17. Cross-study validation of nodule-specific expression for representative RNS-related genes *AgluHB1* and *AgluRPG*.**

(A) Transcript abundance (TPM, mean ± SD) of *AgluHB1* across tissue types.

(B) Transcript abundance (TPM, mean ± SD) of *AgluRPG* across tissue types.

Uninoculated roots were obtained from a public dataset (negative control), whereas inoculated roots and nodules were generated in this study.

**Figure S18. *Alnus*-biased expansion of terpenoid-related gene families relative to non-nodulating Fagales controls.**

Gene copy numbers are shown for representative orthologous groups (OGs) related to terpenoid biosynthesis and modification across *Alnus glutinosa*, *Betula platyphylla*, and *Quercus variabilis*. Copy number was defined as the number of genes assigned to each OG in each species.


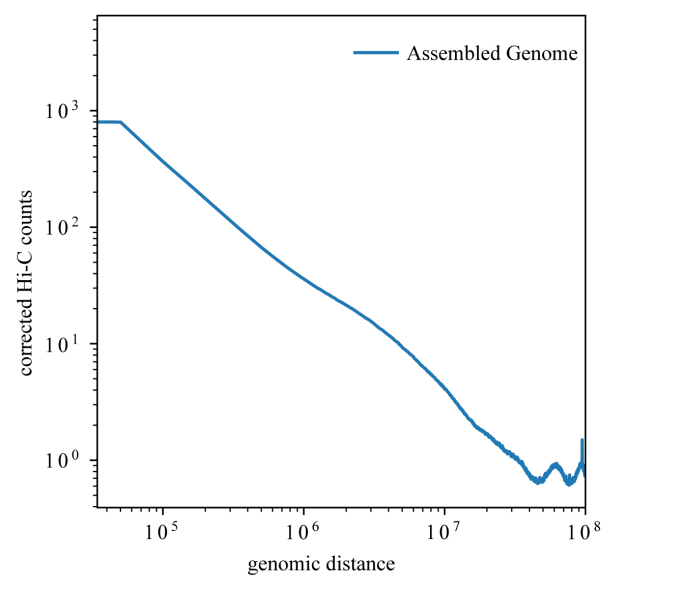


**Figure S19. Genome-wide Hi-C contact probability decay curve of the genome of *Alnus glutinosa*.**

Normalized Hi-C contact counts are plotted against genomic distance on a double-logarithmic scale at 50 kb resolution. The monotonic decrease in interaction frequency with increasing distance supports the structural integrity and accurate ordering of the chromosome-level assembly.

**Figure S20. Coordinated expression of carbon metabolism and transport genes in *Alnus glutinosa*.**

Heatmap showing the expression (Z-score normalized CPM) of 12 core genes identified within the nodule-specific WGCNA module. The panel includes genes for sugar transport (*MFS*), sucrose cleavage (*SUS4*), glycolysis (*PFK, PFP*), starch metabolism (*AMY5, ISA3, PHO1/2, DPE2, PWD1*), and molybdate transport.

**Figure S21. Overlap of nodule-enhanced orthogroups across *Alnus*, legumes, and other actinorhizals.**

Venn diagram showing the overlap of nodule-enhanced orthogroups among *Alnus* (*Alnus glutinosa*), legumes (*Medicago truncatula* and *Lotus japonicus*), and other actinorhizals (*Datisca glomerata* and *Hippophae rhamnoides*). Nodule-enhanced genes were identified within each species (nodule versus root) and mapped to OrthoFinder orthogroups for cross-lineage comparison. Numbers denote orthogroup counts in each region: *Alnus*-specific (1,223), legumes-specific (3,030), other actinorhizals-specific (1,832), pairwise overlaps (*Alnus*–legumes: 485; *Alnus*–actinorhizals: 447; legumes–actinorhizals: 949), and the shared core across all three lineages (452).

**Figure S22. Temporal expression profiles of *AGO5* family genes during *Alnus glutinosa* root nodule development.**

Heatmap showing Z-score normalized expression (CPM) of *AGO5* paralogs across developmental stages. Early-stage data (0 and 22 dpi) were retrieved from Zhang et al. (2024), and mature-stage data (12 wpi) were generated in this study. dpi**:** days post inoculation; wpi**:** weeks post inoculation.

**Figure S23. Working model integrating the nodule-specific module and key candidate components supporting root nodule symbiosis in *Alnus glutinosa*.**

Nodule-specific module is enriched for two transcription factors (bZIP and GLK) and coordinated carbon metabolism genes (MFS, SUS4, PFK/PFP, AMY5/ISA3/PHO1/2/DPE2/PWD1) and a molybdate transporter. nsHB1 (recruitment), RPG (PAV/retained), and AGO5 (CNV expansion) are integrated as additional candidate components supporting root nodule symbiosis in *Alnus glutinosa*.
