# Supplementary material for: Chromosome‐Level Genome and Organ‐Specific Transcriptome of Alnus glutinosa Uncover Lineage‐Specific Innovations in Root Nodule Symbiosis
Source: Plant Cell Environ. 2026 Feb 12;49(6):3003–21. doi: 10.1111/pce.70440 (PMC13136556; doi:10.1111/pce.70440)

Dateset S1 . 275 phylogenetic trees of nitrogen-fixing related genes from 20 species.

A gene tree constructed using 361 nitrogen-fixing related reference genes from four legume model species in 20 species shown in Figure 2D. At the top of the gene tree is the orthogroup to which the used genes belong, along with the integration of the gene names of the reference genes, which characterizes this tree. Different colors represent different species, with the reference genes shown in bold, and the nodule differentially expressed genes of *Alnus glutinosa* also shown in bold. Colors map to species as follows: red – *Alnus glutinosa*; dark brown – *Alnus rubra*; bright blue – *Betula platyphylla*; violet – *Betula pendula*; salmon – *Carpinus fangiana*; peach – *Corylus avellana*; teal – *Casuarina glauca*; olive green – *Cyclocarya paliurus*; light orange – *Pterocarya stenoptera*; beige –*Juglans regia*; dark khaki – *Carya illinoinensis*; pale green – *Platycarya strobilacea*; brick red – *Myrica rubra*; slate blue – *Lithocarpus polystachyus*; aqua – *Quercus variabilis*; sand brown – *Castanea crenata*; magenta – *Medicago truncatula*; bright green – *Lotus japonicus*; navy – *Phaseolus vulgaris*; goldenrod – *Glycine max*; black – *Arabidopsis thaliana*.

OG0000006:symbiotic CYSTEINE RICH KINASE

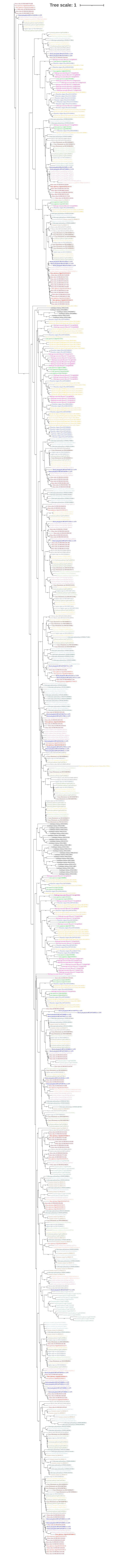

OG0000009:Nodule Number Locus 1/RESISTANCE GENE ANALOGUE

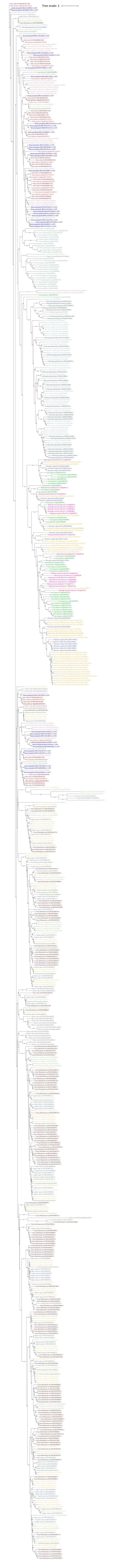

OG0000027 FERRITINS1

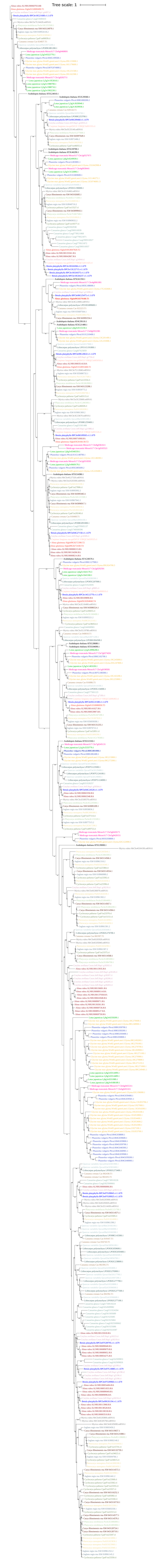

OG0000057:CYCLIC NUCLEOTIDE GATED CHANNELS 15A/15B/15C

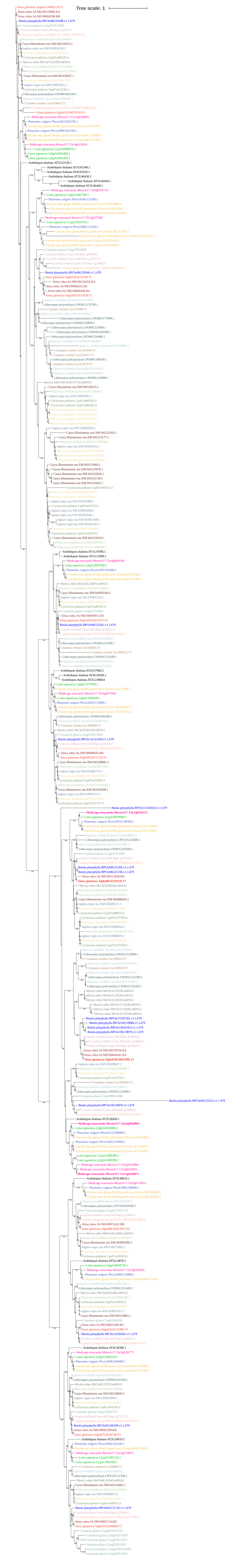

OG0000064/PHOSPHATE TRANSPORTER 5/1

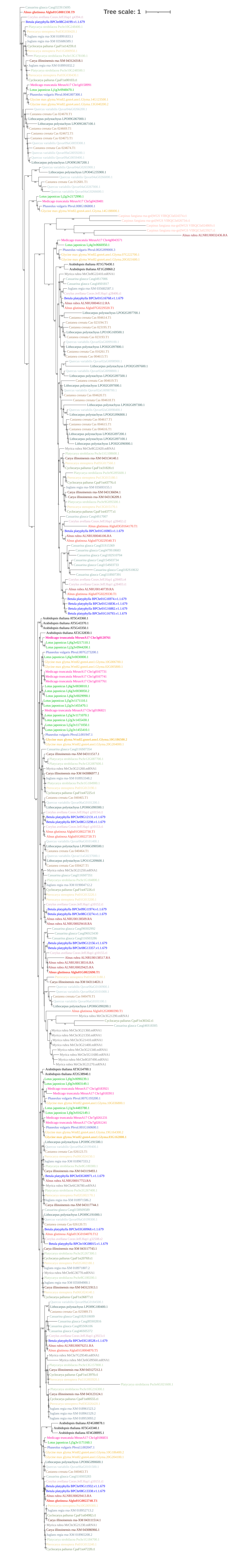

OG0000090 CYSTEINE PROTEINASE 6

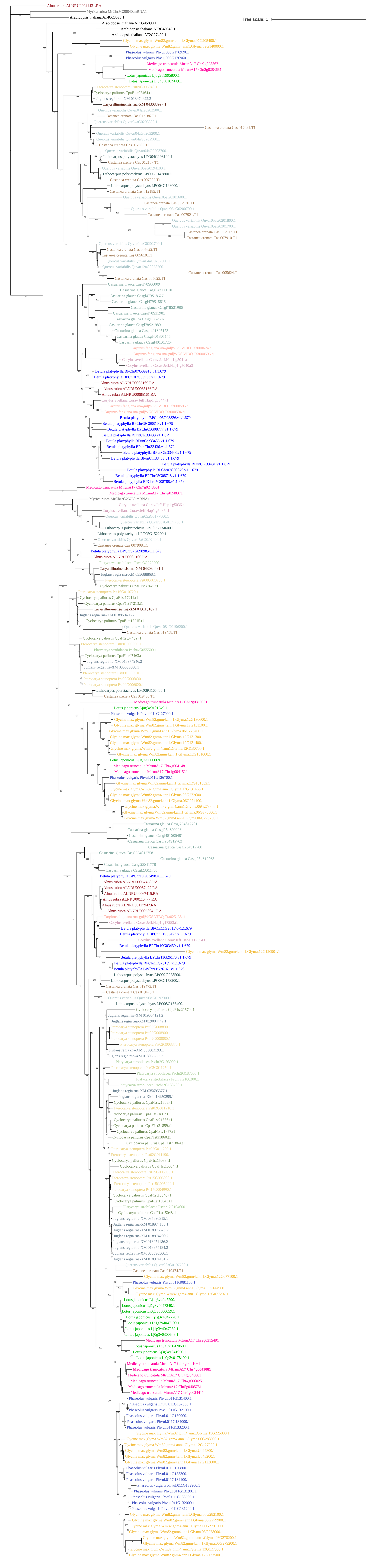

OG0000095:Nodule-specific Cysteine-Rich peptide

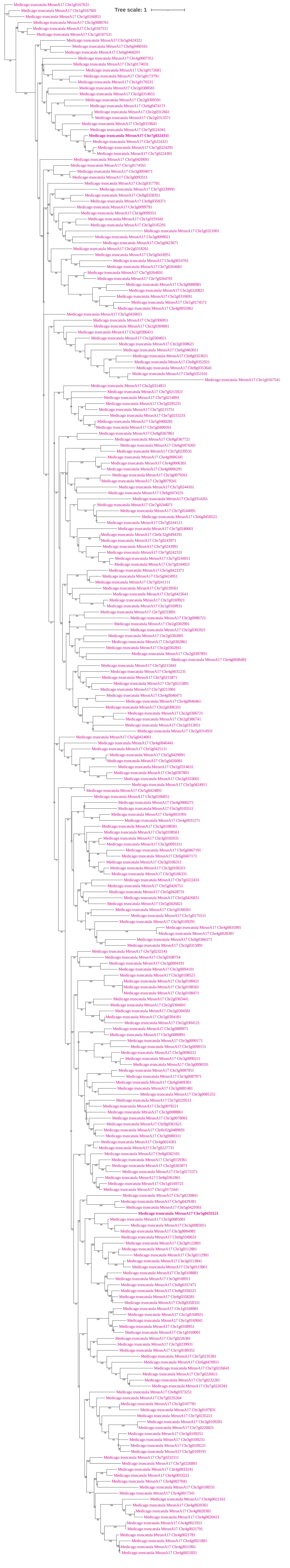

OG0000168:Myeloblastosis(MYB) transcription factor 11

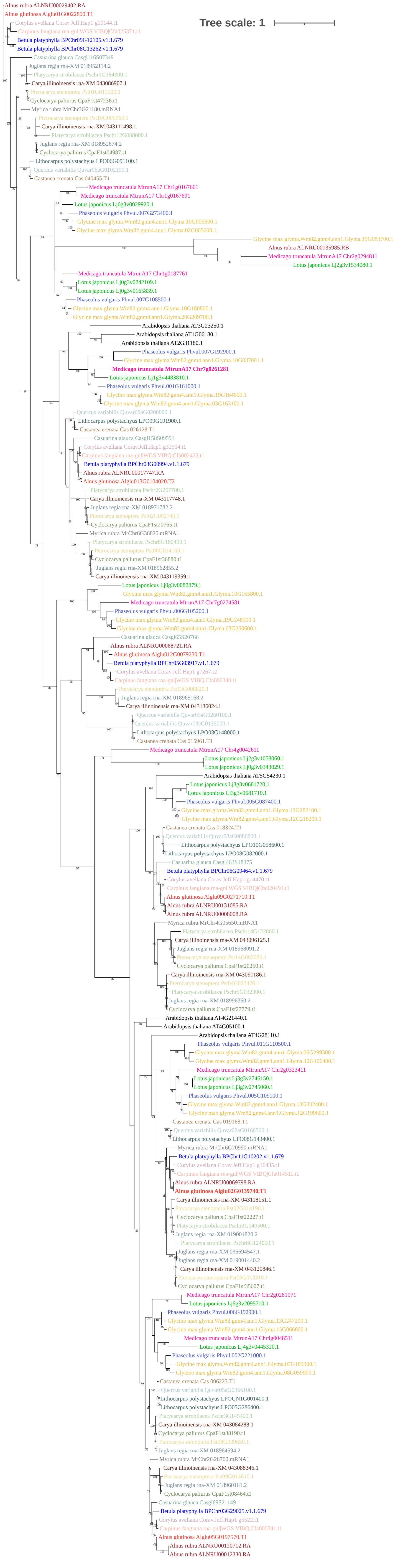

OG0000180:Rho family of small GTPases in plants

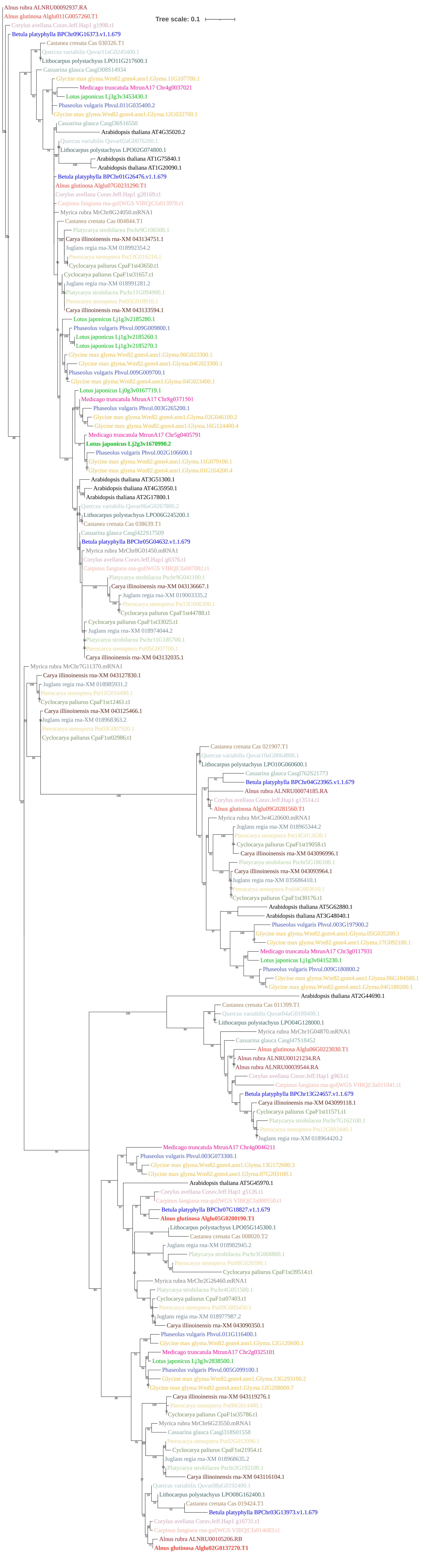

OG0000188:Rotundifolia/Devil

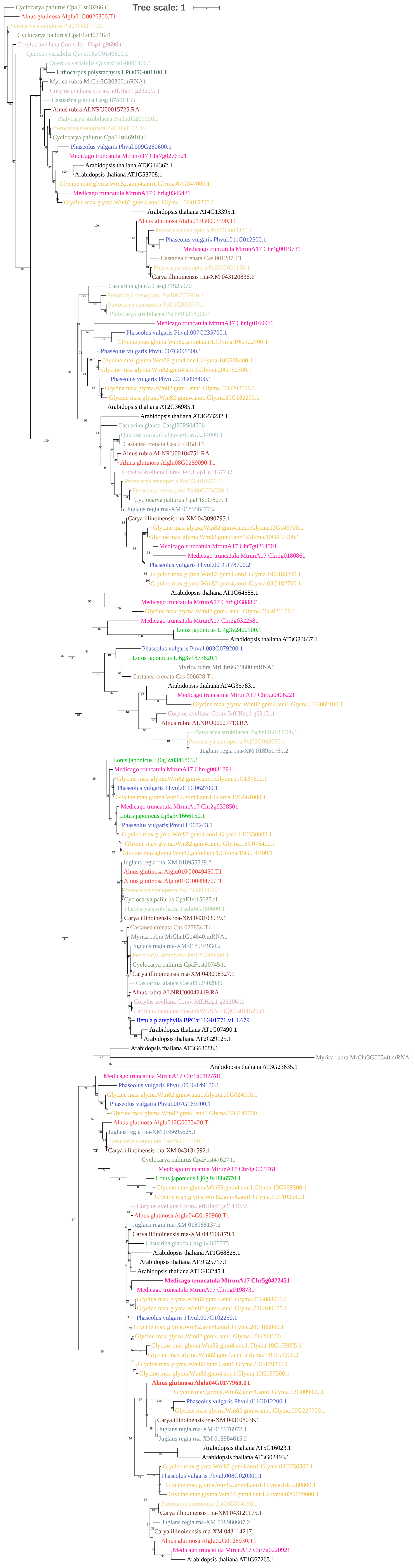

OG0000193:ATP-binding cassette(ABC) transporter type G

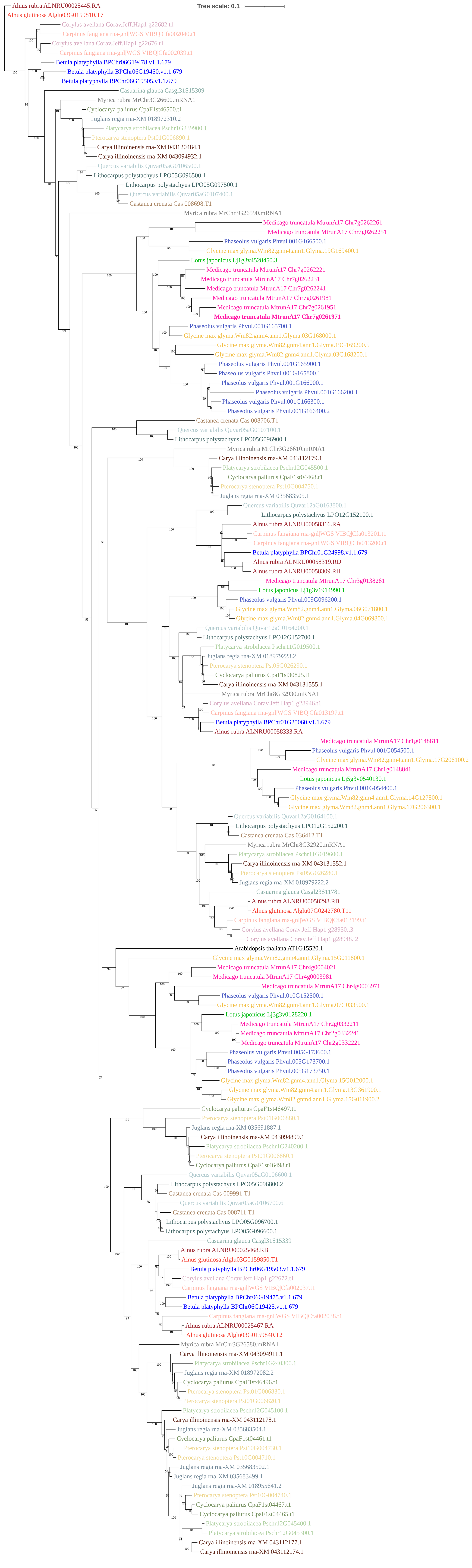

OG0000249:LONELYGUY1

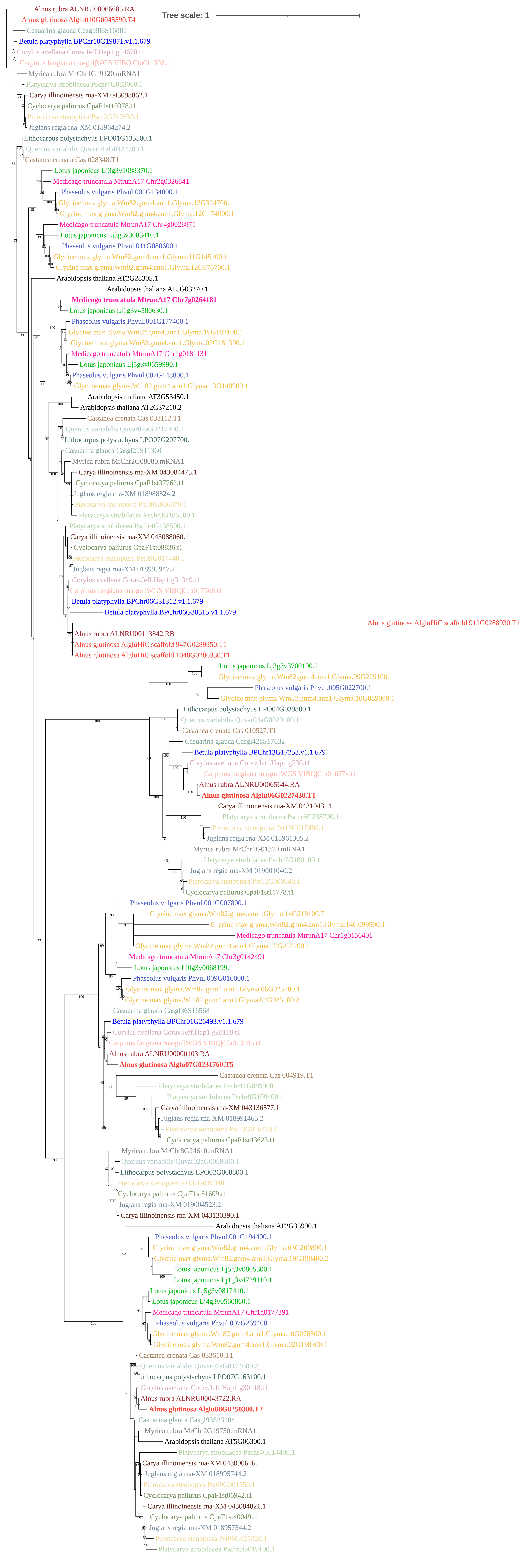

OG0000267:LIGHT-SENSITIVE SHORT HYPOCOTYL

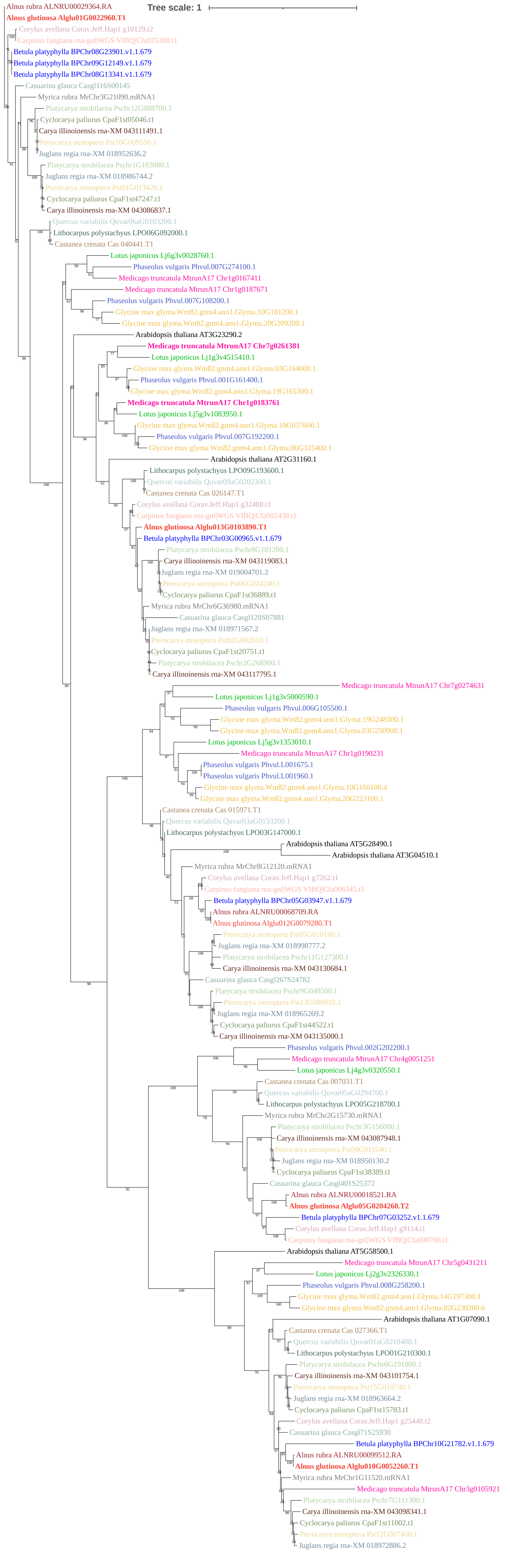

OG0000299:early nodulin 8lAAC26810

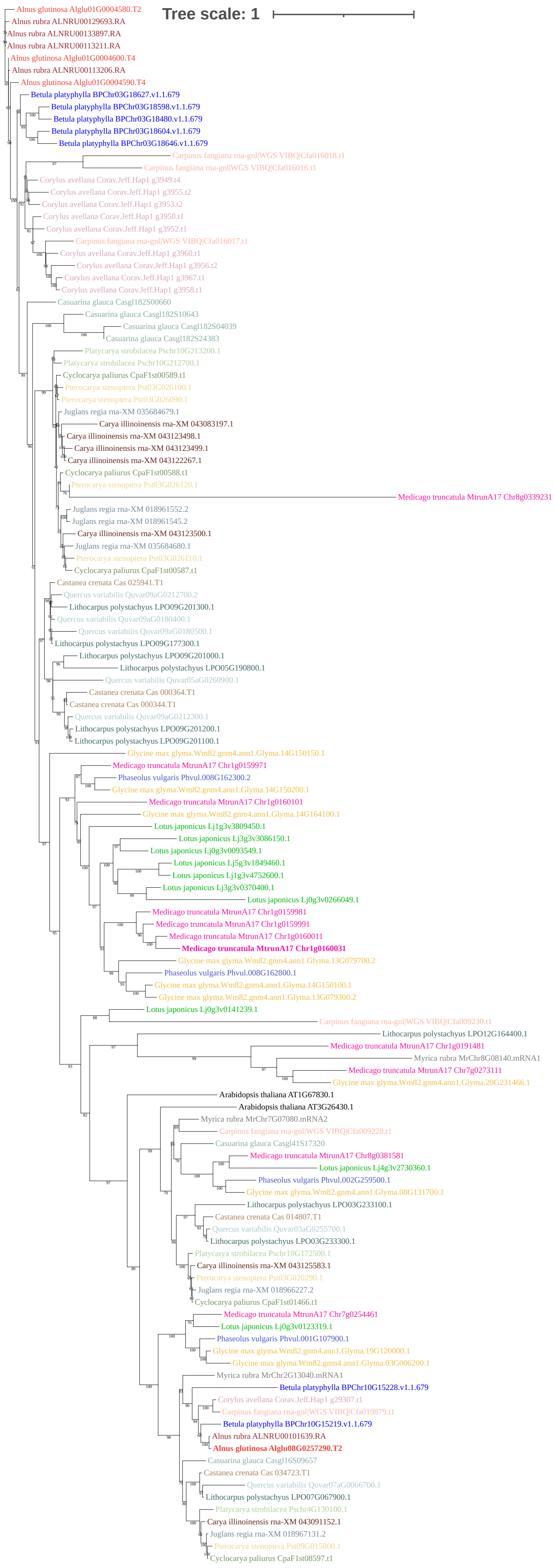

OG0000313:CHALCONE SYNTHASE

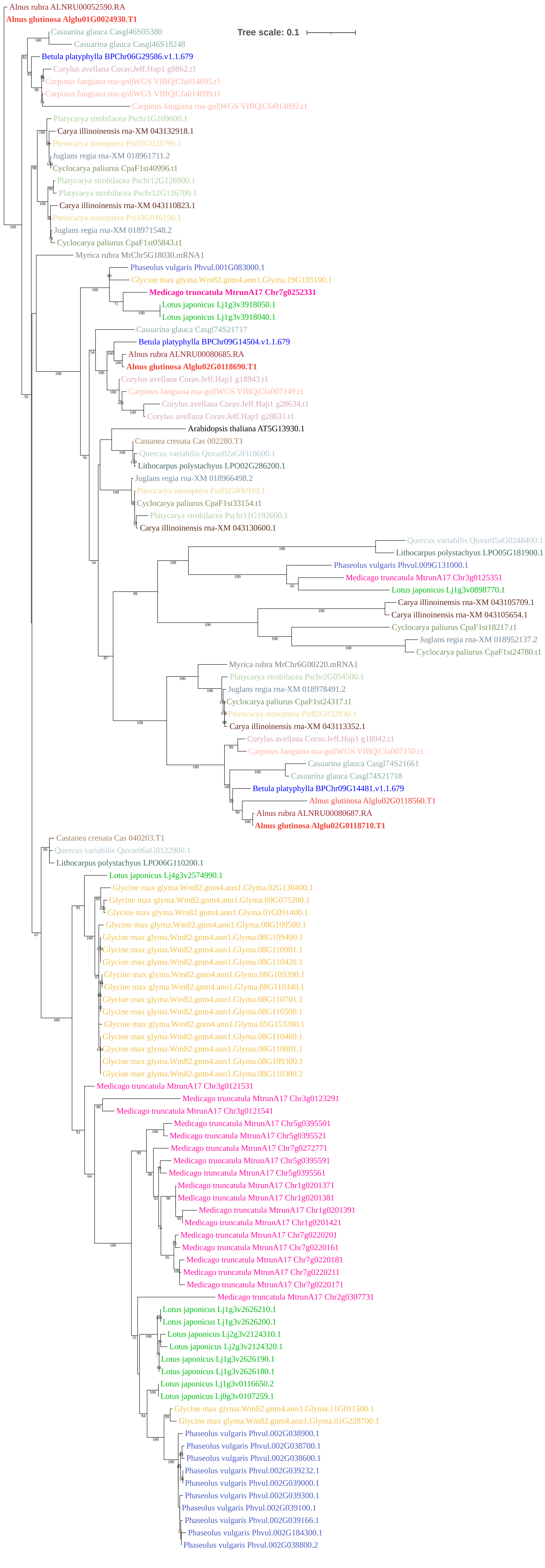

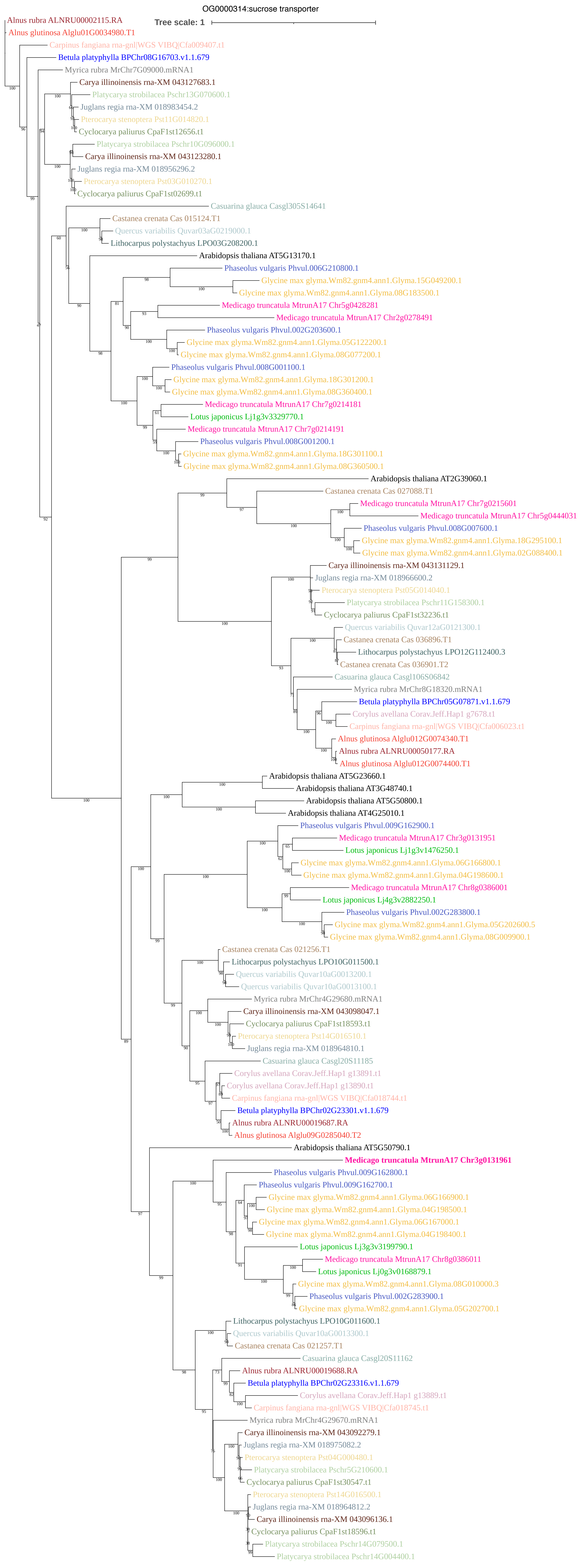

OG0000314:sucrose transporter
Tree scale: 1
Alnus rubra ALNRU00002115.RA
Alnus glutinosa Alglu01G0034980.T1
Carpinus fangiana rna-gnl|WGS VIBQ|Cfa009407.t1
Betula platyphylla BPChr08G16703.v1.1.679
Myrica rubra MrChr7G09000.mRNA1
Carya illinoinensis rna-XM 043127683.1
Platycarya strobilacea Pschr13G070600.1
Juglans regia rna-XM 018983454.2
Pterocarya stenoptera Pst11G014820.1
Cyclocarya paliurus CpaF1st12656.t1
Platycarya strobilacea Pschr10G096000.1
Carya illinoinensis rna-XM 043123280.1
Juglans regia rna-XM 018956296.2
Pterocarya stenoptera Pst03G010270.1
Cyclocarya paliurus CpaF1st02699.t1
Casuarina glauca Casgl305S14641
Castanea crenata Cas 015124.T1
Quercus variabilis Quvar03aG0219000.1
Lithocarpus polystachyus LPO03G208200.1
Arabidopsis thaliana AT5G13170.1
Phaseolus vulgaris Phvul.006G210800.1
Glycine max glyma.Wm82.gnm4.ann1.Glyma.15G049200.1
Glycine max glyma.Wm82.gnm4.ann1.Glyma.08G183500.1
Medicago truncatula MtrunA17 Chr5g0428281
Medicago truncatula MtrunA17 Chr2g0278491
Phaseolus vulgaris Phvul.002G203600.1
Glycine max glyma.Wm82.gnm4.ann1.Glyma.05G122200.1
Glycine max glyma.Wm82.gnm4.ann1.Glyma.08G077200.1
Phaseolus vulgaris Phvul.008G001100.1
Glycine max glyma.Wm82.gnm4.ann1.Glyma.18G301200.1
Glycine max glyma.Wm82.gnm4.ann1.Glyma.08G360400.1
Medicago truncatula MtrunA17 Chr7g0214181
Lotus japonicus Lj1g3v3329770.1
Medicago truncatula MtrunA17 Chr7g0214191
Phaseolus vulgaris Phvul.008G001200.1
Glycine max glyma.Wm82.gnm4.ann1.Glyma.18G301100.1
Glycine max glyma.Wm82.gnm4.ann1.Glyma.08G360500.1
Arabidopsis thaliana AT2G39060.1
Castanea crenata Cas 027088.T1
Medicago truncatula MtrunA17 Chr7g0215601
Medicago truncatula MtrunA17 Chr5g0444031
Phaseolus vulgaris Phvul.008G007600.1
Glycine max glyma.Wm82.gnm4.ann1.Glyma.18G295100.1
Glycine max glyma.Wm82.gnm4.ann1.Glyma.02G088400.1
Carya illinoinensis rna-XM 043131129.1
Juglans regia rna-XM 018966600.2
Pterocarya stenoptera Pst05G014040.1
Platycarya strobilacea Pschr11G158300.1
Cyclocarya paliurus CpaF1st32236.t1
Quercus variabilis Quvar12aG0121300.1
Castanea crenata Cas 036896.T1
Lithocarpus polystachyus LPO12G112400.3
Castanea crenata Cas 036901.T2
Casuarina glauca Casgl106S06842
Myrica rubra MrChr8G18320.mRNA1
Betula platyphylla BPChr05G07871.v1.1.679
Corylus avellana Corav.Jeff.Hap1 g7678.t1
Carpinus fangiana rna-gnl|WGS VIBQ|Cfa006023.t1
Alnus glutinosa Alglu012G0074340.T1
Alnus rubra ALNRU00050177.RA
Alnus glutinosa Alglu012G0074400.T1
Arabidopsis thaliana AT5G23660.1
Arabidopsis thaliana AT3G48740.1
Arabidopsis thaliana AT5G50800.1
Arabidopsis thaliana AT4G25010.1
Phaseolus vulgaris Phvul.009G162900.1
Medicago truncatula MtrunA17 Chr3g0131951
Lotus japonicus Lj1g3v1476250.1
Glycine max glyma.Wm82.gnm4.ann1.Glyma.06G166800.1
Glycine max glyma.Wm82.gnm4.ann1.Glyma.04G198600.1
Medicago truncatula MtrunA17 Chr8g0386001
Lotus japonicus Lj4g3v2882250.1
Phaseolus vulgaris Phvul.002G283800.1
Glycine max glyma.Wm82.gnm4.ann1.Glyma.05G202600.5
Glycine max glyma.Wm82.gnm4.ann1.Glyma.08G009900.1
Castanea crenata Cas 021256.T1
Lithocarpus polystachyus LPO10G011500.1
Quercus variabilis Quvar10aG0013200.1
Quercus variabilis Quvar10aG0013100.1
Myrica rubra MrChr4G29680.mRNA1
Carya illinoinensis rna-XM 043098047.1
Cyclocarya paliurus CpaF1st18593.t1
Pterocarya stenoptera Pst14G016510.1
Juglans regia rna-XM 018964810.1
Casuarina glauca Casgl20S11185
Corylus avellana Corav.Jeff.Hap1 g13891.t1
Corylus avellana Corav.Jeff.Hap1 g13890.t1
Carpinus fangiana rna-gnl|WGS VIBQ|Cfa018744.t1
Betula platyphylla BPChr02G23301.v1.1.679
Alnus rubra ALNRU00019687.RA
Alnus glutinosa Alglu09G0285040.T2
Arabidopsis thaliana AT5G50790.1
Medicago truncatula MtrunA17 Chr3g0131961
Phaseolus vulgaris Phvul.009G162800.1
Phaseolus vulgaris Phvul.009G162700.1
Glycine max glyma.Wm82.gnm4.ann1.Glyma.06G166900.1
Glycine max glyma.Wm82.gnm4.ann1.Glyma.04G198500.1
Glycine max glyma.Wm82.gnm4.ann1.Glyma.06G167000.1
Glycine max glyma.Wm82.gnm4.ann1.Glyma.04G198400.1
Lotus japonicus Lj3g3v3199790.1
Medicago truncatula MtrunA17 Chr8g0386011
Lotus japonicus Lj0g3v0168879.1
Glycine max glyma.Wm82.gnm4.ann1.Glyma.08G010000.3
Phaseolus vulgaris Phvul.002G283900.1
Glycine max glyma.Wm82.gnm4.ann1.Glyma.05G202700.1
Lithocarpus polystachyus LPO10G011600.1
Quercus variabilis Quvar10aG0013300.1
Castanea crenata Cas 021257.T1
Casuarina glauca Casgl20S11162
Alnus rubra ALNRU00019688.RA
Betula platyphylla BPChr02G23316.v1.1.679
Corylus avellana Corav.Jeff.Hap1 g13889.t1
Carpinus fangiana rna-gnl|WGS VIBQ|Cfa018745.t1
Myrica rubra MrChr4G29670.mRNA1
Carya illinoinensis rna-XM 043092279.1
Juglans regia rna-XM 018975082.2
Pterocarya stenoptera Pst04G000480.1
Platycarya strobilacea Pschr5G210600.1
Cyclocarya paliurus CpaF1st30547.t1
Pterocarya stenoptera Pst14G016500.1
Juglans regia rna-XM 018964812.2
Carya illinoinensis rna-XM 043096136.1
Cyclocarya paliurus CpaF1st18596.t1
Platycarya strobilacea Pschr14G079500.1
Platycarya strobilacea Pschr14G004400.1

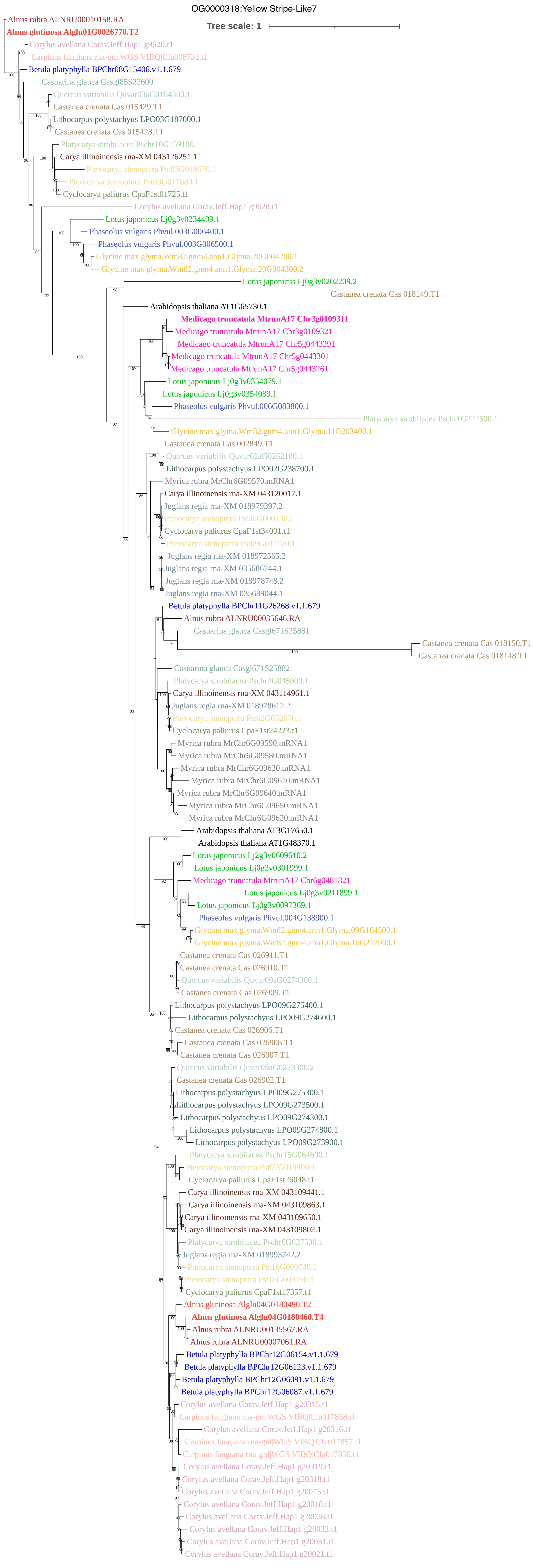
OG0000318:Yellow Stripe-Like7
Tree scale: 1
Alnus rubra ALNRU00010158.RA
Alnus glutinosa Alglu01G0026770.T2
Corylus avellana Corav.Jeff.Hap1 g9620.t1
Carpinus fangiana rna-gnl|WGS VIBQ|Cfa008731.t1
Betula platyphylla BPChr08G15406.v1.1.679
Casuarina glauca Casgl85S22600
Quercus variabilis Quvar03aG0184300.1
Castanea crenata Cas 015429.T1
Lithocarpus polystachyus LPO03G187000.1
Castanea crenata Cas 015428.T1
Platycarya strobilacea Pschr10G159100.1
Carya illinoinensis rna-XM 043126251.1
Pterocarya stenoptera Pst03G019670.1
Pterocarya stenoptera Pst03G017880.1
Cyclocarya paliurus CpaF1st01725.t1
Corylus avellana Corav.Jeff.Hap1 g9628.t1
Lotus japonicus Lj0g3v0234409.1
Phaseolus vulgaris Phvul.003G006400.1
Phaseolus vulgaris Phvul.003G006500.1
Glycine max glyma.Wm82.gnm4.ann1.Glyma.20G004200.1
Glycine max glyma.Wm82.gnm4.ann1.Glyma.20G004300.2
Lotus japonicus Lj0g3v0202209.2
Castanea crenata Cas 018149.T1
Arabidopsis thaliana AT1G65730.1
Medicago truncatula MtrunA17 Chr3g0109311
Medicago truncatula MtrunA17 Chr3g0109321
Medicago truncatula MtrunA17 Chr5g0443291
Medicago truncatula MtrunA17 Chr5g0443301
Medicago truncatula MtrunA17 Chr5g0443261
Lotus japonicus Lj0g3v0354079.1
Lotus japonicus Lj0g3v0354089.1
Phaseolus vulgaris Phvul.006G083800.1
Platycarya strobilacea Pschr1G222500.1
Glycine max glyma.Wm82.gnm4.ann1.Glyma.11G203400.1
Castanea crenata Cas 002849.T1
Quercus variabilis Quvar02aG0262100.1
Lithocarpus polystachyus LPO02G238700.1
Myrica rubra MrChr6G09570.mRNA1
Carya illinoinensis rna-XM 043120017.1
Juglans regia rna-XM 018979397.2
Pterocarya stenoptera Pst06G000730.1
Cyclocarya paliurus CpaF1st34091.t1
Pterocarya stenoptera Pst09G011120.1
Juglans regia rna-XM 018972565.2
Juglans regia rna-XM 035686744.1
Juglans regia rna-XM 018978748.2
Juglans regia rna-XM 035689044.1
Betula platyphylla BPChr11G26268.v1.1.679
Alnus rubra ALNRU00035646.RA
Casuarina glauca Casgl671S25881
Castanea crenata Cas 018150.T1
Castanea crenata Cas 018148.T1
Casuarina glauca Casgl671S25882
Platycarya strobilacea Pschr2G045000.1
Carya illinoinensis rna-XM 043114961.1
Juglans regia rna-XM 018978612.2
Pterocarya stenoptera Pst02G032070.1
Cyclocarya paliurus CpaF1st24223.t1
Myrica rubra MrChr6G09590.mRNA1
Myrica rubra MrChr6G09580.mRNA1
Myrica rubra MrChr6G09630.mRNA1
Myrica rubra MrChr6G09610.mRNA1
Myrica rubra MrChr6G09640.mRNA1
Myrica rubra MrChr6G09650.mRNA1
Myrica rubra MrChr6G09620.mRNA1
Arabidopsis thaliana AT3G17650.1
Arabidopsis thaliana AT1G48370.1
Lotus japonicus Lj2g3v0609610.2
Lotus japonicus Lj0g3v0301999.1
Medicago truncatula MtrunA17 Chr6g0481821
Lotus japonicus Lj0g3v0211899.1
Lotus japonicus Lj0g3v0097369.1
Phaseolus vulgaris Phvul.004G138900.1
Glycine max glyma.Wm82.gnm4.ann1.Glyma.09G164500.1
Glycine max glyma.Wm82.gnm4.ann1.Glyma.16G212900.1
Castanea crenata Cas 026911.T1
Castanea crenata Cas 026910.T1
Quercus variabilis Quvar09aG0274300.1
Castanea crenata Cas 026909.T1
Lithocarpus polystachyus LPO09G275400.1
Lithocarpus polystachyus LPO09G274600.1
Castanea crenata Cas 026906.T1
Castanea crenata Cas 026908.T1
Castanea crenata Cas 026907.T1
Quercus variabilis Quvar09aG0273300.2
Castanea crenata Cas 026902.T1
Lithocarpus polystachyus LPO09G275300.1
Lithocarpus polystachyus LPO09G273500.1
Lithocarpus polystachyus LPO09G274300.1
Lithocarpus polystachyus LPO09G274800.1
Lithocarpus polystachyus LPO09G273900.1
Platycarya strobilacea Pschr15G064600.1
Pterocarya stenoptera Pst07G013900.1
Cyclocarya paliurus CpaF1st26048.t1
Carya illinoinensis rna-XM 043109441.1
Carya illinoinensis rna-XM 043109863.1
Carya illinoinensis rna-XM 043109650.1
Carya illinoinensis rna-XM 043109802.1
Platycarya strobilacea Pschr6G037500.1
Juglans regia rna-XM 018993742.2
Pterocarya stenoptera Pst16G009740.1
Pterocarya stenoptera Pst16G009750.1
Cyclocarya paliurus CpaF1st17357.t1
Alnus glutinosa Alglu04G0180490.T2
Alnus glutinosa Alglu04G0180460.T4
Alnus rubra ALNRU00135567.RA
Alnus rubra ALNRU00007061.RA
Betula platyphylla BPChr12G06154.v1.1.679
Betula platyphylla BPChr12G06123.v1.1.679
Betula platyphylla BPChr12G06091.v1.1.679
Betula platyphylla BPChr12G06087.v1.1.679
Corylus avellana Corav.Jeff.Hap1 g20315.t1
Carpinus fangiana rna-gnl|WGS VIBQ|Cfa017858.t1
Corylus avellana Corav.Jeff.Hap1 g20316.t1
Carpinus fangiana rna-gnl|WGS VIBQ|Cfa017857.t1
Carpinus fangiana rna-gnl|WGS VIBQ|Cfa017856.t1
Corylus avellana Corav.Jeff.Hap1 g20319.t1
Corylus avellana Corav.Jeff.Hap1 g20318.t1
Corylus avellana Corav.Jeff.Hap1 g20015.t1
Corylus avellana Corav.Jeff.Hap1 g20018.t1
Corylus avellana Corav.Jeff.Hap1 g20028.t1
Corylus avellana Corav.Jeff.Hap1 g20033.t1
Corylus avellana Corav.Jeff.Hap1 g20031.t1
Corylus avellana Corav.Jeff.Hap1 g20021.t1

OG0000338:ISOFLAVONE SYNTHASE/Cytochrome P450 monooxygenase

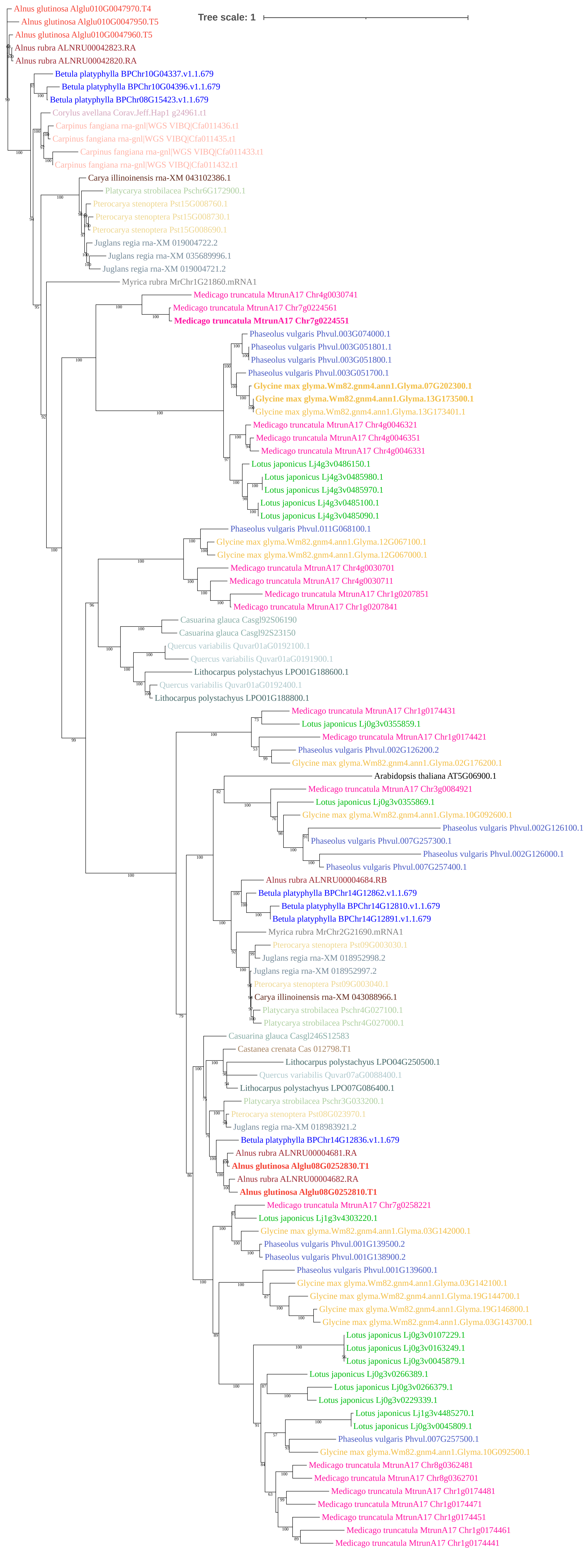

OG0000343:ETHYLENE RESPONSE FACTOR 1

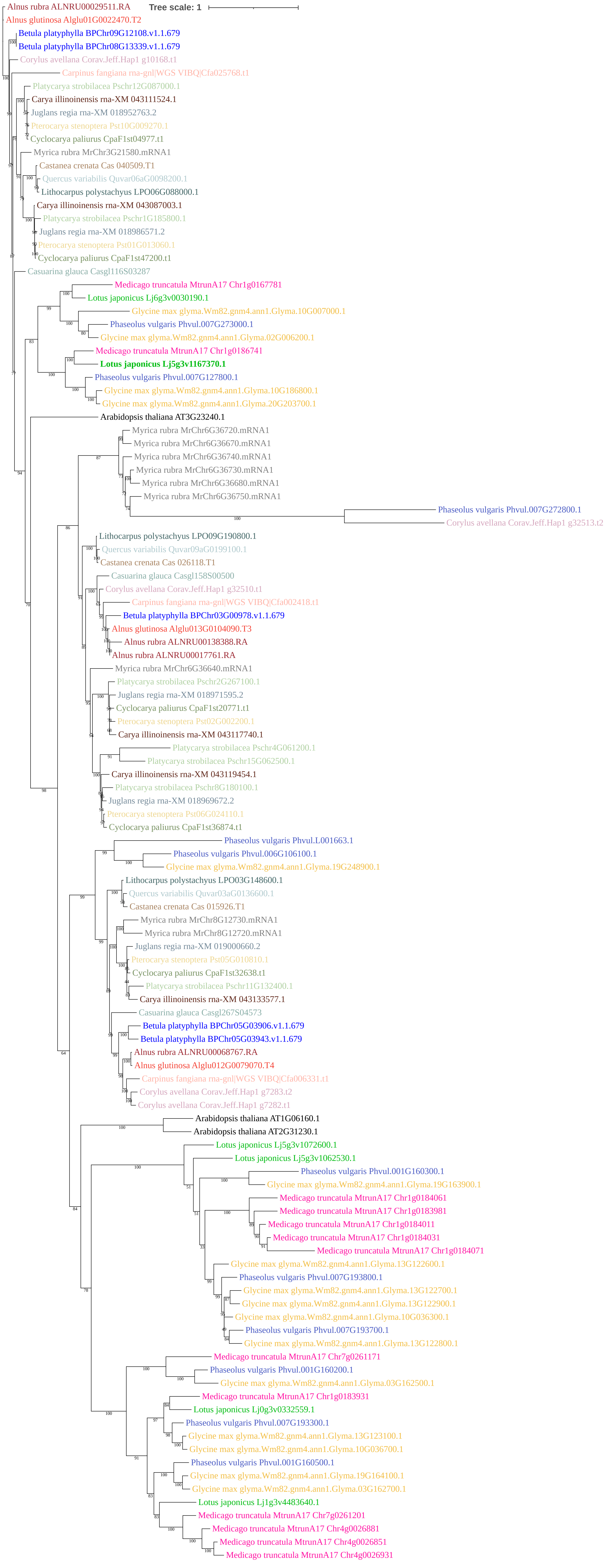

OG0000350:LIKE-AUX1

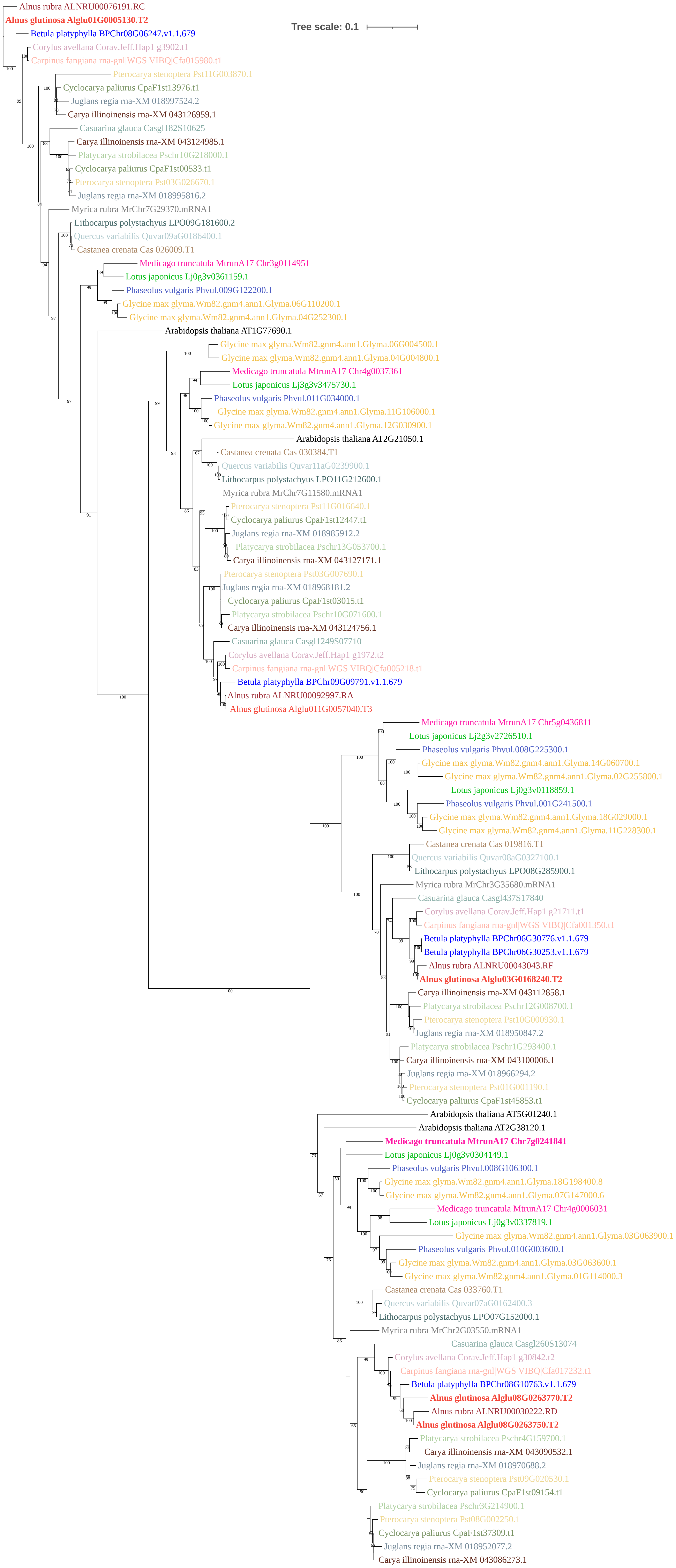

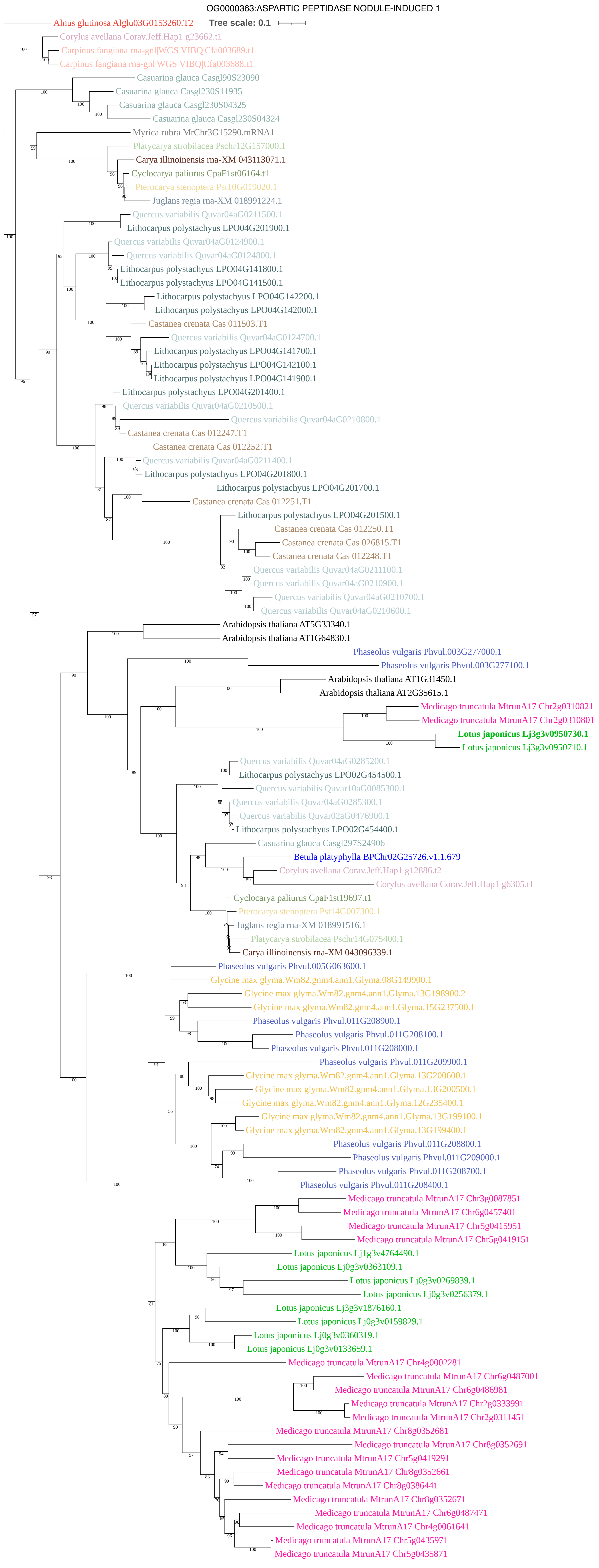
OG0000363:ASPARTIC PEPTIDASE NODULE-INDUCED 1
Tree scale: 0.1
Alnus glutinosa Alglu03G0153260.T2
Corylus avellana Corav.Jeff.Hap1 g23662.t1
Carpinus fangiana rna-gnl|WGS VIBQ|Cfa003689.t1
Carpinus fangiana rna-gnl|WGS VIBQ|Cfa003688.t1
Casuarina glauca Casgl90S23090
Casuarina glauca Casgl230S11935
Casuarina glauca Casgl230S04325
Casuarina glauca Casgl230S04324
Myrica rubra MrChr3G15290.mRNA1
Platycarya strobilacea Pschr12G157000.1
Carya illinoinensis rna-XM 043113071.1
Cyclocarya paliurus CpaF1st06164.t1
Pterocarya stenoptera Pst10G019020.1
Juglans regia rna-XM 018991224.1
Quercus variabilis Quvar04aG0211500.1
Lithocarpus polystachyus LPO04G201900.1
Quercus variabilis Quvar04aG0124900.1
Quercus variabilis Quvar04aG0124800.1
Lithocarpus polystachyus LPO04G141800.1
Lithocarpus polystachyus LPO04G141500.1
Lithocarpus polystachyus LPO04G142200.1
Lithocarpus polystachyus LPO04G142000.1
Castanea crenata Cas 011503.T1
Quercus variabilis Quvar04aG0124700.1
Lithocarpus polystachyus LPO04G141700.1
Lithocarpus polystachyus LPO04G142100.1
Lithocarpus polystachyus LPO04G141900.1
Lithocarpus polystachyus LPO04G201400.1
Quercus variabilis Quvar04aG0210500.1
Quercus variabilis Quvar04aG0210800.1
Castanea crenata Cas 012247.T1
Castanea crenata Cas 012252.T1
Quercus variabilis Quvar04aG0211400.1
Lithocarpus polystachyus LPO04G201800.1
Lithocarpus polystachyus LPO04G201700.1
Castanea crenata Cas 012251.T1
Lithocarpus polystachyus LPO04G201500.1
Castanea crenata Cas 012250.T1
Castanea crenata Cas 026815.T1
Castanea crenata Cas 012248.T1
Quercus variabilis Quvar04aG0211100.1
Quercus variabilis Quvar04aG0210900.1
Quercus variabilis Quvar04aG0210700.1
Quercus variabilis Quvar04aG0210600.1
Arabidopsis thaliana AT5G33340.1
Arabidopsis thaliana AT1G64830.1
Phaseolus vulgaris Phvul.003G277000.1
Phaseolus vulgaris Phvul.003G277100.1
Arabidopsis thaliana AT1G31450.1
Arabidopsis thaliana AT2G35615.1
Medicago truncatula MtrunA17 Chr2g0310821
Medicago truncatula MtrunA17 Chr2g0310801
Lotus japonicus Lj3g3v0950730.1
Lotus japonicus Lj3g3v0950710.1
Quercus variabilis Quvar04aG0285200.1
Lithocarpus polystachyus LPO02G454500.1
Quercus variabilis Quvar10aG0085300.1
Quercus variabilis Quvar04aG0285300.1
Quercus variabilis Quvar02aG0476900.1
Lithocarpus polystachyus LPO02G454400.1
Casuarina glauca Casgl297S24906
Betula platyphylla BPChr02G25726.v1.1.679
Corylus avellana Corav.Jeff.Hap1 g12886.t2
Corylus avellana Corav.Jeff.Hap1 g6305.t1
Cyclocarya paliurus CpaF1st19697.t1
Pterocarya stenoptera Pst14G007300.1
Juglans regia rna-XM 018991516.1
Platycarya strobilacea Pschr14G075400.1
Carya illinoinensis rna-XM 043096339.1
Phaseolus vulgaris Phvul.005G063600.1
Glycine max glyma.Wm82.gnm4.ann1.Glyma.08G149900.1
Glycine max glyma.Wm82.gnm4.ann1.Glyma.13G198900.2
Glycine max glyma.Wm82.gnm4.ann1.Glyma.15G237500.1
Phaseolus vulgaris Phvul.011G208900.1
Phaseolus vulgaris Phvul.011G208100.1
Phaseolus vulgaris Phvul.011G208000.1
Phaseolus vulgaris Phvul.011G209900.1
Glycine max glyma.Wm82.gnm4.ann1.Glyma.13G200600.1
Glycine max glyma.Wm82.gnm4.ann1.Glyma.13G200500.1
Glycine max glyma.Wm82.gnm4.ann1.Glyma.12G235400.1
Glycine max glyma.Wm82.gnm4.ann1.Glyma.13G199100.1
Glycine max glyma.Wm82.gnm4.ann1.Glyma.13G199400.1
Phaseolus vulgaris Phvul.011G208800.1
Phaseolus vulgaris Phvul.011G209000.1
Phaseolus vulgaris Phvul.011G208700.1
Phaseolus vulgaris Phvul.011G208400.1
Medicago truncatula MtrunA17 Chr3g0087851
Medicago truncatula MtrunA17 Chr6g0457401
Medicago truncatula MtrunA17 Chr5g0415951
Medicago truncatula MtrunA17 Chr5g0419151
Lotus japonicus Lj1g3v4764490.1
Lotus japonicus Lj0g3v0363109.1
Lotus japonicus Lj0g3v0269839.1
Lotus japonicus Lj0g3v0256379.1
Lotus japonicus Lj3g3v1876160.1
Lotus japonicus Lj0g3v0159829.1
Lotus japonicus Lj0g3v0360319.1
Lotus japonicus Lj0g3v0133659.1
Medicago truncatula MtrunA17 Chr4g0002281
Medicago truncatula MtrunA17 Chr6g0487001
Medicago truncatula MtrunA17 Chr6g0486981
Medicago truncatula MtrunA17 Chr2g0333991
Medicago truncatula MtrunA17 Chr2g0311451
Medicago truncatula MtrunA17 Chr8g0352681
Medicago truncatula MtrunA17 Chr8g0352691
Medicago truncatula MtrunA17 Chr5g0419291
Medicago truncatula MtrunA17 Chr8g0352661
Medicago truncatula MtrunA17 Chr8g0386441
Medicago truncatula MtrunA17 Chr8g0352671
Medicago truncatula MtrunA17 Chr6g0487471
Medicago truncatula MtrunA17 Chr4g0061641
Medicago truncatula MtrunA17 Chr5g0435971
Medicago truncatula MtrunA17 Chr5g0435871

OG0000405:SUPERNUMERIC NODULE NUMBER

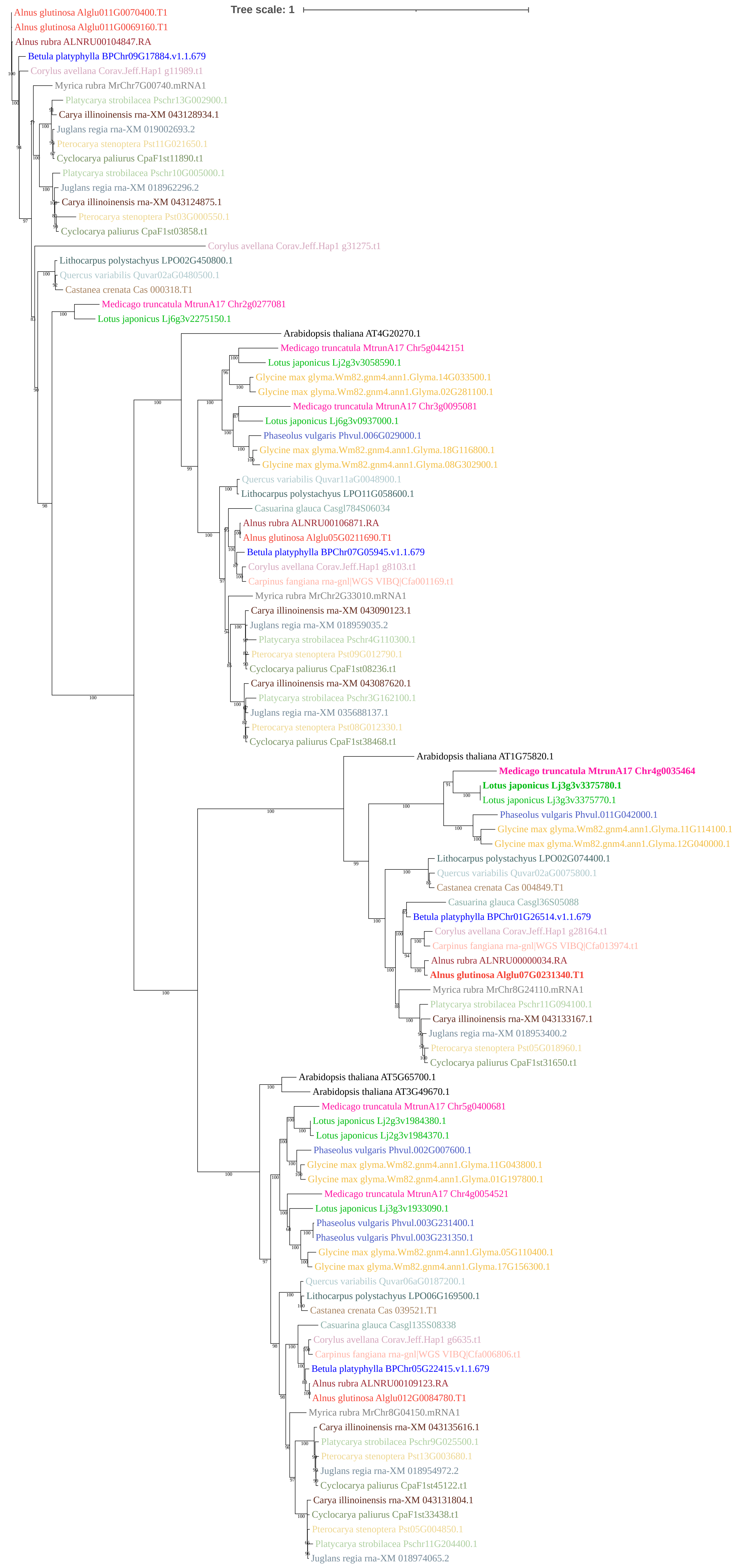

OG0000414:SYMBIOTIC AMMONIA TRANSPORTER/BASIC HELIX?LOOP?HELIX 476

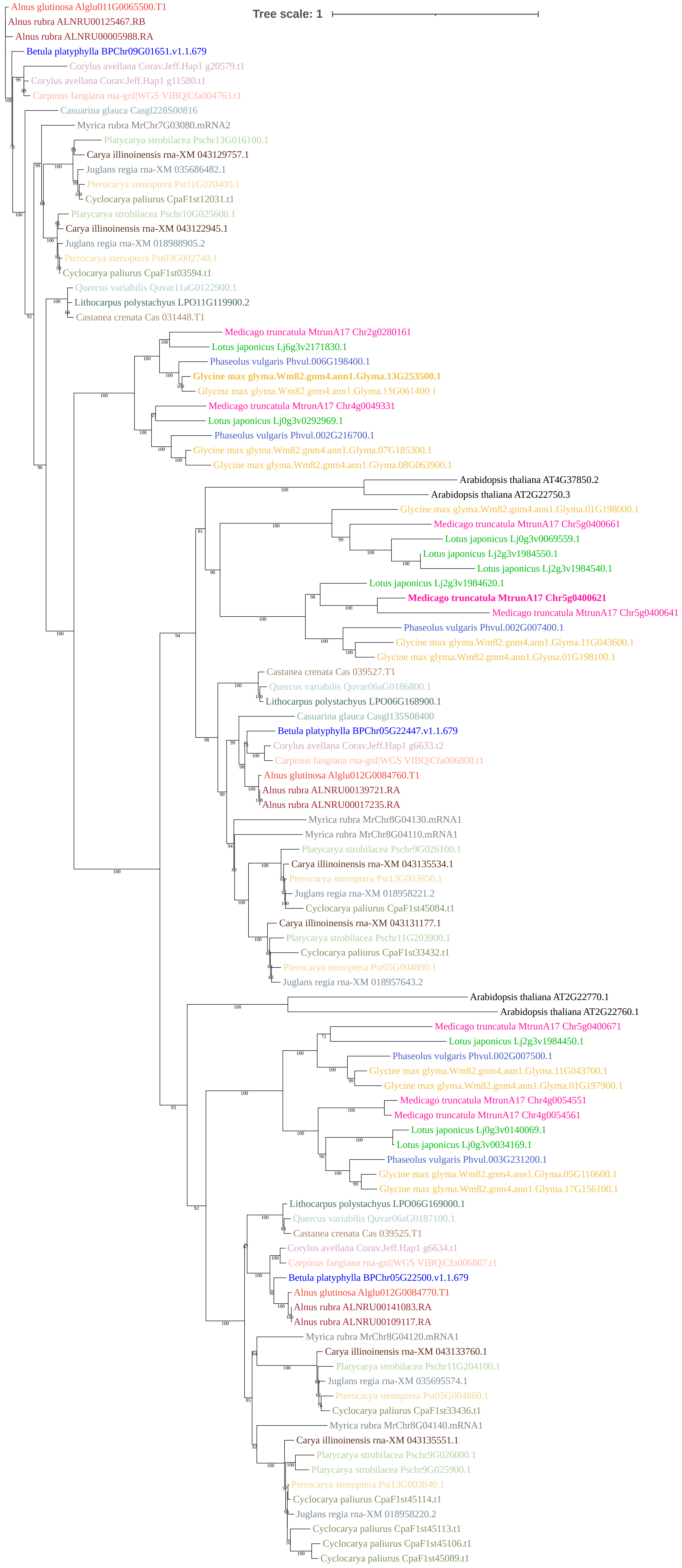

OG0000419:SERPIN

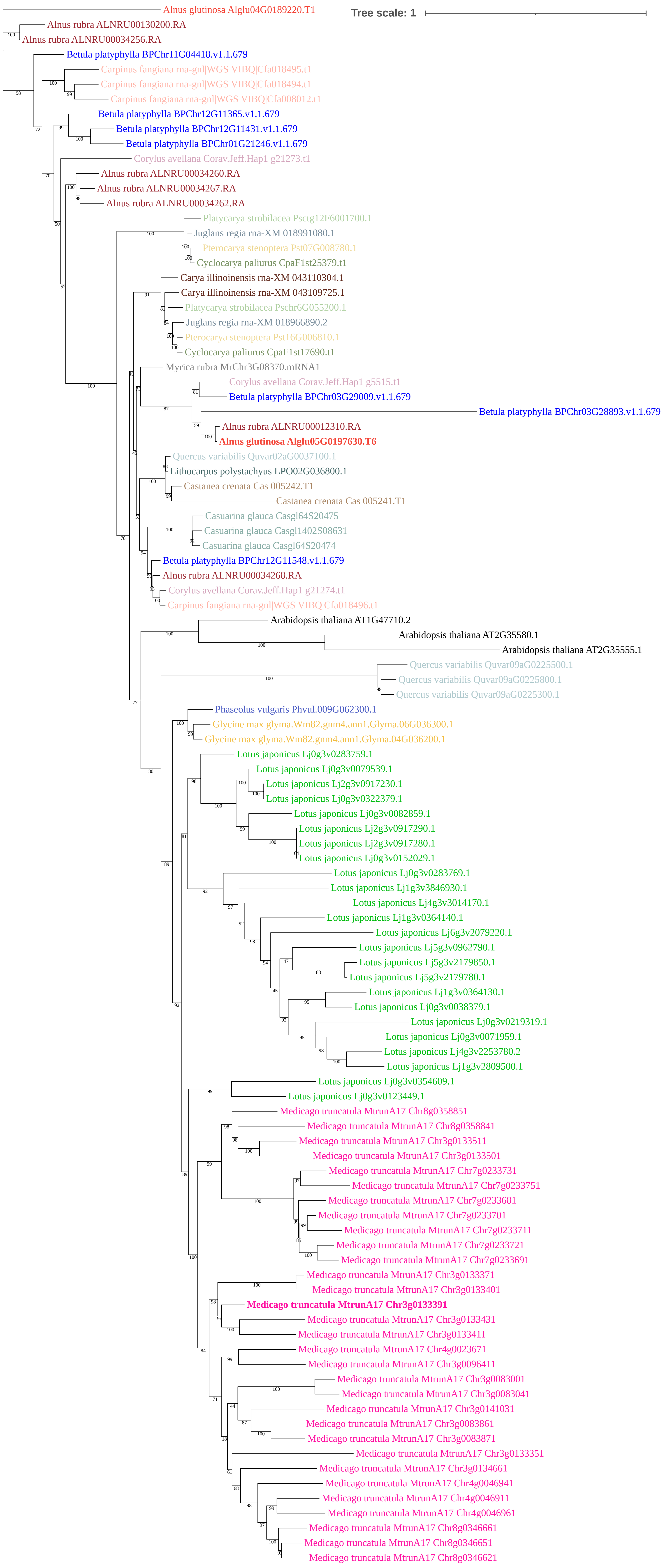

OG0000439:EXOCYST70H4

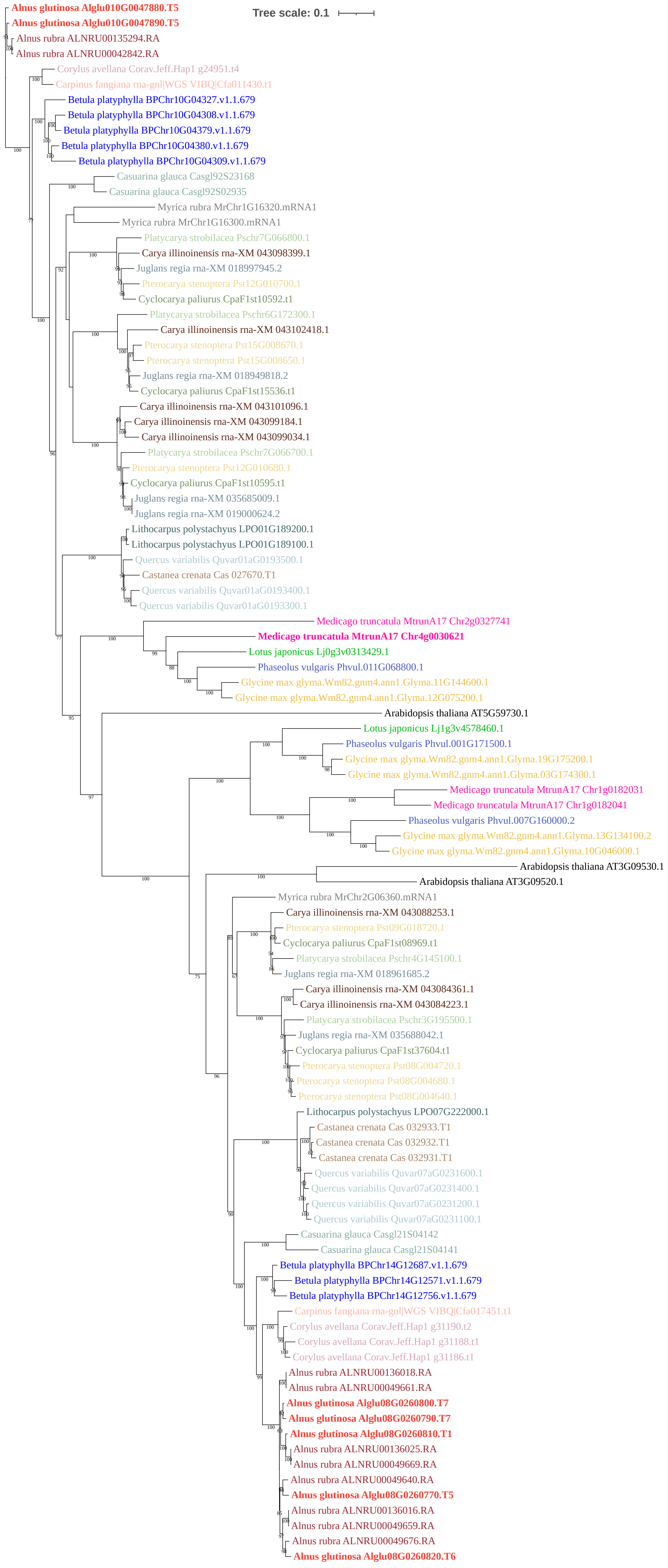

OG0000440:N-terminal MADS-box domain

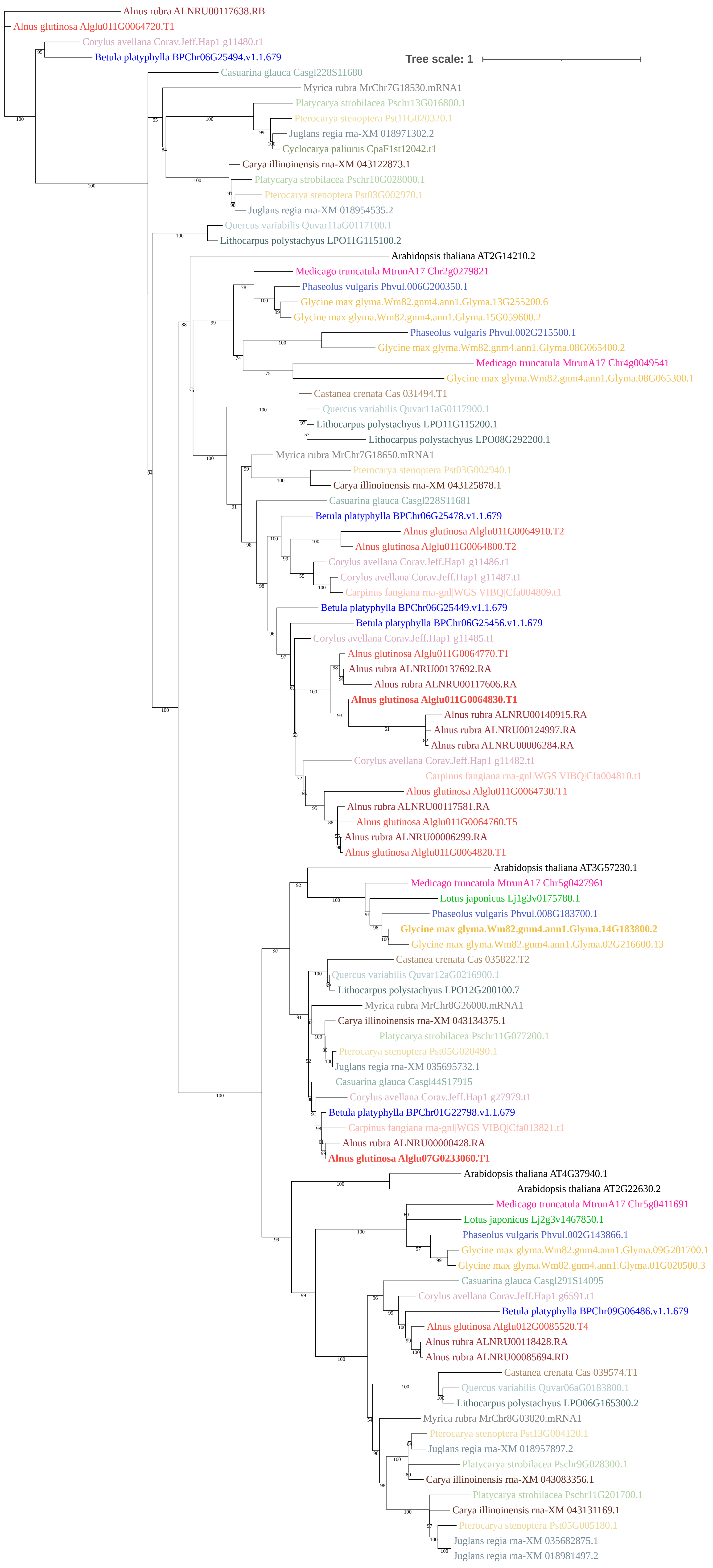

OG0000454:G-box Factor 14-3-3c

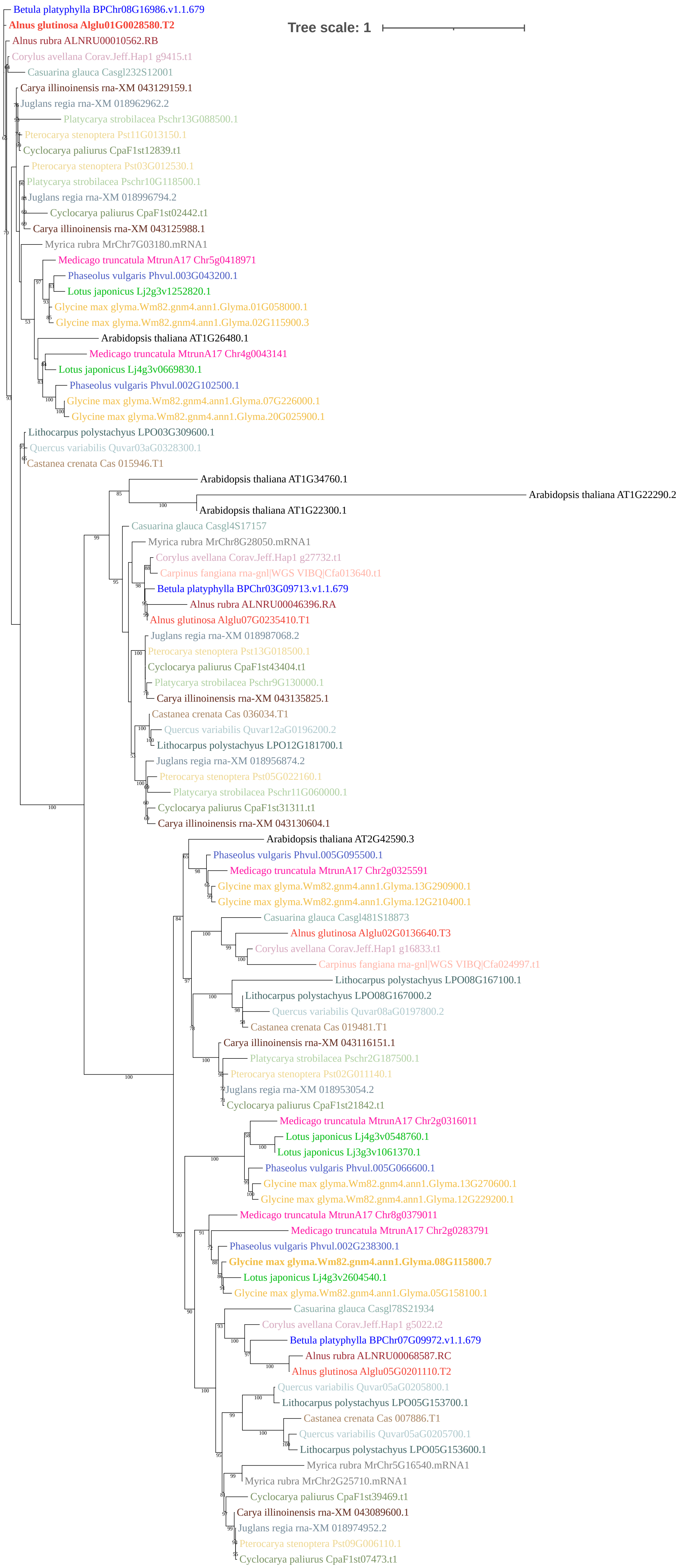

OG0000502:lateral root organ defective/NUMEROUS INFECTIONS AND POLYPHENOLICSINRT1/ PTR FAMILYInitrate / peptide transporter family

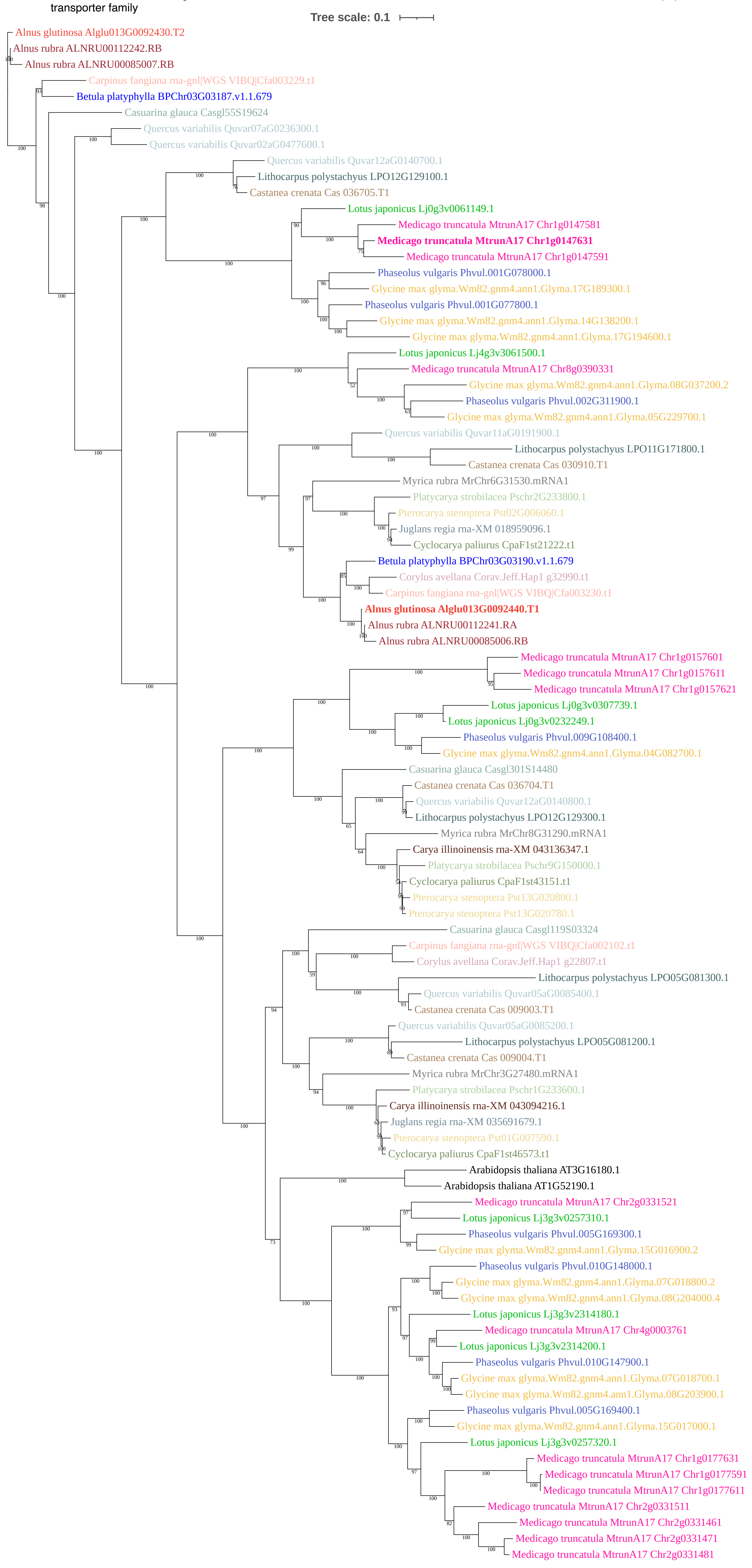

OG0000516:Natural Resistance-Associated Macrophage Protein1

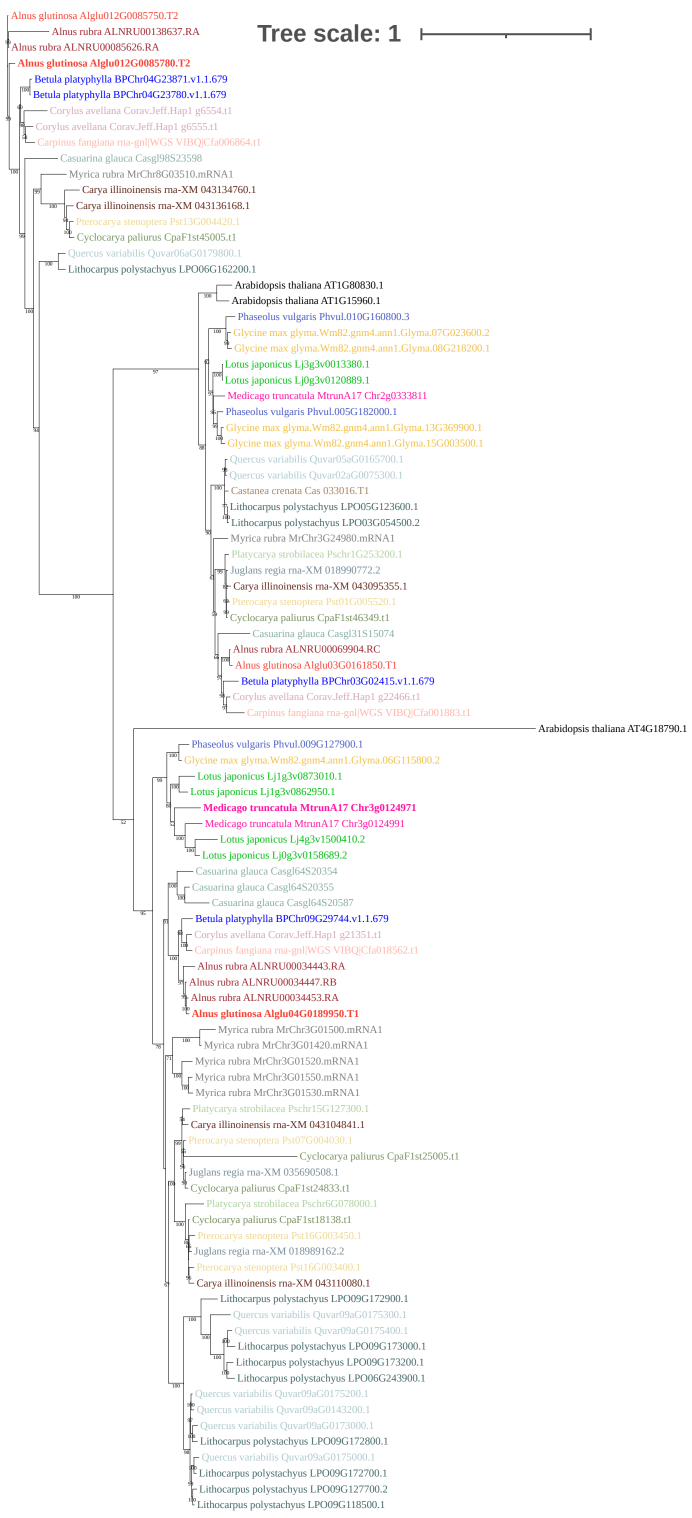

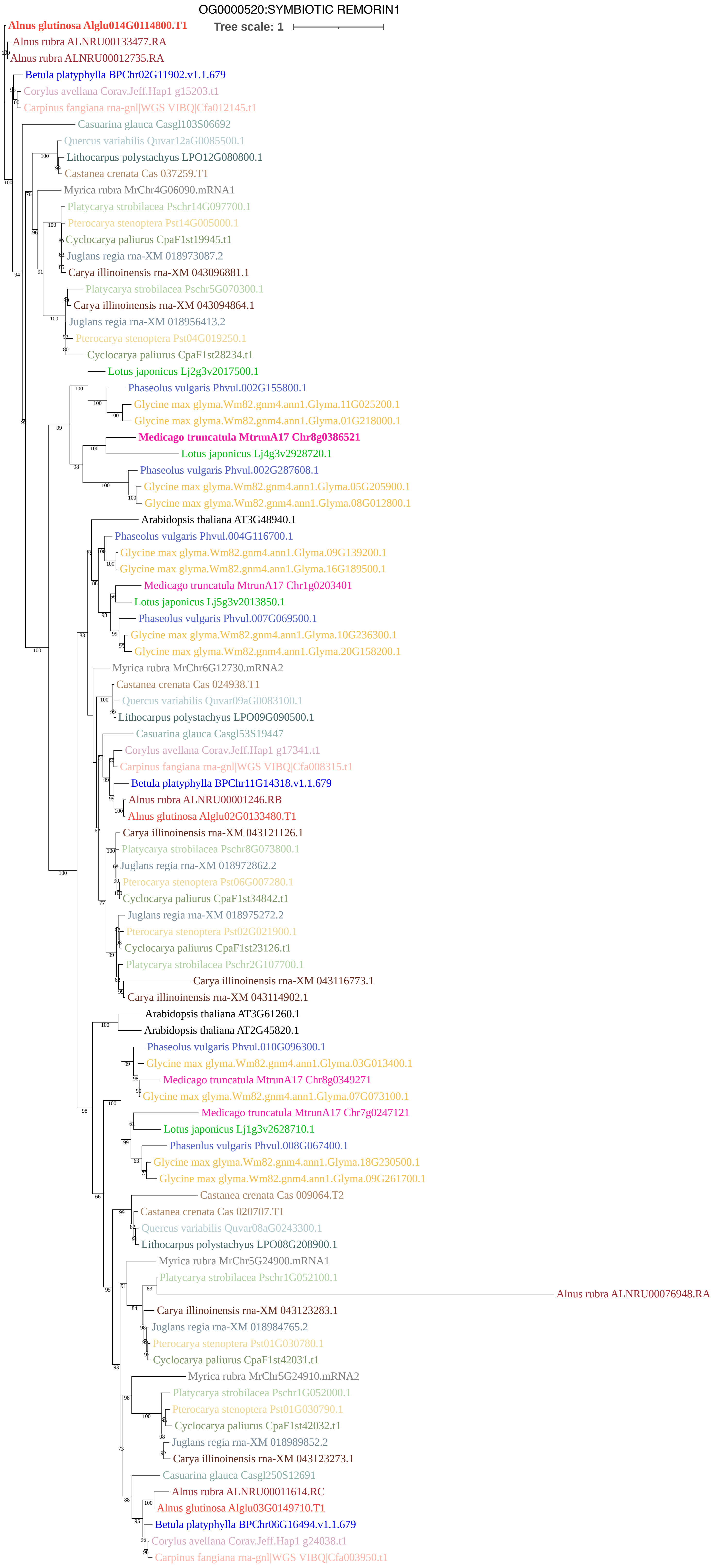
OG0000520:SYMBIOTIC REMORIN1
Tree scale: 1
Alnus glutinosa Alglu014G0114800.T1
Alnus rubra ALNRU00133477.RA
Alnus rubra ALNRU00012735.RA
Betula platyphylla BPChr02G11902.v1.1.679
Corylus avellana Corav.Jeff.Hap1 g15203.t1
Carpinus fangiana rna-gnl|WGS VIBQ|Cfa012145.t1
Casuarina glauca Casgl103S06692
Quercus variabilis Quvar12aG0085500.1
Lithocarpus polystachyus LPO12G080800.1
Castanea crenata Cas 037259.T1
Myrica rubra MrChr4G06090.mRNA1
Platycarya strobilacea Pschr14G097700.1
Pterocarya stenoptera Pst14G005000.1
Cyclocarya paliurus CpaF1st19945.t1
Juglans regia rna-XM 018973087.2
Carya illinoinensis rna-XM 043096881.1
Platycarya strobilacea Pschr5G070300.1
Carya illinoinensis rna-XM 043094864.1
Juglans regia rna-XM 018956413.2
Pterocarya stenoptera Pst04G019250.1
Cyclocarya paliurus CpaF1st28234.t1
Lotus japonicus Lj2g3v2017500.1
Phaseolus vulgaris Phvul.002G155800.1
Glycine max glyma.Wm82.gnm4.ann1.Glyma.11G025200.1
Glycine max glyma.Wm82.gnm4.ann1.Glyma.01G218000.1
Medicago truncatula MtrunA17 Chr8g0386521
Lotus japonicus Lj4g3v2928720.1
Phaseolus vulgaris Phvul.002G287608.1
Glycine max glyma.Wm82.gnm4.ann1.Glyma.05G205900.1
Glycine max glyma.Wm82.gnm4.ann1.Glyma.08G012800.1
Arabidopsis thaliana AT3G48940.1
Phaseolus vulgaris Phvul.004G116700.1
Glycine max glyma.Wm82.gnm4.ann1.Glyma.09G139200.1
Glycine max glyma.Wm82.gnm4.ann1.Glyma.16G189500.1
Medicago truncatula MtrunA17 Chr1g0203401
Lotus japonicus Lj5g3v2013850.1
Phaseolus vulgaris Phvul.007G069500.1
Glycine max glyma.Wm82.gnm4.ann1.Glyma.10G236300.1
Glycine max glyma.Wm82.gnm4.ann1.Glyma.20G158200.1
Myrica rubra MrChr6G12730.mRNA2
Castanea crenata Cas 024938.T1
Quercus variabilis Quvar09aG0083100.1
Lithocarpus polystachyus LPO09G090500.1
Casuarina glauca Casgl53S19447
Corylus avellana Corav.Jeff.Hap1 g17341.t1
Carpinus fangiana rna-gnl|WGS VIBQ|Cfa008315.t1
Betula platyphylla BPChr11G14318.v1.1.679
Alnus rubra ALNRU00001246.RB
Alnus glutinosa Alglu02G0133480.T1
Carya illinoinensis rna-XM 043121126.1
Platycarya strobilacea Pschr8G073800.1
Juglans regia rna-XM 018972862.2
Pterocarya stenoptera Pst06G007280.1
Cyclocarya paliurus CpaF1st34842.t1
Juglans regia rna-XM 018975272.2
Pterocarya stenoptera Pst02G021900.1
Cyclocarya paliurus CpaF1st23126.t1
Platycarya strobilacea Pschr2G107700.1
Carya illinoinensis rna-XM 043116773.1
Carya illinoinensis rna-XM 043114902.1
Arabidopsis thaliana AT3G61260.1
Arabidopsis thaliana AT2G45820.1
Phaseolus vulgaris Phvul.010G096300.1
Glycine max glyma.Wm82.gnm4.ann1.Glyma.03G013400.1
Medicago truncatula MtrunA17 Chr8g0349271
Glycine max glyma.Wm82.gnm4.ann1.Glyma.07G073100.1
Medicago truncatula MtrunA17 Chr7g0247121
Lotus japonicus Lj1g3v2628710.1
Phaseolus vulgaris Phvul.008G067400.1
Glycine max glyma.Wm82.gnm4.ann1.Glyma.18G230500.1
Glycine max glyma.Wm82.gnm4.ann1.Glyma.09G261700.1
Castanea crenata Cas 009064.T2
Castanea crenata Cas 020707.T1
Quercus variabilis Quvar08aG0243300.1
Lithocarpus polystachyus LPO08G208900.1
Myrica rubra MrChr5G24900.mRNA1
Platycarya strobilacea Pschr1G052100.1
Alnus rubra ALNRU00076948.RA
Carya illinoinensis rna-XM 043123283.1
Juglans regia rna-XM 018984765.2
Pterocarya stenoptera Pst01G030780.1
Cyclocarya paliurus CpaF1st42031.t1
Myrica rubra MrChr5G24910.mRNA2
Platycarya strobilacea Pschr1G052000.1
Pterocarya stenoptera Pst01G030790.1
Cyclocarya paliurus CpaF1st42032.t1
Juglans regia rna-XM 018989852.2
Carya illinoinensis rna-XM 043123273.1
Casuarina glauca Casgl250S12691
Alnus rubra ALNRU00011614.RC
Alnus glutinosa Alglu03G0149710.T1
Betula platyphylla BPChr06G16494.v1.1.679
Corylus avellana Corav.Jeff.Hap1 g24038.t1
Carpinus fangiana rna-gnl|WGS VIBQ|Cfa003950.t1

OG0000532:regulating nodulation factor receptor (NFR)-interacting RING-type E3 ligase 1

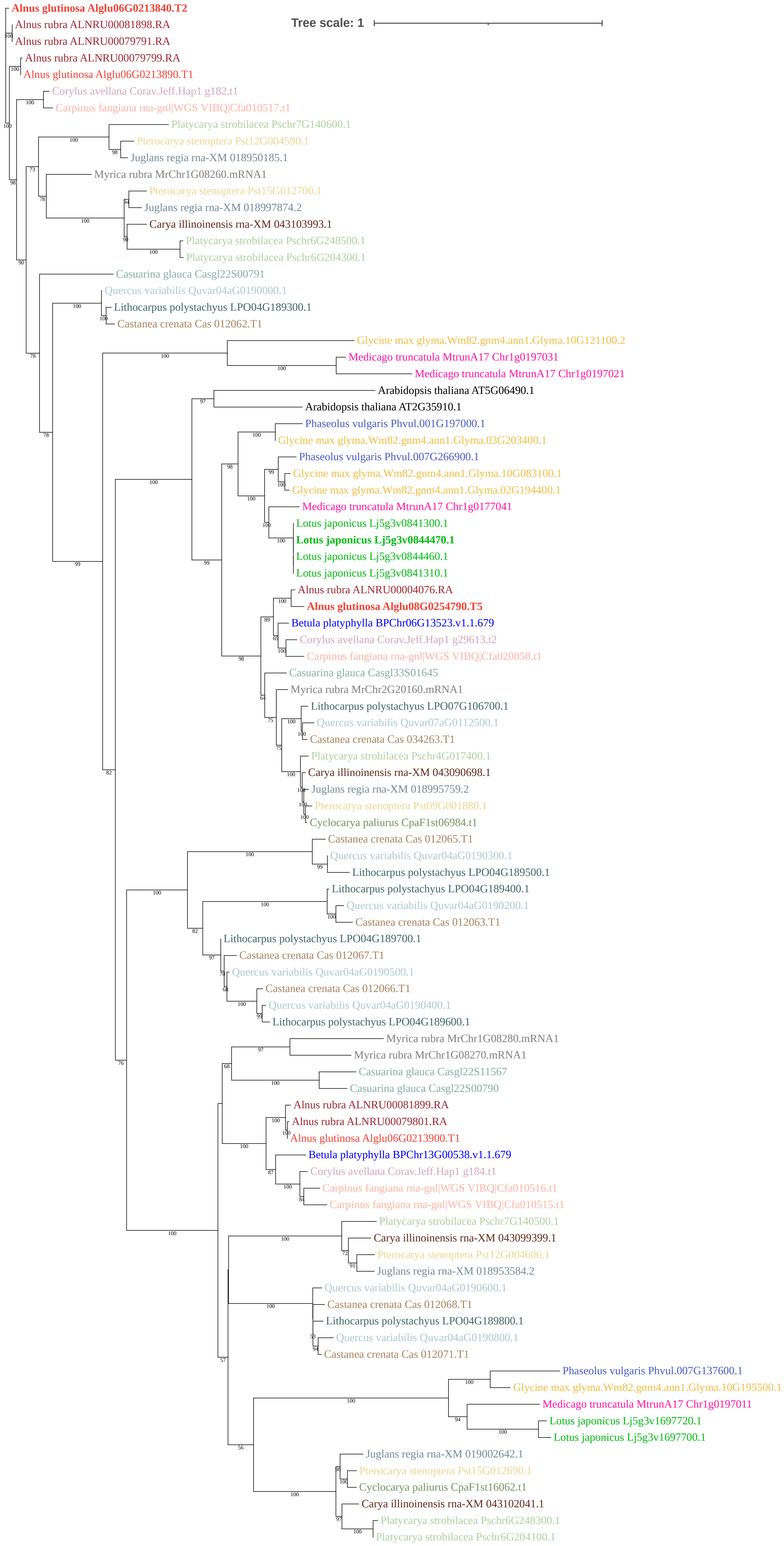

OG0000603:novel pectate lyase enzyme a/nodulation pectate lyase

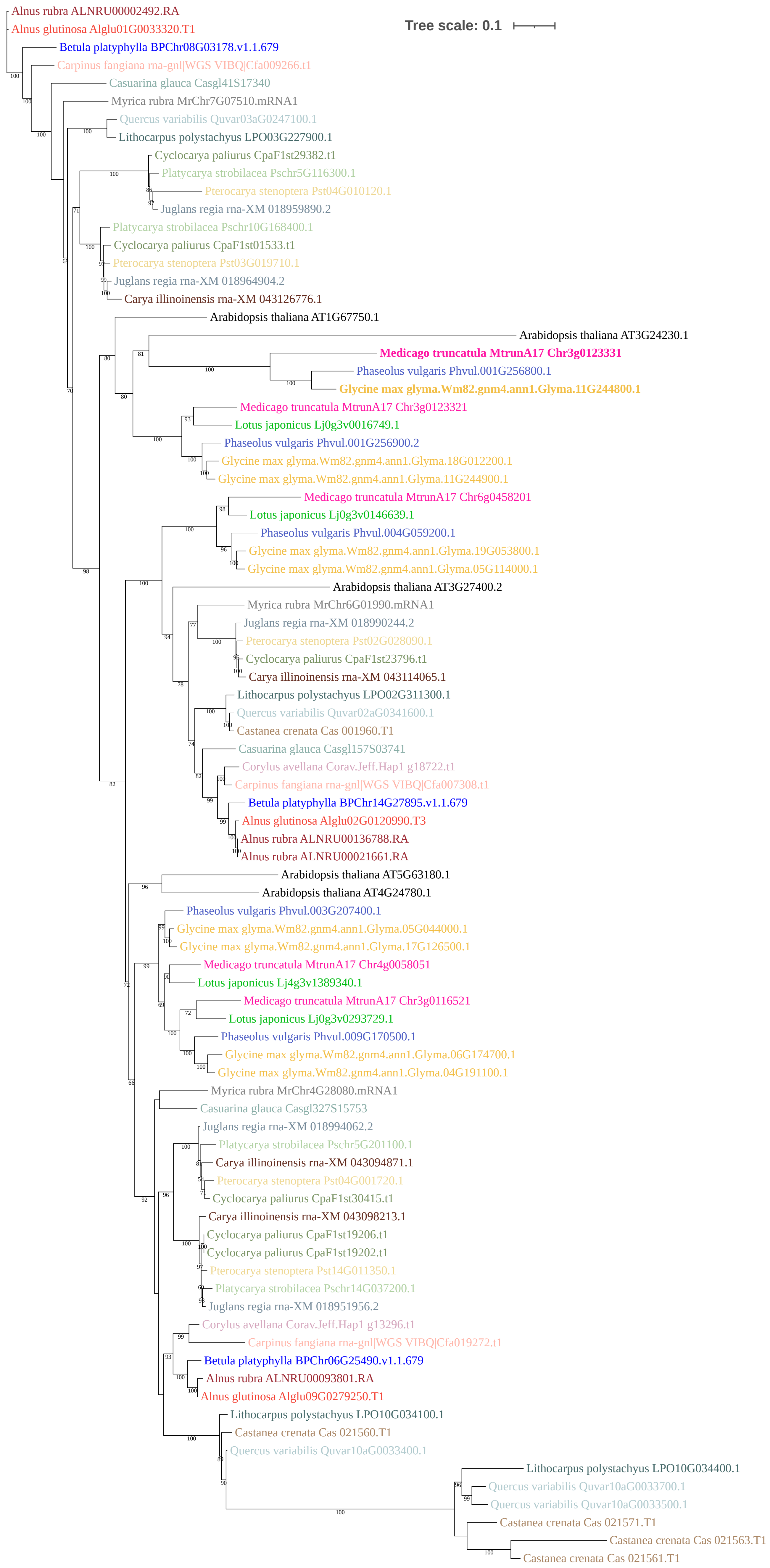

OG0000648:AUXIN RESPONSE FACTOR 16a

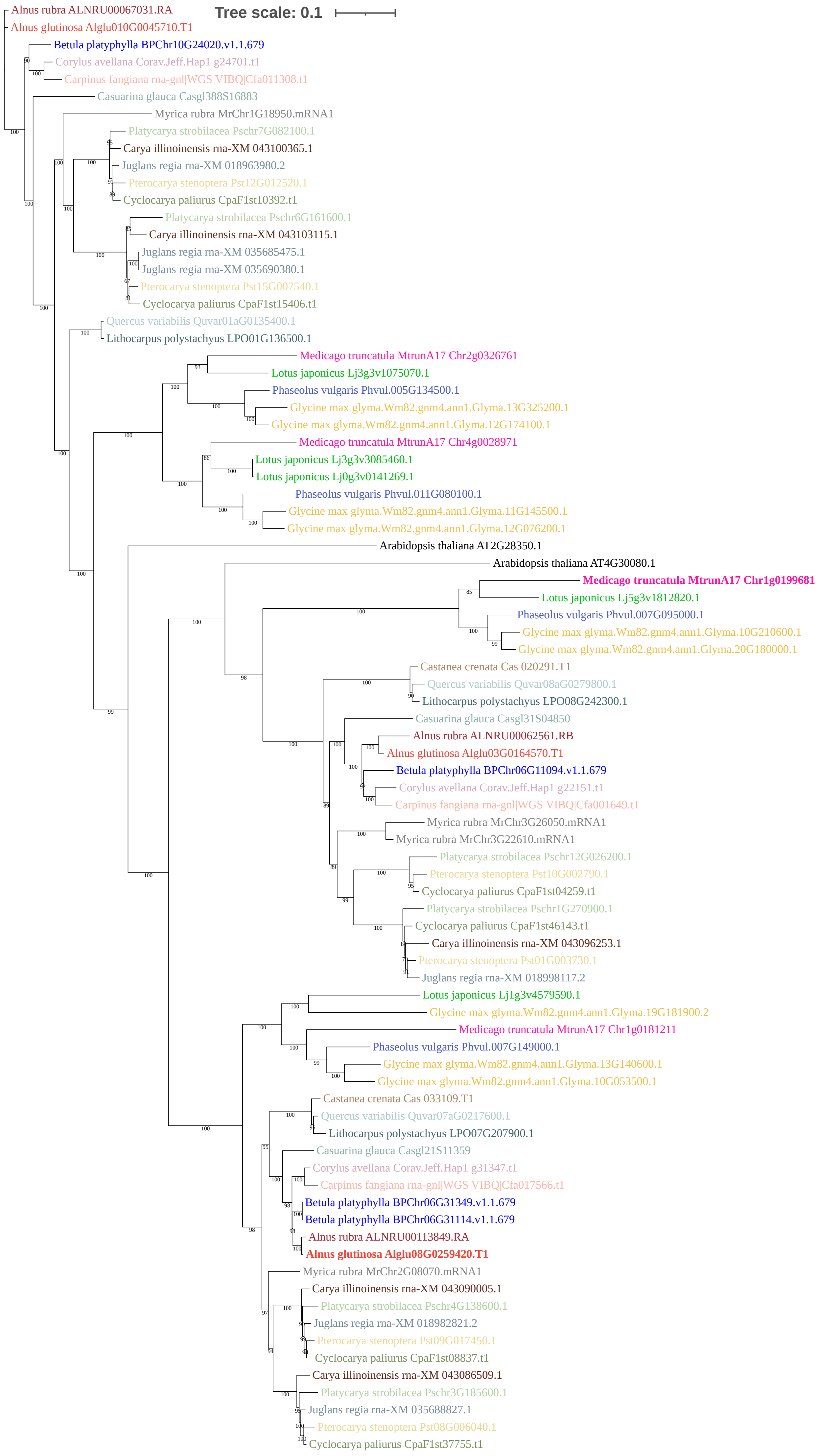

OG0000649:TONOPLAST INTRINSIC PROTEIN 1g

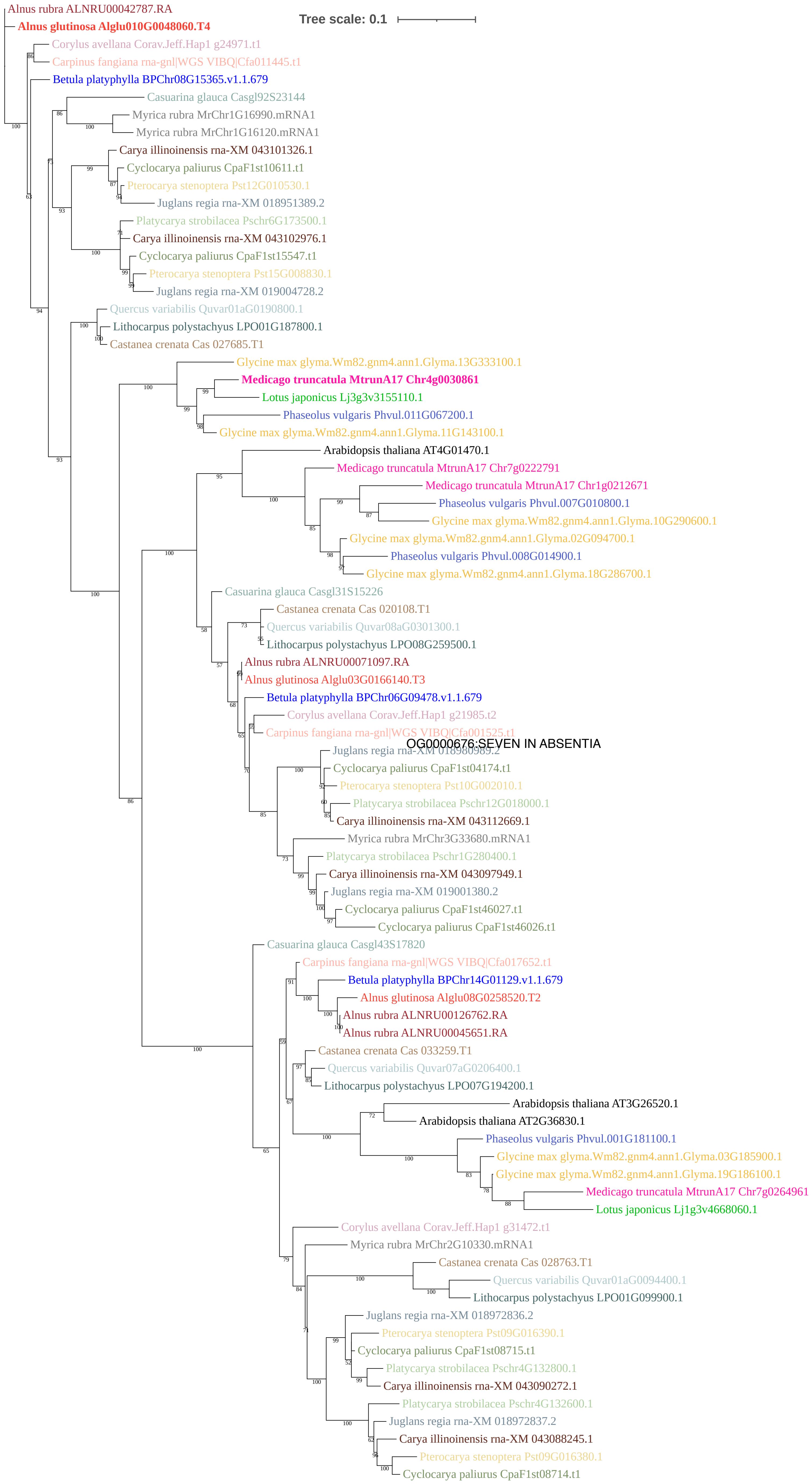

OG0000676:SEVEN IN ABSENTIA

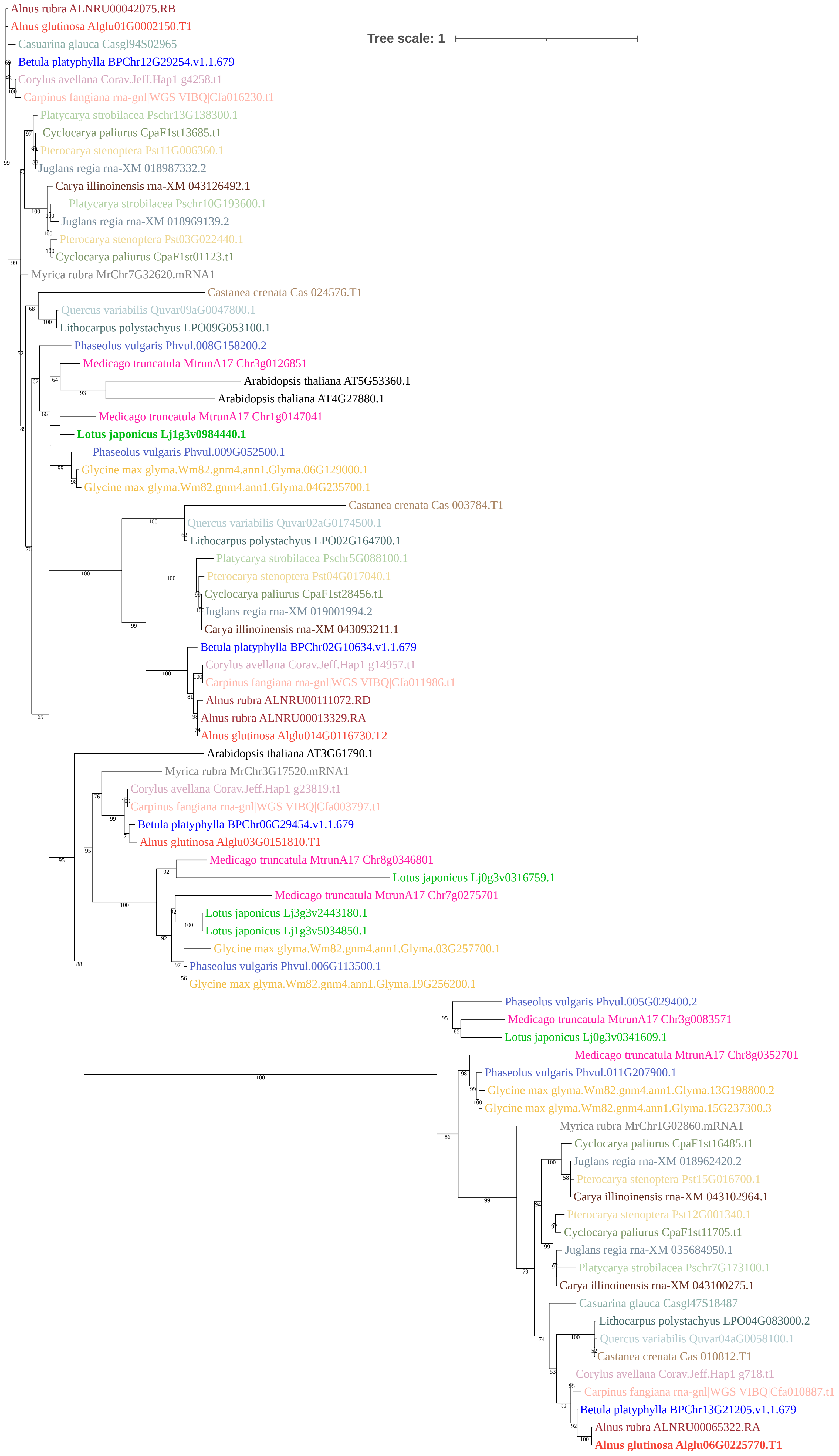

OG0000680:RESPIRATORY BURST OXIDASE HOMOLOG A/G

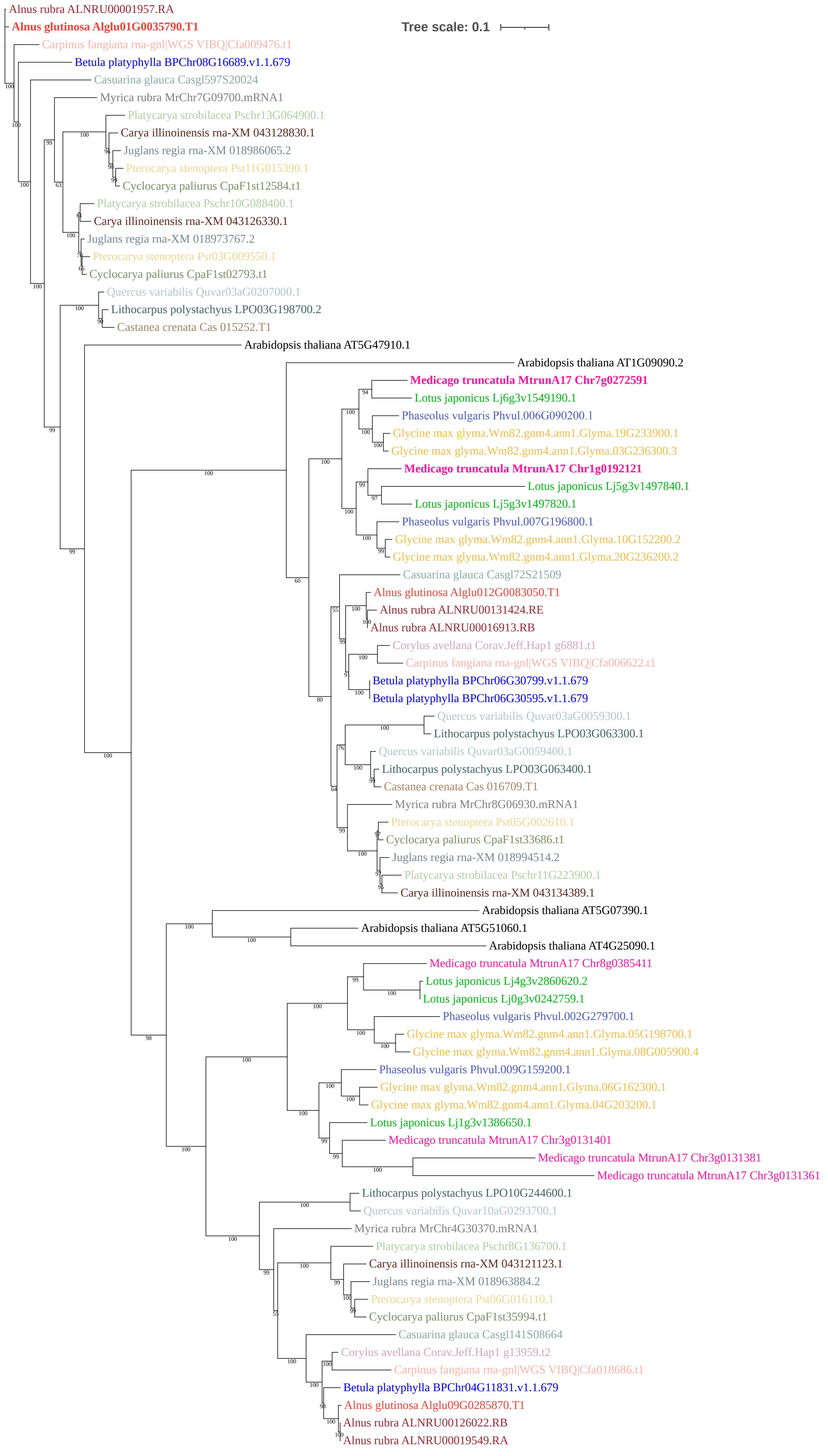

OG0000684:RHIZOBIUM-INDUCED PEROXIDASE 1|peroxidase 29

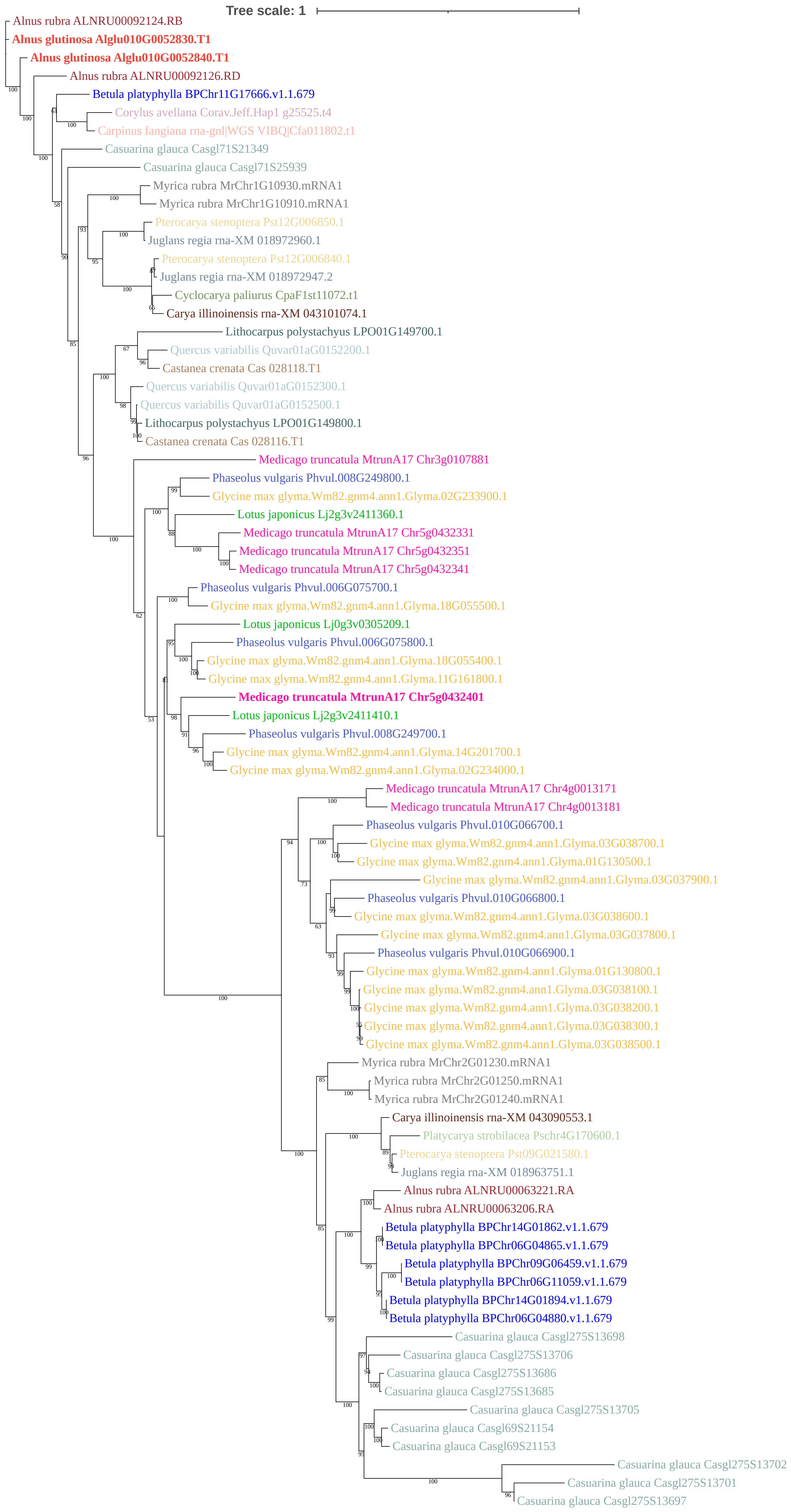

OG0000687:3-deoxy-d-arabino-heptulosonate 7-phosphate synthase 1

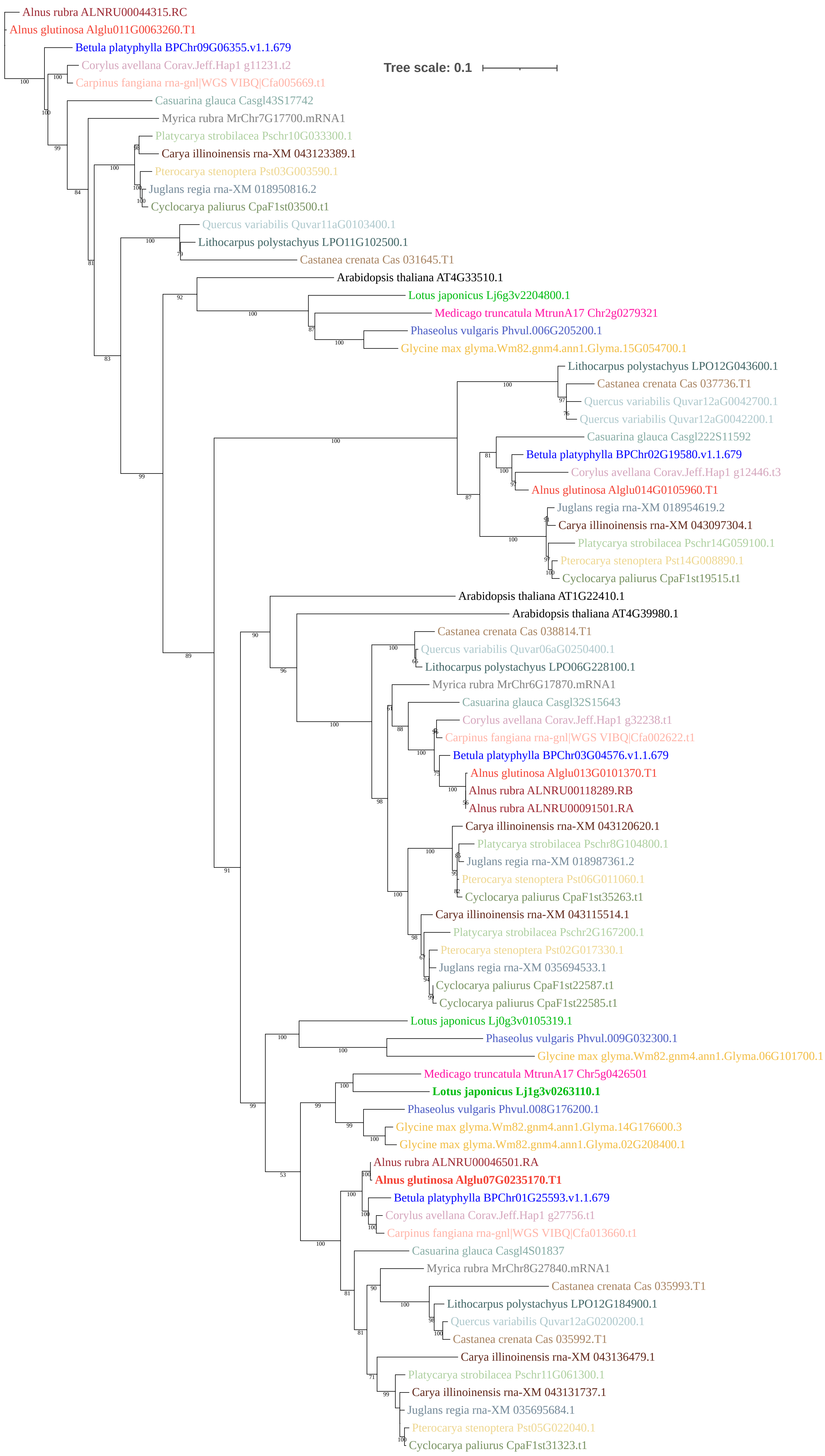

OG0000722:Rab(Ras-related proteins in brain) GTPases RabA2

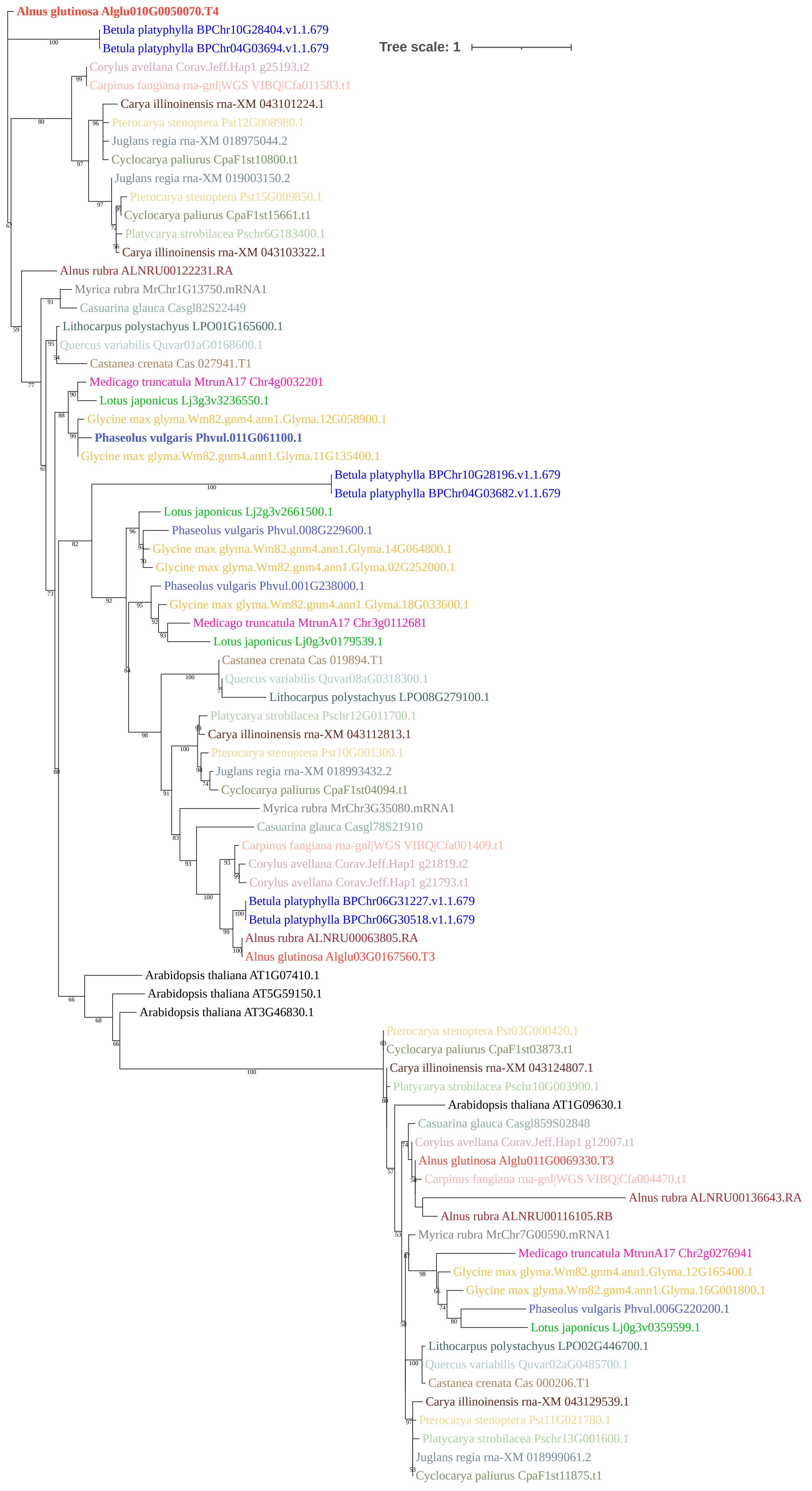

OG0000724:CYTOKININ OXIDASE/DEHYDROGENASE 3

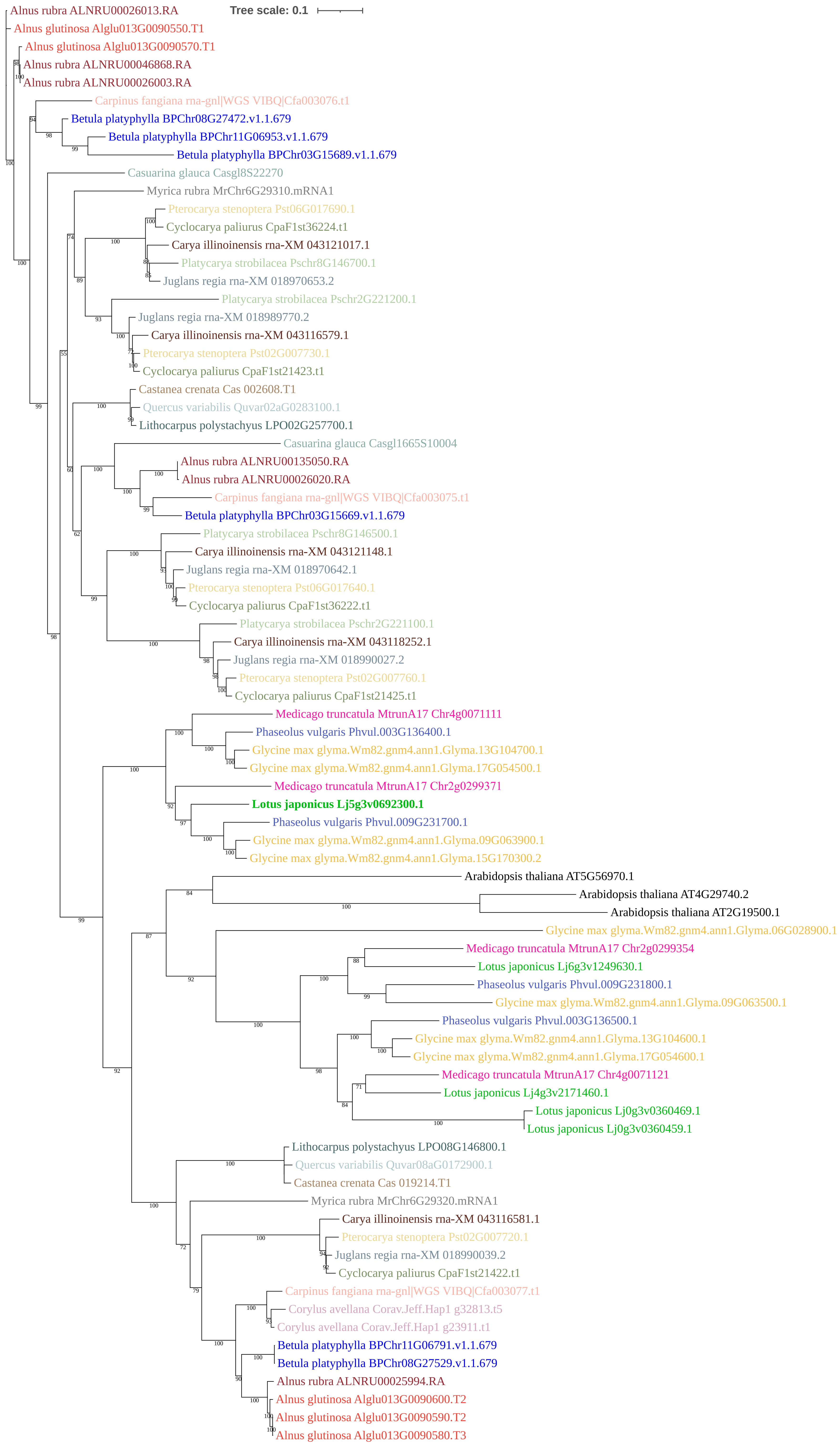

OG0000769:NUCLEOLAR/MITOCHONDRIAL PROTEIN INVOLVED IN NODULATION

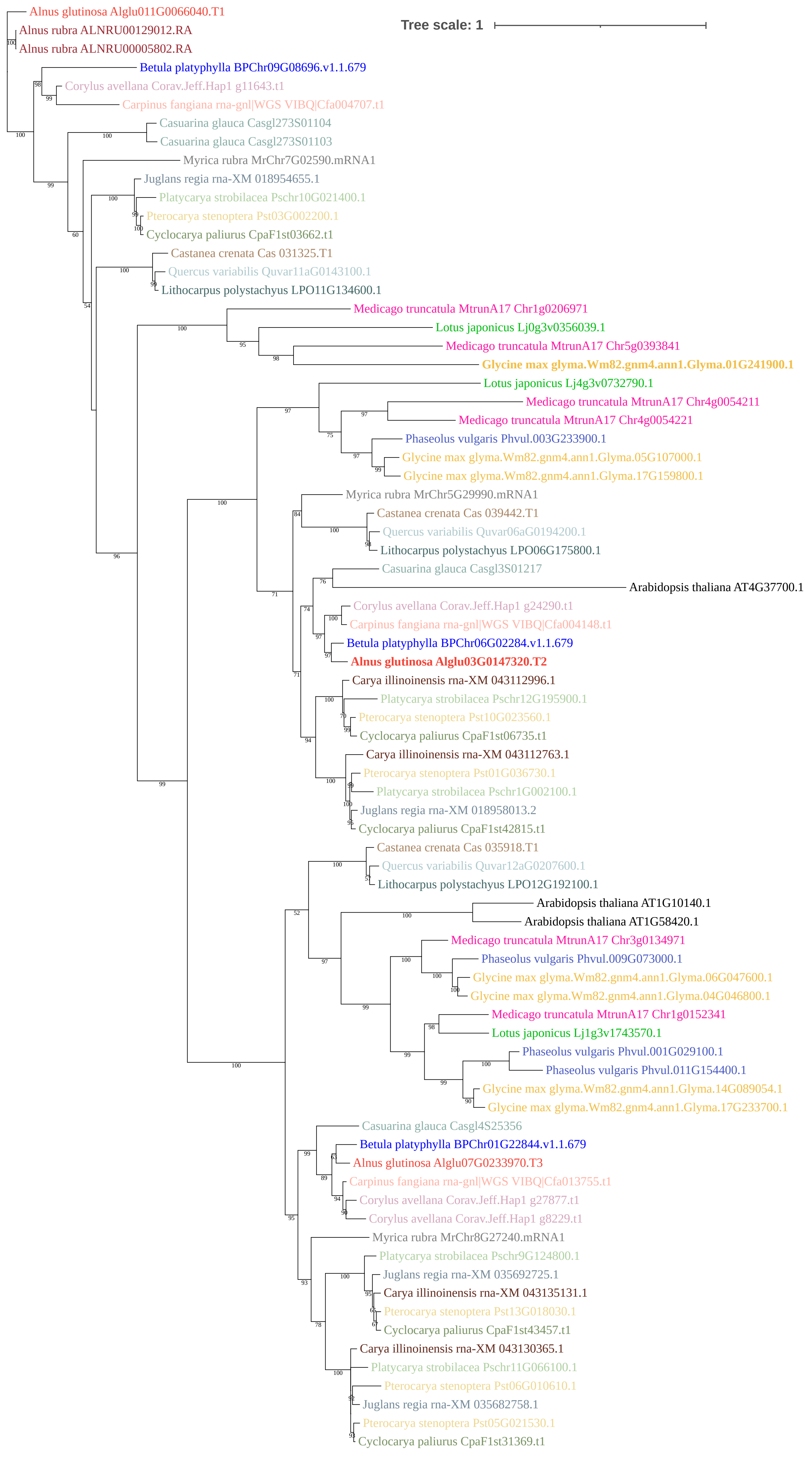

OG0000790:Symbiotic Protein Kinase 1

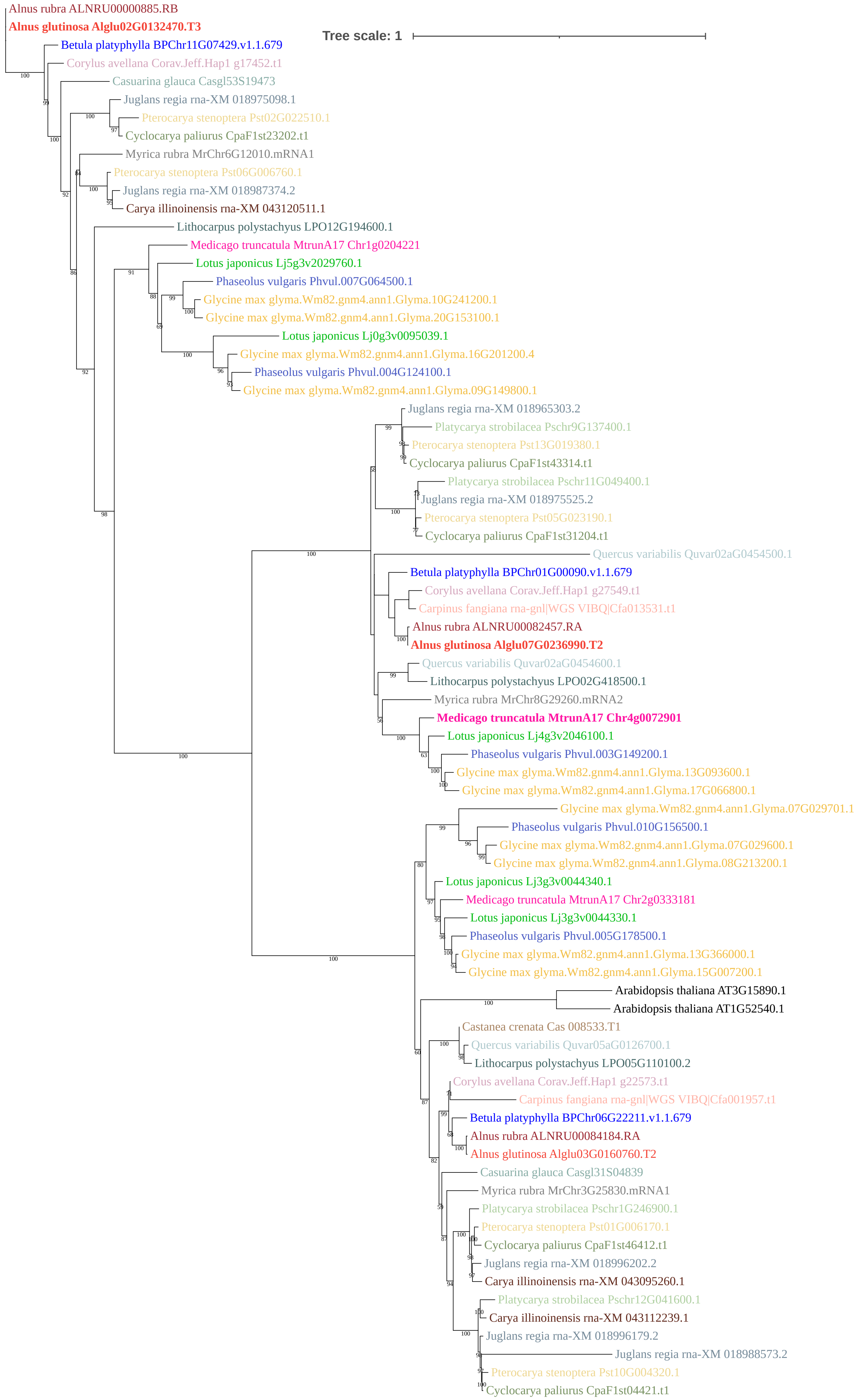

OG0000799:VESICLE ASSOCIATED MEMBRANE PROTEIN

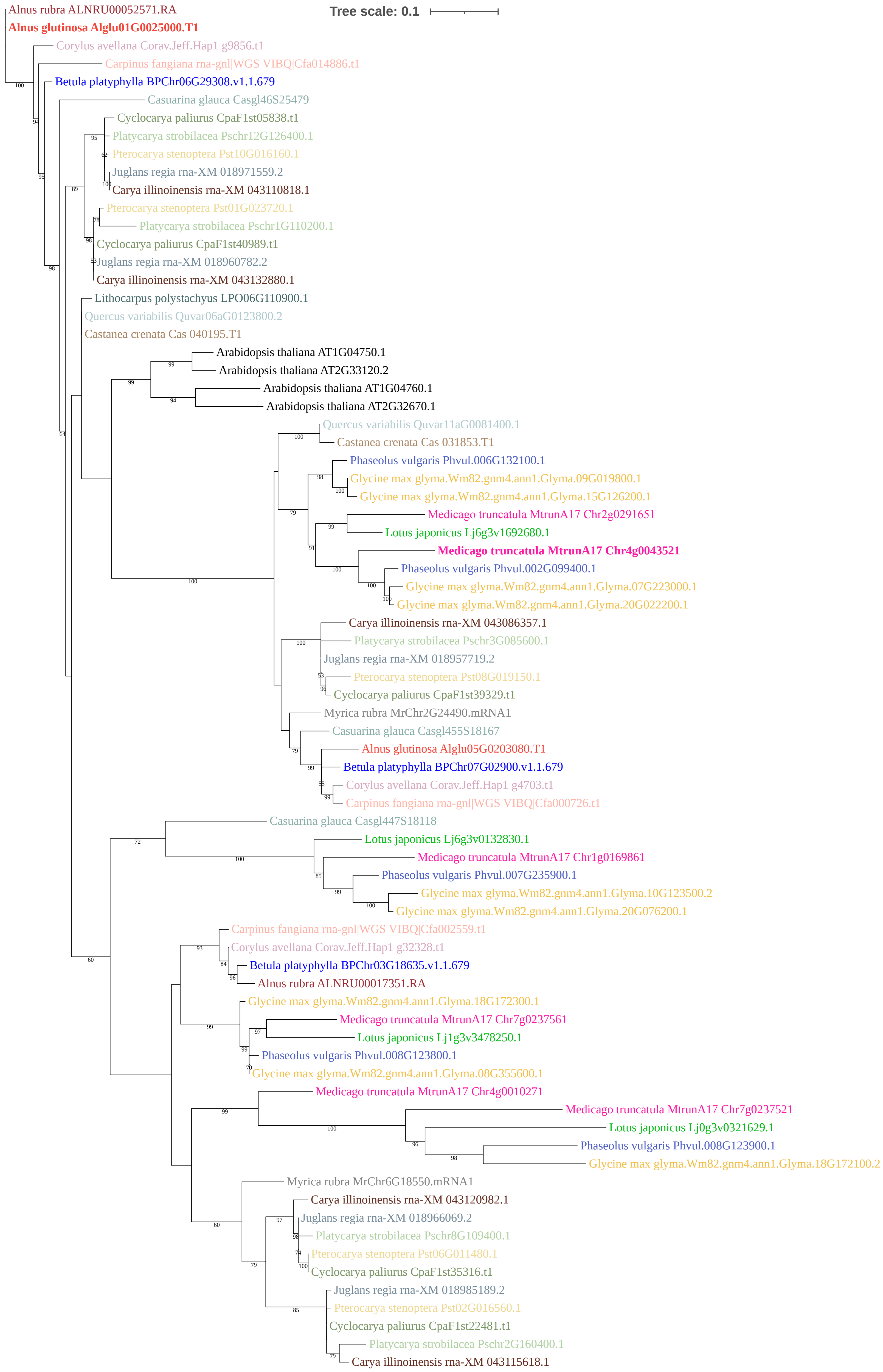

OG0000807:NOD FACTOR RECEPTOR 1/LysM domain-containing receptor-like kinase 3

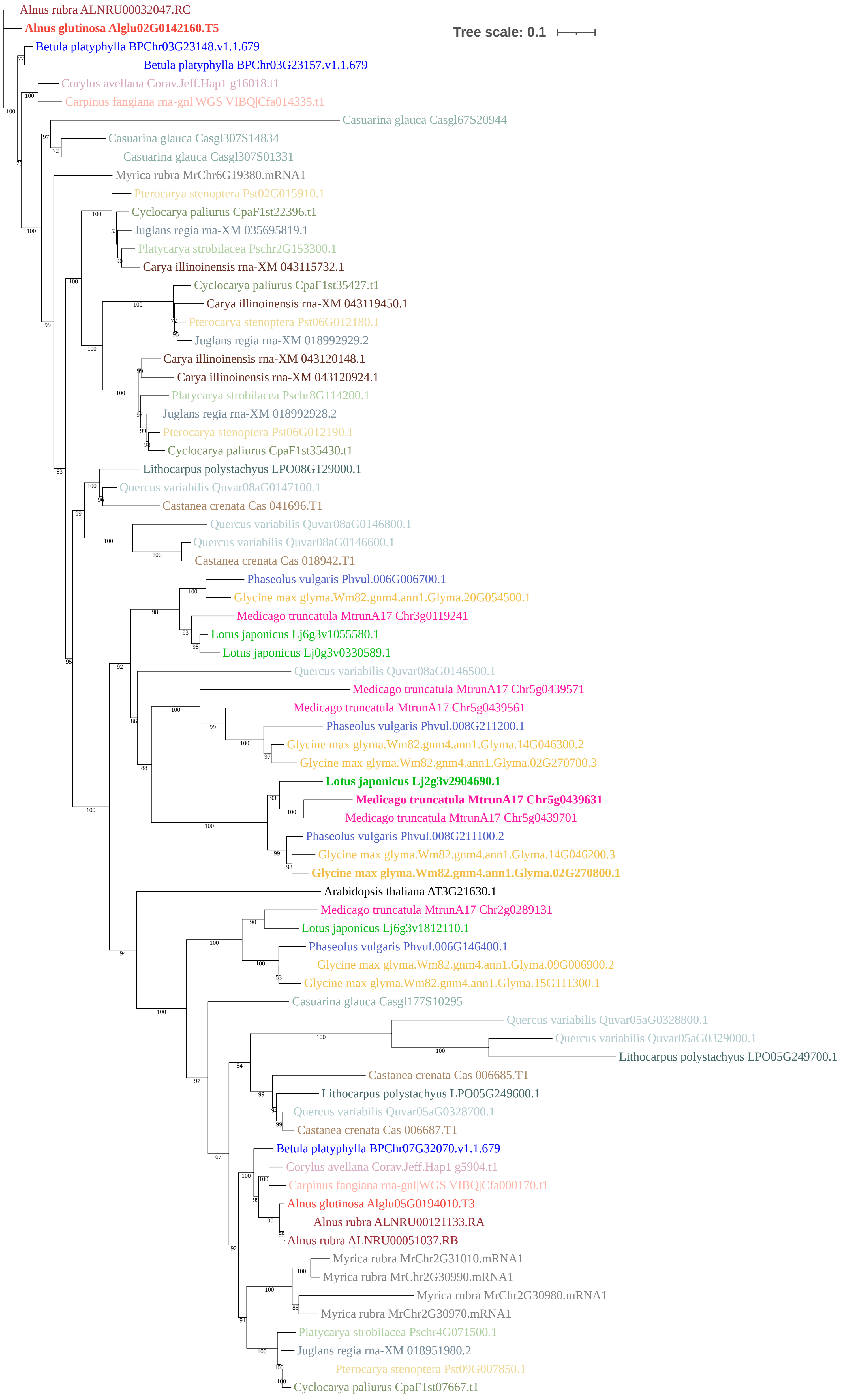

OG0000827:LEGHEMOGLOBIN/OG0000827:non-symbiotic hemoglobin

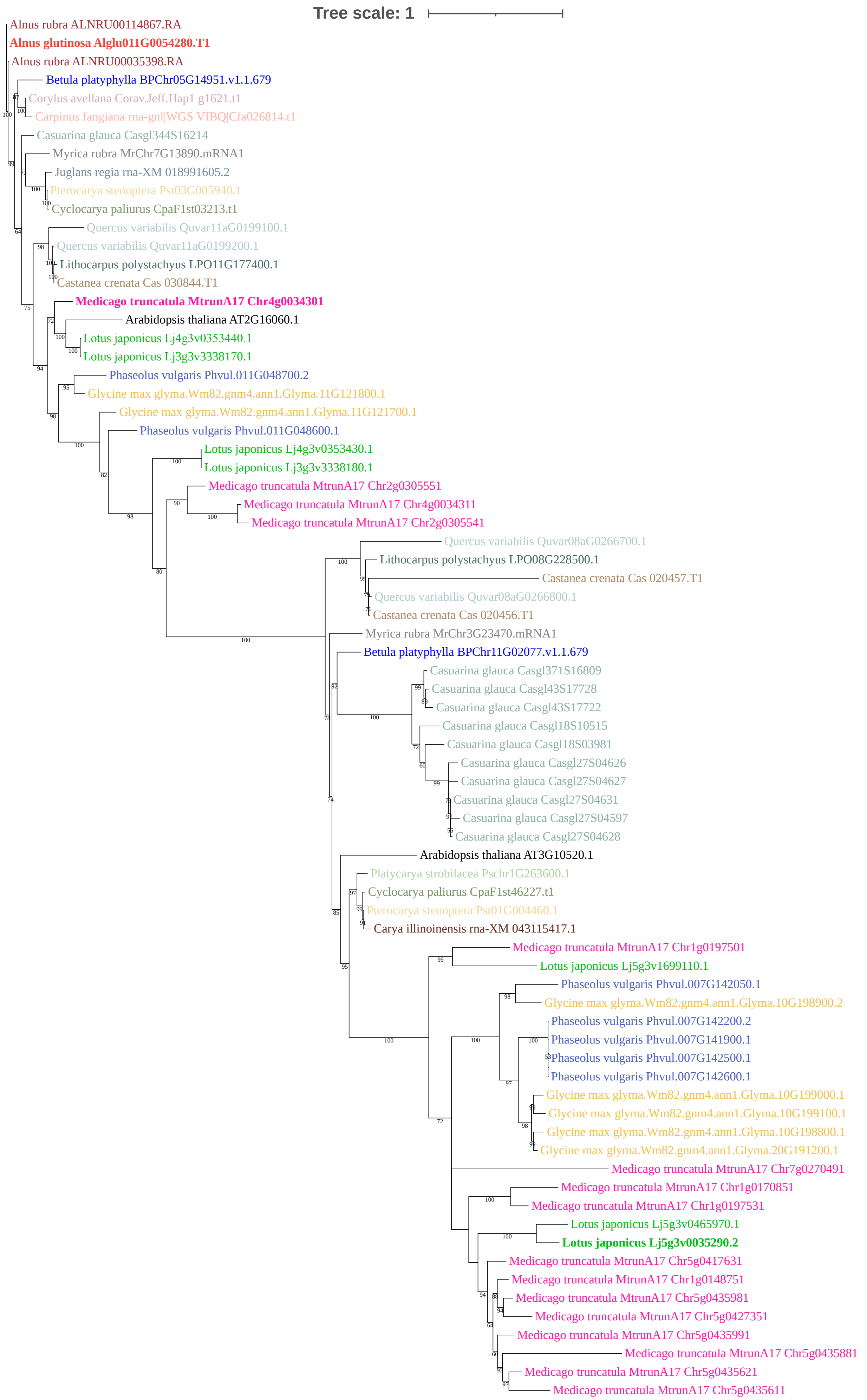

OG0000883:CHITINASE 5/Nod factor hydrolase 1

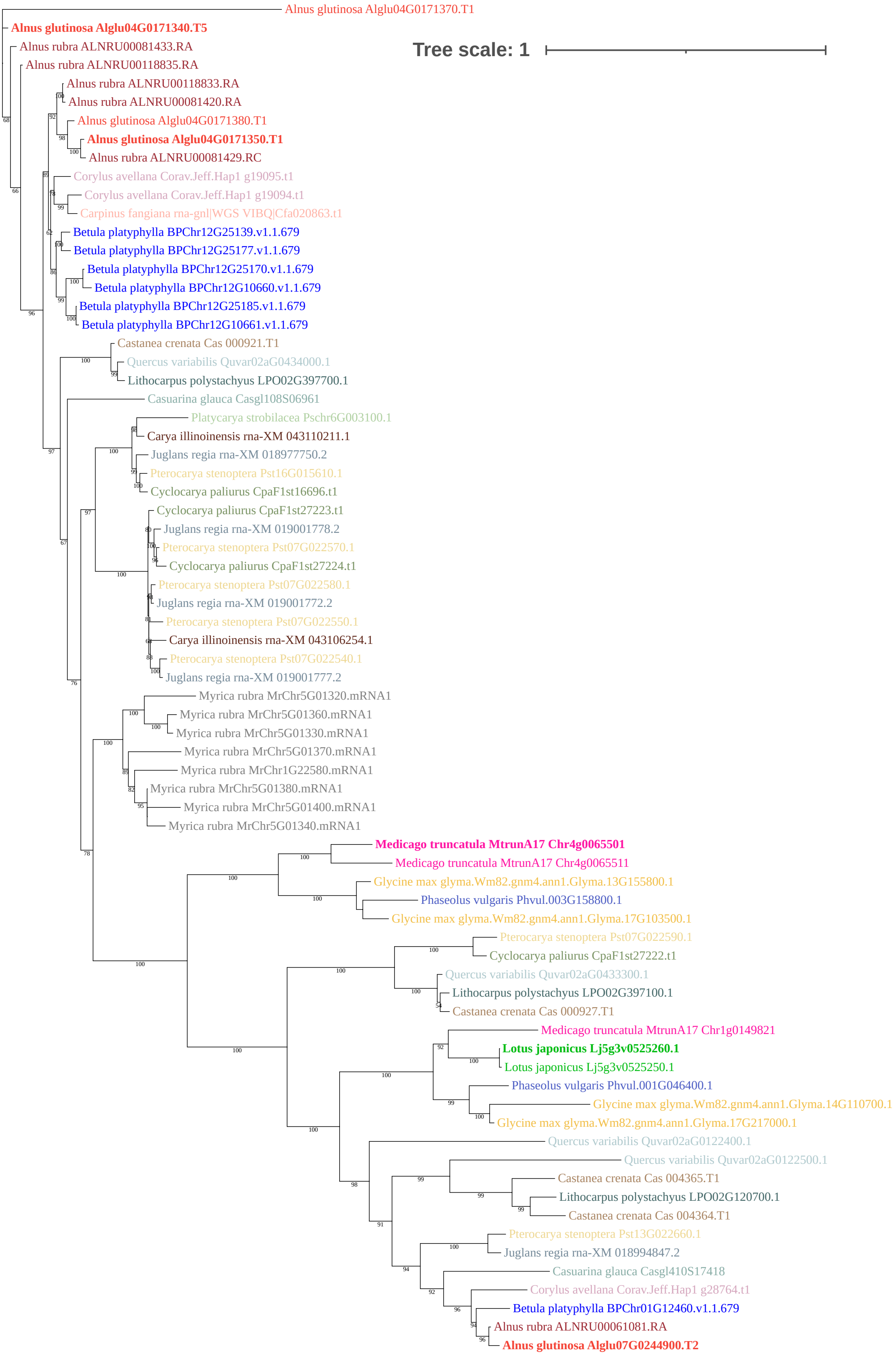

OG0000901:SYNTAXIN OF PLANTS 71

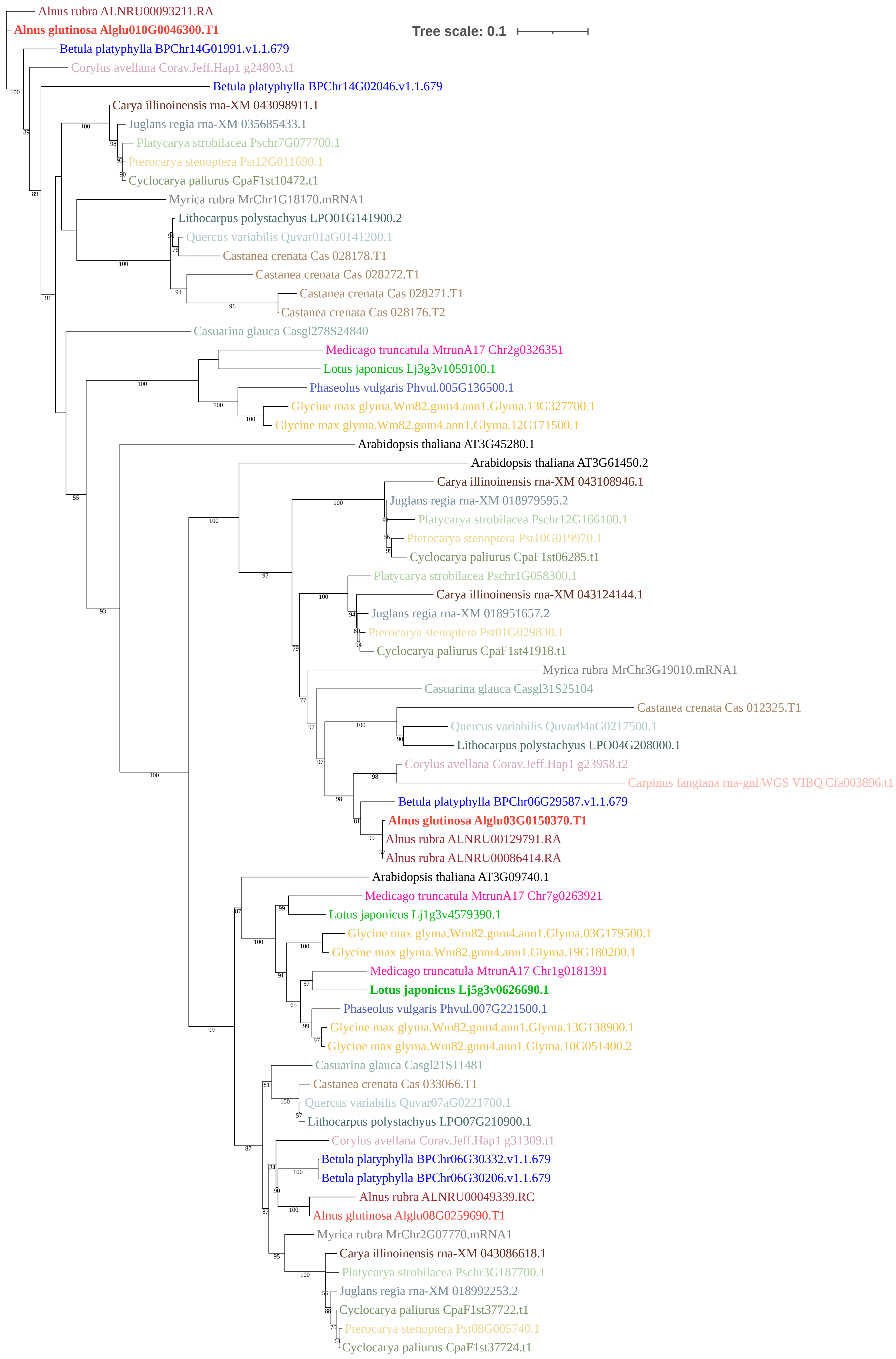

OG0000924:3-HYDROXY-3-METHYLGLUTARYL COENZYME A REDUCTASE 1

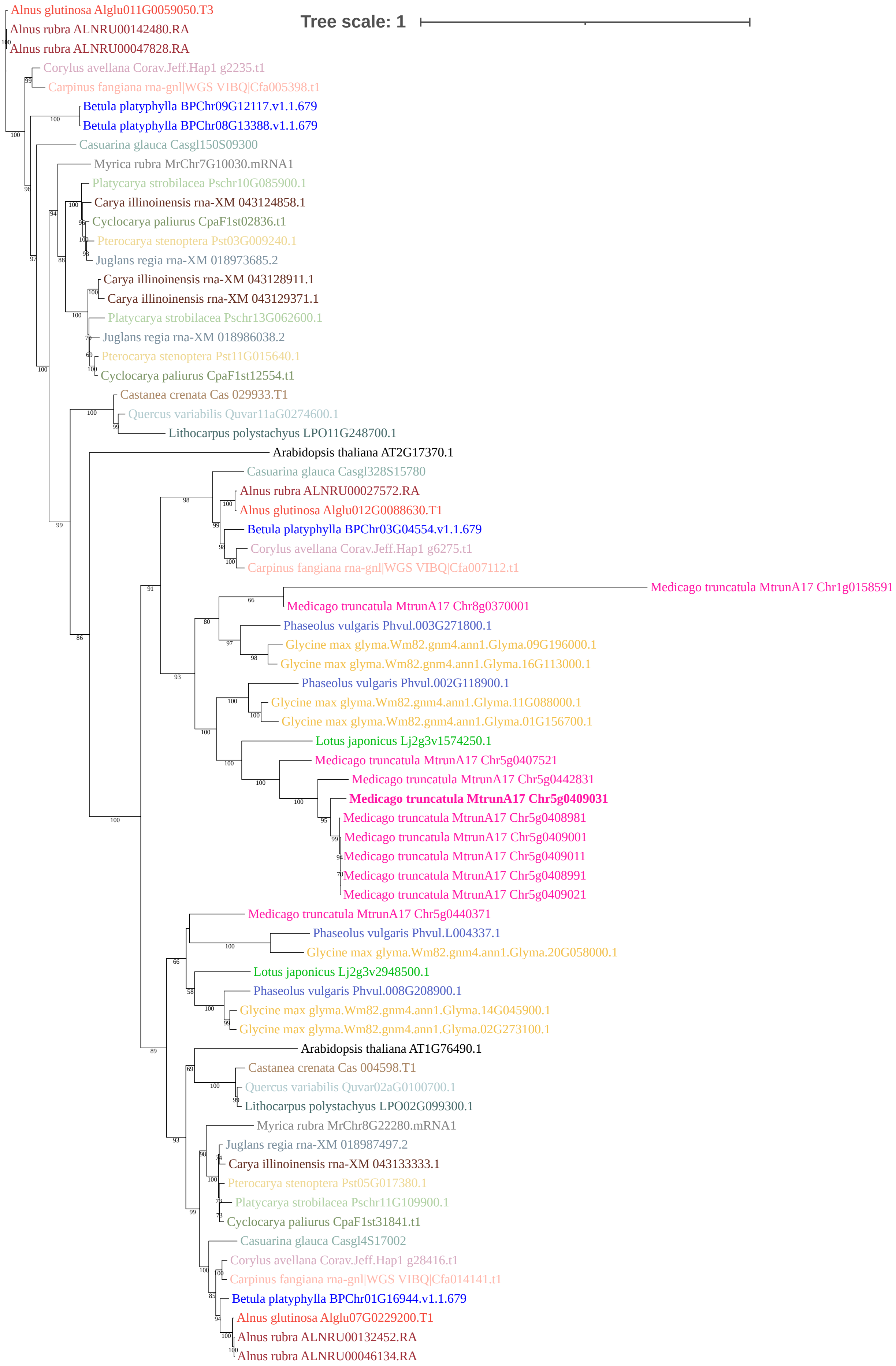

OG0000937:isopentenyl transferase 4/3

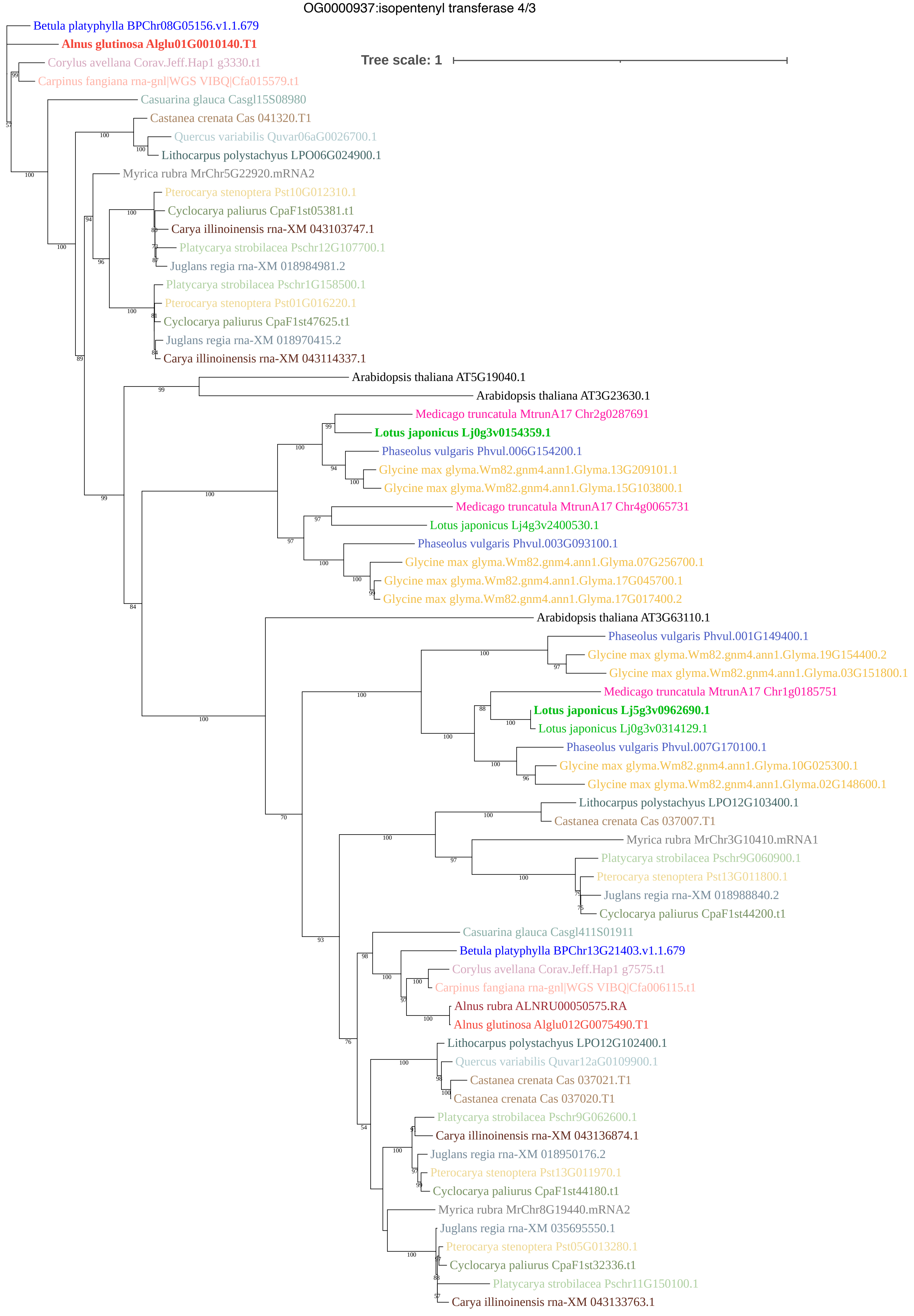

OG0000954:ETHYLENE RESPONSE FACTOR REQUIRED FOR NODULE DIFFERENTIATION

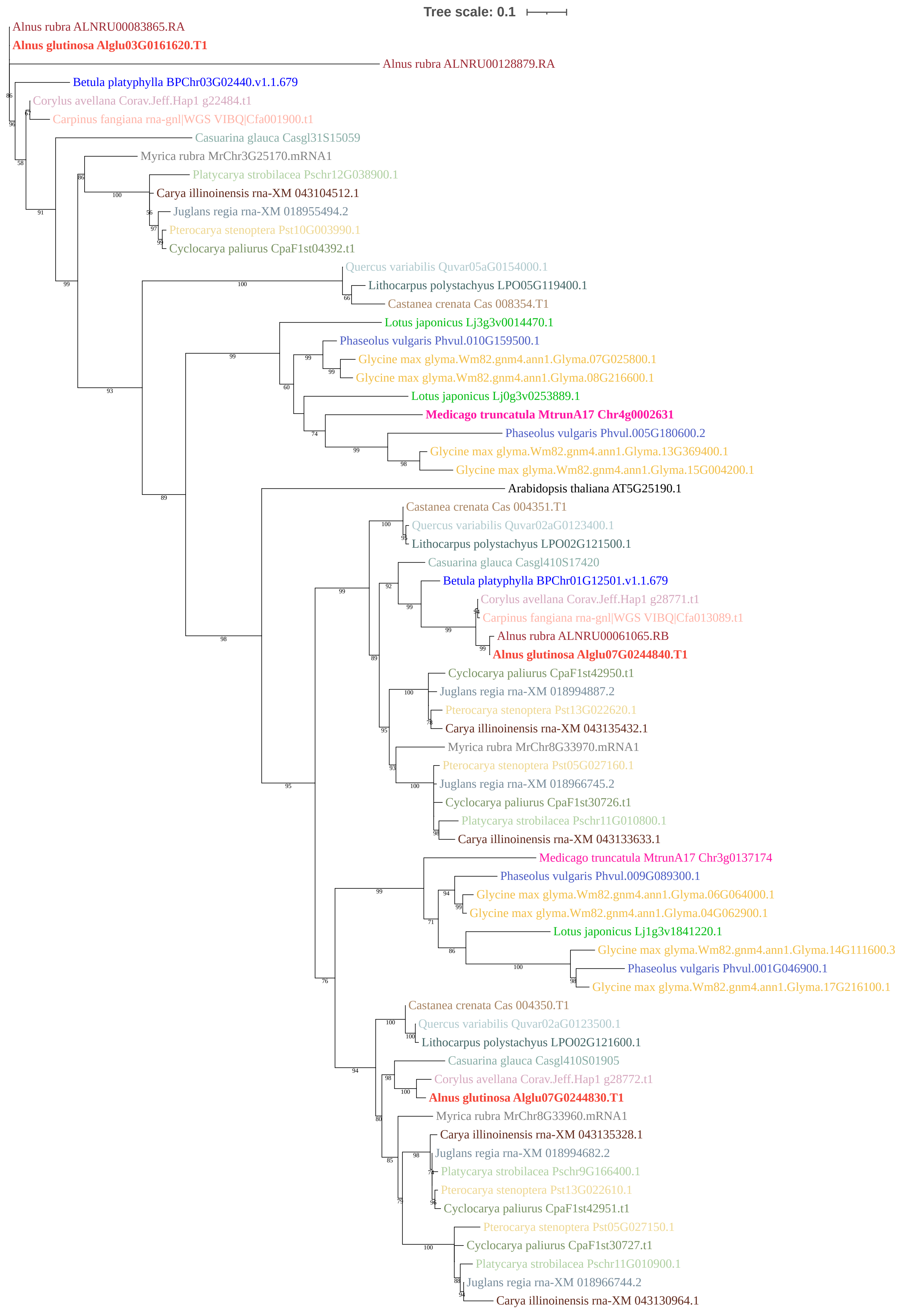

OG0000979:calcium-dependent protein kinase3

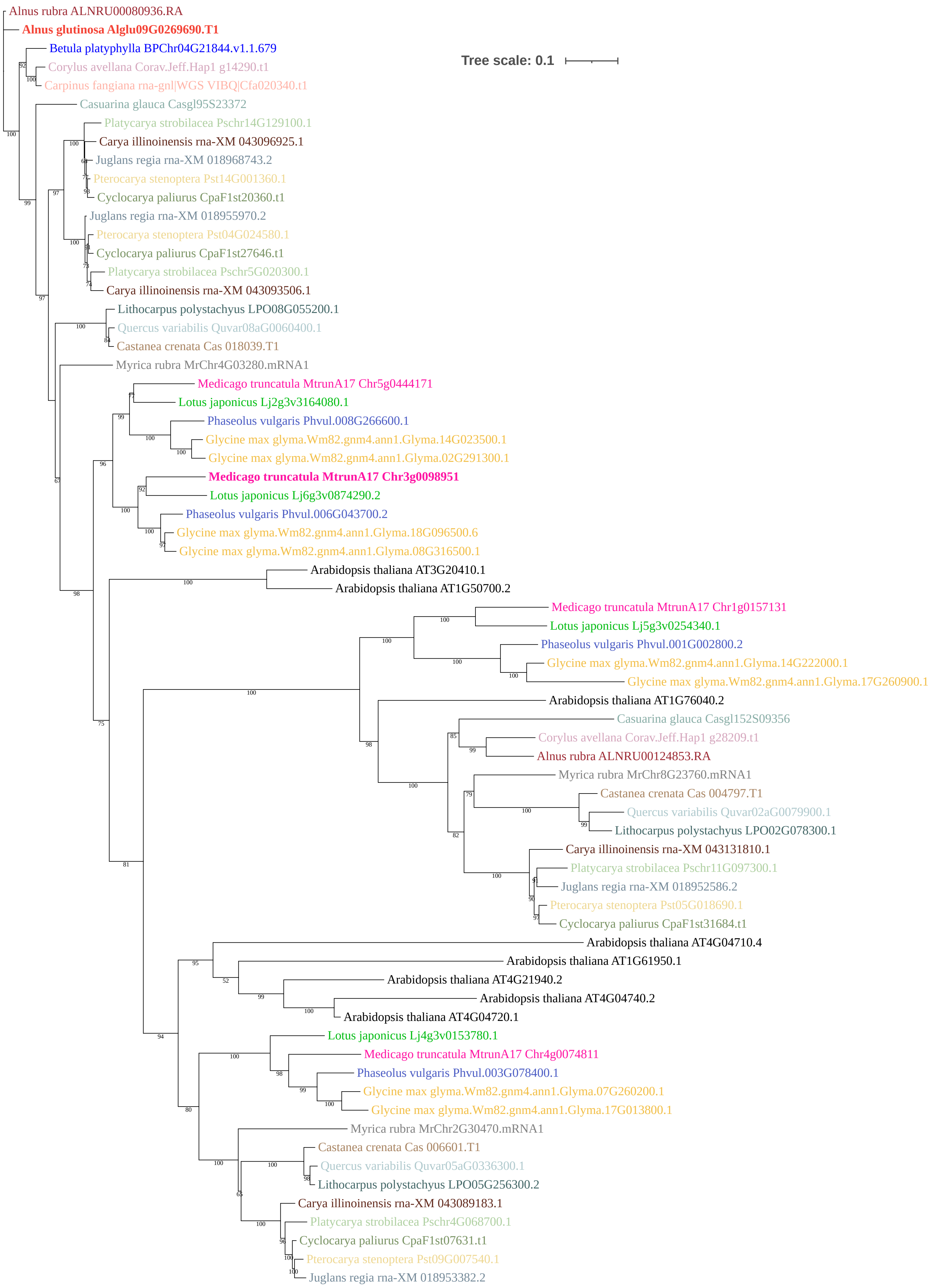

OG0001100:BRUSH

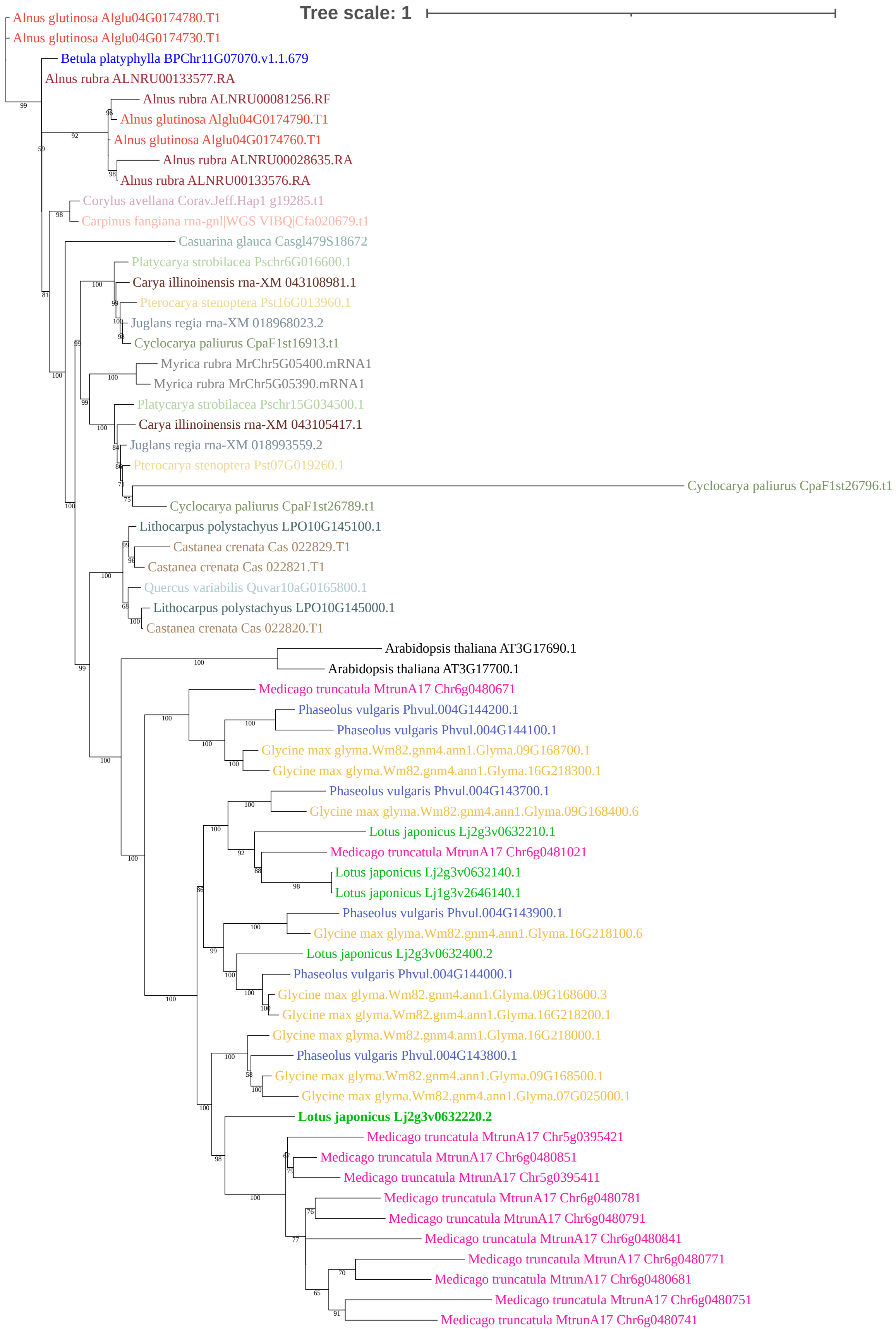

OG0001119:cytosolic glutamine synthetase

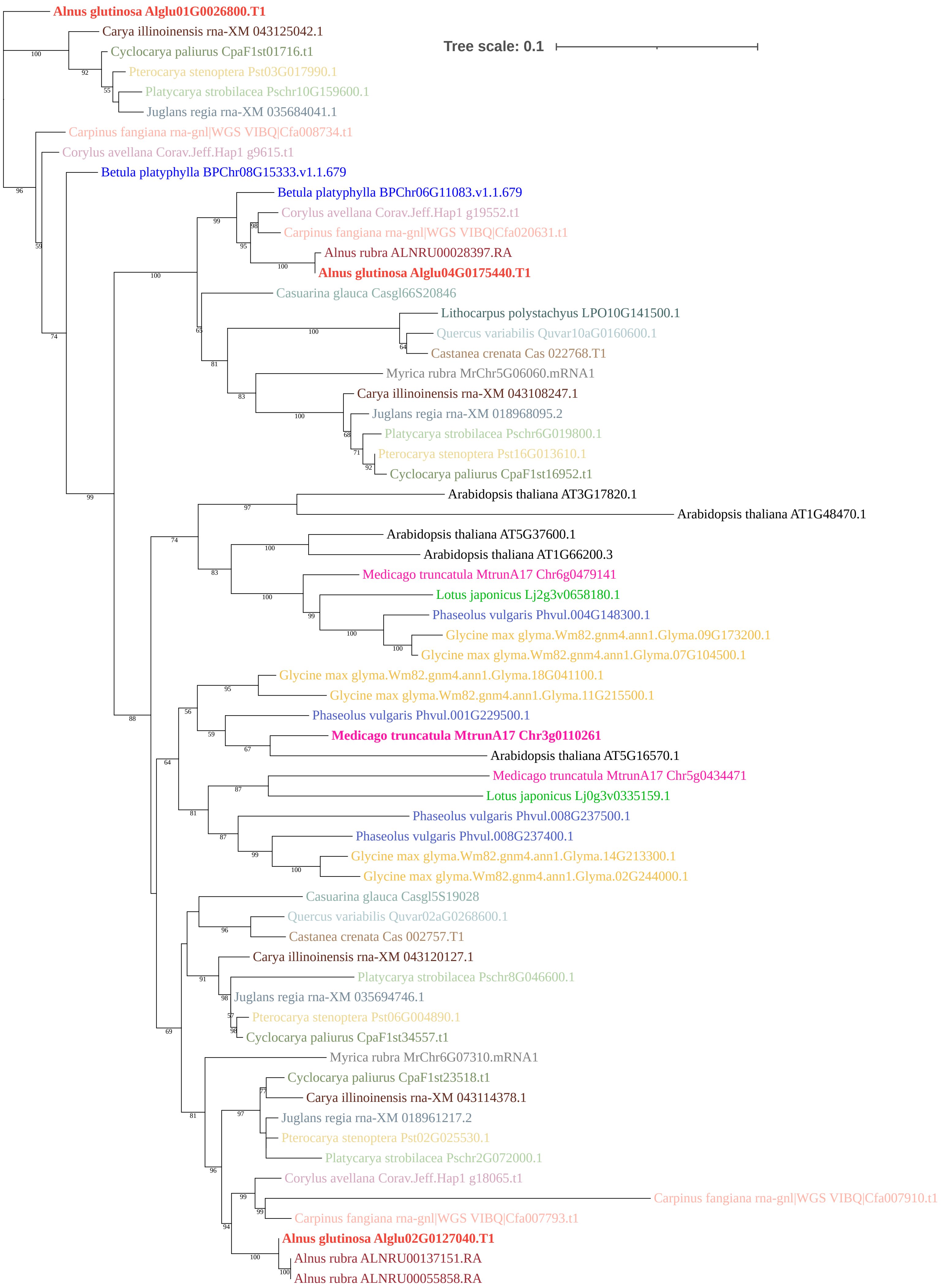

OG0001175:FRUIT WEIGHT 2.2-LIKE 1

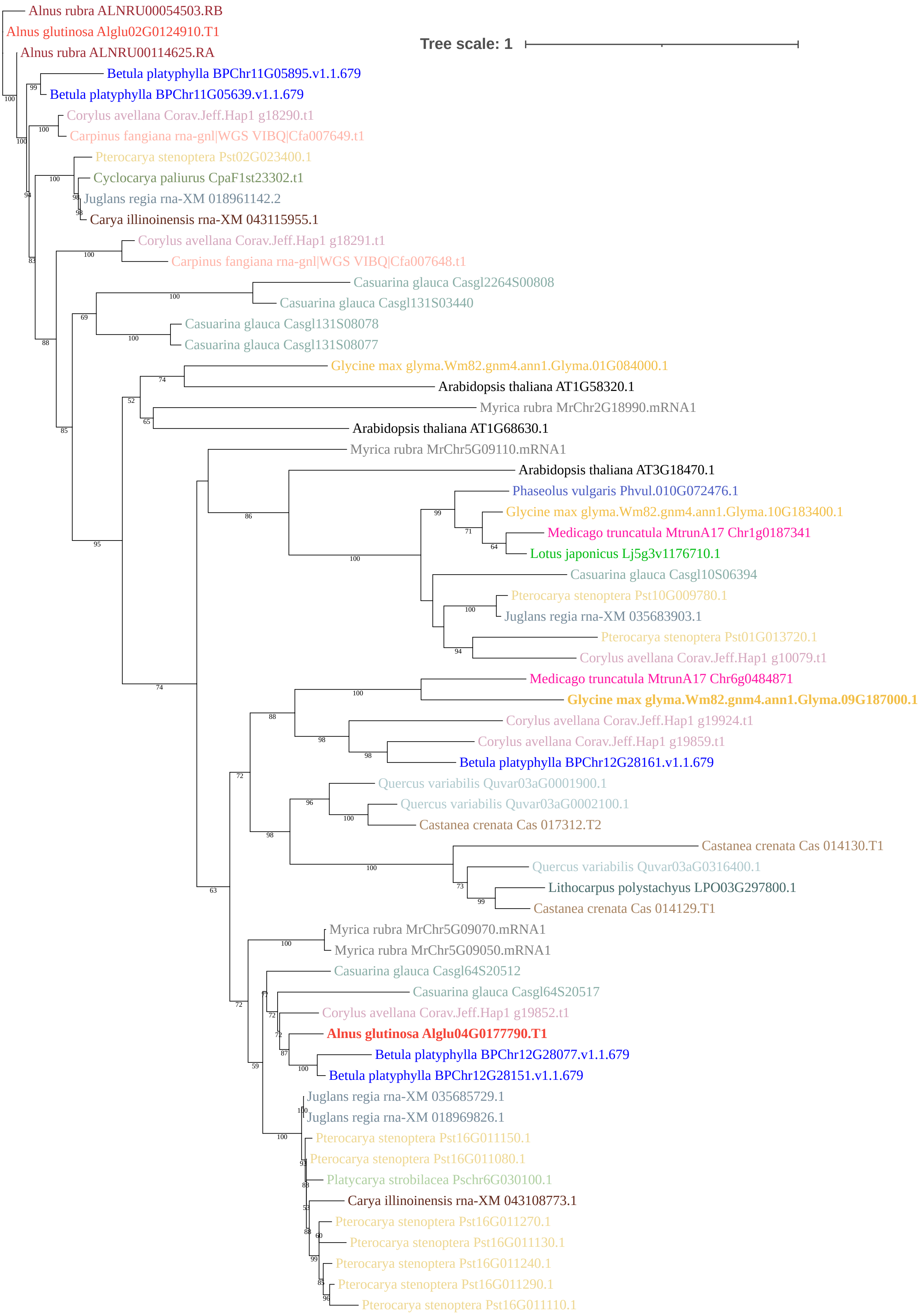

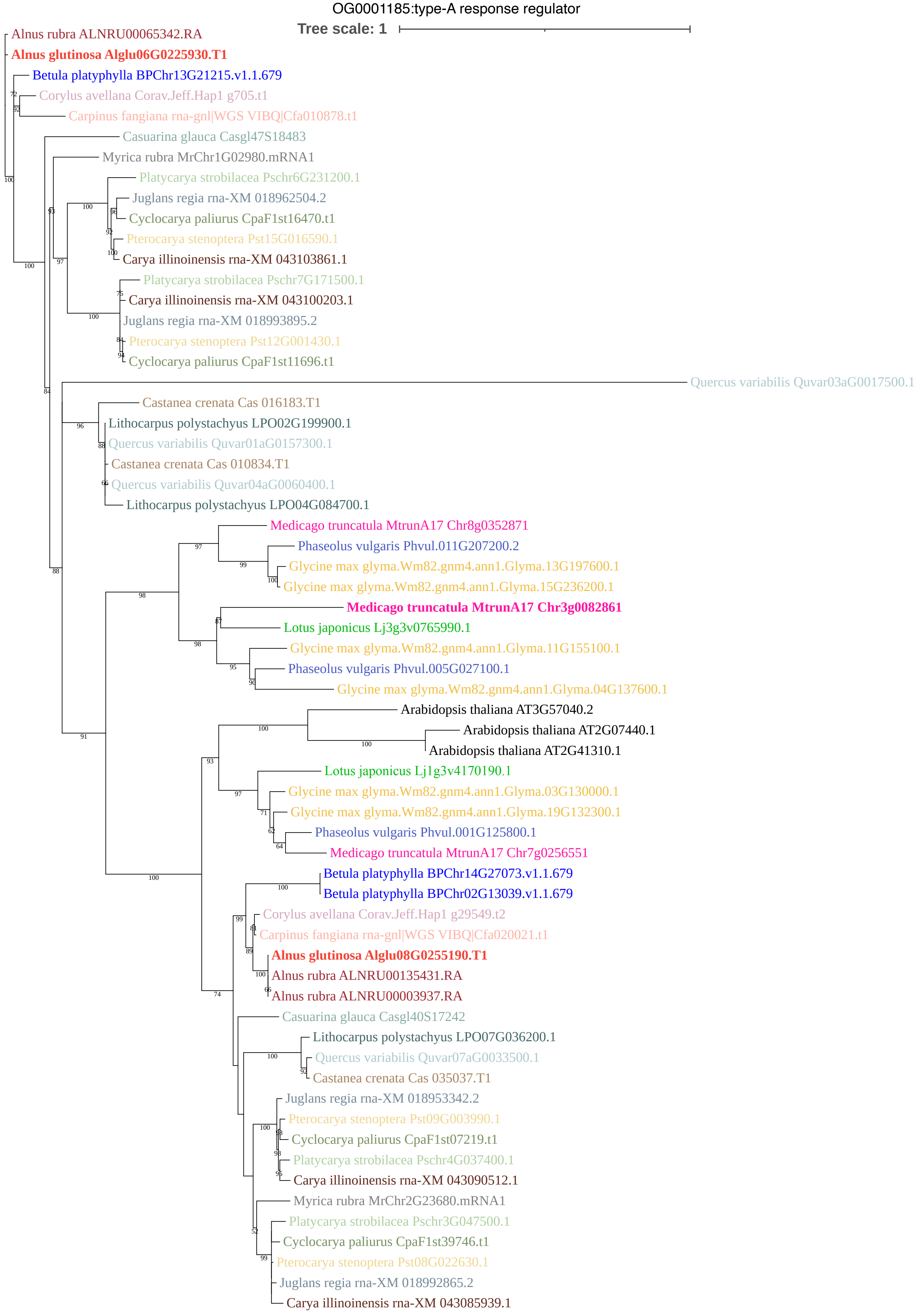
OG0001185:type-A response regulator
Tree scale: 1
Alnus rubra ALNRU00065342.RA
Alnus glutinosa Alglu06G0225930.T1
Betula platyphylla BPChr13G21215.v1.1.679
Corylus avellana Corav.Jeff.Hap1 g705.t1
Carpinus fangiana rna-gnl|WGS VIBQ|Cfa010878.t1
Casuarina glauca Casgl47S18483
Myrica rubra MrChr1G02980.mRNA1
Platycarya strobilacea Pschr6G231200.1
Juglans regia rna-XM 018962504.2
Cyclocarya paliurus CpaF1st16470.t1
Pterocarya stenoptera Pst15G016590.1
Carya illinoinensis rna-XM 043103861.1
Platycarya strobilacea Pschr7G171500.1
Carya illinoinensis rna-XM 043100203.1
Juglans regia rna-XM 018993895.2
Pterocarya stenoptera Pst12G001430.1
Cyclocarya paliurus CpaF1st11696.t1
Quercus variabilis Quvar03aG0017500.1
Castanea crenata Cas 016183.T1
Lithocarpus polystachyus LPO02G199900.1
Quercus variabilis Quvar01aG0157300.1
Castanea crenata Cas 010834.T1
Quercus variabilis Quvar04aG0060400.1
Lithocarpus polystachyus LPO04G084700.1
Medicago truncatula MtrunA17 Chr8g0352871
Phaseolus vulgaris Phvul.011G207200.2
Glycine max glyma.Wm82.gnm4.ann1.Glyma.13G197600.1
Glycine max glyma.Wm82.gnm4.ann1.Glyma.15G236200.1
Medicago truncatula MtrunA17 Chr3g0082861
Lotus japonicus Lj3g3v0765990.1
Glycine max glyma.Wm82.gnm4.ann1.Glyma.11G155100.1
Phaseolus vulgaris Phvul.005G027100.1
Glycine max glyma.Wm82.gnm4.ann1.Glyma.04G137600.1
Arabidopsis thaliana AT3G57040.2
Arabidopsis thaliana AT2G07440.1
Arabidopsis thaliana AT2G41310.1
Lotus japonicus Lj1g3v4170190.1
Glycine max glyma.Wm82.gnm4.ann1.Glyma.03G130000.1
Glycine max glyma.Wm82.gnm4.ann1.Glyma.19G132300.1
Phaseolus vulgaris Phvul.001G125800.1
Medicago truncatula MtrunA17 Chr7g0256551
Betula platyphylla BPChr14G27073.v1.1.679
Betula platyphylla BPChr02G13039.v1.1.679
Corylus avellana Corav.Jeff.Hap1 g29549.t2
Carpinus fangiana rna-gnl|WGS VIBQ|Cfa020021.t1
Alnus glutinosa Alglu08G0255190.T1
Alnus rubra ALNRU00135431.RA
Alnus rubra ALNRU00003937.RA
Casuarina glauca Casgl40S17242
Lithocarpus polystachyus LPO07G036200.1
Quercus variabilis Quvar07aG0033500.1
Castanea crenata Cas 035037.T1
Juglans regia rna-XM 018953342.2
Pterocarya stenoptera Pst09G003990.1
Cyclocarya paliurus CpaF1st07219.t1
Platycarya strobilacea Pschr4G037400.1
Carya illinoinensis rna-XM 043090512.1
Myrica rubra MrChr2G23680.mRNA1
Platycarya strobilacea Pschr3G047500.1
Cyclocarya paliurus CpaF1st39746.t1
Pterocarya stenoptera Pst08G022630.1
Juglans regia rna-XM 018992865.2
Carya illinoinensis rna-XM 043085939.1

OG0001206:NRT1/PTR Family

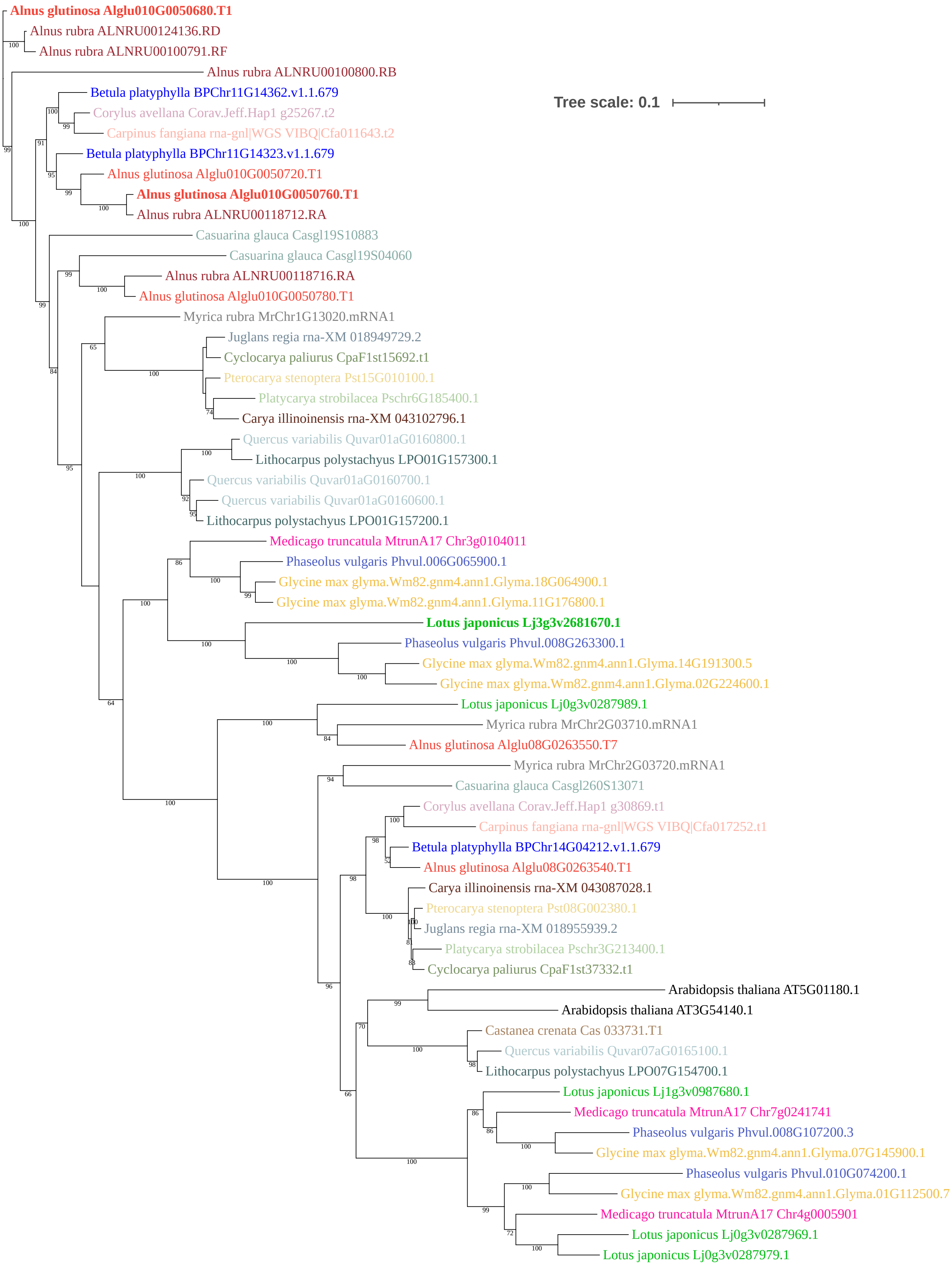

OG0001208:GRETCHEN HAGEN 3

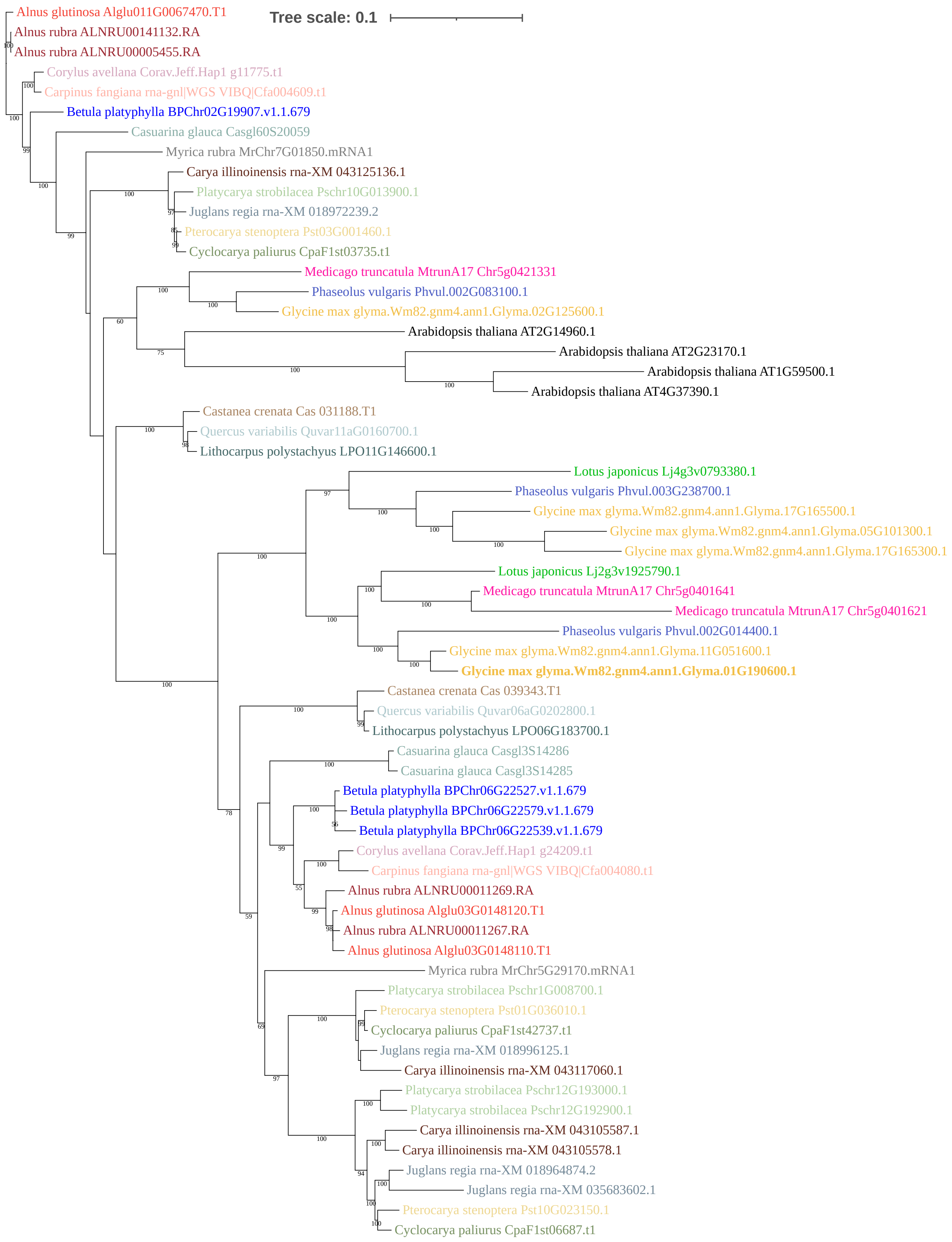

OG0001260:AMMONIUM TRANSPORTER 1.1

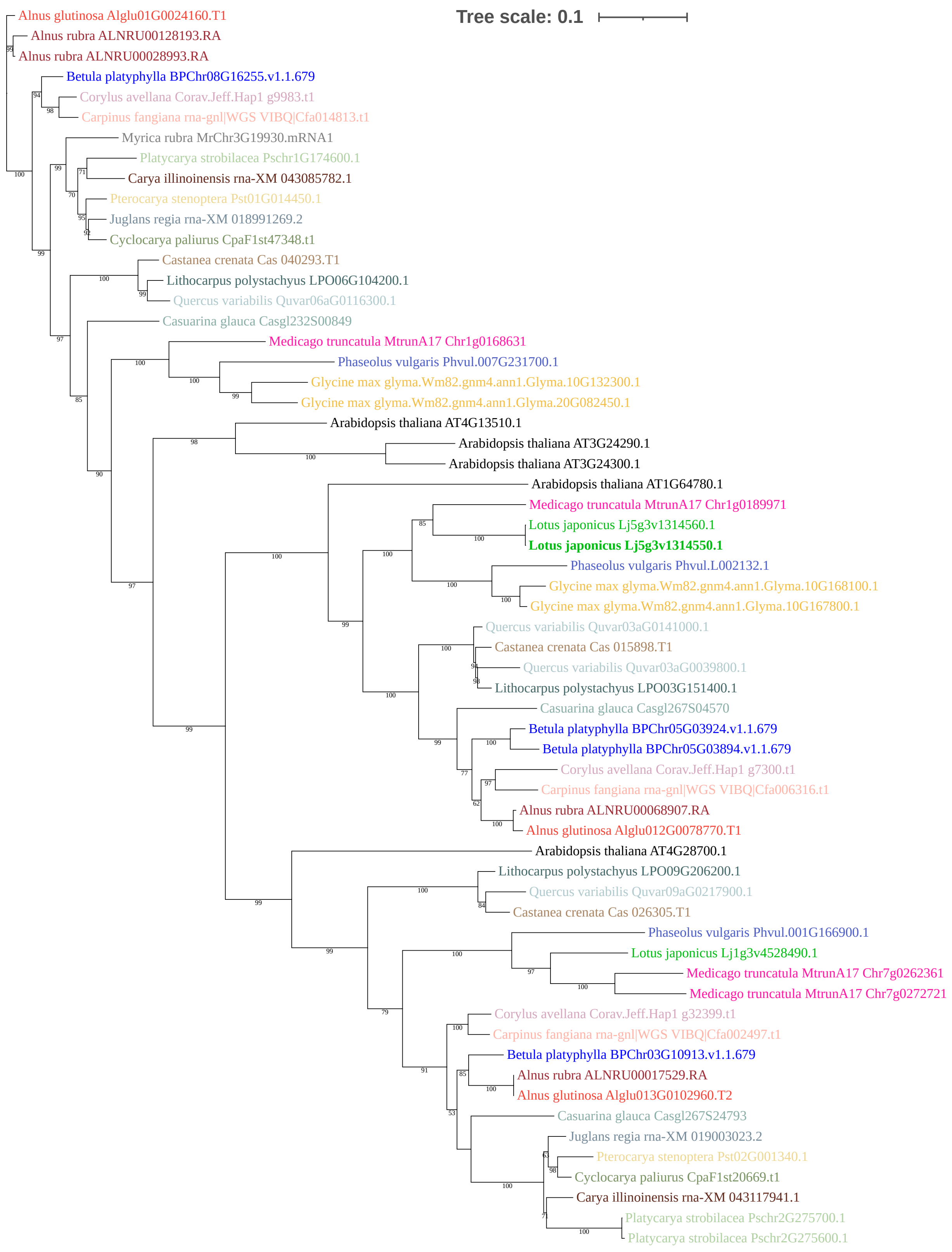

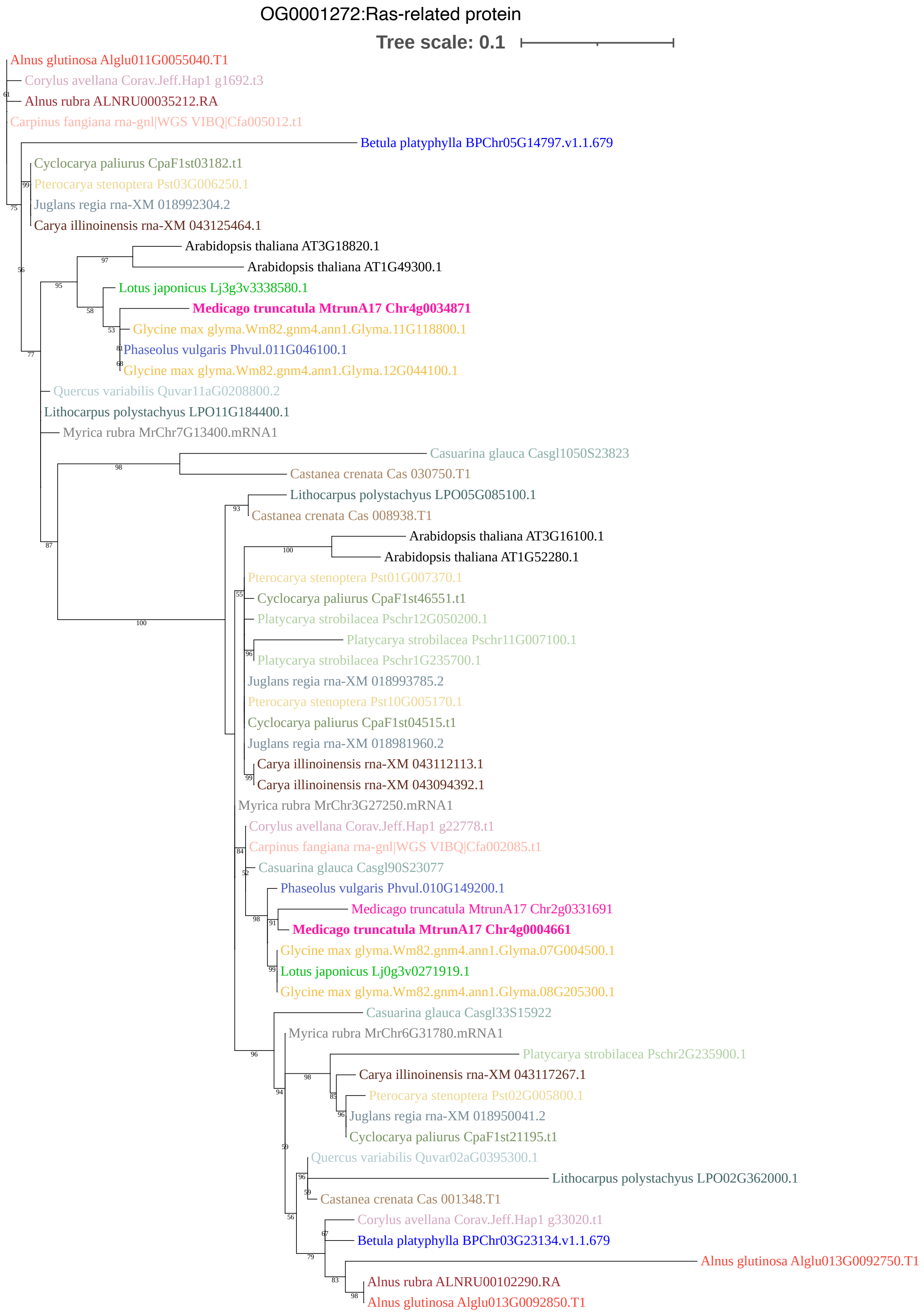
OG0001272:Ras-related protein
Tree scale: 0.1
Alnus glutinosa Alglu011G0055040.T1
Corylus avellana Corav.Jeff.Hap1 g1692.t3
Alnus rubra ALNRU00035212.RA
Carpinus fangiana rna-gnl|WGS VIBQ|Cfa005012.t1
Betula platyphylla BPChr05G14797.v1.1.679
Cyclocarya paliurus CpaF1st03182.t1
Pterocarya stenoptera Pst03G006250.1
Juglans regia rna-XM 018992304.2
Carya illinoinensis rna-XM 043125464.1
Arabidopsis thaliana AT3G18820.1
Arabidopsis thaliana AT1G49300.1
Lotus japonicus Lj3g3v3338580.1
Medicago truncatula MtrunA17 Chr4g0034871
Glycine max glyma.Wm82.gnm4.ann1.Glyma.11G118800.1
Phaseolus vulgaris Phvul.011G046100.1
Glycine max glyma.Wm82.gnm4.ann1.Glyma.12G044100.1
Quercus variabilis Quvar11aG0208800.2
Lithocarpus polystachyus LPO11G184400.1
Myrica rubra MrChr7G13400.mRNA1
Casuarina glauca Casgl1050S23823
Castanea crenata Cas 030750.T1
Lithocarpus polystachyus LPO05G085100.1
Castanea crenata Cas 008938.T1
Arabidopsis thaliana AT3G16100.1
Arabidopsis thaliana AT1G52280.1
Pterocarya stenoptera Pst01G007370.1
Cyclocarya paliurus CpaF1st46551.t1
Platycarya strobilacea Pschr12G050200.1
Platycarya strobilacea Pschr11G007100.1
Platycarya strobilacea Pschr1G235700.1
Juglans regia rna-XM 018993785.2
Pterocarya stenoptera Pst10G005170.1
Cyclocarya paliurus CpaF1st04515.t1
Juglans regia rna-XM 018981960.2
Carya illinoinensis rna-XM 043112113.1
Carya illinoinensis rna-XM 043094392.1
Myrica rubra MrChr3G27250.mRNA1
Corylus avellana Corav.Jeff.Hap1 g22778.t1
Carpinus fangiana rna-gnl|WGS VIBQ|Cfa002085.t1
Casuarina glauca Casgl90S23077
Phaseolus vulgaris Phvul.010G149200.1
Medicago truncatula MtrunA17 Chr2g0331691
Medicago truncatula MtrunA17 Chr4g0004661
Glycine max glyma.Wm82.gnm4.ann1.Glyma.07G004500.1
Lotus japonicus Lj0g3v0271919.1
Glycine max glyma.Wm82.gnm4.ann1.Glyma.08G205300.1
Casuarina glauca Casgl33S15922
Myrica rubra MrChr6G31780.mRNA1
Platycarya strobilacea Pschr2G235900.1
Carya illinoinensis rna-XM 043117267.1
Pterocarya stenoptera Pst02G005800.1
Juglans regia rna-XM 018950041.2
Cyclocarya paliurus CpaF1st21195.t1
Quercus variabilis Quvar02aG0395300.1
Lithocarpus polystachyus LPO02G362000.1
Castanea crenata Cas 001348.T1
Corylus avellana Corav.Jeff.Hap1 g33020.t1
Betula platyphylla BPChr03G23134.v1.1.679
Alnus glutinosa Alglu013G0092750.T1
Alnus rubra ALNRU00102290.RA
Alnus glutinosa Alglu013G0092850.T1

OG0001282:GIBERELLIC ACID 20-OXIDASE1a

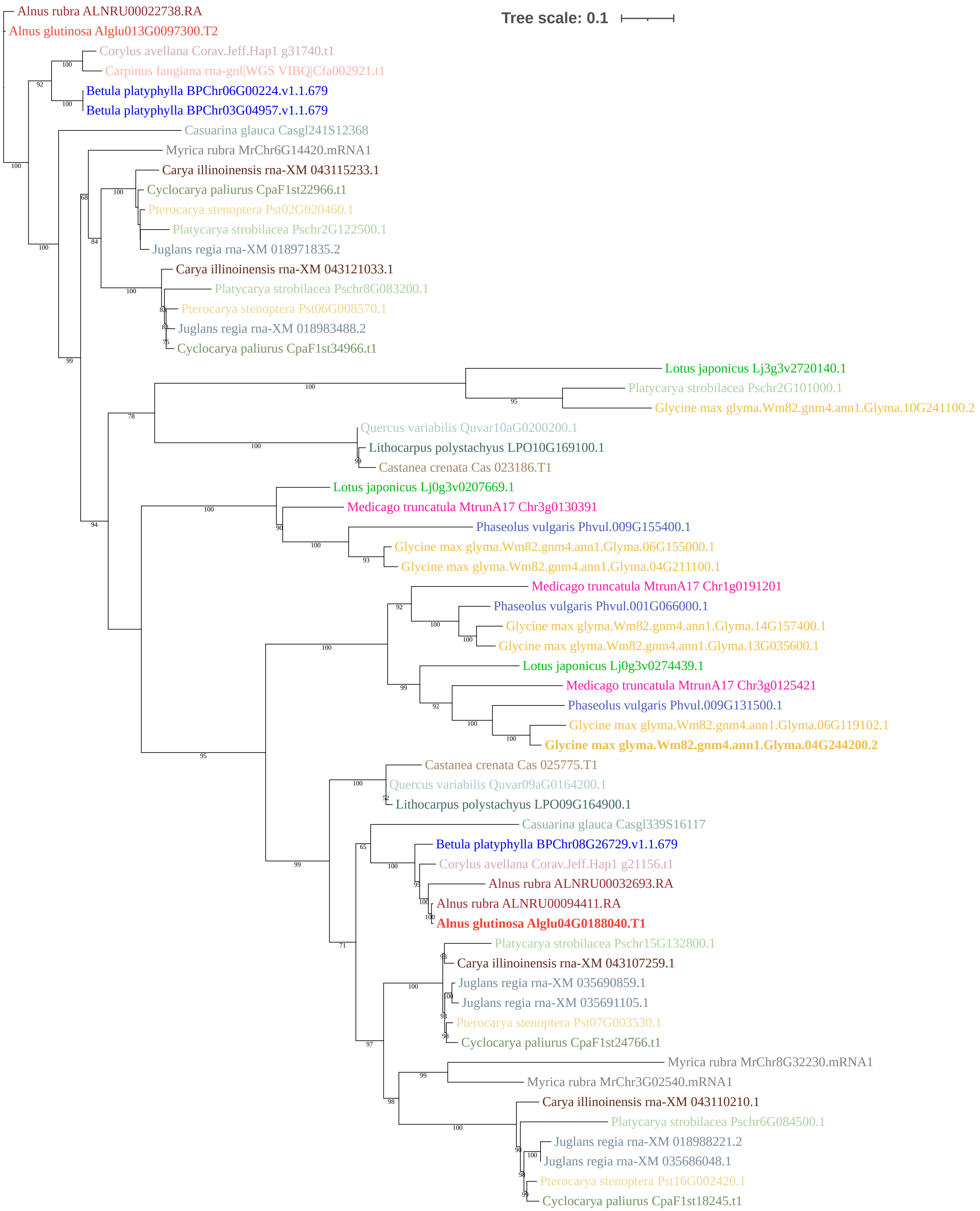

OG0001338:CLATHARIN HEAVY CHAIN1

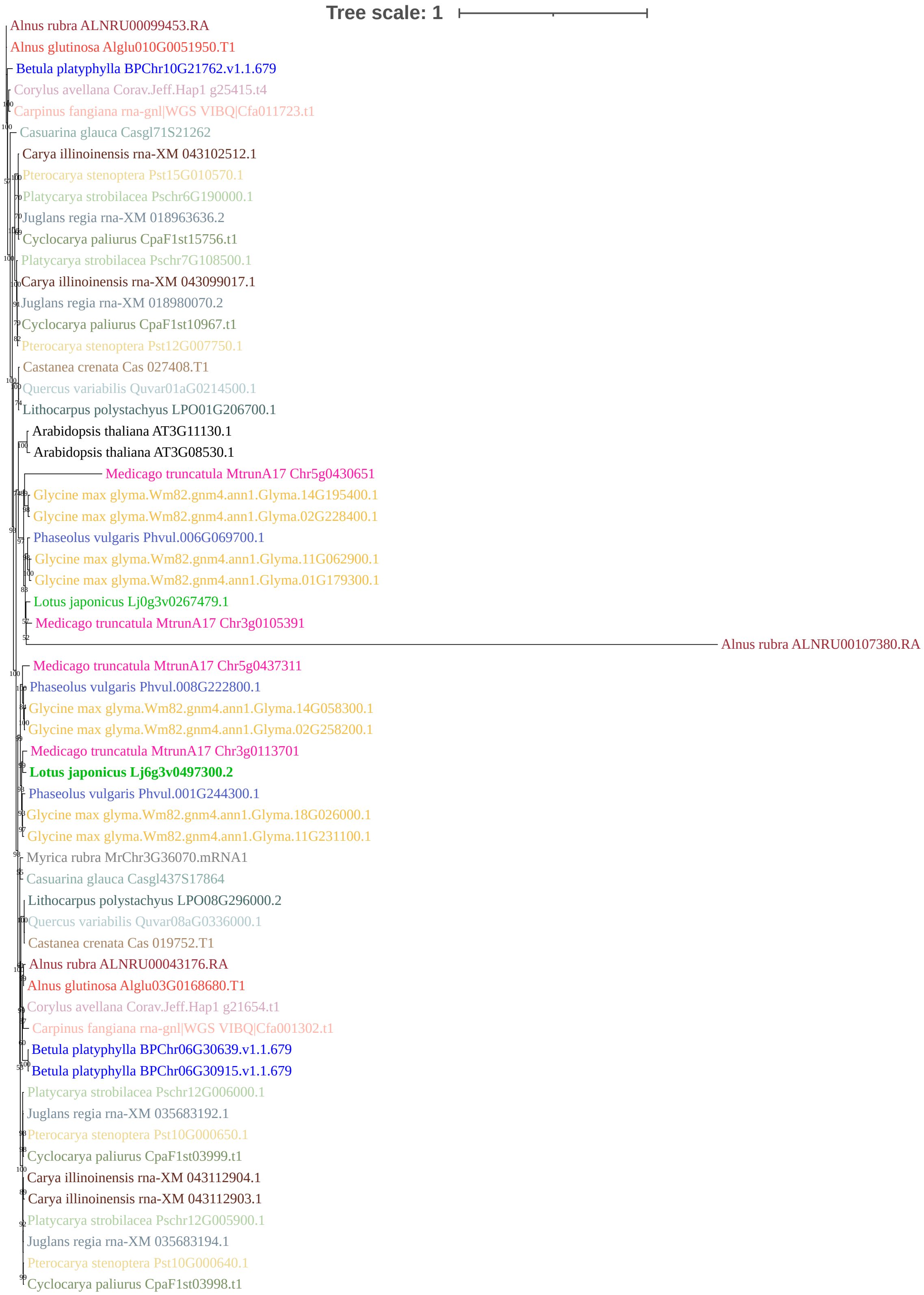

OG0001341:CYSTEINE PROTEASE 15a

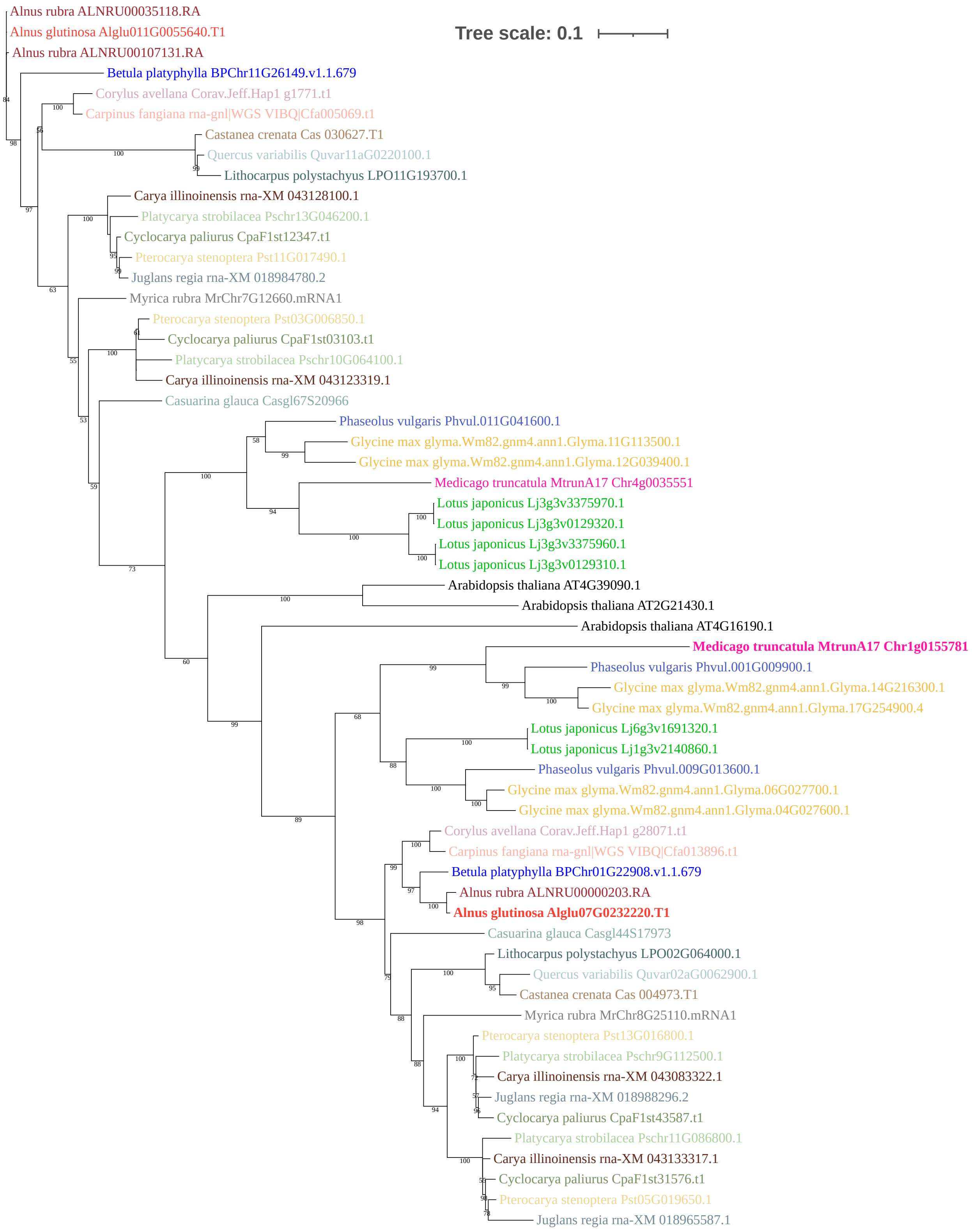

OG0001393:NAM/ATAF/CUC-encoding 018/039

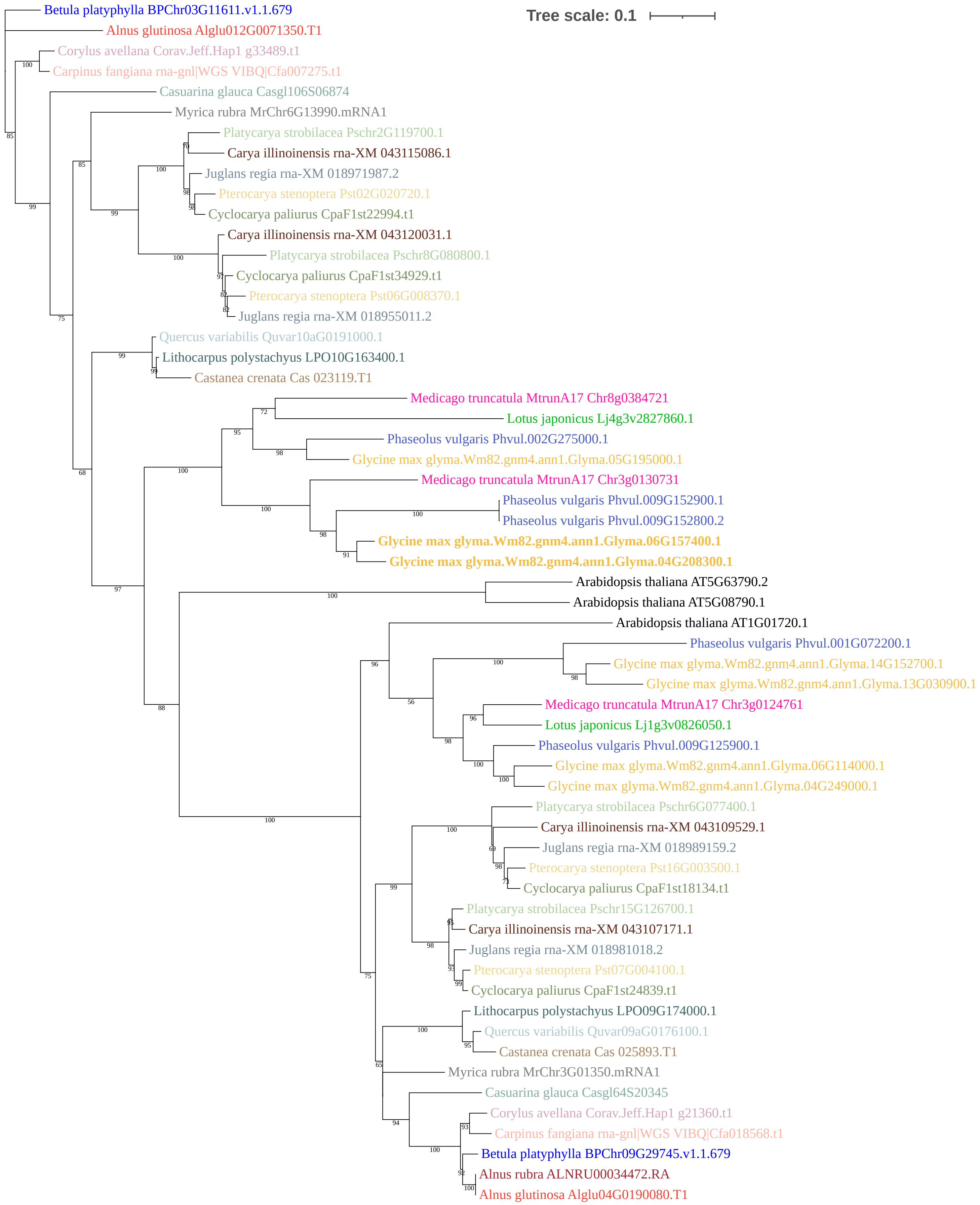

OG0001395:PHOSPHATE STARVATION RESPONSE1

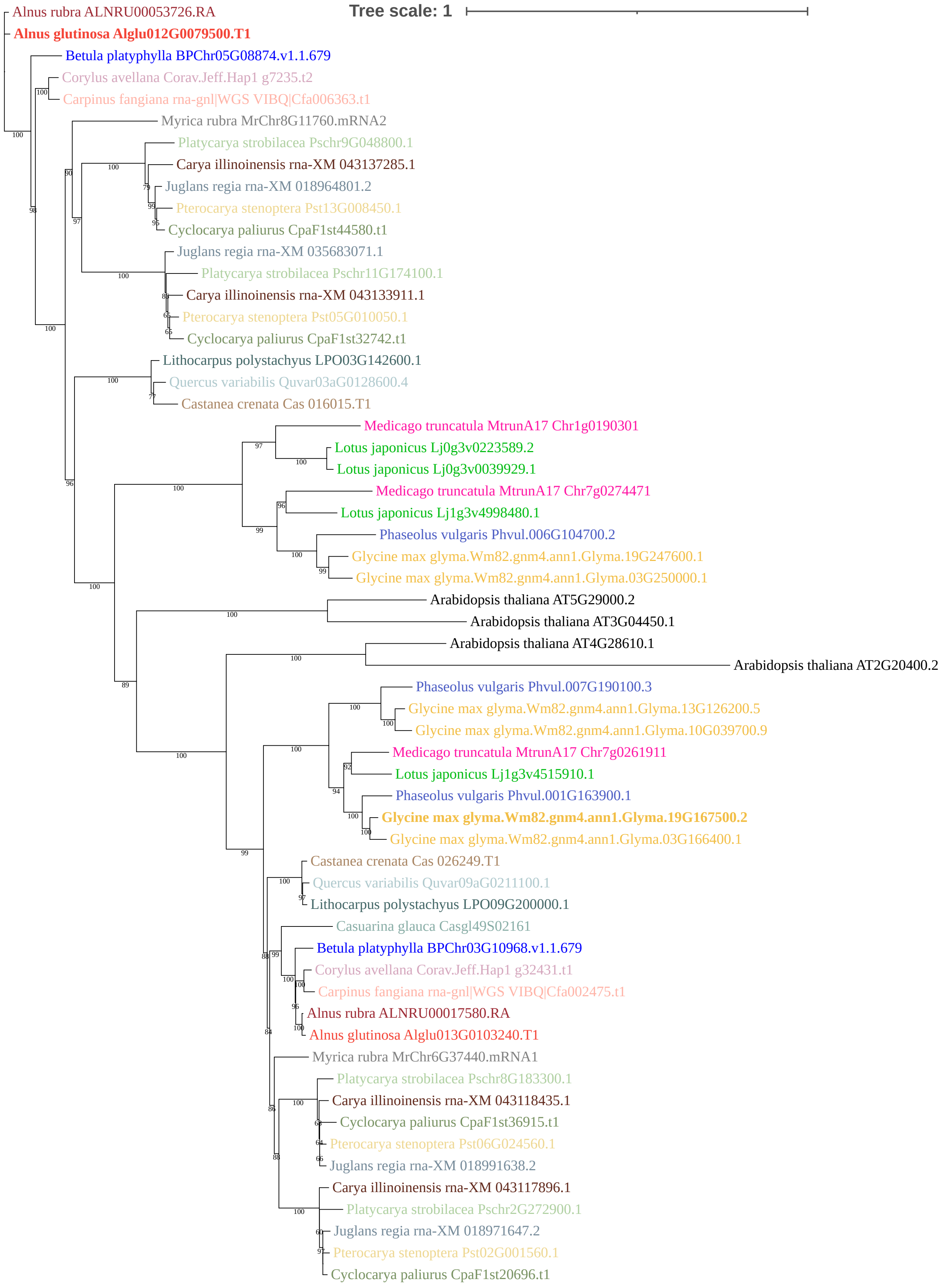

OG0001398:RESPIRATORY BURST OXIDASE HOMOLOG K

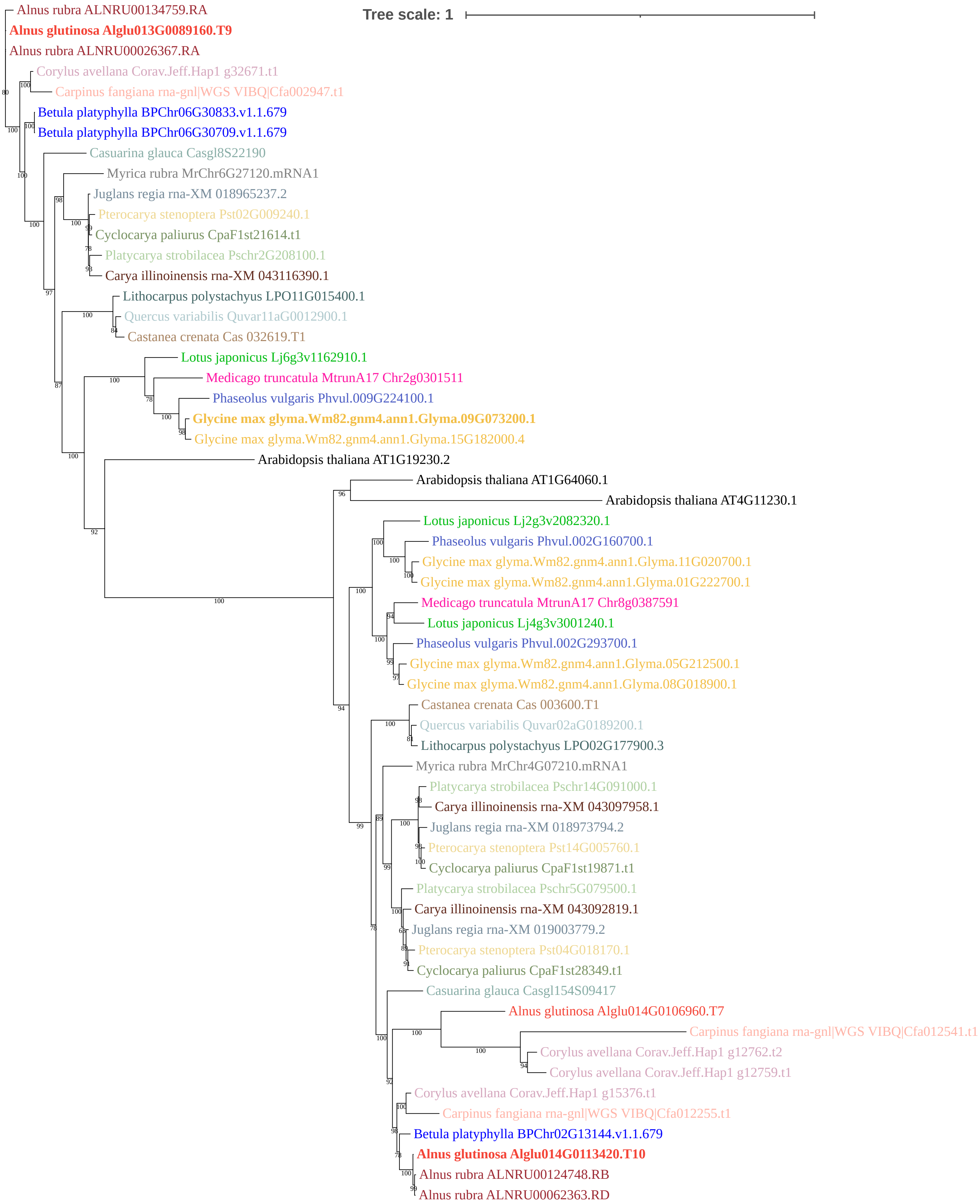

OG0001414:annexin

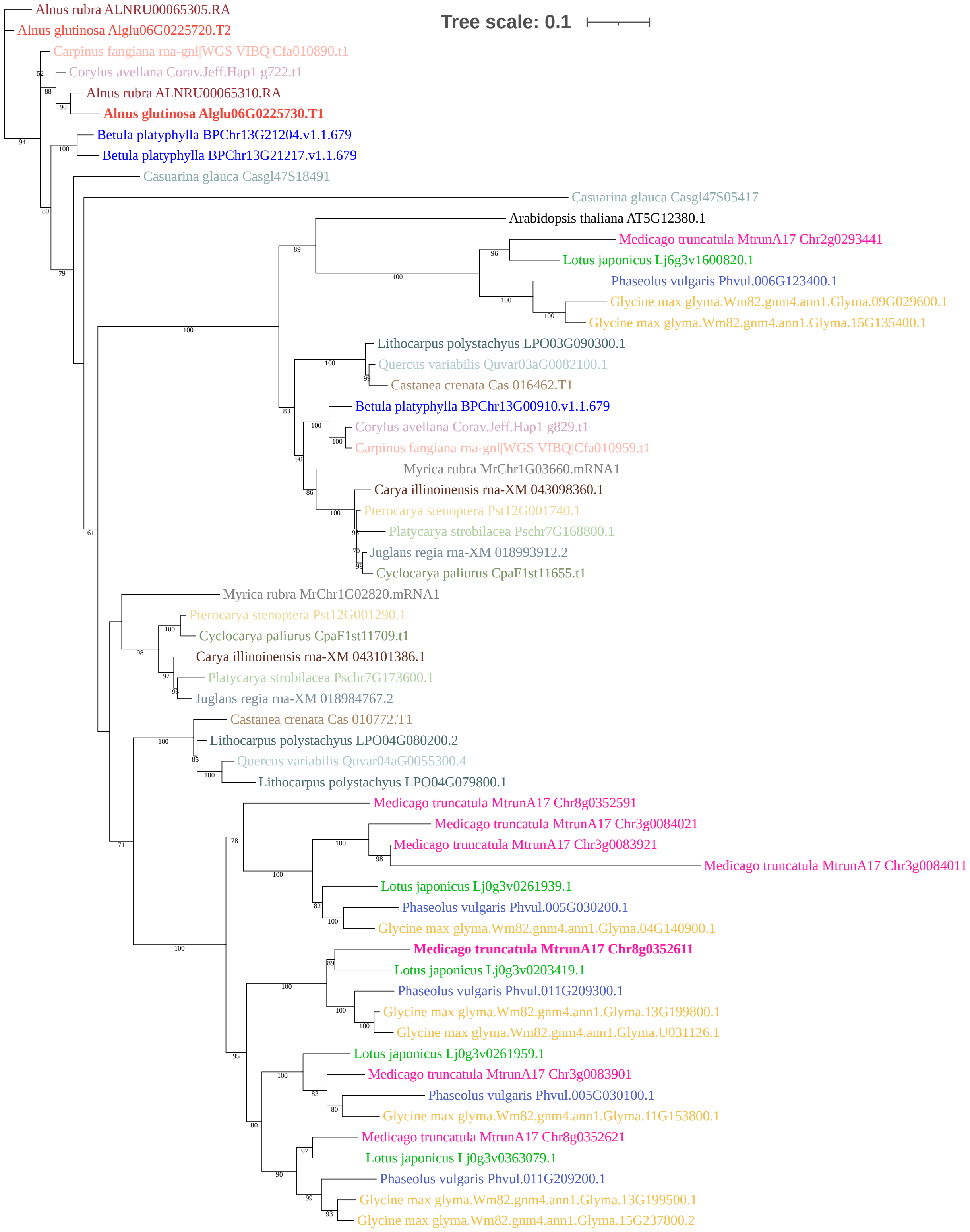

OG0001429:NUCLEAR FACTOR YC1

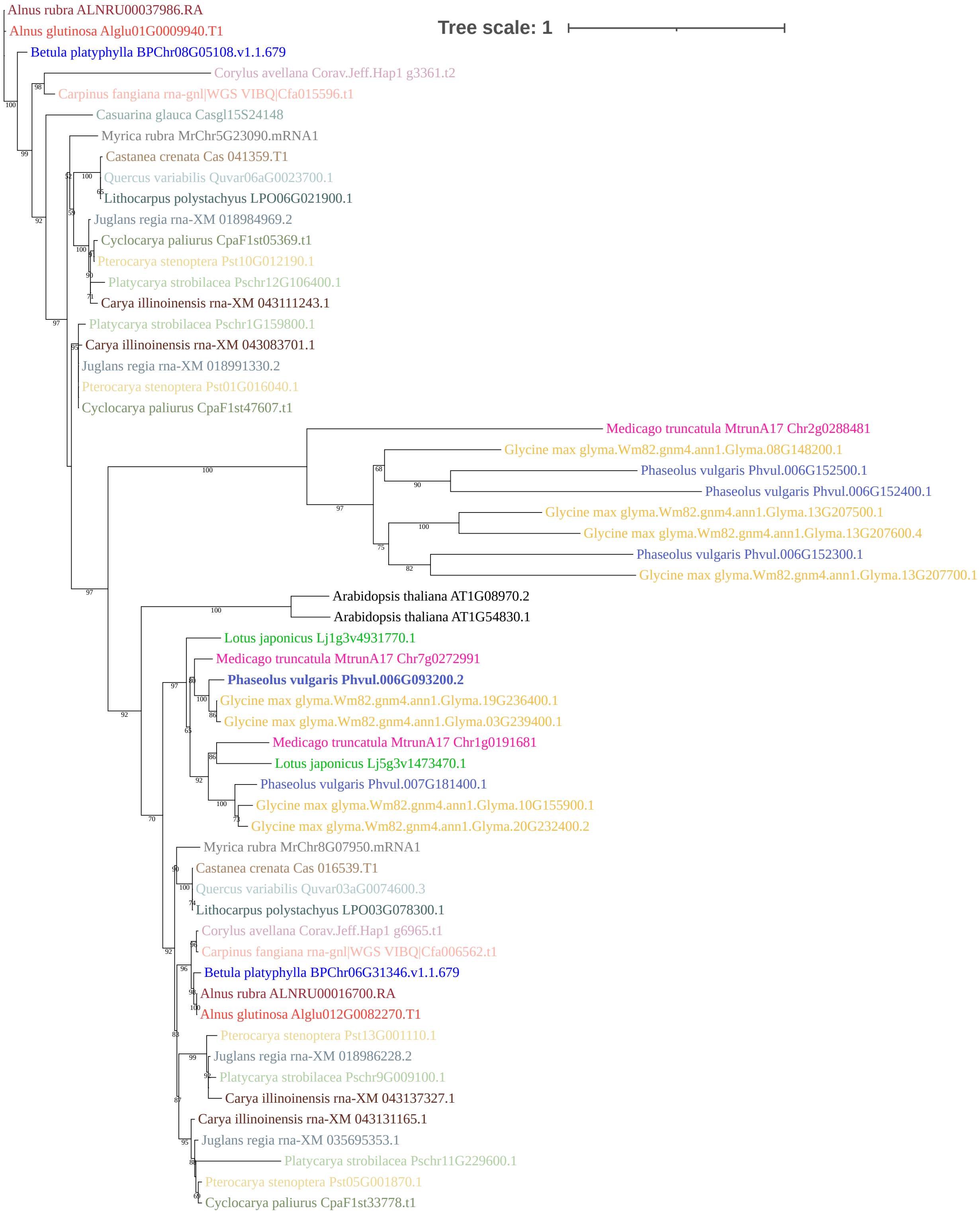

OG0001464:FERRITINS3/2

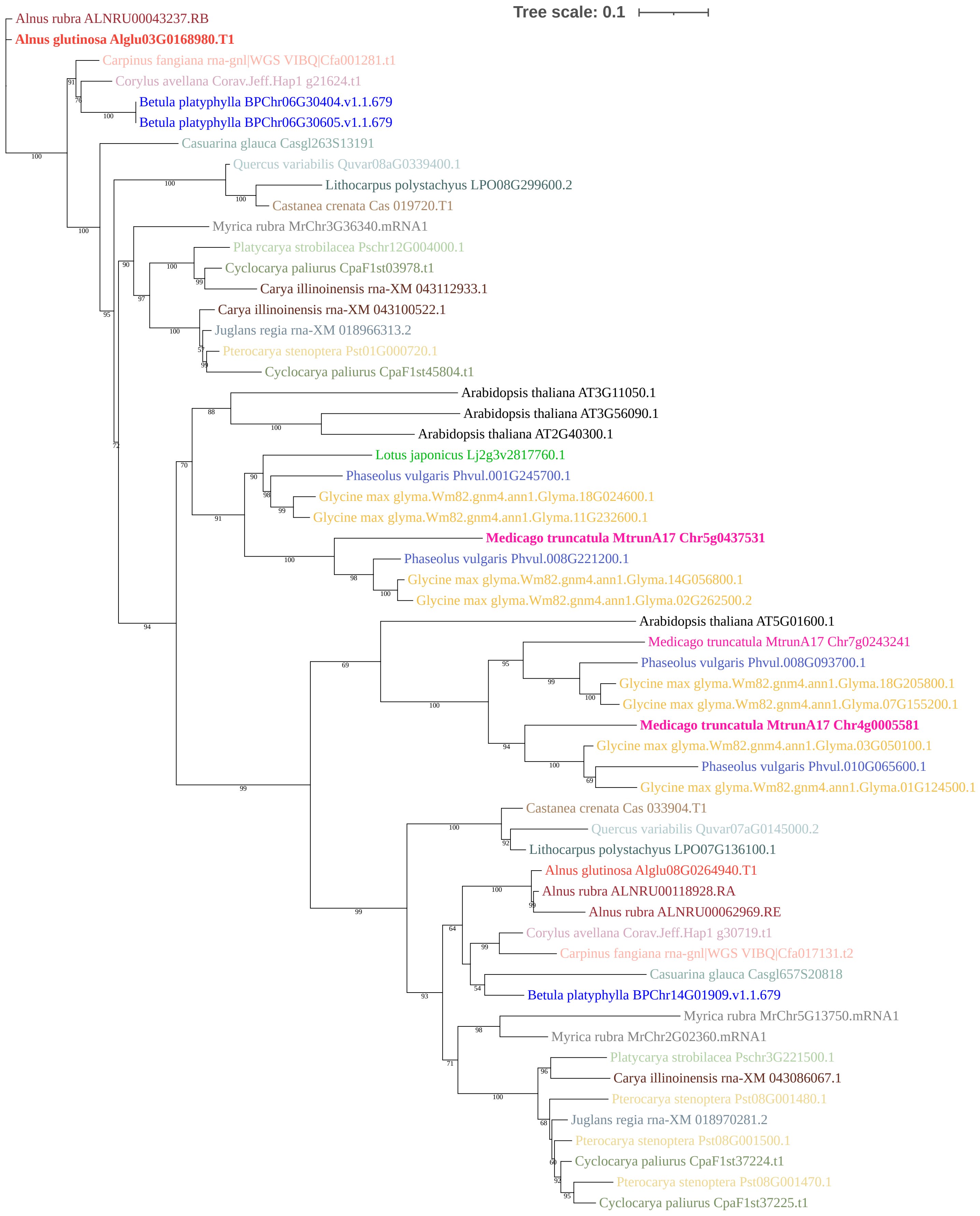

OG0001492:type IIA calcium ATPase 8

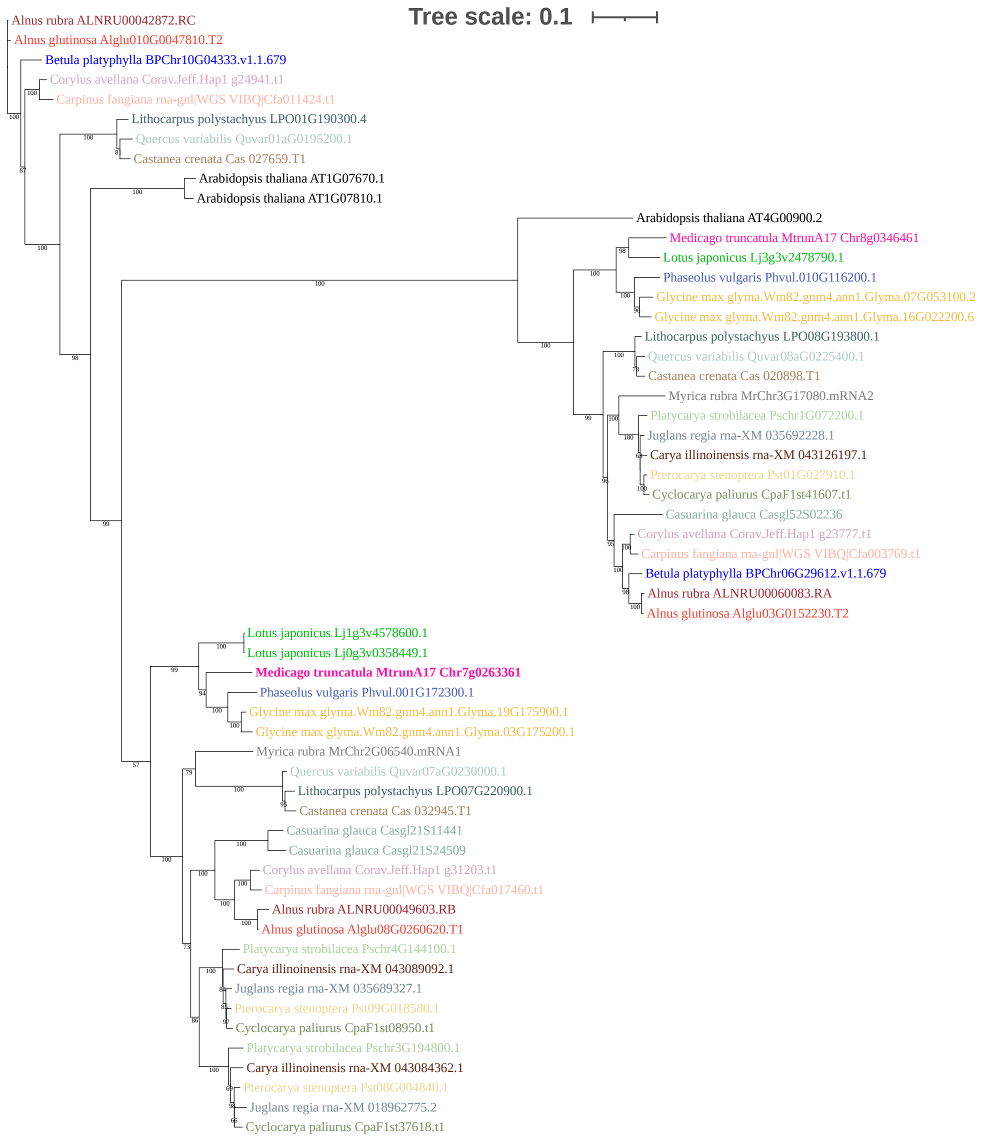

OG0001525:Phosphorylation of phosphoenolpyruvate carboxylase kinase

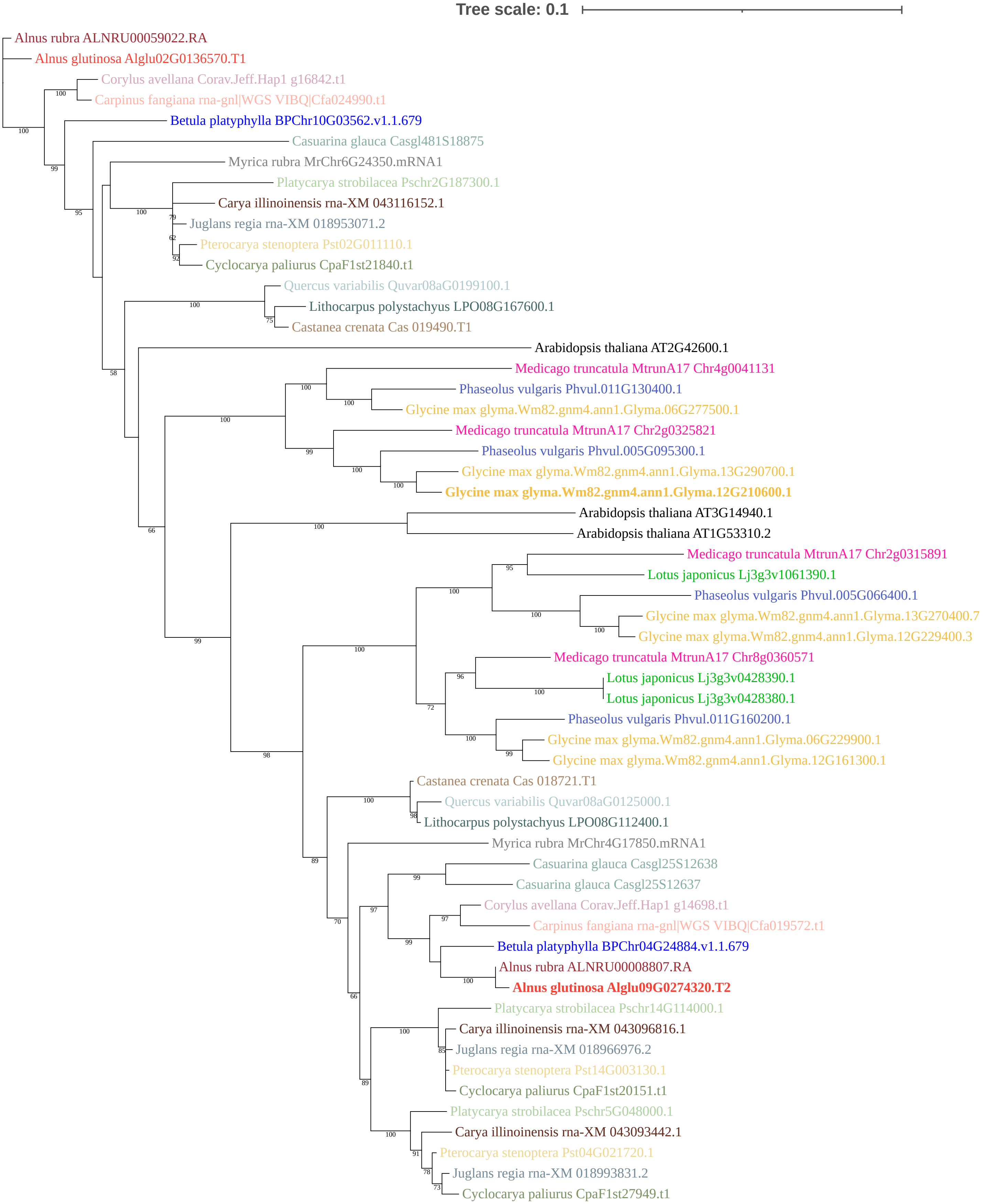

OG0001533:KNOTTED-1 LIKE TAKE HOMEDOMAIN proteins

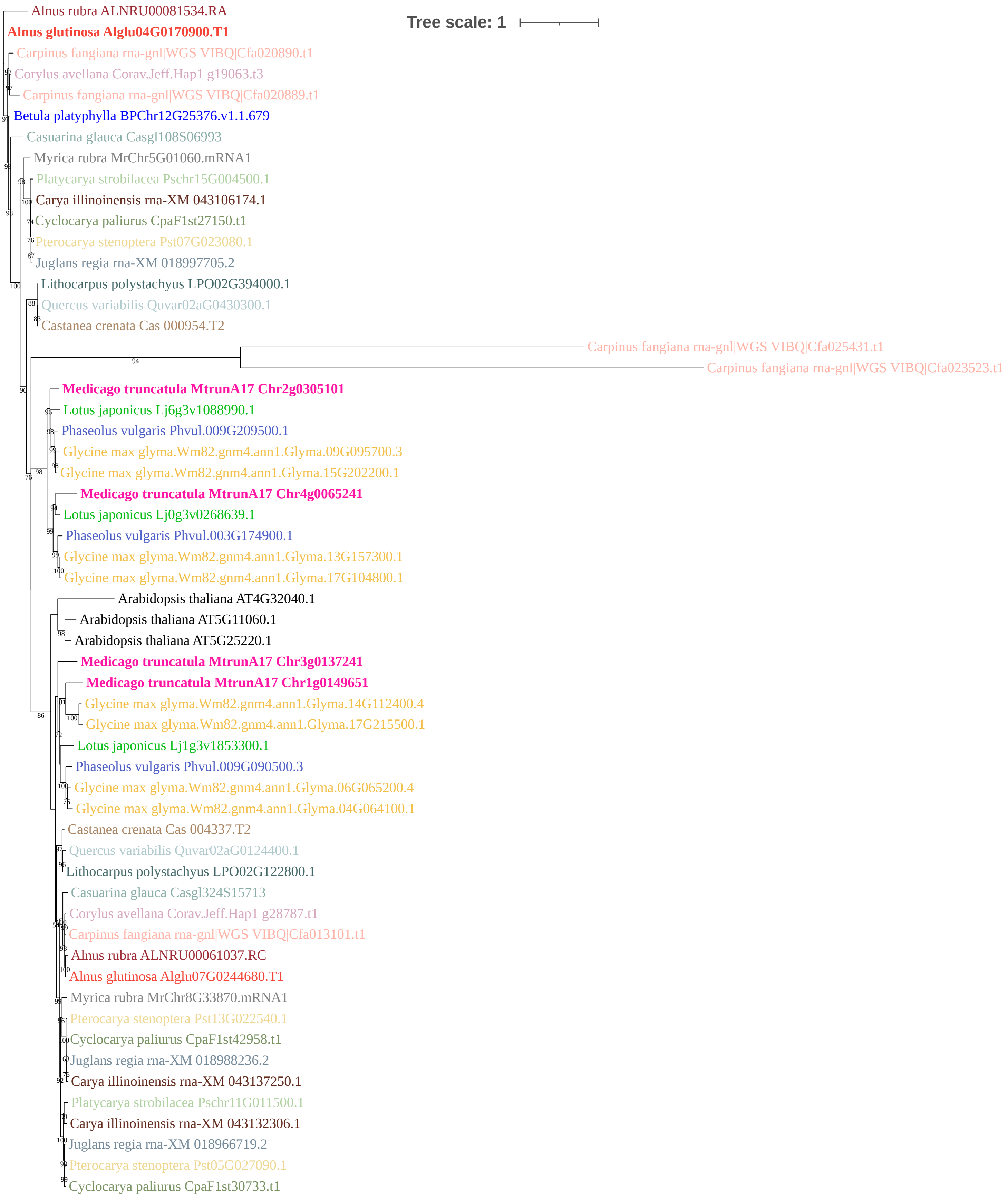

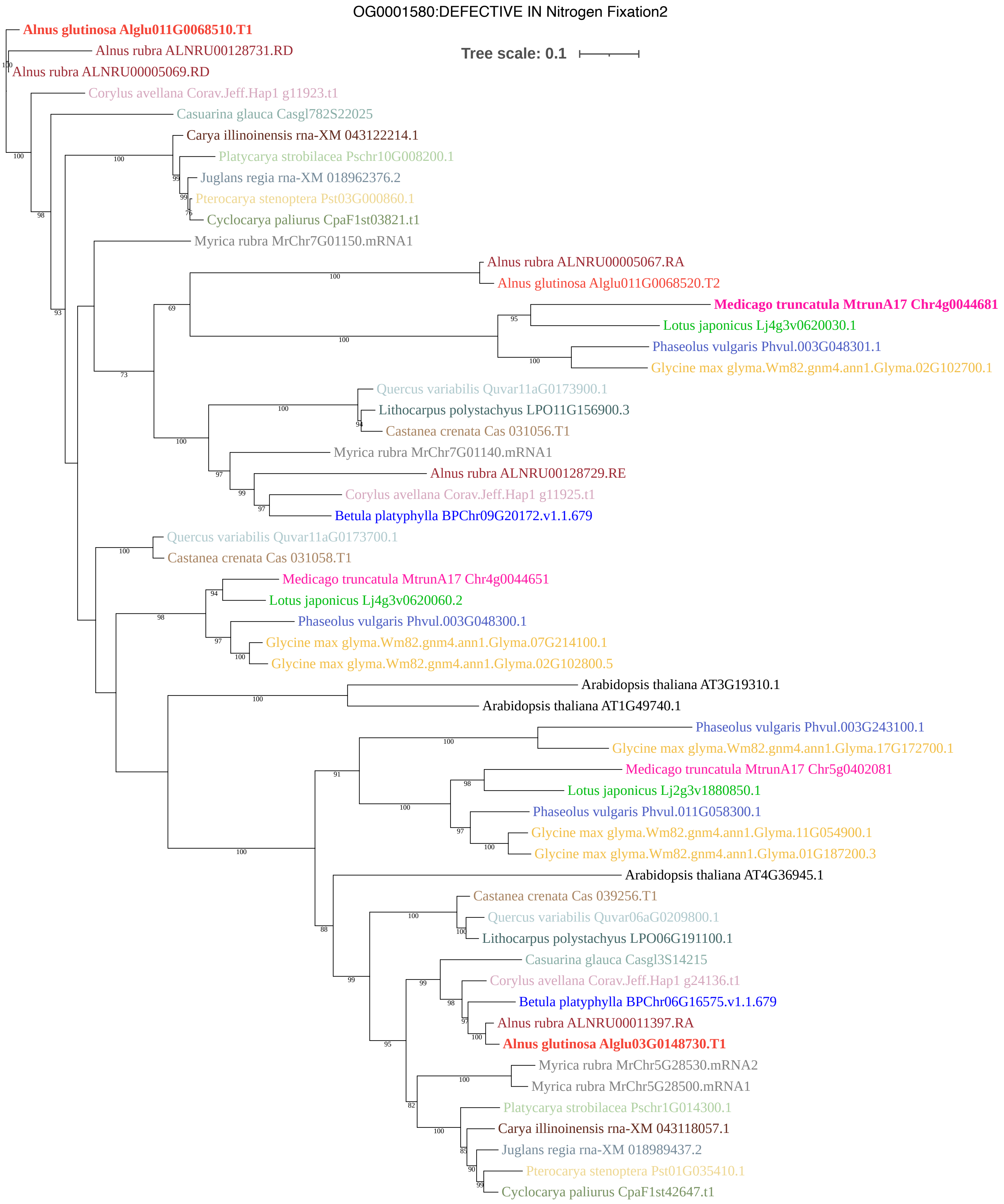
OG0001580:DEFECTIVE IN Nitrogen Fixation2
Tree scale: 0.1
Alnus glutinosa Alglu011G0068510.T1
Alnus rubra ALNRU00128731.RD
Alnus rubra ALNRU00005069.RD
Corylus avellana Corav.Jeff.Hap1 g11923.t1
Casuarina glauca Casgl782S22025
Carya illinoinensis rna-XM 043122214.1
Platycarya strobilacea Pschr10G008200.1
Juglans regia rna-XM 018962376.2
Pterocarya stenoptera Pst03G000860.1
Cyclocarya paliurus CpaF1st03821.t1
Myrica rubra MrChr7G01150.mRNA1
Alnus rubra ALNRU00005067.RA
Alnus glutinosa Alglu011G0068520.T2
Medicago truncatula MtrunA17 Chr4g0044681
Lotus japonicus Lj4g3v0620030.1
Phaseolus vulgaris Phvul.003G048301.1
Glycine max glyma.Wm82.gnm4.ann1.Glyma.02G102700.1
Quercus variabilis Quvar11aG0173900.1
Lithocarpus polystachyus LPO11G156900.3
Castanea crenata Cas 031056.T1
Myrica rubra MrChr7G01140.mRNA1
Alnus rubra ALNRU00128729.RE
Corylus avellana Corav.Jeff.Hap1 g11925.t1
Betula platyphylla BPChr09G20172.v1.1.679
Quercus variabilis Quvar11aG0173700.1
Castanea crenata Cas 031058.T1
Medicago truncatula MtrunA17 Chr4g0044651
Lotus japonicus Lj4g3v0620060.2
Phaseolus vulgaris Phvul.003G048300.1
Glycine max glyma.Wm82.gnm4.ann1.Glyma.07G214100.1
Glycine max glyma.Wm82.gnm4.ann1.Glyma.02G102800.5
Arabidopsis thaliana AT3G19310.1
Arabidopsis thaliana AT1G49740.1
Phaseolus vulgaris Phvul.003G243100.1
Glycine max glyma.Wm82.gnm4.ann1.Glyma.17G172700.1
Medicago truncatula MtrunA17 Chr5g0402081
Lotus japonicus Lj2g3v1880850.1
Phaseolus vulgaris Phvul.011G058300.1
Glycine max glyma.Wm82.gnm4.ann1.Glyma.11G054900.1
Glycine max glyma.Wm82.gnm4.ann1.Glyma.01G187200.3
Arabidopsis thaliana AT4G36945.1
Castanea crenata Cas 039256.T1
Quercus variabilis Quvar06aG0209800.1
Lithocarpus polystachyus LPO06G191100.1
Casuarina glauca Casgl3S14215
Corylus avellana Corav.Jeff.Hap1 g24136.t1
Betula platyphylla BPChr06G16575.v1.1.679
Alnus rubra ALNRU00011397.RA
Alnus glutinosa Alglu03G0148730.T1
Myrica rubra MrChr5G28530.mRNA2
Myrica rubra MrChr5G28500.mRNA1
Platycarya strobilacea Pschr1G014300.1
Carya illinoinensis rna-XM 043118057.1
Juglans regia rna-XM 018989437.2
Pterocarya stenoptera Pst01G035410.1
Cyclocarya paliurus CpaF1st42647.t1

OG0001647:Krüppel-like Cys-2/His-2 ZINC FINGER PROTEIN

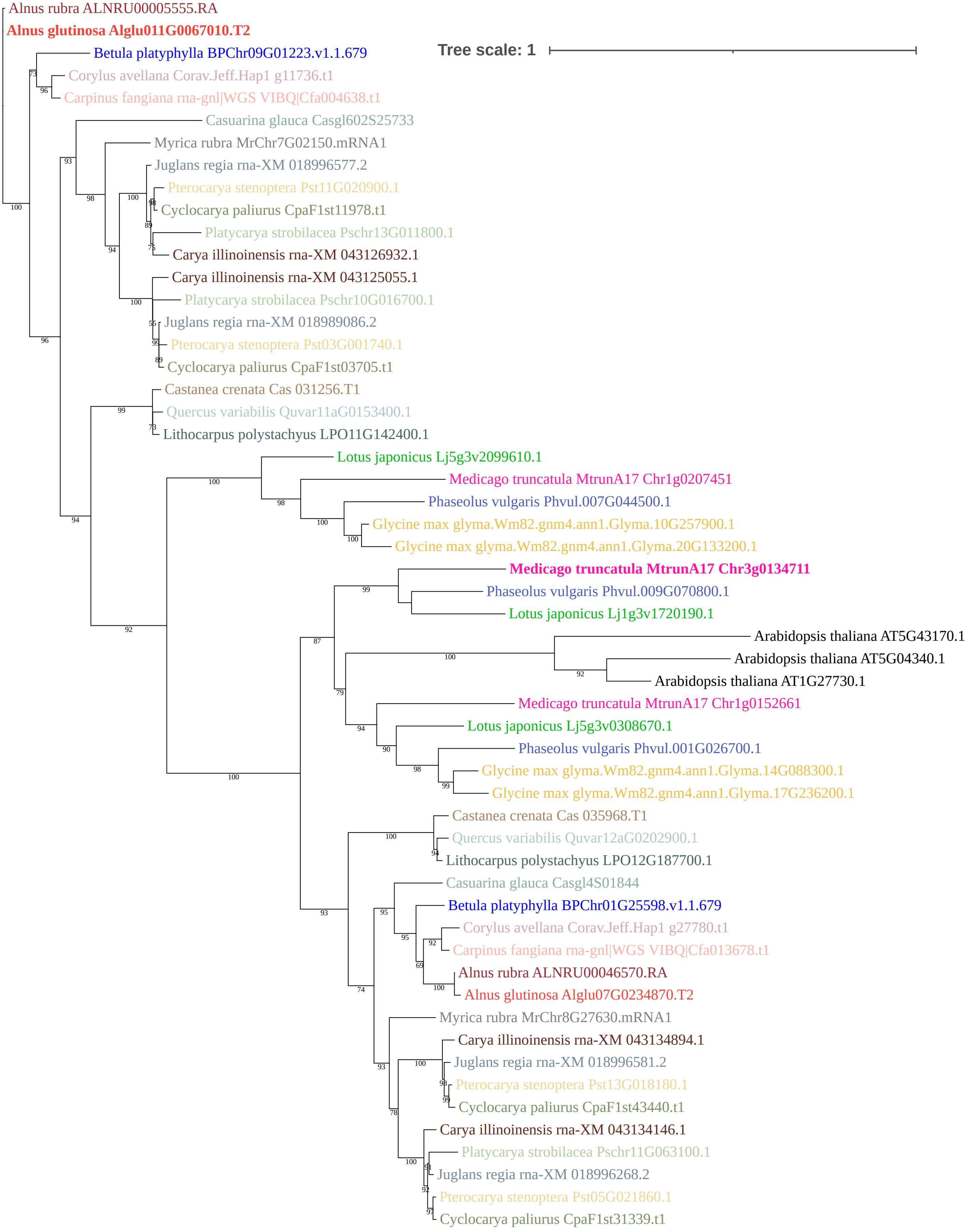

OG0001673:VACUOLAR PROCESSING ENZYME

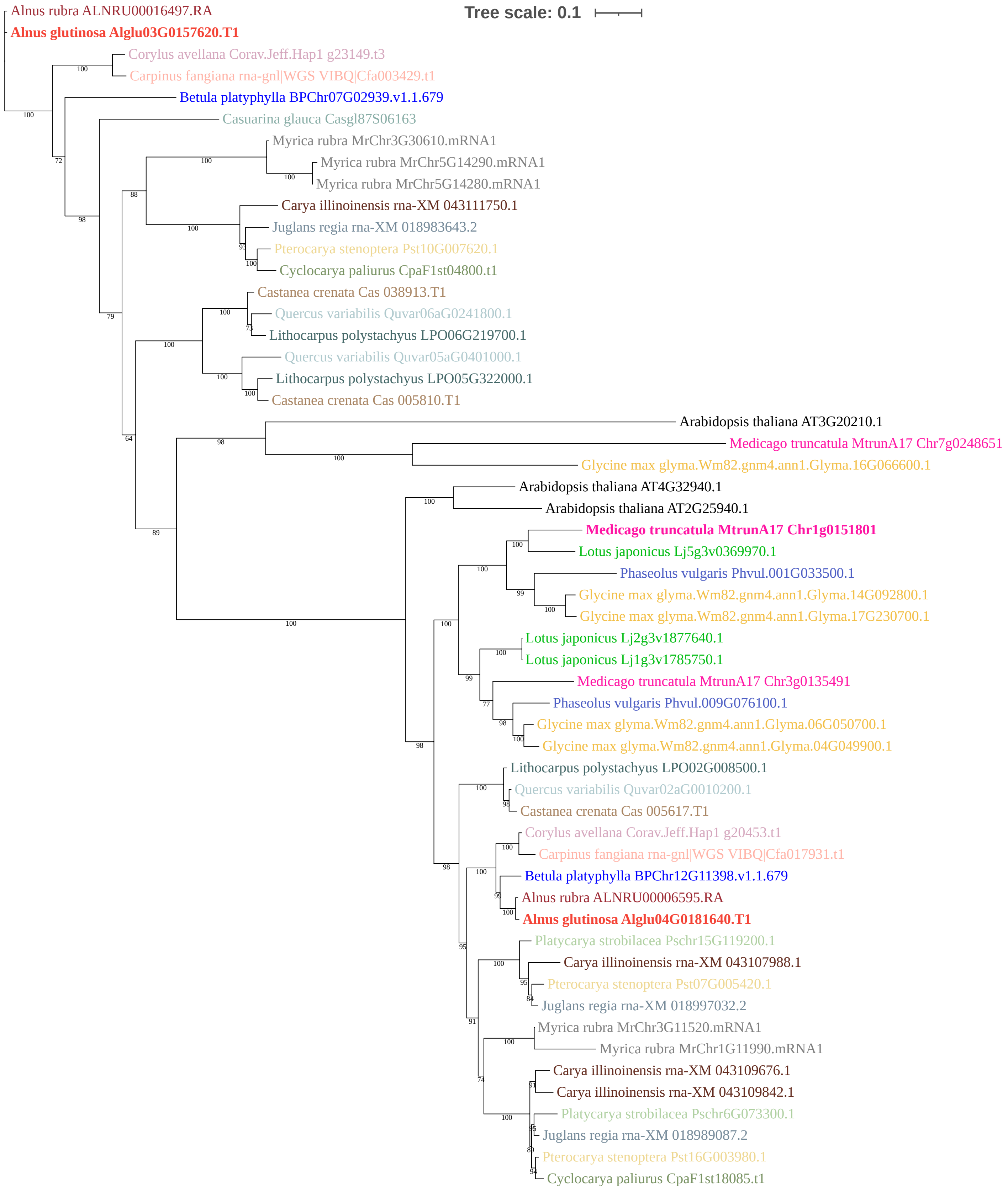

OG0001752:glycogen synthase kinase 3 (GSK3)-like kinase

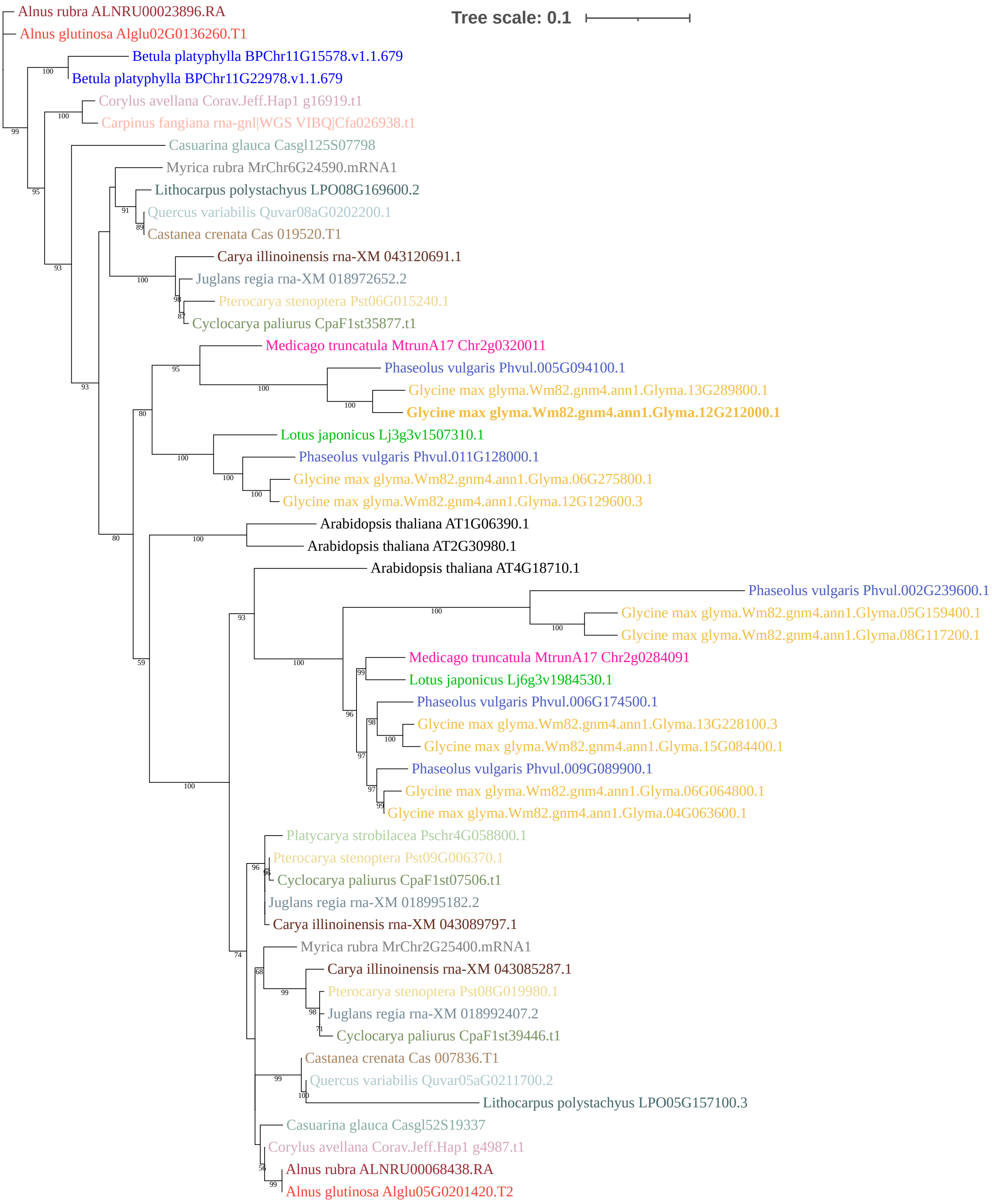

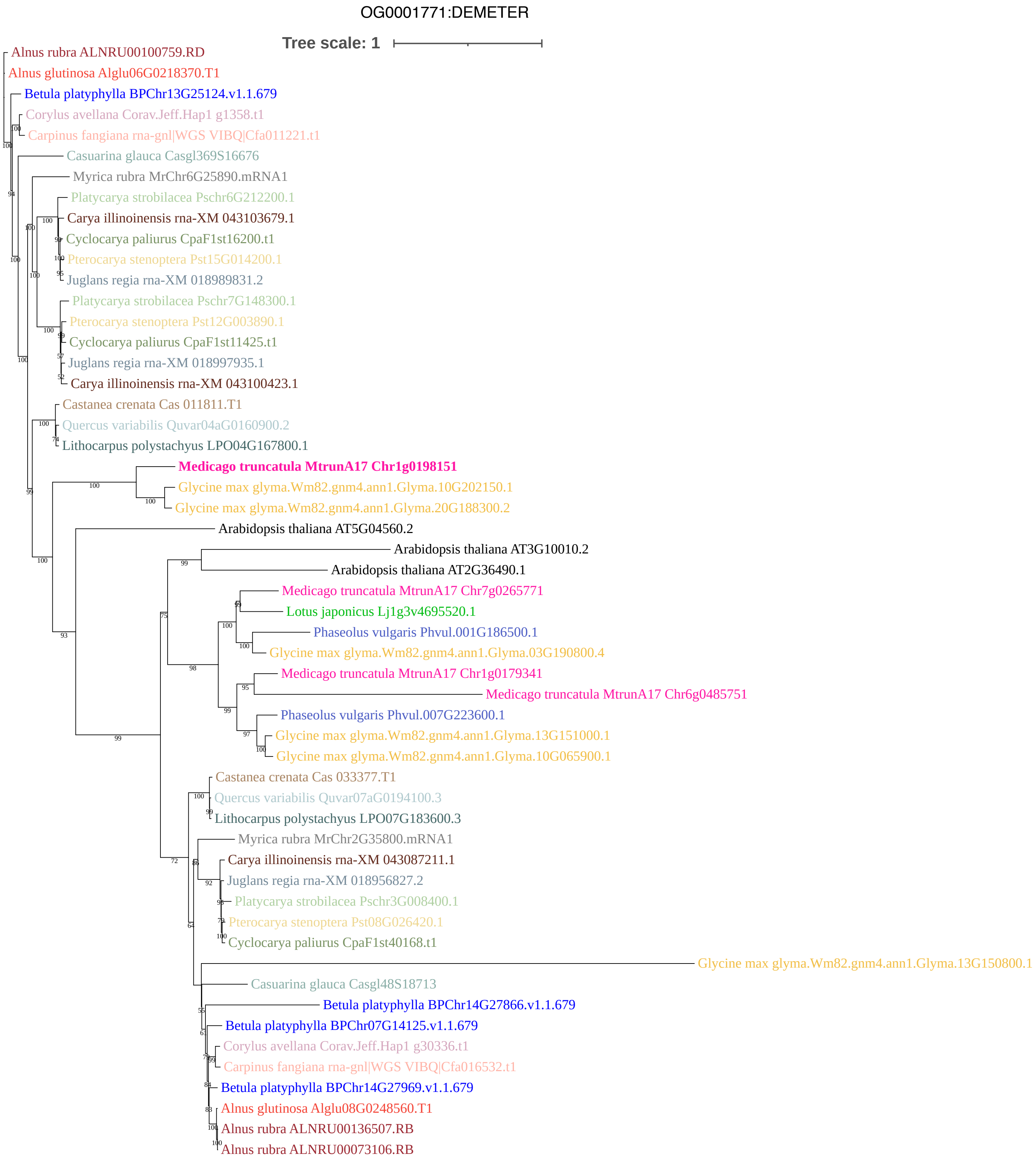
OG0001771:DEMETER
Tree scale: 1
Alnus rubra ALNRU00100759.RD
Alnus glutinosa Alglu06G0218370.T1
Betula platyphylla BPChr13G25124.v1.1.679
Corylus avellana Corav.Jeff.Hap1 g1358.t1
Carpinus fangiana rna-gnl|WGS VIBQ|Cfa011221.t1
Casuarina glauca Casgl369S16676
Myrica rubra MrChr6G25890.mRNA1
Platycarya strobilacea Pschr6G212200.1
Carya illinoinensis rna-XM 043103679.1
Cyclocarya paliurus CpaF1st16200.t1
Pterocarya stenoptera Pst15G014200.1
Juglans regia rna-XM 018989831.2
Platycarya strobilacea Pschr7G148300.1
Pterocarya stenoptera Pst12G003890.1
Cyclocarya paliurus CpaF1st11425.t1
Juglans regia rna-XM 018997935.1
Carya illinoinensis rna-XM 043100423.1
Castanea crenata Cas 011811.T1
Quercus variabilis Quvar04aG0160900.2
Lithocarpus polystachyus LPO04G167800.1
Medicago truncatula MtrunA17 Chr1g0198151
Glycine max glyma.Wm82.gnm4.ann1.Glyma.10G202150.1
Glycine max glyma.Wm82.gnm4.ann1.Glyma.20G188300.2
Arabidopsis thaliana AT5G04560.2
Arabidopsis thaliana AT3G10010.2
Arabidopsis thaliana AT2G36490.1
Medicago truncatula MtrunA17 Chr7g0265771
Lotus japonicus Lj1g3v4695520.1
Phaseolus vulgaris Phvul.001G186500.1
Glycine max glyma.Wm82.gnm4.ann1.Glyma.03G190800.4
Medicago truncatula MtrunA17 Chr1g0179341
Medicago truncatula MtrunA17 Chr6g0485751
Phaseolus vulgaris Phvul.007G223600.1
Glycine max glyma.Wm82.gnm4.ann1.Glyma.13G151000.1
Glycine max glyma.Wm82.gnm4.ann1.Glyma.10G065900.1
Castanea crenata Cas 033377.T1
Quercus variabilis Quvar07aG0194100.3
Lithocarpus polystachyus LPO07G183600.3
Myrica rubra MrChr2G35800.mRNA1
Carya illinoinensis rna-XM 043087211.1
Juglans regia rna-XM 018956827.2
Platycarya strobilacea Pschr3G008400.1
Pterocarya stenoptera Pst08G026420.1
Cyclocarya paliurus CpaF1st40168.t1
Glycine max glyma.Wm82.gnm4.ann1.Glyma.13G150800.1
Casuarina glauca Casgl48S18713
Betula platyphylla BPChr14G27866.v1.1.679
Betula platyphylla BPChr07G14125.v1.1.679
Corylus avellana Corav.Jeff.Hap1 g30336.t1
Carpinus fangiana rna-gnl|WGS VIBQ|Cfa016532.t1
Betula platyphylla BPChr14G27969.v1.1.679
Alnus glutinosa Alglu08G0248560.T1
Alnus rubra ALNRU00136507.RB
Alnus rubra ALNRU00073106.RB

OG0001891:INCREASING NODULE SIZE 1

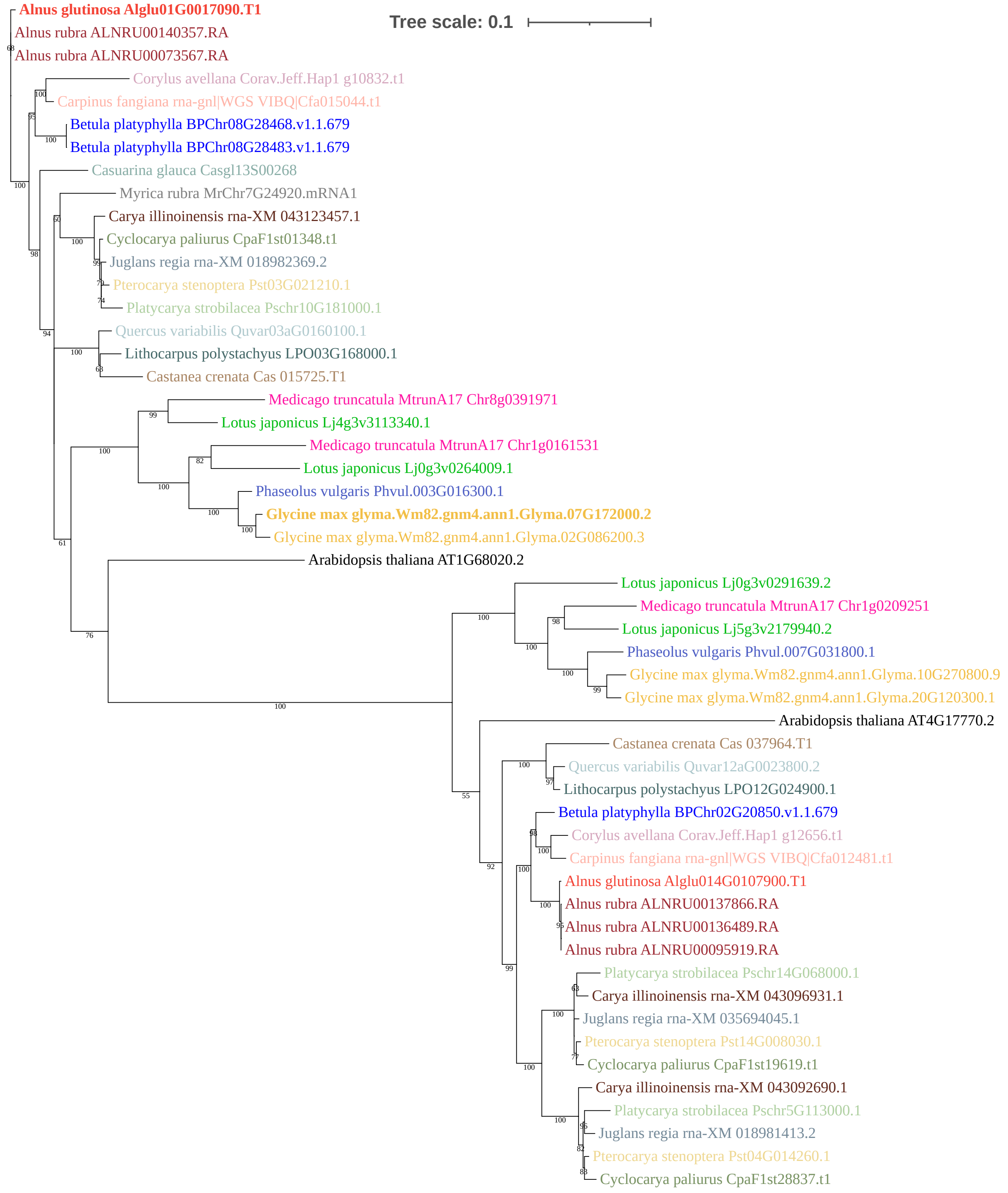

OG0001933:BES1/BZR1 HOMOLOG LIKE1/brassinosteroids signaling component

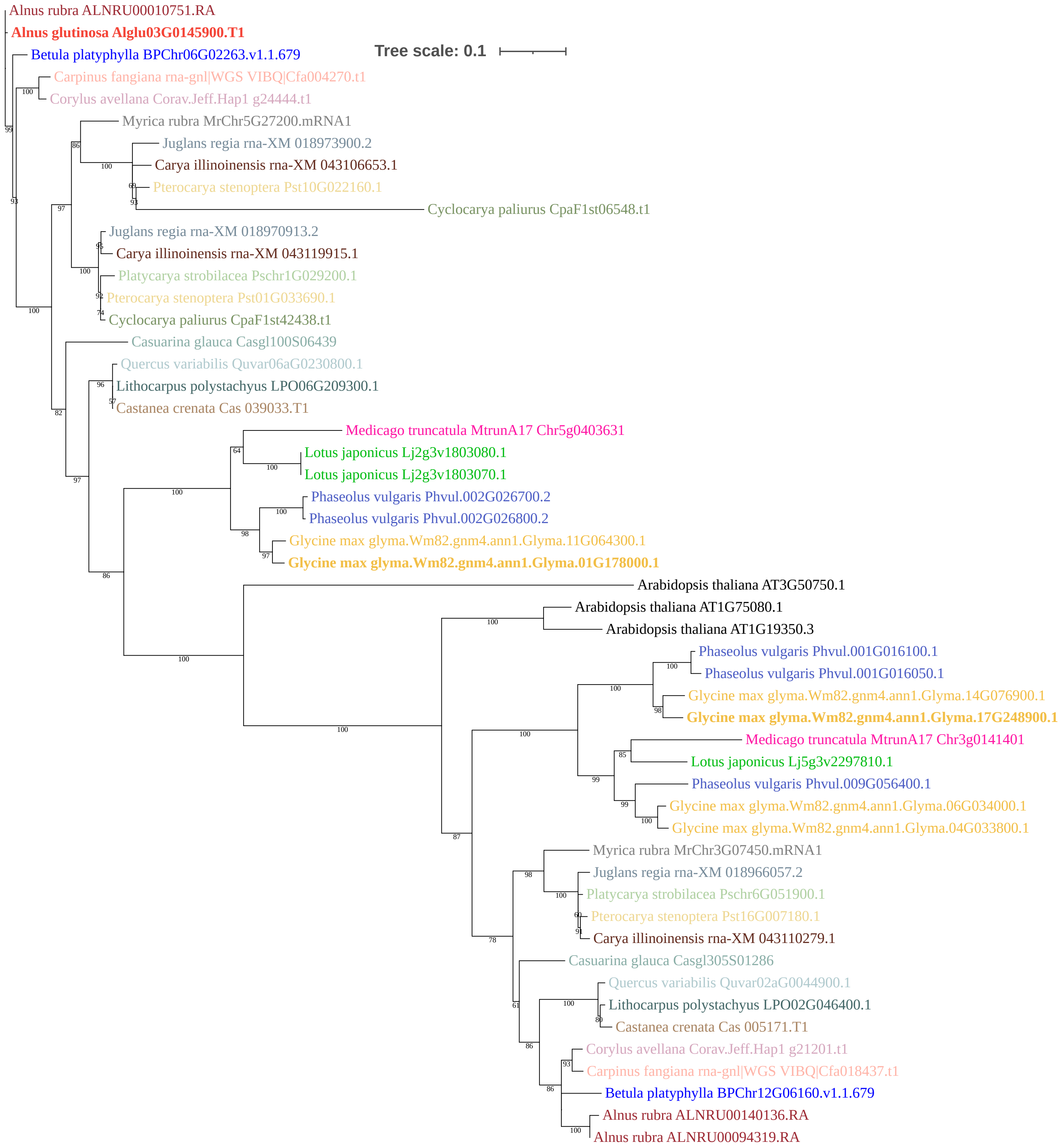

OG0001951:NFR5-interacting cytoplasmic kinase 4

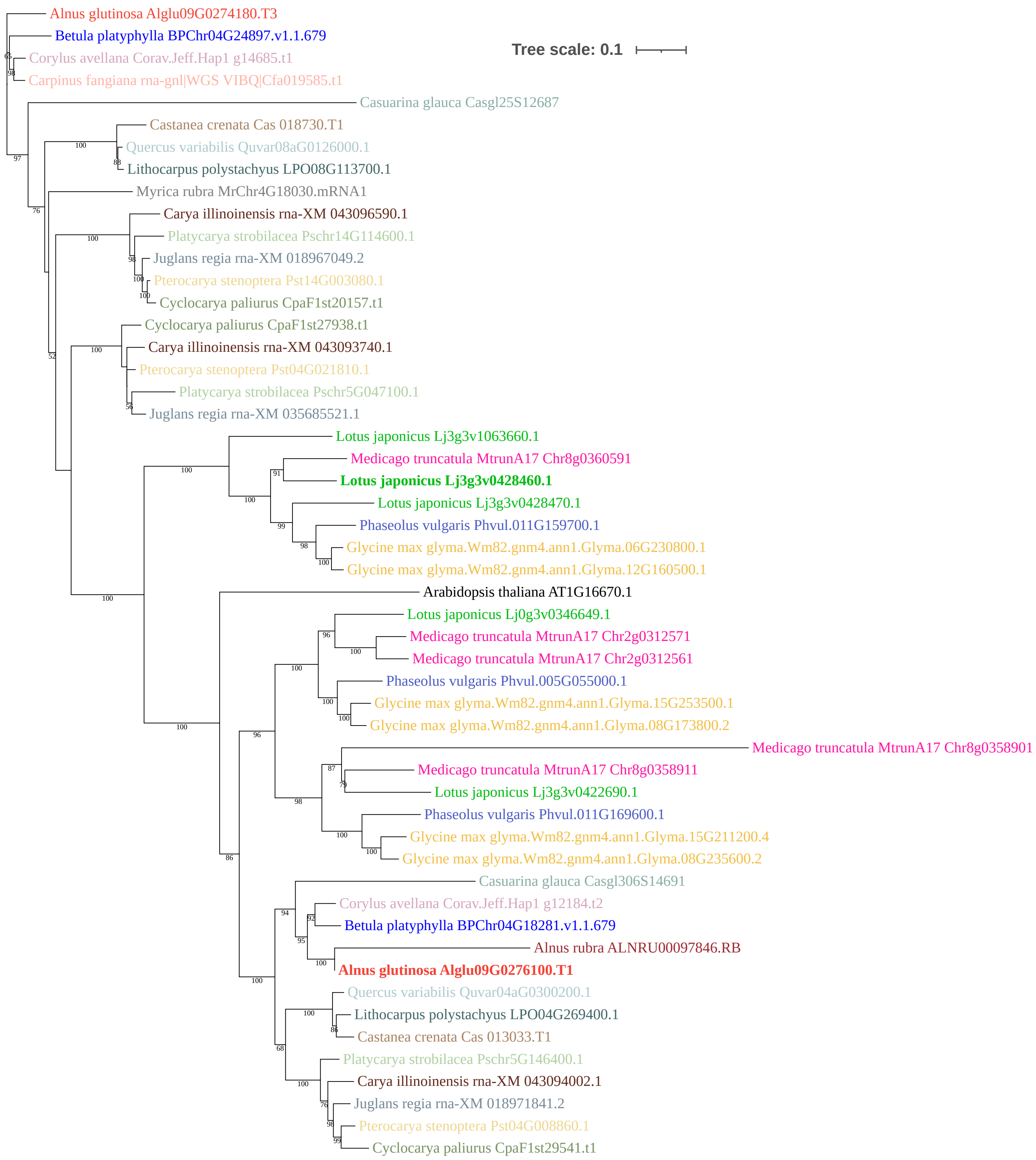

OG0001981:SYMBIOTIC FORMIN1

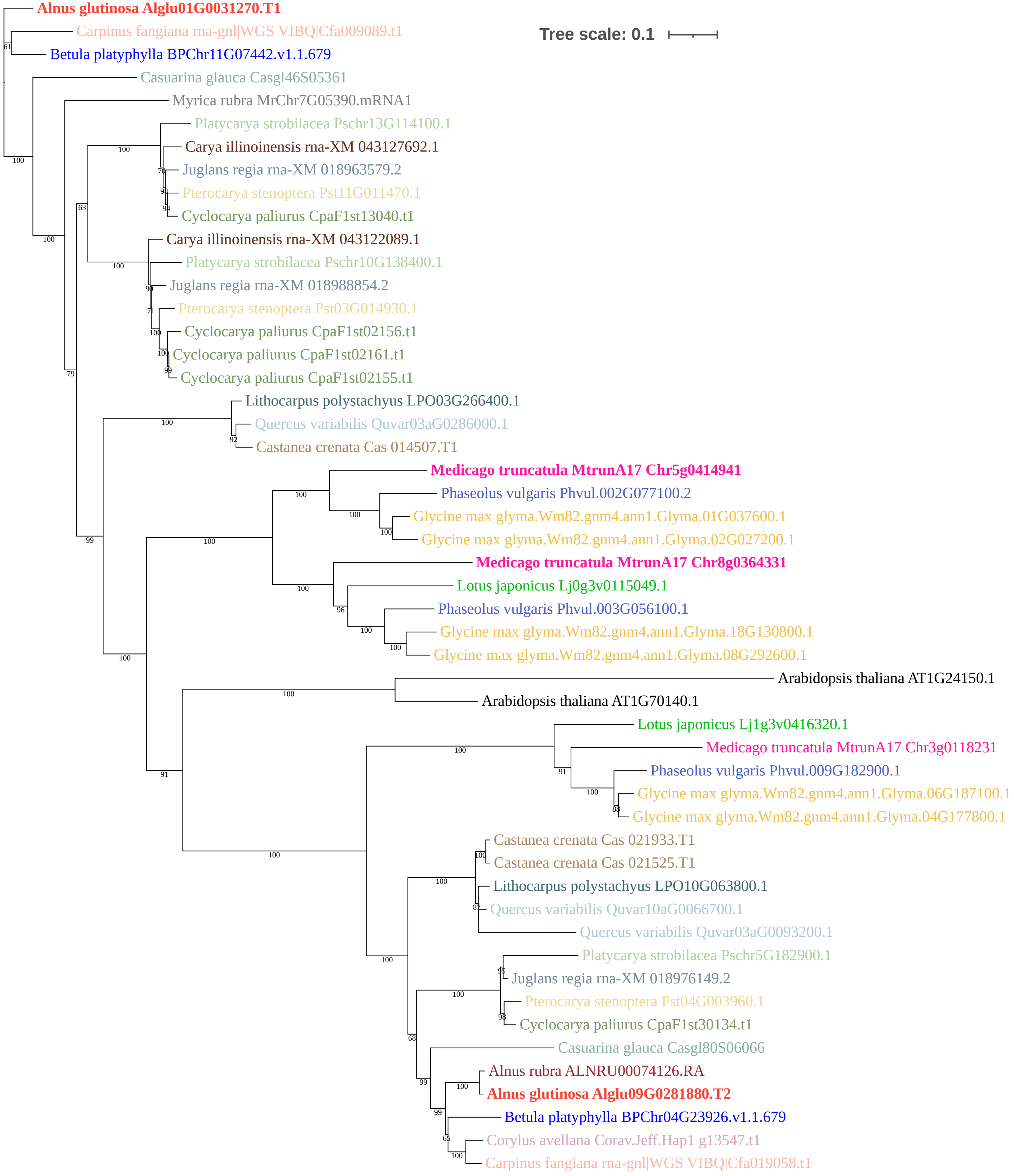

OG0001985:ROOT DETERMINED NODULATION 1

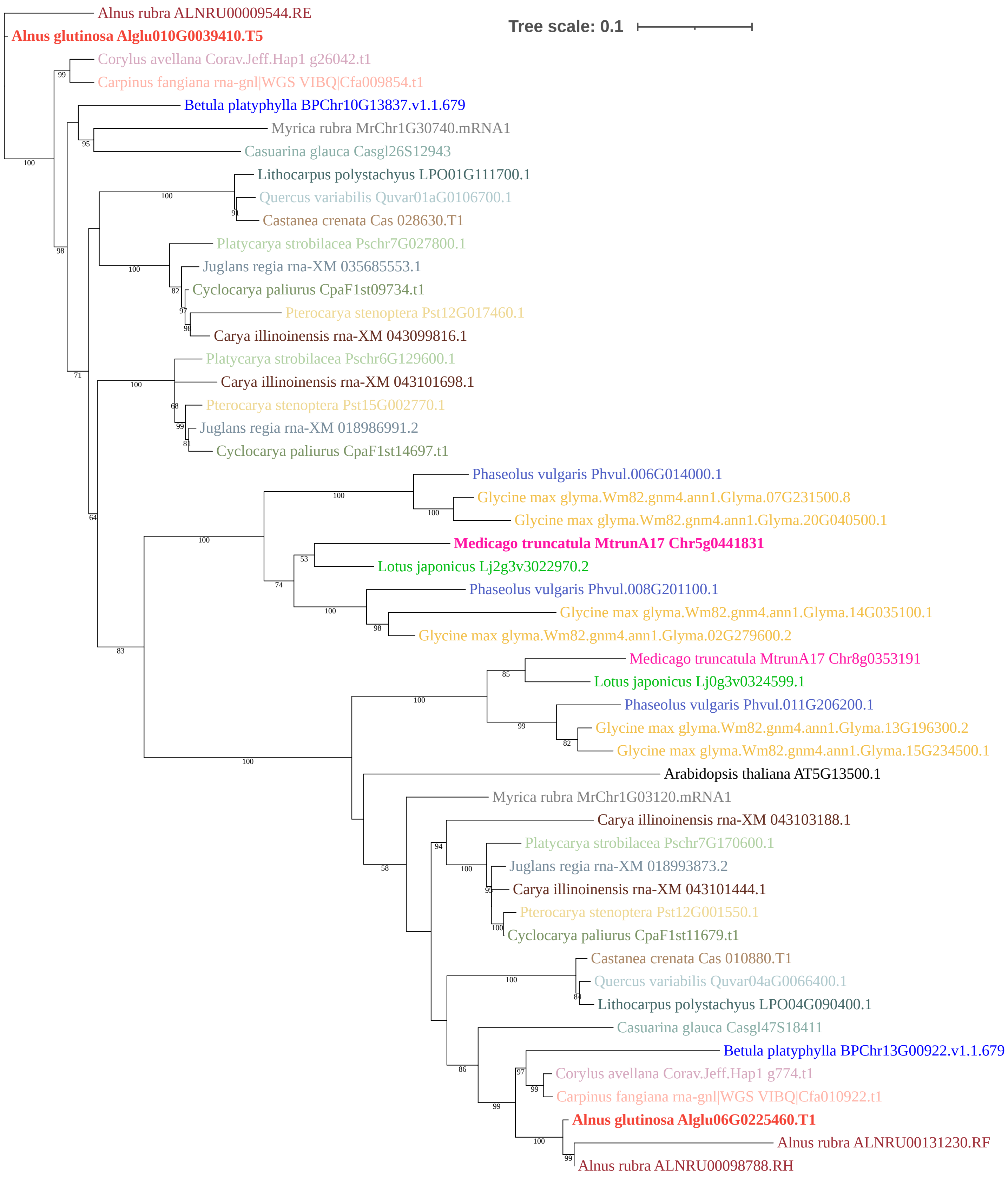

OG0001998:nitrogen nutrition locus 1/FLOWERING LOCUS T

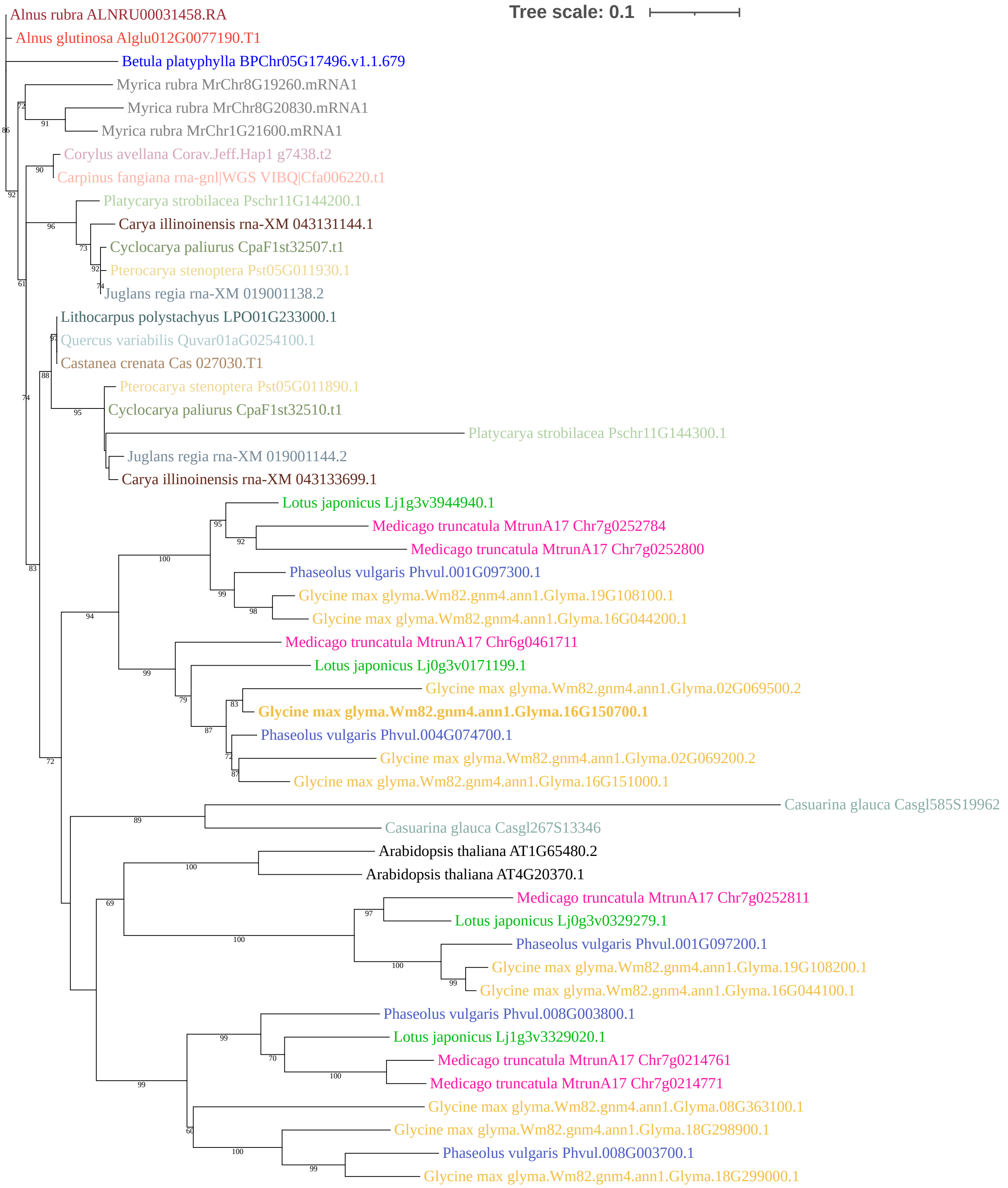

OG0002040:β-EXPANSIN B2

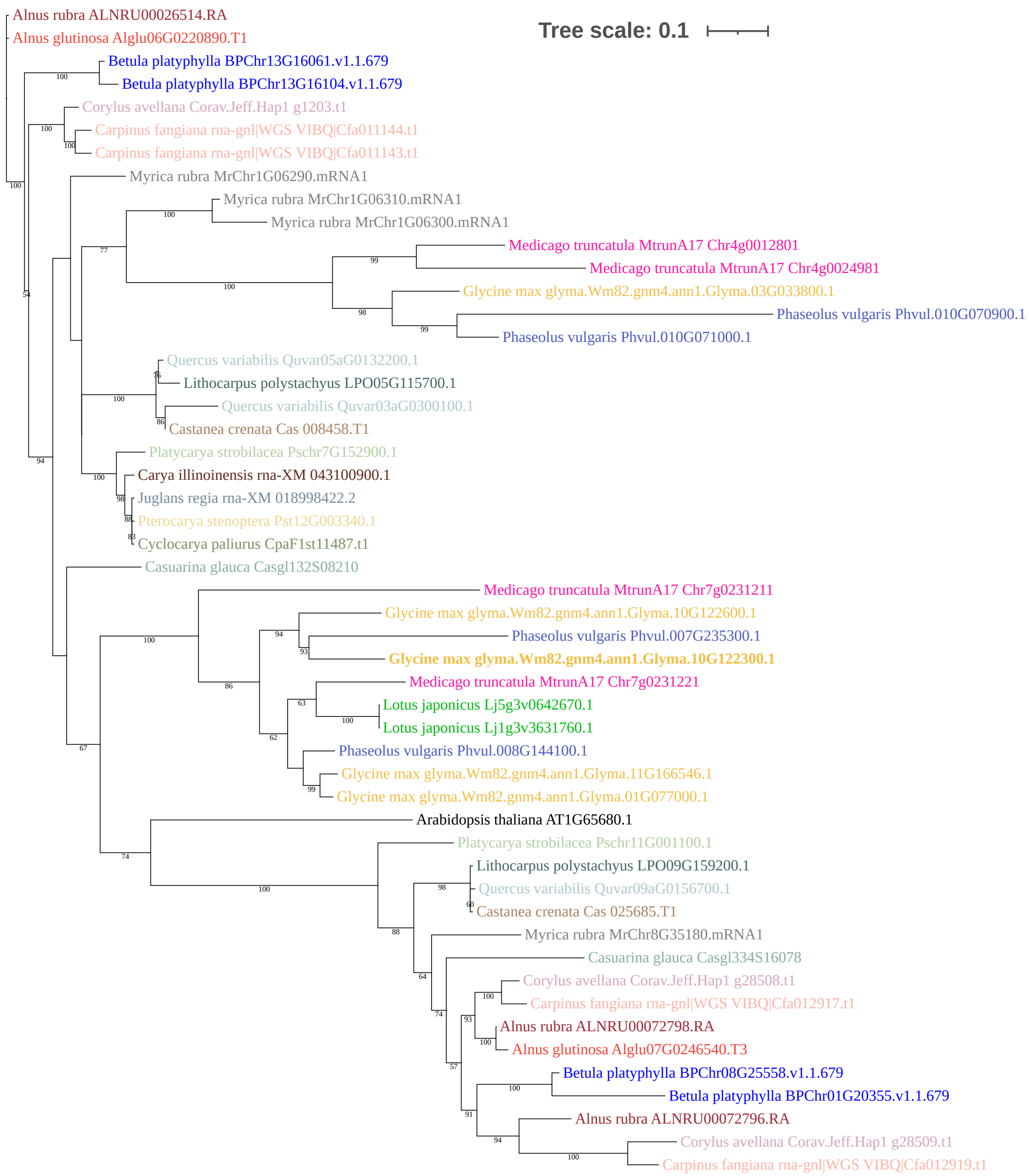

OG0002070:E3 ubiquitin ligase

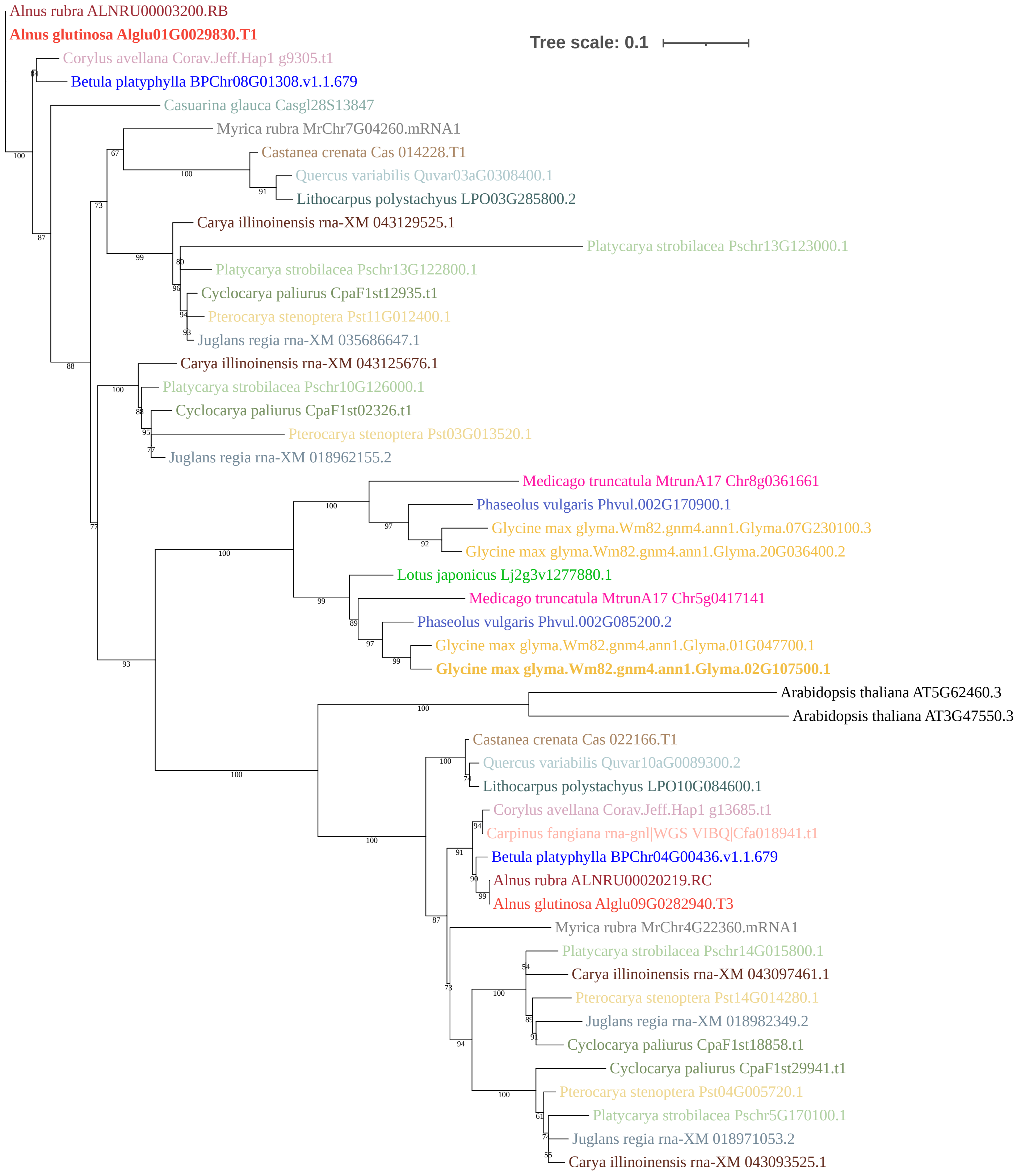

OG0002086:synaptotagmin 2

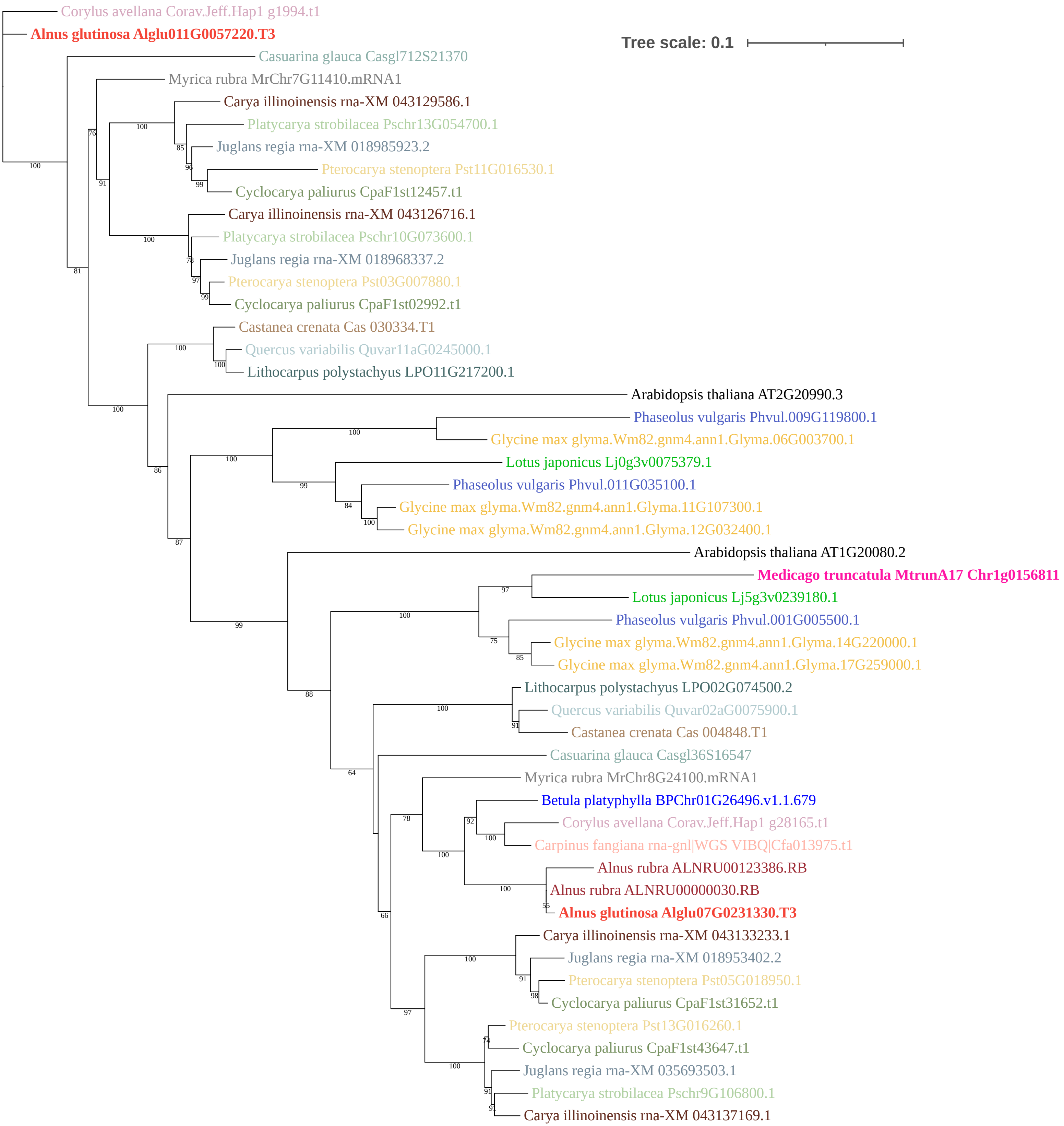

OG0002163:Nitrogen FixationSPECIFICITY 2

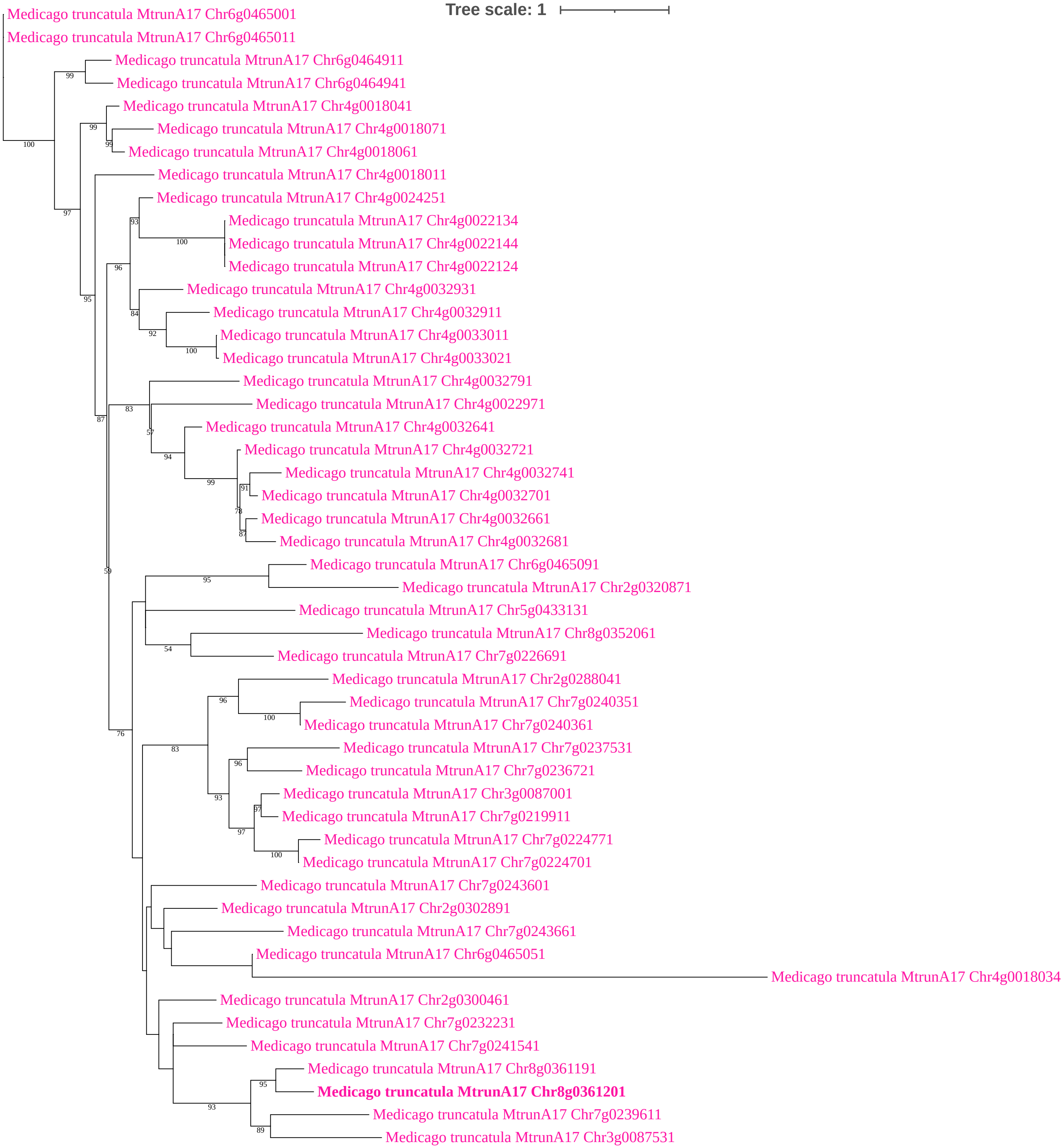

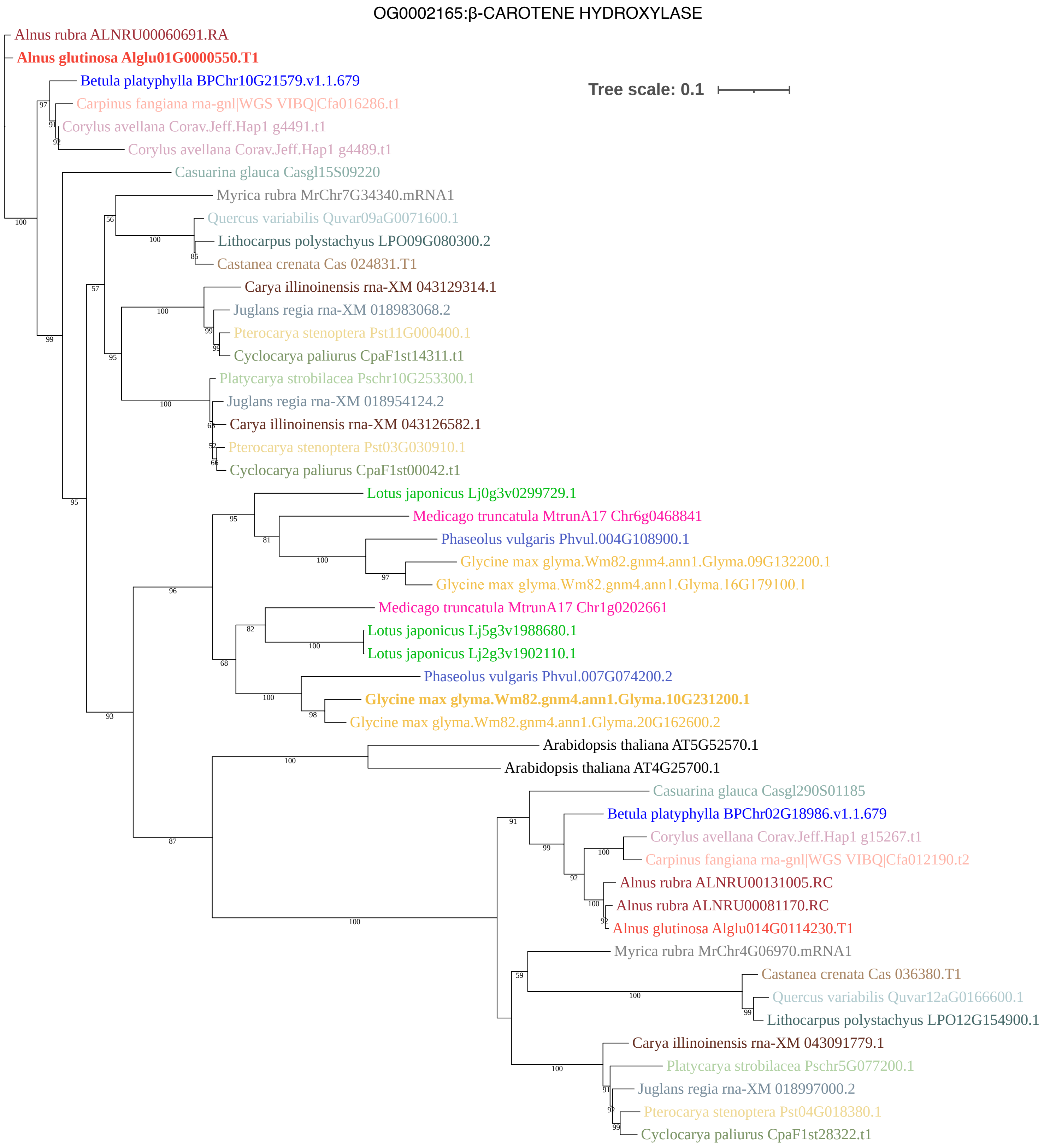
OG0002165:β-CAROTENE HYDROXYLASE
Tree scale: 0.1
Alnus rubra ALNRU00060691.RA
Alnus glutinosa Alglu01G0000550.T1
Betula platyphylla BPChr10G21579.v1.1.679
Carpinus fangiana rna-gnl|WGS VIBQ|Cfa016286.t1
Corylus avellana Corav.Jeff.Hap1 g4491.t1
Corylus avellana Corav.Jeff.Hap1 g4489.t1
Casuarina glauca Casgl15S09220
Myrica rubra MrChr7G34340.mRNA1
Quercus variabilis Quvar09aG0071600.1
Lithocarpus polystachyus LPO09G080300.2
Castanea crenata Cas 024831.T1
Carya illinoinensis rna-XM 043129314.1
Juglans regia rna-XM 018983068.2
Pterocarya stenoptera Pst11G000400.1
Cyclocarya paliurus CpaF1st14311.t1
Platycarya strobilacea Pschr10G253300.1
Juglans regia rna-XM 018954124.2
Carya illinoinensis rna-XM 043126582.1
Pterocarya stenoptera Pst03G030910.1
Cyclocarya paliurus CpaF1st00042.t1
Lotus japonicus Lj0g3v0299729.1
Medicago truncatula MtrunA17 Chr6g0468841
Phaseolus vulgaris Phvul.004G108900.1
Glycine max glyma.Wm82.gnm4.ann1.Glyma.09G132200.1
Glycine max glyma.Wm82.gnm4.ann1.Glyma.16G179100.1
Medicago truncatula MtrunA17 Chr1g0202661
Lotus japonicus Lj5g3v1988680.1
Lotus japonicus Lj2g3v1902110.1
Phaseolus vulgaris Phvul.007G074200.2
Glycine max glyma.Wm82.gnm4.ann1.Glyma.10G231200.1
Glycine max glyma.Wm82.gnm4.ann1.Glyma.20G162600.2
Arabidopsis thaliana AT5G52570.1
Arabidopsis thaliana AT4G25700.1
Casuarina glauca Casgl290S01185
Betula platyphylla BPChr02G18986.v1.1.679
Corylus avellana Corav.Jeff.Hap1 g15267.t1
Carpinus fangiana rna-gnl|WGS VIBQ|Cfa012190.t2
Alnus rubra ALNRU00131005.RC
Alnus rubra ALNRU00081170.RC
Alnus glutinosa Alglu014G0114230.T1
Myrica rubra MrChr4G06970.mRNA1
Castanea crenata Cas 036380.T1
Quercus variabilis Quvar12aG0166600.1
Lithocarpus polystachyus LPO12G154900.1
Carya illinoinensis rna-XM 043091779.1
Platycarya strobilacea Pschr5G077200.1
Juglans regia rna-XM 018997000.2
Pterocarya stenoptera Pst04G018380.1
Cyclocarya paliurus CpaF1st28322.t1

OG0002178:ZINC IRON PERMEASE 6

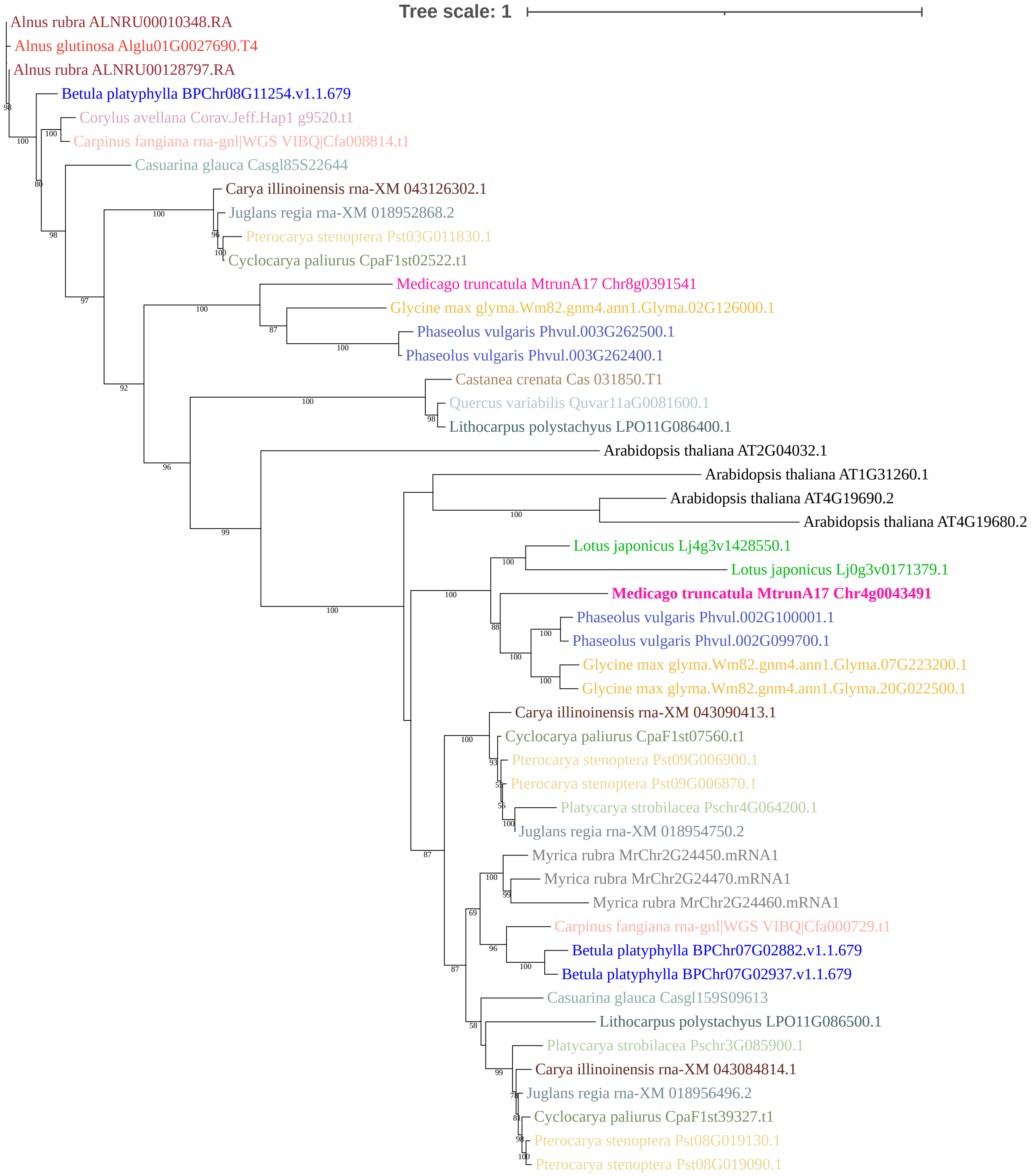

OG0002237:Mtnodulin 19

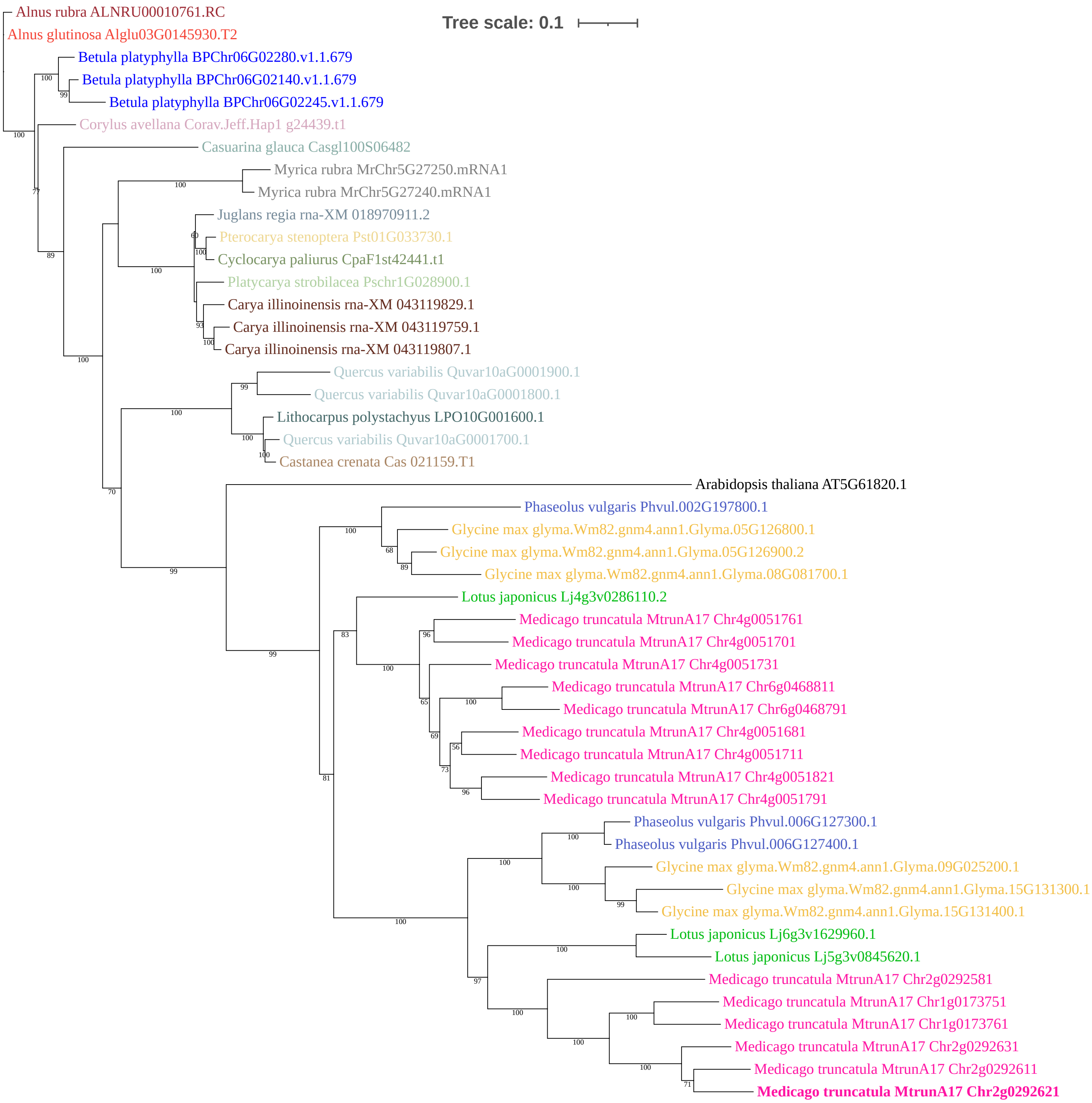

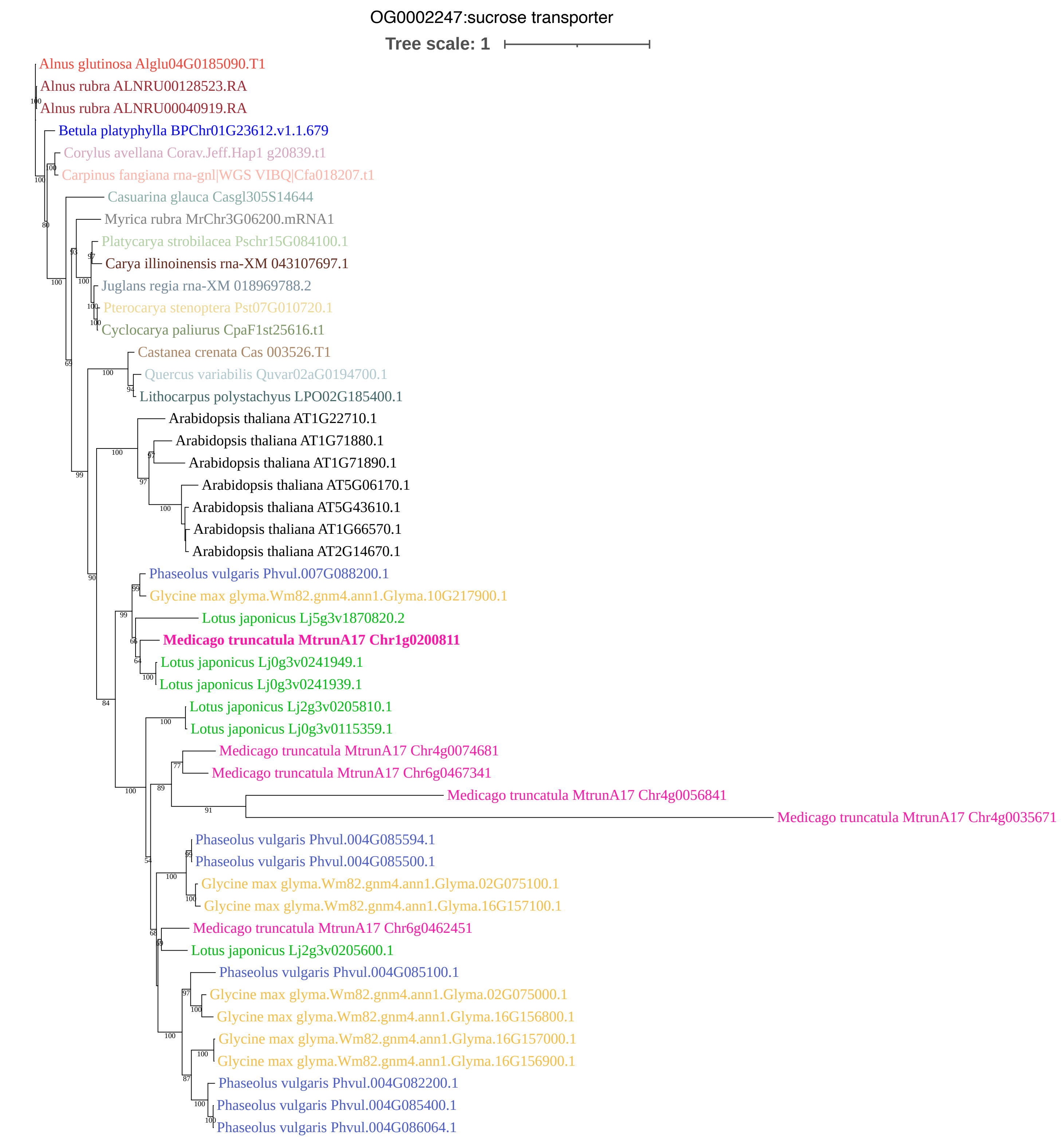

OG0002247:sucrose transporter
Tree scale: 1
Alnus glutinosa Alglu04G0185090.T1
Alnus rubra ALNRU00128523.RA
Alnus rubra ALNRU00040919.RA
Betula platyphylla BPChr01G23612.v1.1.679
Corylus avellana Corav.Jeff.Hap1 g20839.t1
Carpinus fangiana rna-gnl|WGS VIBQ|Cfa018207.t1
Casuarina glauca Casgl305S14644
Myrica rubra MrChr3G06200.mRNA1
Platycarya strobilacea Pschr15G084100.1
Carya illinoinensis rna-XM 043107697.1
Juglans regia rna-XM 018969788.2
Pterocarya stenoptera Pst07G010720.1
Cyclocarya paliurus CpaF1st25616.t1
Castanea crenata Cas 003526.T1
Quercus variabilis Quvar02aG0194700.1
Lithocarpus polystachyus LPO02G185400.1
Arabidopsis thaliana AT1G22710.1
Arabidopsis thaliana AT1G71880.1
Arabidopsis thaliana AT1G71890.1
Arabidopsis thaliana AT5G06170.1
Arabidopsis thaliana AT5G43610.1
Arabidopsis thaliana AT1G66570.1
Arabidopsis thaliana AT2G14670.1
Phaseolus vulgaris Phvul.007G088200.1
Glycine max glyma.Wm82.gnm4.ann1.Glyma.10G217900.1
Lotus japonicus Lj5g3v1870820.2
Medicago truncatula MtrunA17 Chr1g0200811
Lotus japonicus Lj0g3v0241949.1
Lotus japonicus Lj0g3v0241939.1
Lotus japonicus Lj2g3v0205810.1
Lotus japonicus Lj0g3v0115359.1
Medicago truncatula MtrunA17 Chr4g0074681
Medicago truncatula MtrunA17 Chr6g0467341
Medicago truncatula MtrunA17 Chr4g0056841
Medicago truncatula MtrunA17 Chr4g0035671
Phaseolus vulgaris Phvul.004G085594.1
Phaseolus vulgaris Phvul.004G085500.1
Glycine max glyma.Wm82.gnm4.ann1.Glyma.02G075100.1
Glycine max glyma.Wm82.gnm4.ann1.Glyma.16G157100.1
Medicago truncatula MtrunA17 Chr6g0462451
Lotus japonicus Lj2g3v0205600.1
Phaseolus vulgaris Phvul.004G085100.1
Glycine max glyma.Wm82.gnm4.ann1.Glyma.02G075000.1
Glycine max glyma.Wm82.gnm4.ann1.Glyma.16G156800.1
Glycine max glyma.Wm82.gnm4.ann1.Glyma.16G157000.1
Glycine max glyma.Wm82.gnm4.ann1.Glyma.16G156900.1
Phaseolus vulgaris Phvul.004G082200.1
Phaseolus vulgaris Phvul.004G085400.1
Phaseolus vulgaris Phvul.004G086064.1

OG0002279:ARGONAUTE

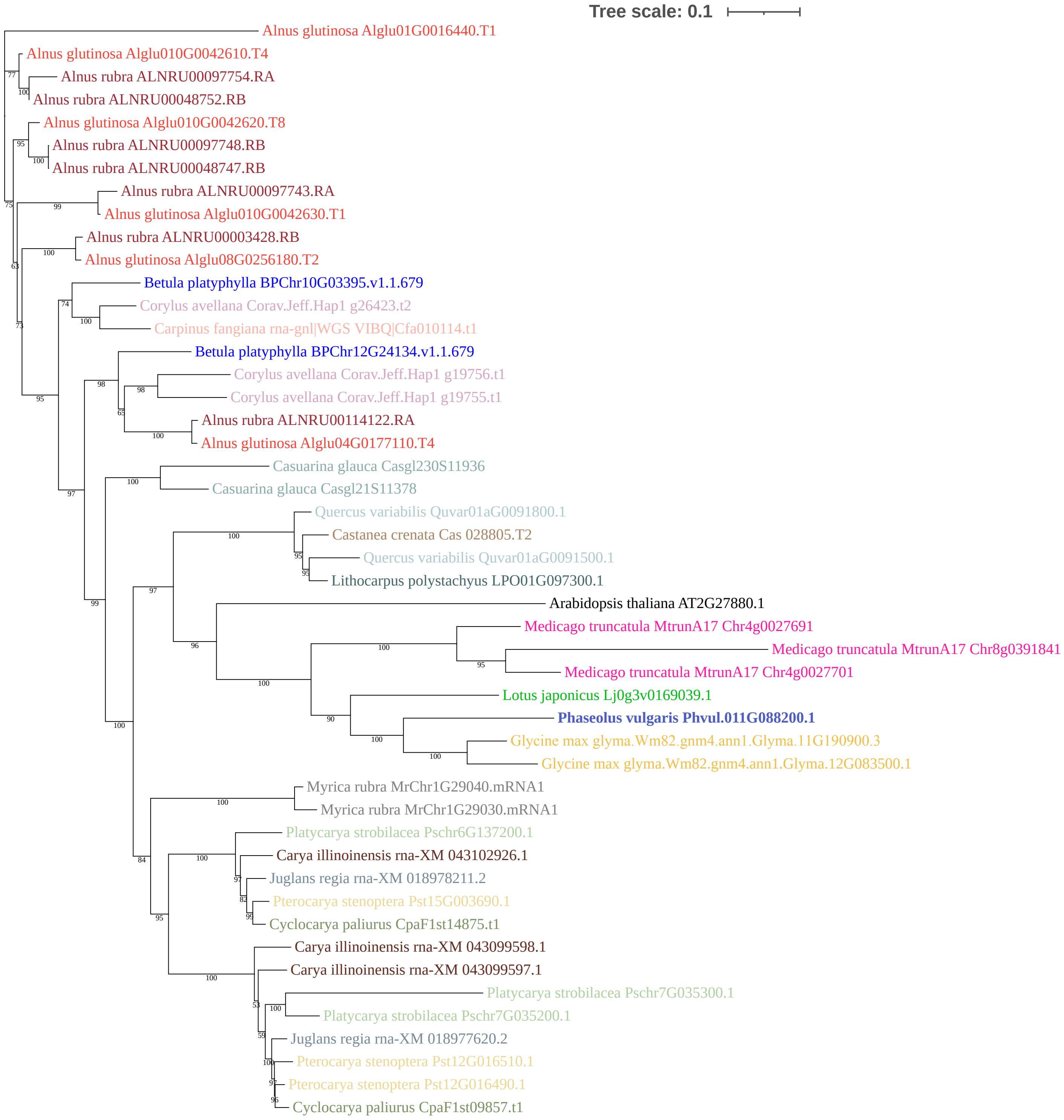

OG0002289:EARLY NODULIN 40

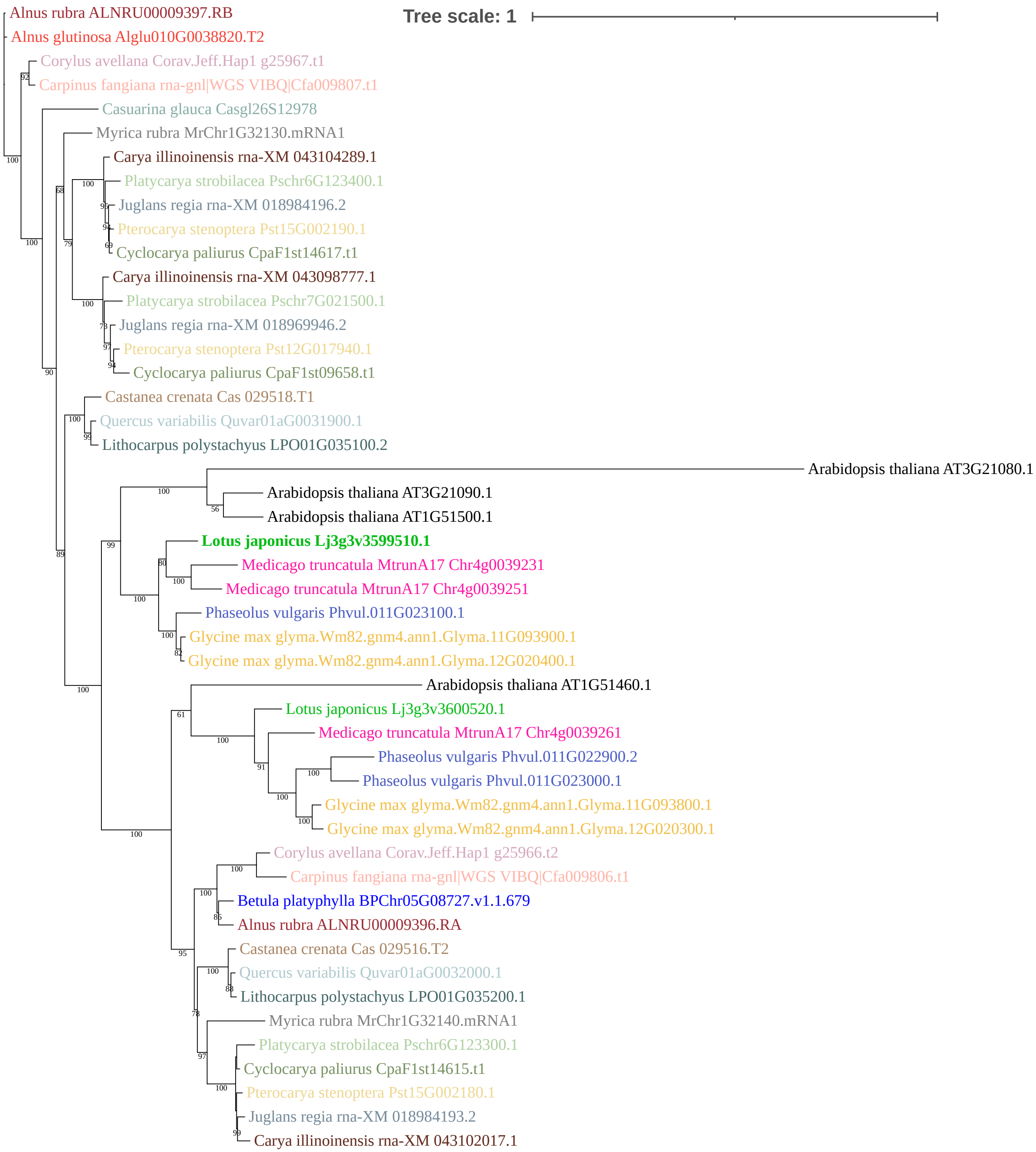

OG0002360:MOLYBDATE TRANSPORTER TYPE 1.2/1.3

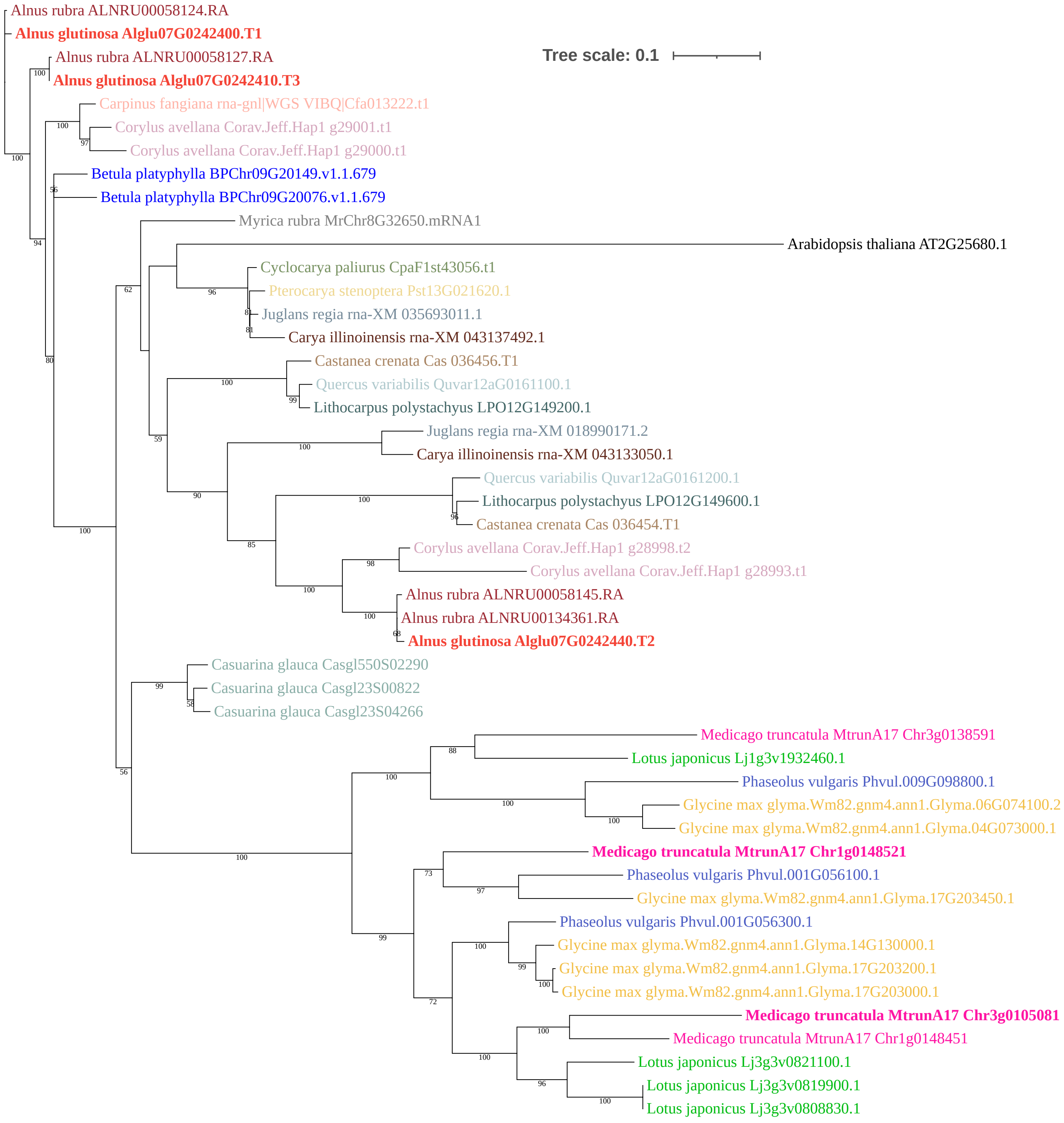

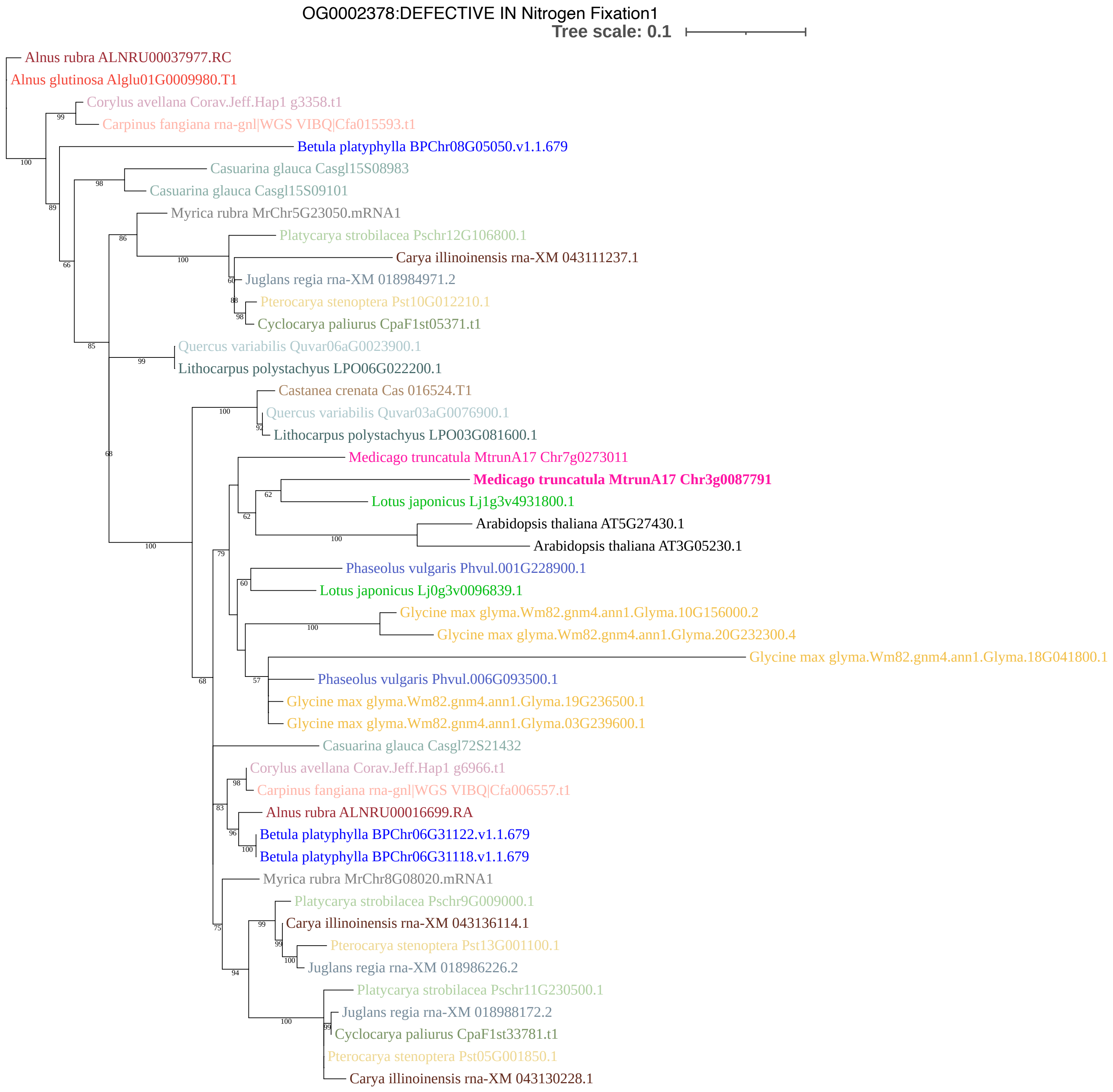
OG0002378:DEFECTIVE IN Nitrogen Fixation1
Tree scale: 0.1
Alnus rubra ALNRU00037977.RC
Alnus glutinosa Alglu01G0009980.T1
Corylus avellana Corav.Jeff.Hap1 g3358.t1
Carpinus fangiana rna-gnl|WGS VIBQ|Cfa015593.t1
Betula platyphylla BPChr08G05050.v1.1.679
Casuarina glauca Casgl15S08983
Casuarina glauca Casgl15S09101
Myrica rubra MrChr5G23050.mRNA1
Platycarya strobilacea Pschr12G106800.1
Carya illinoinensis rna-XM 043111237.1
Juglans regia rna-XM 018984971.2
Pterocarya stenoptera Pst10G012210.1
Cyclocarya paliurus CpaF1st05371.t1
Quercus variabilis Quvar06aG0023900.1
Lithocarpus polystachyus LPO06G022200.1
Castanea crenata Cas 016524.T1
Quercus variabilis Quvar03aG0076900.1
Lithocarpus polystachyus LPO03G081600.1
Medicago truncatula MtrunA17 Chr7g0273011
Medicago truncatula MtrunA17 Chr3g0087791
Lotus japonicus Lj1g3v4931800.1
Arabidopsis thaliana AT5G27430.1
Arabidopsis thaliana AT3G05230.1
Phaseolus vulgaris Phvul.001G228900.1
Lotus japonicus Lj0g3v0096839.1
Glycine max glyma.Wm82.gnm4.ann1.Glyma.10G156000.2
Glycine max glyma.Wm82.gnm4.ann1.Glyma.20G232300.4
Glycine max glyma.Wm82.gnm4.ann1.Glyma.18G041800.1
Phaseolus vulgaris Phvul.006G093500.1
Glycine max glyma.Wm82.gnm4.ann1.Glyma.19G236500.1
Glycine max glyma.Wm82.gnm4.ann1.Glyma.03G239600.1
Casuarina glauca Casgl72S21432
Corylus avellana Corav.Jeff.Hap1 g6966.t1
Carpinus fangiana rna-gnl|WGS VIBQ|Cfa006557.t1
Alnus rubra ALNRU00016699.RA
Betula platyphylla BPChr06G31122.v1.1.679
Betula platyphylla BPChr06G31118.v1.1.679
Myrica rubra MrChr8G08020.mRNA1
Platycarya strobilacea Pschr9G009000.1
Carya illinoinensis rna-XM 043136114.1
Pterocarya stenoptera Pst13G001100.1
Juglans regia rna-XM 018986226.2
Platycarya strobilacea Pschr11G230500.1
Juglans regia rna-XM 018988172.2
Cyclocarya paliurus CpaF1st33781.t1
Pterocarya stenoptera Pst05G001850.1
Carya illinoinensis rna-XM 043130228.1

OG0002448:BYPASS 1.1/1.2

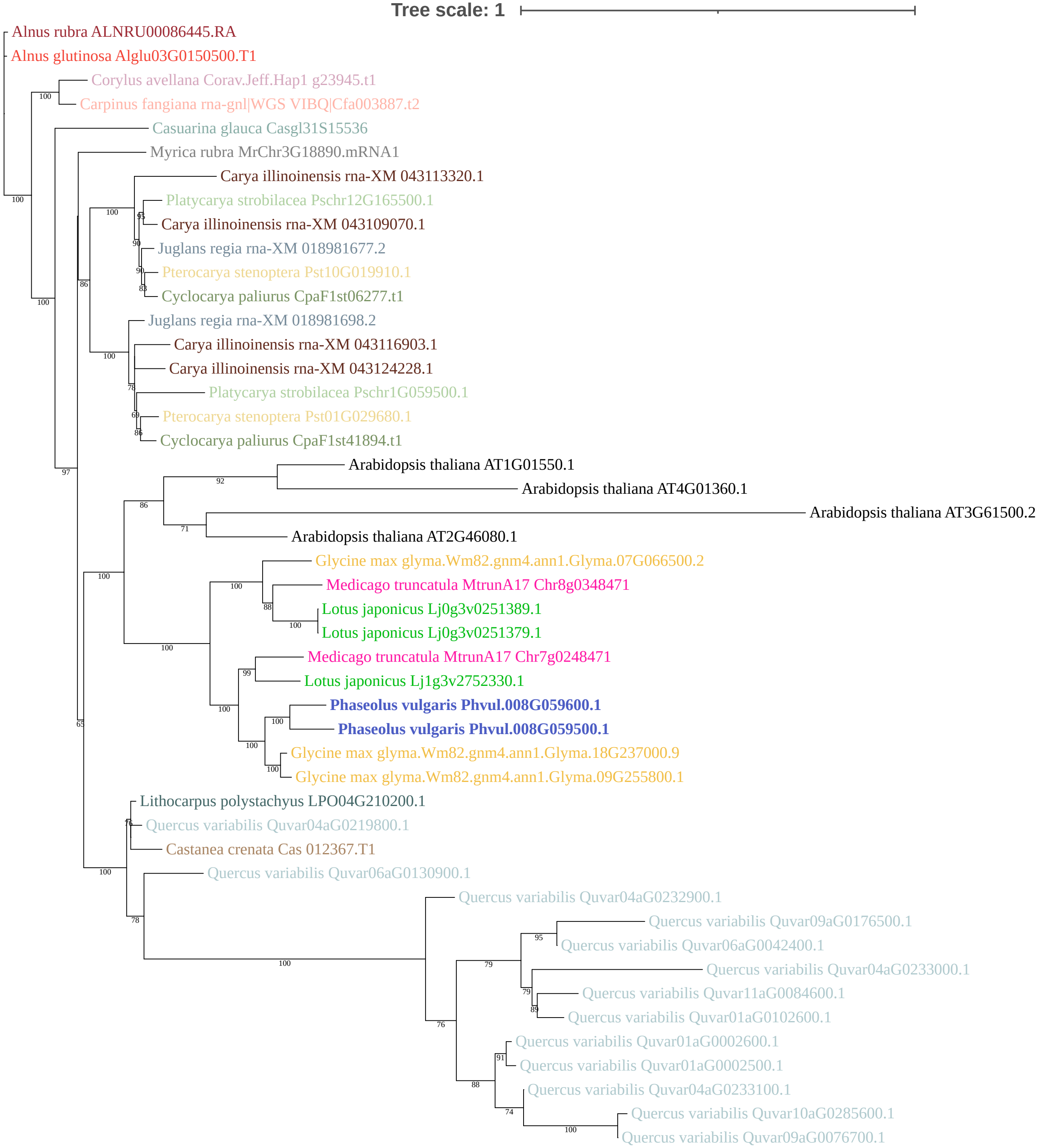

OG0002617:ETHYLENE RESPONSE FACTOR REQUIRED FOR NODULATION2/1

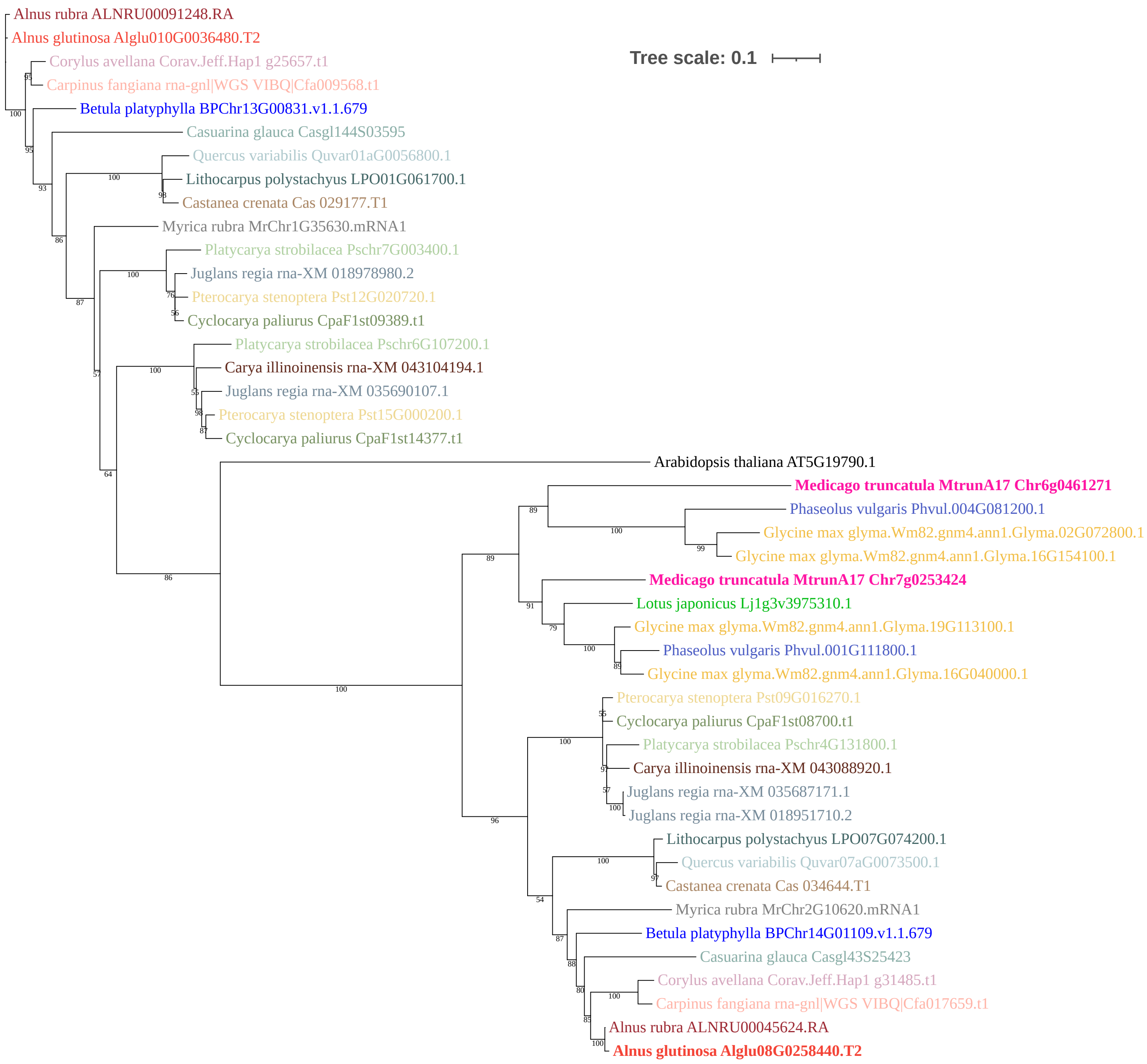

OG0002755:SYMRK INTERACTING E3 UBIQUITIN LIGASE

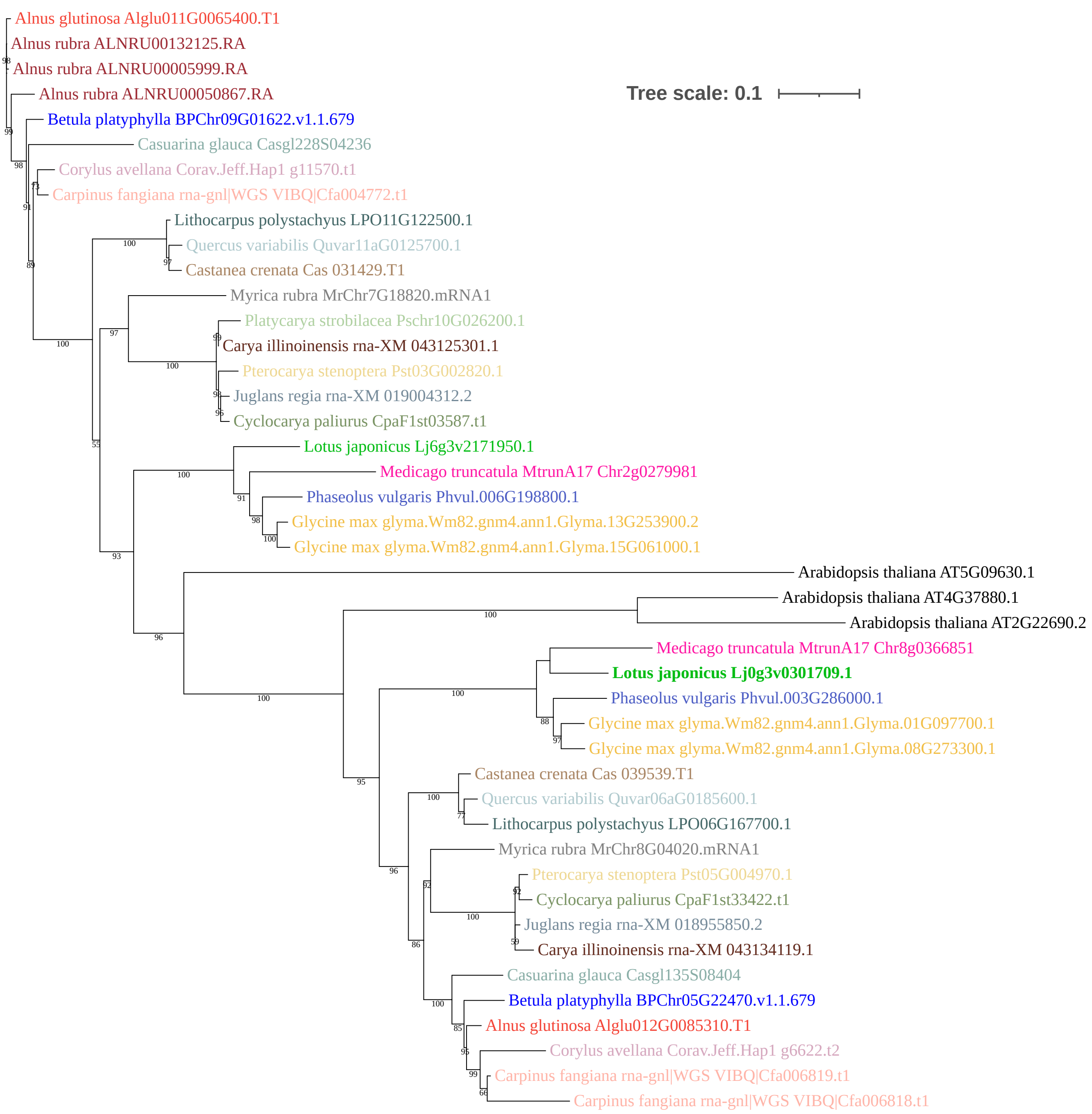

OG0002761:ENT-COPALYL DIPHOSPHATE SYNTHETASE 1

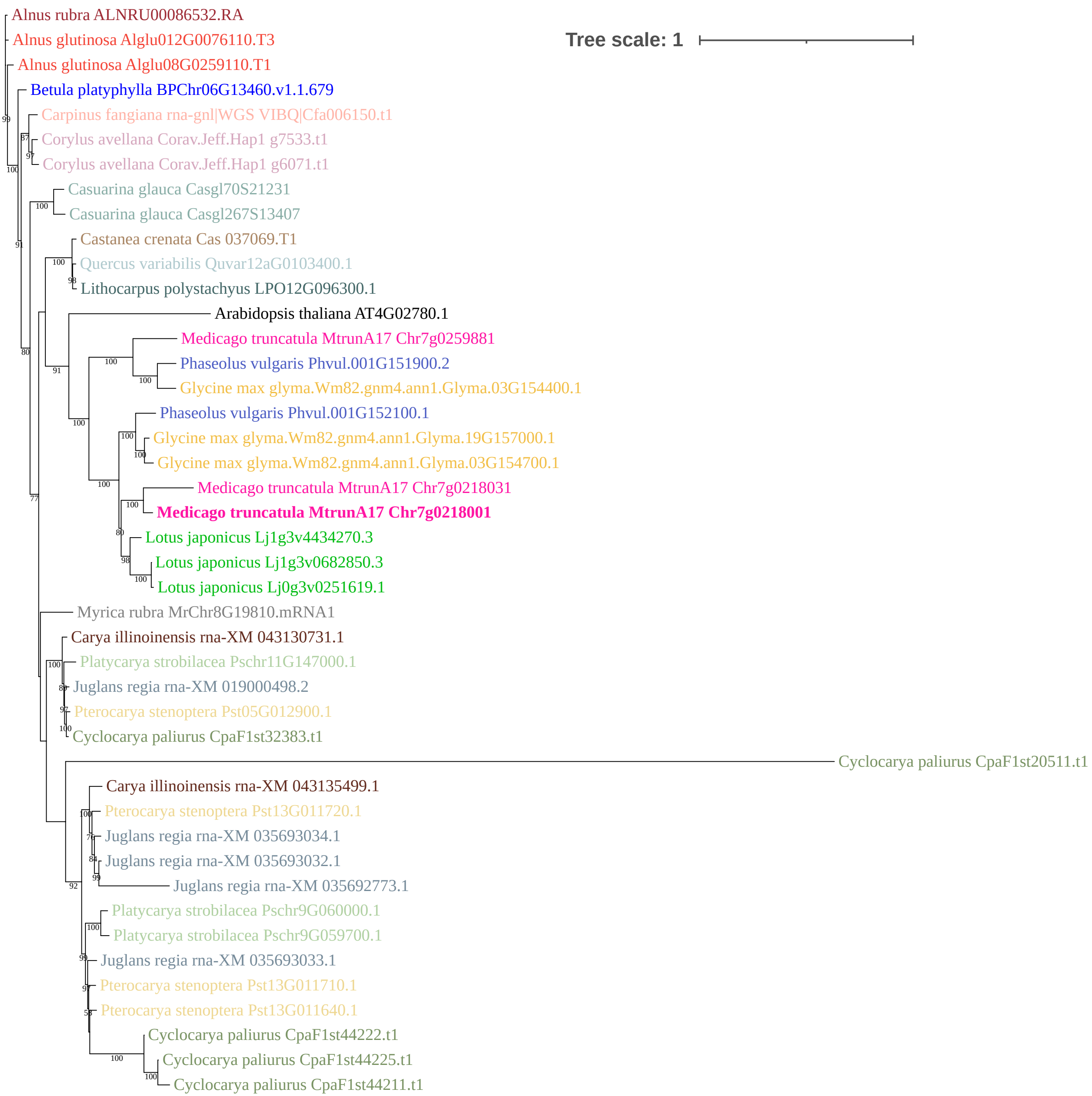

OG0002780:LOB Domain protein 17

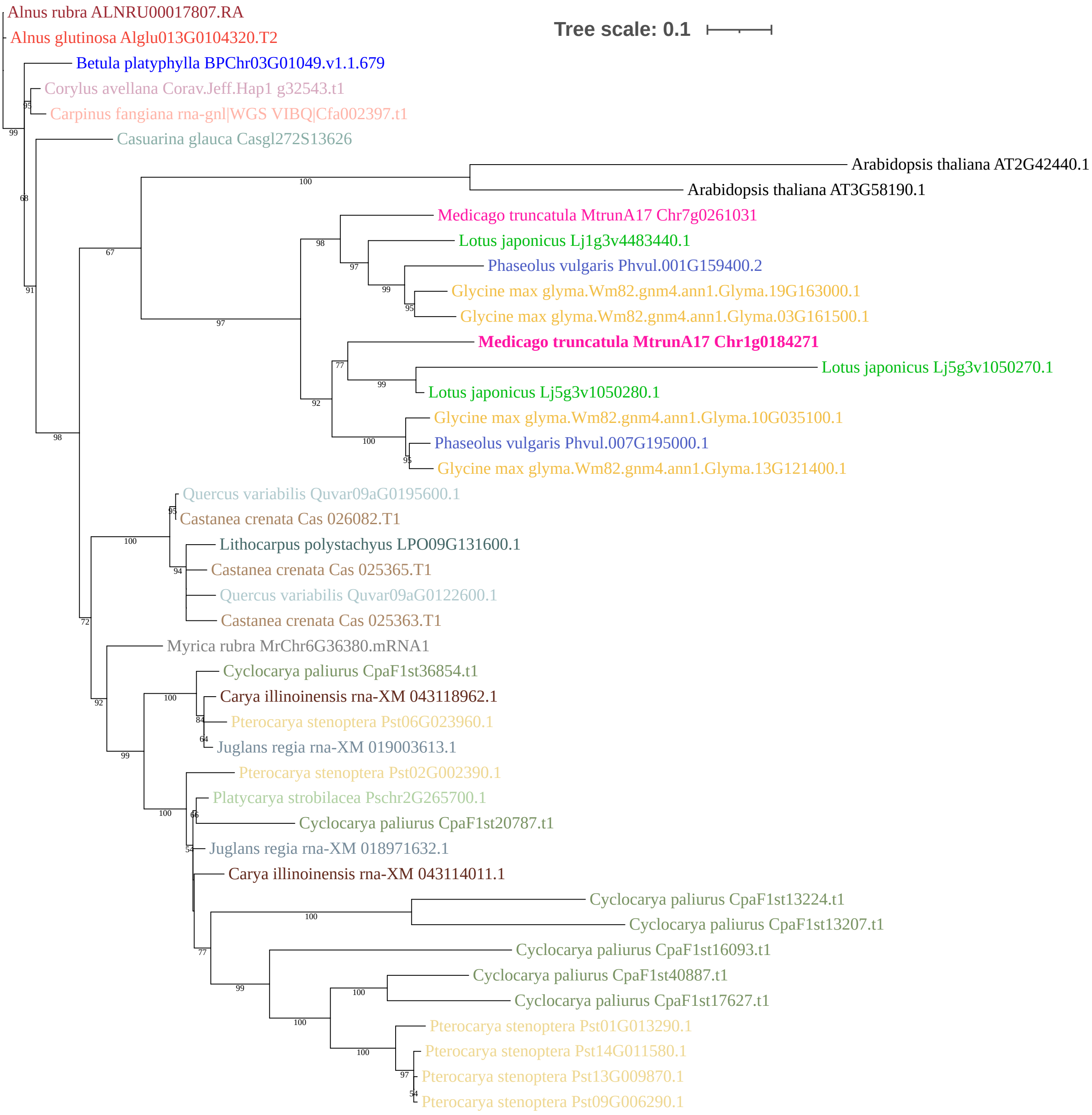

OG0002864:TEOSINTE-BRANCHED1/CYCLOIDEA/PCF

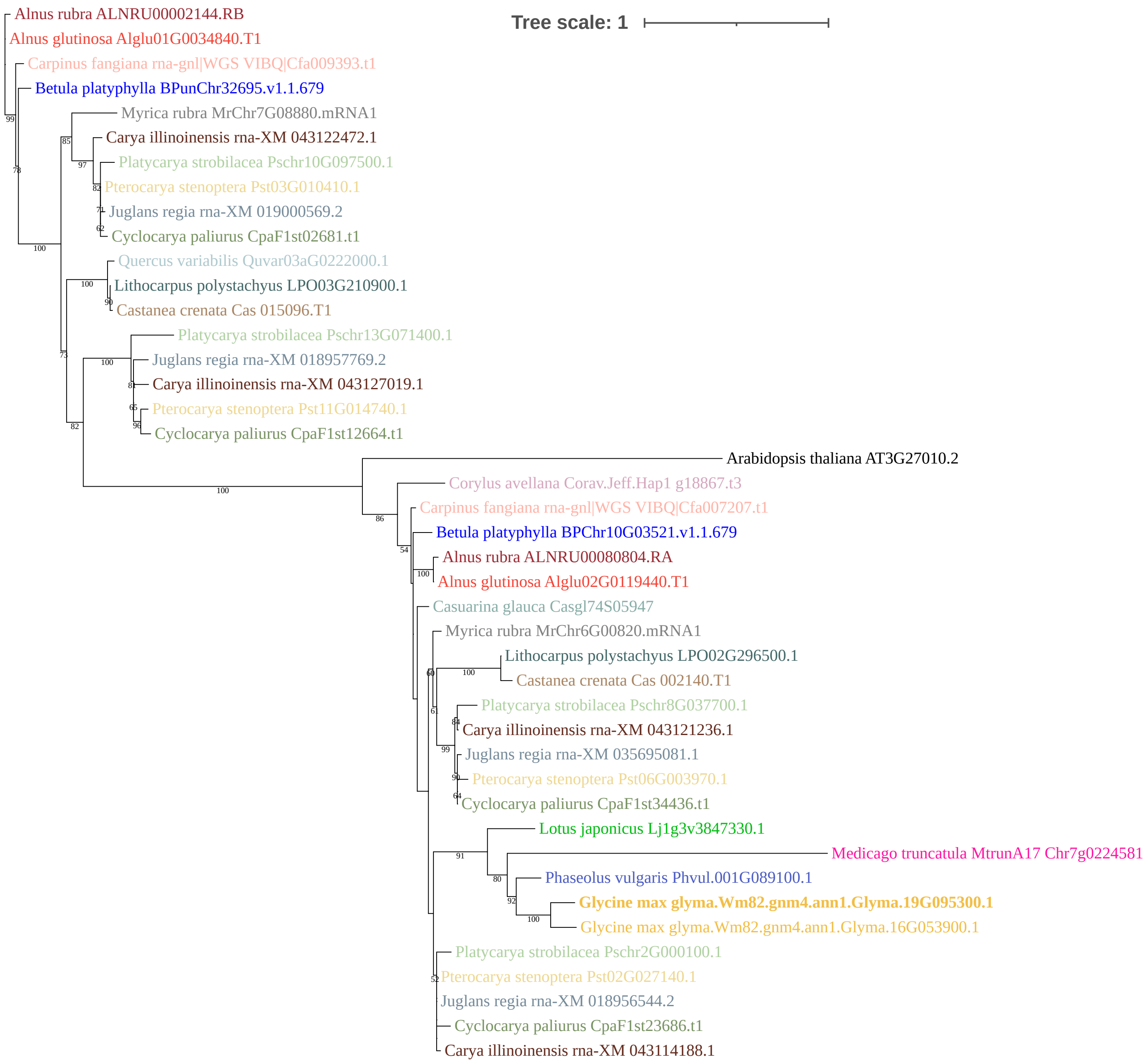

OG0002957:CYSTATHIONE BETASYNTHASE 1

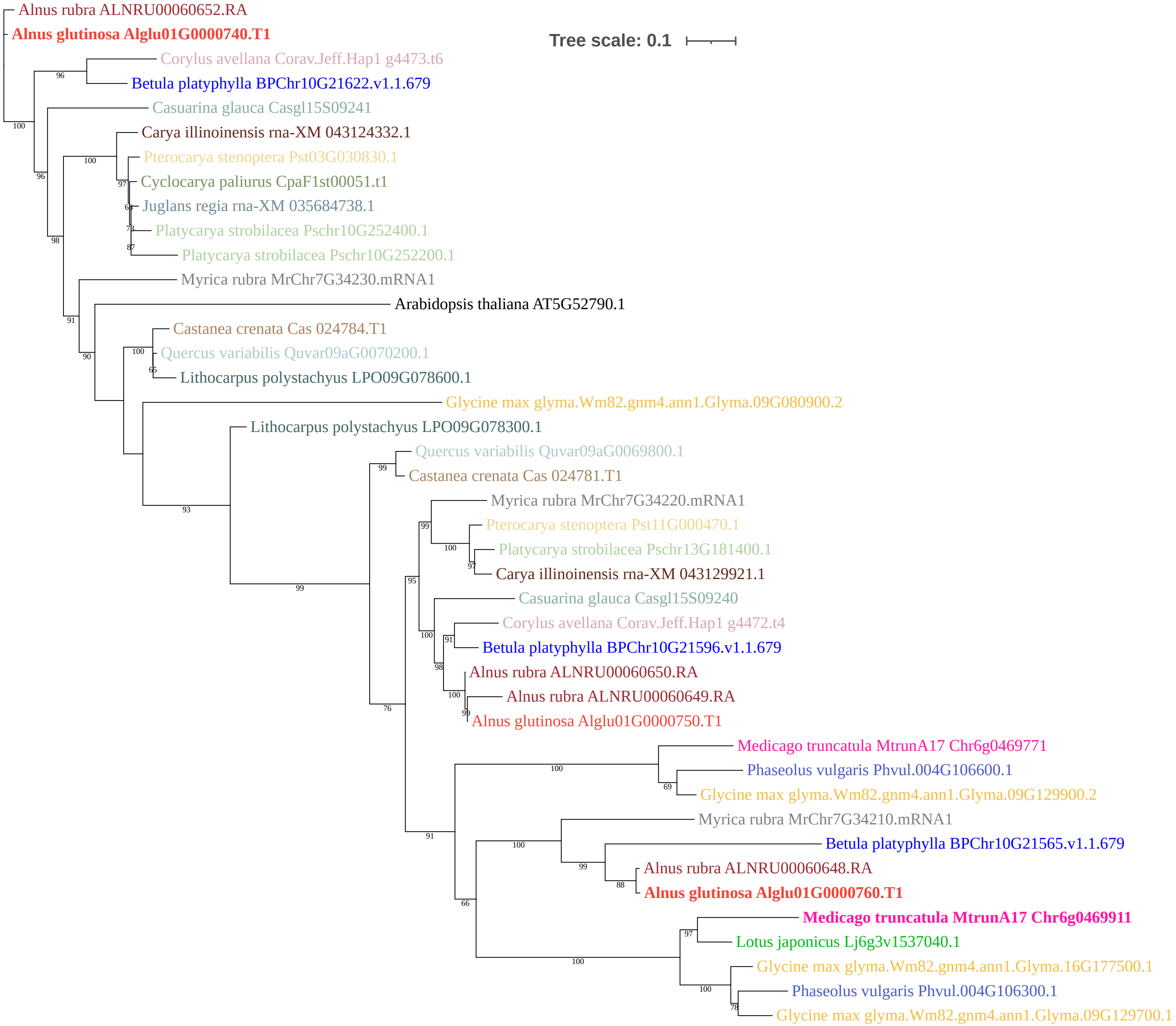

OG0002992:PIN-FORMED auxin efflux carrier component 2

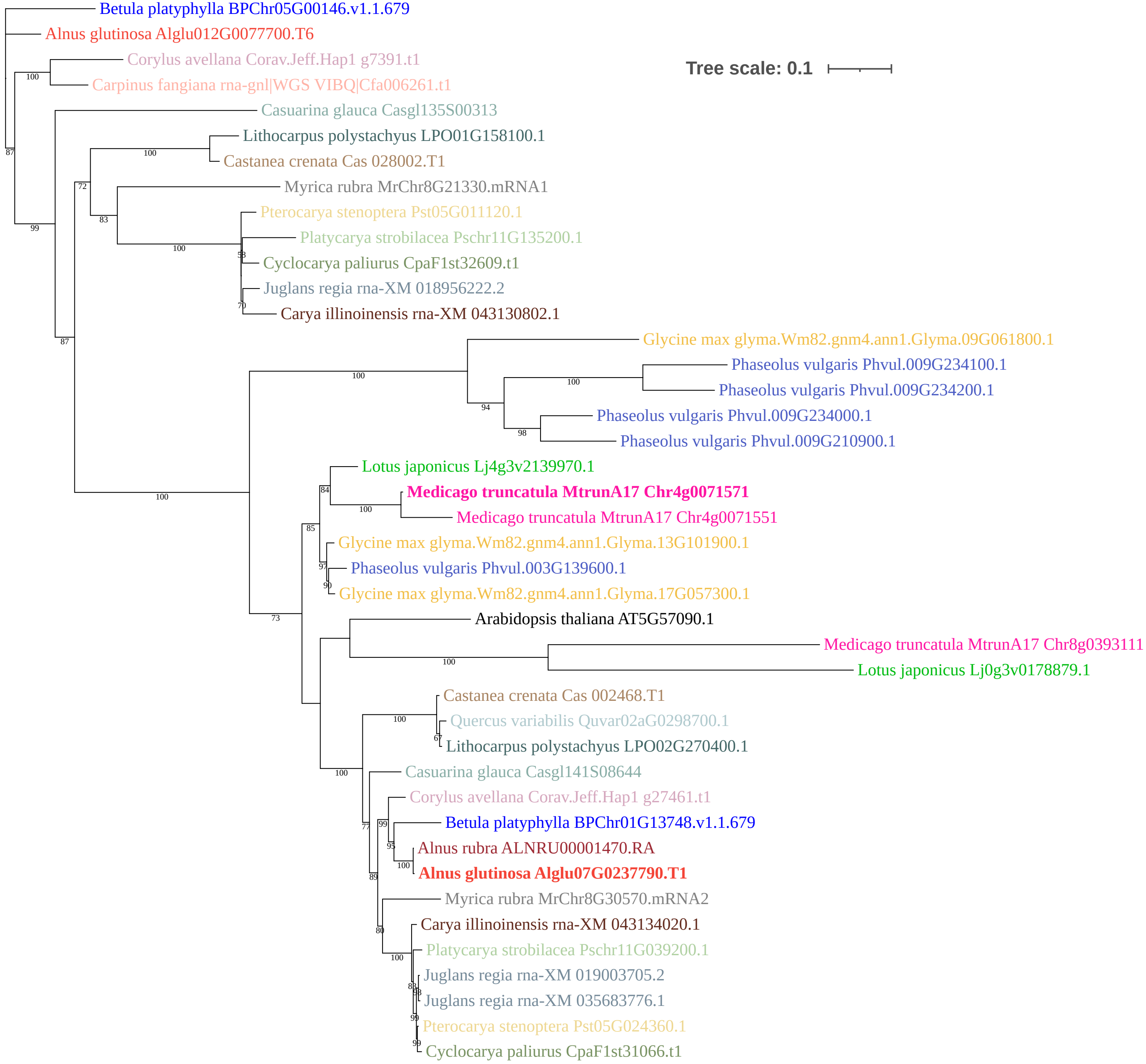

OG0003017:Rhizobial Infection Receptor-like Kinase1

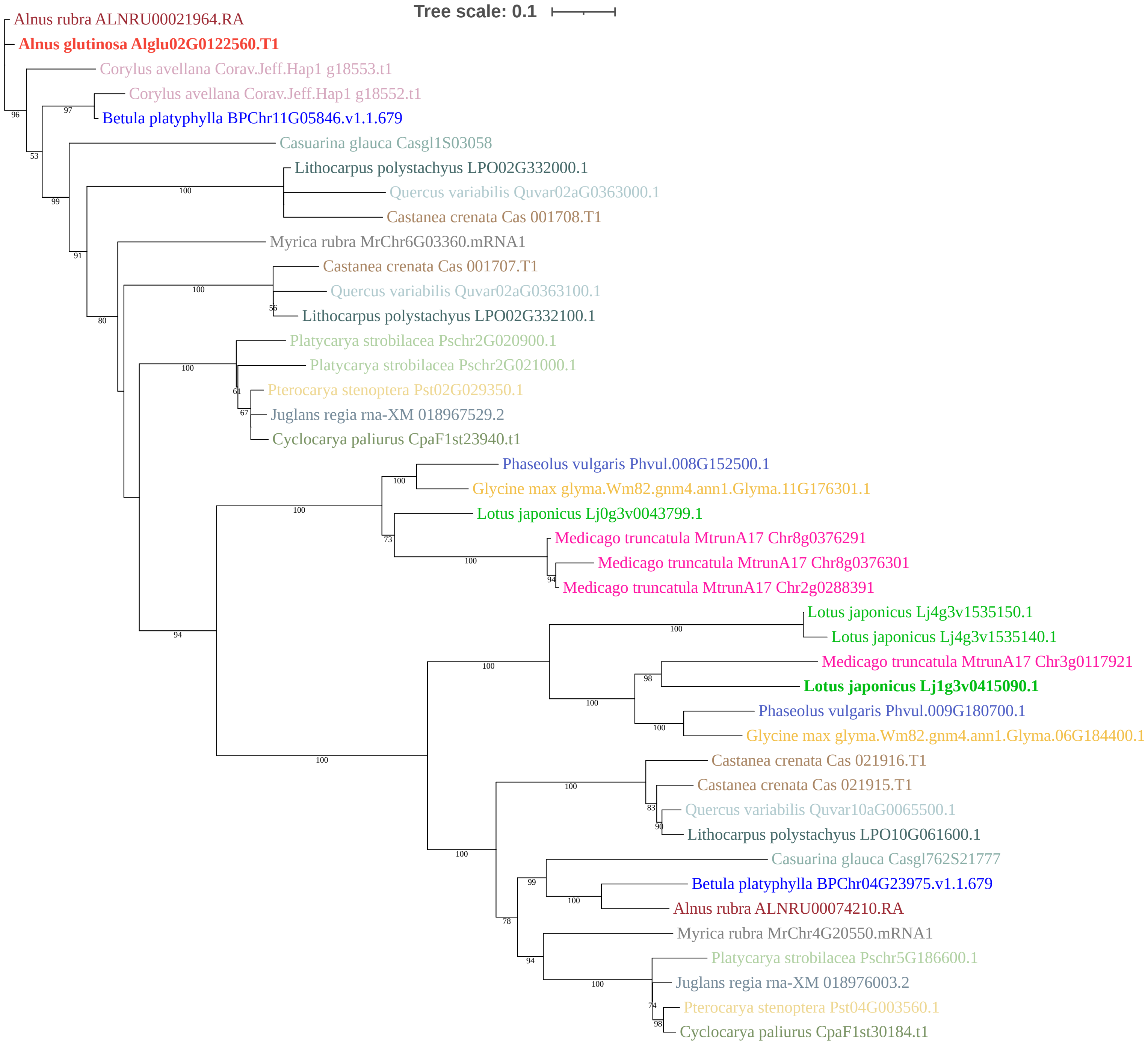

OG0003018:Golven/ Root Growth Factor|Root Meristem Growth Factor(peptide)

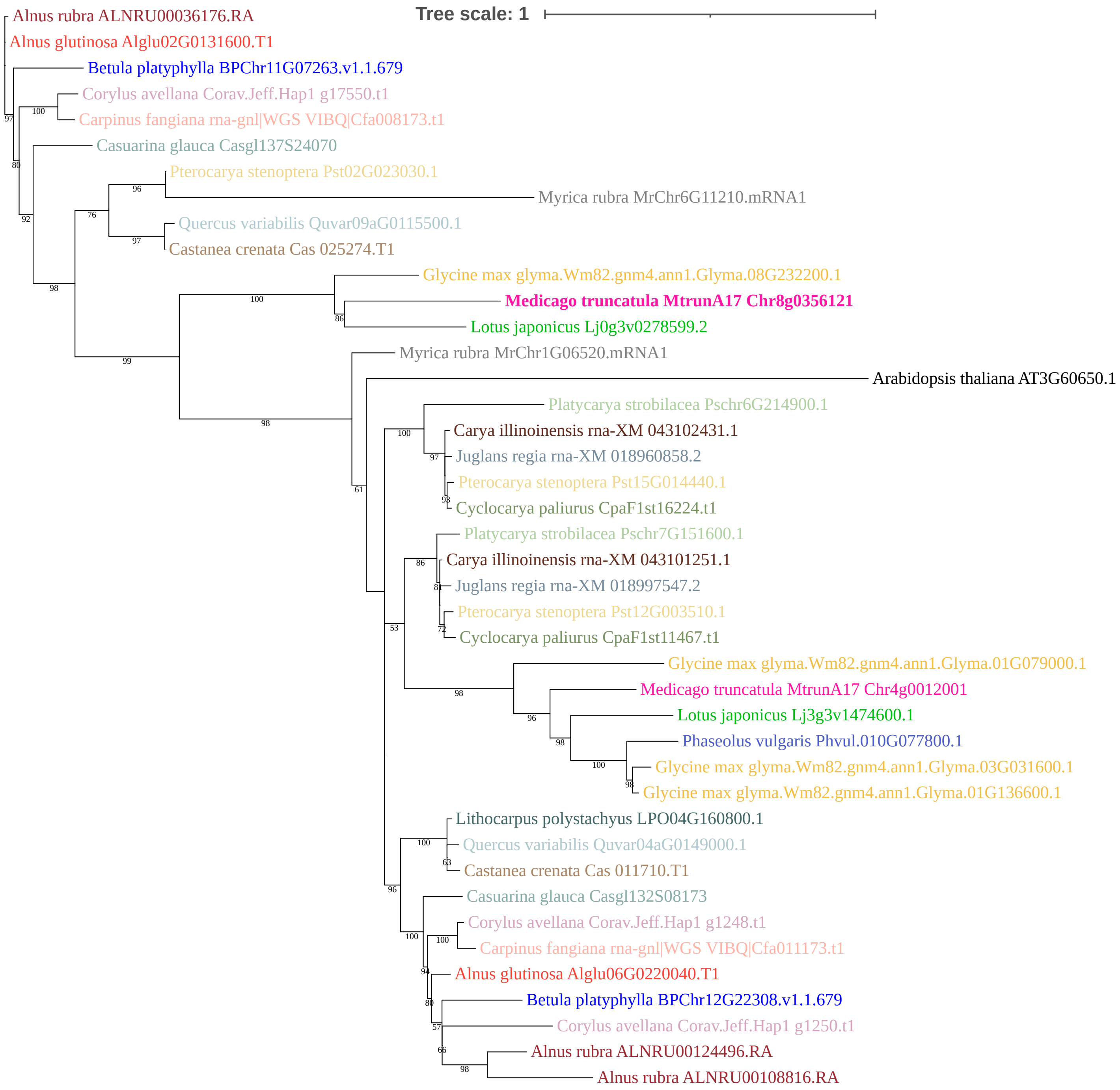

OG0003023:SYNTAXIN OF PLANTS 132

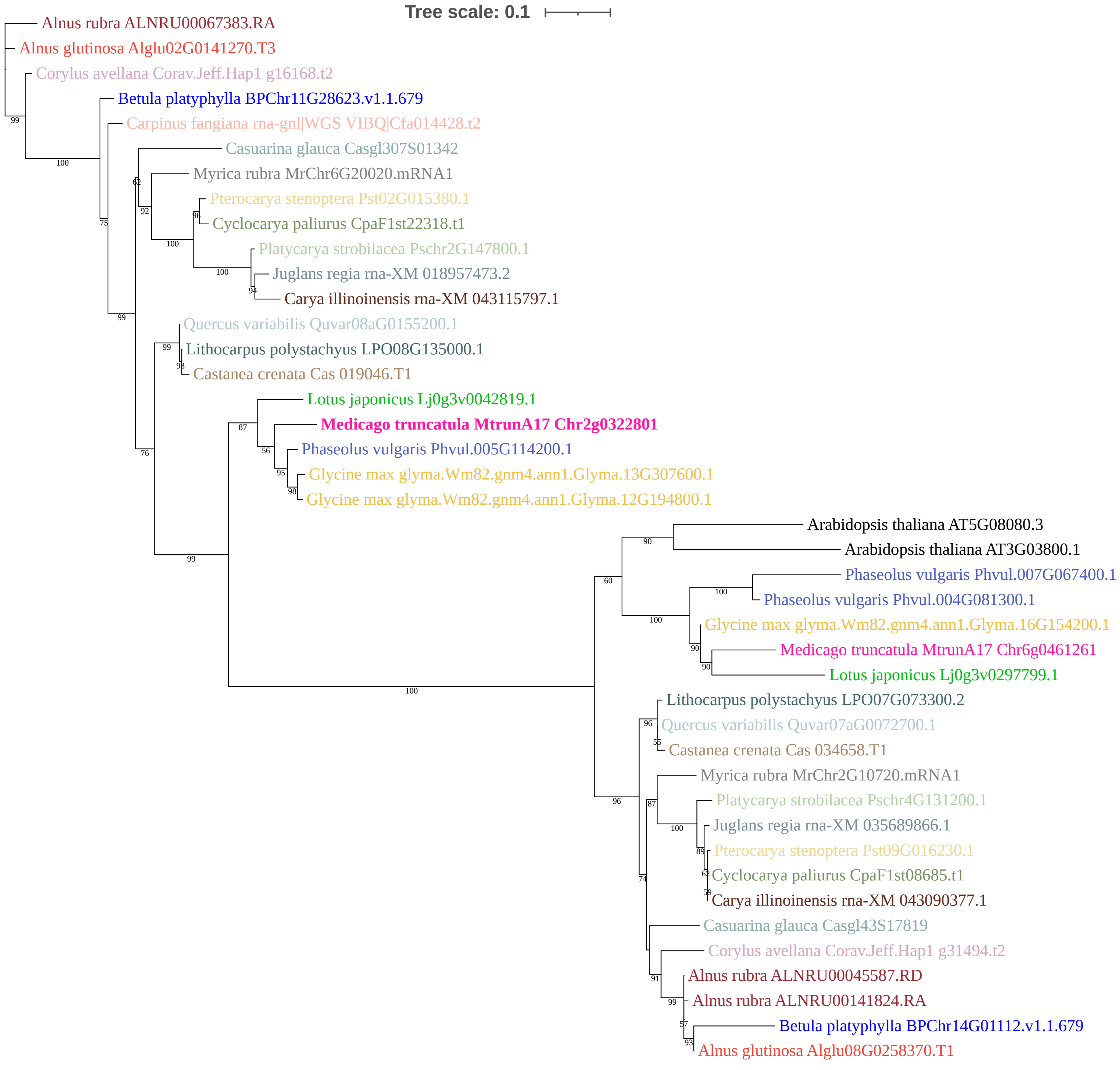

OG0003054:LECTIN NUCLEOTIDE PHOSPHOHYDROLASE

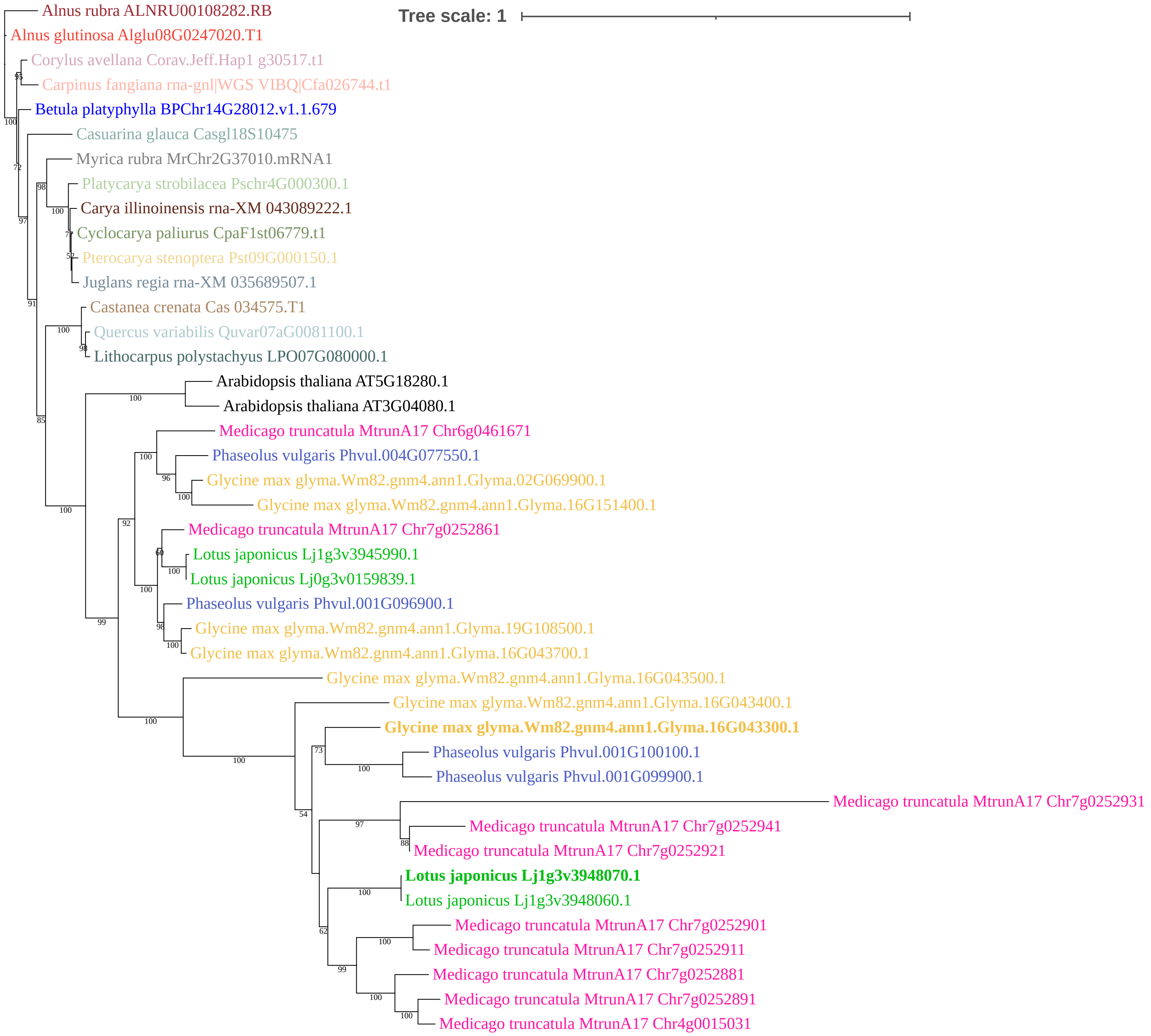

OG0003135:INEFFECTIVE GREENISH NODULE 1

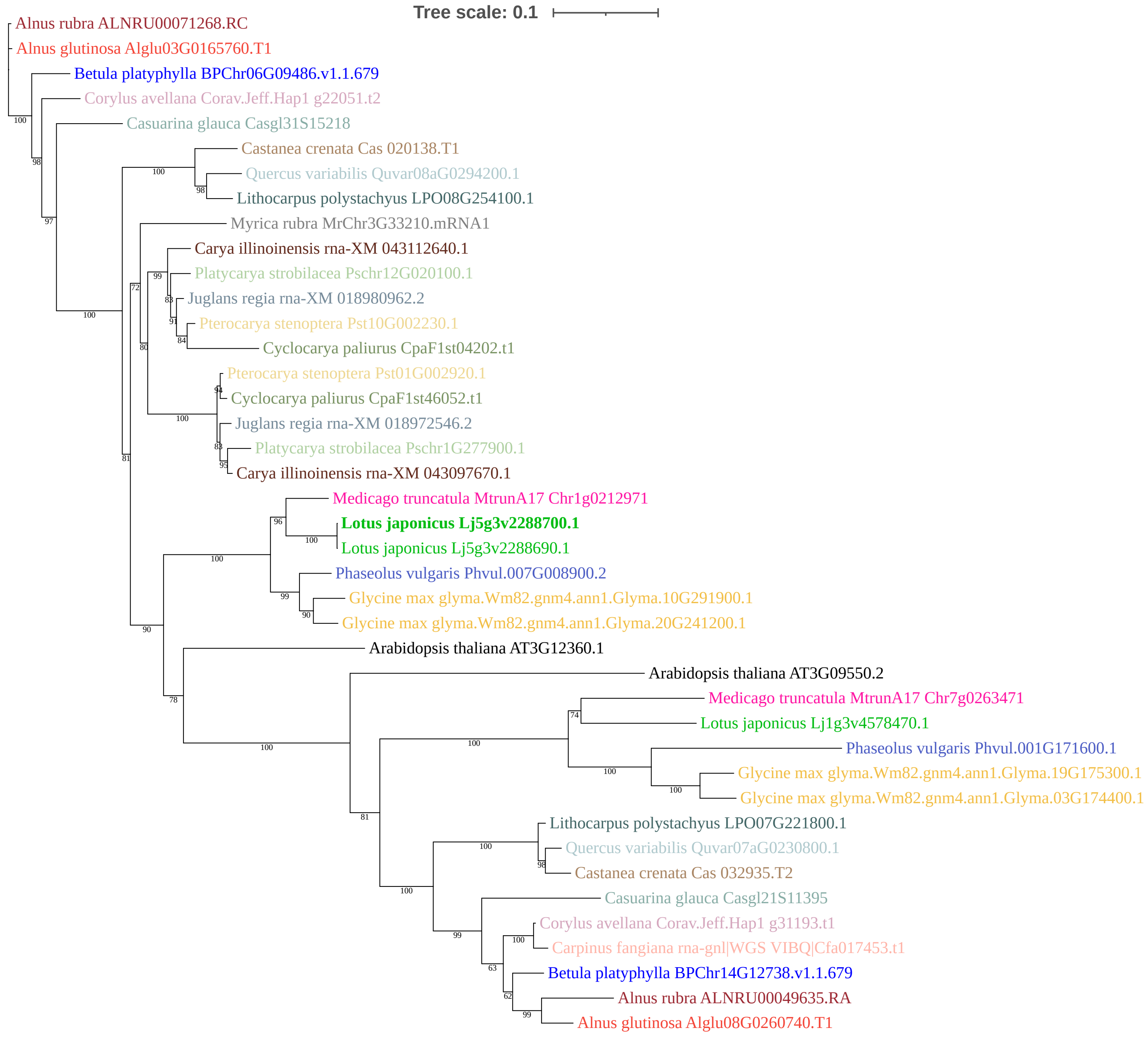

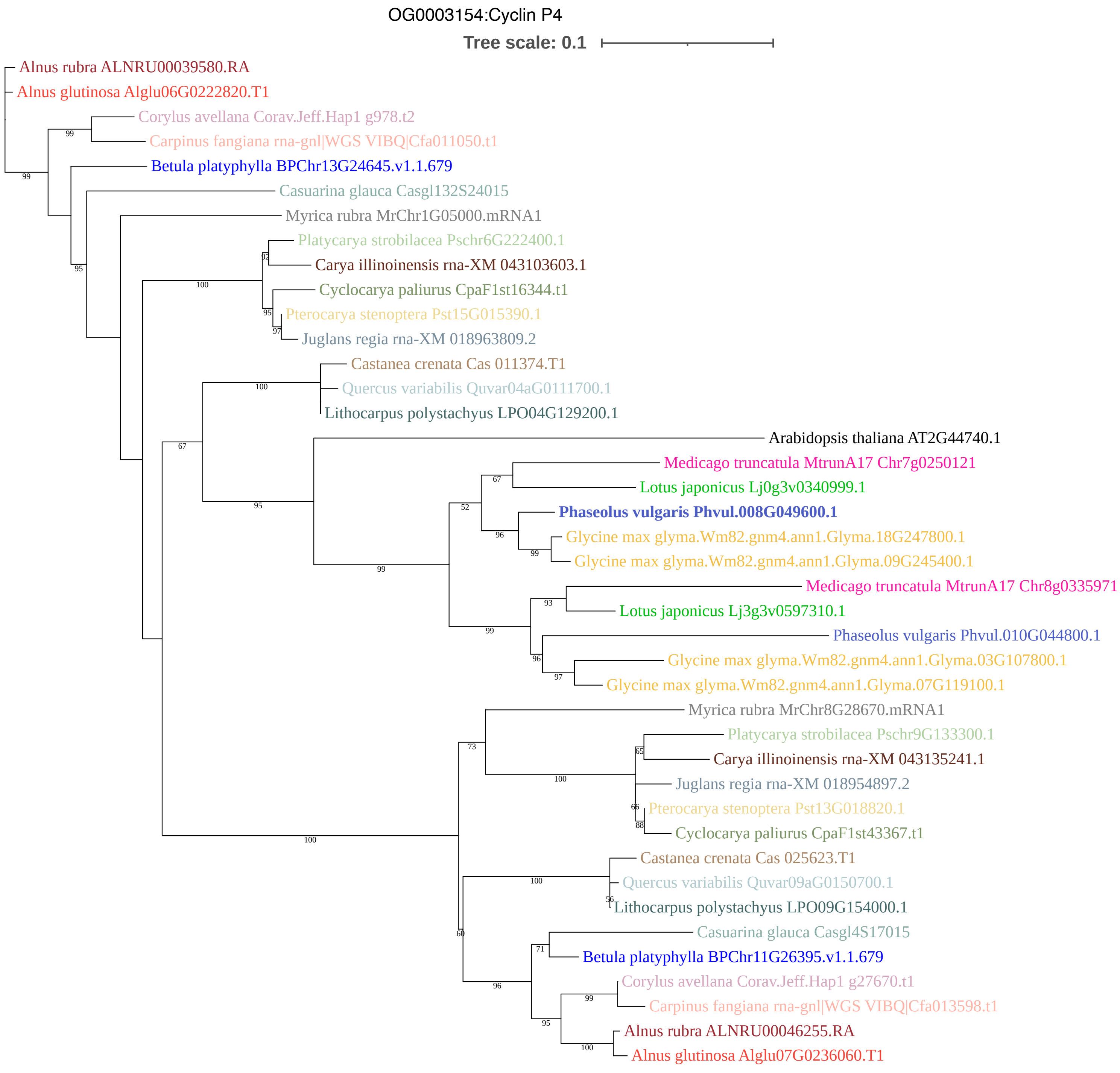
OG0003154:Cyclin P4
Tree scale: 0.1
Alnus rubra ALNRU00039580.RA
Alnus glutinosa Alglu06G0222820.T1
Corylus avellana Corav.Jeff.Hap1 g978.t2
Carpinus fangiana rna-gnl|WGS VIBQ|Cfa011050.t1
Betula platyphylla BPChr13G24645.v1.1.679
Casuarina glauca Casgl132S24015
Myrica rubra MrChr1G05000.mRNA1
Platycarya strobilacea Pschr6G222400.1
Carya illinoinensis rna-XM 043103603.1
Cyclocarya paliurus CpaF1st16344.t1
Pterocarya stenoptera Pst15G015390.1
Juglans regia rna-XM 018963809.2
Castanea crenata Cas 011374.T1
Quercus variabilis Quvar04aG0111700.1
Lithocarpus polystachyus LPO04G129200.1
Arabidopsis thaliana AT2G44740.1
Medicago truncatula MtrunA17 Chr7g0250121
Lotus japonicus Lj0g3v0340999.1
Phaseolus vulgaris Phvul.008G049600.1
Glycine max glyma.Wm82.gnm4.ann1.Glyma.18G247800.1
Glycine max glyma.Wm82.gnm4.ann1.Glyma.09G245400.1
Medicago truncatula MtrunA17 Chr8g0335971
Lotus japonicus Lj3g3v0597310.1
Phaseolus vulgaris Phvul.010G044800.1
Glycine max glyma.Wm82.gnm4.ann1.Glyma.03G107800.1
Glycine max glyma.Wm82.gnm4.ann1.Glyma.07G119100.1
Myrica rubra MrChr8G28670.mRNA1
Platycarya strobilacea Pschr9G133300.1
Carya illinoinensis rna-XM 043135241.1
Juglans regia rna-XM 018954897.2
Pterocarya stenoptera Pst13G018820.1
Cyclocarya paliurus CpaF1st43367.t1
Castanea crenata Cas 025623.T1
Quercus variabilis Quvar09aG0150700.1
Lithocarpus polystachyus LPO09G154000.1
Casuarina glauca Casgl4S17015
Betula platyphylla BPChr11G26395.v1.1.679
Corylus avellana Corav.Jeff.Hap1 g27670.t1
Carpinus fangiana rna-gnl|WGS VIBQ|Cfa013598.t1
Alnus rubra ALNRU00046255.RA
Alnus glutinosa Alglu07G0236060.T1

OG0003203:KLAVIER

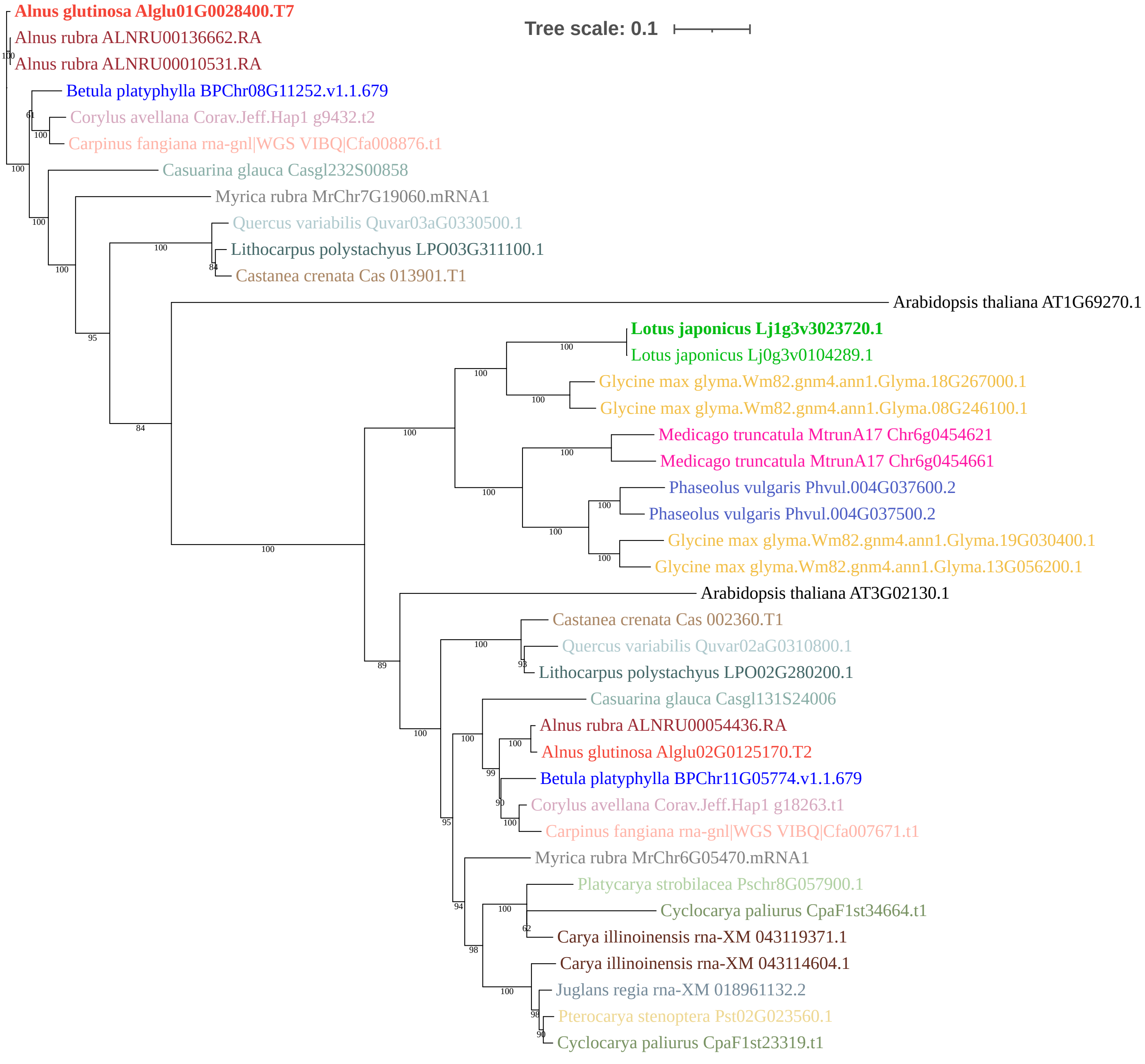

OG0003219:NODULIN HOMEOBOX

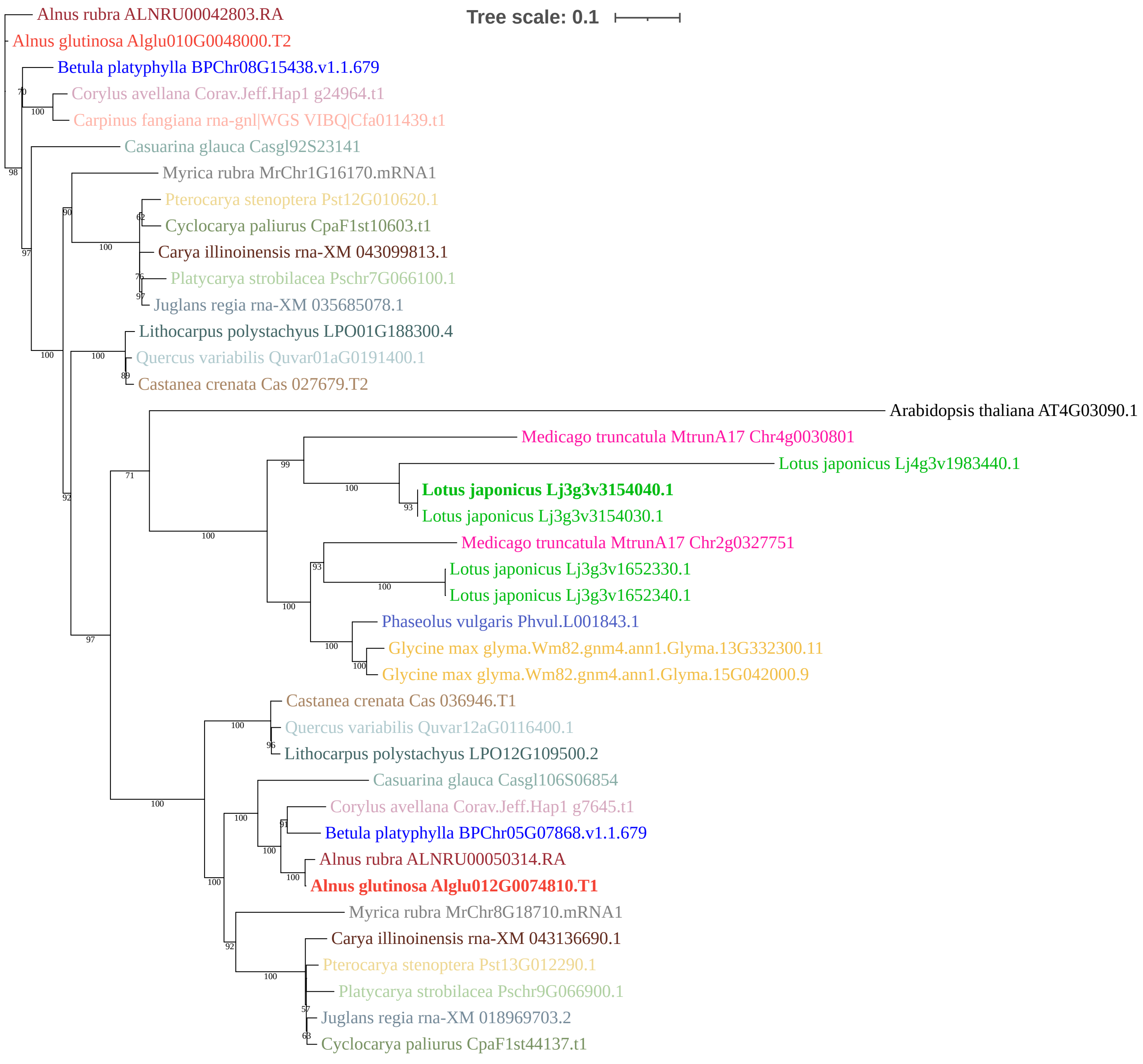

OG0003226:Protein phosphatase 2C

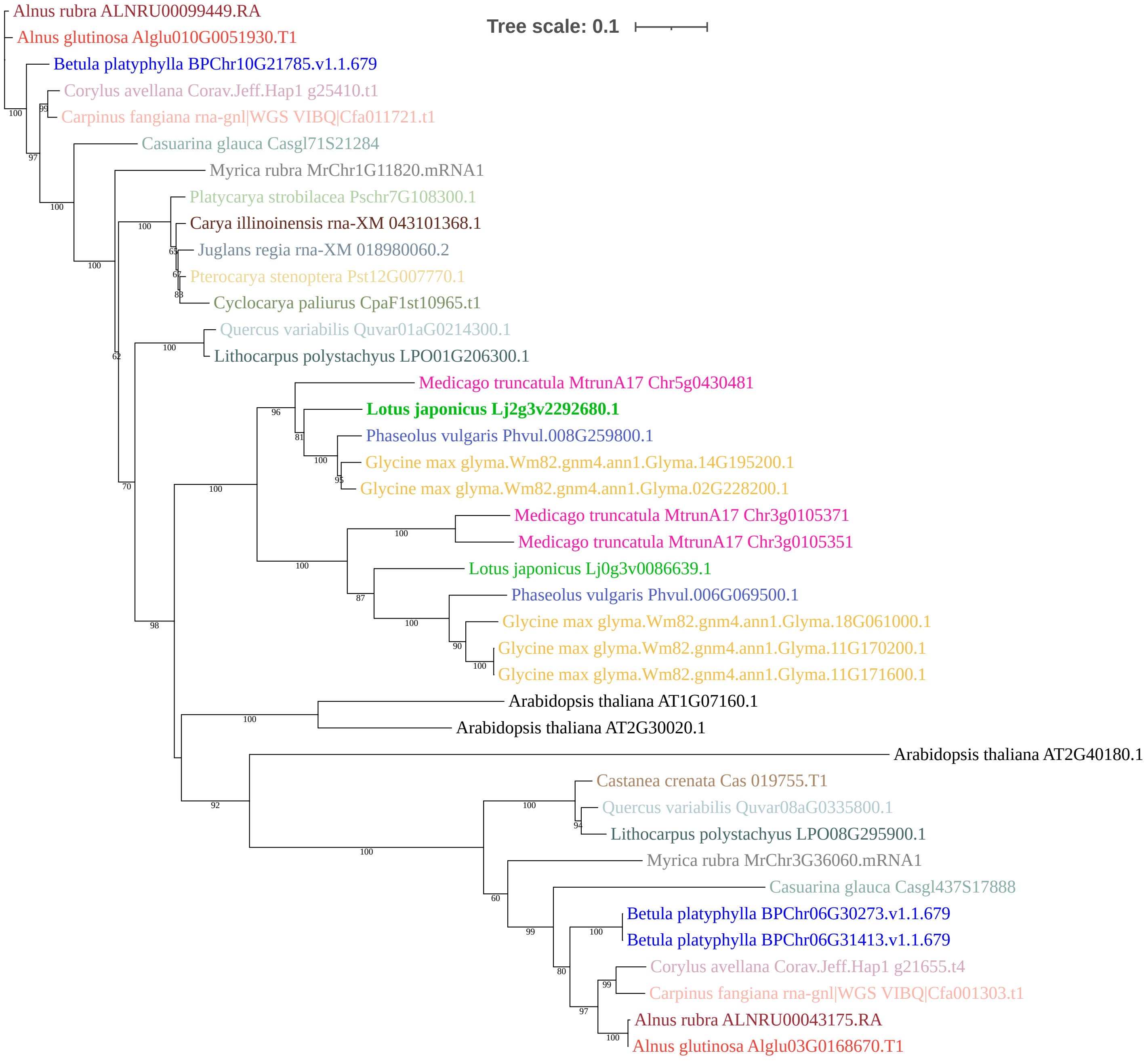

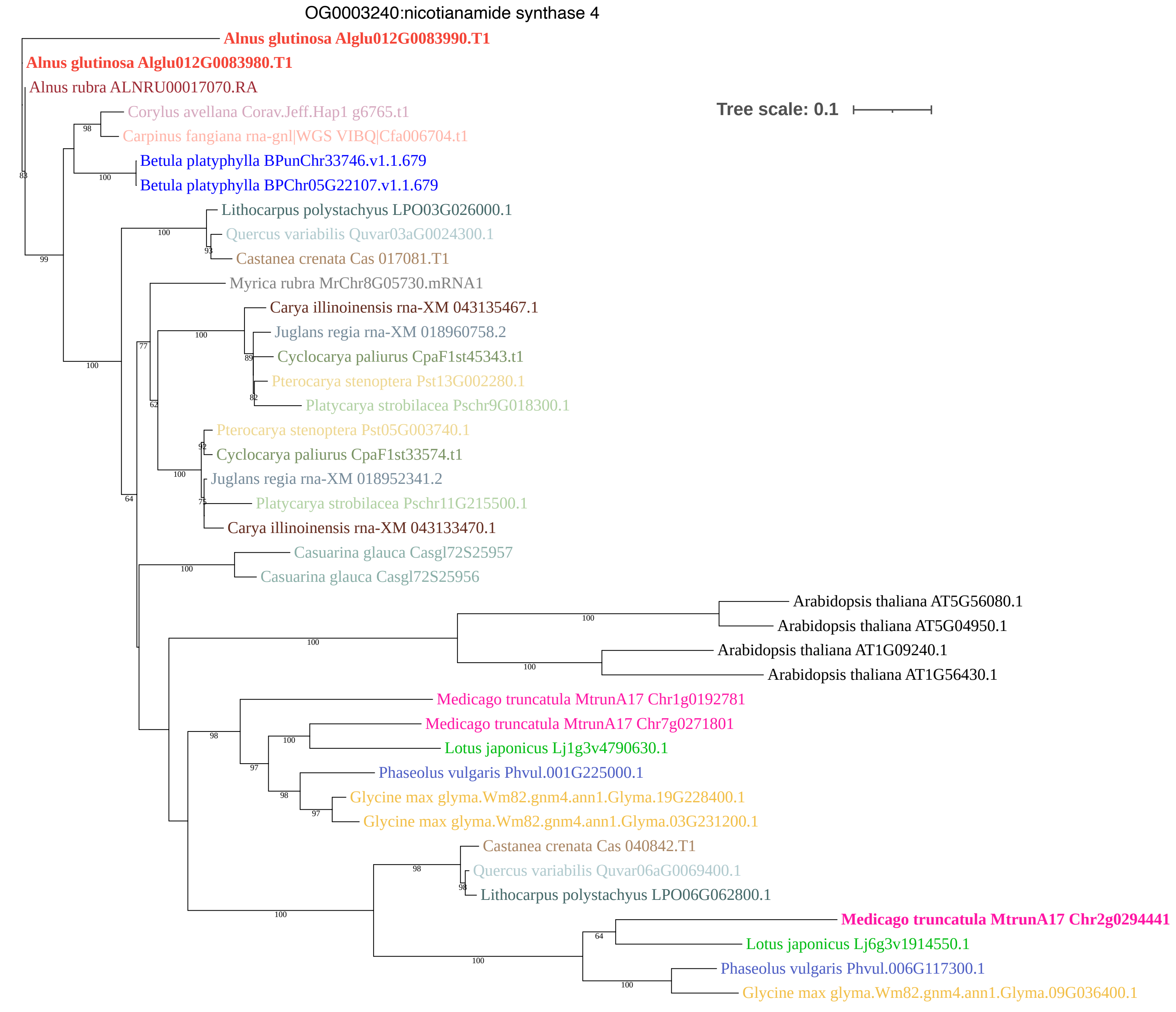
OG0003240:nicotianamide synthase 4
Tree scale: 0.1
Alnus glutinosa Alglu012G0083990.T1
Alnus glutinosa Alglu012G0083980.T1
Alnus rubra ALNRU00017070.RA
Corylus avellana Corav.Jeff.Hap1 g6765.t1
Carpinus fangiana rna-gnl|WGS VIBQ|Cfa006704.t1
Betula platyphylla BPunChr33746.v1.1.679
Betula platyphylla BPChr05G22107.v1.1.679
Lithocarpus polystachyus LPO03G026000.1
Quercus variabilis Quvar03aG0024300.1
Castanea crenata Cas 017081.T1
Myrica rubra MrChr8G05730.mRNA1
Carya illinoinensis rna-XM 043135467.1
Juglans regia rna-XM 018960758.2
Cyclocarya paliurus CpaF1st45343.t1
Pterocarya stenoptera Pst13G002280.1
Platycarya strobilacea Pschr9G018300.1
Pterocarya stenoptera Pst05G003740.1
Cyclocarya paliurus CpaF1st33574.t1
Juglans regia rna-XM 018952341.2
Platycarya strobilacea Pschr11G215500.1
Carya illinoinensis rna-XM 043133470.1
Casuarina glauca Casgl72S25957
Casuarina glauca Casgl72S25956
Arabidopsis thaliana AT5G56080.1
Arabidopsis thaliana AT5G04950.1
Arabidopsis thaliana AT1G09240.1
Arabidopsis thaliana AT1G56430.1
Medicago truncatula MtrunA17 Chr1g0192781
Medicago truncatula MtrunA17 Chr7g0271801
Lotus japonicus Lj1g3v4790630.1
Phaseolus vulgaris Phvul.001G225000.1
Glycine max glyma.Wm82.gnm4.ann1.Glyma.19G228400.1
Glycine max glyma.Wm82.gnm4.ann1.Glyma.03G231200.1
Castanea crenata Cas 040842.T1
Quercus variabilis Quvar06aG0069400.1
Lithocarpus polystachyus LPO06G062800.1
Medicago truncatula MtrunA17 Chr2g0294441
Lotus japonicus Lj6g3v1914550.1
Phaseolus vulgaris Phvul.006G117300.1
Glycine max glyma.Wm82.gnm4.ann1.Glyma.09G036400.1

OG0003258:CELL CYCLE SWITCH GENE encoding a 52 kDa protein

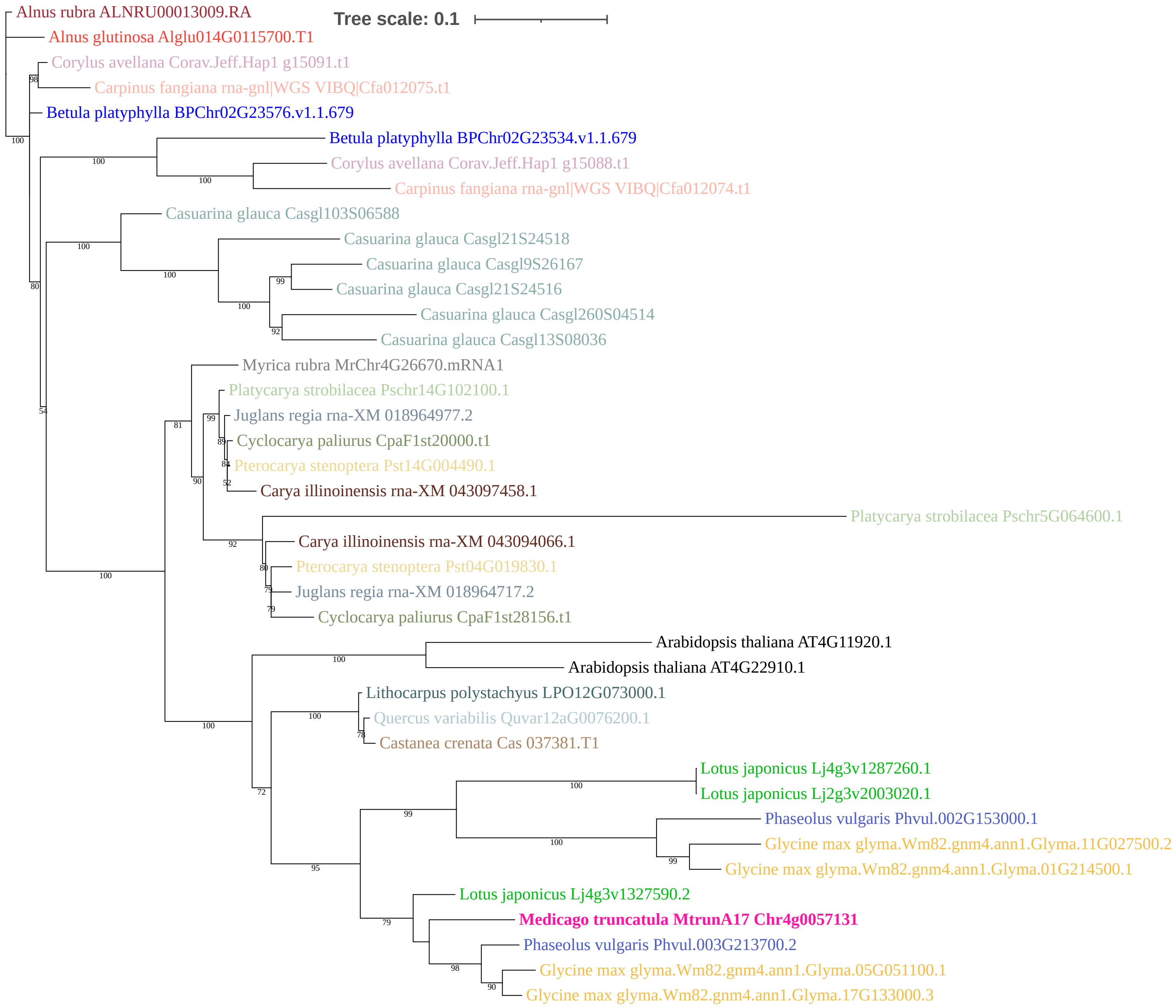

OG0003271:CLAVATA3/ENDOSPERM SURROUNDING REGION12

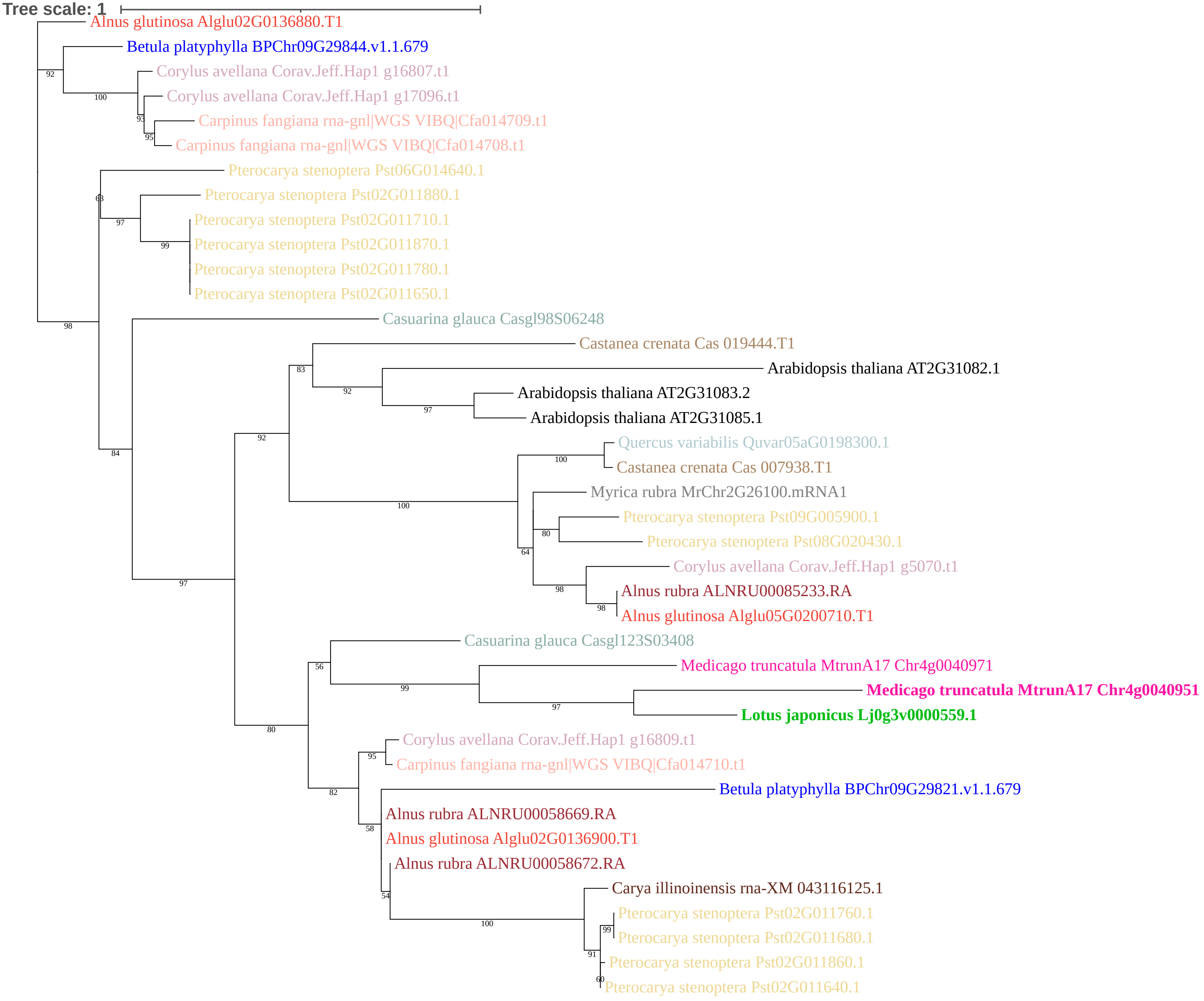

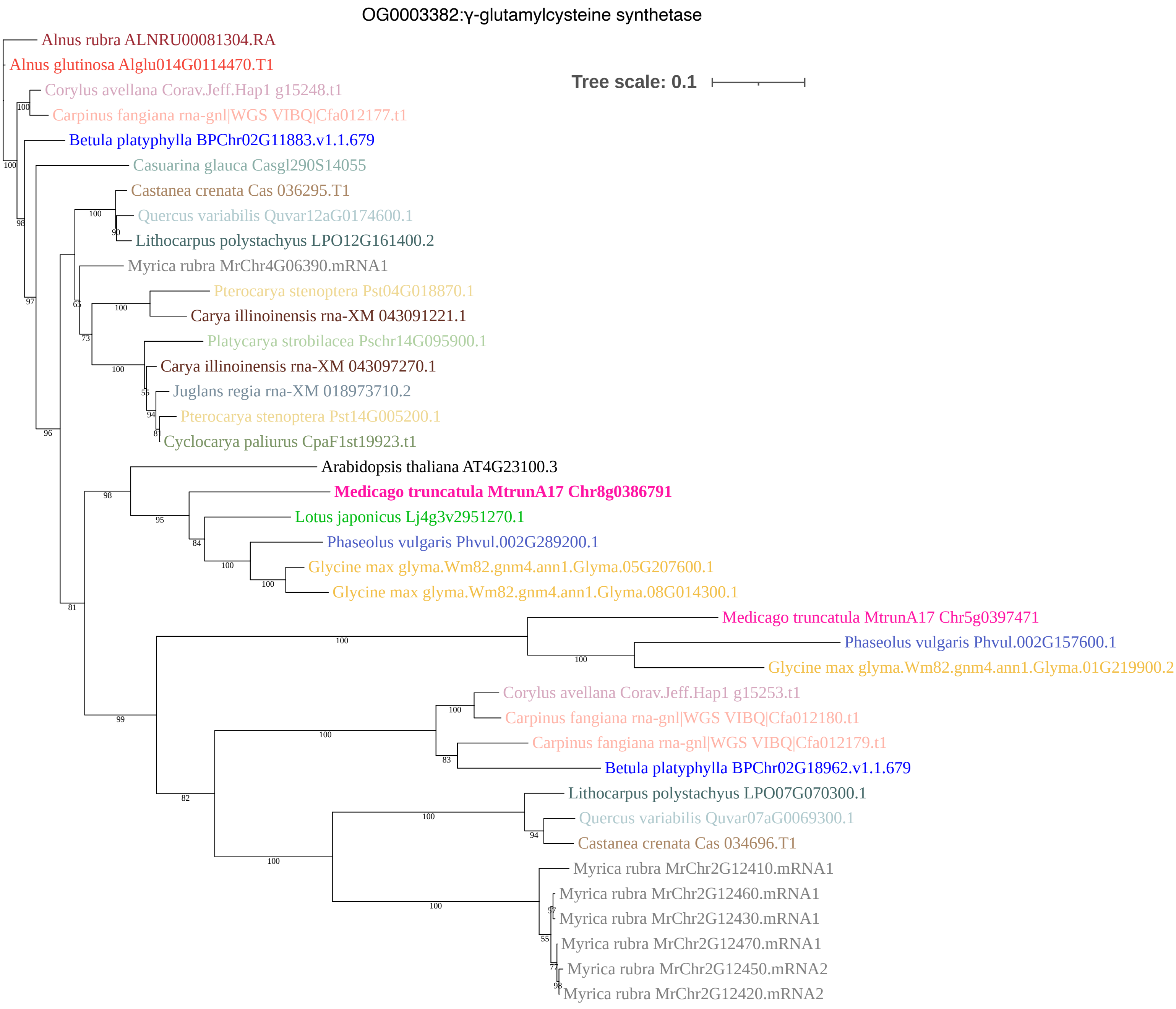
OG0003382:γ-glutamylcysteine synthetase
Tree scale: 0.1
Alnus rubra ALNRU00081304.RA
Alnus glutinosa Alglu014G0114470.T1
Corylus avellana Corav.Jeff.Hap1 g15248.t1
Carpinus fangiana rna-gnl|WGS VIBQ|Cfa012177.t1
Betula platyphylla BPChr02G11883.v1.1.679
Casuarina glauca Casgl290S14055
Castanea crenata Cas 036295.T1
Quercus variabilis Quvar12aG0174600.1
Lithocarpus polystachyus LPO12G161400.2
Myrica rubra MrChr4G06390.mRNA1
Pterocarya stenoptera Pst04G018870.1
Carya illinoinensis rna-XM 043091221.1
Platycarya strobilacea Pschr14G095900.1
Carya illinoinensis rna-XM 043097270.1
Juglans regia rna-XM 018973710.2
Pterocarya stenoptera Pst14G005200.1
Cyclocarya paliurus CpaF1st19923.t1
Arabidopsis thaliana AT4G23100.3
Medicago truncatula MtrunA17 Chr8g0386791
Lotus japonicus Lj4g3v2951270.1
Phaseolus vulgaris Phvul.002G289200.1
Glycine max glyma.Wm82.gnm4.ann1.Glyma.05G207600.1
Glycine max glyma.Wm82.gnm4.ann1.Glyma.08G014300.1
Medicago truncatula MtrunA17 Chr5g0397471
Phaseolus vulgaris Phvul.002G157600.1
Glycine max glyma.Wm82.gnm4.ann1.Glyma.01G219900.2
Corylus avellana Corav.Jeff.Hap1 g15253.t1
Carpinus fangiana rna-gnl|WGS VIBQ|Cfa012180.t1
Carpinus fangiana rna-gnl|WGS VIBQ|Cfa012179.t1
Betula platyphylla BPChr02G18962.v1.1.679
Lithocarpus polystachyus LPO07G070300.1
Quercus variabilis Quvar07aG0069300.1
Castanea crenata Cas 034696.T1
Myrica rubra MrChr2G12410.mRNA1
Myrica rubra MrChr2G12460.mRNA1
Myrica rubra MrChr2G12430.mRNA1
Myrica rubra MrChr2G12470.mRNA1
Myrica rubra MrChr2G12450.mRNA2
Myrica rubra MrChr2G12420.mRNA2

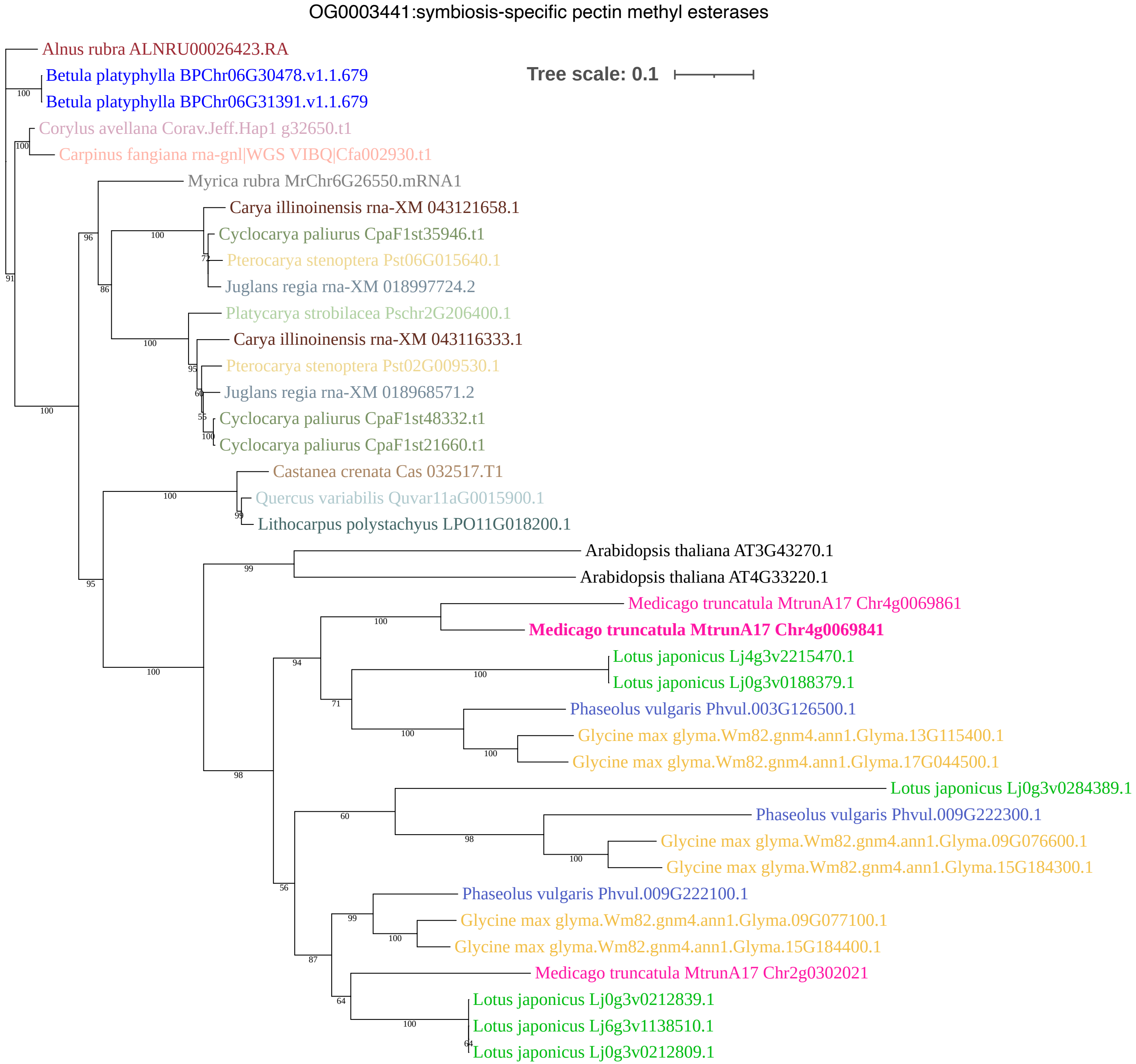
OG0003441:symbiosis-specific pectin methyl esterases
Tree scale: 0.1
Alnus rubra ALNRU00026423.RA
Betula platyphylla BPChr06G30478.v1.1.679
Betula platyphylla BPChr06G31391.v1.1.679
Corylus avellana Corav.Jeff.Hap1 g32650.t1
Carpinus fangiana rna-gnl|WGS VIBQ|Cfa002930.t1
Myrica rubra MrChr6G26550.mRNA1
Carya illinoinensis rna-XM 043121658.1
Cyclocarya paliurus CpaF1st35946.t1
Pterocarya stenoptera Pst06G015640.1
Juglans regia rna-XM 018997724.2
Platycarya strobilacea Pschr2G206400.1
Carya illinoinensis rna-XM 043116333.1
Pterocarya stenoptera Pst02G009530.1
Juglans regia rna-XM 018968571.2
Cyclocarya paliurus CpaF1st48332.t1
Cyclocarya paliurus CpaF1st21660.t1
Castanea crenata Cas 032517.T1
Quercus variabilis Quvar11aG0015900.1
Lithocarpus polystachyus LPO11G018200.1
Arabidopsis thaliana AT3G43270.1
Arabidopsis thaliana AT4G33220.1
Medicago truncatula MtrunA17 Chr4g0069861
Medicago truncatula MtrunA17 Chr4g0069841
Lotus japonicus Lj4g3v2215470.1
Lotus japonicus Lj0g3v0188379.1
Phaseolus vulgaris Phvul.003G126500.1
Glycine max glyma.Wm82.gnm4.ann1.Glyma.13G115400.1
Glycine max glyma.Wm82.gnm4.ann1.Glyma.17G044500.1
Lotus japonicus Lj0g3v0284389.1
Phaseolus vulgaris Phvul.009G222300.1
Glycine max glyma.Wm82.gnm4.ann1.Glyma.09G076600.1
Glycine max glyma.Wm82.gnm4.ann1.Glyma.15G184300.1
Phaseolus vulgaris Phvul.009G222100.1
Glycine max glyma.Wm82.gnm4.ann1.Glyma.09G077100.1
Glycine max glyma.Wm82.gnm4.ann1.Glyma.15G184400.1
Medicago truncatula MtrunA17 Chr2g0302021
Lotus japonicus Lj0g3v0212839.1
Lotus japonicus Lj6g3v1138510.1
Lotus japonicus Lj0g3v0212809.1

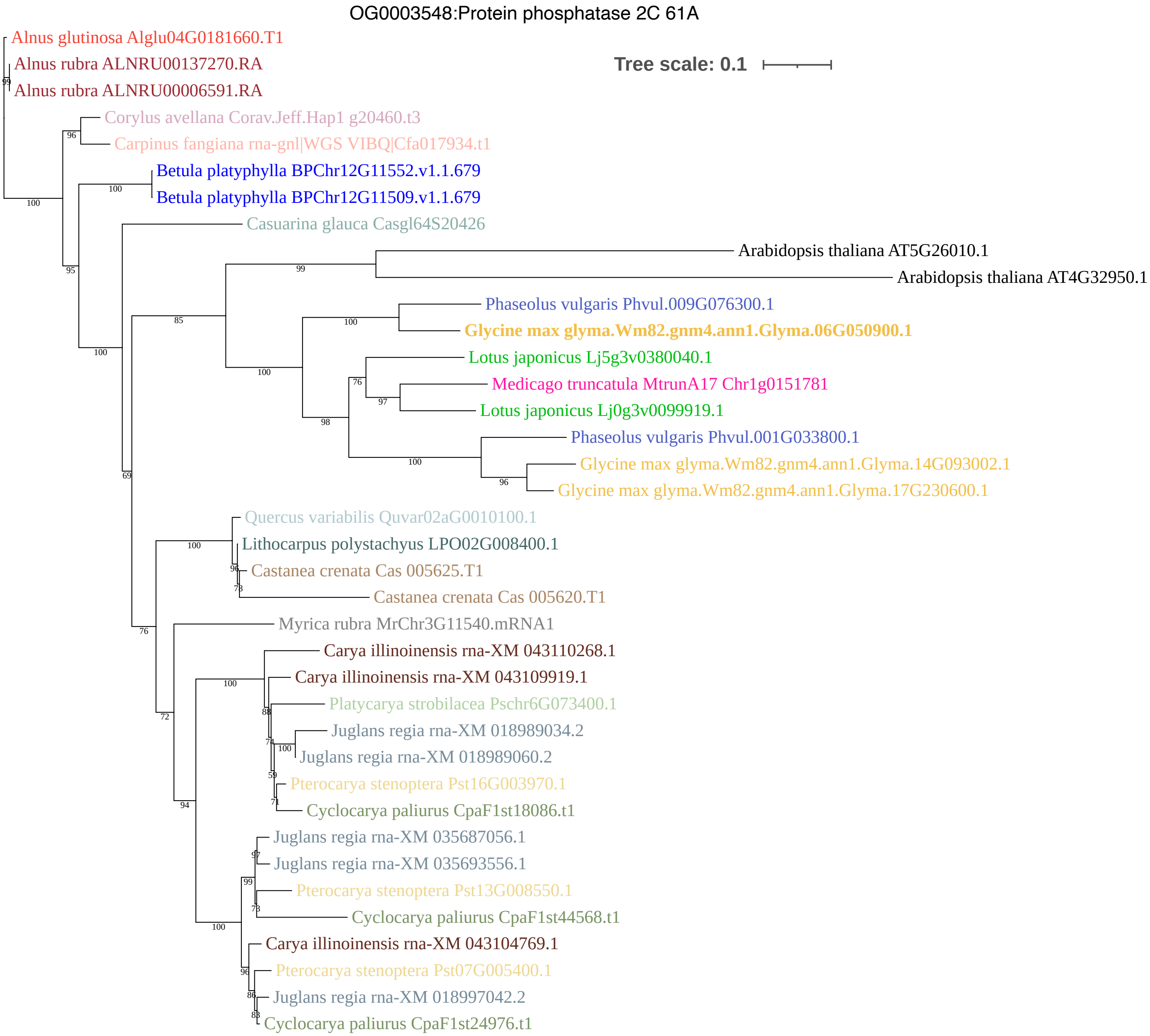
OG0003548:Protein phosphatase 2C 61A
Tree scale: 0.1
Alnus glutinosa Alglu04G0181660.T1
Alnus rubra ALNRU00137270.RA
Alnus rubra ALNRU00006591.RA
Corylus avellana Corav.Jeff.Hap1 g20460.t3
Carpinus fangiana rna-gnl|WGS VIBQ|Cfa017934.t1
Betula platyphylla BPChr12G11552.v1.1.679
Betula platyphylla BPChr12G11509.v1.1.679
Casuarina glauca Casgl64S20426
Arabidopsis thaliana AT5G26010.1
Arabidopsis thaliana AT4G32950.1
Phaseolus vulgaris Phvul.009G076300.1
Glycine max glyma.Wm82.gnm4.ann1.Glyma.06G050900.1
Lotus japonicus Lj5g3v0380040.1
Medicago truncatula MtrunA17 Chr1g0151781
Lotus japonicus Lj0g3v0099919.1
Phaseolus vulgaris Phvul.001G033800.1
Glycine max glyma.Wm82.gnm4.ann1.Glyma.14G093002.1
Glycine max glyma.Wm82.gnm4.ann1.Glyma.17G230600.1
Quercus variabilis Quvar02aG0010100.1
Lithocarpus polystachyus LPO02G008400.1
Castanea crenata Cas 005625.T1
Castanea crenata Cas 005620.T1
Myrica rubra MrChr3G11540.mRNA1
Carya illinoinensis rna-XM 043110268.1
Carya illinoinensis rna-XM 043109919.1
Platycarya strobilacea Pschr6G073400.1
Juglans regia rna-XM 018989034.2
Juglans regia rna-XM 018989060.2
Pterocarya stenoptera Pst16G003970.1
Cyclocarya paliurus CpaF1st18086.t1
Juglans regia rna-XM 035687056.1
Juglans regia rna-XM 035693556.1
Pterocarya stenoptera Pst13G008550.1
Cyclocarya paliurus CpaF1st44568.t1
Carya illinoinensis rna-XM 043104769.1
Pterocarya stenoptera Pst07G005400.1
Juglans regia rna-XM 018997042.2
Cyclocarya paliurus CpaF1st24976.t1

OG0003636:SUCROSE SYNTHASE

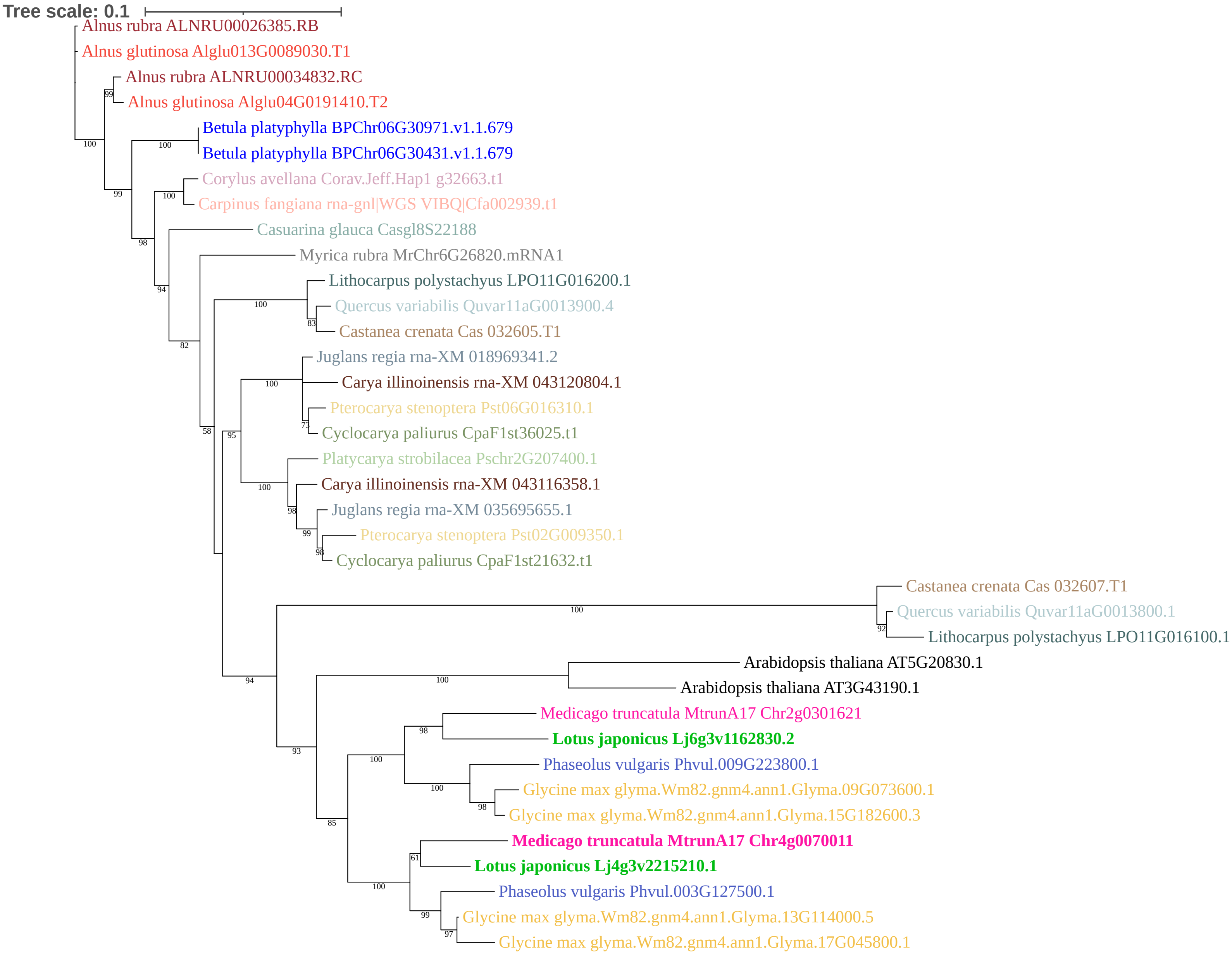

OG0003676:PENETRATION3 - LIKE

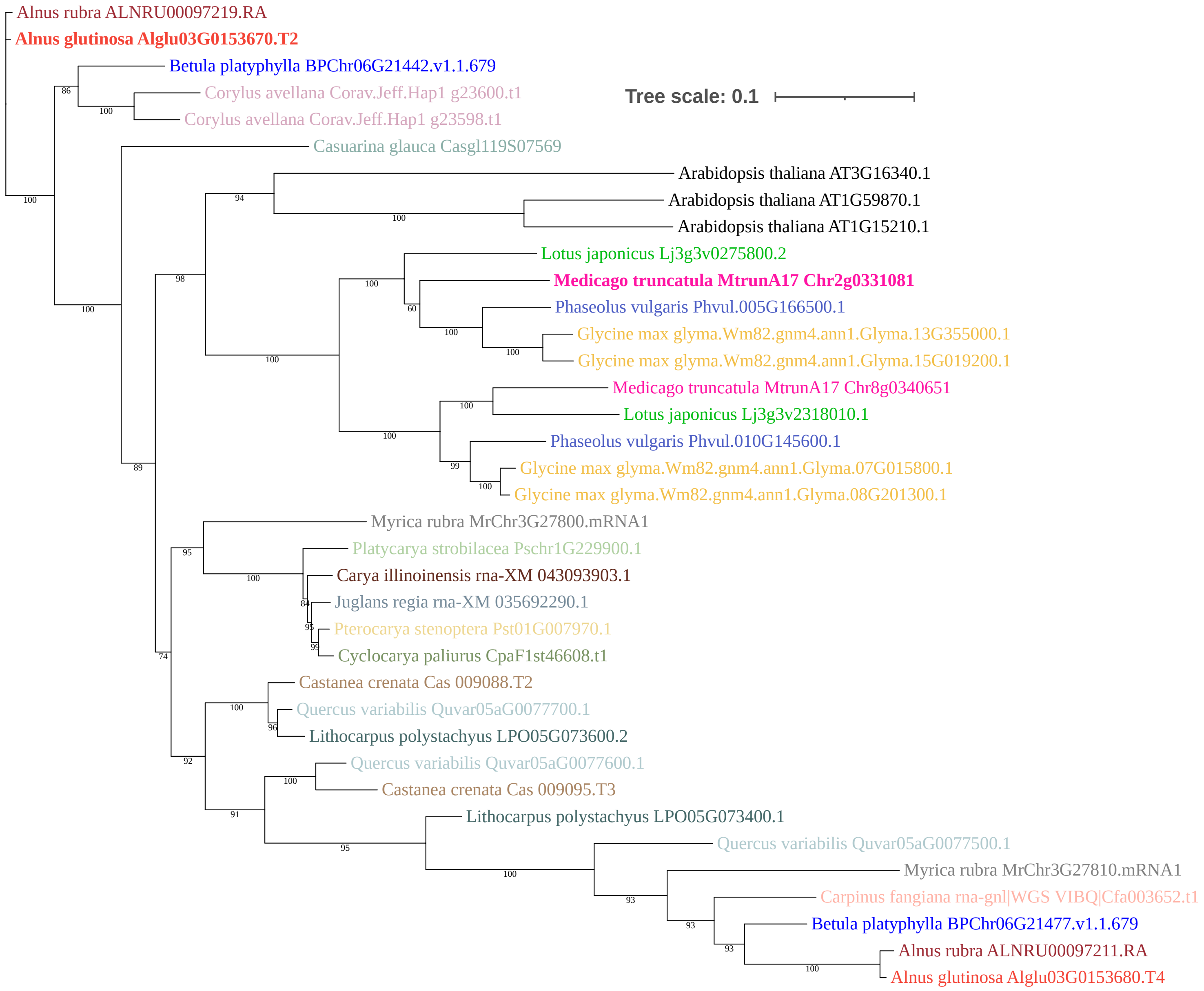

OG0003711:early phospohorylated protein 1

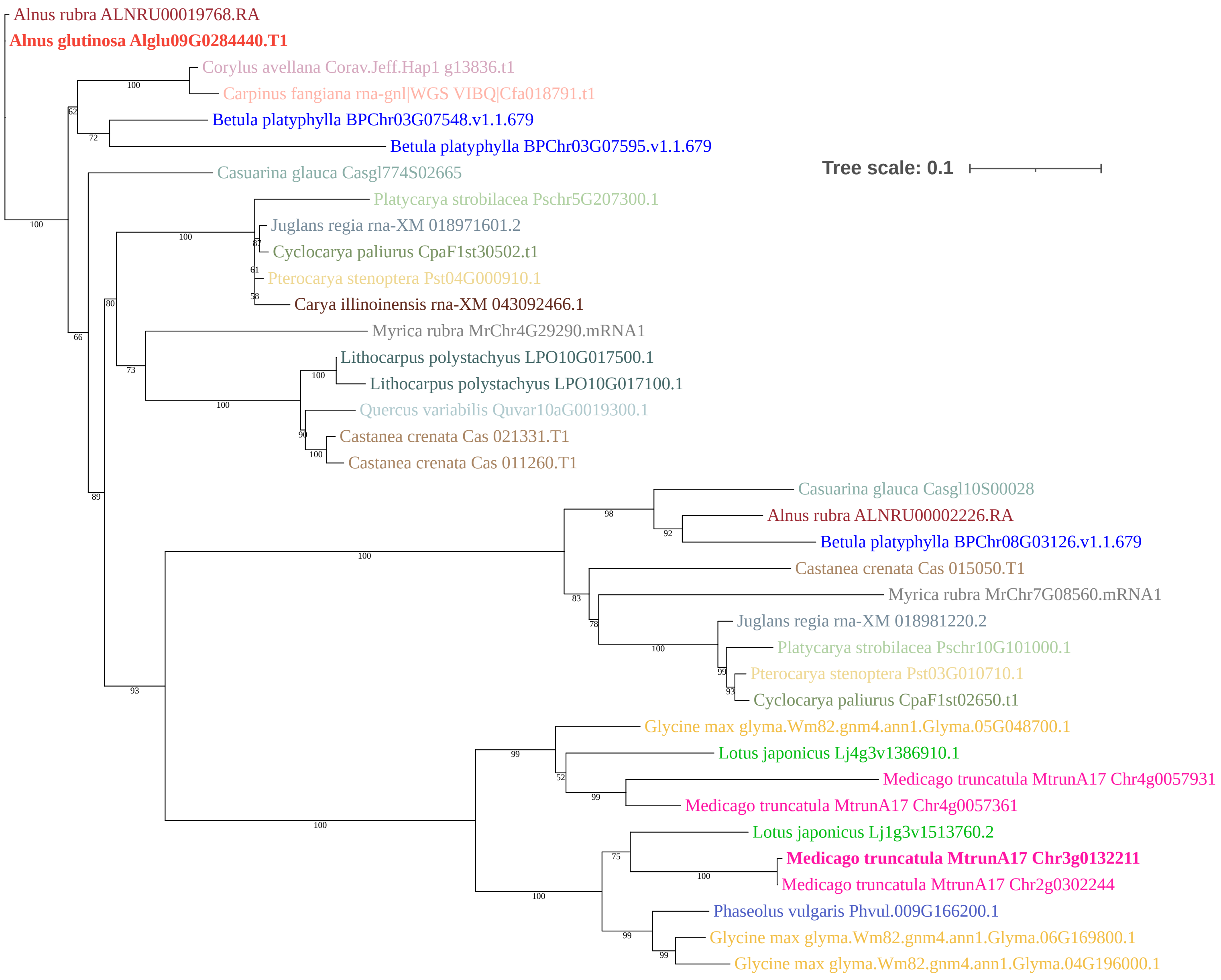

OG0003775:nitrate transporter 2.1

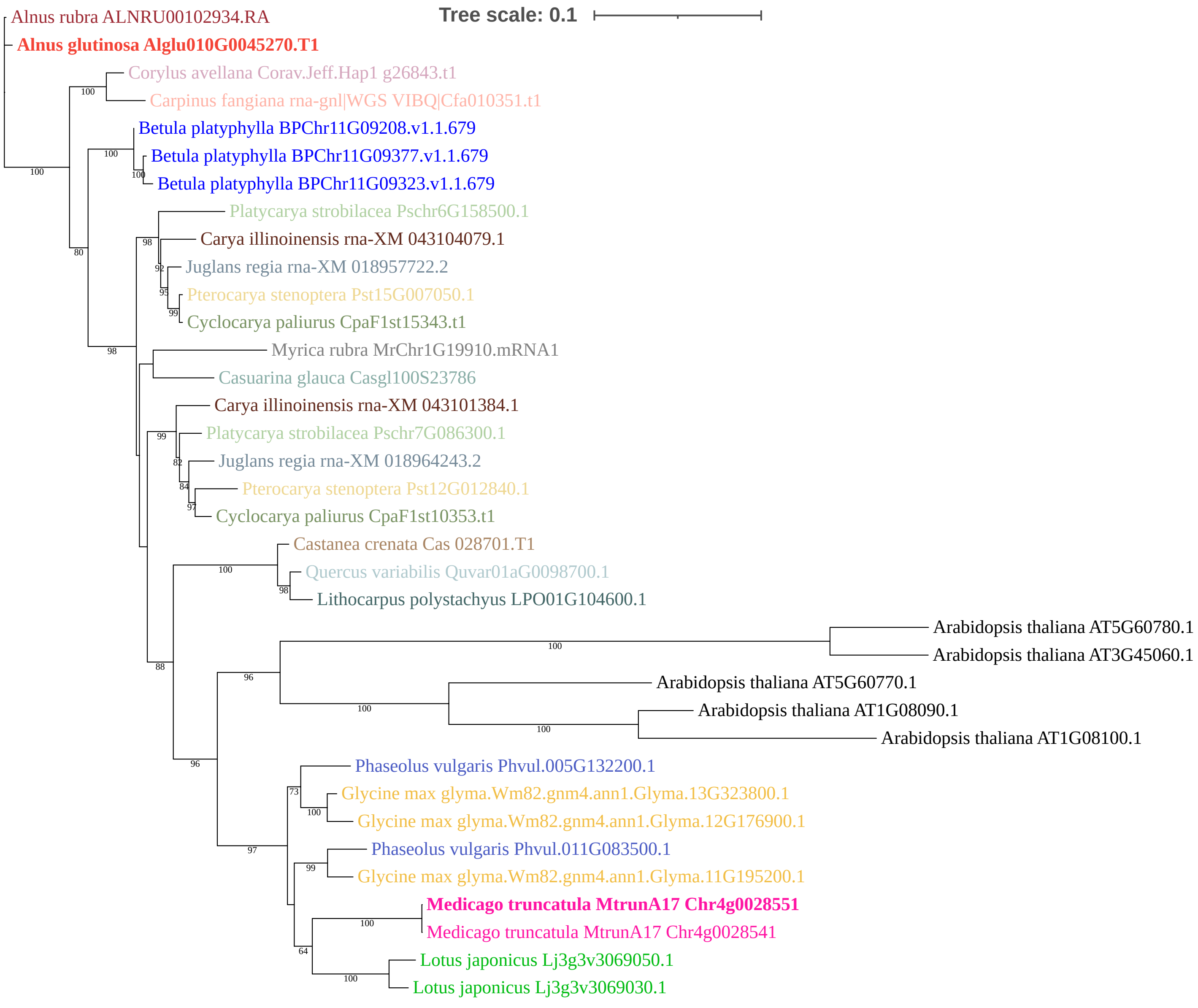

OG0003785:The target of rapamycin

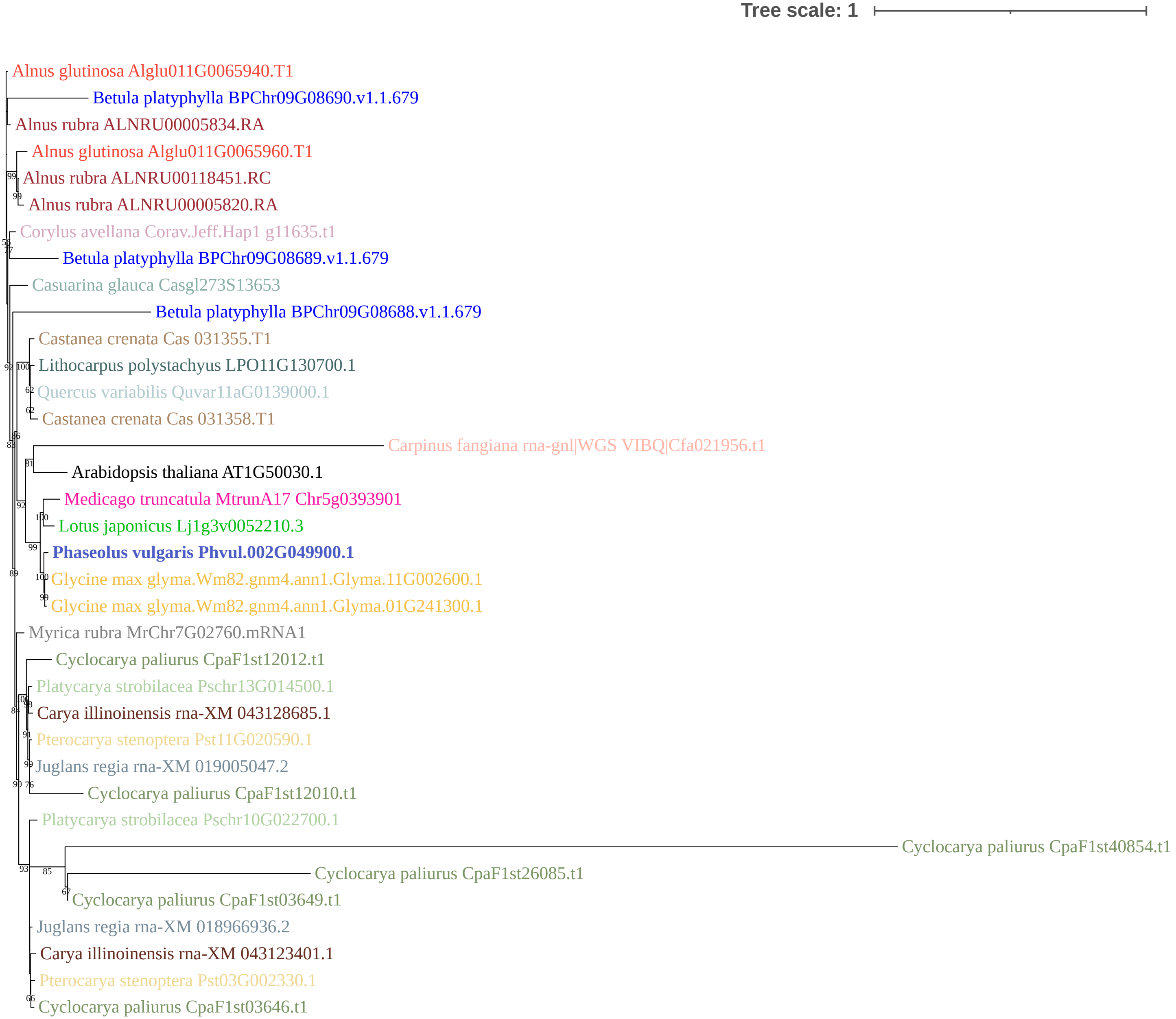

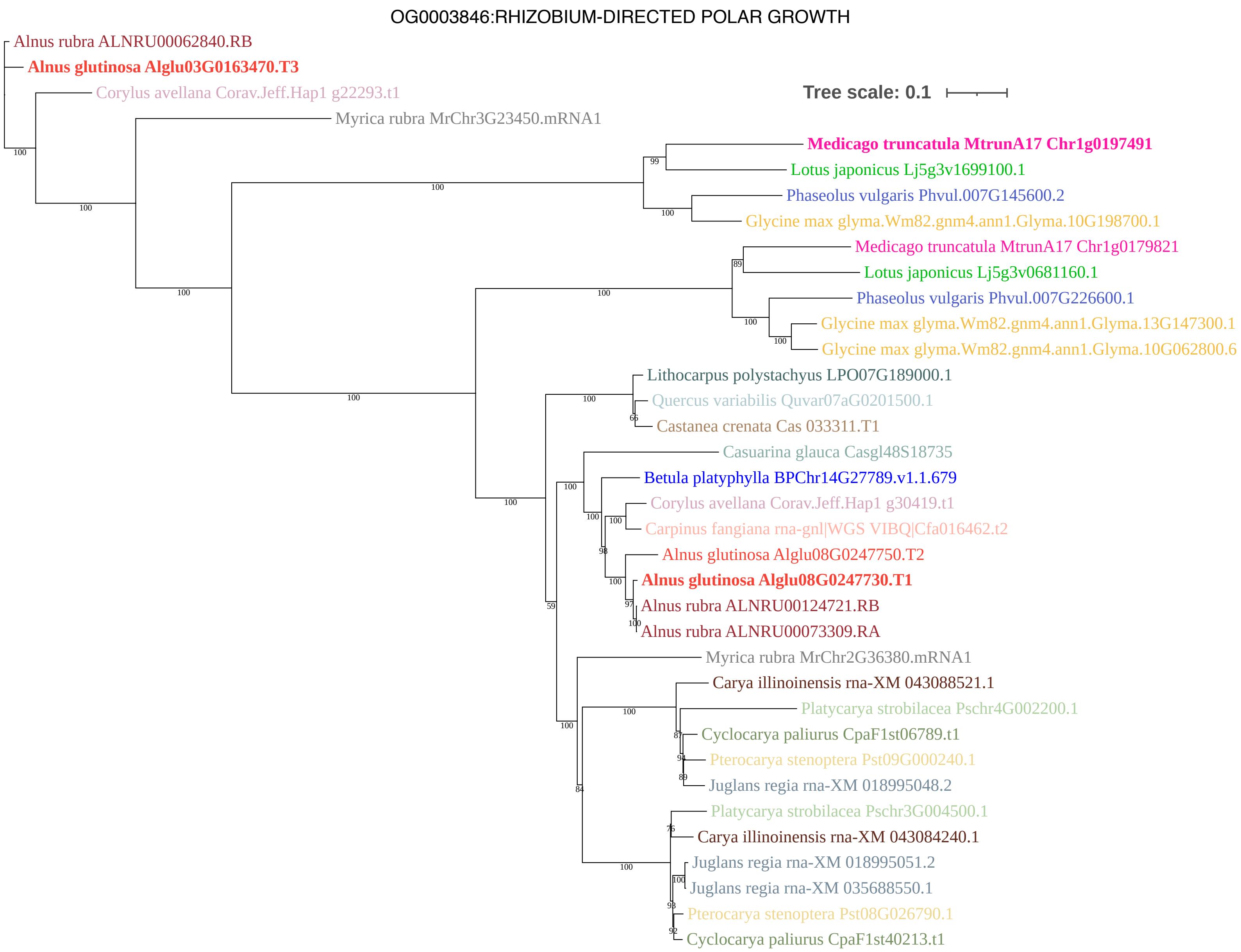
OG0003846:RHIZOBIUM-DIRECTED POLAR GROWTH
Tree scale: 0.1
Alnus rubra ALNRU00062840.RB
Alnus glutinosa Alglu03G0163470.T3
Corylus avellana Corav.Jeff.Hap1 g22293.t1
Myrica rubra MrChr3G23450.mRNA1
Medicago truncatula MtrunA17 Chr1g0197491
Lotus japonicus Lj5g3v1699100.1
Phaseolus vulgaris Phvul.007G145600.2
Glycine max glyma.Wm82.gnm4.ann1.Glyma.10G198700.1
Medicago truncatula MtrunA17 Chr1g0179821
Lotus japonicus Lj5g3v0681160.1
Phaseolus vulgaris Phvul.007G226600.1
Glycine max glyma.Wm82.gnm4.ann1.Glyma.13G147300.1
Glycine max glyma.Wm82.gnm4.ann1.Glyma.10G062800.6
Lithocarpus polystachyus LPO07G189000.1
Quercus variabilis Quvar07aG0201500.1
Castanea crenata Cas 033311.T1
Casuarina glauca Casgl48S18735
Betula platyphylla BPChr14G27789.v1.1.679
Corylus avellana Corav.Jeff.Hap1 g30419.t1
Carpinus fangiana rna-gnl|WGS VIBQ|Cfa016462.t2
Alnus glutinosa Alglu08G0247750.T2
Alnus glutinosa Alglu08G0247730.T1
Alnus rubra ALNRU00124721.RB
Alnus rubra ALNRU00073309.RA
Myrica rubra MrChr2G36380.mRNA1
Carya illinoinensis rna-XM 043088521.1
Platycarya strobilacea Pschr4G002200.1
Cyclocarya paliurus CpaF1st06789.t1
Pterocarya stenoptera Pst09G000240.1
Juglans regia rna-XM 018995048.2
Platycarya strobilacea Pschr3G004500.1
Carya illinoinensis rna-XM 043084240.1
Juglans regia rna-XM 018995051.2
Juglans regia rna-XM 035688550.1
Pterocarya stenoptera Pst08G026790.1
Cyclocarya paliurus CpaF1st40213.t1

OG0003947:UREIDE PERMEASE

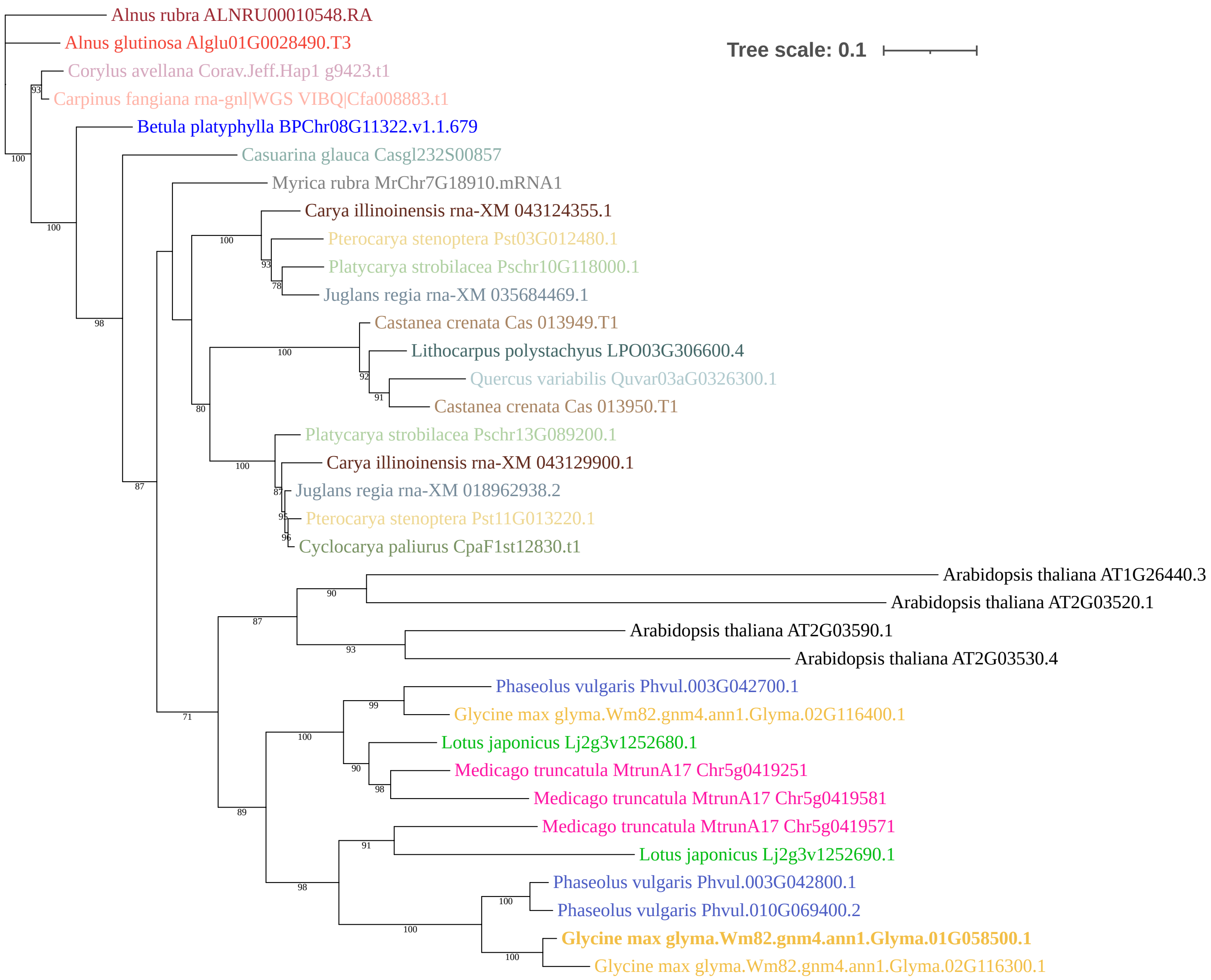

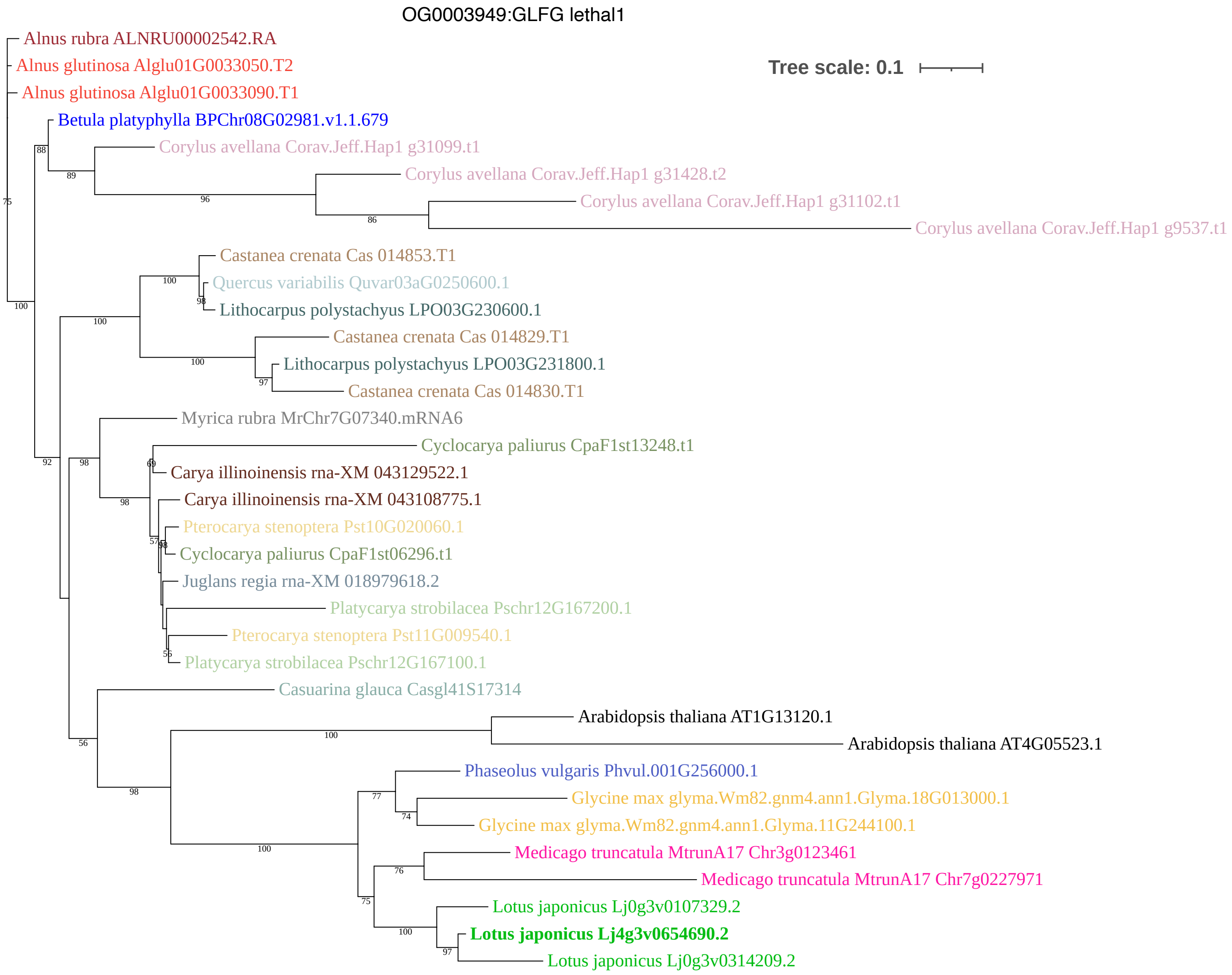

OG0003949:GLFG lethal1
Tree scale: 0.1
Alnus rubra ALNRU00002542.RA
Alnus glutinosa Alglu01G0033050.T2
Alnus glutinosa Alglu01G0033090.T1
Betula platyphylla BPChr08G02981.v1.1.679
Corylus avellana Corav.Jeff.Hap1 g31099.t1
Corylus avellana Corav.Jeff.Hap1 g31428.t2
Corylus avellana Corav.Jeff.Hap1 g31102.t1
Corylus avellana Corav.Jeff.Hap1 g9537.t1
Castanea crenata Cas 014853.T1
Quercus variabilis Quvar03aG0250600.1
Lithocarpus polystachyus LPO03G230600.1
Castanea crenata Cas 014829.T1
Lithocarpus polystachyus LPO03G231800.1
Castanea crenata Cas 014830.T1
Myrica rubra MrChr7G07340.mRNA6
Cyclocarya paliurus CpaF1st13248.t1
Carya illinoinensis rna-XM 043129522.1
Carya illinoinensis rna-XM 043108775.1
Pterocarya stenoptera Pst10G020060.1
Cyclocarya paliurus CpaF1st06296.t1
Juglans regia rna-XM 018979618.2
Platycarya strobilacea Pschr12G167200.1
Pterocarya stenoptera Pst11G009540.1
Platycarya strobilacea Pschr12G167100.1
Casuarina glauca Casgl41S17314
Arabidopsis thaliana AT1G13120.1
Arabidopsis thaliana AT4G05523.1
Phaseolus vulgaris Phvul.001G256000.1
Glycine max glyma.Wm82.gnm4.ann1.Glyma.18G013000.1
Glycine max glyma.Wm82.gnm4.ann1.Glyma.11G244100.1
Medicago truncatula MtrunA17 Chr3g0123461
Medicago truncatula MtrunA17 Chr7g0227971
Lotus japonicus Lj0g3v0107329.2
Lotus japonicus Lj4g3v0654690.2
Lotus japonicus Lj0g3v0314209.2

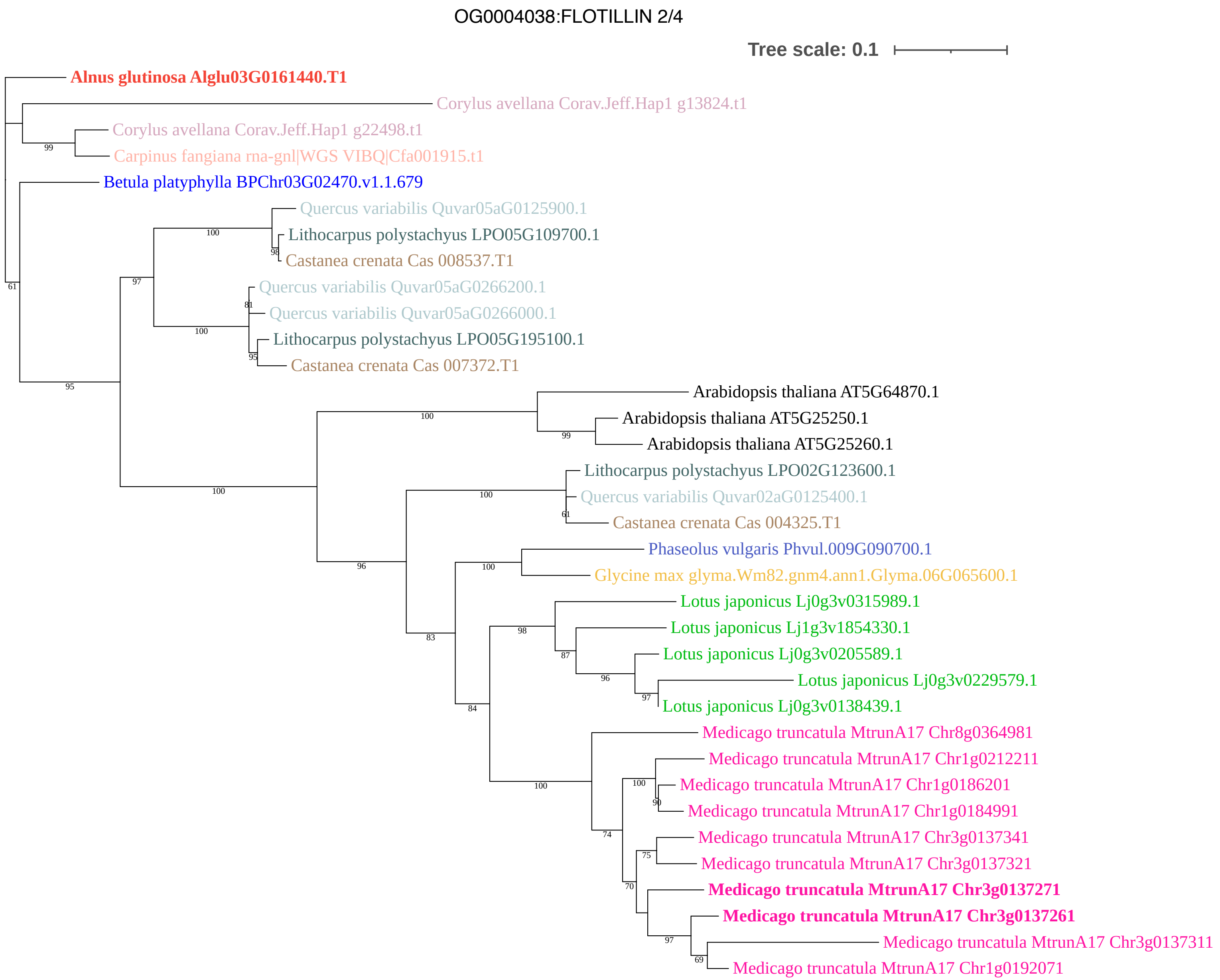
OG0004038:FLOTILLIN 2/4
Tree scale: 0.1
Alnus glutinosa Alglu03G0161440.T1
Corylus avellana Corav.Jeff.Hap1 g13824.t1
Corylus avellana Corav.Jeff.Hap1 g22498.t1
Carpinus fangiana rna-gnl|WGS VIBQ|Cfa001915.t1
Betula platyphylla BPChr03G02470.v1.1.679
Quercus variabilis Quvar05aG0125900.1
Lithocarpus polystachyus LPO05G109700.1
Castanea crenata Cas 008537.T1
Quercus variabilis Quvar05aG0266200.1
Quercus variabilis Quvar05aG0266000.1
Lithocarpus polystachyus LPO05G195100.1
Castanea crenata Cas 007372.T1
Arabidopsis thaliana AT5G64870.1
Arabidopsis thaliana AT5G25250.1
Arabidopsis thaliana AT5G25260.1
Lithocarpus polystachyus LPO02G123600.1
Quercus variabilis Quvar02aG0125400.1
Castanea crenata Cas 004325.T1
Phaseolus vulgaris Phvul.009G090700.1
Glycine max glyma.Wm82.gnm4.ann1.Glyma.06G065600.1
Lotus japonicus Lj0g3v0315989.1
Lotus japonicus Lj1g3v1854330.1
Lotus japonicus Lj0g3v0205589.1
Lotus japonicus Lj0g3v0229579.1
Lotus japonicus Lj0g3v0138439.1
Medicago truncatula MtrunA17 Chr8g0364981
Medicago truncatula MtrunA17 Chr1g0212211
Medicago truncatula MtrunA17 Chr1g0186201
Medicago truncatula MtrunA17 Chr1g0184991
Medicago truncatula MtrunA17 Chr3g0137341
Medicago truncatula MtrunA17 Chr3g0137321
Medicago truncatula MtrunA17 Chr3g0137271
Medicago truncatula MtrunA17 Chr3g0137261
Medicago truncatula MtrunA17 Chr3g0137311
Medicago truncatula MtrunA17 Chr1g0192071

OG0004042:synaptotagmin 3

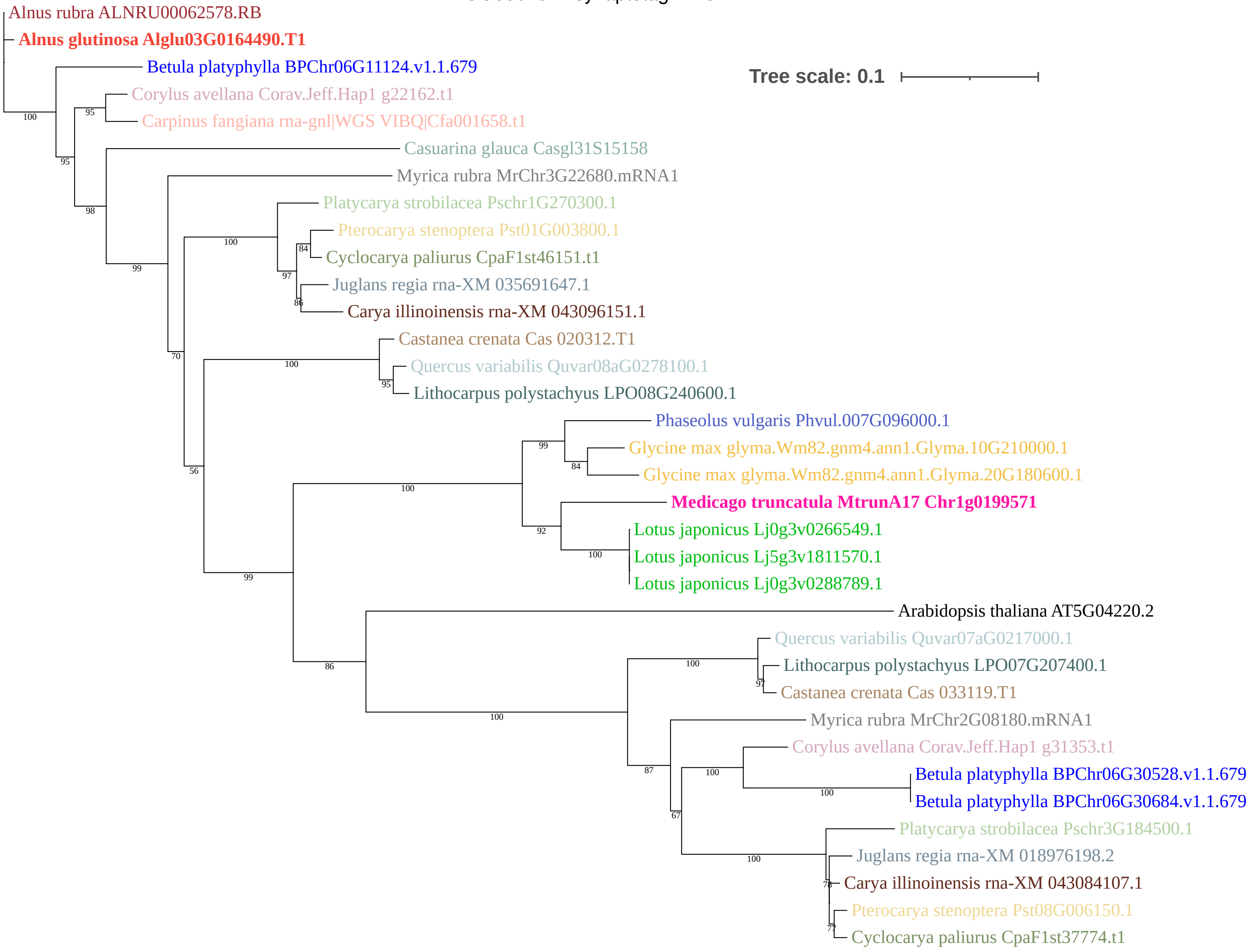

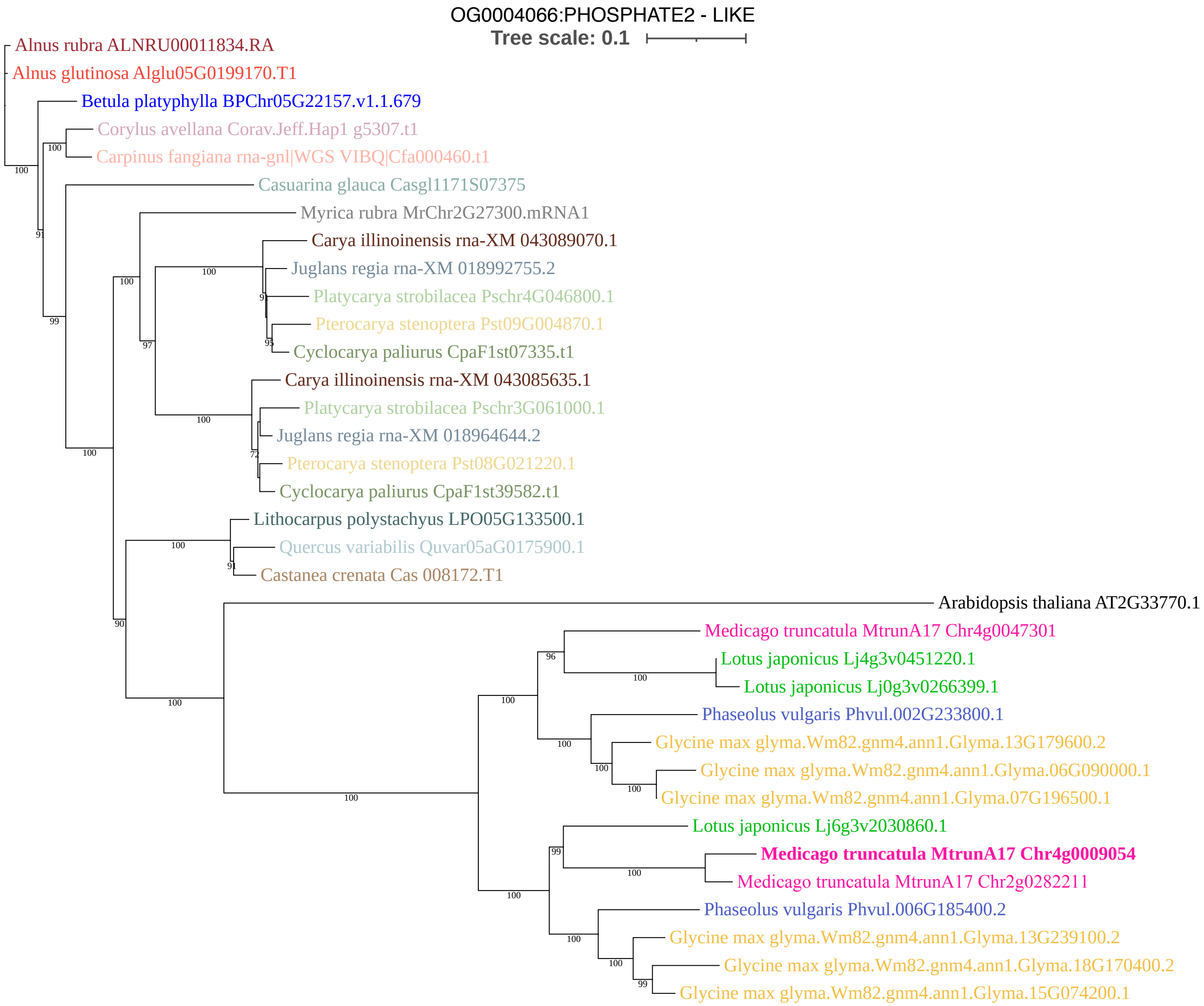
OG0004066:PHOSPHATE2 - LIKE
Tree scale: 0.1
Alnus rubra ALNRU00011834.RA
Alnus glutinosa Alglu05G0199170.T1
Betula platyphylla BPChr05G22157.v1.1.679
Corylus avellana Corav.Jeff.Hap1 g5307.t1
Carpinus fangiana rna-gnl|WGS VIBQ|Cfa000460.t1
Casuarina glauca Casgl1171S07375
Myrica rubra MrChr2G27300.mRNA1
Carya illinoinensis rna-XM 043089070.1
Juglans regia rna-XM 018992755.2
Platycarya strobilacea Pschr4G046800.1
Pterocarya stenoptera Pst09G004870.1
Cyclocarya paliurus CpaF1st07335.t1
Carya illinoinensis rna-XM 043085635.1
Platycarya strobilacea Pschr3G061000.1
Juglans regia rna-XM 018964644.2
Pterocarya stenoptera Pst08G021220.1
Cyclocarya paliurus CpaF1st39582.t1
Lithocarpus polystachyus LPO05G133500.1
Quercus variabilis Quvar05aG0175900.1
Castanea crenata Cas 008172.T1
Arabidopsis thaliana AT2G33770.1
Medicago truncatula MtrunA17 Chr4g0047301
Lotus japonicus Lj4g3v0451220.1
Lotus japonicus Lj0g3v0266399.1
Phaseolus vulgaris Phvul.002G233800.1
Glycine max glyma.Wm82.gnm4.ann1.Glyma.13G179600.2
Glycine max glyma.Wm82.gnm4.ann1.Glyma.06G090000.1
Glycine max glyma.Wm82.gnm4.ann1.Glyma.07G196500.1
Lotus japonicus Lj6g3v2030860.1
Medicago truncatula MtrunA17 Chr4g0009054
Medicago truncatula MtrunA17 Chr2g0282211
Phaseolus vulgaris Phvul.006G185400.2
Glycine max glyma.Wm82.gnm4.ann1.Glyma.13G239100.2
Glycine max glyma.Wm82.gnm4.ann1.Glyma.18G170400.2
Glycine max glyma.Wm82.gnm4.ann1.Glyma.15G074200.1

OG0004081:MULTIDRUG AND TOXIC COMPOUND EXTRUSION 1

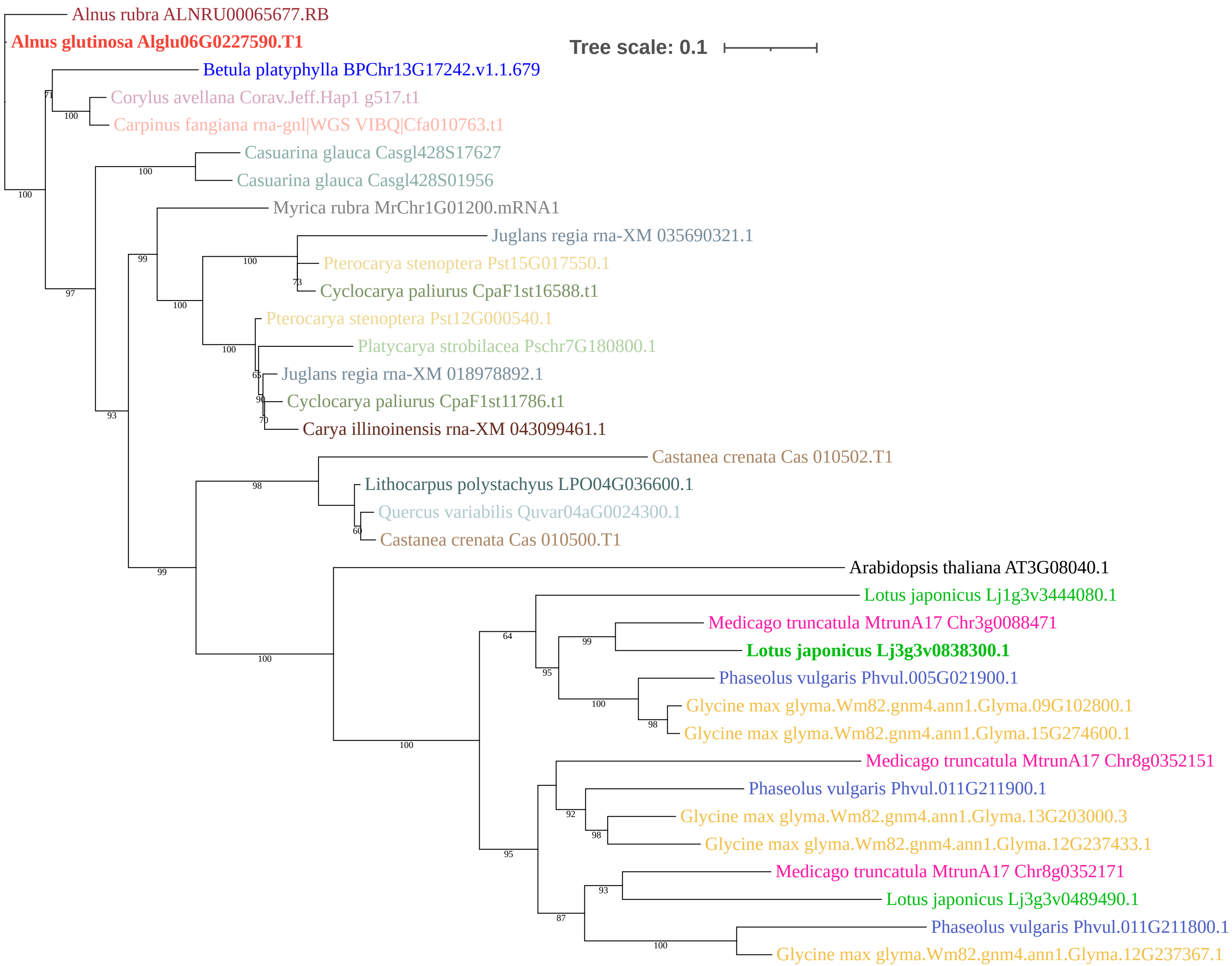

OG0004142:SHORT-ROOT 4/5

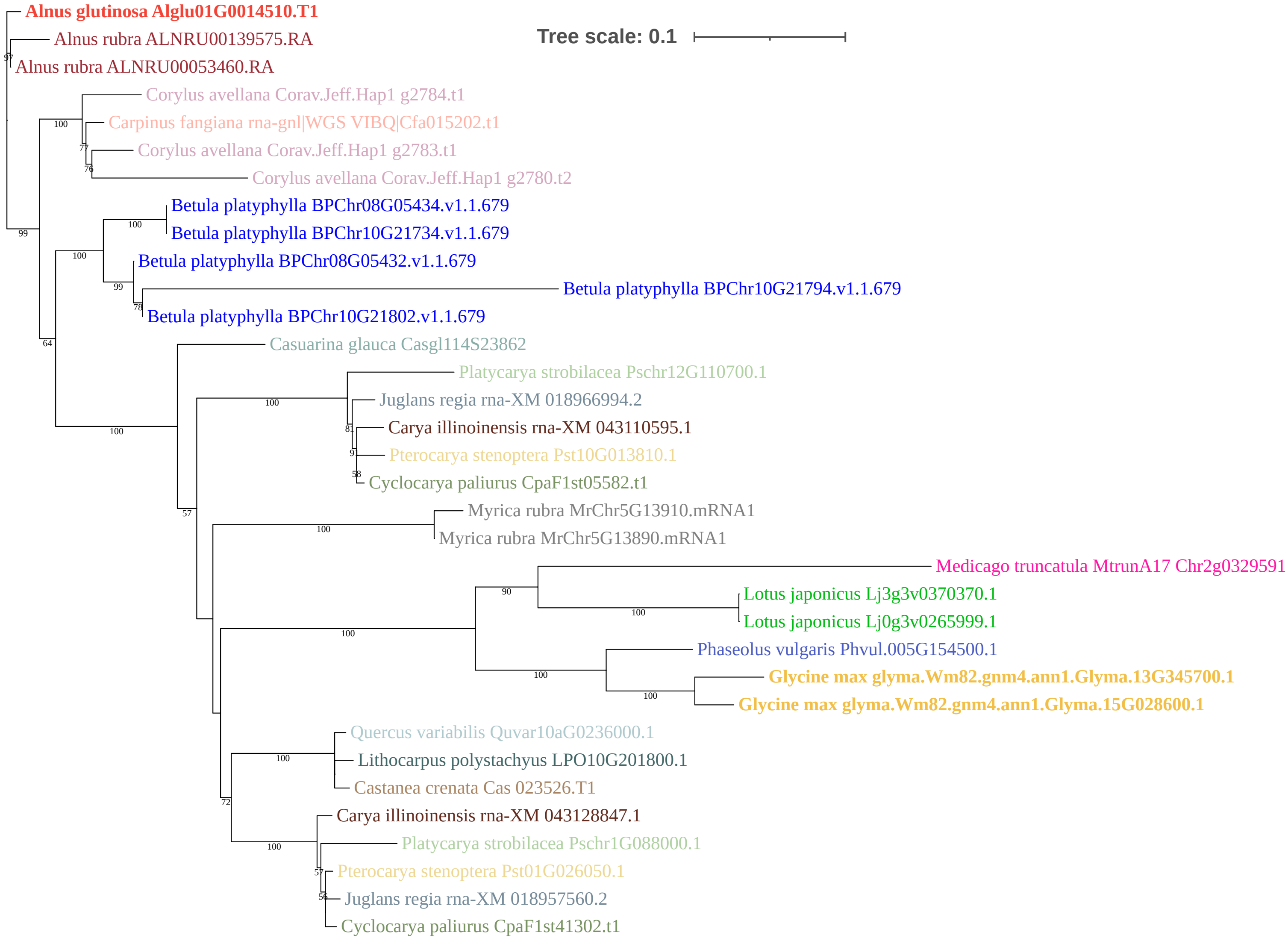

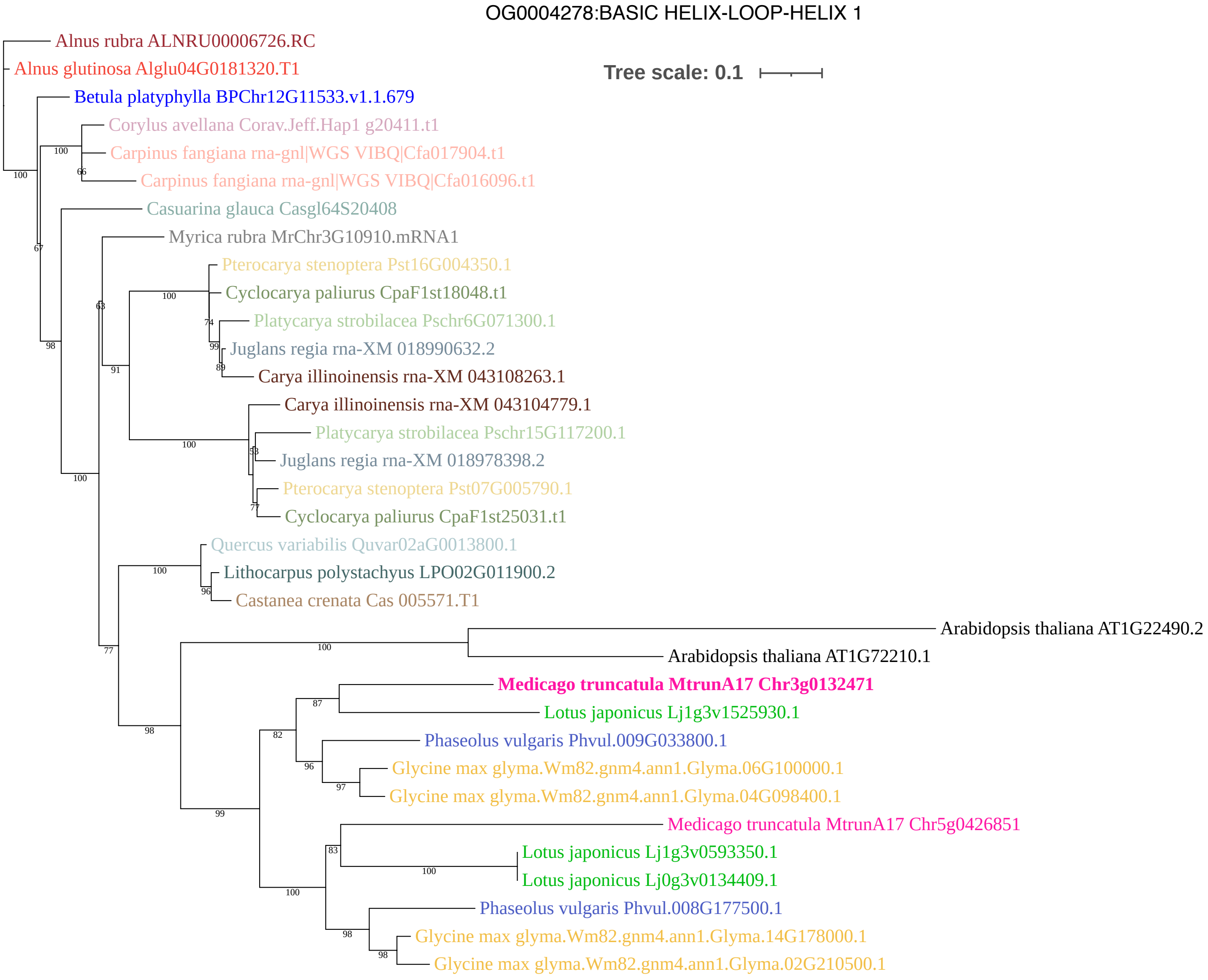
OG0004278:BASIC HELIX-LOOP-HELIX 1
Tree scale: 0.1
Alnus rubra ALNRU00006726.RC
Alnus glutinosa Alglu04G0181320.T1
Betula platyphylla BPChr12G11533.v1.1.679
Corylus avellana Corav.Jeff.Hap1 g20411.t1
Carpinus fangiana rna-gnl|WGS VIBQ|Cfa017904.t1
Carpinus fangiana rna-gnl|WGS VIBQ|Cfa016096.t1
Casuarina glauca Casgl64S20408
Myrica rubra MrChr3G10910.mRNA1
Pterocarya stenoptera Pst16G004350.1
Cyclocarya paliurus CpaF1st18048.t1
Platycarya strobilacea Pschr6G071300.1
Juglans regia rna-XM 018990632.2
Carya illinoinensis rna-XM 043108263.1
Carya illinoinensis rna-XM 043104779.1
Platycarya strobilacea Pschr15G117200.1
Juglans regia rna-XM 018978398.2
Pterocarya stenoptera Pst07G005790.1
Cyclocarya paliurus CpaF1st25031.t1
Quercus variabilis Quvar02aG0013800.1
Lithocarpus polystachyus LPO02G011900.2
Castanea crenata Cas 005571.T1
Arabidopsis thaliana AT1G22490.2
Arabidopsis thaliana AT1G72210.1
Medicago truncatula MtrunA17 Chr3g0132471
Lotus japonicus Lj1g3v1525930.1
Phaseolus vulgaris Phvul.009G033800.1
Glycine max glyma.Wm82.gnm4.ann1.Glyma.06G100000.1
Glycine max glyma.Wm82.gnm4.ann1.Glyma.04G098400.1
Medicago truncatula MtrunA17 Chr5g0426851
Lotus japonicus Lj1g3v0593350.1
Lotus japonicus Lj0g3v0134409.1
Phaseolus vulgaris Phvul.008G177500.1
Glycine max glyma.Wm82.gnm4.ann1.Glyma.14G178000.1
Glycine max glyma.Wm82.gnm4.ann1.Glyma.02G210500.1

OG0004316:novel pectatelyase enzyme(GmNPLa)transcriptional regulator 1a

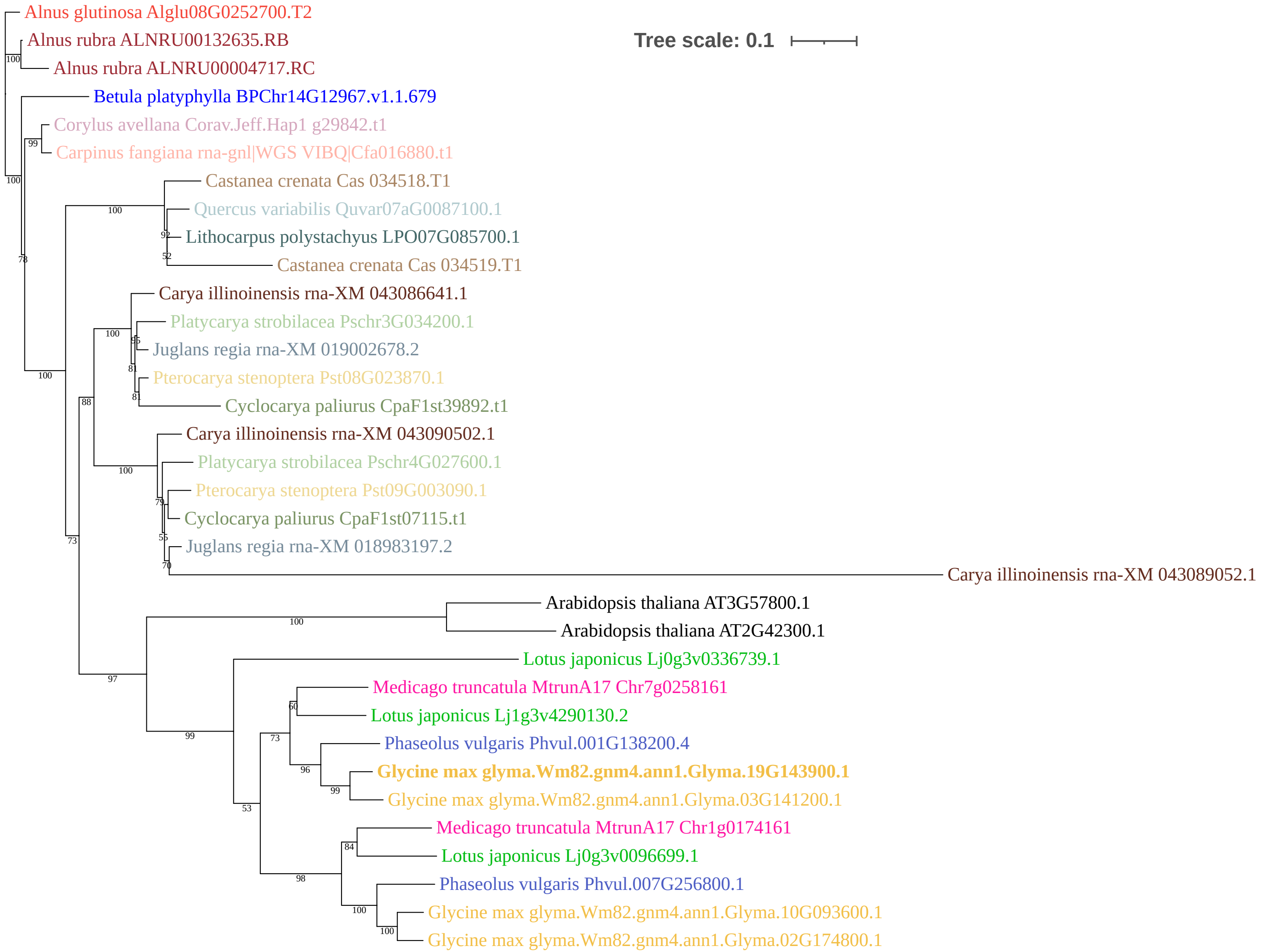

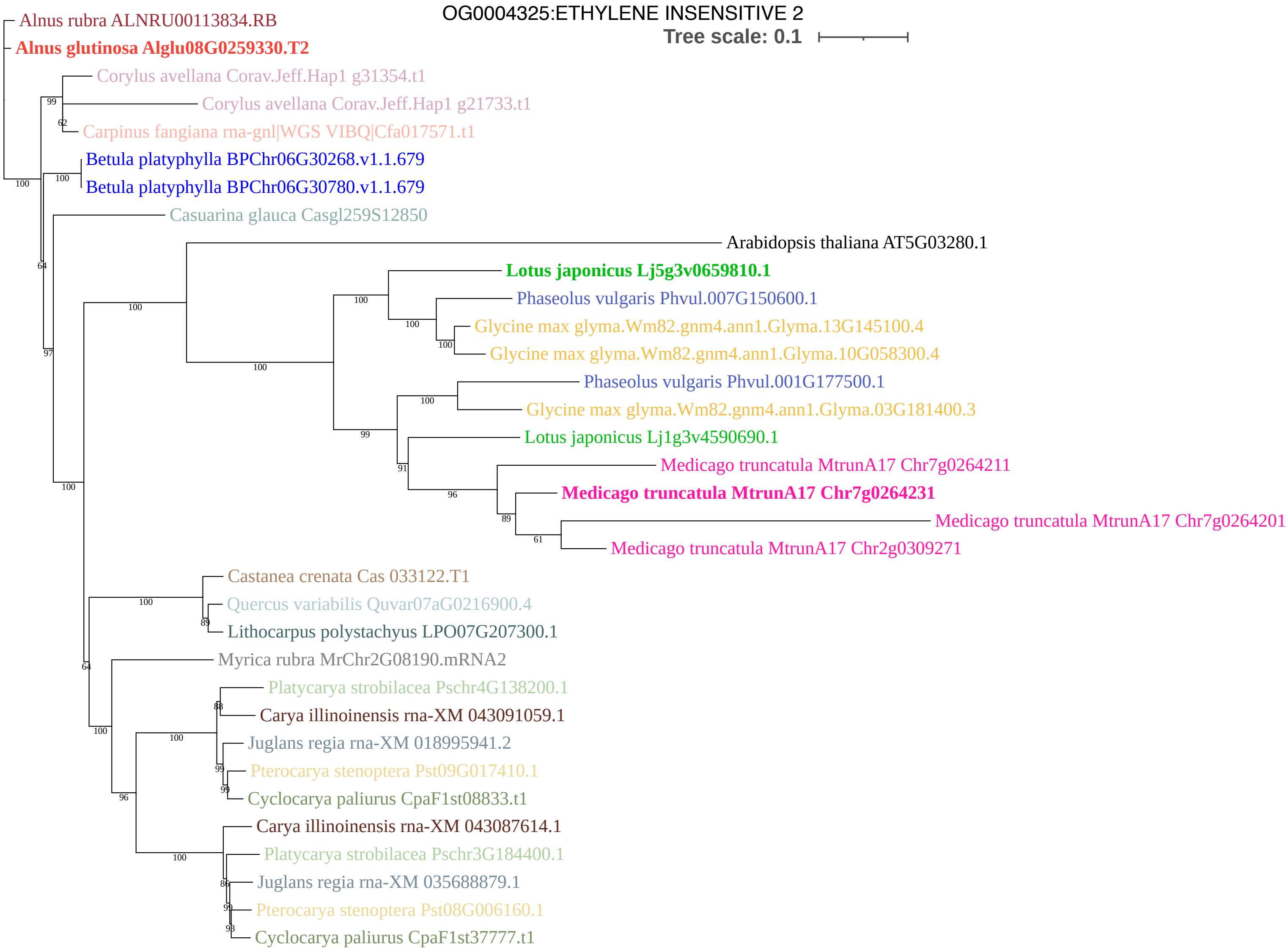
OG0004325:ETHYLENE INSENSITIVE 2
Tree scale: 0.1
Alnus rubra ALNRU00113834.RB
Alnus glutinosa Alglu08G0259330.T2
Corylus avellana Corav.Jeff.Hap1 g31354.t1
Corylus avellana Corav.Jeff.Hap1 g21733.t1
Carpinus fangiana rna-gnl|WGS VIBQ|Cfa017571.t1
Betula platyphylla BPChr06G30268.v1.1.679
Betula platyphylla BPChr06G30780.v1.1.679
Casuarina glauca Casgl259S12850
Arabidopsis thaliana AT5G03280.1
Lotus japonicus Lj5g3v0659810.1
Phaseolus vulgaris Phvul.007G150600.1
Glycine max glyma.Wm82.gnm4.ann1.Glyma.13G145100.4
Glycine max glyma.Wm82.gnm4.ann1.Glyma.10G058300.4
Phaseolus vulgaris Phvul.001G177500.1
Glycine max glyma.Wm82.gnm4.ann1.Glyma.03G181400.3
Lotus japonicus Lj1g3v4590690.1
Medicago truncatula MtrunA17 Chr7g0264211
Medicago truncatula MtrunA17 Chr7g0264231
Medicago truncatula MtrunA17 Chr7g0264201
Medicago truncatula MtrunA17 Chr2g0309271
Castanea crenata Cas 033122.T1
Quercus variabilis Quvar07aG0216900.4
Lithocarpus polystachyus LPO07G207300.1
Myrica rubra MrChr2G08190.mRNA2
Platycarya strobilacea Pschr4G138200.1
Carya illinoinensis rna-XM 043091059.1
Juglans regia rna-XM 018995941.2
Pterocarya stenoptera Pst09G017410.1
Cyclocarya paliurus CpaF1st08833.t1
Carya illinoinensis rna-XM 043087614.1
Platycarya strobilacea Pschr3G184400.1
Juglans regia rna-XM 035688879.1
Pterocarya stenoptera Pst08G006160.1
Cyclocarya paliurus CpaF1st37777.t1

OG0004403:CBL(Calcineurin B-like proteins)-interacting protein kinases

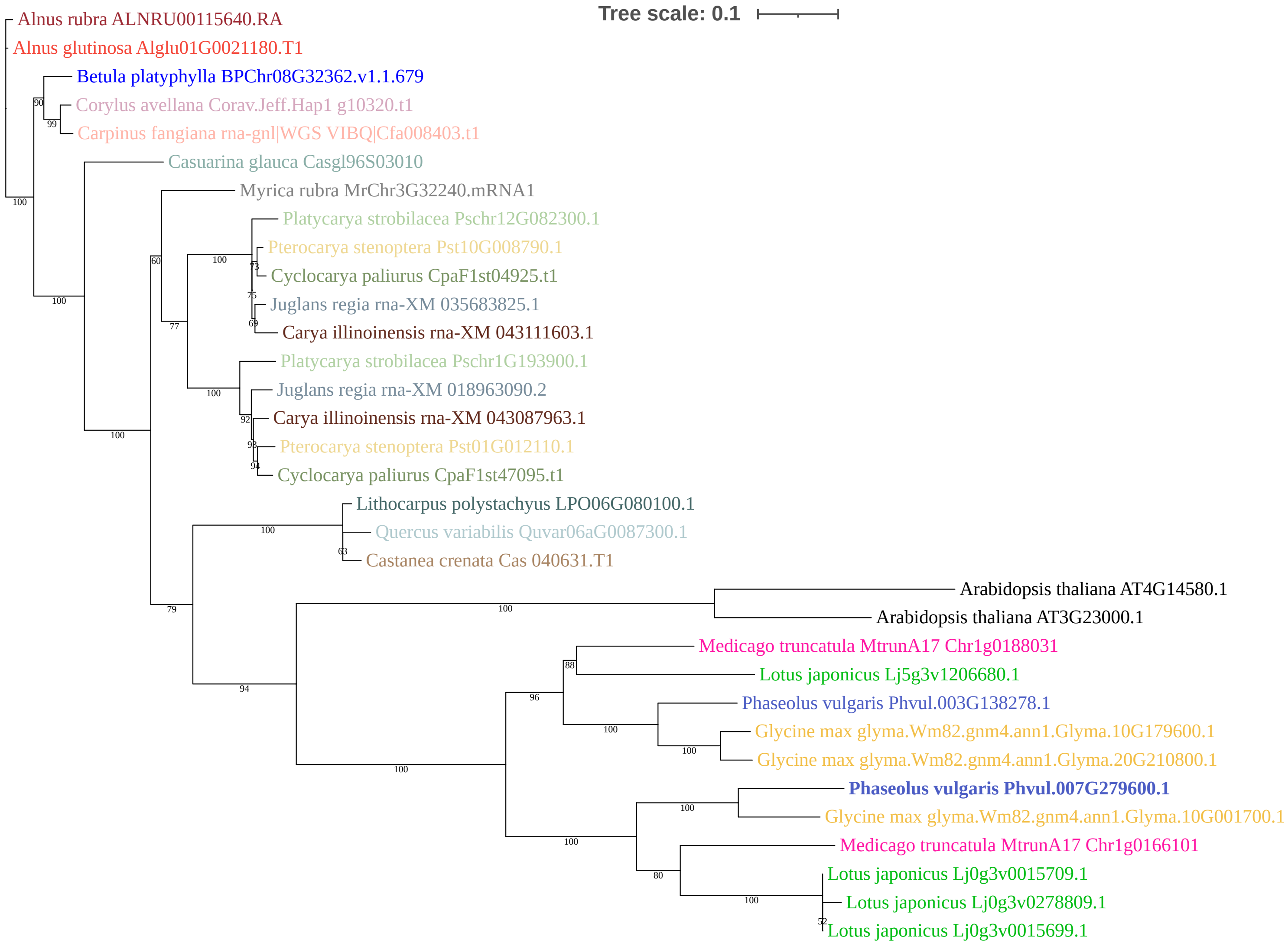

OG0004481:CALCIUM DEPENDANT PROTEIN KINASE 1

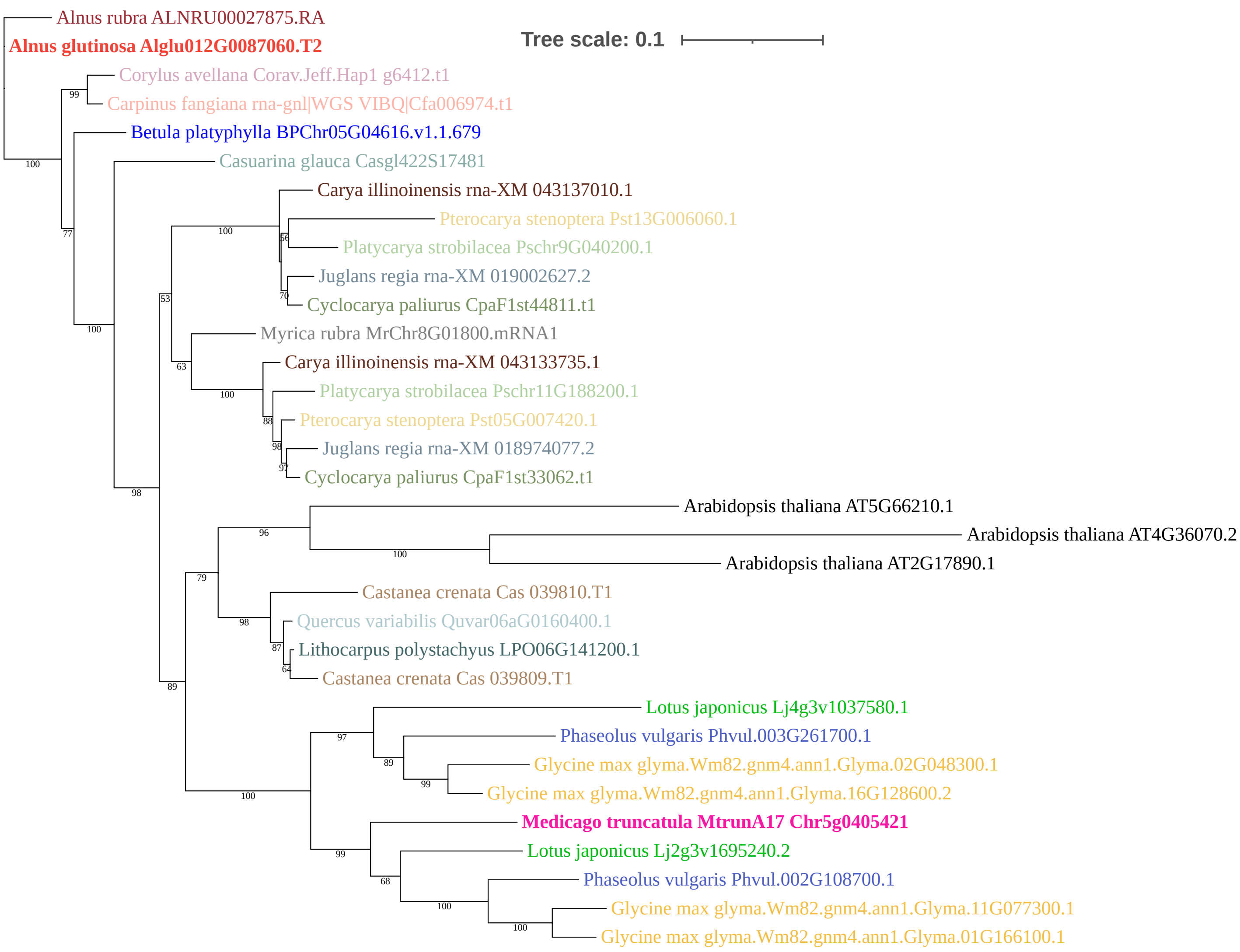

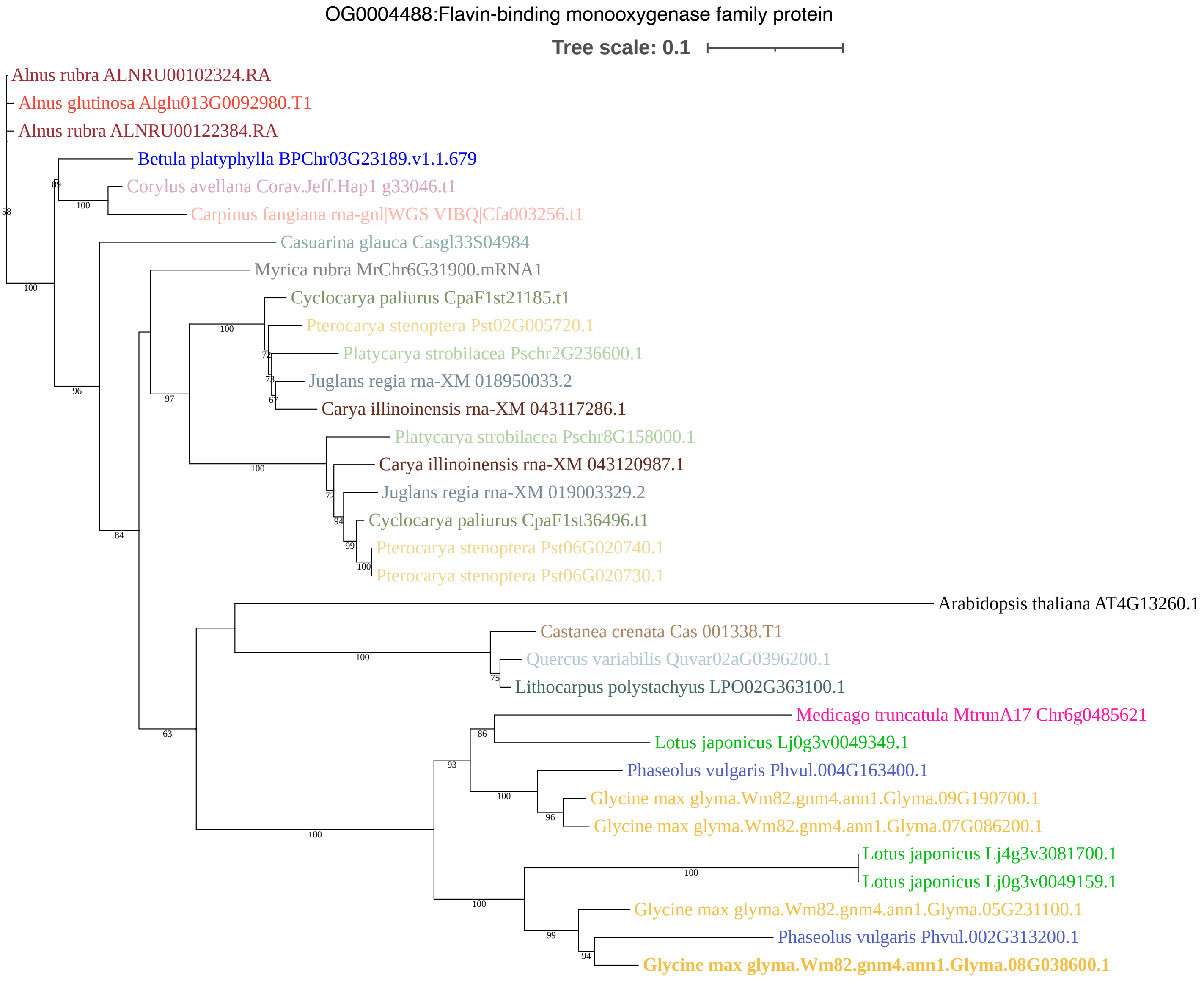
OG0004488:Flavin-binding monooxygenase family protein
Tree scale: 0.1
Alnus rubra ALNRU00102324.RA
Alnus glutinosa Alglu013G0092980.T1
Alnus rubra ALNRU00122384.RA
Betula platyphylla BPChr03G23189.v1.1.679
Corylus avellana Corav.Jeff.Hap1 g33046.t1
Carpinus fangiana rna-gnl|WGS VIBQ|Cfa003256.t1
Casuarina glauca Casgl33S04984
Myrica rubra MrChr6G31900.mRNA1
Cyclocarya paliurus CpaF1st21185.t1
Pterocarya stenoptera Pst02G005720.1
Platycarya strobilacea Pschr2G236600.1
Juglans regia rna-XM 018950033.2
Carya illinoinensis rna-XM 043117286.1
Platycarya strobilacea Pschr8G158000.1
Carya illinoinensis rna-XM 043120987.1
Juglans regia rna-XM 019003329.2
Cyclocarya paliurus CpaF1st36496.t1
Pterocarya stenoptera Pst06G020740.1
Pterocarya stenoptera Pst06G020730.1
Arabidopsis thaliana AT4G13260.1
Castanea crenata Cas 001338.T1
Quercus variabilis Quvar02aG0396200.1
Lithocarpus polystachyus LPO02G363100.1
Medicago truncatula MtrunA17 Chr6g0485621
Lotus japonicus Lj0g3v0049349.1
Phaseolus vulgaris Phvul.004G163400.1
Glycine max glyma.Wm82.gnm4.ann1.Glyma.09G190700.1
Glycine max glyma.Wm82.gnm4.ann1.Glyma.07G086200.1
Lotus japonicus Lj4g3v3081700.1
Lotus japonicus Lj0g3v0049159.1
Glycine max glyma.Wm82.gnm4.ann1.Glyma.05G231100.1
Phaseolus vulgaris Phvul.002G313200.1
Glycine max glyma.Wm82.gnm4.ann1.Glyma.08G038600.1

OG0004519:Targeting protein for Xklp2(TPX2) like protein

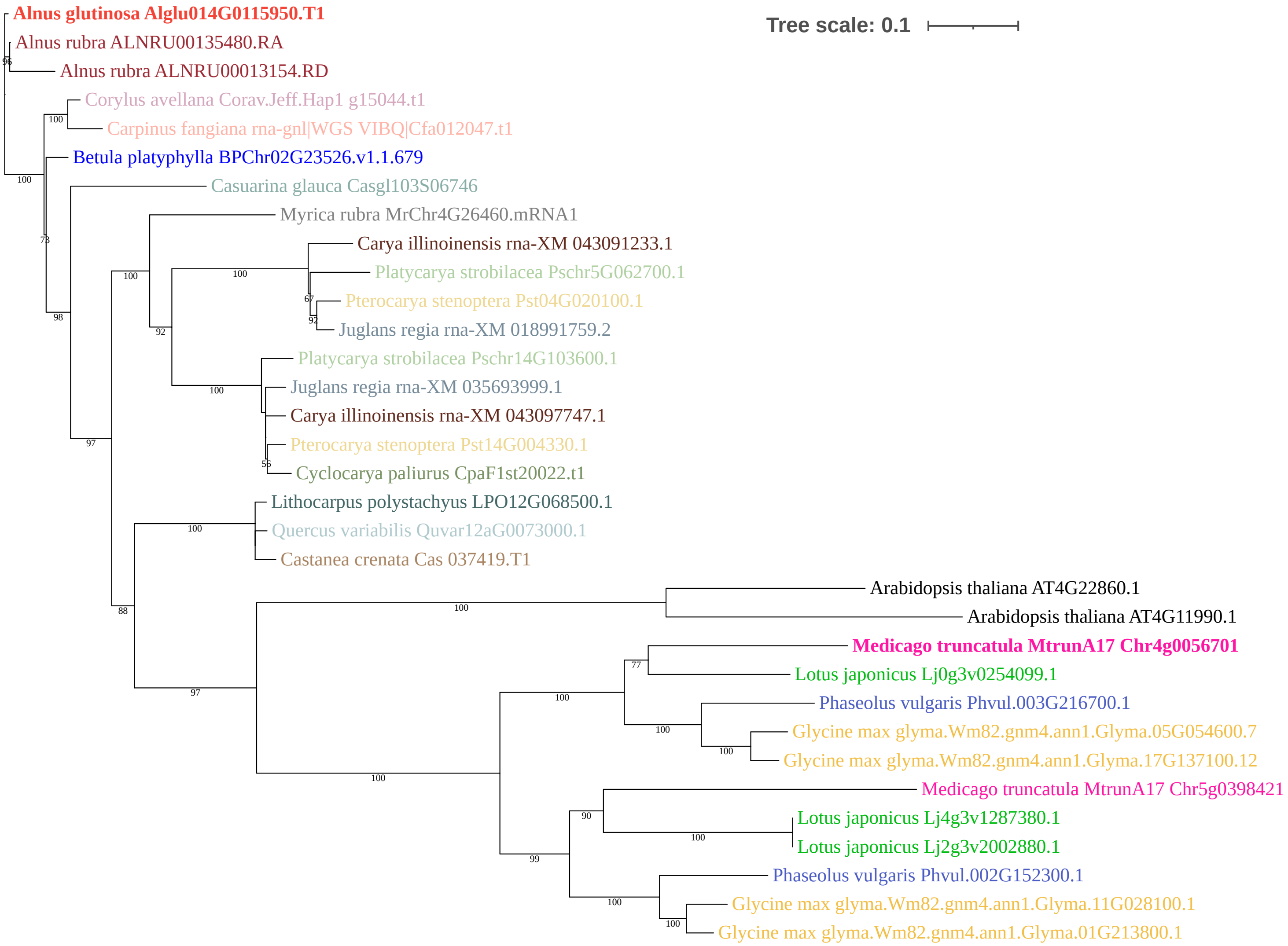

OG0004520:NIN-like protein 7a/NITRATE UNRESPONSIVE SYMBIOSIS 1

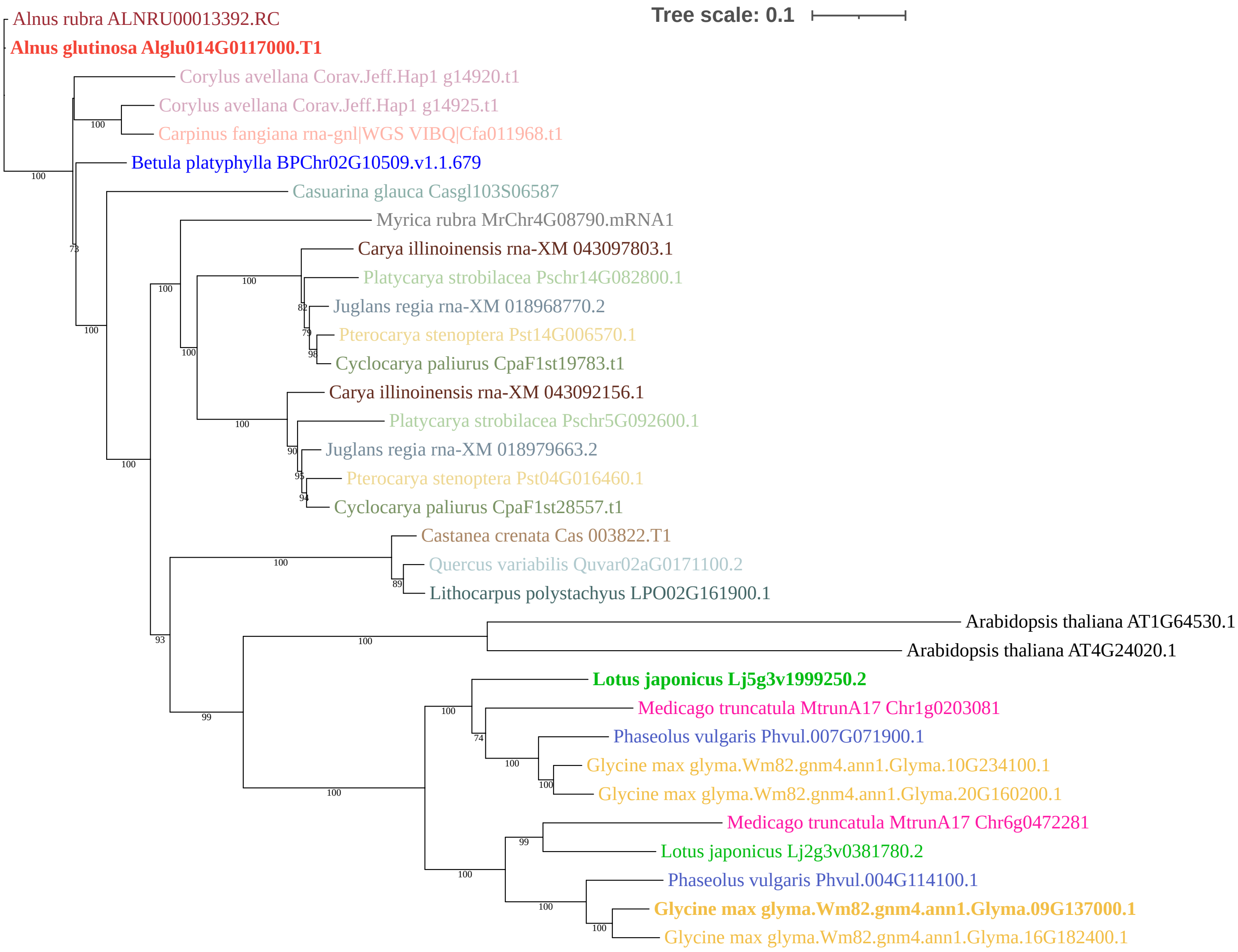

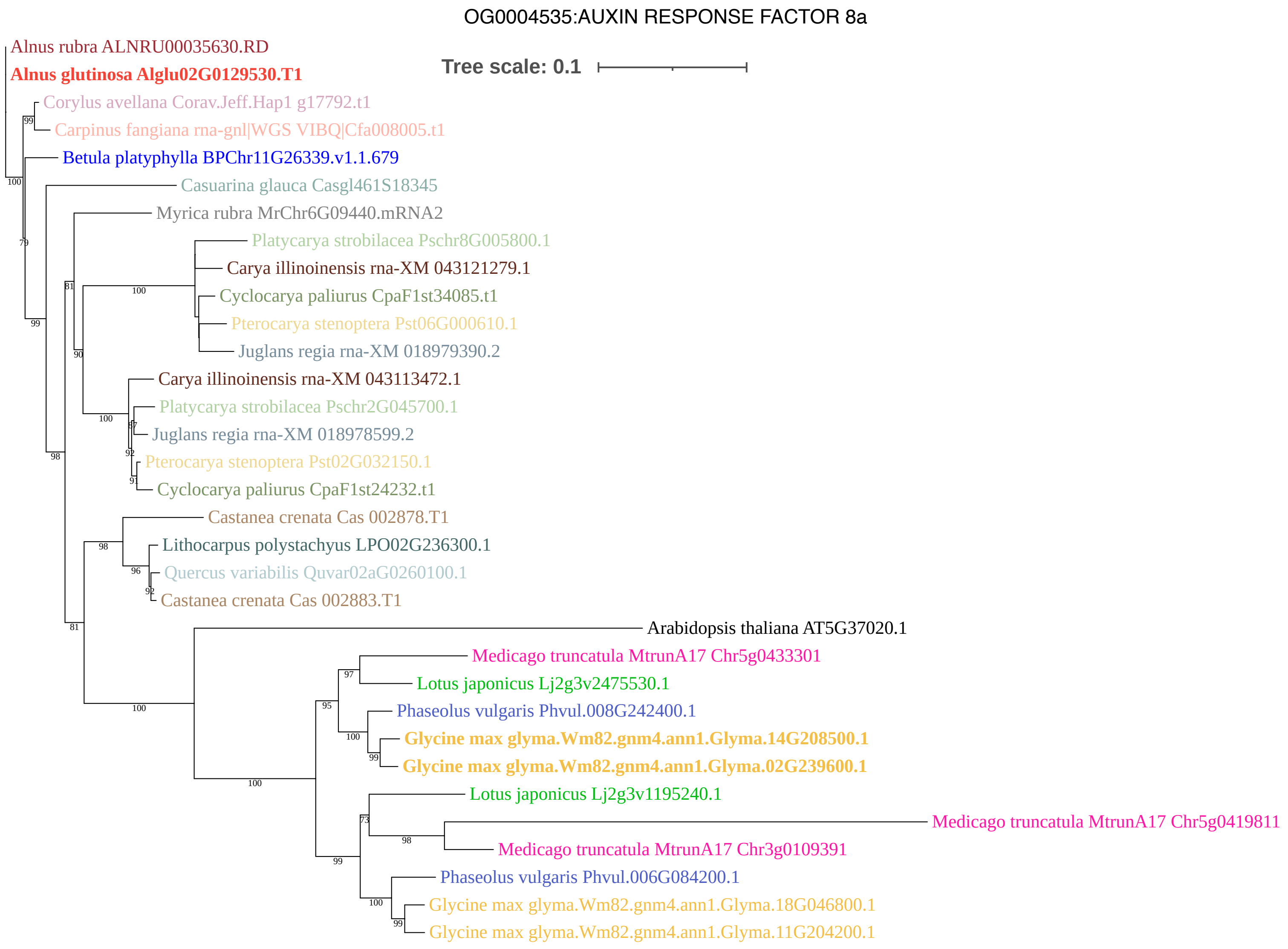
OG0004535:AUXIN RESPONSE FACTOR 8a
Tree scale: 0.1
Alnus rubra ALNRU00035630.RD
Alnus glutinosa Alglu02G0129530.T1
Corylus avellana Corav.Jeff.Hap1 g17792.t1
Carpinus fangiana rna-gnl|WGS VIBQ|Cfa008005.t1
Betula platyphylla BPChr11G26339.v1.1.679
Casuarina glauca Casgl461S18345
Myrica rubra MrChr6G09440.mRNA2
Platycarya strobilacea Pschr8G005800.1
Carya illinoinensis rna-XM 043121279.1
Cyclocarya paliurus CpaF1st34085.t1
Pterocarya stenoptera Pst06G000610.1
Juglans regia rna-XM 018979390.2
Carya illinoinensis rna-XM 043113472.1
Platycarya strobilacea Pschr2G045700.1
Juglans regia rna-XM 018978599.2
Pterocarya stenoptera Pst02G032150.1
Cyclocarya paliurus CpaF1st24232.t1
Castanea crenata Cas 002878.T1
Lithocarpus polystachyus LPO02G236300.1
Quercus variabilis Quvar02aG0260100.1
Castanea crenata Cas 002883.T1
Arabidopsis thaliana AT5G37020.1
Medicago truncatula MtrunA17 Chr5g0433301
Lotus japonicus Lj2g3v2475530.1
Phaseolus vulgaris Phvul.008G242400.1
Glycine max glyma.Wm82.gnm4.ann1.Glyma.14G208500.1
Glycine max glyma.Wm82.gnm4.ann1.Glyma.02G239600.1
Lotus japonicus Lj2g3v1195240.1
Medicago truncatula MtrunA17 Chr5g0419811
Medicago truncatula MtrunA17 Chr3g0109391
Phaseolus vulgaris Phvul.006G084200.1
Glycine max glyma.Wm82.gnm4.ann1.Glyma.18G046800.1
Glycine max glyma.Wm82.gnm4.ann1.Glyma.11G204200.1

OG0004580:SYMBIOTIC RECEPTOR KINASE like 1

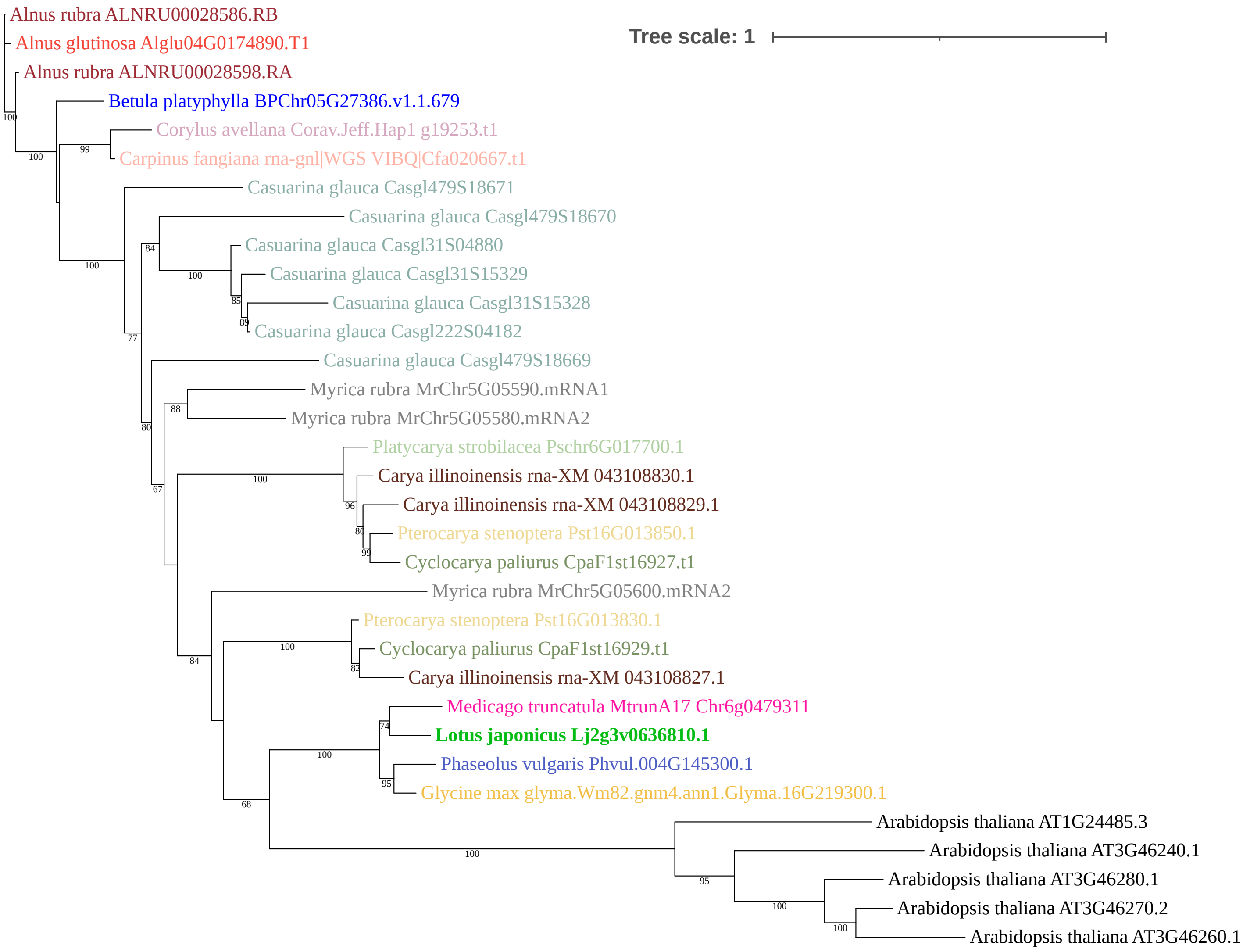

OG0004646:NUCLEAR FACTOR YA1

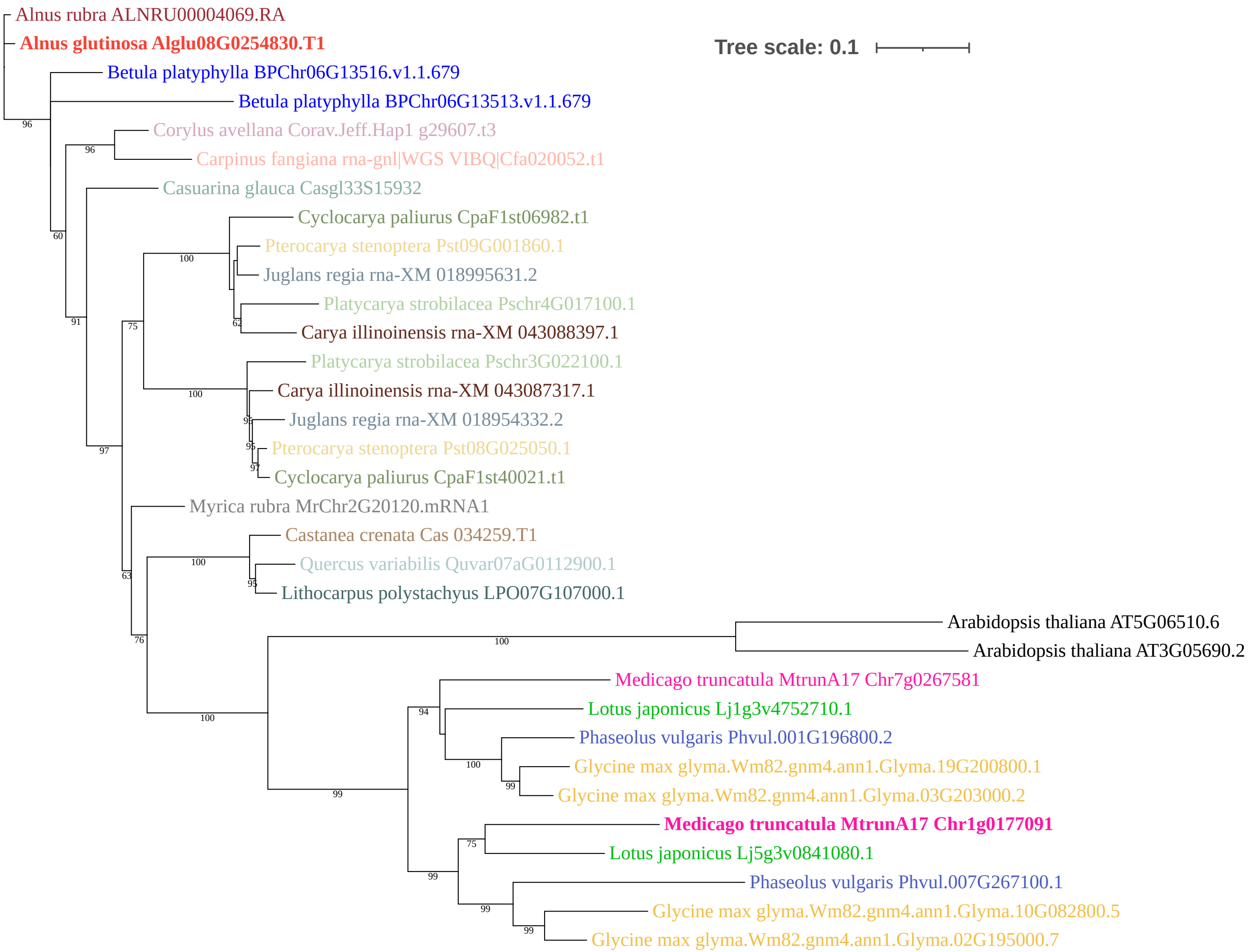

OG0004659:SCAR-Nodulation(SCAR/WAVE actin regulatory complex)|altered nodule primordia invasion

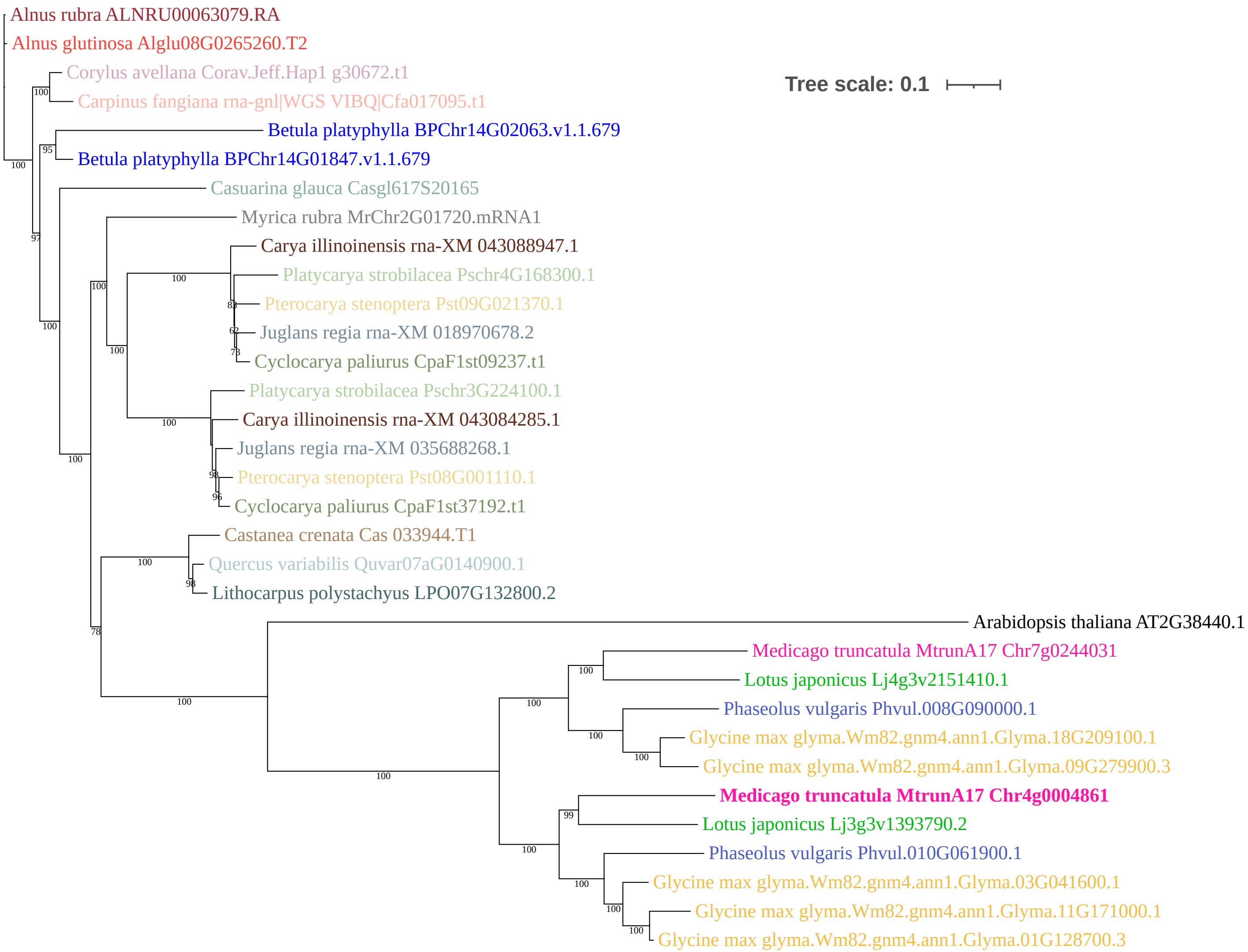

OG0004713

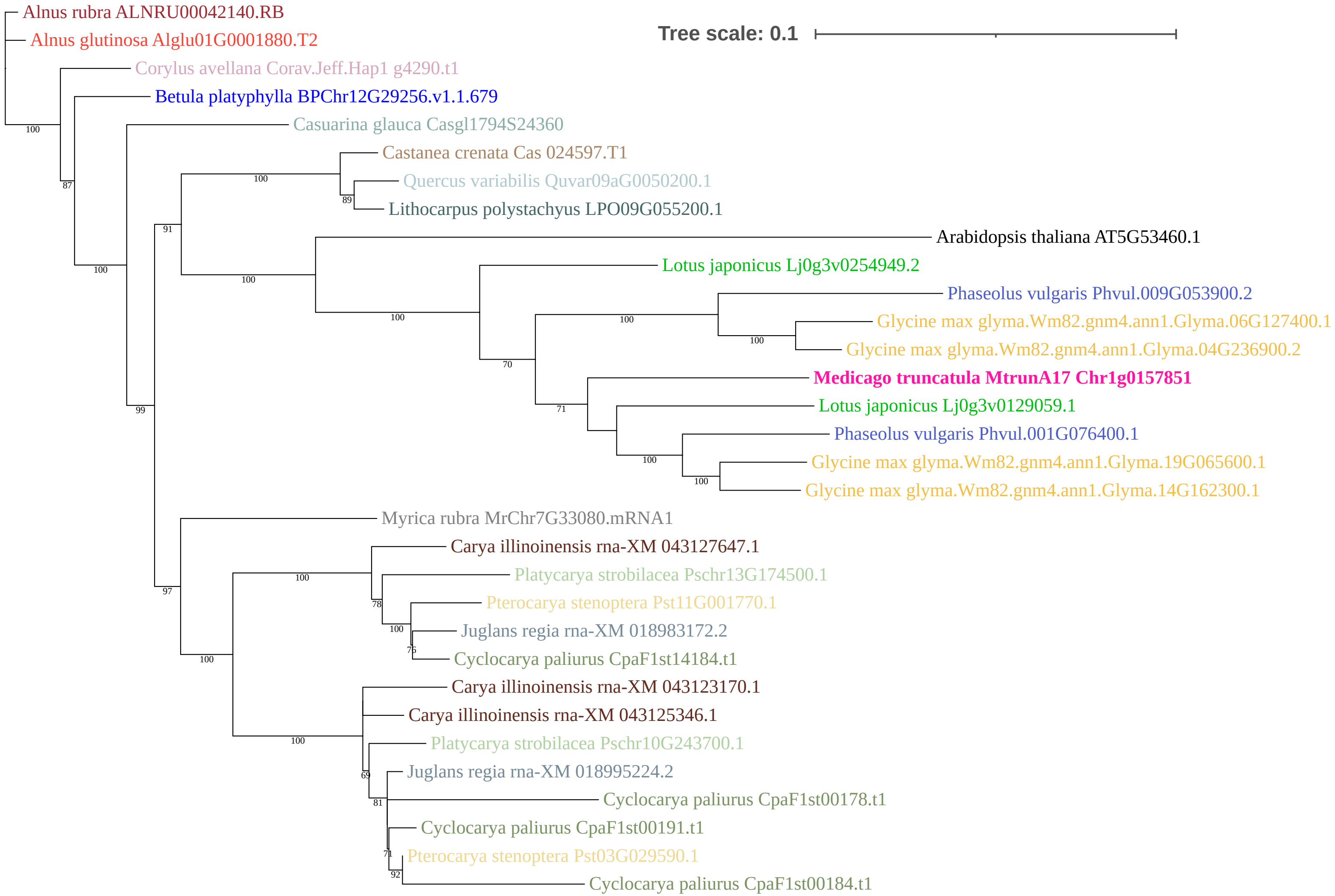
Tree scale: 0.1
Alnus rubra ALNRU00042140.RB
Alnus glutinosa Alglu01G0001880.T2
Corylus avellana Corav.Jeff.Hap1 g4290.t1
Betula platyphylla BPChr12G29256.v1.1.679
Casuarina glauca Casgl1794S24360
Castanea crenata Cas 024597.T1
Quercus variabilis Quvar09aG0050200.1
Lithocarpus polystachyus LPO09G055200.1
Arabidopsis thaliana AT5G53460.1
Lotus japonicus Lj0g3v0254949.2
Phaseolus vulgaris Phvul.009G053900.2
Glycine max glyma.Wm82.gnm4.ann1.Glyma.06G127400.1
Glycine max glyma.Wm82.gnm4.ann1.Glyma.04G236900.2
Medicago truncatula MtrunA17 Chr1g0157851
Lotus japonicus Lj0g3v0129059.1
Phaseolus vulgaris Phvul.001G076400.1
Glycine max glyma.Wm82.gnm4.ann1.Glyma.19G065600.1
Glycine max glyma.Wm82.gnm4.ann1.Glyma.14G162300.1
Myrica rubra MrChr7G33080.mRNA1
Carya illinoinensis rna-XM 043127647.1
Platycarya strobilacea Pschr13G174500.1
Pterocarya stenoptera Pst11G001770.1
Juglans regia rna-XM 018983172.2
Cyclocarya paliurus CpaF1st14184.t1
Carya illinoinensis rna-XM 043123170.1
Carya illinoinensis rna-XM 043125346.1
Platycarya strobilacea Pschr10G243700.1
Juglans regia rna-XM 018995224.2
Cyclocarya paliurus CpaF1st00178.t1
Cyclocarya paliurus CpaF1st00191.t1
Pterocarya stenoptera Pst03G029590.1
Cyclocarya paliurus CpaF1st00184.t1

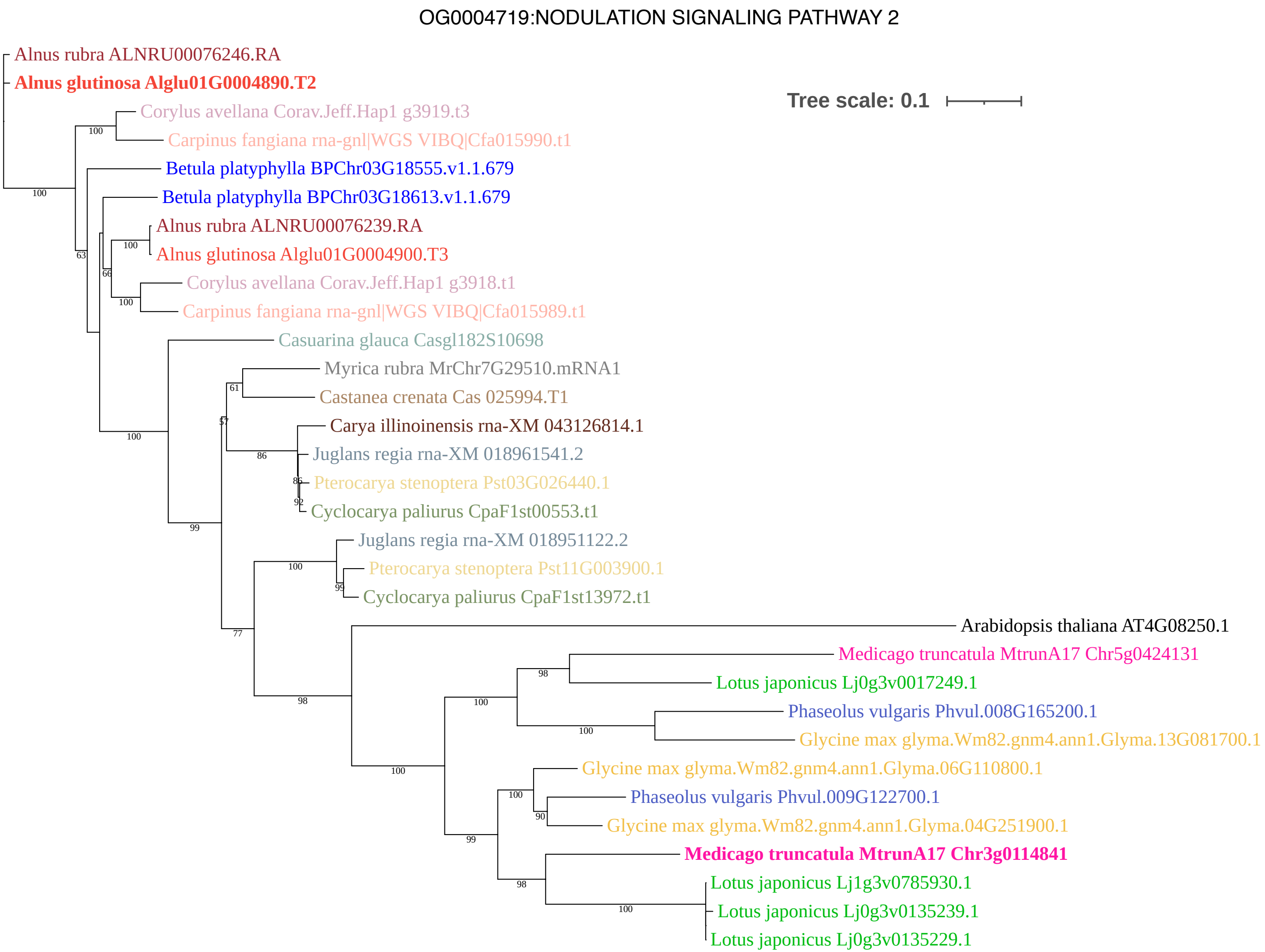
OG0004719:NODULATION SIGNALING PATHWAY 2
Tree scale: 0.1
Alnus rubra ALNRU00076246.RA
Alnus glutinosa Alglu01G0004890.T2
Corylus avellana Corav.Jeff.Hap1 g3919.t3
Carpinus fangiana rna-gnl|WGS VIBQ|Cfa015990.t1
Betula platyphylla BPChr03G18555.v1.1.679
Betula platyphylla BPChr03G18613.v1.1.679
Alnus rubra ALNRU00076239.RA
Alnus glutinosa Alglu01G0004900.T3
Corylus avellana Corav.Jeff.Hap1 g3918.t1
Carpinus fangiana rna-gnl|WGS VIBQ|Cfa015989.t1
Casuarina glauca Casgl182S10698
Myrica rubra MrChr7G29510.mRNA1
Castanea crenata Cas 025994.T1
Carya illinoinensis rna-XM 043126814.1
Juglans regia rna-XM 018961541.2
Pterocarya stenoptera Pst03G026440.1
Cyclocarya paliurus CpaF1st00553.t1
Juglans regia rna-XM 018951122.2
Pterocarya stenoptera Pst11G003900.1
Cyclocarya paliurus CpaF1st13972.t1
Arabidopsis thaliana AT4G08250.1
Medicago truncatula MtrunA17 Chr5g0424131
Lotus japonicus Lj0g3v0017249.1
Phaseolus vulgaris Phvul.008G165200.1
Glycine max glyma.Wm82.gnm4.ann1.Glyma.13G081700.1
Glycine max glyma.Wm82.gnm4.ann1.Glyma.06G110800.1
Phaseolus vulgaris Phvul.009G122700.1
Glycine max glyma.Wm82.gnm4.ann1.Glyma.04G251900.1
Medicago truncatula MtrunA17 Chr3g0114841
Lotus japonicus Lj1g3v0785930.1
Lotus japonicus Lj0g3v0135239.1
Lotus japonicus Lj0g3v0135229.1

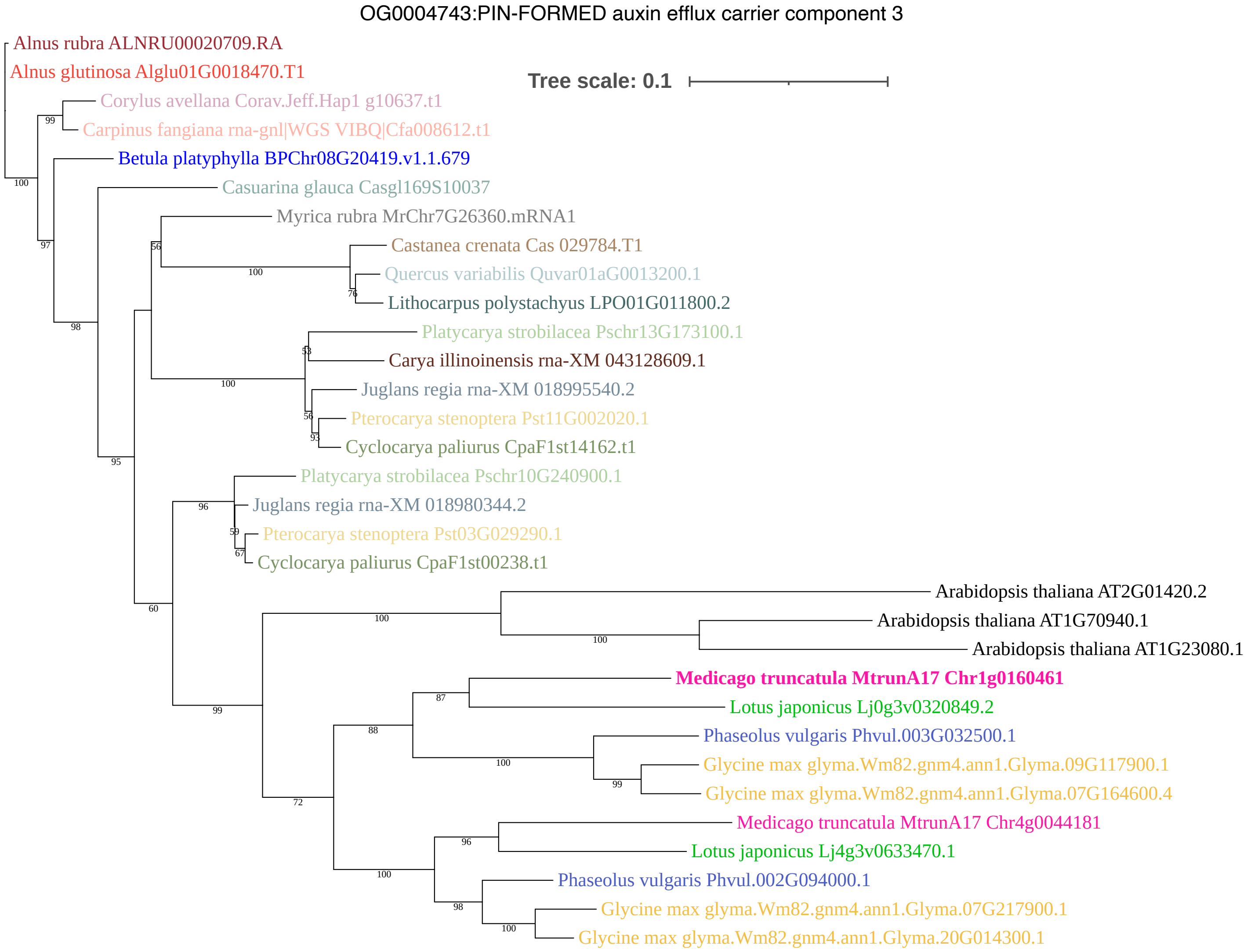
OG0004743:PIN-FORMED auxin efflux carrier component 3
Tree scale: 0.1
Alnus rubra ALNRU00020709.RA
Alnus glutinosa Alglu01G0018470.T1
Corylus avellana Corav.Jeff.Hap1 g10637.t1
Carpinus fangiana rna-gnl|WGS VIBQ|Cfa008612.t1
Betula platyphylla BPChr08G20419.v1.1.679
Casuarina glauca Casgl169S10037
Myrica rubra MrChr7G26360.mRNA1
Castanea crenata Cas 029784.T1
Quercus variabilis Quvar01aG0013200.1
Lithocarpus polystachyus LPO01G011800.2
Platycarya strobilacea Pschr13G173100.1
Carya illinoinensis rna-XM 043128609.1
Juglans regia rna-XM 018995540.2
Pterocarya stenoptera Pst11G002020.1
Cyclocarya paliurus CpaF1st14162.t1
Platycarya strobilacea Pschr10G240900.1
Juglans regia rna-XM 018980344.2
Pterocarya stenoptera Pst03G029290.1
Cyclocarya paliurus CpaF1st00238.t1
Arabidopsis thaliana AT2G01420.2
Arabidopsis thaliana AT1G70940.1
Arabidopsis thaliana AT1G23080.1
Medicago truncatula MtrunA17 Chr1g0160461
Lotus japonicus Lj0g3v0320849.2
Phaseolus vulgaris Phvul.003G032500.1
Glycine max glyma.Wm82.gnm4.ann1.Glyma.09G117900.1
Glycine max glyma.Wm82.gnm4.ann1.Glyma.07G164600.4
Medicago truncatula MtrunA17 Chr4g0044181
Lotus japonicus Lj4g3v0633470.1
Phaseolus vulgaris Phvul.002G094000.1
Glycine max glyma.Wm82.gnm4.ann1.Glyma.07G217900.1
Glycine max glyma.Wm82.gnm4.ann1.Glyma.20G014300.1

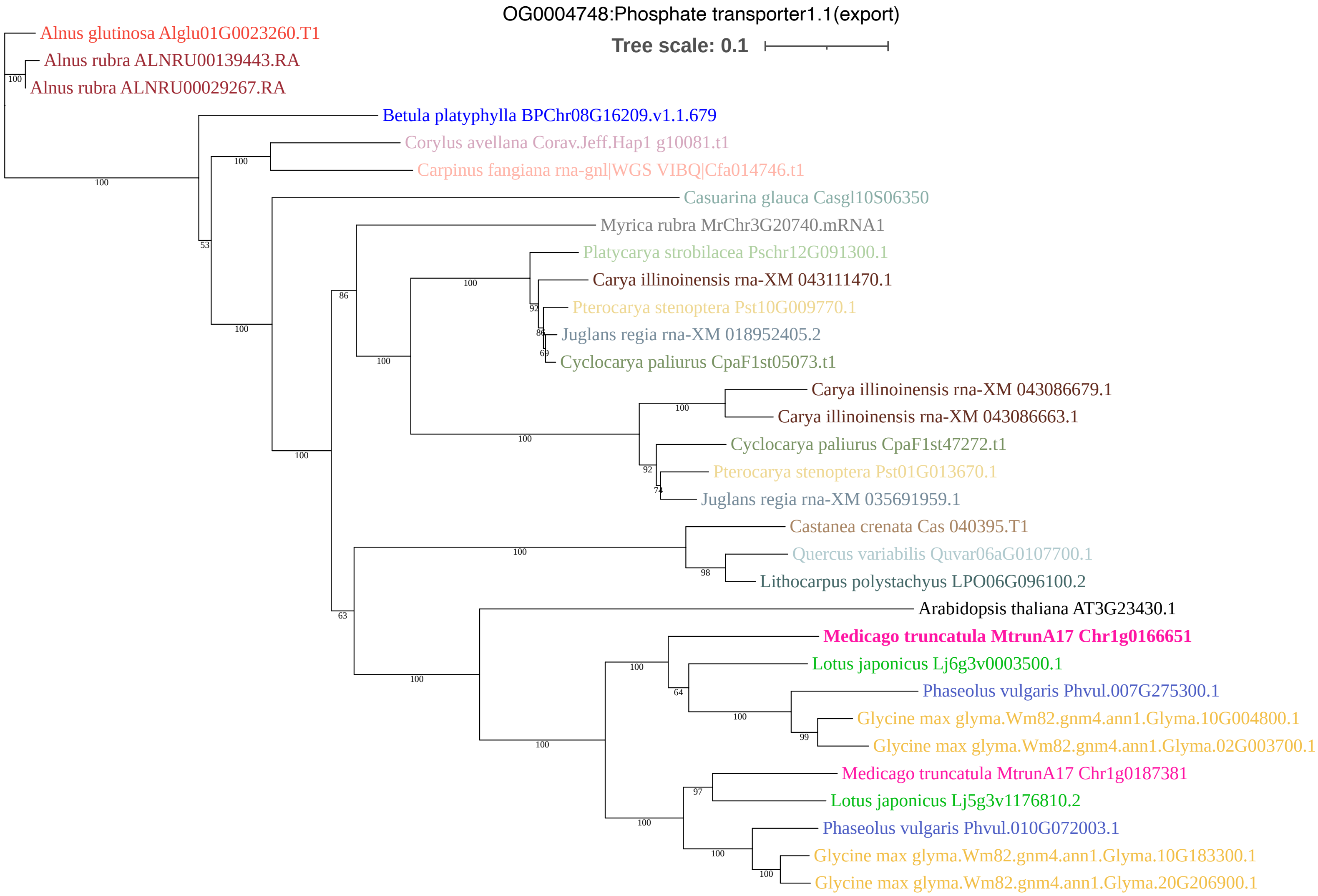
OG0004748:Phosphate transporter1.1(export)
Tree scale: 0.1
Alnus glutinosa Alglu01G0023260.T1
Alnus rubra ALNRU00139443.RA
Alnus rubra ALNRU00029267.RA
Betula platyphylla BPChr08G16209.v1.1.679
Corylus avellana Corav.Jeff.Hap1 g10081.t1
Carpinus fangiana rna-gnl|WGS VIBQ|Cfa014746.t1
Casuarina glauca Casgl10S06350
Myrica rubra MrChr3G20740.mRNA1
Platycarya strobilacea Pschr12G091300.1
Carya illinoinensis rna-XM 043111470.1
Pterocarya stenoptera Pst10G009770.1
Juglans regia rna-XM 018952405.2
Cyclocarya paliurus CpaF1st05073.t1
Carya illinoinensis rna-XM 043086679.1
Carya illinoinensis rna-XM 043086663.1
Cyclocarya paliurus CpaF1st47272.t1
Pterocarya stenoptera Pst01G013670.1
Juglans regia rna-XM 035691959.1
Castanea crenata Cas 040395.T1
Quercus variabilis Quvar06aG0107700.1
Lithocarpus polystachyus LPO06G096100.2
Arabidopsis thaliana AT3G23430.1
Medicago truncatula MtrunA17 Chr1g0166651
Lotus japonicus Lj6g3v0003500.1
Phaseolus vulgaris Phvul.007G275300.1
Glycine max glyma.Wm82.gnm4.ann1.Glyma.10G004800.1
Glycine max glyma.Wm82.gnm4.ann1.Glyma.02G003700.1
Medicago truncatula MtrunA17 Chr1g0187381
Lotus japonicus Lj5g3v1176810.2
Phaseolus vulgaris Phvul.010G072003.1
Glycine max glyma.Wm82.gnm4.ann1.Glyma.10G183300.1
Glycine max glyma.Wm82.gnm4.ann1.Glyma.20G206900.1

OG0004762:NRT1/ PTR FAMILY|nitrate transporter / peptide transporter

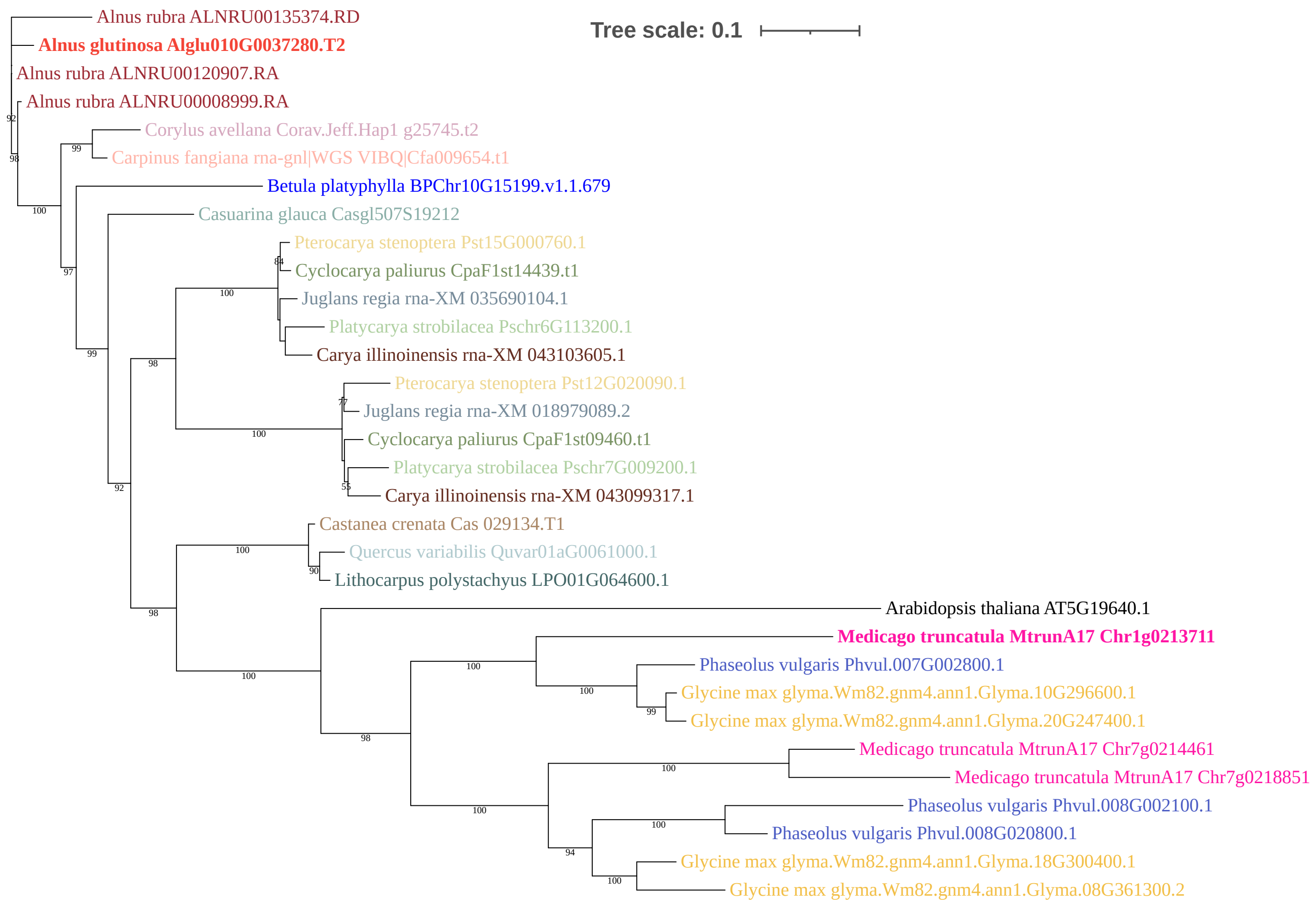

OG0004793:GLUTAMINE PHOSPHORIBOSYL PYROPHOSPHATE AMIDOTRANSFERASE 3

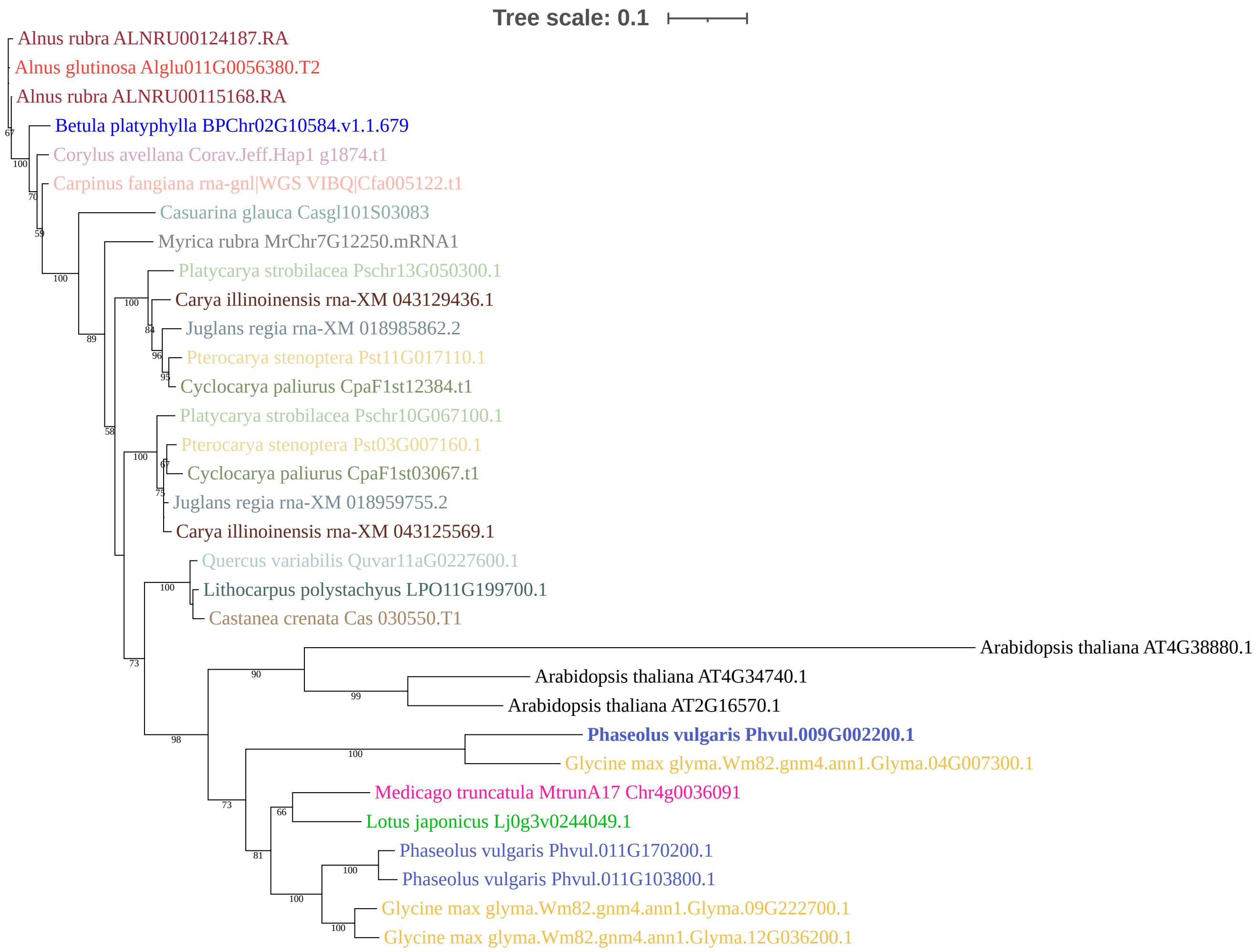

OG0004918:RECEPTOR FOR ACTIVATED C KINASE 1a/1b/1

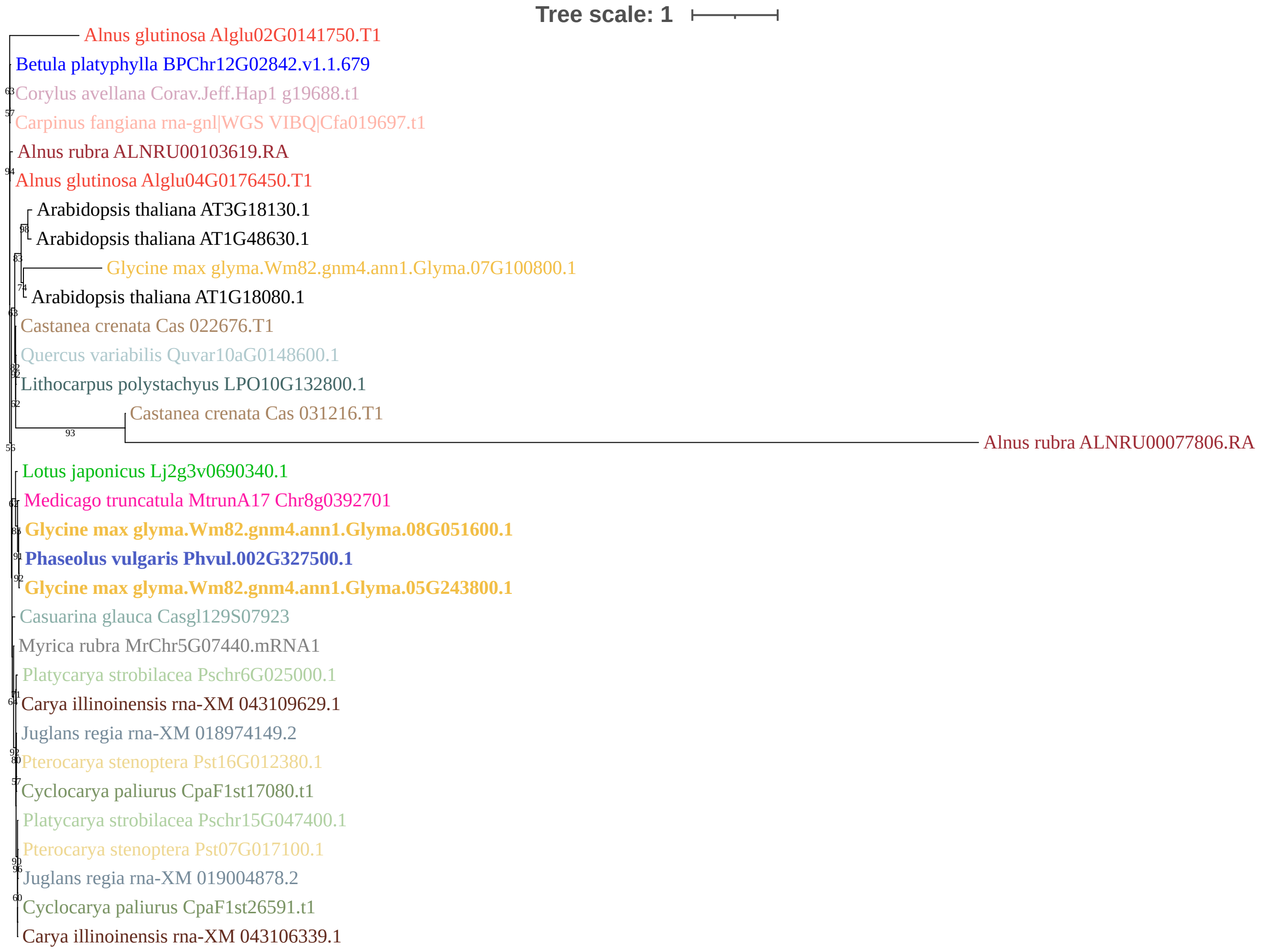

OG0004923:SHAGGY LIKE KINASE

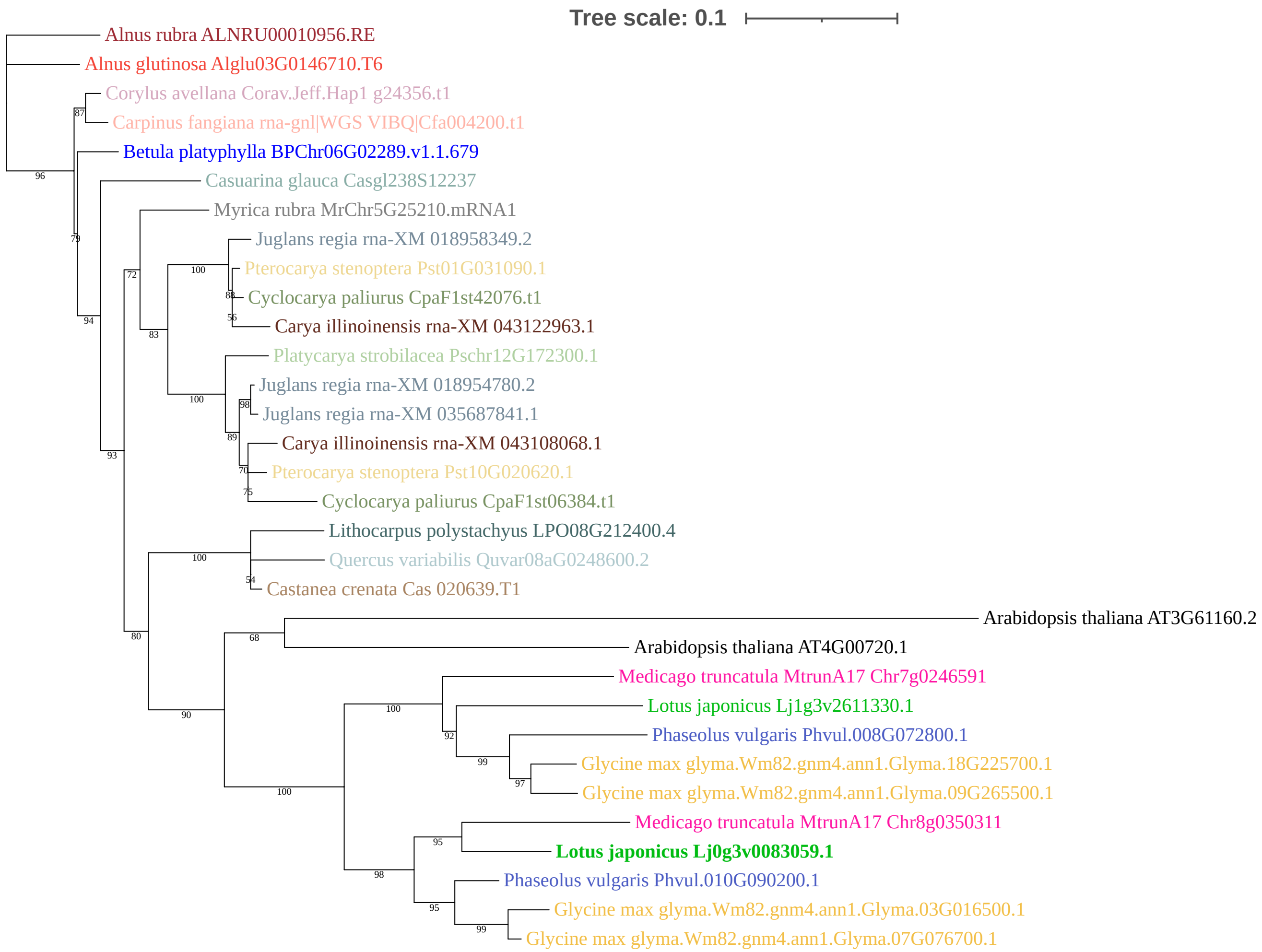

OG0004967:PIN-FORMED auxin efflux carrier component 1b/1c/1a/4

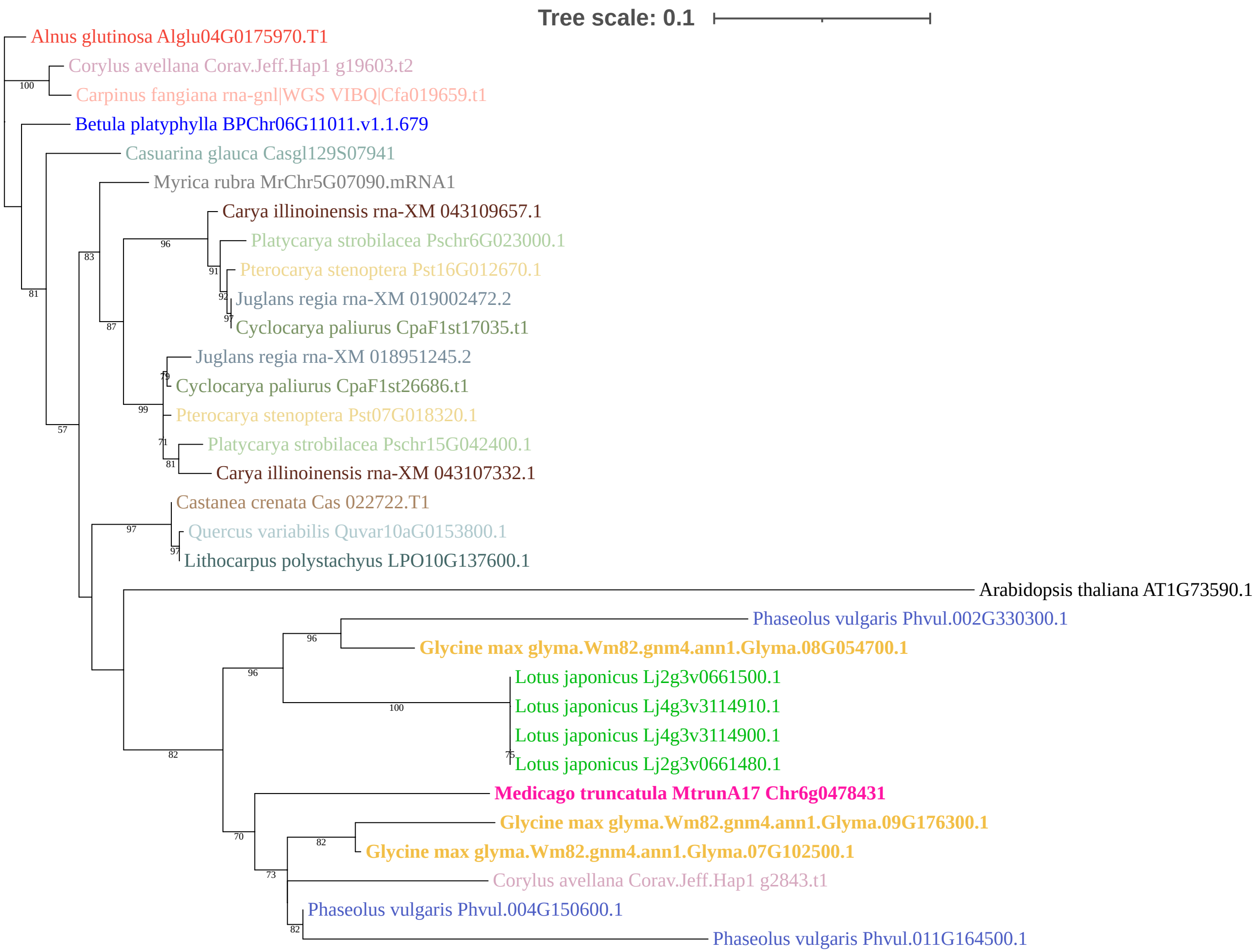

OG0005101:COPPER TRASPORTER 1

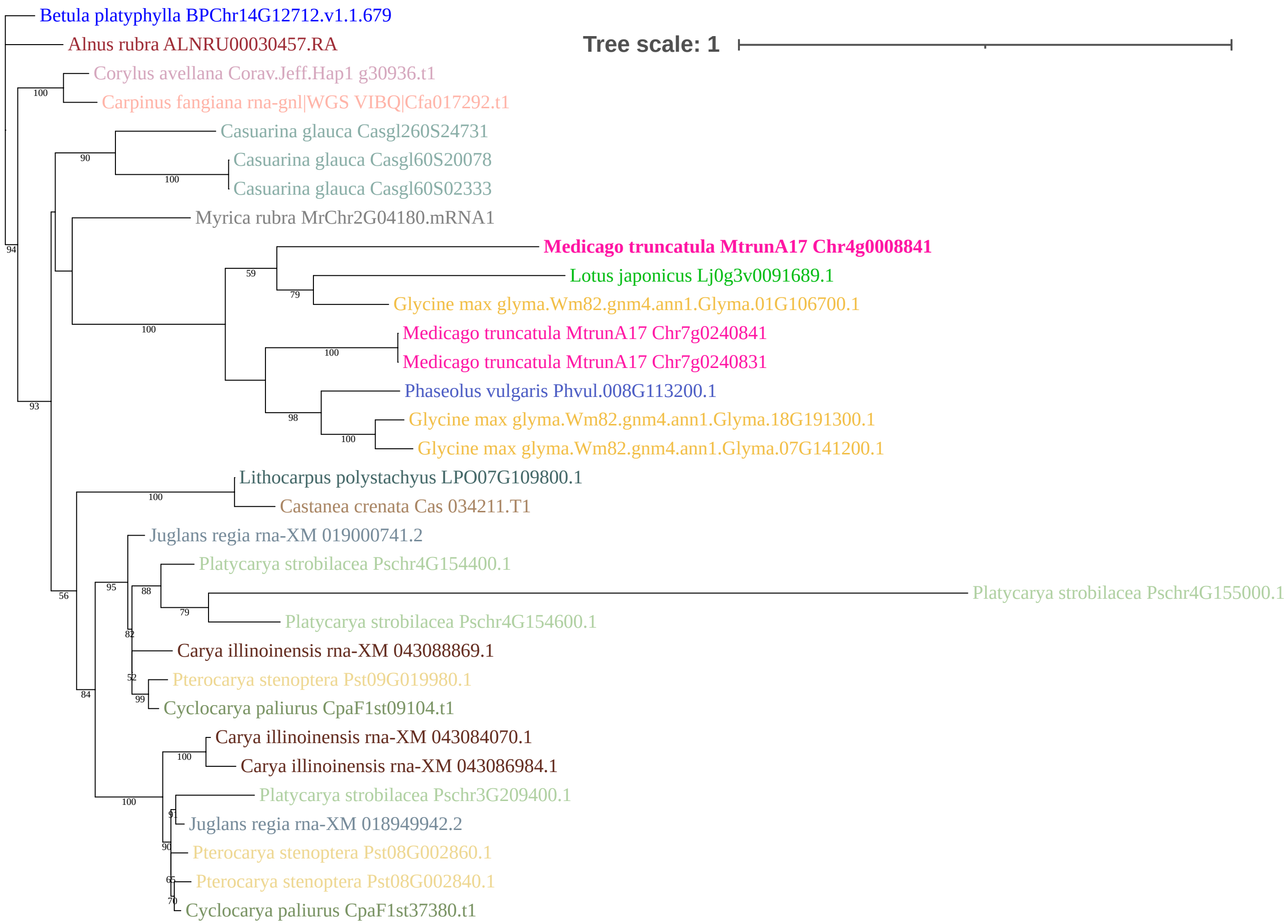

OG0005192:β-1,3 -GLUCANASE

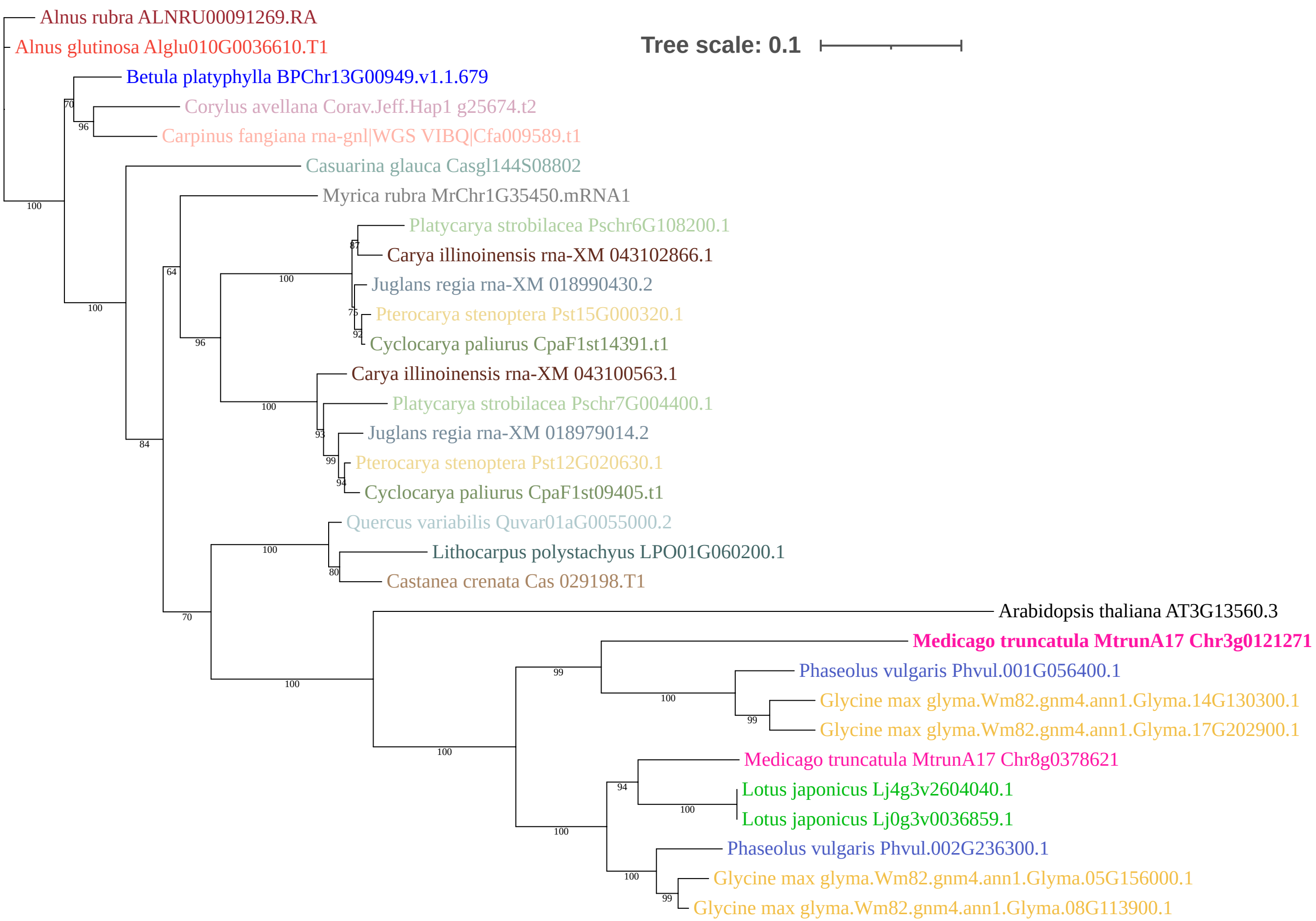

OG0005203:NODULE NUMBER CONTROL 1

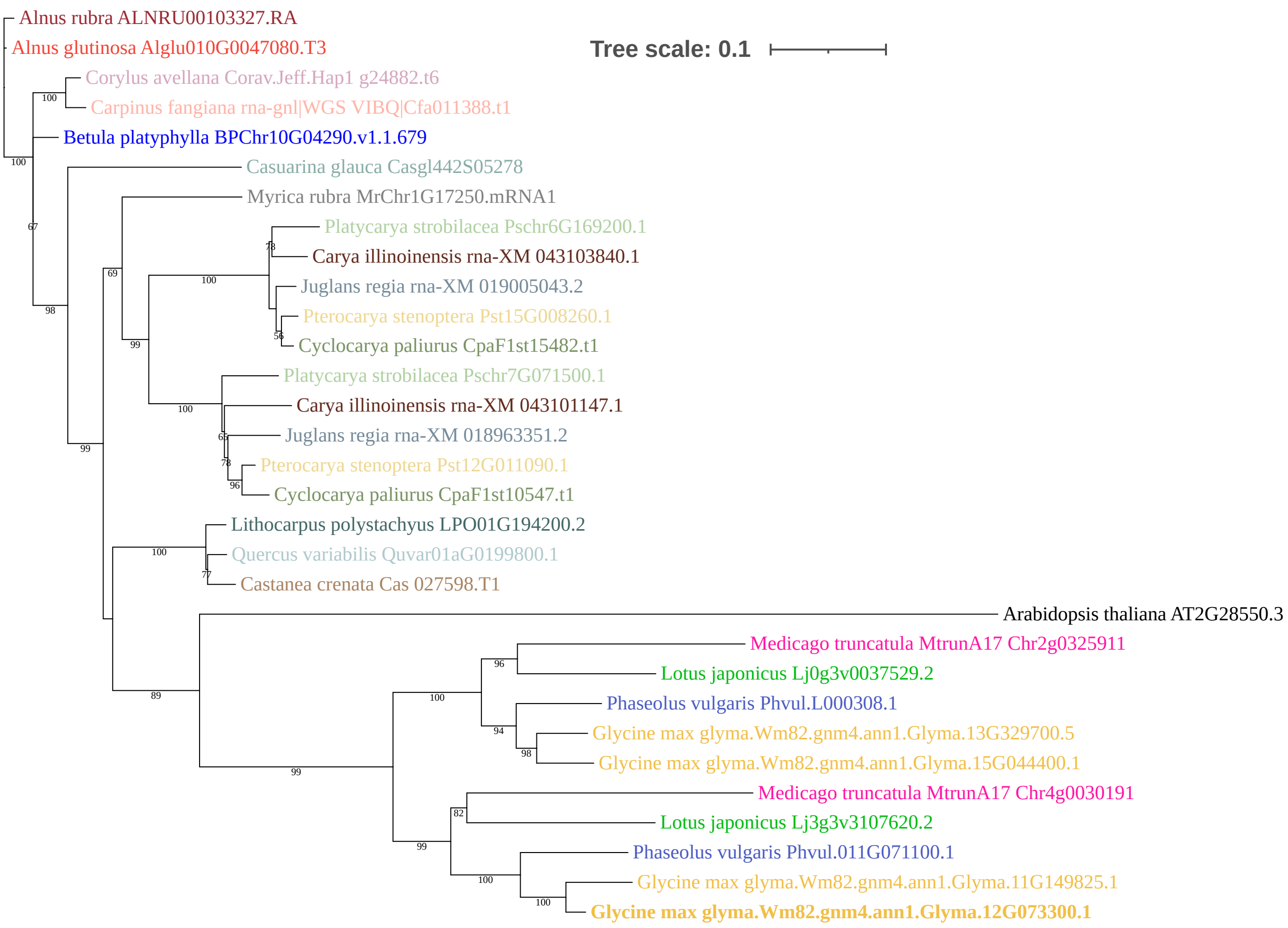

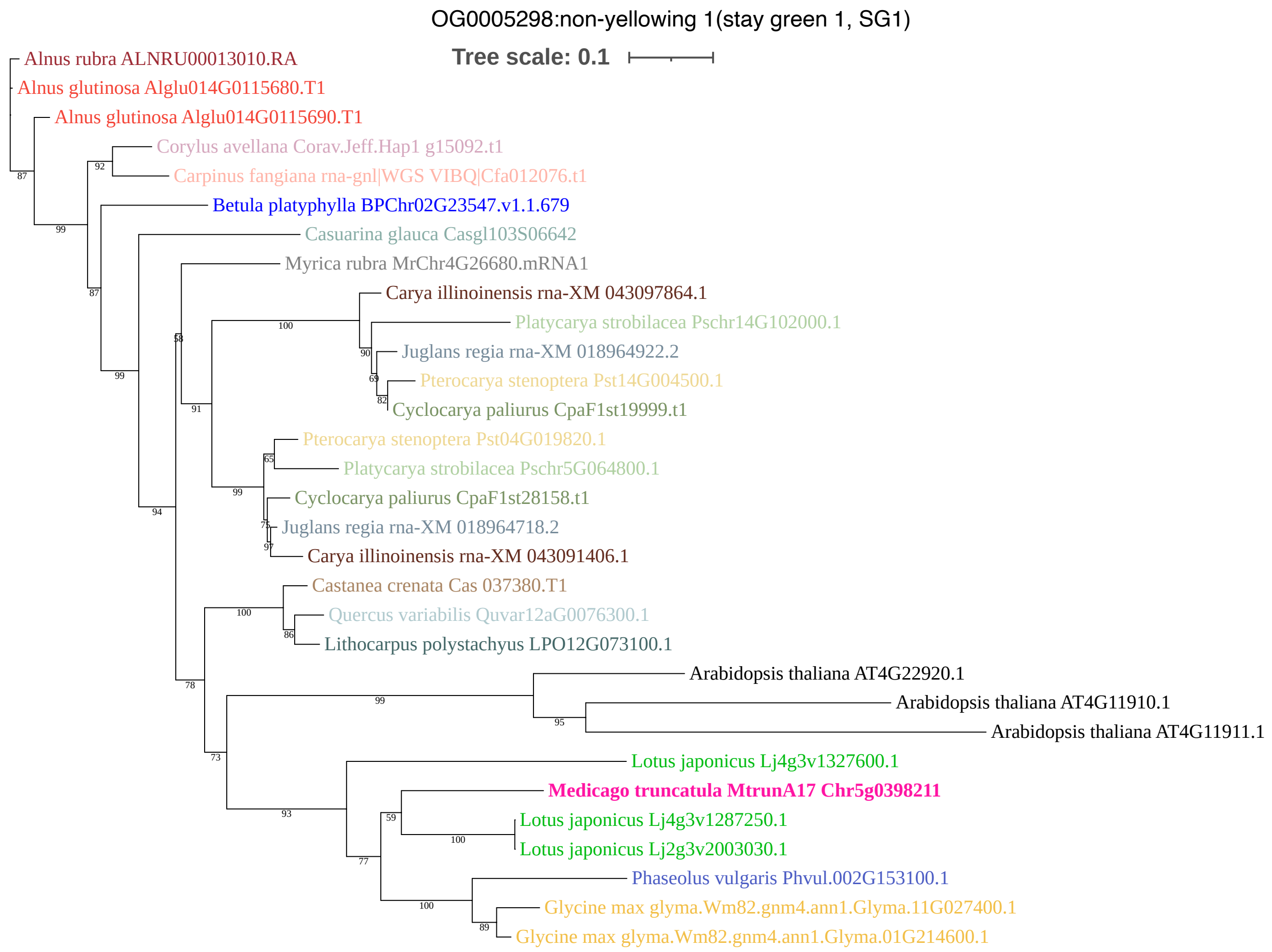
OG0005298:non-yellowing 1(stay green 1, SG1)
Tree scale: 0.1
Alnus rubra ALNRU00013010.RA
Alnus glutinosa Alglu014G0115680.T1
Alnus glutinosa Alglu014G0115690.T1
Corylus avellana Corav.Jeff.Hap1 g15092.t1
Carpinus fangiana rna-gnl|WGS VIBQ|Cfa012076.t1
Betula platyphylla BPChr02G23547.v1.1.679
Casuarina glauca Casgl103S06642
Myrica rubra MrChr4G26680.mRNA1
Carya illinoinensis rna-XM 043097864.1
Platycarya strobilacea Pschr14G102000.1
Juglans regia rna-XM 018964922.2
Pterocarya stenoptera Pst14G004500.1
Cyclocarya paliurus CpaF1st19999.t1
Pterocarya stenoptera Pst04G019820.1
Platycarya strobilacea Pschr5G064800.1
Cyclocarya paliurus CpaF1st28158.t1
Juglans regia rna-XM 018964718.2
Carya illinoinensis rna-XM 043091406.1
Castanea crenata Cas 037380.T1
Quercus variabilis Quvar12aG0076300.1
Lithocarpus polystachyus LPO12G073100.1
Arabidopsis thaliana AT4G22920.1
Arabidopsis thaliana AT4G11910.1
Arabidopsis thaliana AT4G11911.1
Lotus japonicus Lj4g3v1327600.1
Medicago truncatula MtrunA17 Chr5g0398211
Lotus japonicus Lj4g3v1287250.1
Lotus japonicus Lj2g3v2003030.1
Phaseolus vulgaris Phvul.002G153100.1
Glycine max glyma.Wm82.gnm4.ann1.Glyma.11G027400.1
Glycine max glyma.Wm82.gnm4.ann1.Glyma.01G214600.1

OG0005356:SHORT ROOT 1

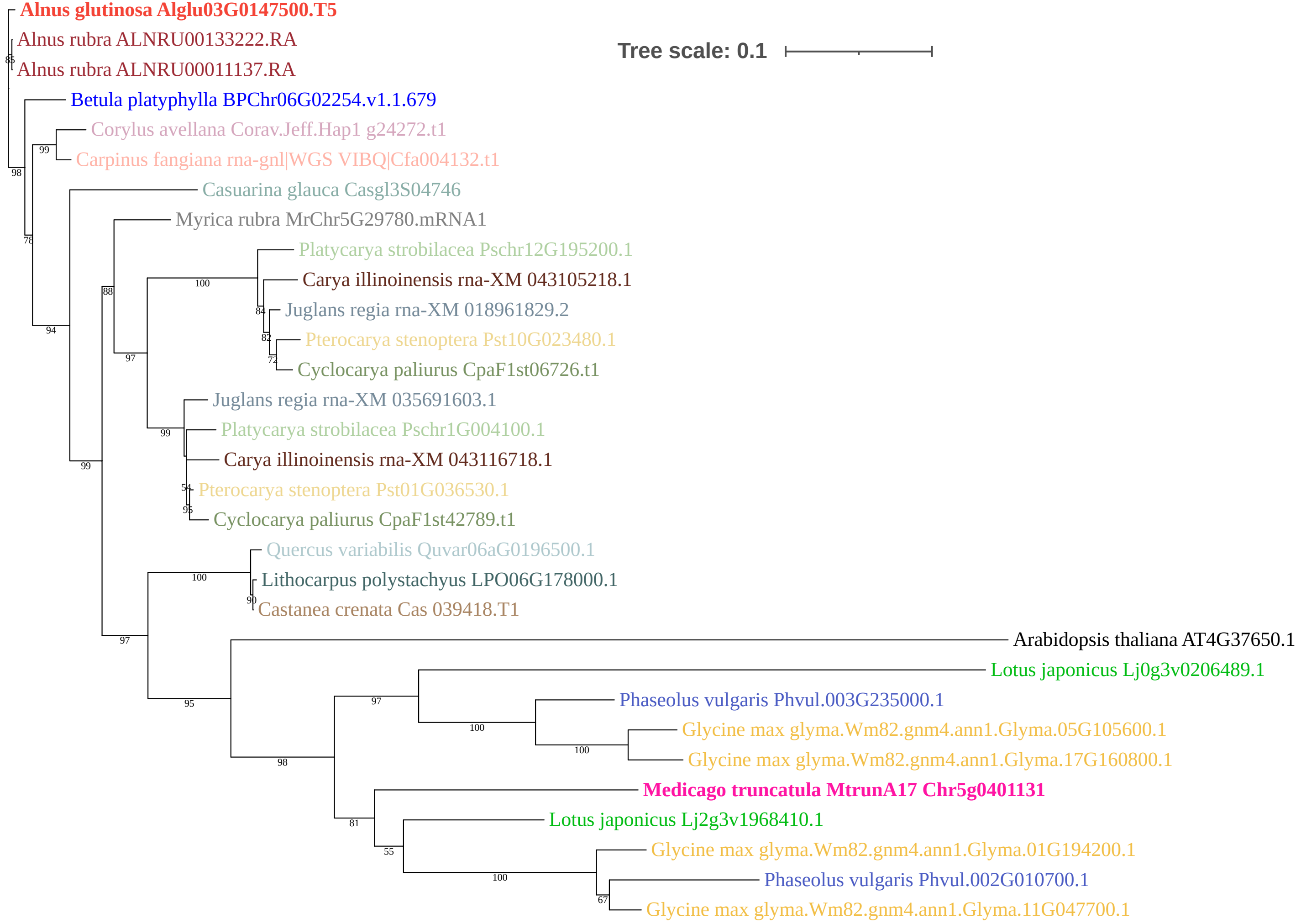

OG0005417:SCARECROW LIKE-13 INVOLVED IN NODULATION 1

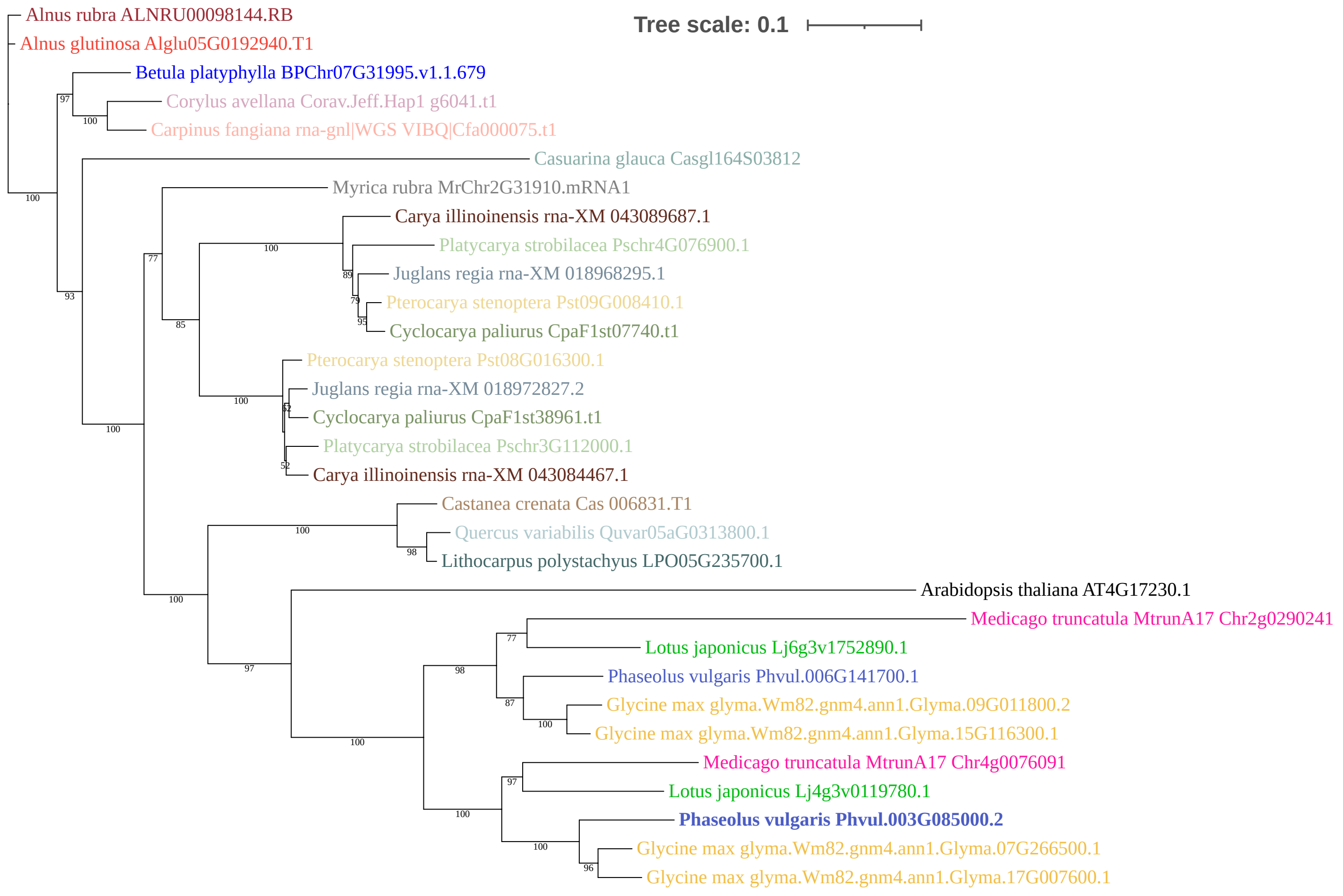

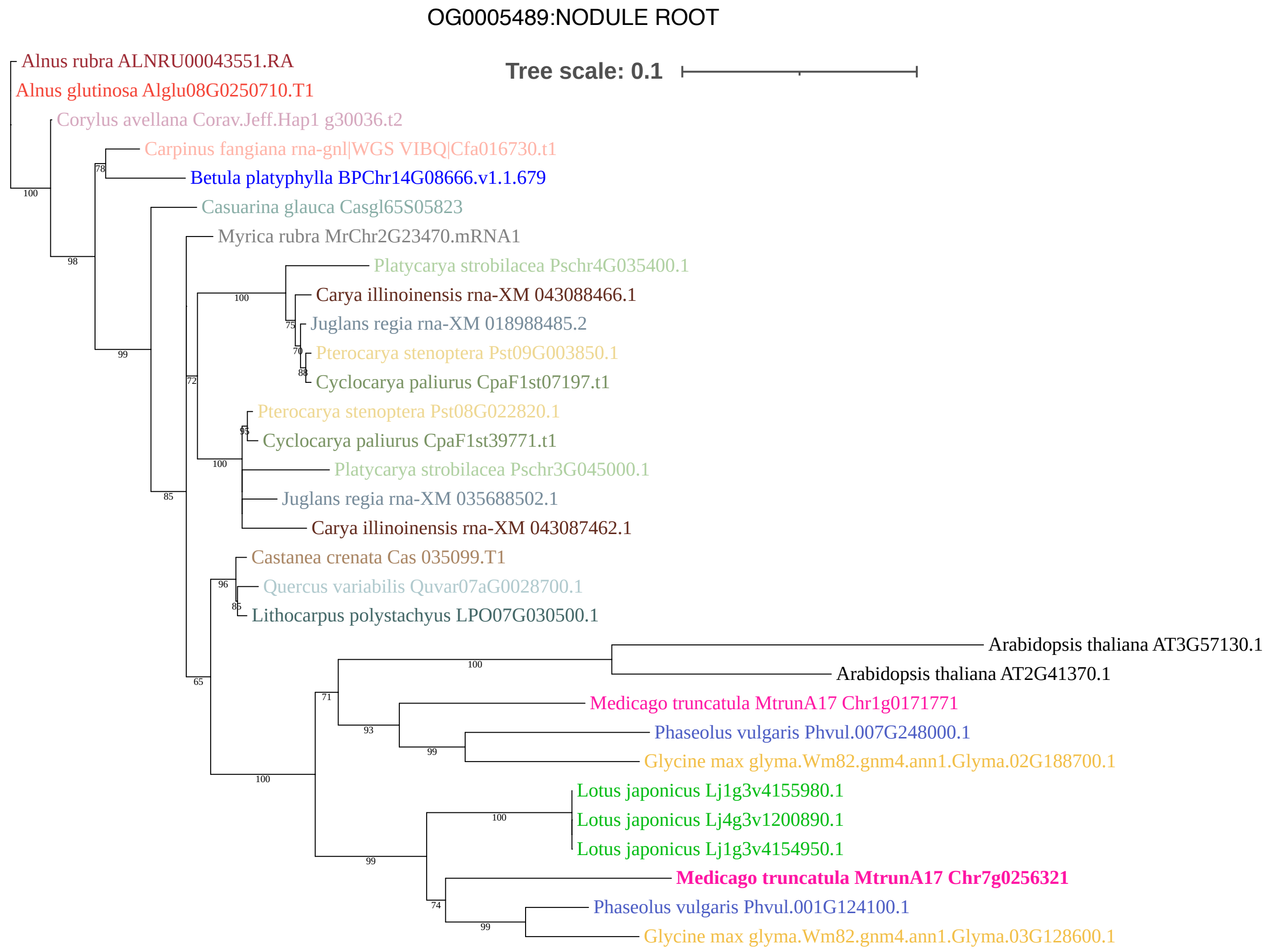
OG0005489:NODULE ROOT
Tree scale: 0.1
Alnus rubra ALNRU00043551.RA
Alnus glutinosa Alglu08G0250710.T1
Corylus avellana Corav.Jeff.Hap1 g30036.t2
Carpinus fangiana rna-gnl|WGS VIBQ|Cfa016730.t1
Betula platyphylla BPChr14G08666.v1.1.679
Casuarina glauca Casgl65S05823
Myrica rubra MrChr2G23470.mRNA1
Platycarya strobilacea Pschr4G035400.1
Carya illinoinensis rna-XM 043088466.1
Juglans regia rna-XM 018988485.2
Pterocarya stenoptera Pst09G003850.1
Cyclocarya paliurus CpaF1st07197.t1
Pterocarya stenoptera Pst08G022820.1
Cyclocarya paliurus CpaF1st39771.t1
Platycarya strobilacea Pschr3G045000.1
Juglans regia rna-XM 035688502.1
Carya illinoinensis rna-XM 043087462.1
Castanea crenata Cas 035099.T1
Quercus variabilis Quvar07aG0028700.1
Lithocarpus polystachyus LPO07G030500.1
Arabidopsis thaliana AT3G57130.1
Arabidopsis thaliana AT2G41370.1
Medicago truncatula MtrunA17 Chr1g0171771
Phaseolus vulgaris Phvul.007G248000.1
Glycine max glyma.Wm82.gnm4.ann1.Glyma.02G188700.1
Lotus japonicus Lj1g3v4155980.1
Lotus japonicus Lj4g3v1200890.1
Lotus japonicus Lj1g3v4154950.1
Medicago truncatula MtrunA17 Chr7g0256321
Phaseolus vulgaris Phvul.001G124100.1
Glycine max glyma.Wm82.gnm4.ann1.Glyma.03G128600.1
100
98
78
99
100
75
70
88
72
95
100
85
96
85
65
100
71
93
99
100
100
99
74
99

OG0005516:PARTNER OF NOB1-LIKE

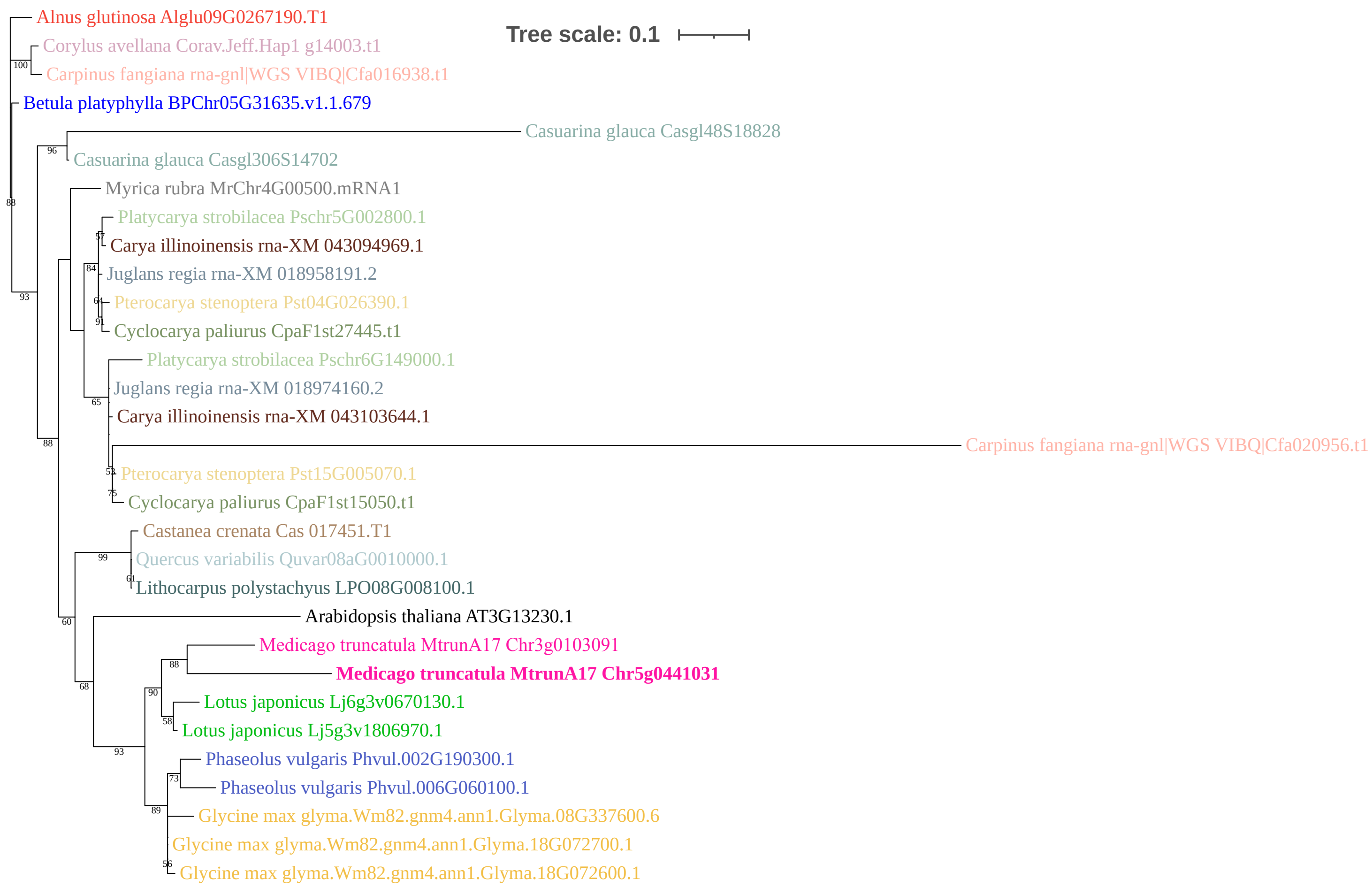

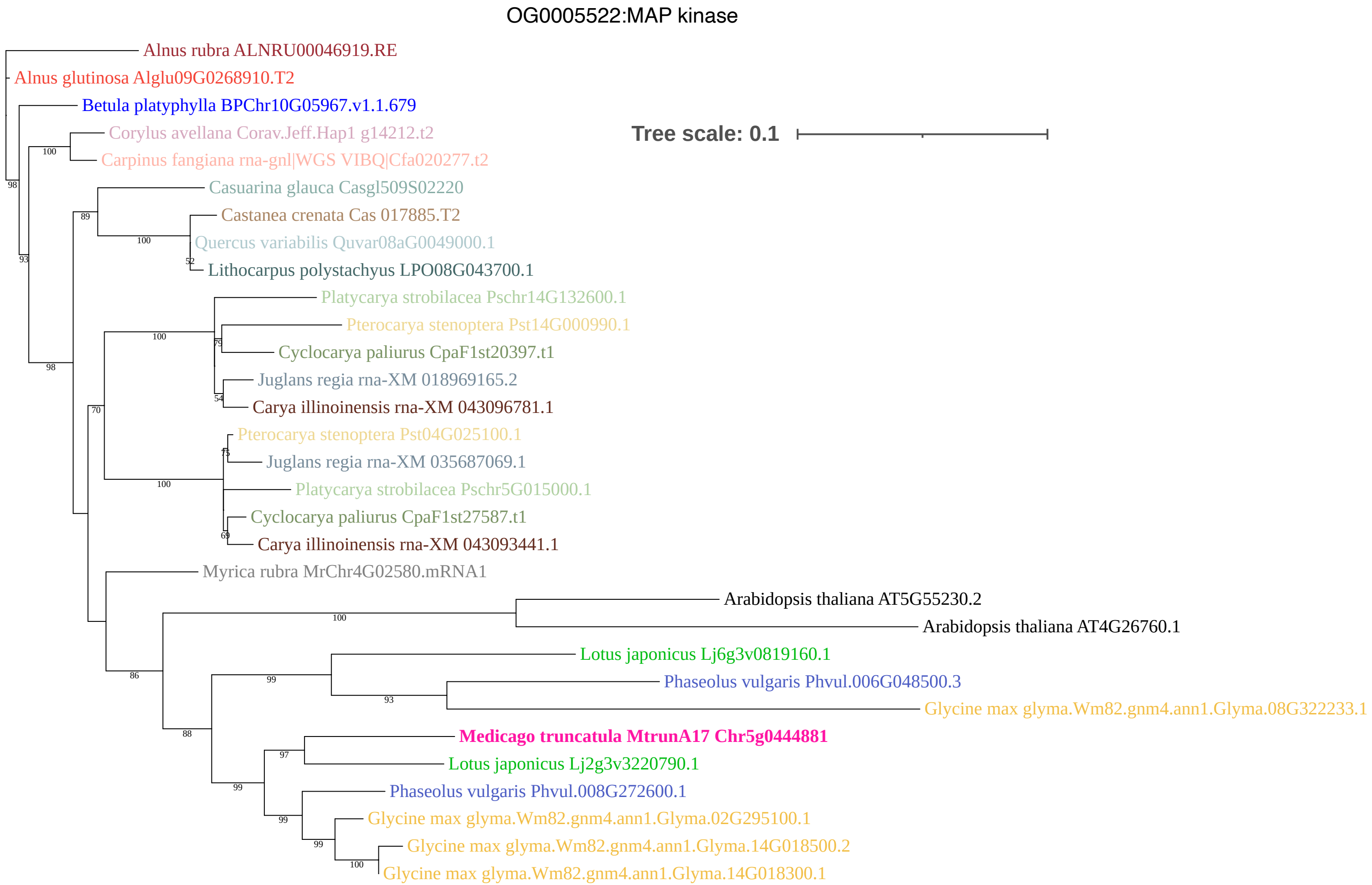
OG0005522:MAP kinase
Tree scale: 0.1
Alnus rubra ALNRU00046919.RE
Alnus glutinosa Alglu09G0268910.T2
Betula platyphylla BPChr10G05967.v1.1.679
Corylus avellana Corav.Jeff.Hap1 g14212.t2
Carpinus fangiana rna-gnl|WGS VIBQ|Cfa020277.t2
Casuarina glauca Casgl509S02220
Castanea crenata Cas 017885.T2
Quercus variabilis Quvar08aG0049000.1
Lithocarpus polystachyus LPO08G043700.1
Platycarya strobilacea Pschr14G132600.1
Pterocarya stenoptera Pst14G000990.1
Cyclocarya paliurus CpaF1st20397.t1
Juglans regia rna-XM 018969165.2
Carya illinoinensis rna-XM 043096781.1
Pterocarya stenoptera Pst04G025100.1
Juglans regia rna-XM 035687069.1
Platycarya strobilacea Pschr5G015000.1
Cyclocarya paliurus CpaF1st27587.t1
Carya illinoinensis rna-XM 043093441.1
Myrica rubra MrChr4G02580.mRNA1
Arabidopsis thaliana AT5G55230.2
Arabidopsis thaliana AT4G26760.1
Lotus japonicus Lj6g3v0819160.1
Phaseolus vulgaris Phvul.006G048500.3
Glycine max glyma.Wm82.gnm4.ann1.Glyma.08G322233.1
Medicago truncatula MtrunA17 Chr5g0444881
Lotus japonicus Lj2g3v3220790.1
Phaseolus vulgaris Phvul.008G272600.1
Glycine max glyma.Wm82.gnm4.ann1.Glyma.02G295100.1
Glycine max glyma.Wm82.gnm4.ann1.Glyma.14G018500.2
Glycine max glyma.Wm82.gnm4.ann1.Glyma.14G018300.1

OG0005631:NAM/ATAF/CUC-encoding 969

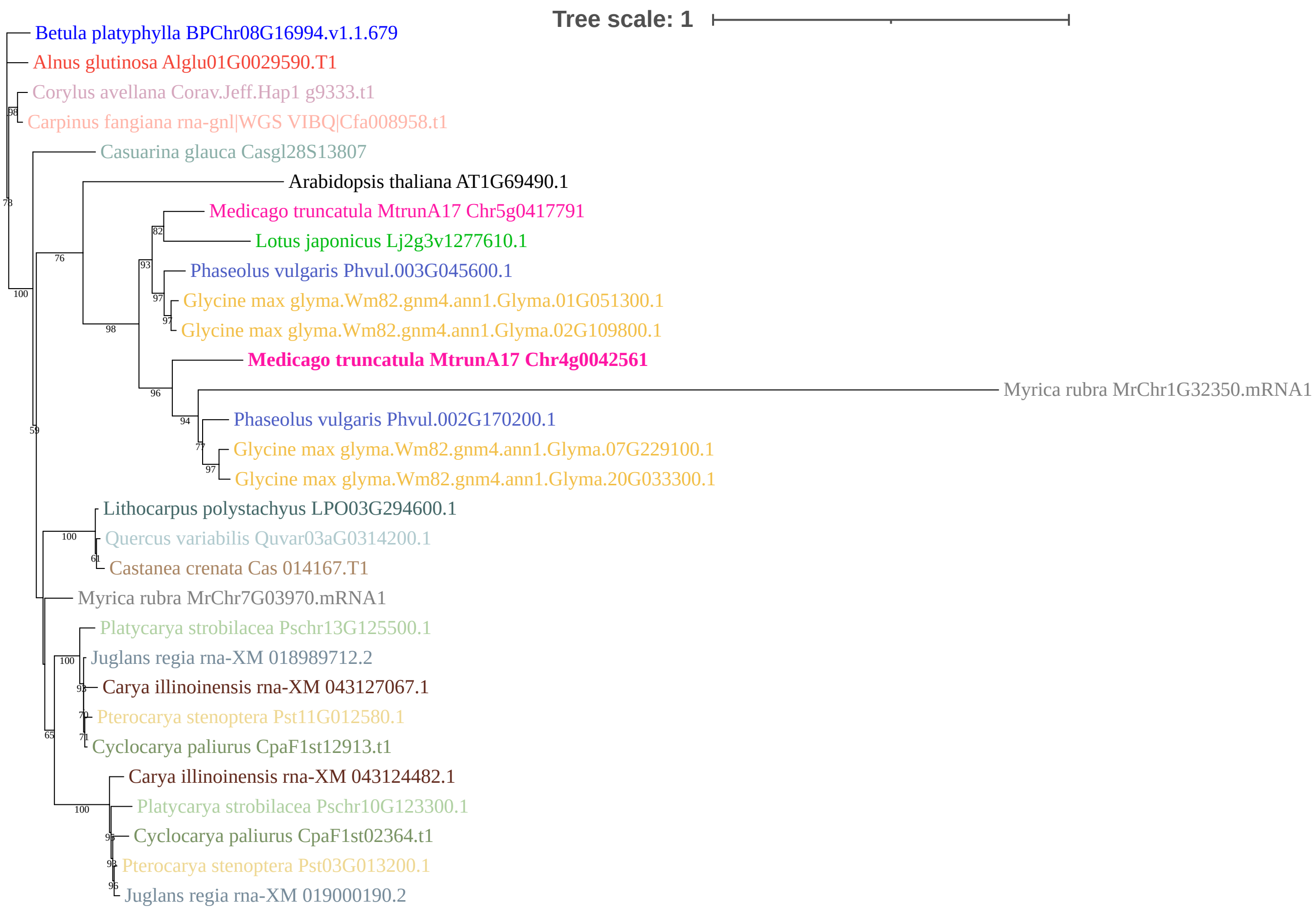

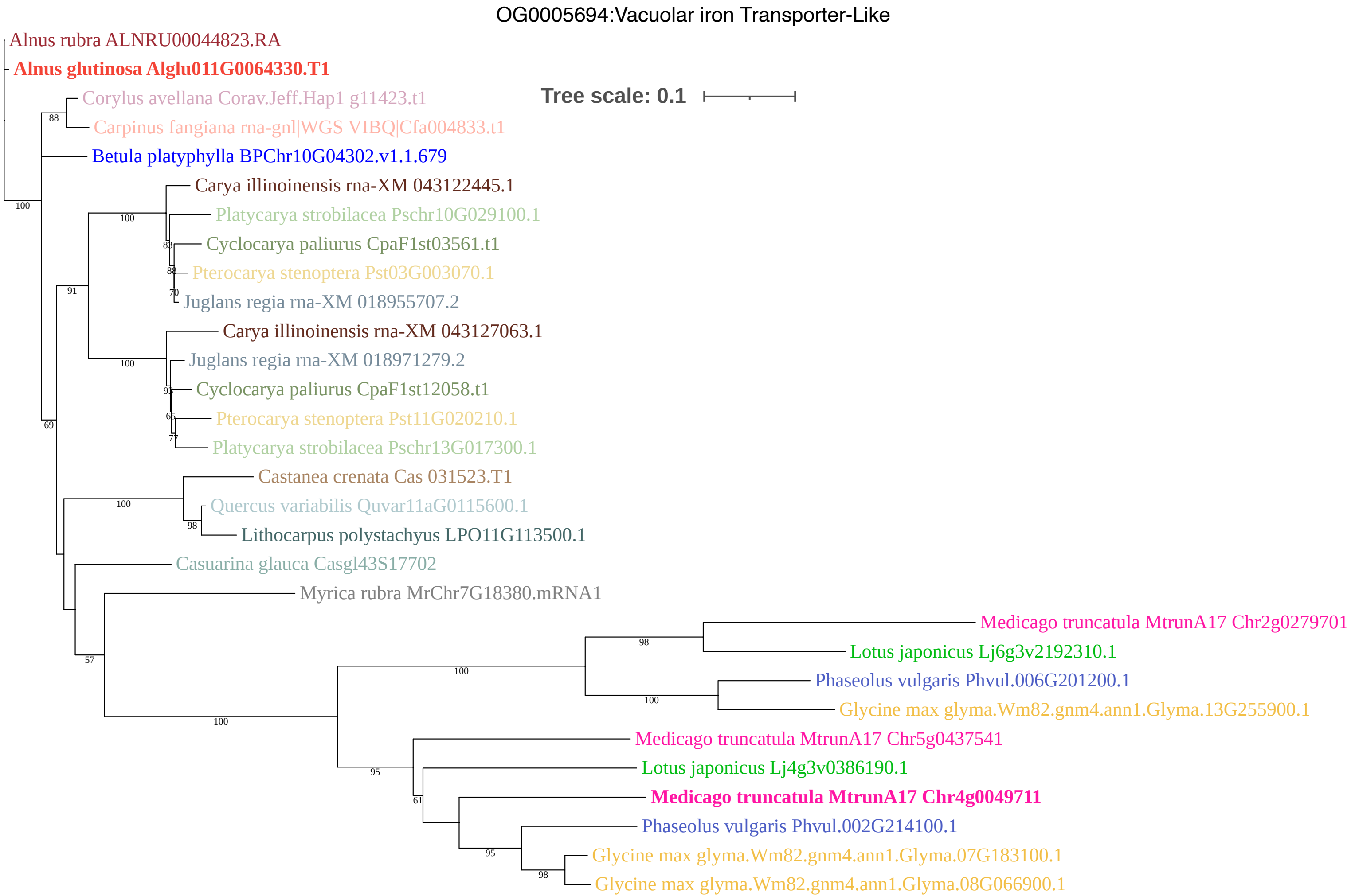
OG0005694:Vacuolar iron Transporter-Like
Tree scale: 0.1
Alnus rubra ALNRU00044823.RA
Alnus glutinosa Alglu011G0064330.T1
Corylus avellana Corav.Jeff.Hap1 g11423.t1
Carpinus fangiana rna-gnl|WGS VIBQ|Cfa004833.t1
Betula platyphylla BPChr10G04302.v1.1.679
Carya illinoinensis rna-XM 043122445.1
Platycarya strobilacea Pschr10G029100.1
Cyclocarya paliurus CpaF1st03561.t1
Pterocarya stenoptera Pst03G003070.1
Juglans regia rna-XM 018955707.2
Carya illinoinensis rna-XM 043127063.1
Juglans regia rna-XM 018971279.2
Cyclocarya paliurus CpaF1st12058.t1
Pterocarya stenoptera Pst11G020210.1
Platycarya strobilacea Pschr13G017300.1
Castanea crenata Cas 031523.T1
Quercus variabilis Quvar11aG0115600.1
Lithocarpus polystachyus LPO11G113500.1
Casuarina glauca Casgl43S17702
Myrica rubra MrChr7G18380.mRNA1
Medicago truncatula MtrunA17 Chr2g0279701
Lotus japonicus Lj6g3v2192310.1
Phaseolus vulgaris Phvul.006G201200.1
Glycine max glyma.Wm82.gnm4.ann1.Glyma.13G255900.1
Medicago truncatula MtrunA17 Chr5g0437541
Lotus japonicus Lj4g3v0386190.1
Medicago truncatula MtrunA17 Chr4g0049711
Phaseolus vulgaris Phvul.002G214100.1
Glycine max glyma.Wm82.gnm4.ann1.Glyma.07G183100.1
Glycine max glyma.Wm82.gnm4.ann1.Glyma.08G066900.1

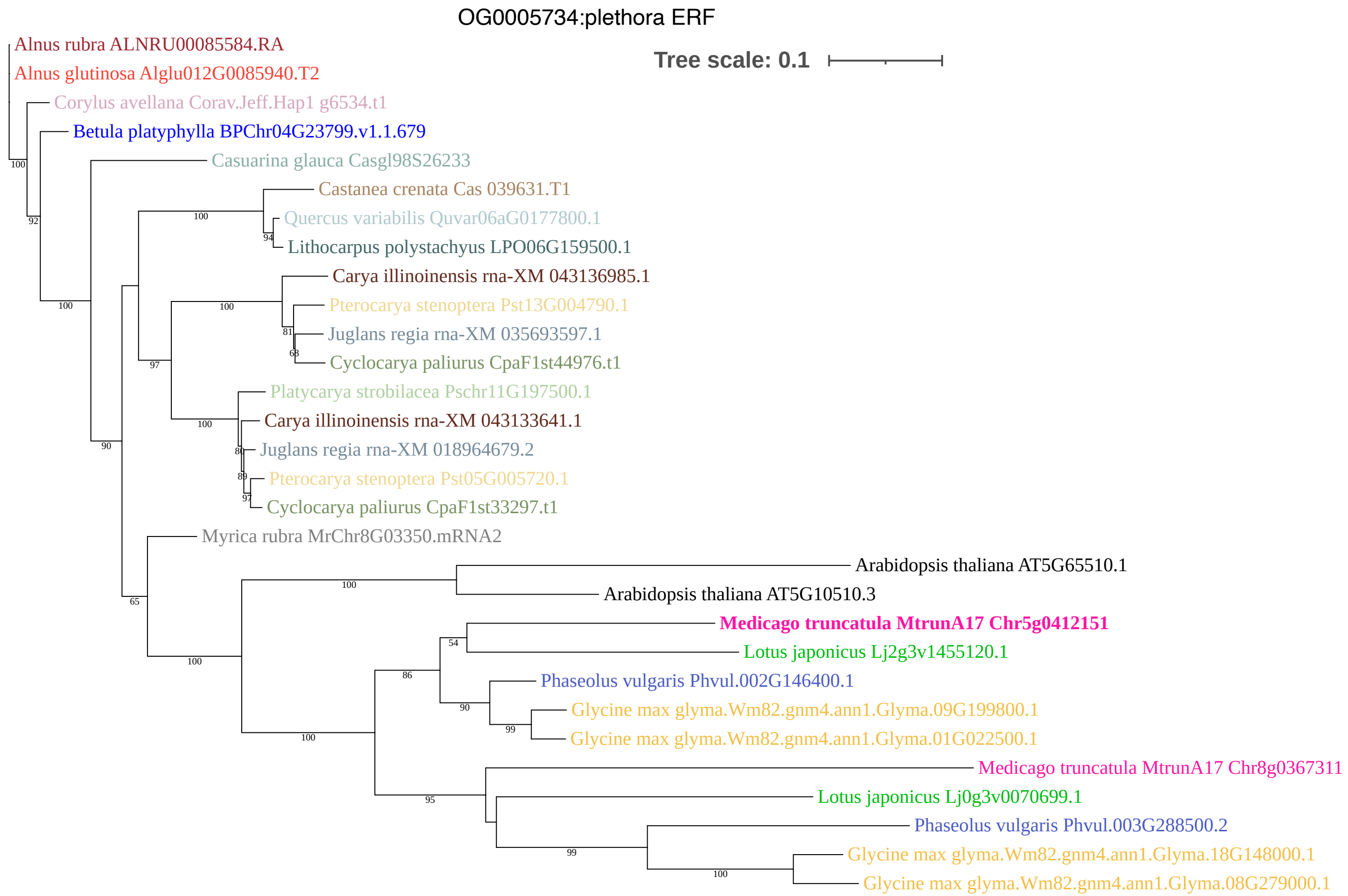
OG0005734:plethora ERF
Tree scale: 0.1
Alnus rubra ALNRU00085584.RA
Alnus glutinosa Alglu012G0085940.T2
Corylus avellana Corav.Jeff.Hap1 g6534.t1
Betula platyphylla BPChr04G23799.v1.1.679
Casuarina glauca Casgl98S26233
Castanea crenata Cas 039631.T1
Quercus variabilis Quvar06aG0177800.1
Lithocarpus polystachyus LPO06G159500.1
Carya illinoinensis rna-XM 043136985.1
Pterocarya stenoptera Pst13G004790.1
Juglans regia rna-XM 035693597.1
Cyclocarya paliurus CpaF1st44976.t1
Platycarya strobilacea Pschr11G197500.1
Carya illinoinensis rna-XM 043133641.1
Juglans regia rna-XM 018964679.2
Pterocarya stenoptera Pst05G005720.1
Cyclocarya paliurus CpaF1st33297.t1
Myrica rubra MrChr8G03350.mRNA2
Arabidopsis thaliana AT5G65510.1
Arabidopsis thaliana AT5G10510.3
Medicago truncatula MtrunA17 Chr5g0412151
Lotus japonicus Lj2g3v1455120.1
Phaseolus vulgaris Phvul.002G146400.1
Glycine max glyma.Wm82.gnm4.ann1.Glyma.09G199800.1
Glycine max glyma.Wm82.gnm4.ann1.Glyma.01G022500.1
Medicago truncatula MtrunA17 Chr8g0367311
Lotus japonicus Lj0g3v0070699.1
Phaseolus vulgaris Phvul.003G288500.2
Glycine max glyma.Wm82.gnm4.ann1.Glyma.18G148000.1
Glycine max glyma.Wm82.gnm4.ann1.Glyma.08G279000.1

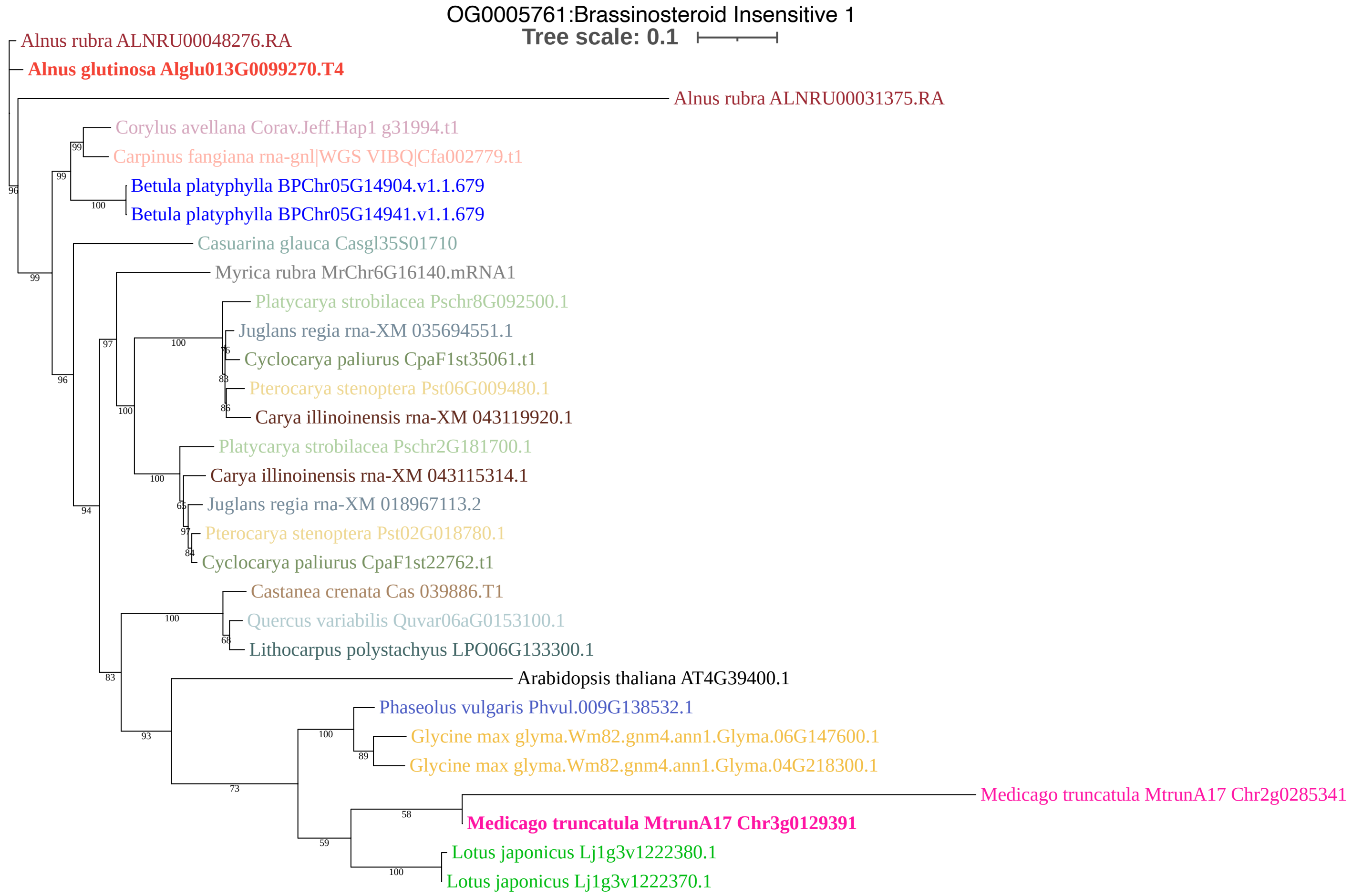
OG0005761:Brassinosteroid Insensitive 1
Tree scale: 0.1
Alnus rubra ALNRU00048276.RA
Alnus glutinosa Alglu013G0099270.T4
Alnus rubra ALNRU00031375.RA
Corylus avellana Corav.Jeff.Hap1 g31994.t1
Carpinus fangiana rna-gnl|WGS VIBQ|Cfa002779.t1
Betula platyphylla BPChr05G14904.v1.1.679
Betula platyphylla BPChr05G14941.v1.1.679
Casuarina glauca Casgl35S01710
Myrica rubra MrChr6G16140.mRNA1
Platycarya strobilacea Pschr8G092500.1
Juglans regia rna-XM 035694551.1
Cyclocarya paliurus CpaF1st35061.t1
Pterocarya stenoptera Pst06G009480.1
Carya illinoinensis rna-XM 043119920.1
Platycarya strobilacea Pschr2G181700.1
Carya illinoinensis rna-XM 043115314.1
Juglans regia rna-XM 018967113.2
Pterocarya stenoptera Pst02G018780.1
Cyclocarya paliurus CpaF1st22762.t1
Castanea crenata Cas 039886.T1
Quercus variabilis Quvar06aG0153100.1
Lithocarpus polystachyus LPO06G133300.1
Arabidopsis thaliana AT4G39400.1
Phaseolus vulgaris Phvul.009G138532.1
Glycine max glyma.Wm82.gnm4.ann1.Glyma.06G147600.1
Glycine max glyma.Wm82.gnm4.ann1.Glyma.04G218300.1
Medicago truncatula MtrunA17 Chr2g0285341
Medicago truncatula MtrunA17 Chr3g0129391
Lotus japonicus Lj1g3v1222380.1
Lotus japonicus Lj1g3v1222370.1

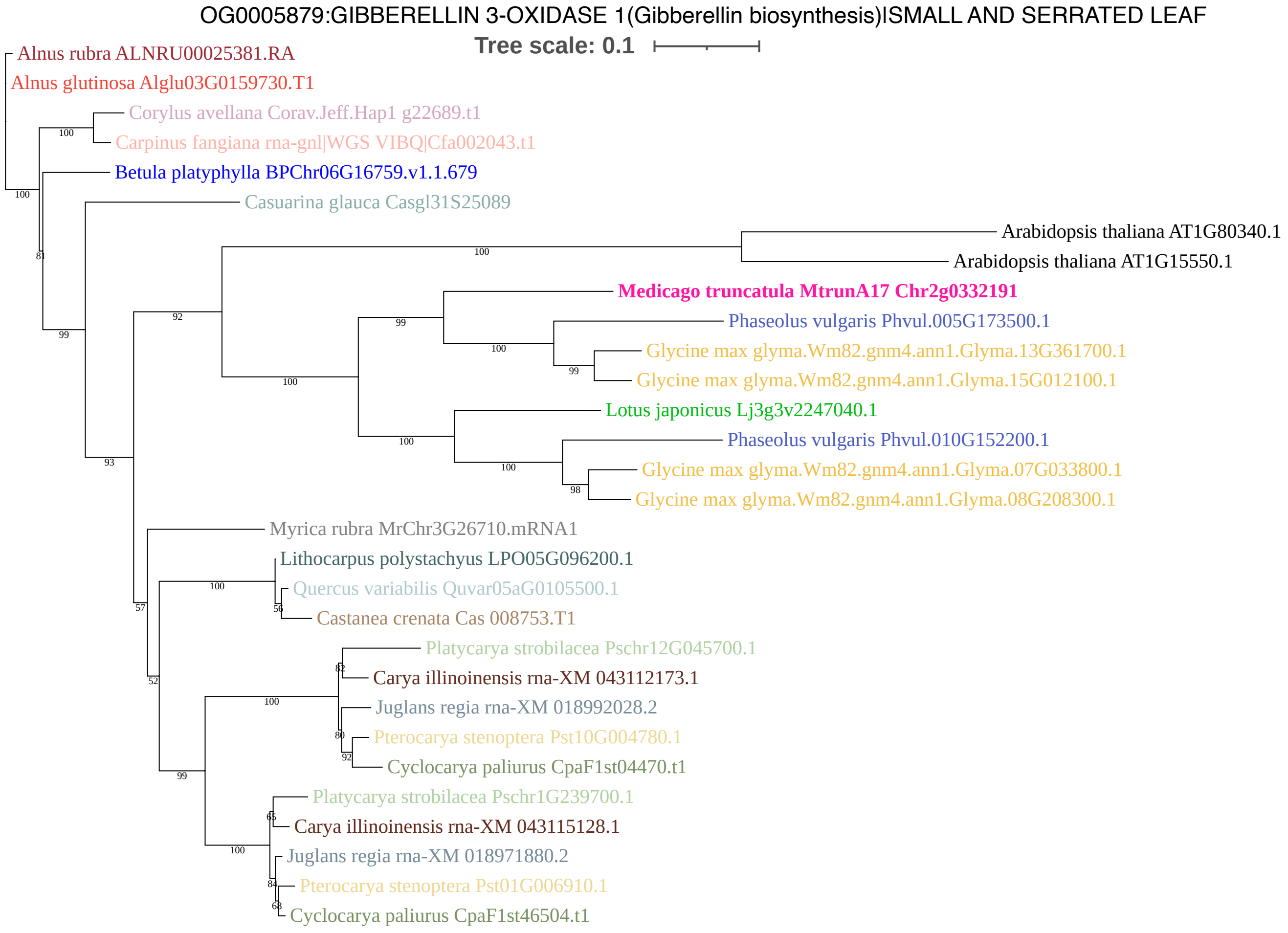
OG0005879:GIBBERELLIN 3-OXIDASE 1(Gibberellin biosynthesis)|SMALL AND SERRATED LEAF
Tree scale: 0.1
Alnus rubra ALNRU00025381.RA
Alnus glutinosa Alglu03G0159730.T1
Corylus avellana Corav.Jeff.Hap1 g22689.t1
Carpinus fangiana rna-gnl|WGS VIBQ|Cfa002043.t1
Betula platyphylla BPChr06G16759.v1.1.679
Casuarina glauca Casgl31S25089
Arabidopsis thaliana AT1G80340.1
Arabidopsis thaliana AT1G15550.1
Medicago truncatula MtrunA17 Chr2g0332191
Phaseolus vulgaris Phvul.005G173500.1
Glycine max glyma.Wm82.gnm4.ann1.Glyma.13G361700.1
Glycine max glyma.Wm82.gnm4.ann1.Glyma.15G012100.1
Lotus japonicus Lj3g3v2247040.1
Phaseolus vulgaris Phvul.010G152200.1
Glycine max glyma.Wm82.gnm4.ann1.Glyma.07G033800.1
Glycine max glyma.Wm82.gnm4.ann1.Glyma.08G208300.1
Myrica rubra MrChr3G26710.mRNA1
Lithocarpus polystachyus LPO05G096200.1
Quercus variabilis Quvar05aG0105500.1
Castanea crenata Cas 008753.T1
Platycarya strobilacea Pschr12G045700.1
Carya illinoinensis rna-XM 043112173.1
Juglans regia rna-XM 018992028.2
Pterocarya stenoptera Pst10G004780.1
Cyclocarya paliurus CpaF1st04470.t1
Platycarya strobilacea Pschr1G239700.1
Carya illinoinensis rna-XM 043115128.1
Juglans regia rna-XM 018971880.2
Pterocarya stenoptera Pst01G006910.1
Cyclocarya paliurus CpaF1st46504.t1

OG0005894:Golven/ Root Growth Factor|Root Meristem Growth Factor(peptide)

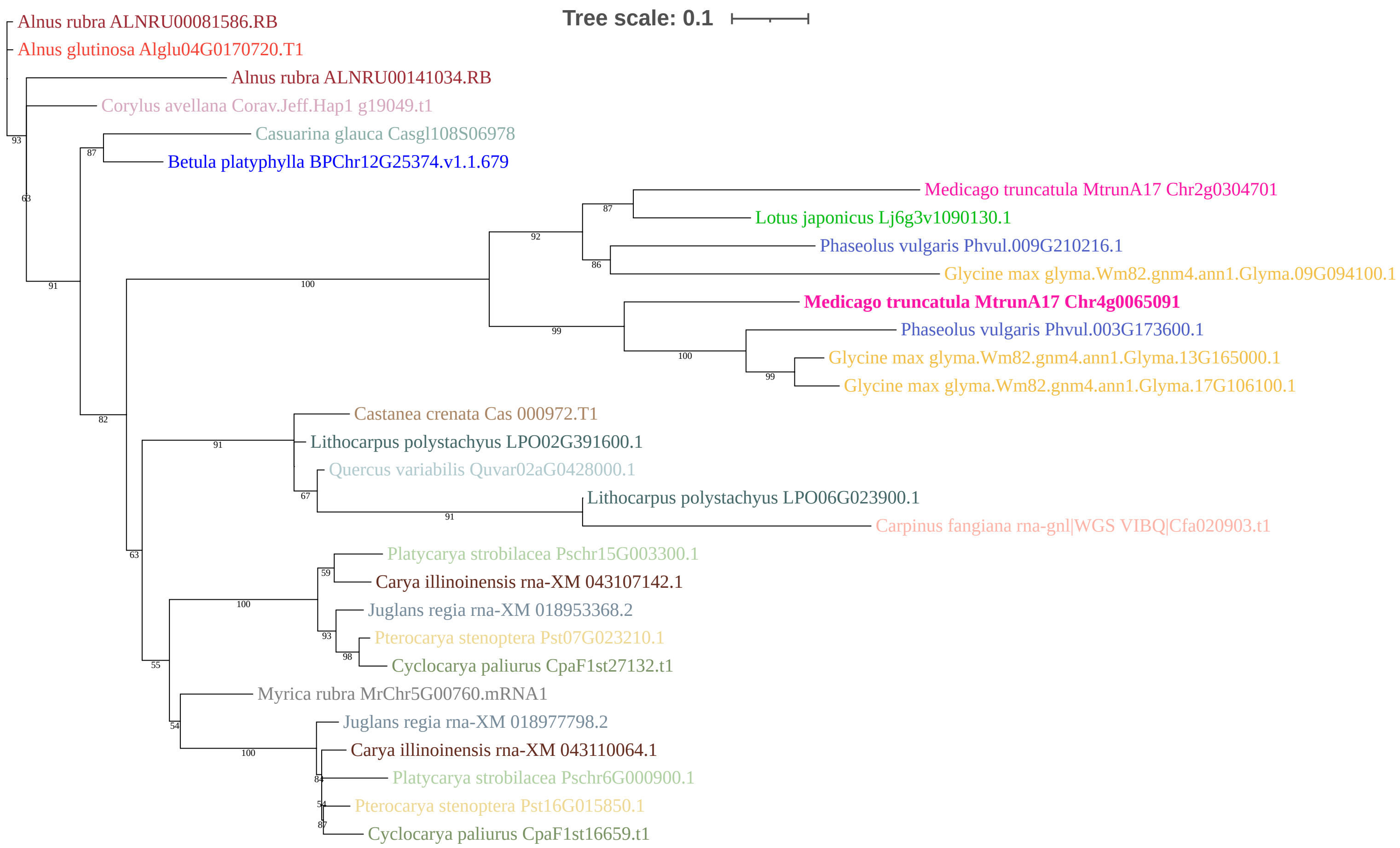

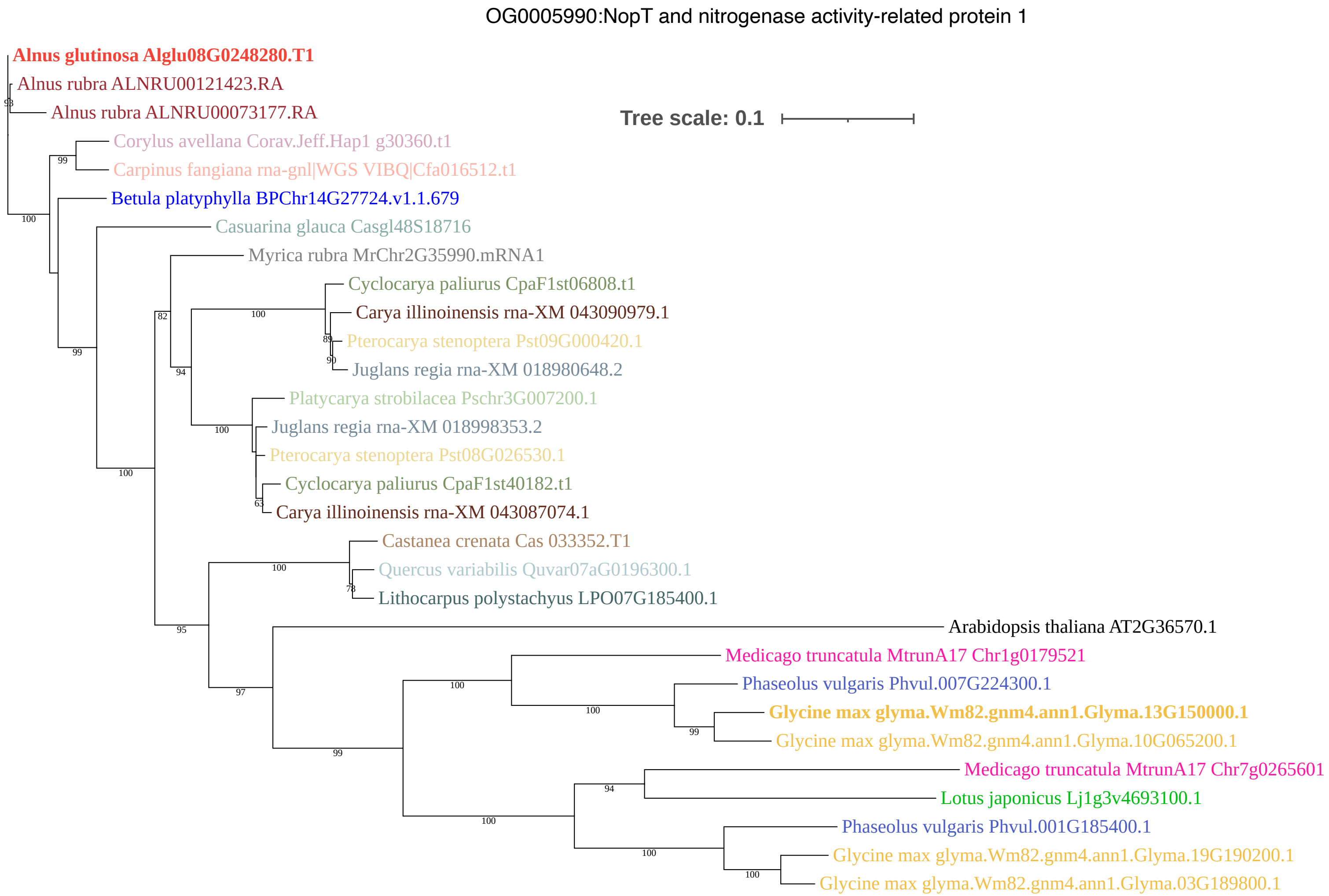
OG0005990:NopT and nitrogenase activity-related protein 1
Tree scale: 0.1
Alnus glutinosa Alglu08G0248280.T1
Alnus rubra ALNRU00121423.RA
Alnus rubra ALNRU00073177.RA
Corylus avellana Corav.Jeff.Hap1 g30360.t1
Carpinus fangiana rna-gnl|WGS VIBQ|Cfa016512.t1
Betula platyphylla BPChr14G27724.v1.1.679
Casuarina glauca Casgl48S18716
Myrica rubra MrChr2G35990.mRNA1
Cyclocarya paliurus CpaF1st06808.t1
Carya illinoinensis rna-XM 043090979.1
Pterocarya stenoptera Pst09G000420.1
Juglans regia rna-XM 018980648.2
Platycarya strobilacea Pschr3G007200.1
Juglans regia rna-XM 018998353.2
Pterocarya stenoptera Pst08G026530.1
Cyclocarya paliurus CpaF1st40182.t1
Carya illinoinensis rna-XM 043087074.1
Castanea crenata Cas 033352.T1
Quercus variabilis Quvar07aG0196300.1
Lithocarpus polystachyus LPO07G185400.1
Arabidopsis thaliana AT2G36570.1
Medicago truncatula MtrunA17 Chr1g0179521
Phaseolus vulgaris Phvul.007G224300.1
Glycine max glyma.Wm82.gnm4.ann1.Glyma.13G150000.1
Glycine max glyma.Wm82.gnm4.ann1.Glyma.10G065200.1
Medicago truncatula MtrunA17 Chr7g0265601
Lotus japonicus Lj1g3v4693100.1
Phaseolus vulgaris Phvul.001G185400.1
Glycine max glyma.Wm82.gnm4.ann1.Glyma.19G190200.1
Glycine max glyma.Wm82.gnm4.ann1.Glyma.03G189800.1

OG0006098:nodulin 22 in common bean

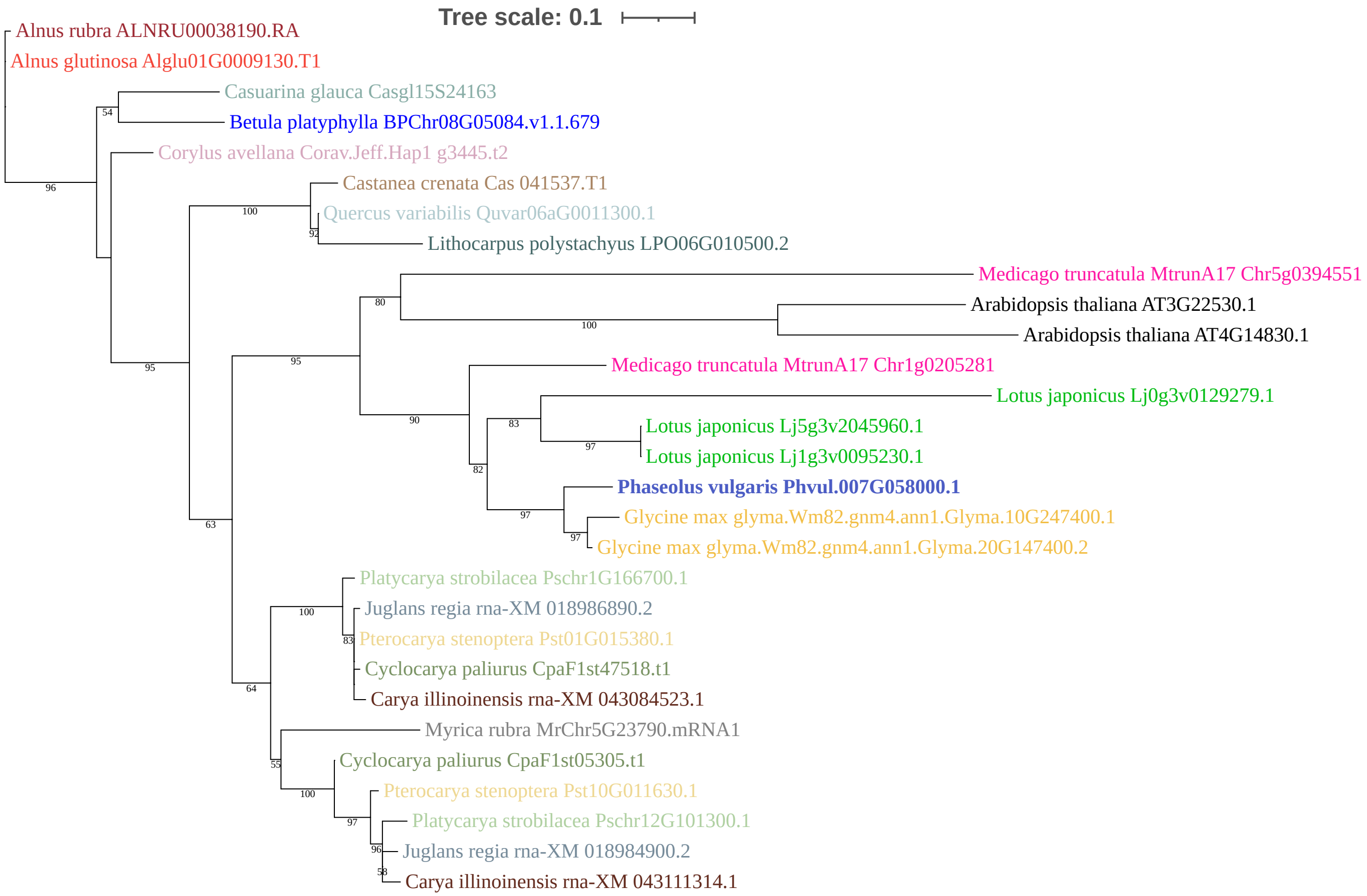

OG0006119:CYTOKININ RESPONSE 1

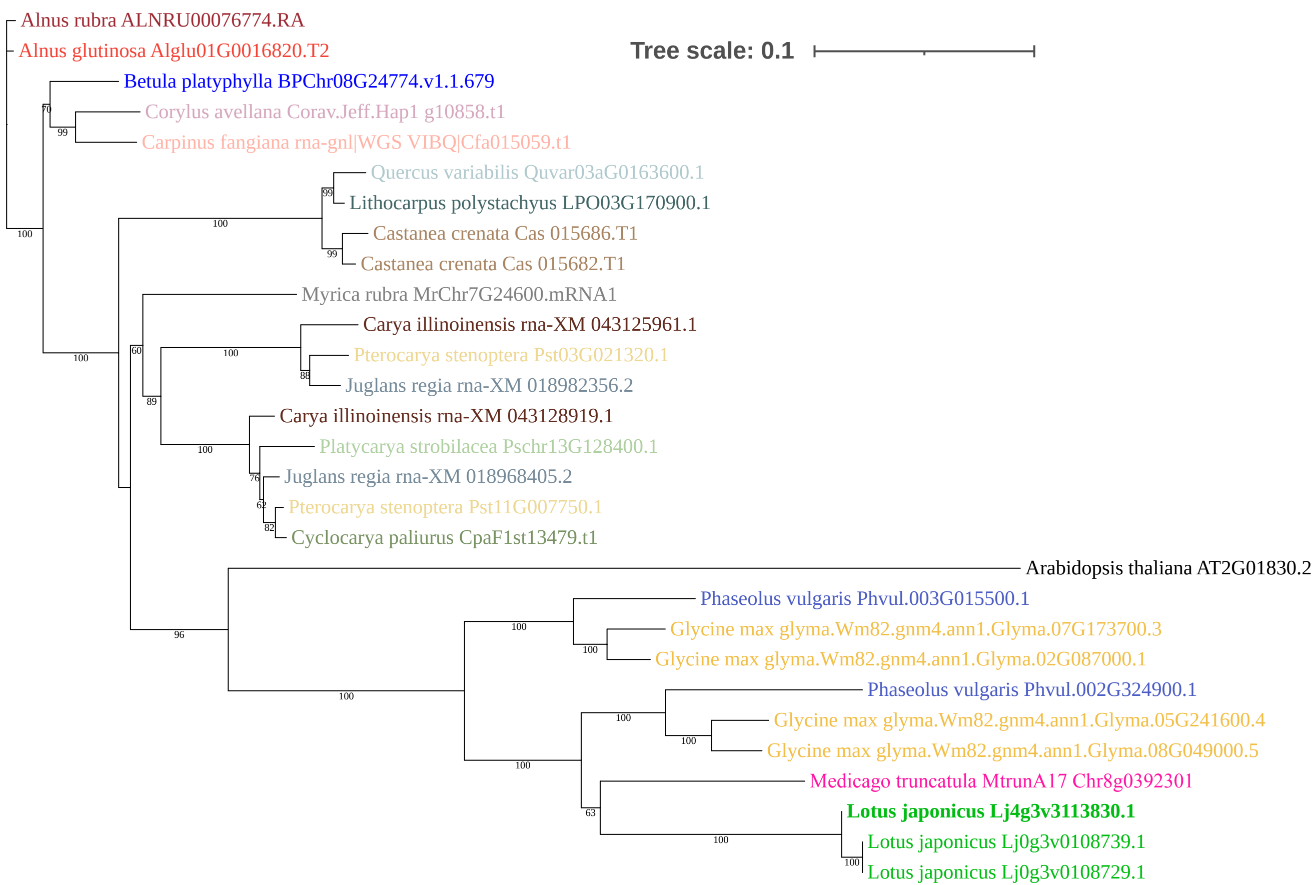

OG0006190:Soybean heterotrimeric G PROTEINS

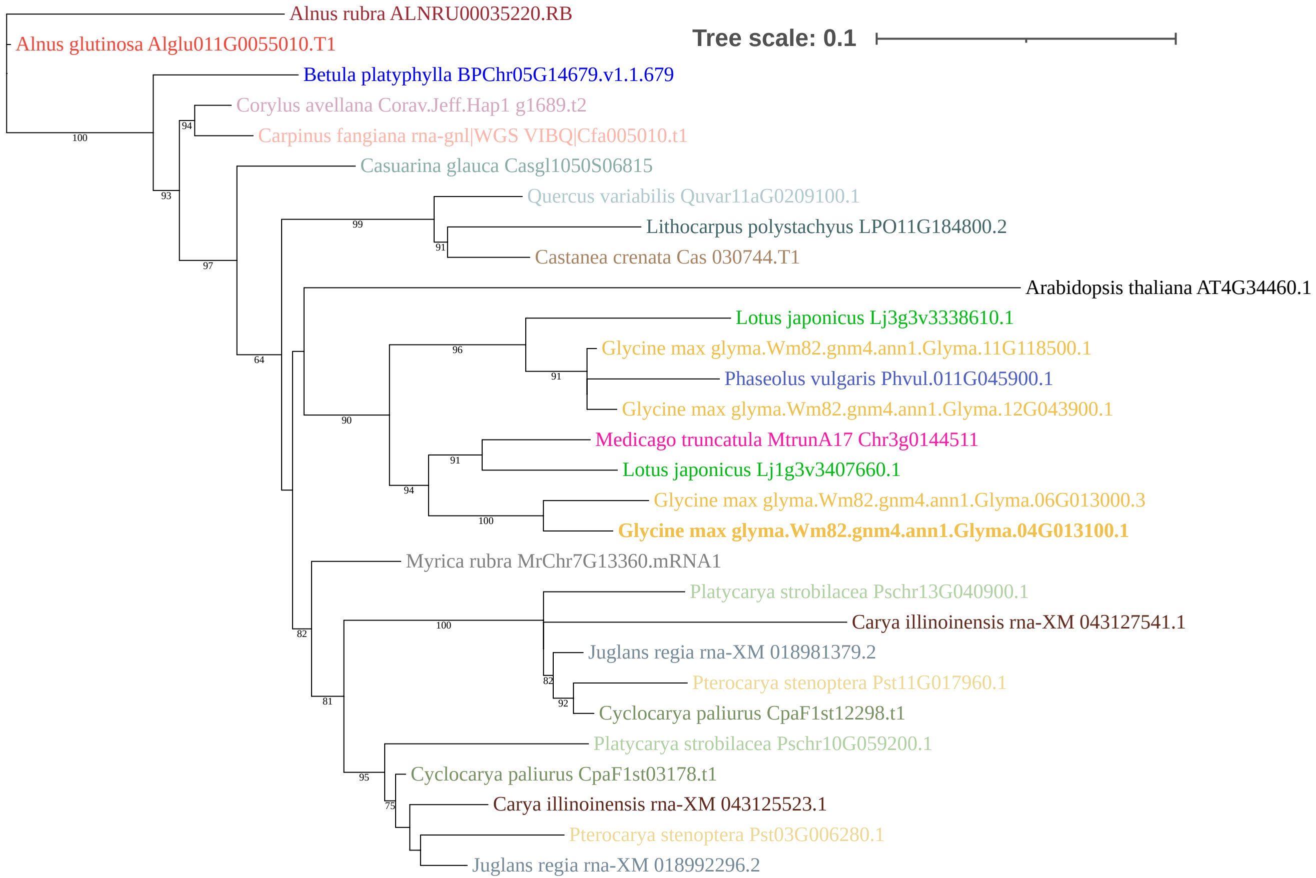

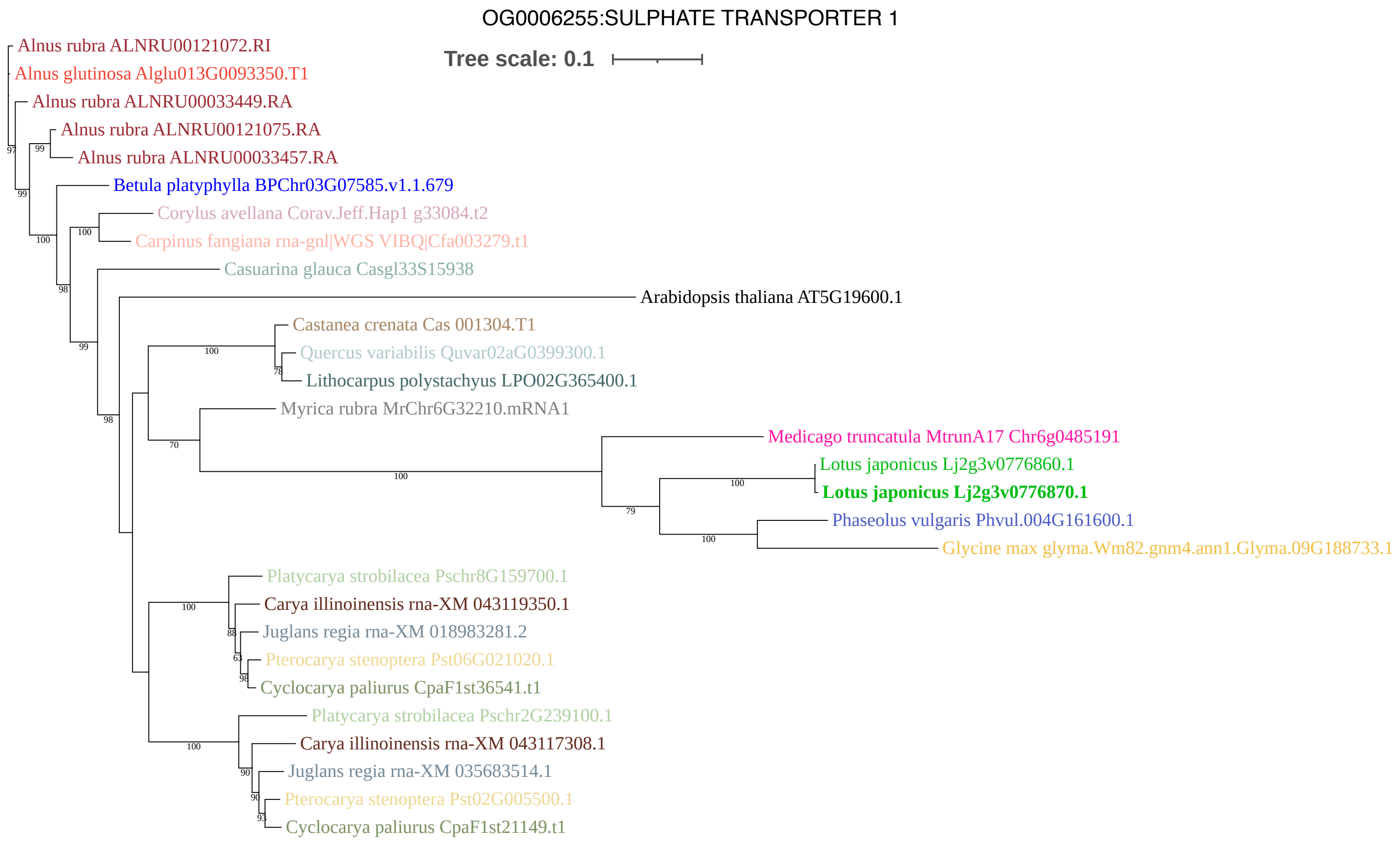

OG0006255:SULPHATE TRANSPORTER 1
Tree scale: 0.1
Alnus rubra ALNRU00121072.RI
Alnus glutinosa Alglu013G0093350.T1
Alnus rubra ALNRU00033449.RA
Alnus rubra ALNRU00121075.RA
Alnus rubra ALNRU00033457.RA
Betula platyphylla BPChr03G07585.v1.1.679
Corylus avellana Corav.Jeff.Hap1 g33084.t2
Carpinus fangiana rna-gnl|WGS VIBQ|Cfa003279.t1
Casuarina glauca Casgl33S15938
Arabidopsis thaliana AT5G19600.1
Castanea crenata Cas 001304.T1
Quercus variabilis Quvar02aG0399300.1
Lithocarpus polystachyus LPO02G365400.1
Myrica rubra MrChr6G32210.mRNA1
Medicago truncatula MtrunA17 Chr6g0485191
Lotus japonicus Lj2g3v0776860.1
Lotus japonicus Lj2g3v0776870.1
Phaseolus vulgaris Phvul.004G161600.1
Glycine max glyma.Wm82.gnm4.ann1.Glyma.09G188733.1
Platycarya strobilacea Pschr8G159700.1
Carya illinoinensis rna-XM 043119350.1
Juglans regia rna-XM 018983281.2
Pterocarya stenoptera Pst06G021020.1
Cyclocarya paliurus CpaF1st36541.t1
Platycarya strobilacea Pschr2G239100.1
Carya illinoinensis rna-XM 043117308.1
Juglans regia rna-XM 035683514.1
Pterocarya stenoptera Pst02G005500.1
Cyclocarya paliurus CpaF1st21149.t1

OG0006281:LOB Domain protein 16

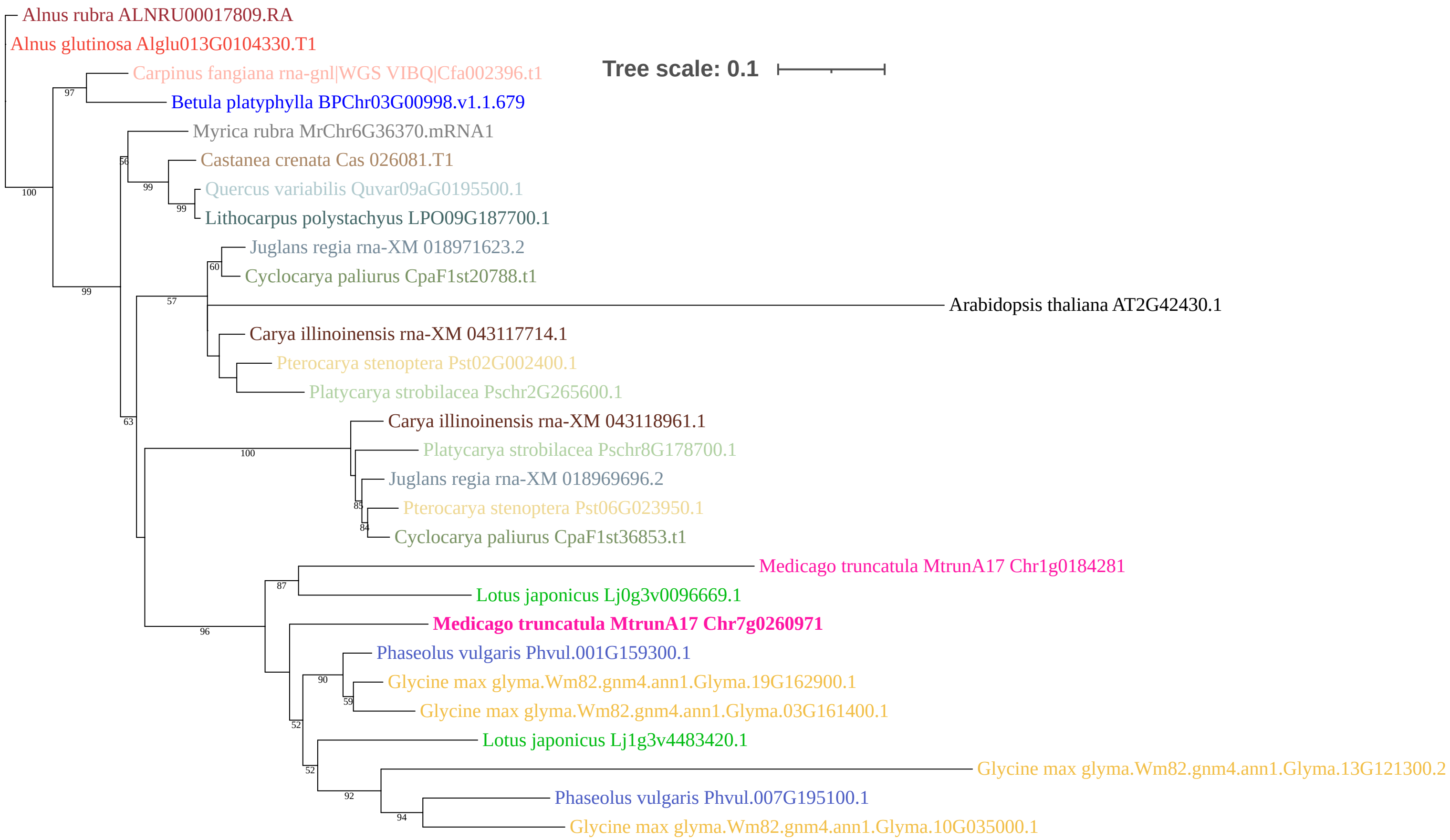

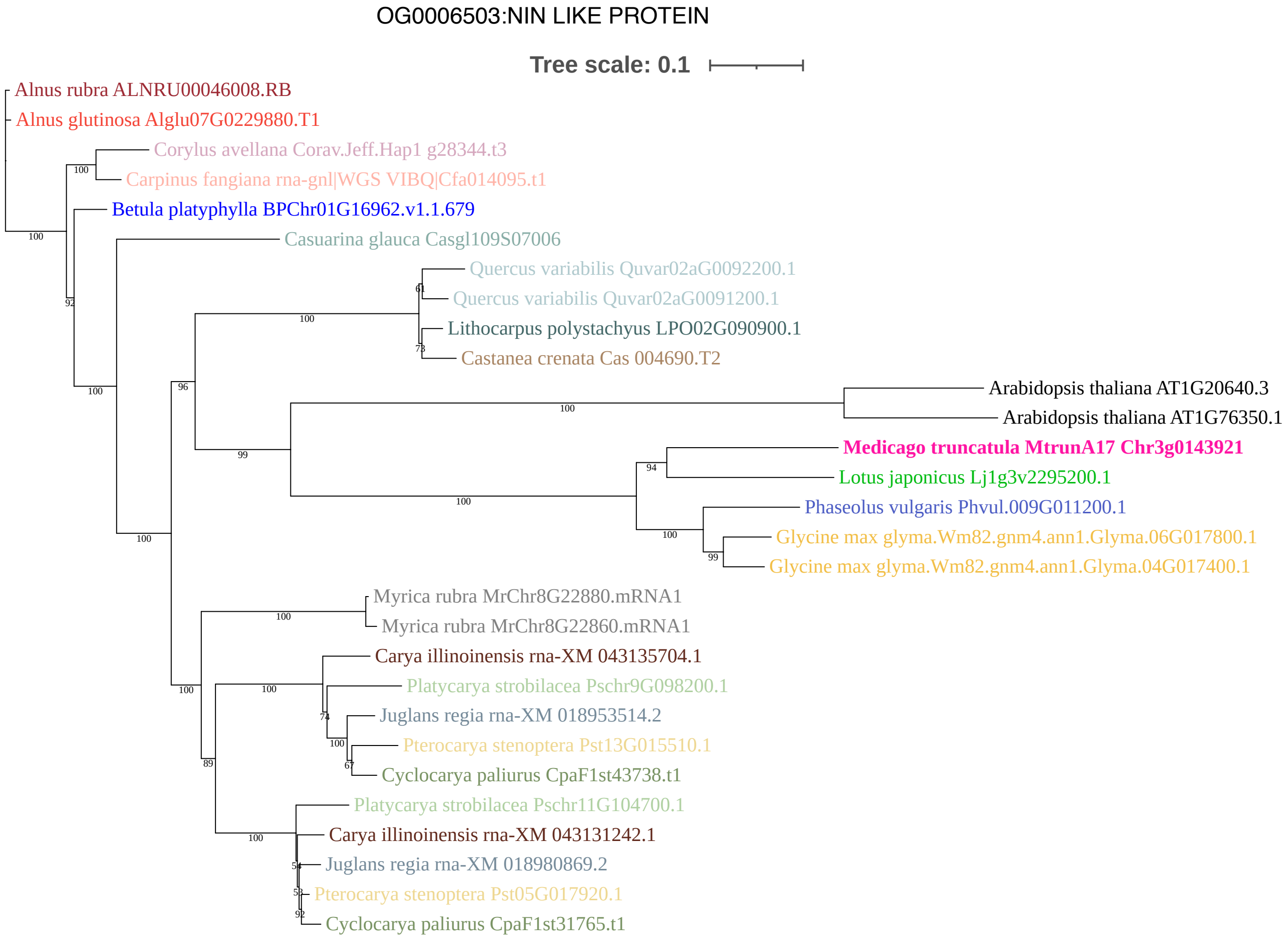
OG0006503:NIN LIKE PROTEIN
Tree scale: 0.1
Alnus rubra ALNRU00046008.RB
Alnus glutinosa Alglu07G0229880.T1
Corylus avellana Corav.Jeff.Hap1 g28344.t3
Carpinus fangiana rna-gnl|WGS VIBQ|Cfa014095.t1
Betula platyphylla BPChr01G16962.v1.1.679
Casuarina glauca Casgl109S07006
Quercus variabilis Quvar02aG0092200.1
Quercus variabilis Quvar02aG0091200.1
Lithocarpus polystachyus LPO02G090900.1
Castanea crenata Cas 004690.T2
Arabidopsis thaliana AT1G20640.3
Arabidopsis thaliana AT1G76350.1
Medicago truncatula MtrunA17 Chr3g0143921
Lotus japonicus Lj1g3v2295200.1
Phaseolus vulgaris Phvul.009G011200.1
Glycine max glyma.Wm82.gnm4.ann1.Glyma.06G017800.1
Glycine max glyma.Wm82.gnm4.ann1.Glyma.04G017400.1
Myrica rubra MrChr8G22880.mRNA1
Myrica rubra MrChr8G22860.mRNA1
Carya illinoinensis rna-XM 043135704.1
Platycarya strobilacea Pschr9G098200.1
Juglans regia rna-XM 018953514.2
Pterocarya stenoptera Pst13G015510.1
Cyclocarya paliurus CpaF1st43738.t1
Platycarya strobilacea Pschr11G104700.1
Carya illinoinensis rna-XM 043131242.1
Juglans regia rna-XM 018980869.2
Pterocarya stenoptera Pst05G017920.1
Cyclocarya paliurus CpaF1st31765.t1

OG0006520:Myeloblastosis(MYB) transcription factor 120

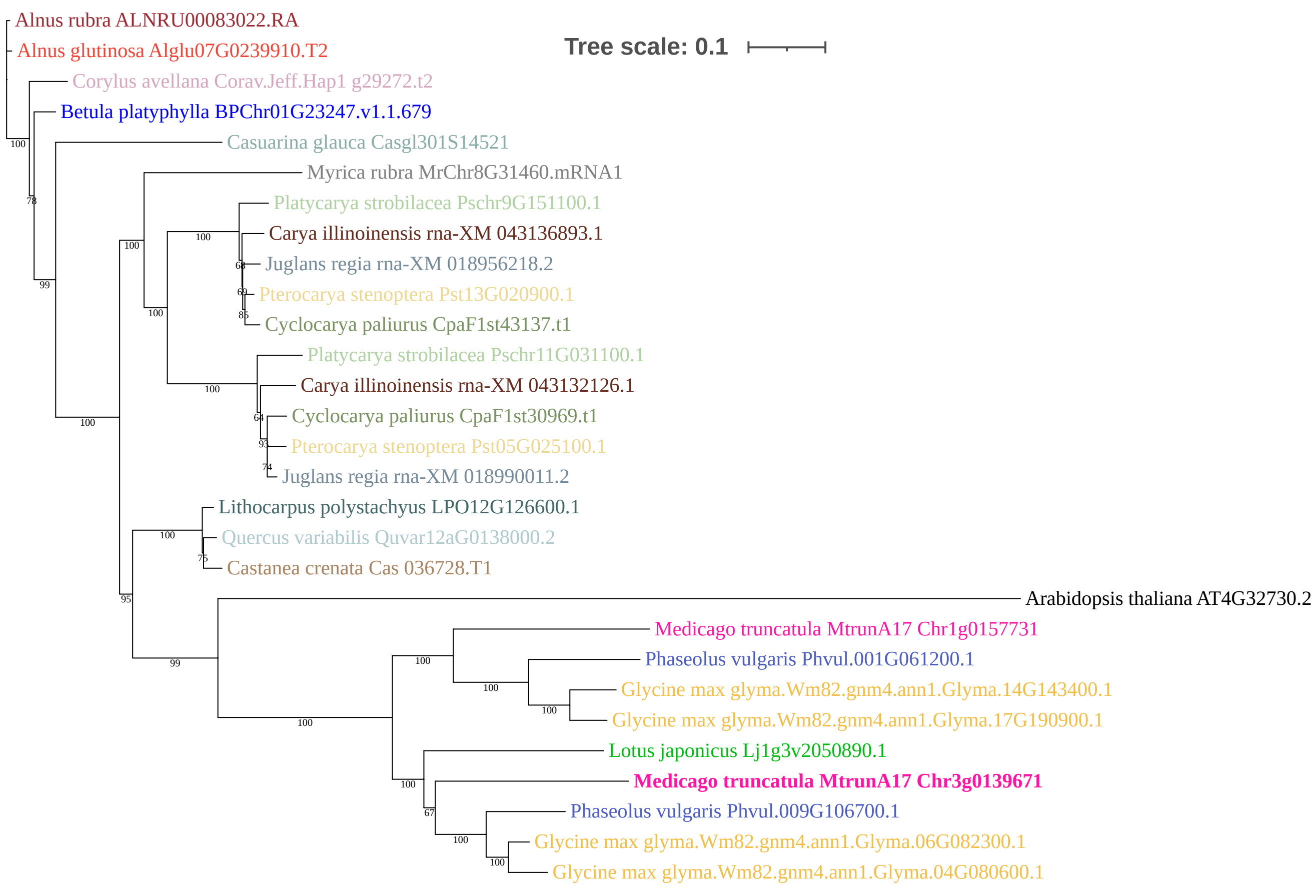

OG0006521:signal peptide peptidase

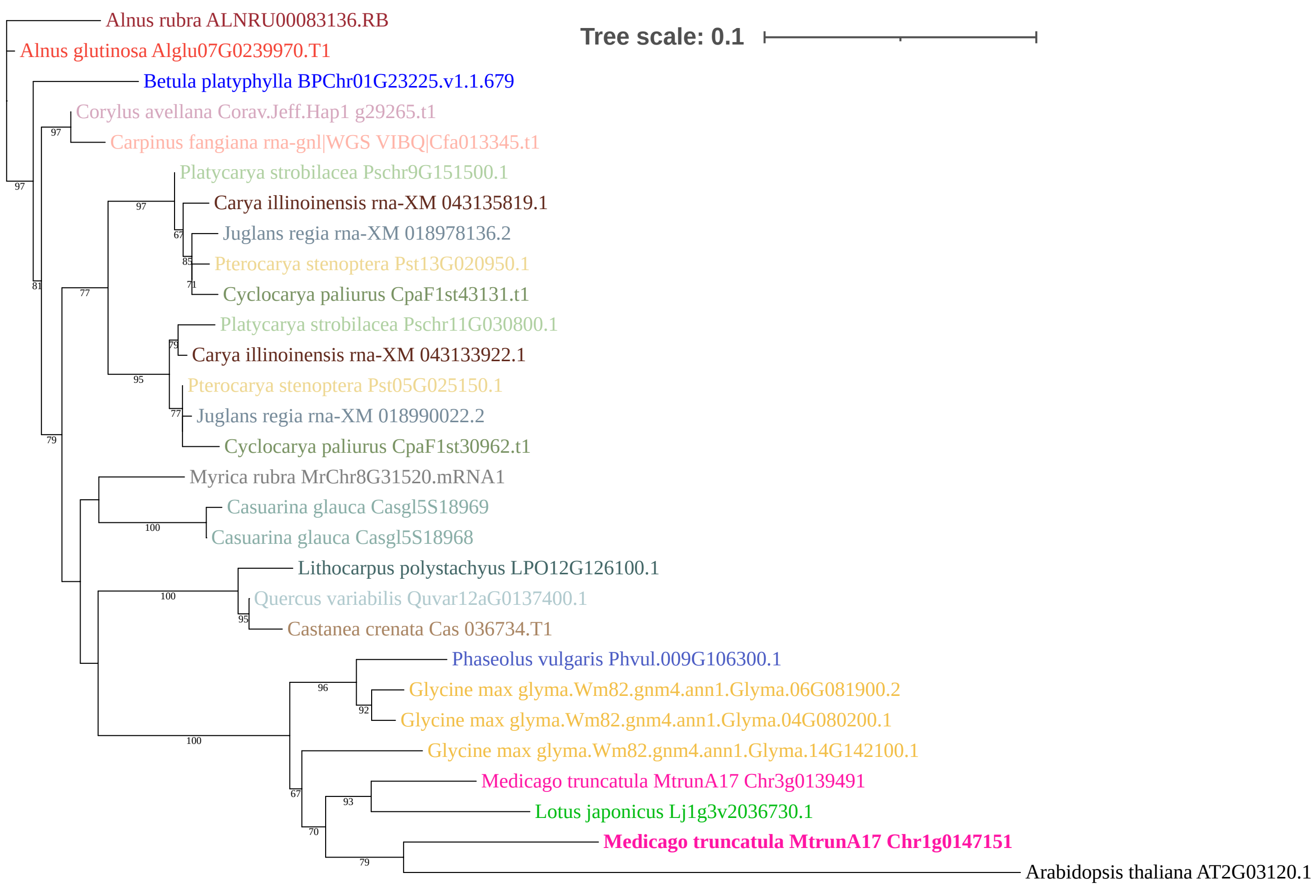

OG0006576:pathogenesis-related protein 1

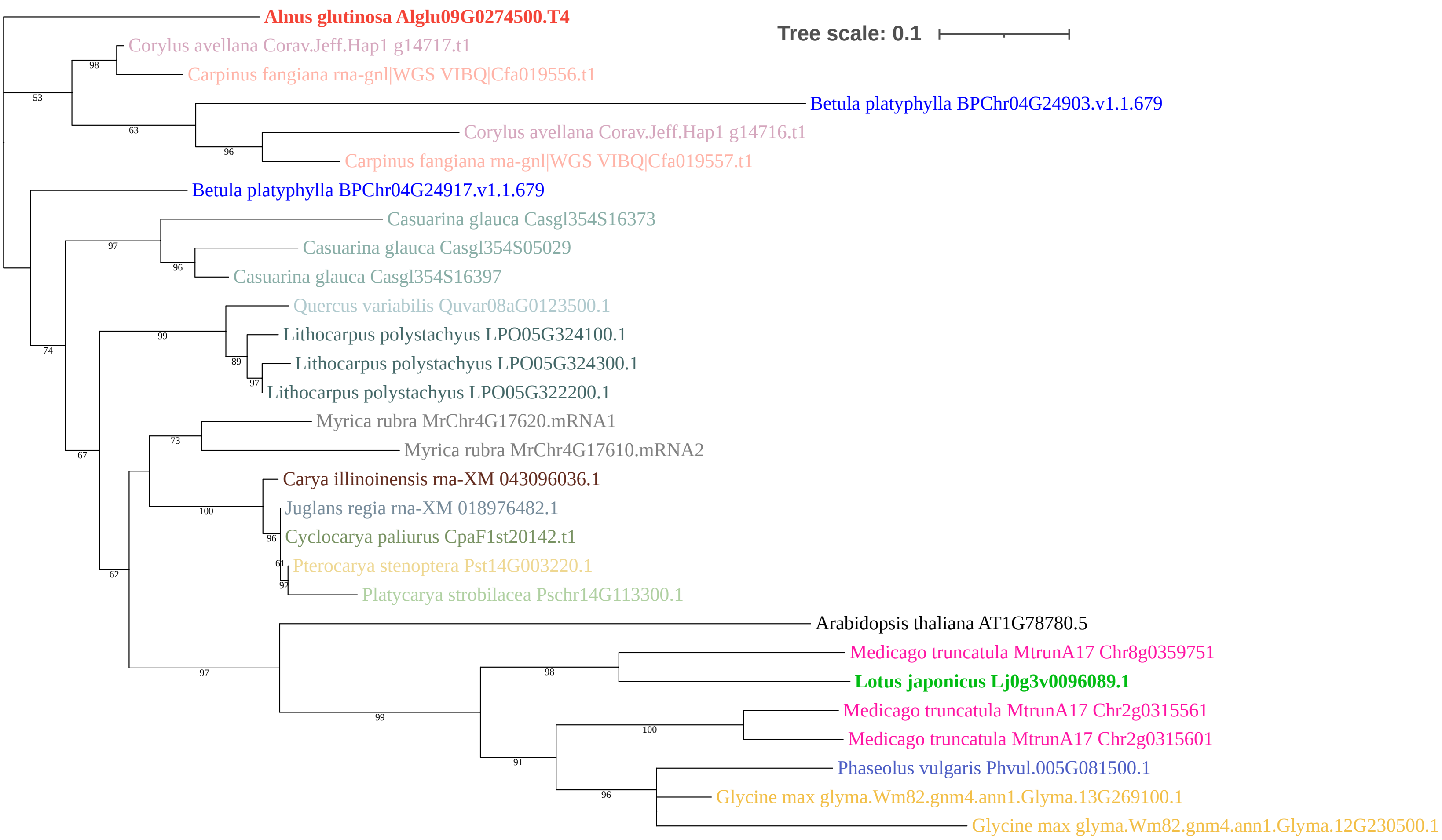

OG0006634:plastid glutamine synthetase

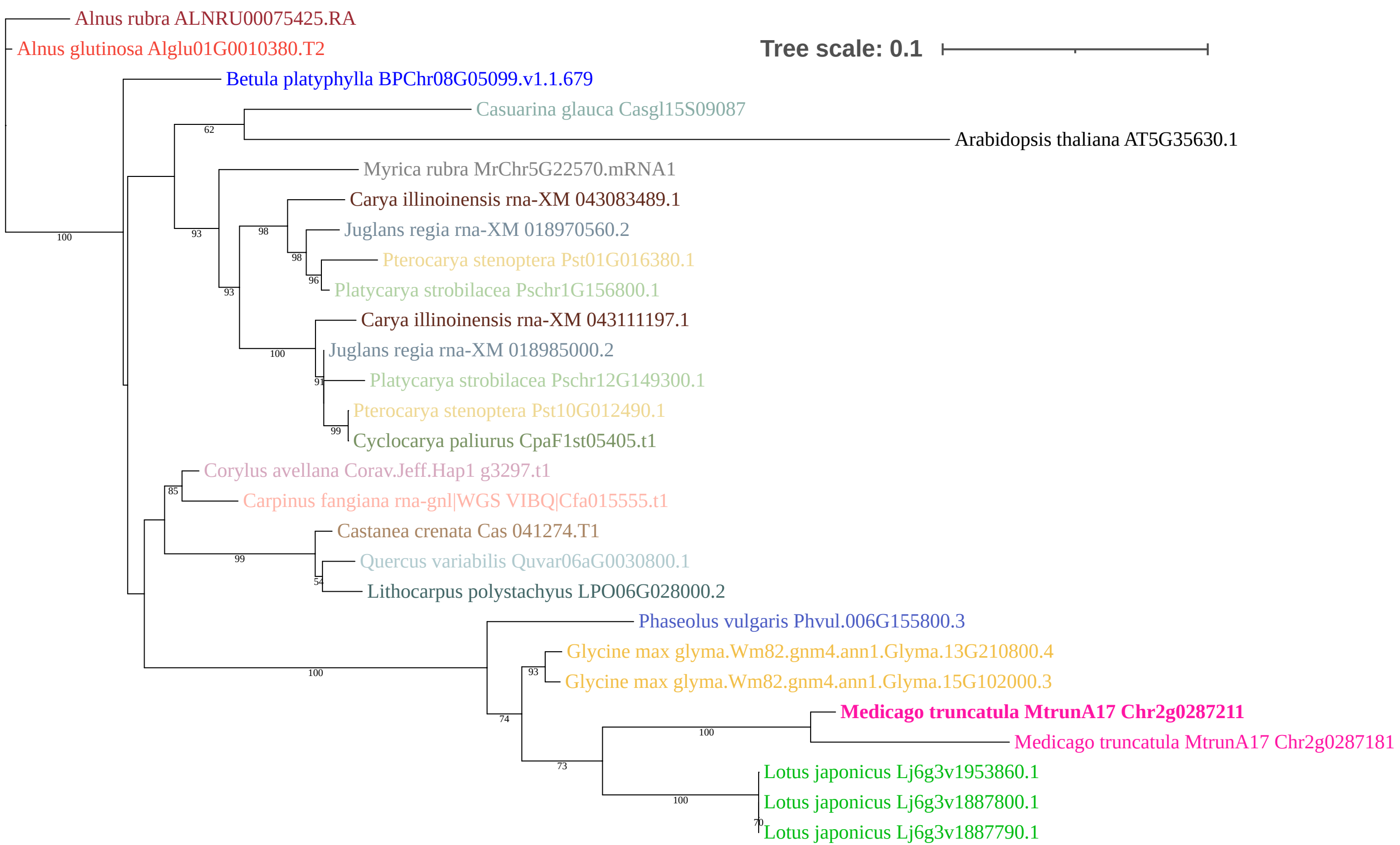

OG0006743:NODULE INCEPTION

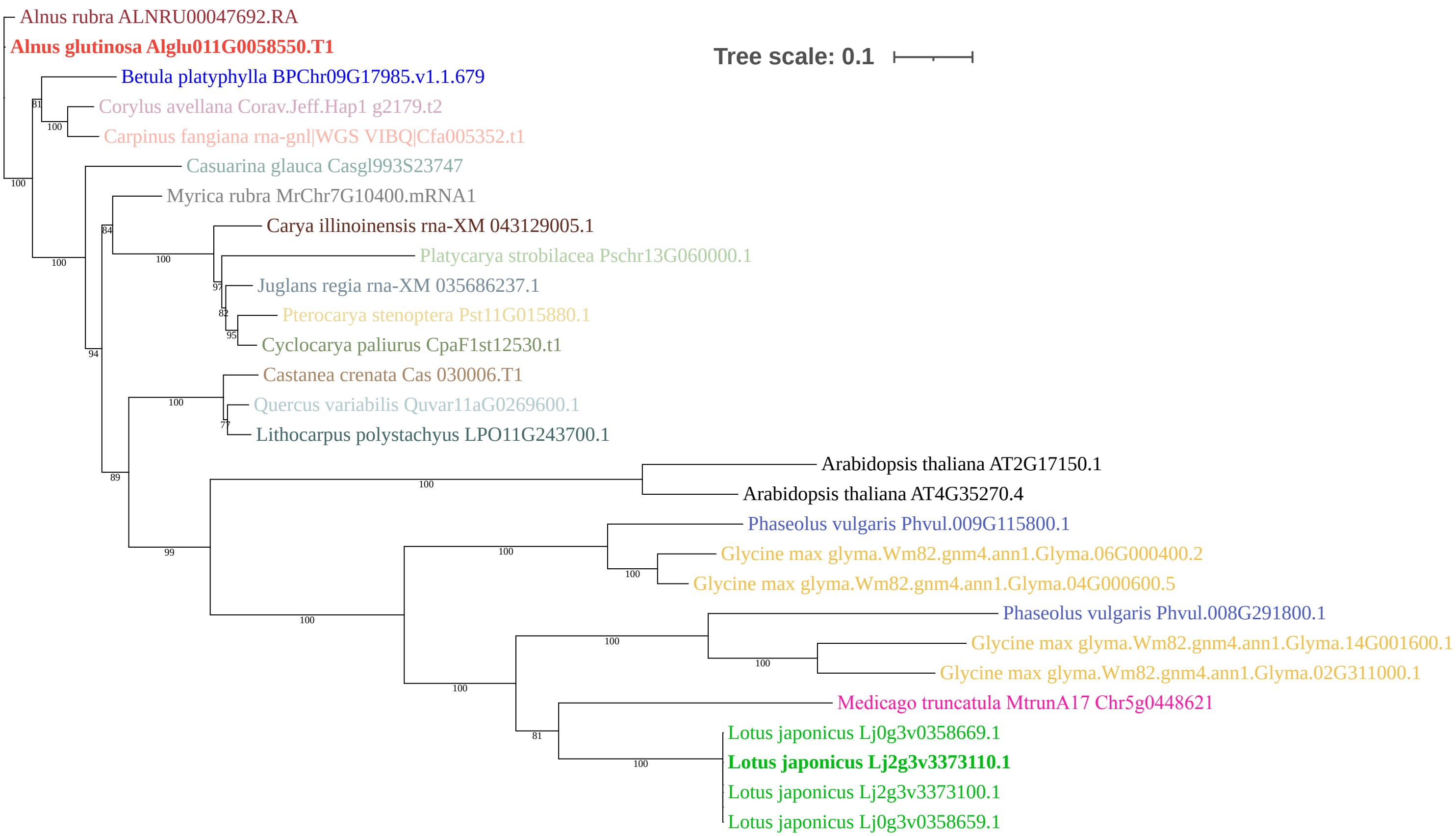

OG0006779:ACYL CARRIER PROTEIN

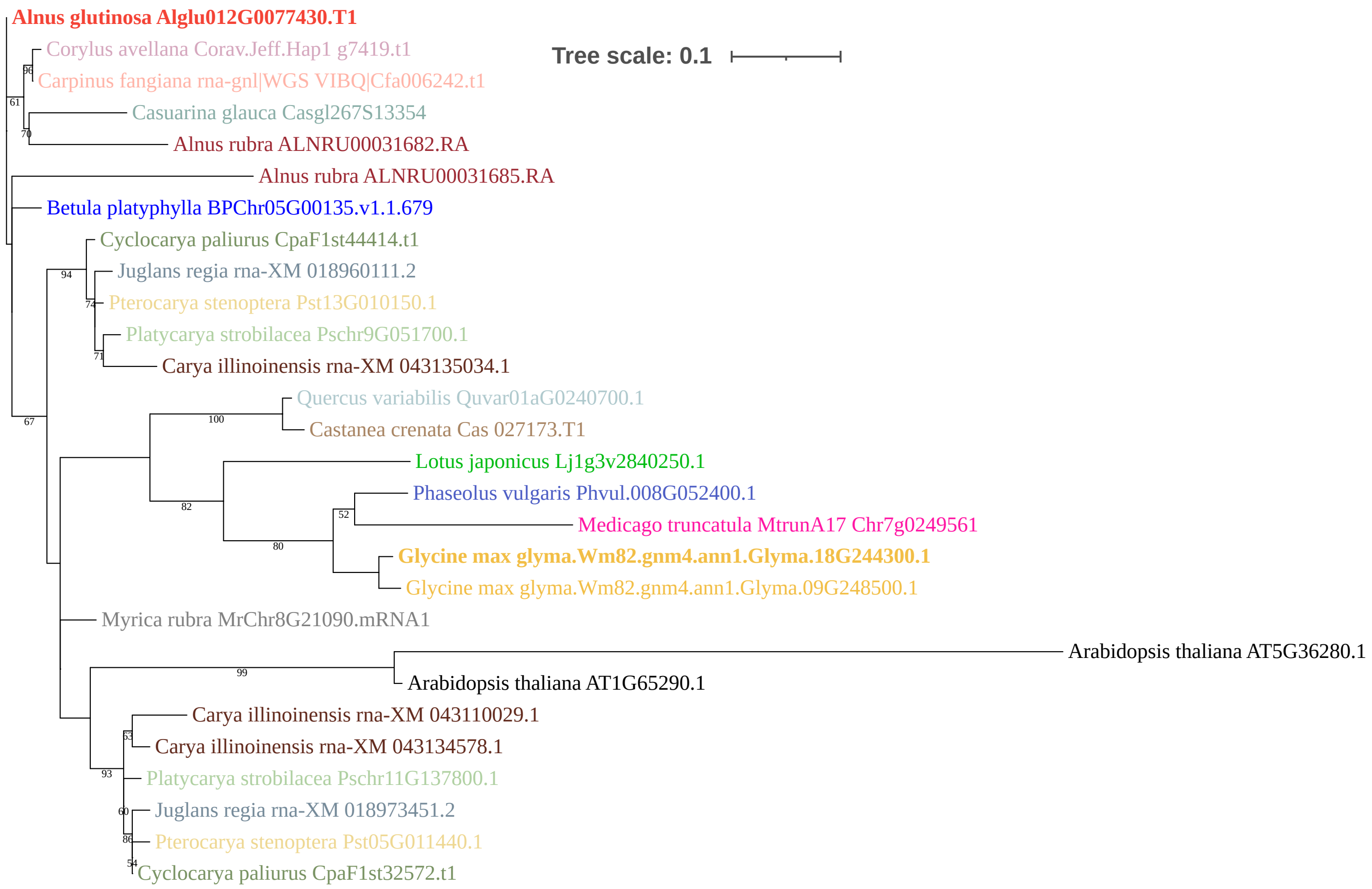

OG0006838:MtDWARF27(beta carotene isomeraselstrigolactone biosynthesis)

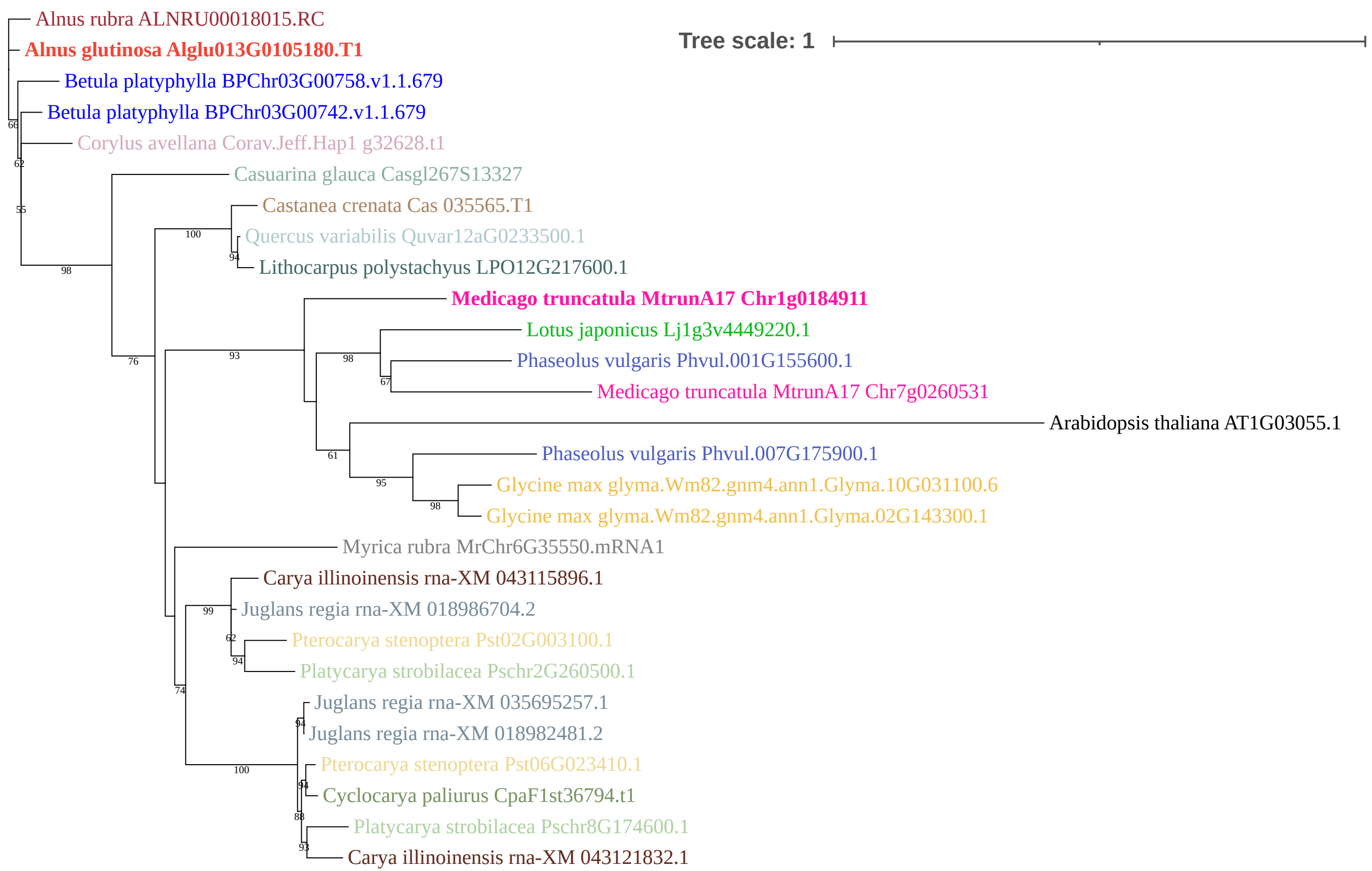

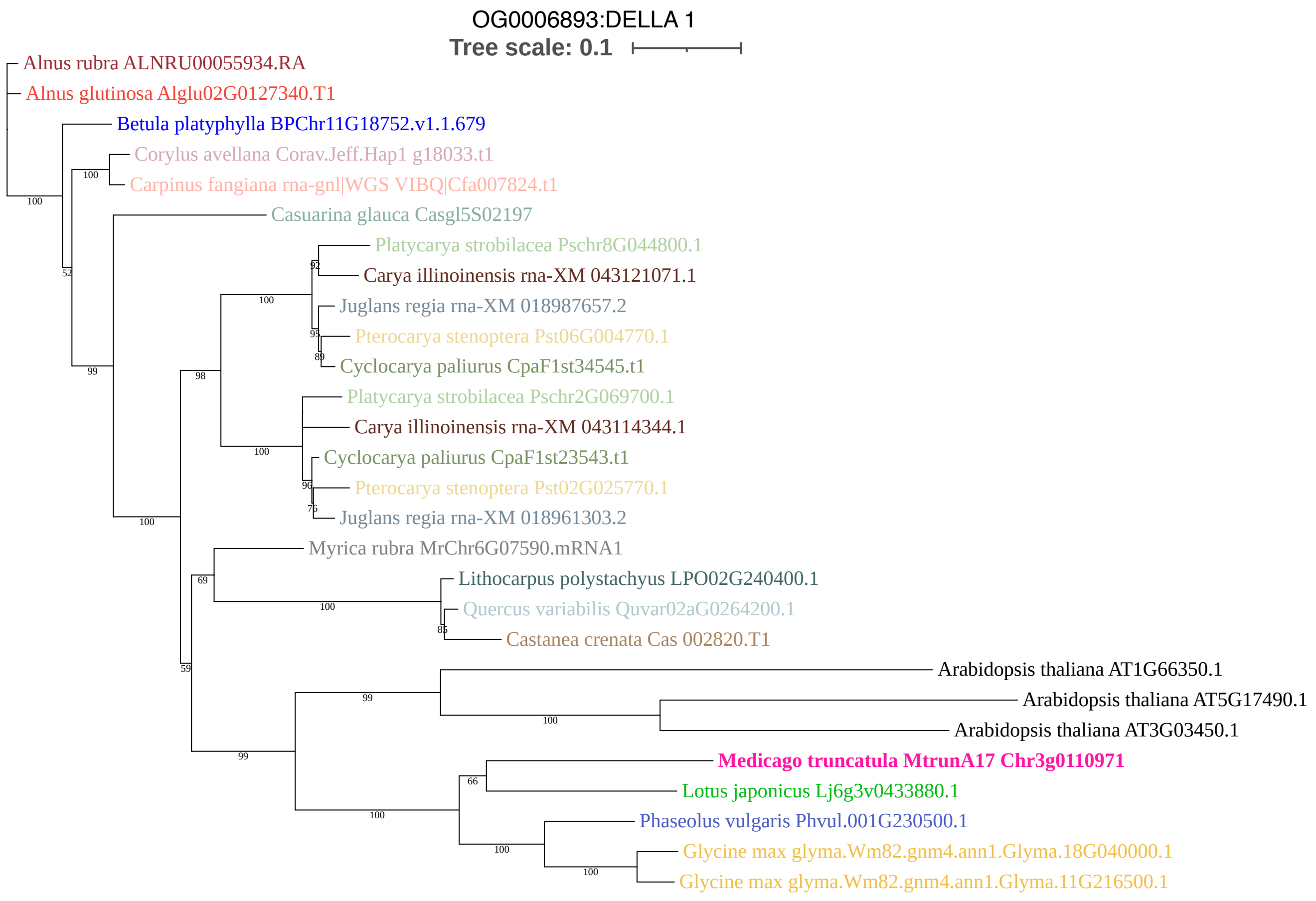

OG0006893:DELLA 1
Tree scale: 0.1
Alnus rubra ALNRU00055934.RA
Alnus glutinosa Alglu02G0127340.T1
Betula platyphylla BPChr11G18752.v1.1.679
Corylus avellana Corav.Jeff.Hap1 g18033.t1
Carpinus fangiana rna-gnl|WGS VIBQ|Cfa007824.t1
Casuarina glauca Casgl5S02197
Platycarya strobilacea Pschr8G044800.1
Carya illinoinensis rna-XM 043121071.1
Juglans regia rna-XM 018987657.2
Pterocarya stenoptera Pst06G004770.1
Cyclocarya paliurus CpaF1st34545.t1
Platycarya strobilacea Pschr2G069700.1
Carya illinoinensis rna-XM 043114344.1
Cyclocarya paliurus CpaF1st23543.t1
Pterocarya stenoptera Pst02G025770.1
Juglans regia rna-XM 018961303.2
Myrica rubra MrChr6G07590.mRNA1
Lithocarpus polystachyus LPO02G240400.1
Quercus variabilis Quvar02aG0264200.1
Castanea crenata Cas 002820.T1
Arabidopsis thaliana AT1G66350.1
Arabidopsis thaliana AT5G17490.1
Arabidopsis thaliana AT3G03450.1
Medicago truncatula MtrunA17 Chr3g0110971
Lotus japonicus Lj6g3v0433880.1
Phaseolus vulgaris Phvul.001G230500.1
Glycine max glyma.Wm82.gnm4.ann1.Glyma.18G040000.1
Glycine max glyma.Wm82.gnm4.ann1.Glyma.11G216500.1

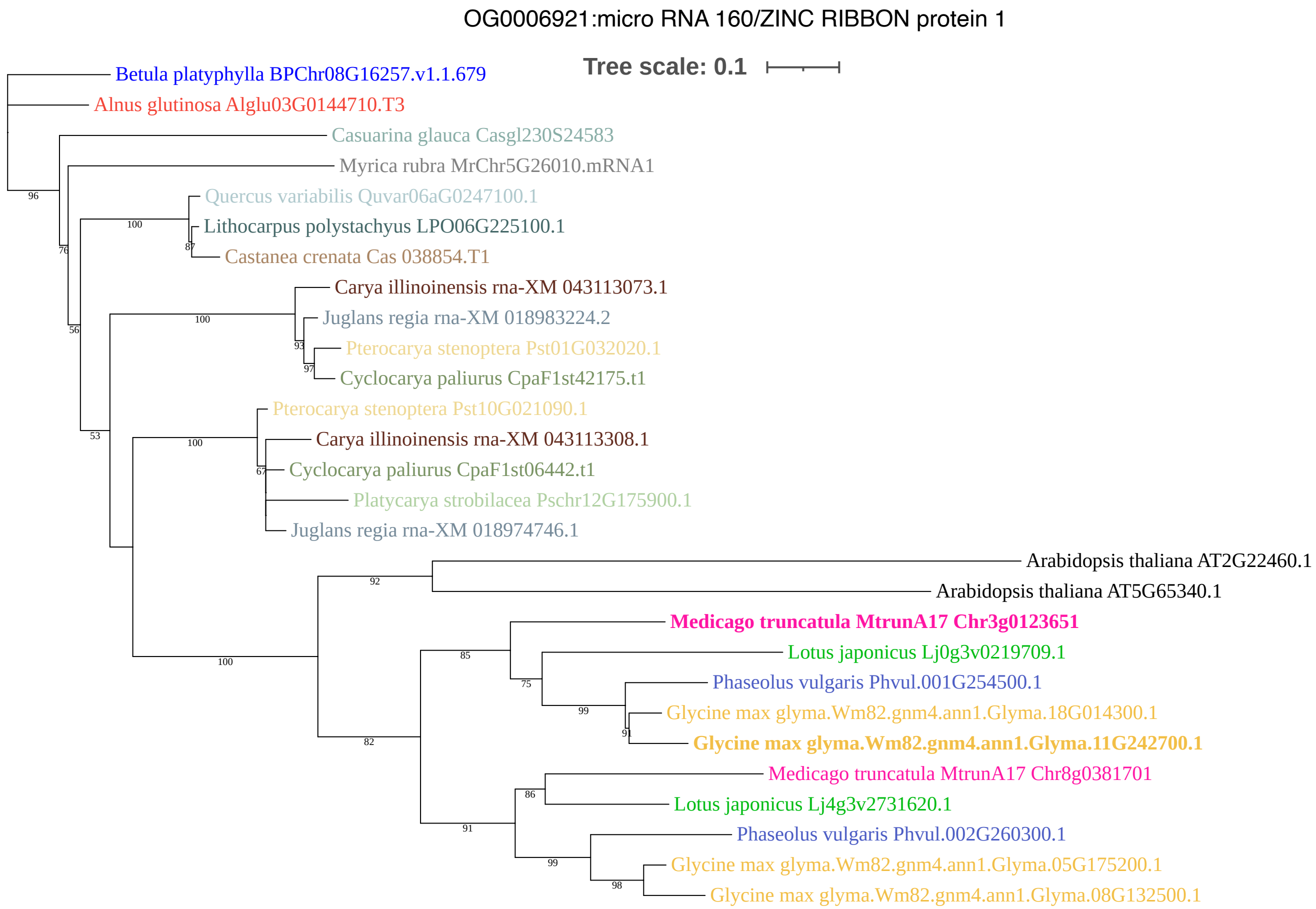
OG0006921:micro RNA 160/ZINC RIBBON protein 1
Tree scale: 0.1
Betula platyphylla BPChr08G16257.v1.1.679
Alnus glutinosa Alglu03G0144710.T3
Casuarina glauca Casgl230S24583
Myrica rubra MrChr5G26010.mRNA1
Quercus variabilis Quvar06aG0247100.1
Lithocarpus polystachyus LPO06G225100.1
Castanea crenata Cas 038854.T1
Carya illinoinensis rna-XM 043113073.1
Juglans regia rna-XM 018983224.2
Pterocarya stenoptera Pst01G032020.1
Cyclocarya paliurus CpaF1st42175.t1
Pterocarya stenoptera Pst10G021090.1
Carya illinoinensis rna-XM 043113308.1
Cyclocarya paliurus CpaF1st06442.t1
Platycarya strobilacea Pschr12G175900.1
Juglans regia rna-XM 018974746.1
Arabidopsis thaliana AT2G22460.1
Arabidopsis thaliana AT5G65340.1
Medicago truncatula MtrunA17 Chr3g0123651
Lotus japonicus Lj0g3v0219709.1
Phaseolus vulgaris Phvul.001G254500.1
Glycine max glyma.Wm82.gnm4.ann1.Glyma.18G014300.1
Glycine max glyma.Wm82.gnm4.ann1.Glyma.11G242700.1
Medicago truncatula MtrunA17 Chr8g0381701
Lotus japonicus Lj4g3v2731620.1
Phaseolus vulgaris Phvul.002G260300.1
Glycine max glyma.Wm82.gnm4.ann1.Glyma.05G175200.1
Glycine max glyma.Wm82.gnm4.ann1.Glyma.08G132500.1

OG0006996:sucrose transporter

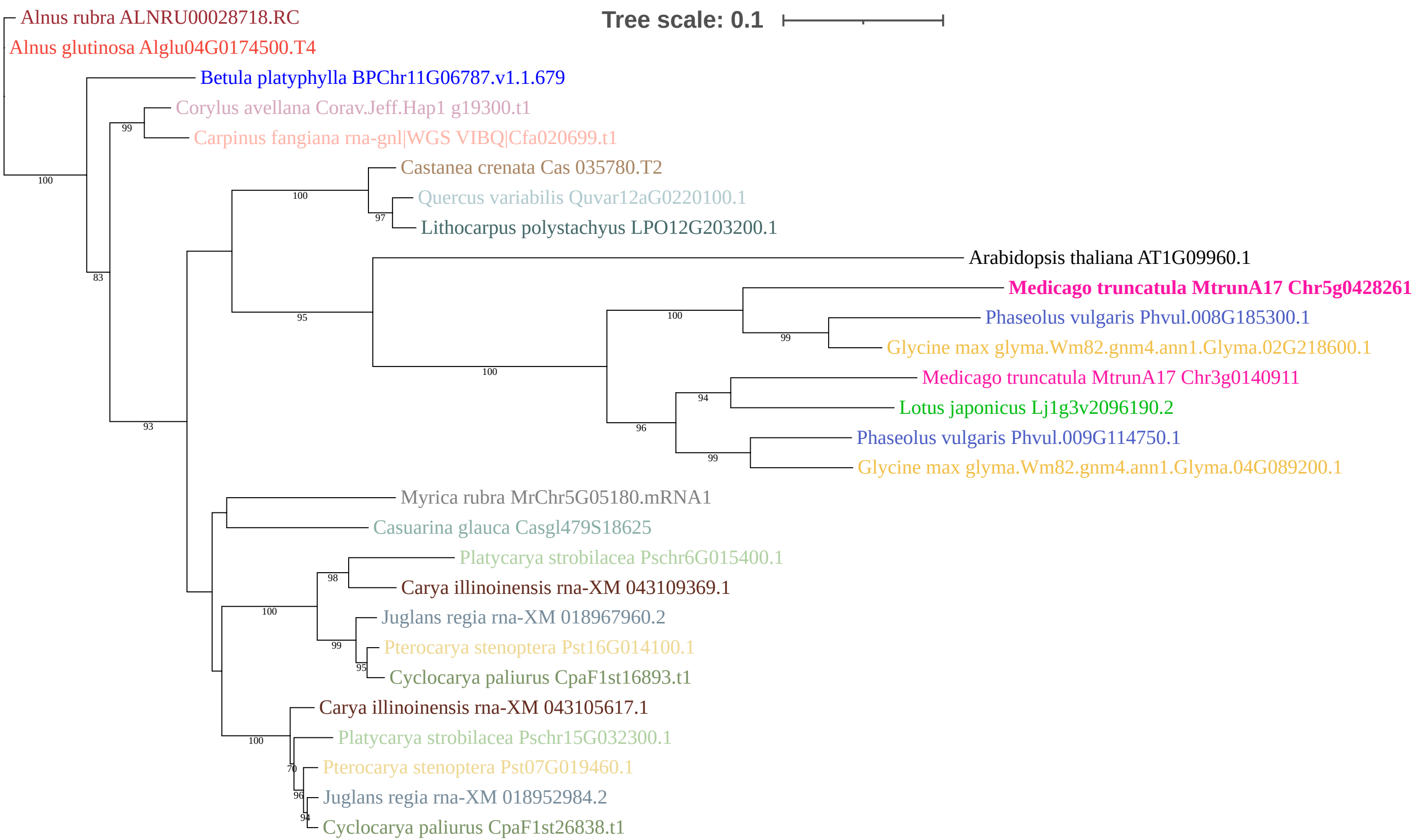

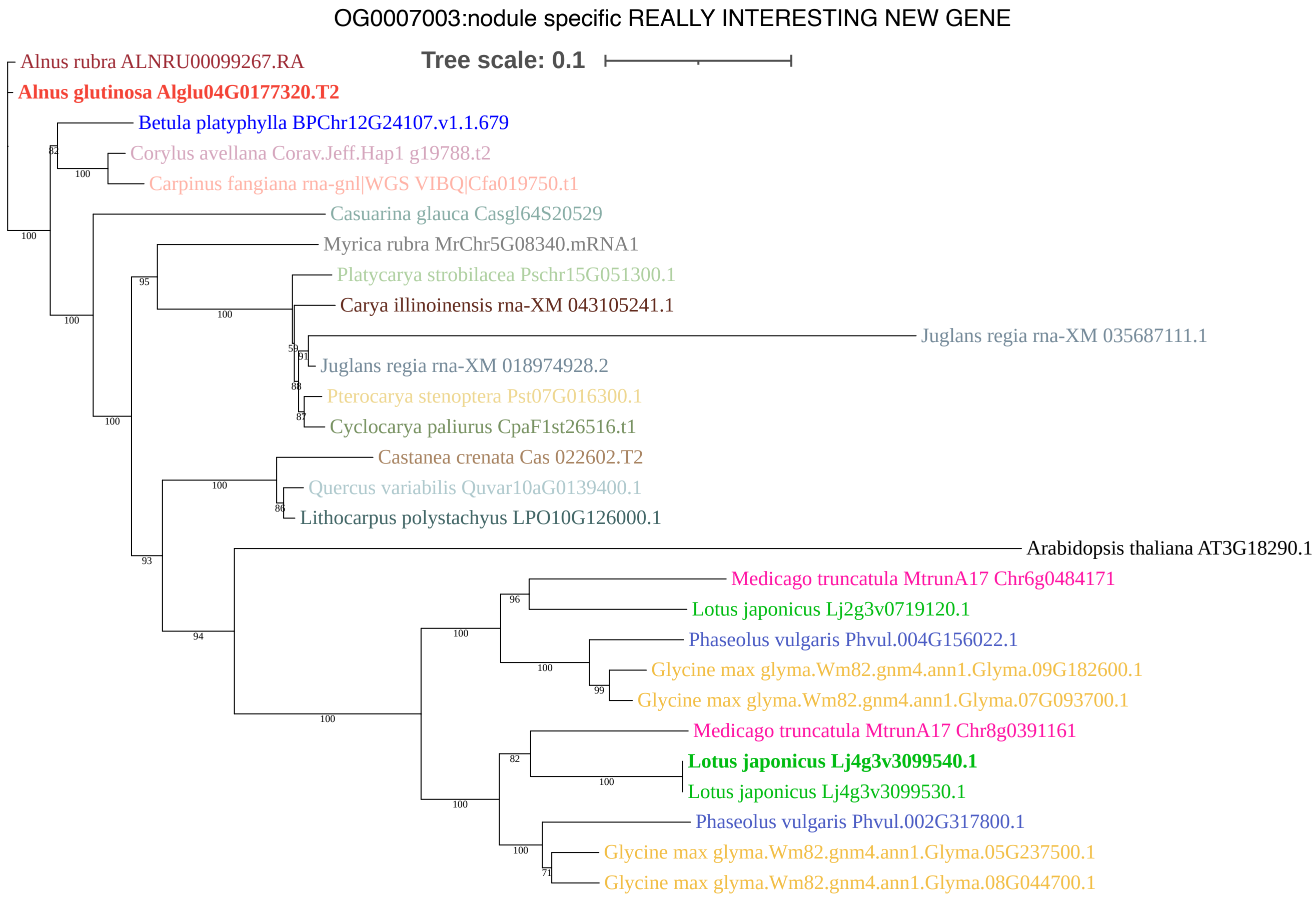
OG0007003:nodule specific REALLY INTERESTING NEW GENE
Tree scale: 0.1
Alnus rubra ALNRU00099267.RA
Alnus glutinosa Alglu04G0177320.T2
Betula platyphylla BPChr12G24107.v1.1.679
Corylus avellana Corav.Jeff.Hap1 g19788.t2
Carpinus fangiana rna-gnl|WGS VIBQ|Cfa019750.t1
Casuarina glauca Casgl64S20529
Myrica rubra MrChr5G08340.mRNA1
Platycarya strobilacea Pschr15G051300.1
Carya illinoinensis rna-XM 043105241.1
Juglans regia rna-XM 035687111.1
Juglans regia rna-XM 018974928.2
Pterocarya stenoptera Pst07G016300.1
Cyclocarya paliurus CpaF1st26516.t1
Castanea crenata Cas 022602.T2
Quercus variabilis Quvar10aG0139400.1
Lithocarpus polystachyus LPO10G126000.1
Arabidopsis thaliana AT3G18290.1
Medicago truncatula MtrunA17 Chr6g0484171
Lotus japonicus Lj2g3v0719120.1
Phaseolus vulgaris Phvul.004G156022.1
Glycine max glyma.Wm82.gnm4.ann1.Glyma.09G182600.1
Glycine max glyma.Wm82.gnm4.ann1.Glyma.07G093700.1
Medicago truncatula MtrunA17 Chr8g0391161
Lotus japonicus Lj4g3v3099540.1
Lotus japonicus Lj4g3v3099530.1
Phaseolus vulgaris Phvul.002G317800.1
Glycine max glyma.Wm82.gnm4.ann1.Glyma.05G237500.1
Glycine max glyma.Wm82.gnm4.ann1.Glyma.08G044700.1

OG0007028:Nodulation Receptor Kinase?Interacting Small Protein

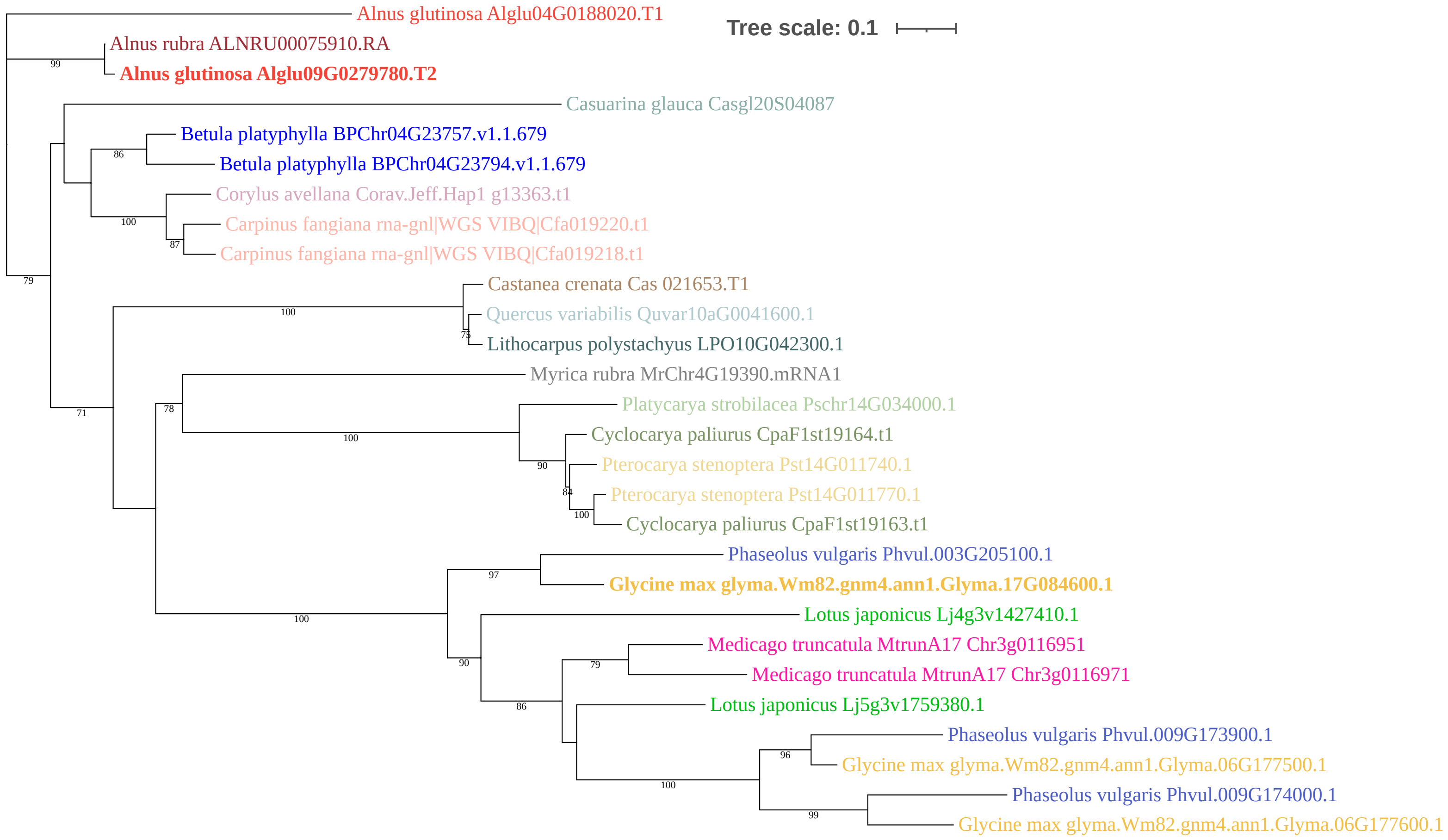

OG0007101:lumpy infections|Plant U-box protein 9

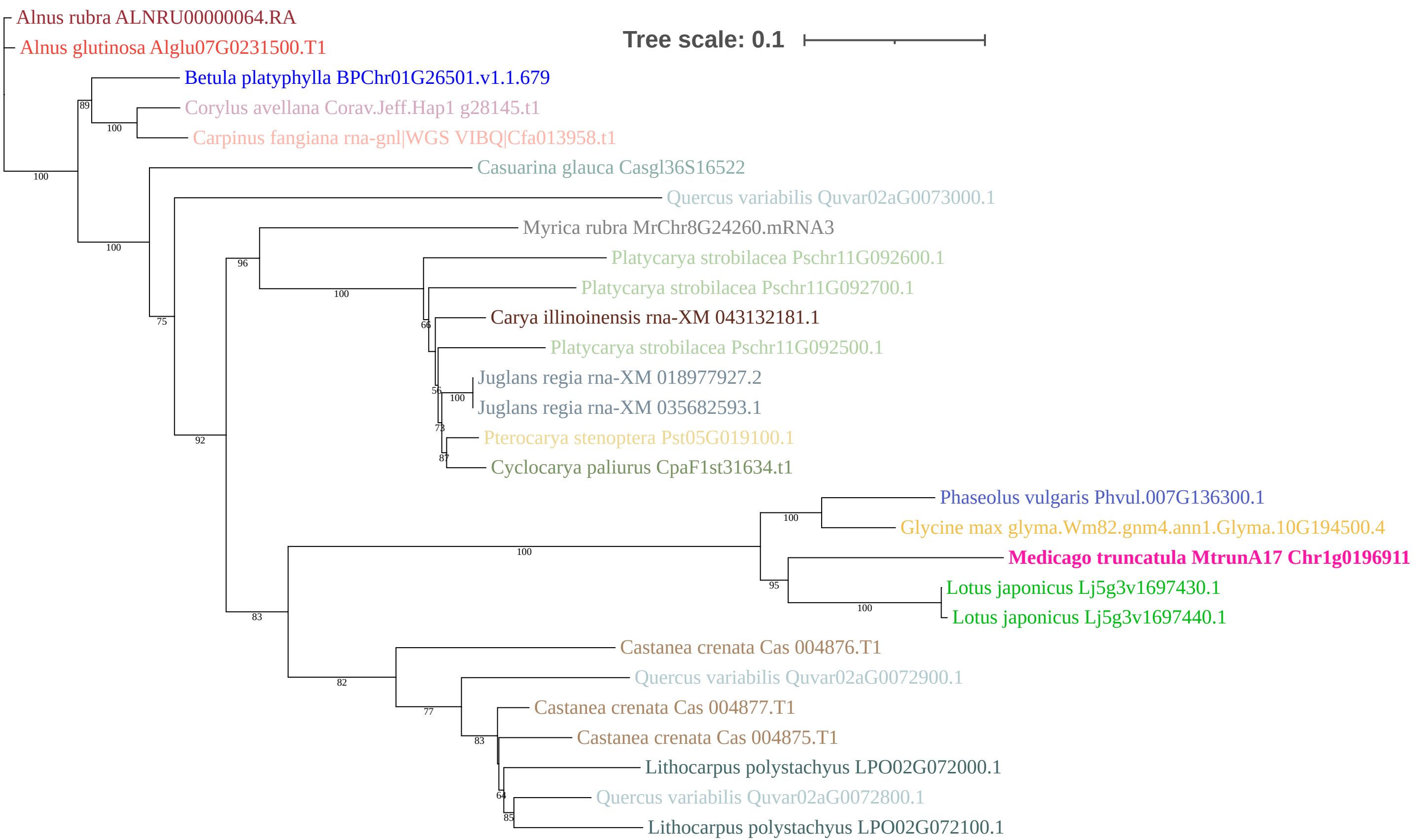

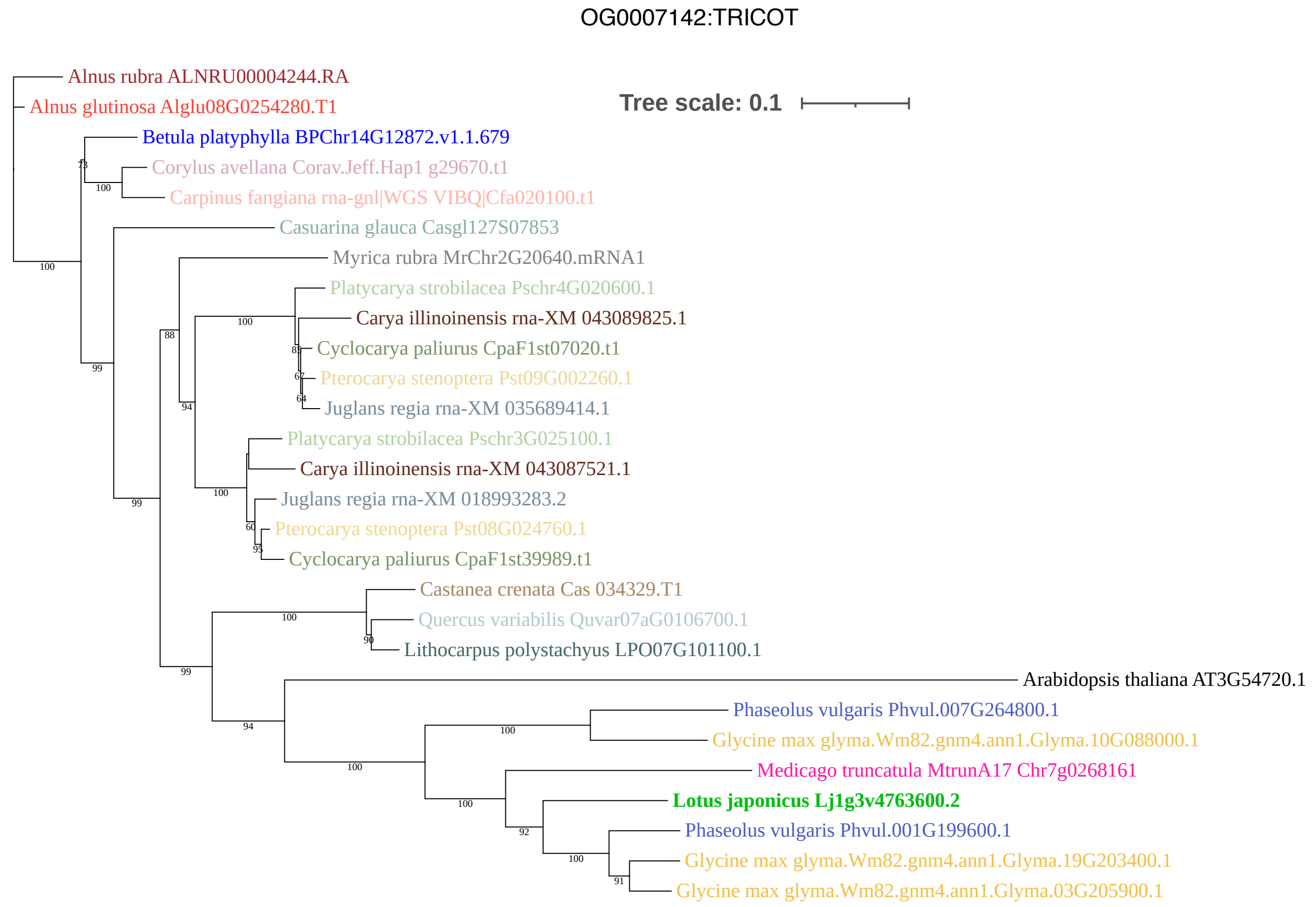
OG0007142:TRICOT
Tree scale: 0.1
Alnus rubra ALNRU00004244.RA
Alnus glutinosa Alglu08G0254280.T1
Betula platyphylla BPChr14G12872.v1.1.679
Corylus avellana Corav.Jeff.Hap1 g29670.t1
Carpinus fangiana rna-gnl|WGS VIBQ|Cfa020100.t1
Casuarina glauca Casgl127S07853
Myrica rubra MrChr2G20640.mRNA1
Platycarya strobilacea Pschr4G020600.1
Carya illinoinensis rna-XM 043089825.1
Cyclocarya paliurus CpaF1st07020.t1
Pterocarya stenoptera Pst09G002260.1
Juglans regia rna-XM 035689414.1
Platycarya strobilacea Pschr3G025100.1
Carya illinoinensis rna-XM 043087521.1
Juglans regia rna-XM 018993283.2
Pterocarya stenoptera Pst08G024760.1
Cyclocarya paliurus CpaF1st39989.t1
Castanea crenata Cas 034329.T1
Quercus variabilis Quvar07aG0106700.1
Lithocarpus polystachyus LPO07G101100.1
Arabidopsis thaliana AT3G54720.1
Phaseolus vulgaris Phvul.007G264800.1
Glycine max glyma.Wm82.gnm4.ann1.Glyma.10G088000.1
Medicago truncatula MtrunA17 Chr7g0268161
Lotus japonicus Lj1g3v4763600.2
Phaseolus vulgaris Phvul.001G199600.1
Glycine max glyma.Wm82.gnm4.ann1.Glyma.19G203400.1
Glycine max glyma.Wm82.gnm4.ann1.Glyma.03G205900.1

OG0007254:FAIL IN ENLARGEMENT of infection CELLS

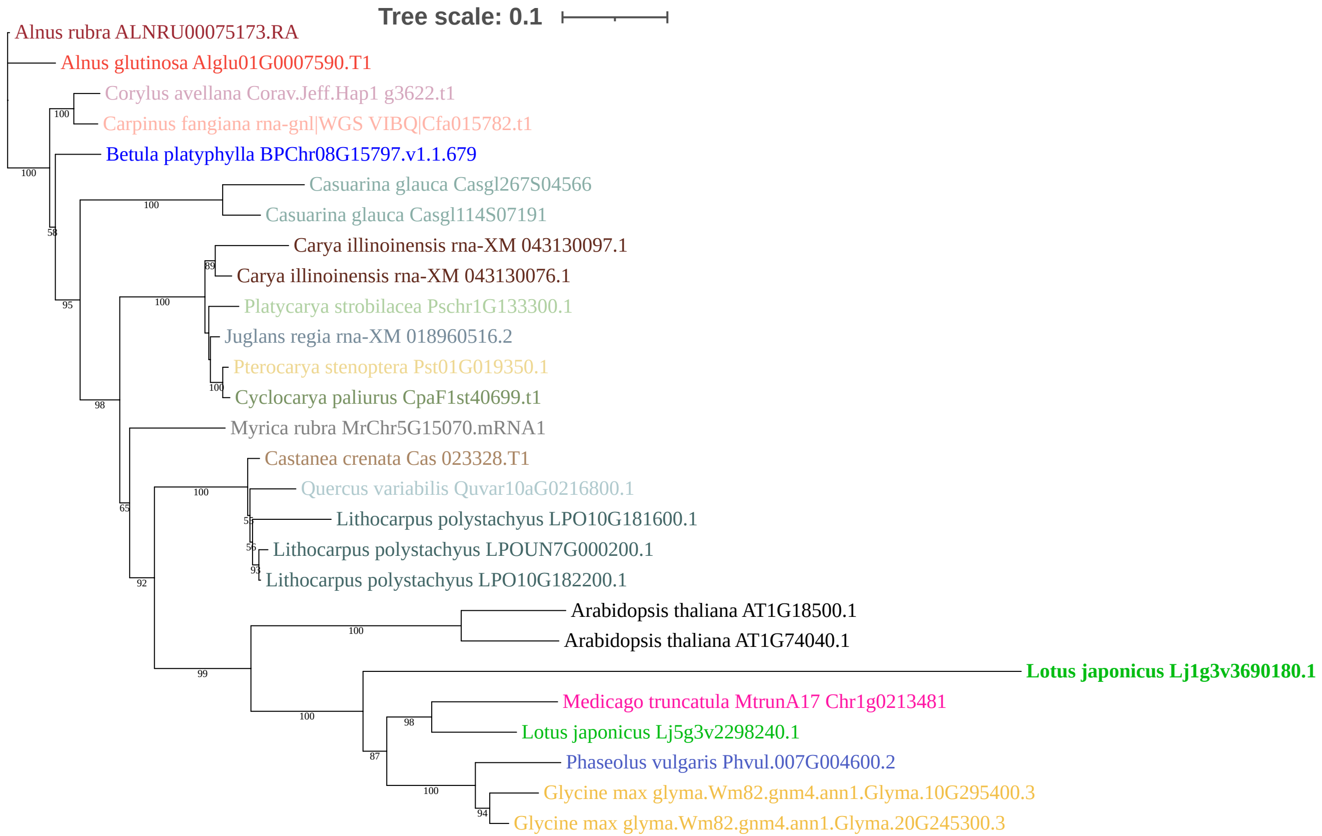

OG0007277:NIN-like protein 2

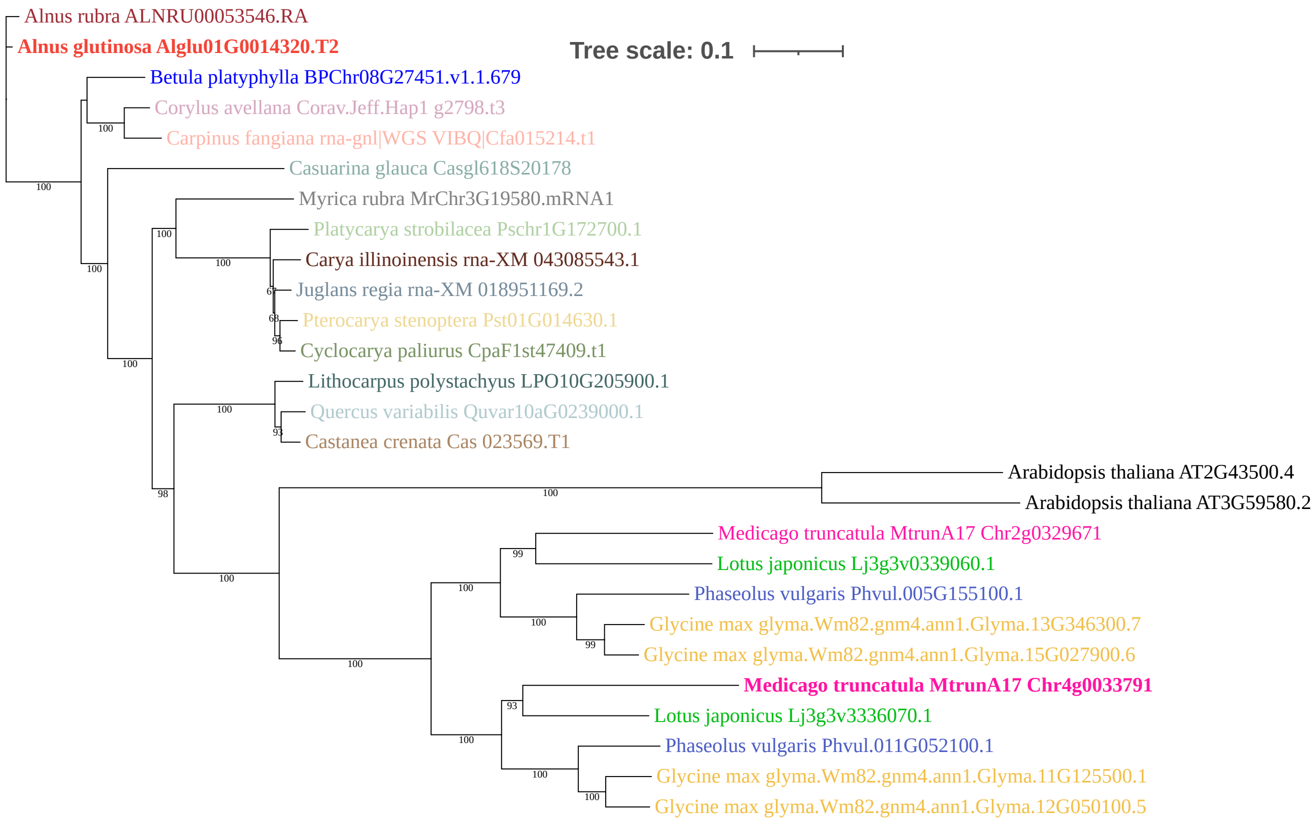

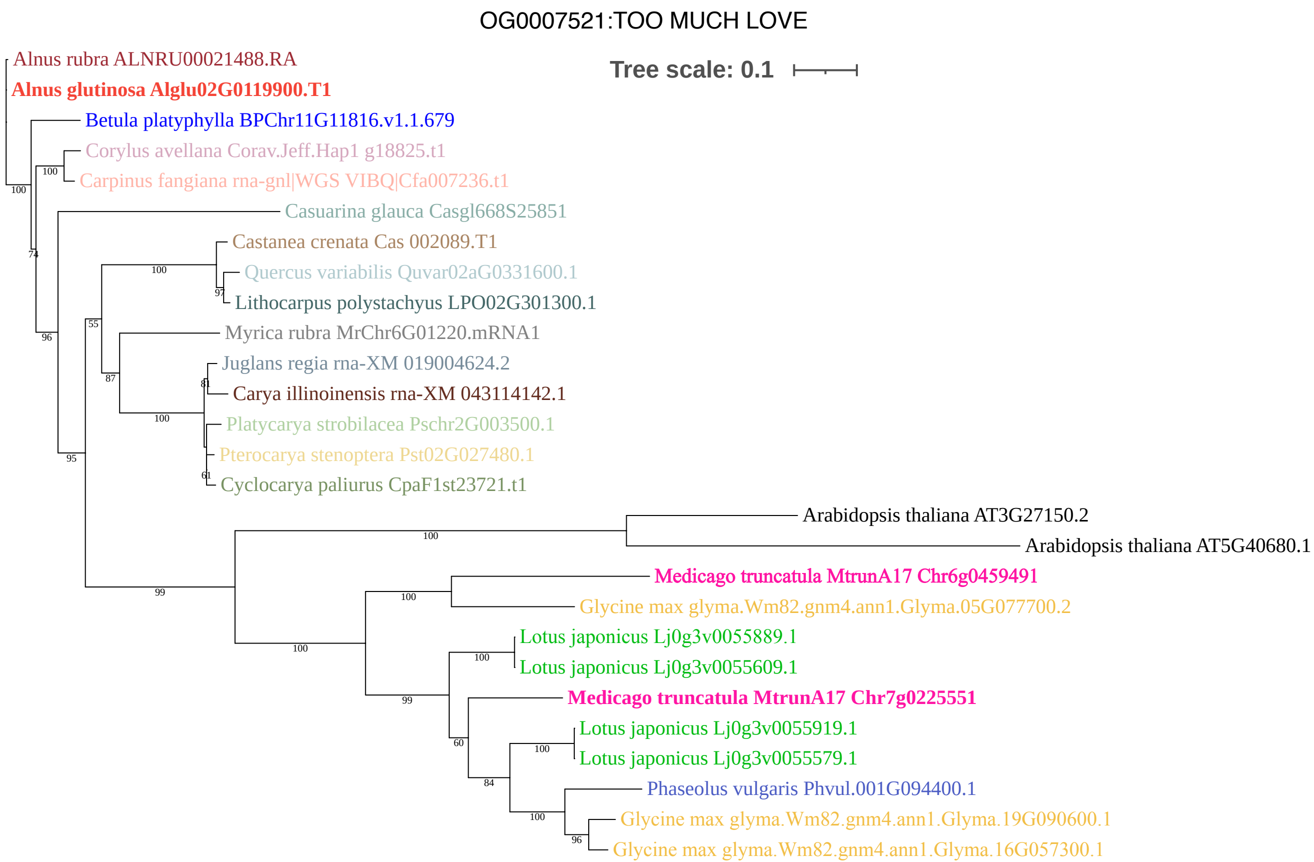
OG0007521:TOO MUCH LOVE
Tree scale: 0.1
Alnus rubra ALNRU00021488.RA
Alnus glutinosa Alglu02G0119900.T1
Betula platyphylla BPChr11G11816.v1.1.679
Corylus avellana Corav.Jeff.Hap1 g18825.t1
Carpinus fangiana rna-gnl|WGS VIBQ|Cfa007236.t1
Casuarina glauca Casgl668S25851
Castanea crenata Cas 002089.T1
Quercus variabilis Quvar02aG0331600.1
Lithocarpus polystachyus LPO02G301300.1
Myrica rubra MrChr6G01220.mRNA1
Juglans regia rna-XM 019004624.2
Carya illinoinensis rna-XM 043114142.1
Platycarya strobilacea Pschr2G003500.1
Pterocarya stenoptera Pst02G027480.1
Cyclocarya paliurus CpaF1st23721.t1
Arabidopsis thaliana AT3G27150.2
Arabidopsis thaliana AT5G40680.1
Medicago truncatula MtrunA17 Chr6g0459491
Glycine max glyma.Wm82.gnm4.ann1.Glyma.05G077700.2
Lotus japonicus Lj0g3v0055889.1
Lotus japonicus Lj0g3v0055609.1
Medicago truncatula MtrunA17 Chr7g0225551
Lotus japonicus Lj0g3v0055919.1
Lotus japonicus Lj0g3v0055579.1
Phaseolus vulgaris Phvul.001G094400.1
Glycine max glyma.Wm82.gnm4.ann1.Glyma.19G090600.1
Glycine max glyma.Wm82.gnm4.ann1.Glyma.16G057300.1

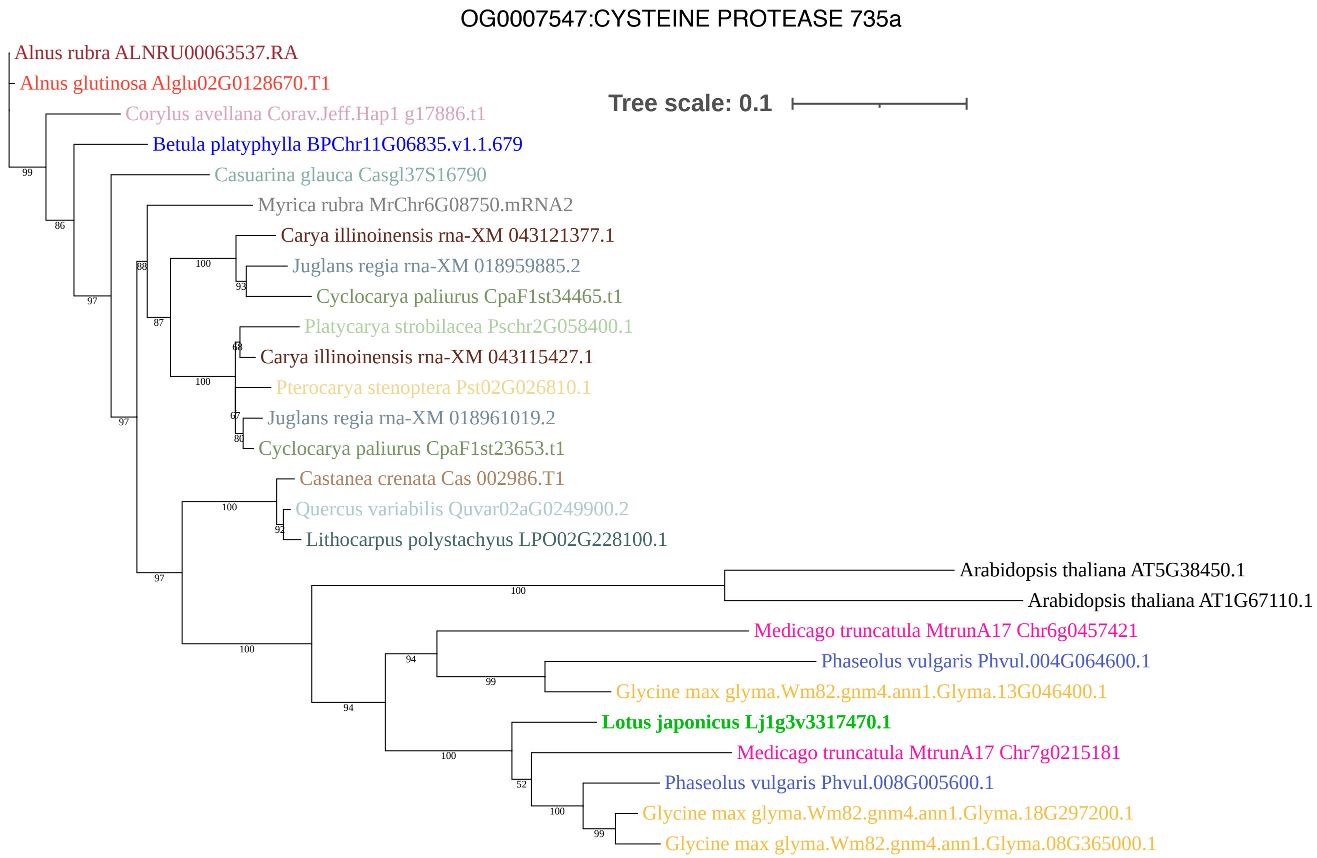

OG0007547:CYSTEINE PROTEASE 735a
Tree scale: 0.1
Alnus rubra ALNRU00063537.RA
Alnus glutinosa Alglu02G0128670.T1
Corylus avellana Corav.Jeff.Hap1 g17886.t1
Betula platyphylla BPChr11G06835.v1.1.679
Casuarina glauca Casgl37S16790
Myrica rubra MrChr6G08750.mRNA2
Carya illinoinensis rna-XM 043121377.1
Juglans regia rna-XM 018959885.2
Cyclocarya paliurus CpaF1st34465.t1
Platycarya strobilacea Pschr2G058400.1
Carya illinoinensis rna-XM 043115427.1
Pterocarya stenoptera Pst02G026810.1
Juglans regia rna-XM 018961019.2
Cyclocarya paliurus CpaF1st23653.t1
Castanea crenata Cas 002986.T1
Quercus variabilis Quvar02aG0249900.2
Lithocarpus polystachyus LPO02G228100.1
Arabidopsis thaliana AT5G38450.1
Arabidopsis thaliana AT1G67110.1
Medicago truncatula MtrunA17 Chr6g0457421
Phaseolus vulgaris Phvul.004G064600.1
Glycine max glyma.Wm82.gnm4.ann1.Glyma.13G046400.1
Lotus japonicus Lj1g3v3317470.1
Medicago truncatula MtrunA17 Chr7g0215181
Phaseolus vulgaris Phvul.008G005600.1
Glycine max glyma.Wm82.gnm4.ann1.Glyma.18G297200.1
Glycine max glyma.Wm82.gnm4.ann1.Glyma.08G365000.1

OG0007557:Targeting protein for Xklp2(TPX2) like protein

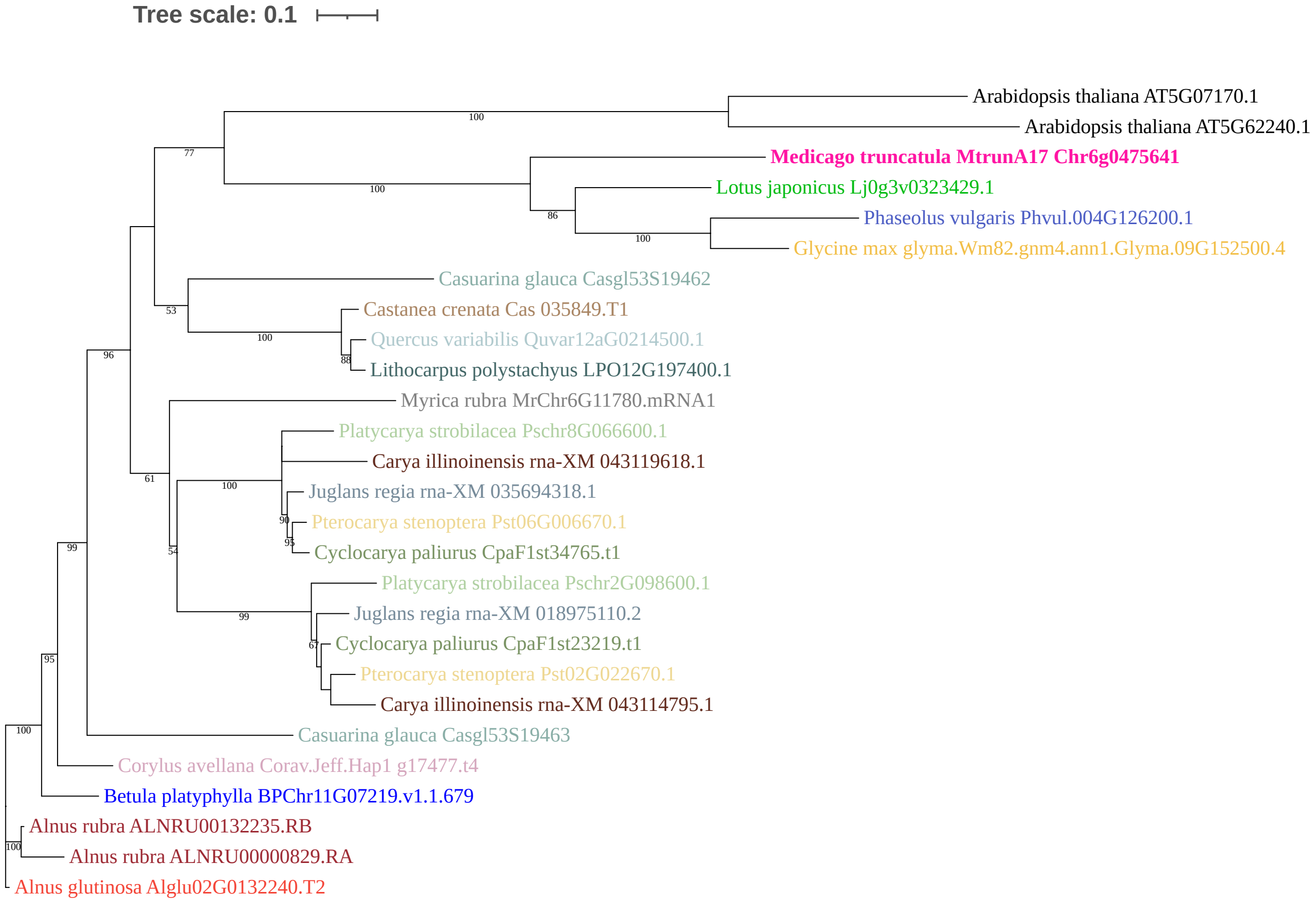

OG0007607:SYMRK INTERACTING PROTEIN 2

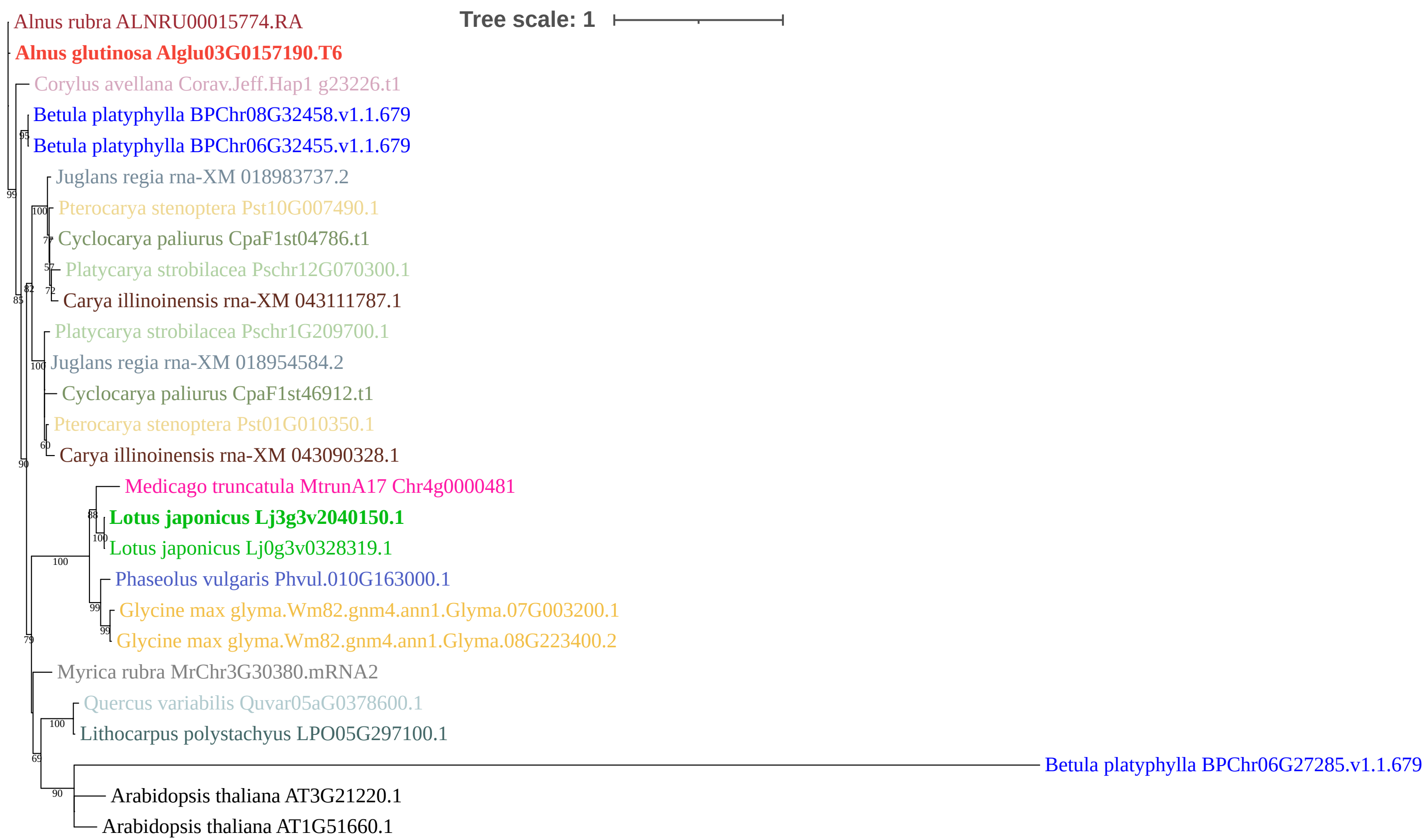

OG0007695:glycinamide ribonucleotide (GAR) transformylase

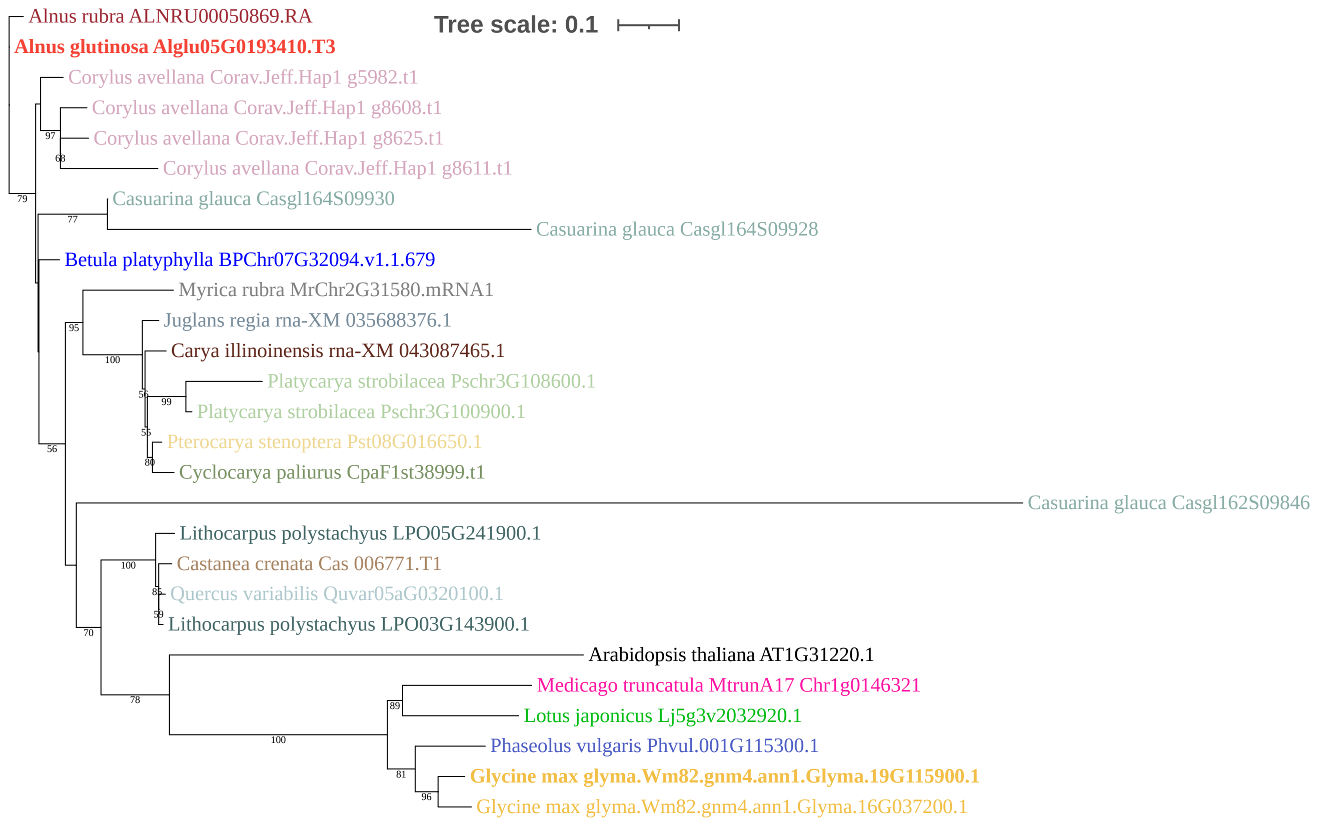

OG0007795:Aurora kinase 1

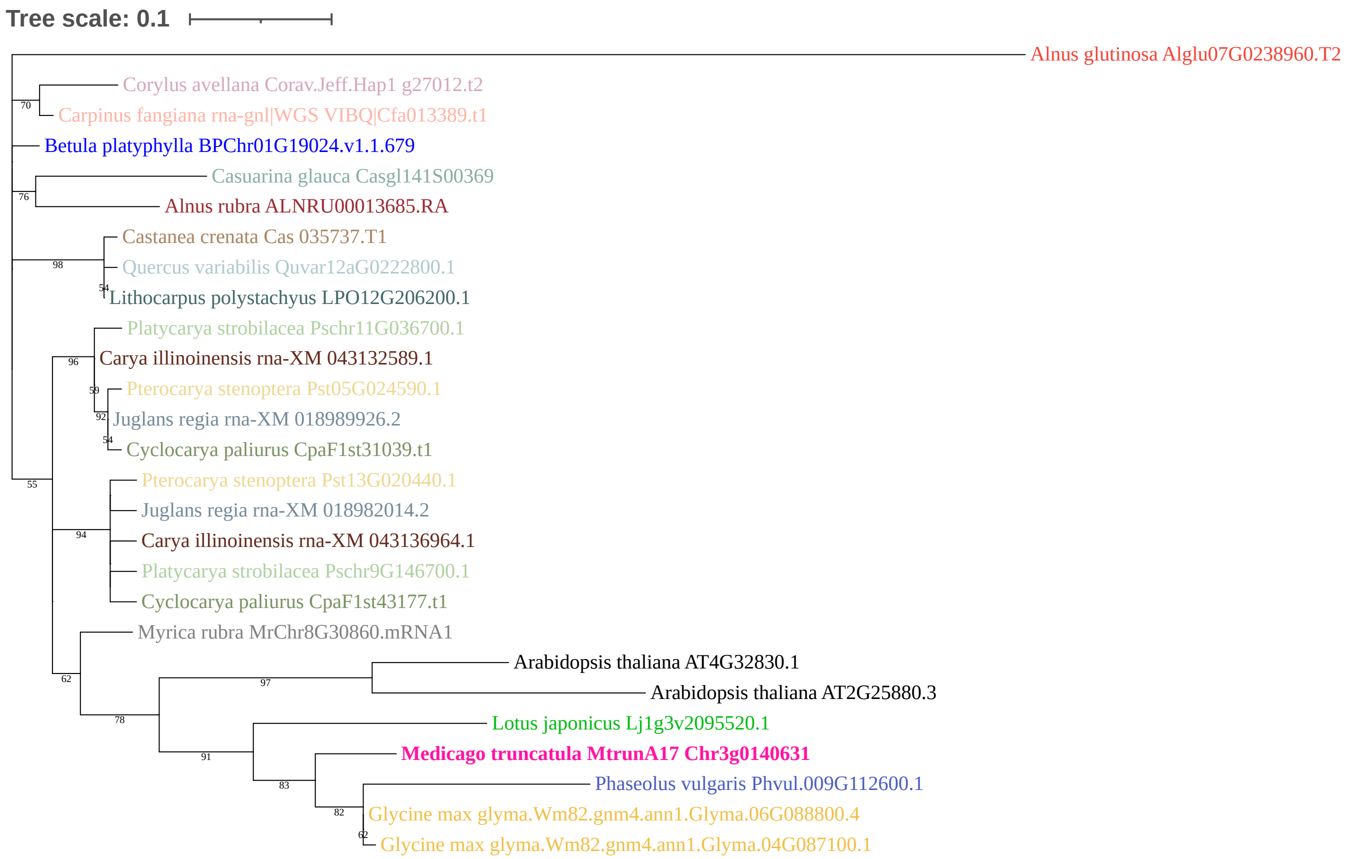

OG0008013:MAP kinase

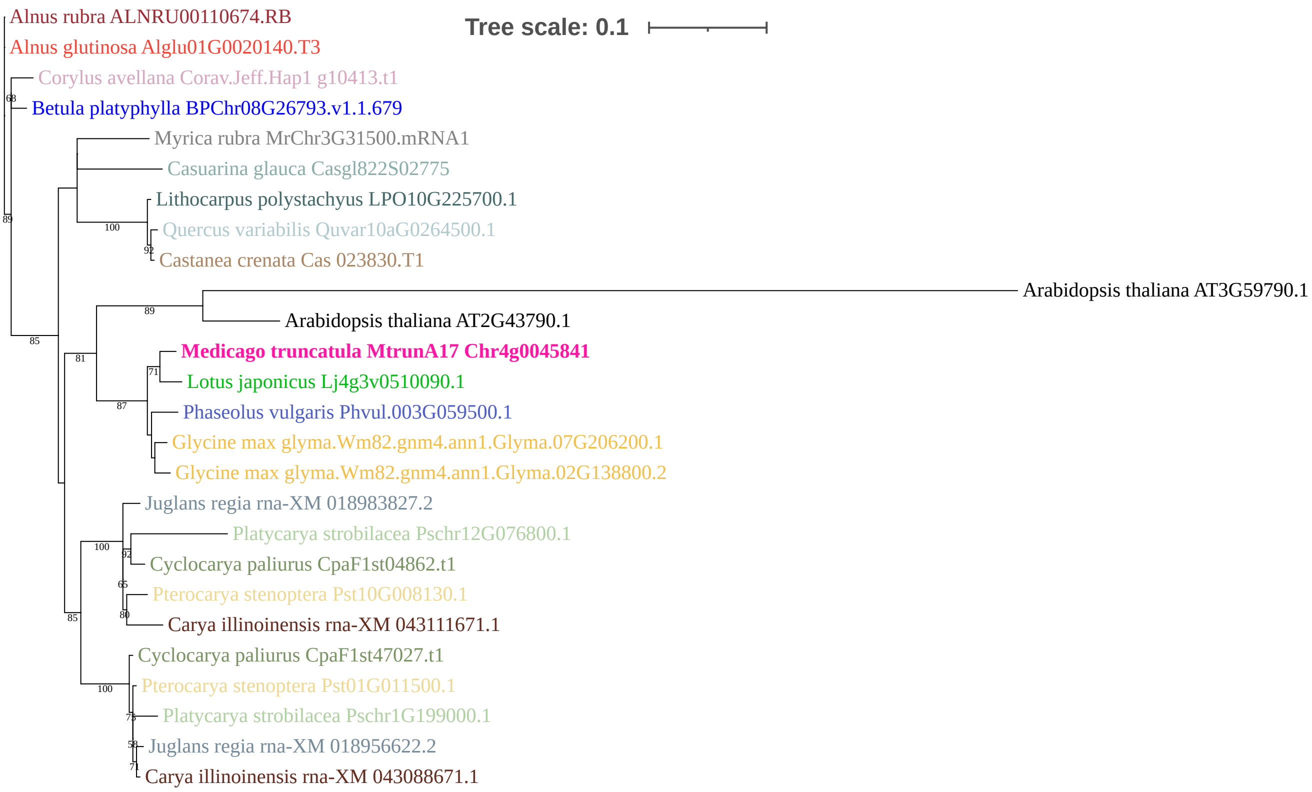

OG0008081:plethora 1/2

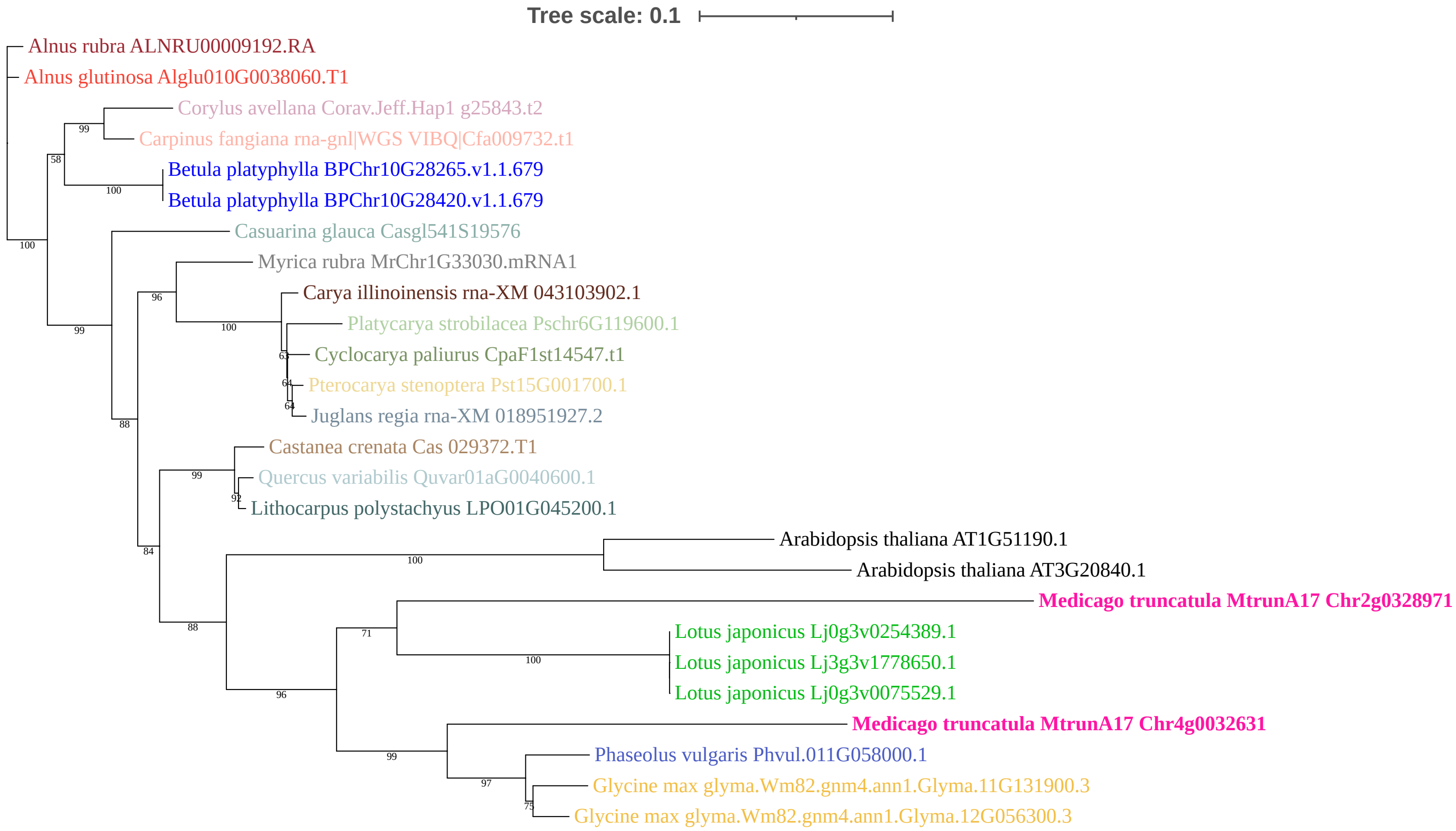

OG0008118:LACK OF SYMBIONT ACCOMMODATION

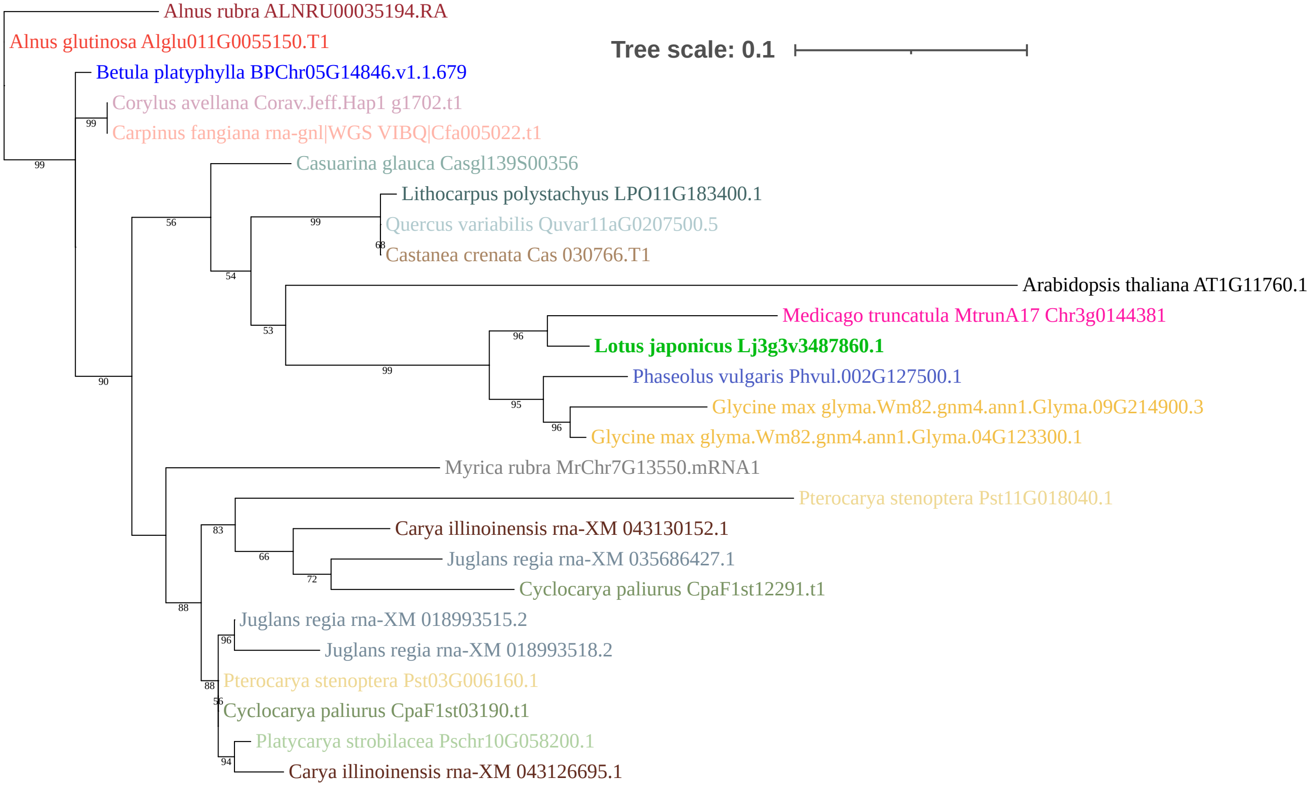

OG0008203:small guanosine triphosphatase (GTPase) 9a/9b/Rho-related GTPase 9

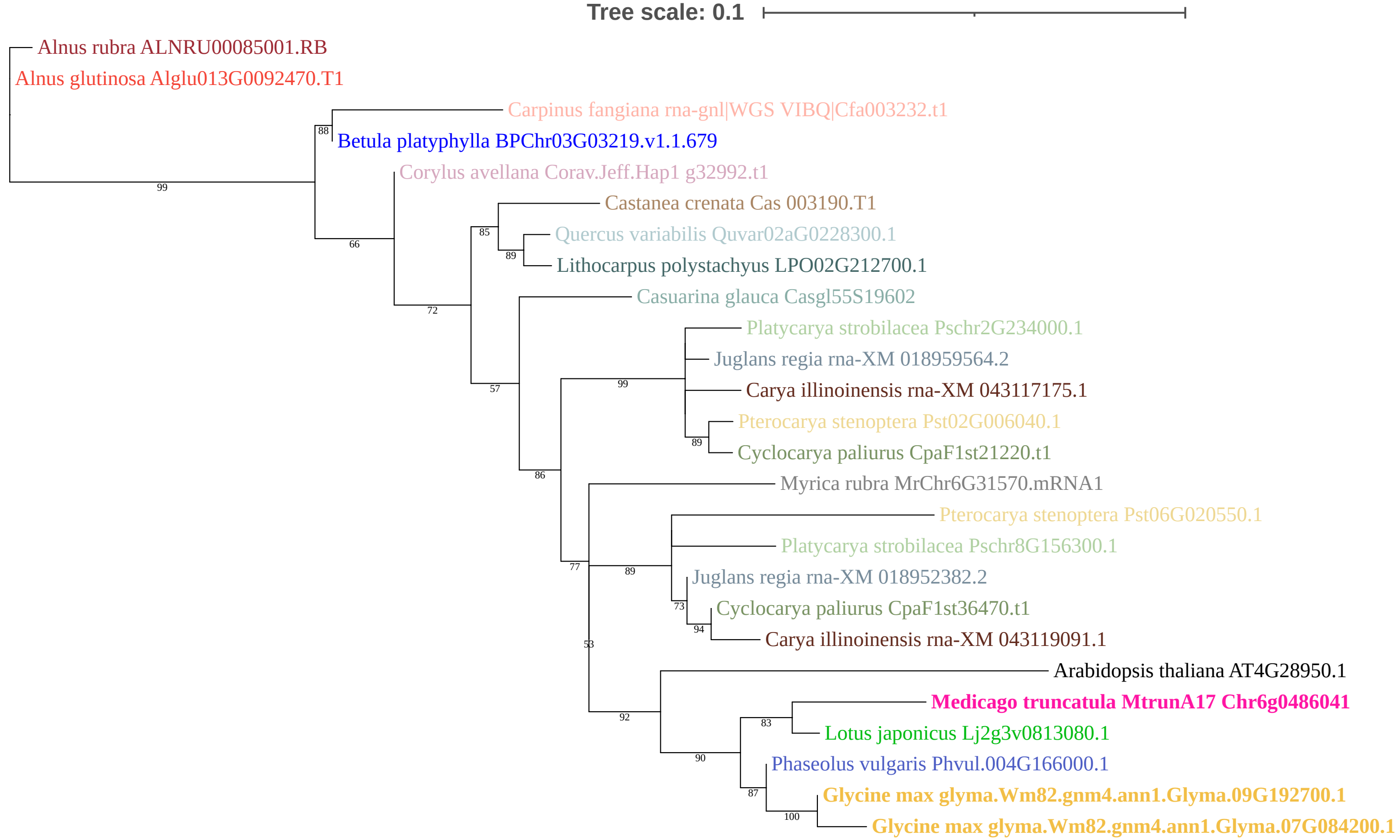

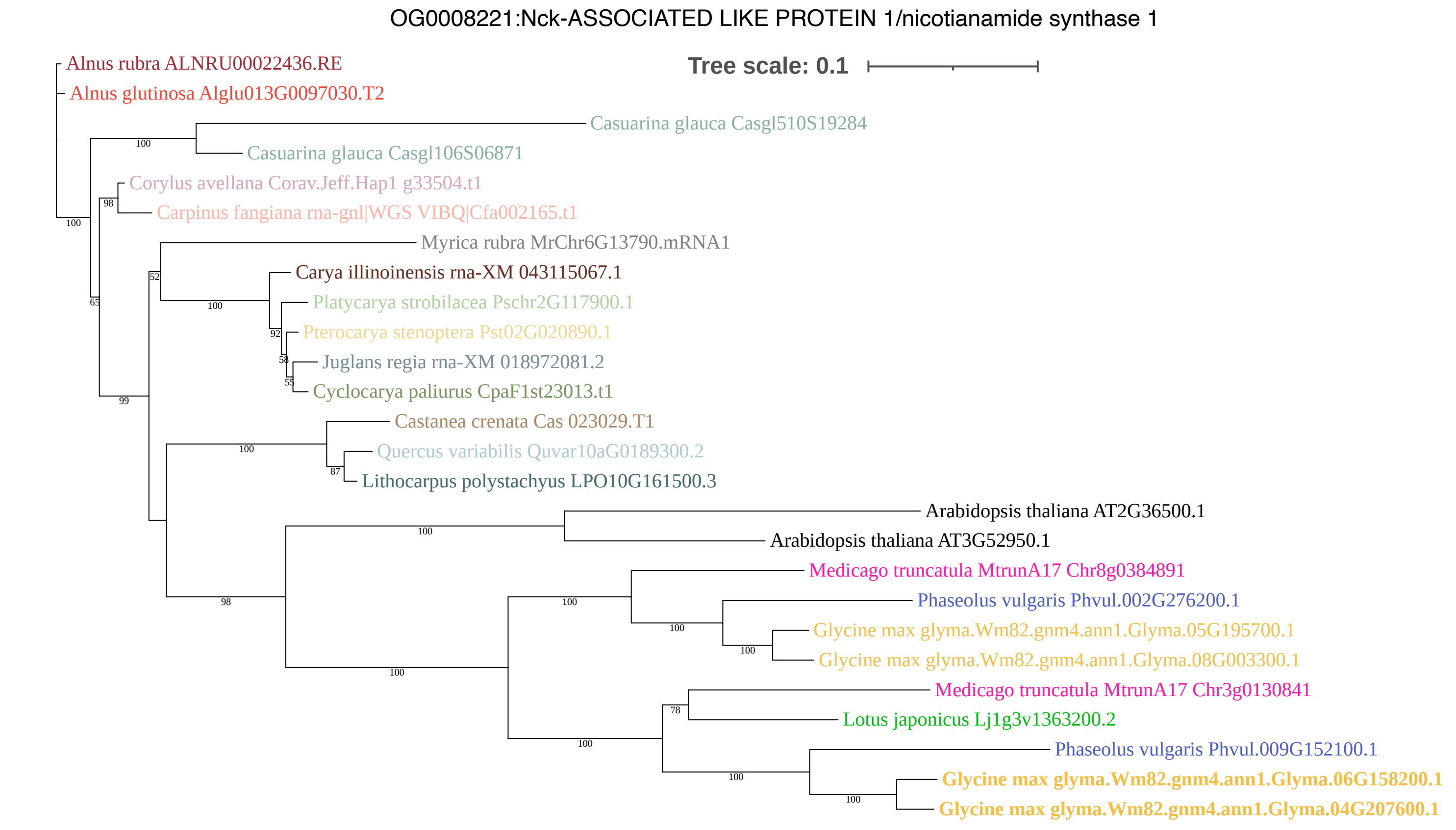
OG0008221:Nck-ASSOCIATED LIKE PROTEIN 1/nicotianamide synthase 1
Tree scale: 0.1
Alnus rubra ALNRU00022436.RE
Alnus glutinosa Alglu013G0097030.T2
Casuarina glauca Casgl510S19284
Casuarina glauca Casgl106S06871
Corylus avellana Corav.Jeff.Hap1 g33504.t1
Carpinus fangiana rna-gnl|WGS VIBQ|Cfa002165.t1
Myrica rubra MrChr6G13790.mRNA1
Carya illinoinensis rna-XM 043115067.1
Platycarya strobilacea Pschr2G117900.1
Pterocarya stenoptera Pst02G020890.1
Juglans regia rna-XM 018972081.2
Cyclocarya paliurus CpaF1st23013.t1
Castanea crenata Cas 023029.T1
Quercus variabilis Quvar10aG0189300.2
Lithocarpus polystachyus LPO10G161500.3
Arabidopsis thaliana AT2G36500.1
Arabidopsis thaliana AT3G52950.1
Medicago truncatula MtrunA17 Chr8g0384891
Phaseolus vulgaris Phvul.002G276200.1
Glycine max glyma.Wm82.gnm4.ann1.Glyma.05G195700.1
Glycine max glyma.Wm82.gnm4.ann1.Glyma.08G003300.1
Medicago truncatula MtrunA17 Chr3g0130841
Lotus japonicus Lj1g3v1363200.2
Phaseolus vulgaris Phvul.009G152100.1
Glycine max glyma.Wm82.gnm4.ann1.Glyma.06G158200.1
Glycine max glyma.Wm82.gnm4.ann1.Glyma.04G207600.1
100
100
98
65
52
100
92
58
55
99
100
87
100
98
100
100
100
100
100
78
100
100

OG0008271:HSC/HSP70 INTERACTING PROTEIN

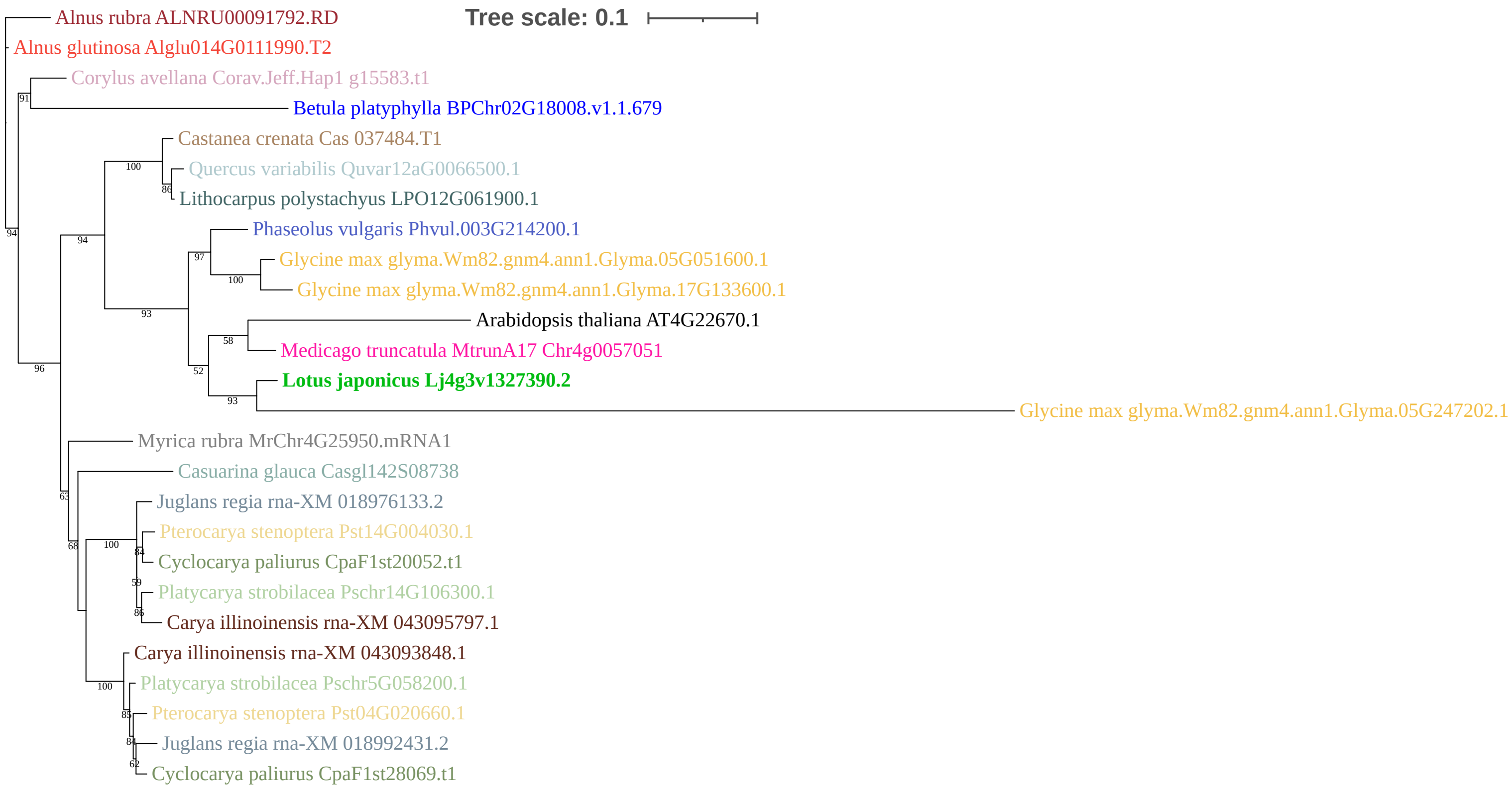

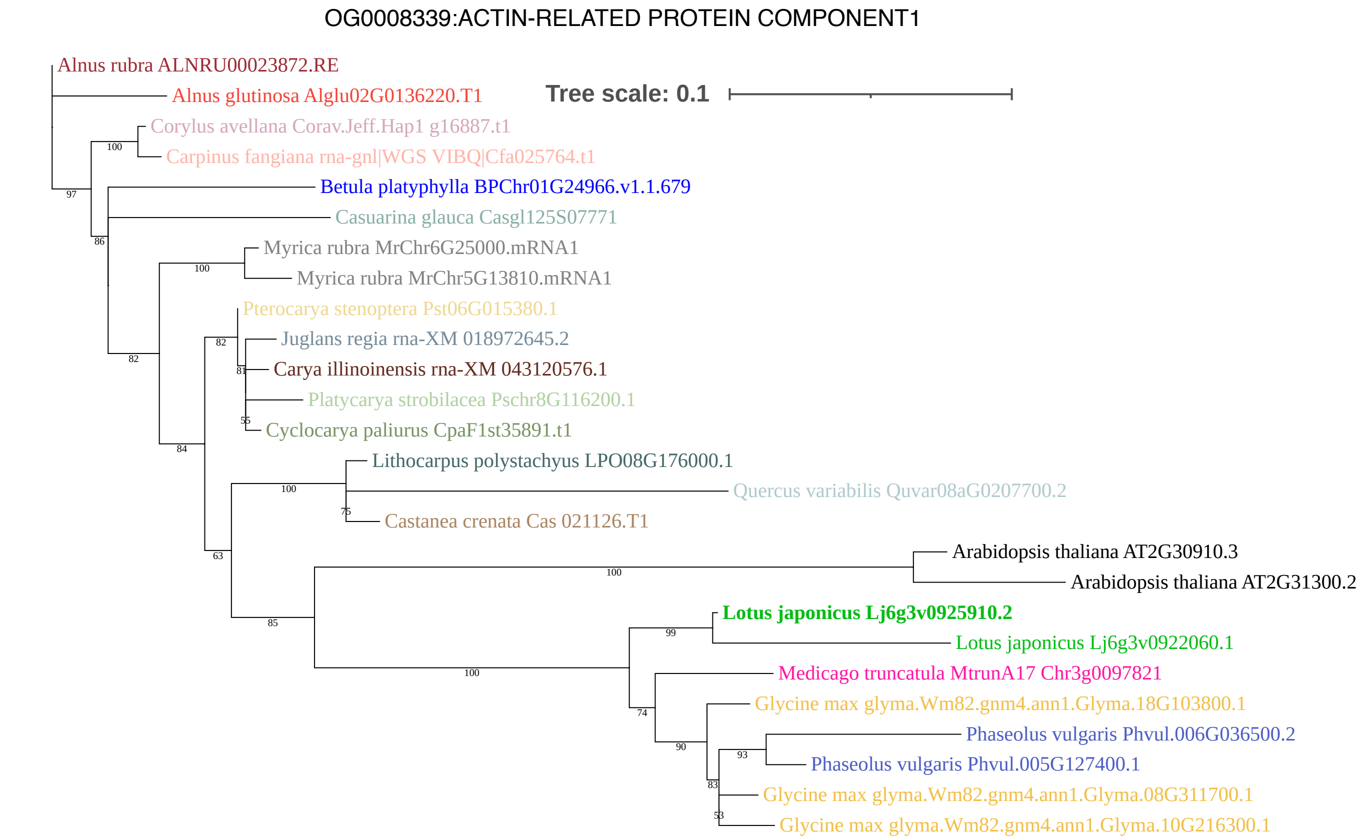
OG0008339:ACTIN-RELATED PROTEIN COMPONENT1
Tree scale: 0.1
Alnus rubra ALNRU00023872.RE
Alnus glutinosa Alglu02G0136220.T1
Corylus avellana Corav.Jeff.Hap1 g16887.t1
Carpinus fangiana rna-gnl|WGS VIBQ|Cfa025764.t1
Betula platyphylla BPChr01G24966.v1.1.679
Casuarina glauca Casgl125S07771
Myrica rubra MrChr6G25000.mRNA1
Myrica rubra MrChr5G13810.mRNA1
Pterocarya stenoptera Pst06G015380.1
Juglans regia rna-XM 018972645.2
Carya illinoinensis rna-XM 043120576.1
Platycarya strobilacea Pschr8G116200.1
Cyclocarya paliurus CpaF1st35891.t1
Lithocarpus polystachyus LPO08G176000.1
Quercus variabilis Quvar08aG0207700.2
Castanea crenata Cas 021126.T1
Arabidopsis thaliana AT2G30910.3
Arabidopsis thaliana AT2G31300.2
Lotus japonicus Lj6g3v0925910.2
Lotus japonicus Lj6g3v0922060.1
Medicago truncatula MtrunA17 Chr3g0097821
Glycine max glyma.Wm82.gnm4.ann1.Glyma.18G103800.1
Phaseolus vulgaris Phvul.006G036500.2
Phaseolus vulgaris Phvul.005G127400.1
Glycine max glyma.Wm82.gnm4.ann1.Glyma.08G311700.1
Glycine max glyma.Wm82.gnm4.ann1.Glyma.10G216300.1
100
97
86
100
82
81
55
82
84
100
75
63
100
85
99
100
74
90
93
83
53

OG0008448:ASSOCIATED MOLECULE WITH THE SH3 DOMAIN OF STAM

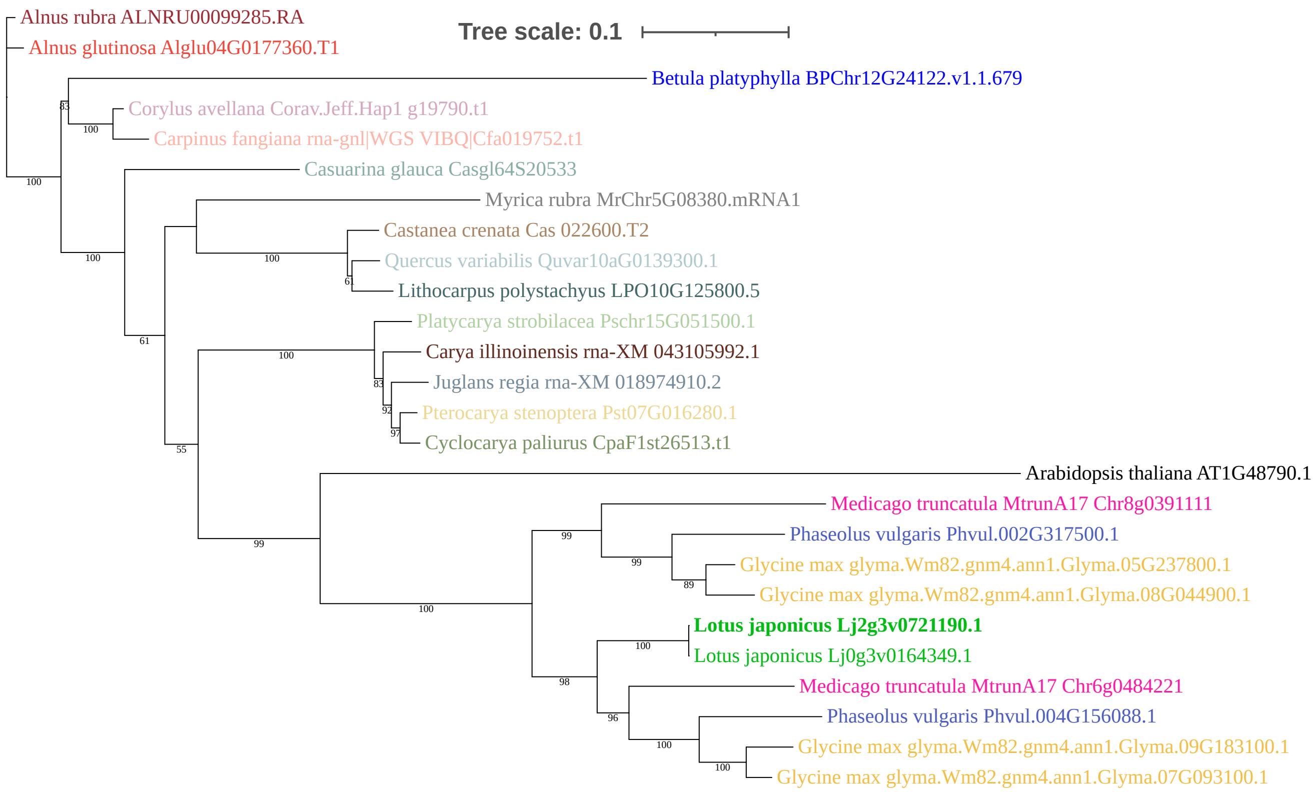

OG0008451:Soybean heterotrimeric G PROTEINS(Ga)/heteromeric G-protein alpha subunit1

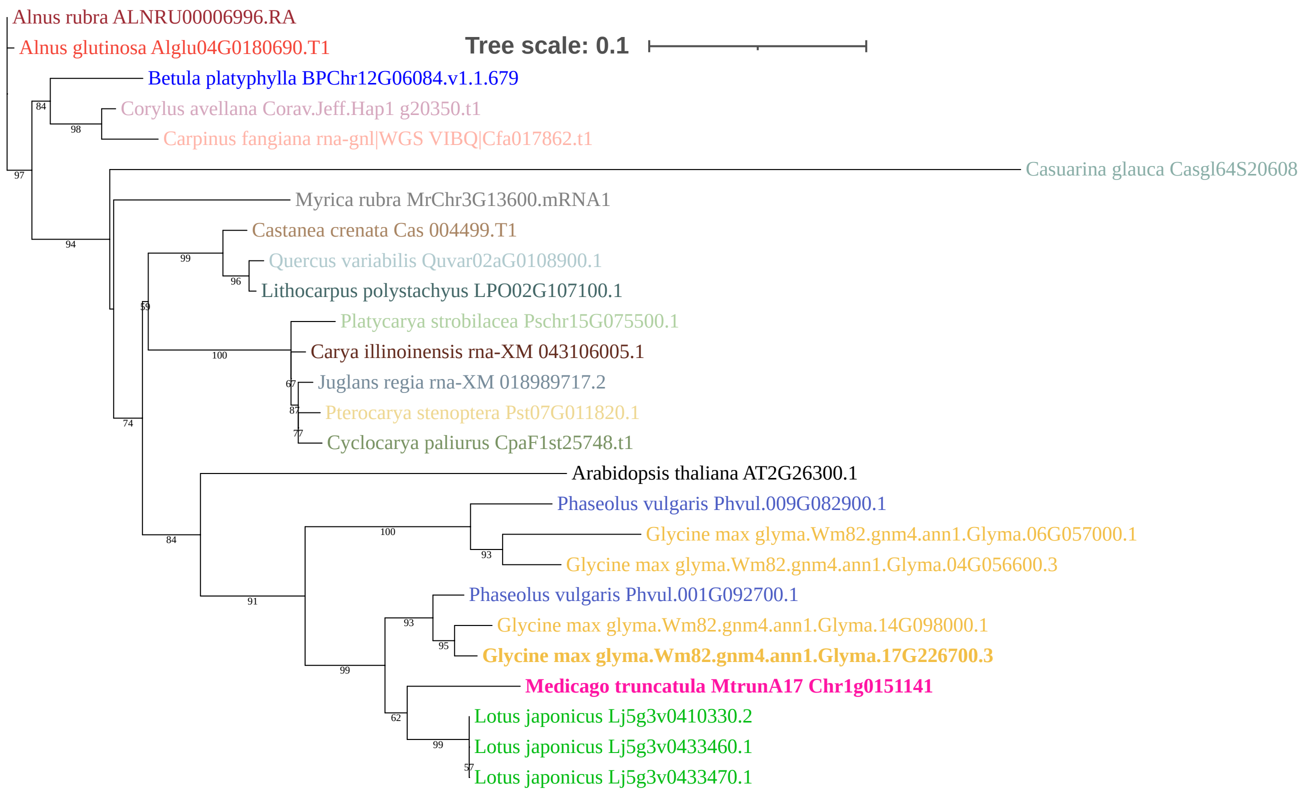

OG0008600:TGACG-motif binding factor 3/1/BASIC LEUCINE ZIPPER FAMILY PROTEIN

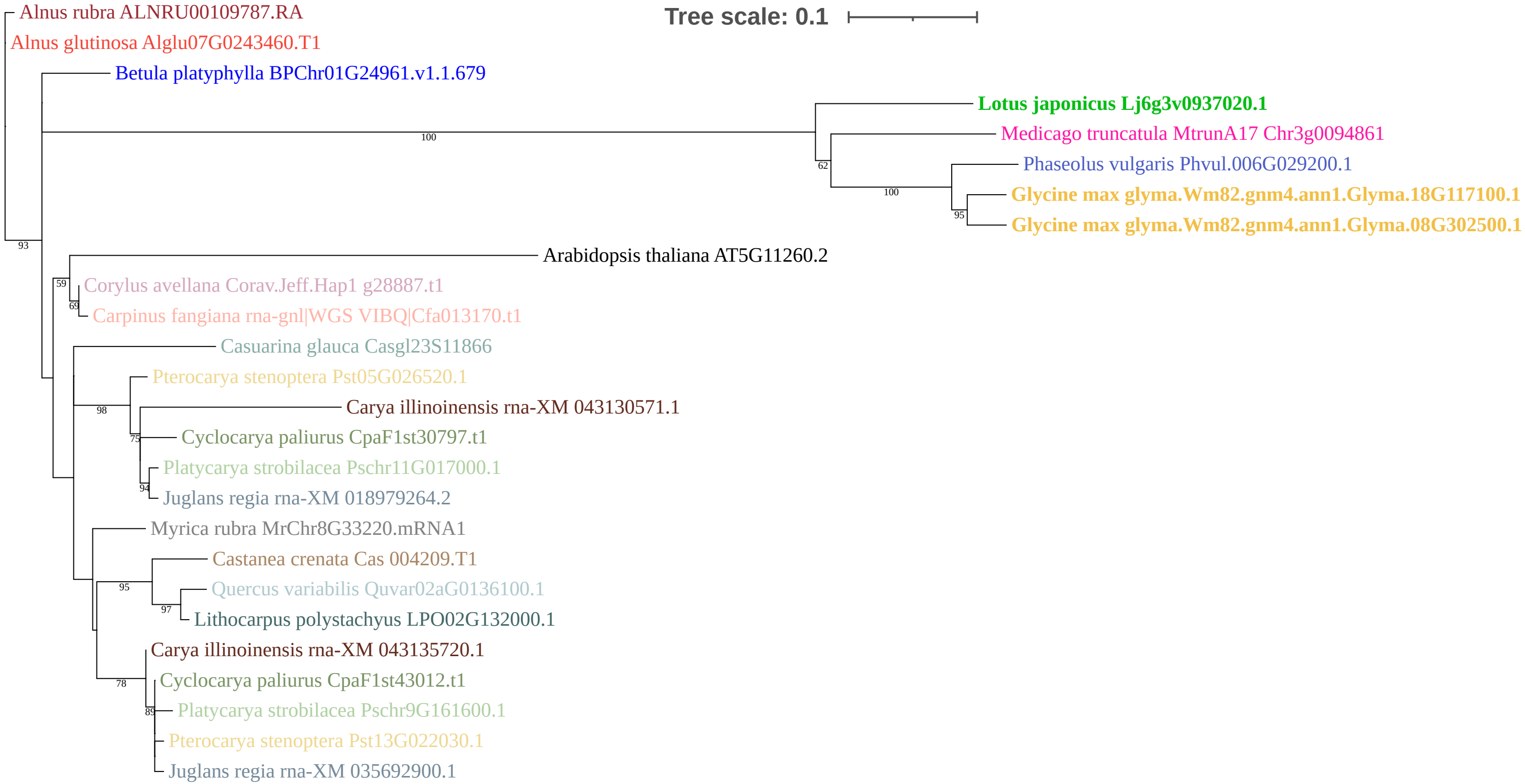

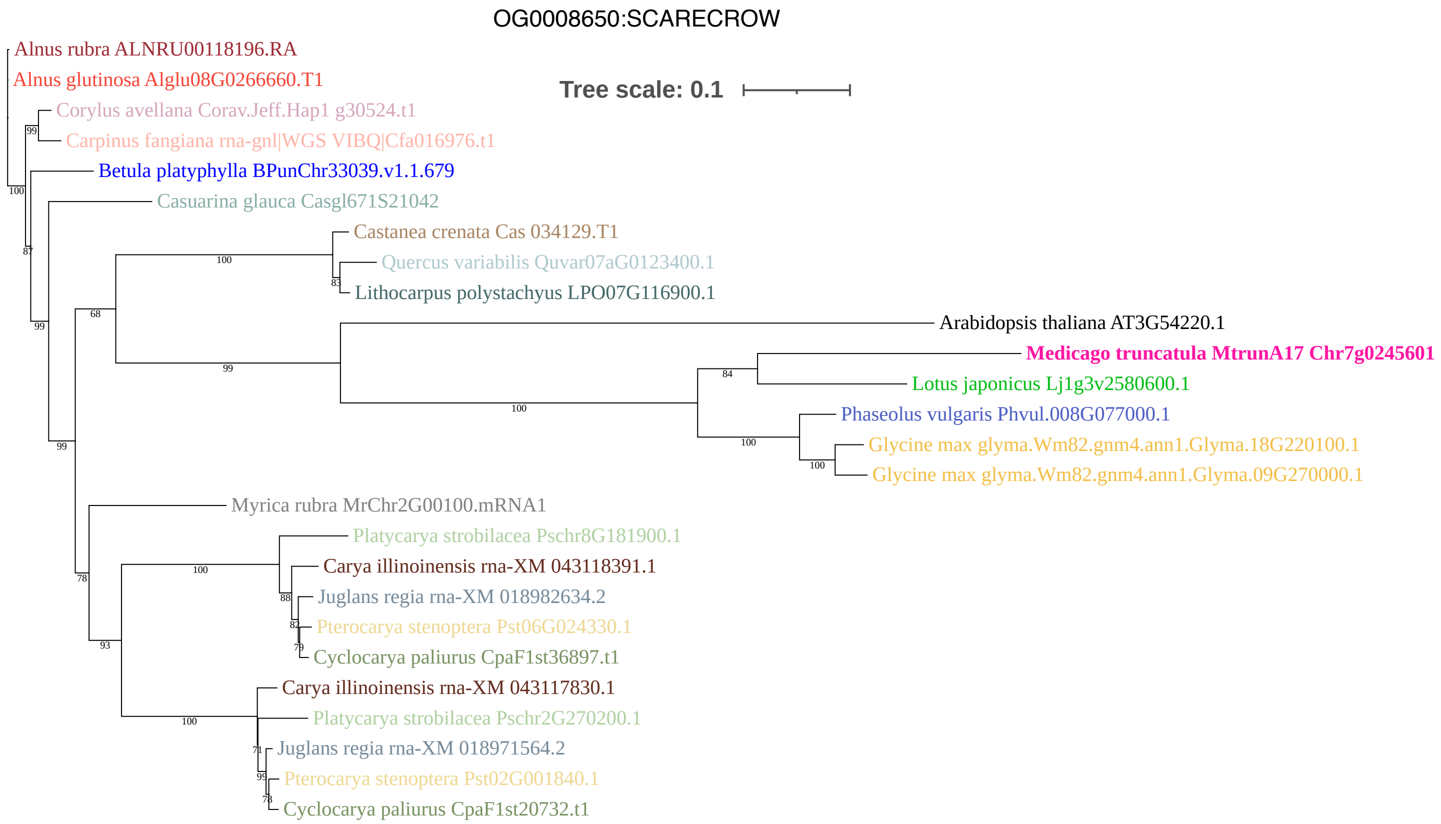

OG0008650:SCARECROW
Tree scale: 0.1
Alnus rubra ALNRU00118196.RA
Alnus glutinosa Alglu08G0266660.T1
Corylus avellana Corav.Jeff.Hap1 g30524.t1
Carpinus fangiana rna-gnl|WGS VIBQ|Cfa016976.t1
Betula platyphylla BPunChr33039.v1.1.679
Casuarina glauca Casgl671S21042
Castanea crenata Cas 034129.T1
Quercus variabilis Quvar07aG0123400.1
Lithocarpus polystachyus LPO07G116900.1
Arabidopsis thaliana AT3G54220.1
Medicago truncatula MtrunA17 Chr7g0245601
Lotus japonicus Lj1g3v2580600.1
Phaseolus vulgaris Phvul.008G077000.1
Glycine max glyma.Wm82.gnm4.ann1.Glyma.18G220100.1
Glycine max glyma.Wm82.gnm4.ann1.Glyma.09G270000.1
Myrica rubra MrChr2G00100.mRNA1
Platycarya strobilacea Pschr8G181900.1
Carya illinoinensis rna-XM 043118391.1
Juglans regia rna-XM 018982634.2
Pterocarya stenoptera Pst06G024330.1
Cyclocarya paliurus CpaF1st36897.t1
Carya illinoinensis rna-XM 043117830.1
Platycarya strobilacea Pschr2G270200.1
Juglans regia rna-XM 018971564.2
Pterocarya stenoptera Pst02G001840.1
Cyclocarya paliurus CpaF1st20732.t1

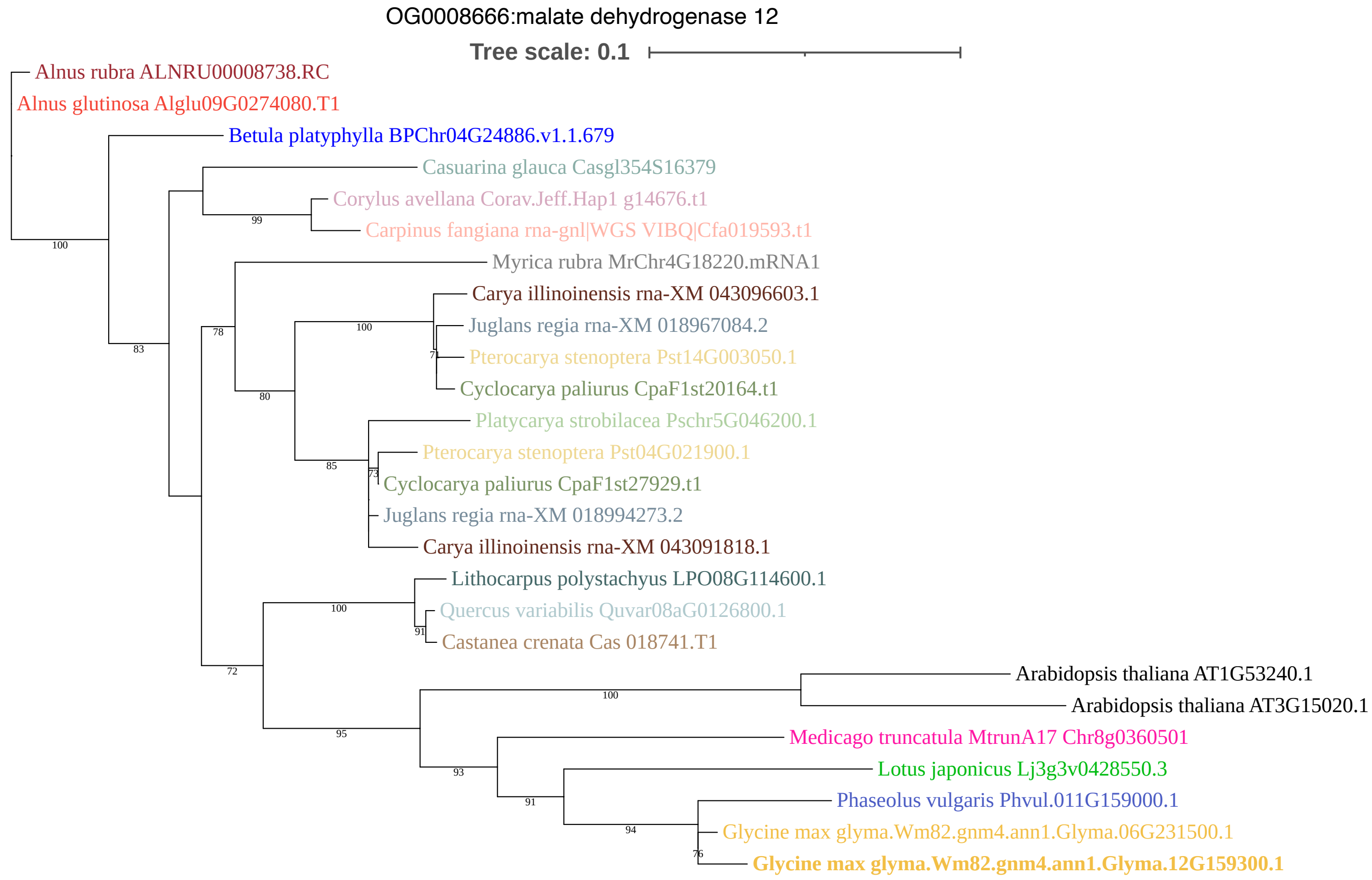
OG0008666:malate dehydrogenase 12
Tree scale: 0.1
Alnus rubra ALNRU00008738.RC
Alnus glutinosa Alglu09G0274080.T1
Betula platyphylla BPChr04G24886.v1.1.679
Casuarina glauca Casgl354S16379
Corylus avellana Corav.Jeff.Hap1 g14676.t1
Carpinus fangiana rna-gnl|WGS VIBQ|Cfa019593.t1
Myrica rubra MrChr4G18220.mRNA1
Carya illinoinensis rna-XM 043096603.1
Juglans regia rna-XM 018967084.2
Pterocarya stenoptera Pst14G003050.1
Cyclocarya paliurus CpaF1st20164.t1
Platycarya strobilacea Pschr5G046200.1
Pterocarya stenoptera Pst04G021900.1
Cyclocarya paliurus CpaF1st27929.t1
Juglans regia rna-XM 018994273.2
Carya illinoinensis rna-XM 043091818.1
Lithocarpus polystachyus LPO08G114600.1
Quercus variabilis Quvar08aG0126800.1
Castanea crenata Cas 018741.T1
Arabidopsis thaliana AT1G53240.1
Arabidopsis thaliana AT3G15020.1
Medicago truncatula MtrunA17 Chr8g0360501
Lotus japonicus Lj3g3v0428550.3
Phaseolus vulgaris Phvul.011G159000.1
Glycine max glyma.Wm82.gnm4.ann1.Glyma.06G231500.1
Glycine max glyma.Wm82.gnm4.ann1.Glyma.12G159300.1
100
99
83
78
100
71
80
85
73
100
91
72
100
95
93
91
94
76

OG0008679:a DOCK family guanine nucleotide exchange factor

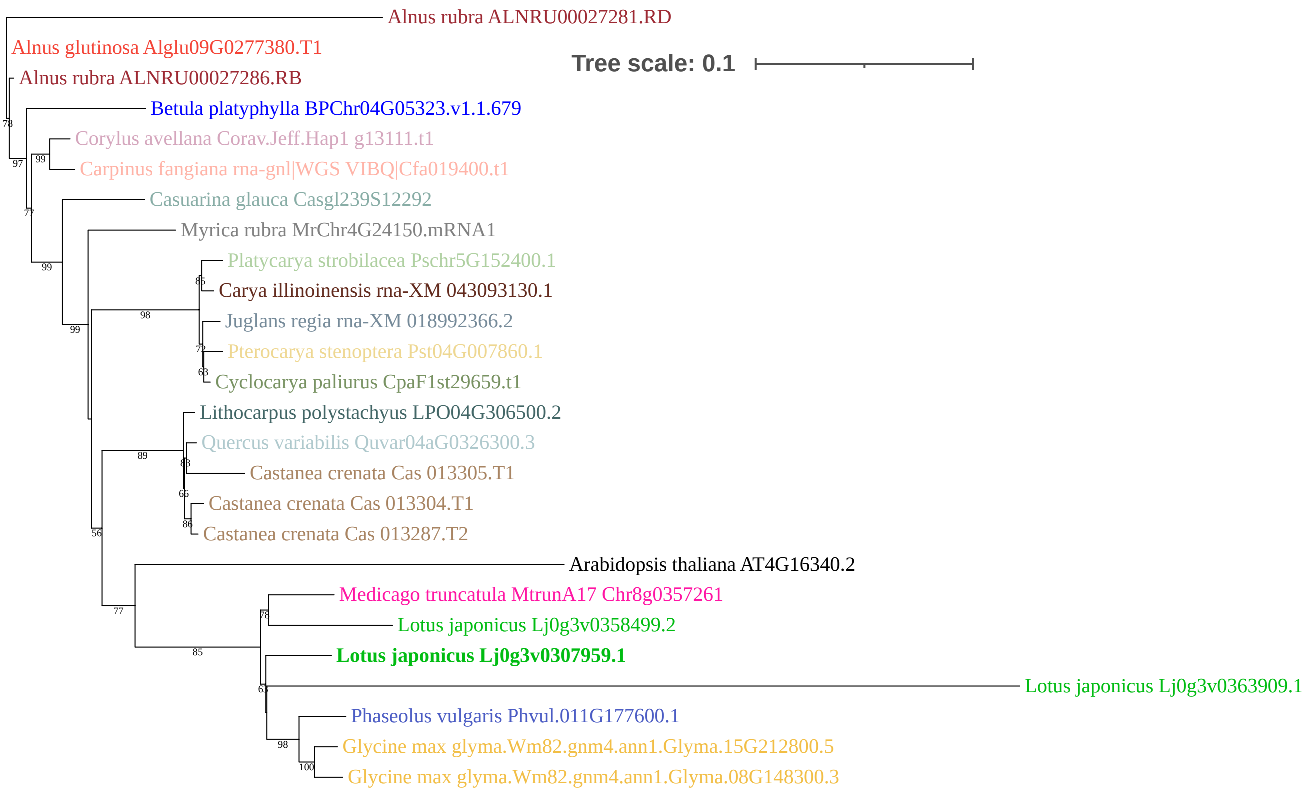

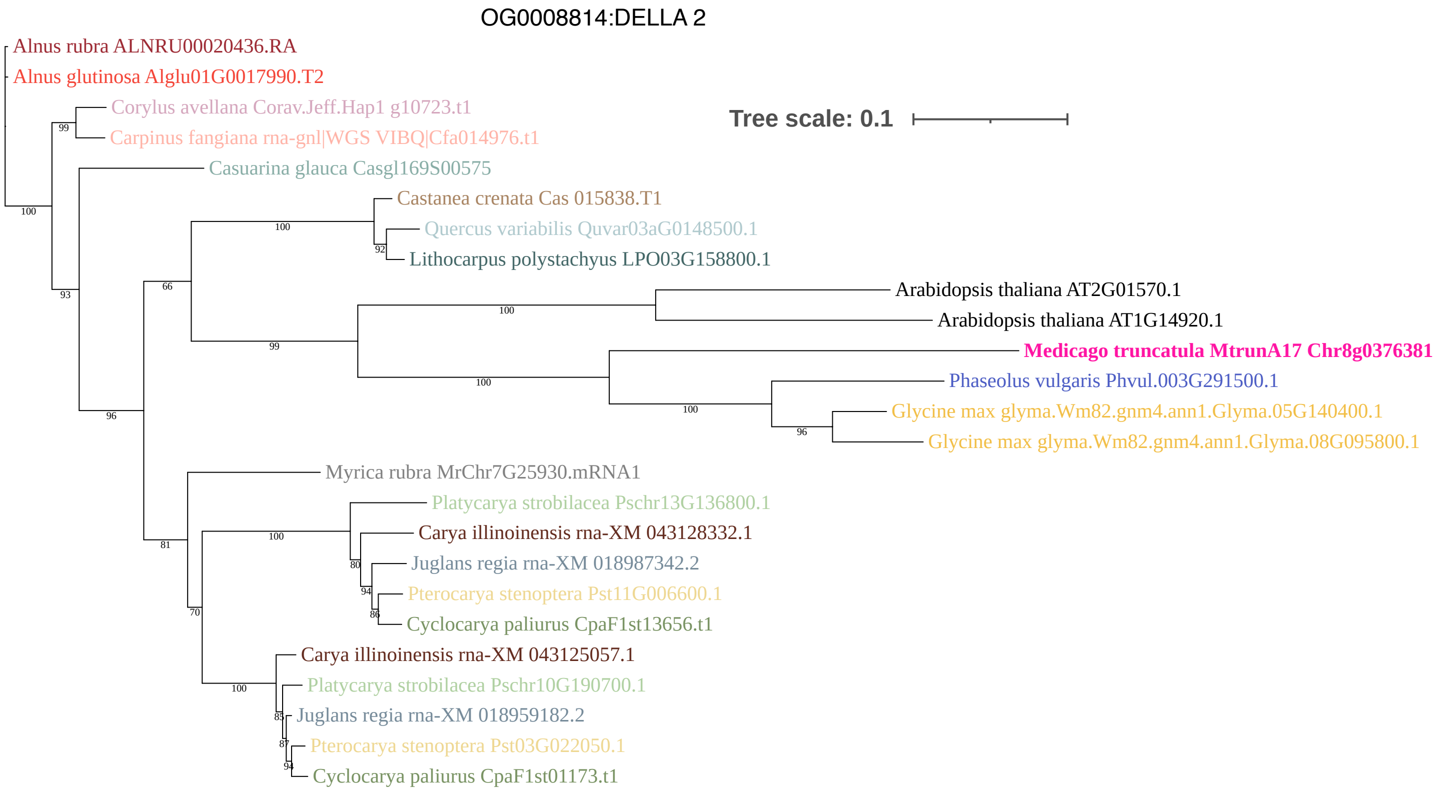
OG0008814:DELLA 2
Tree scale: 0.1
Alnus rubra ALNRU00020436.RA
Alnus glutinosa Alglu01G0017990.T2
Corylus avellana Corav.Jeff.Hap1 g10723.t1
Carpinus fangiana rna-gnl|WGS VIBQ|Cfa014976.t1
Casuarina glauca Casgl169S00575
Castanea crenata Cas 015838.T1
Quercus variabilis Quvar03aG0148500.1
Lithocarpus polystachyus LPO03G158800.1
Arabidopsis thaliana AT2G01570.1
Arabidopsis thaliana AT1G14920.1
Medicago truncatula MtrunA17 Chr8g0376381
Phaseolus vulgaris Phvul.003G291500.1
Glycine max glyma.Wm82.gnm4.ann1.Glyma.05G140400.1
Glycine max glyma.Wm82.gnm4.ann1.Glyma.08G095800.1
Myrica rubra MrChr7G25930.mRNA1
Platycarya strobilacea Pschr13G136800.1
Carya illinoinensis rna-XM 043128332.1
Juglans regia rna-XM 018987342.2
Pterocarya stenoptera Pst11G006600.1
Cyclocarya paliurus CpaF1st13656.t1
Carya illinoinensis rna-XM 043125057.1
Platycarya strobilacea Pschr10G190700.1
Juglans regia rna-XM 018959182.2
Pterocarya stenoptera Pst03G022050.1
Cyclocarya paliurus CpaF1st01173.t1

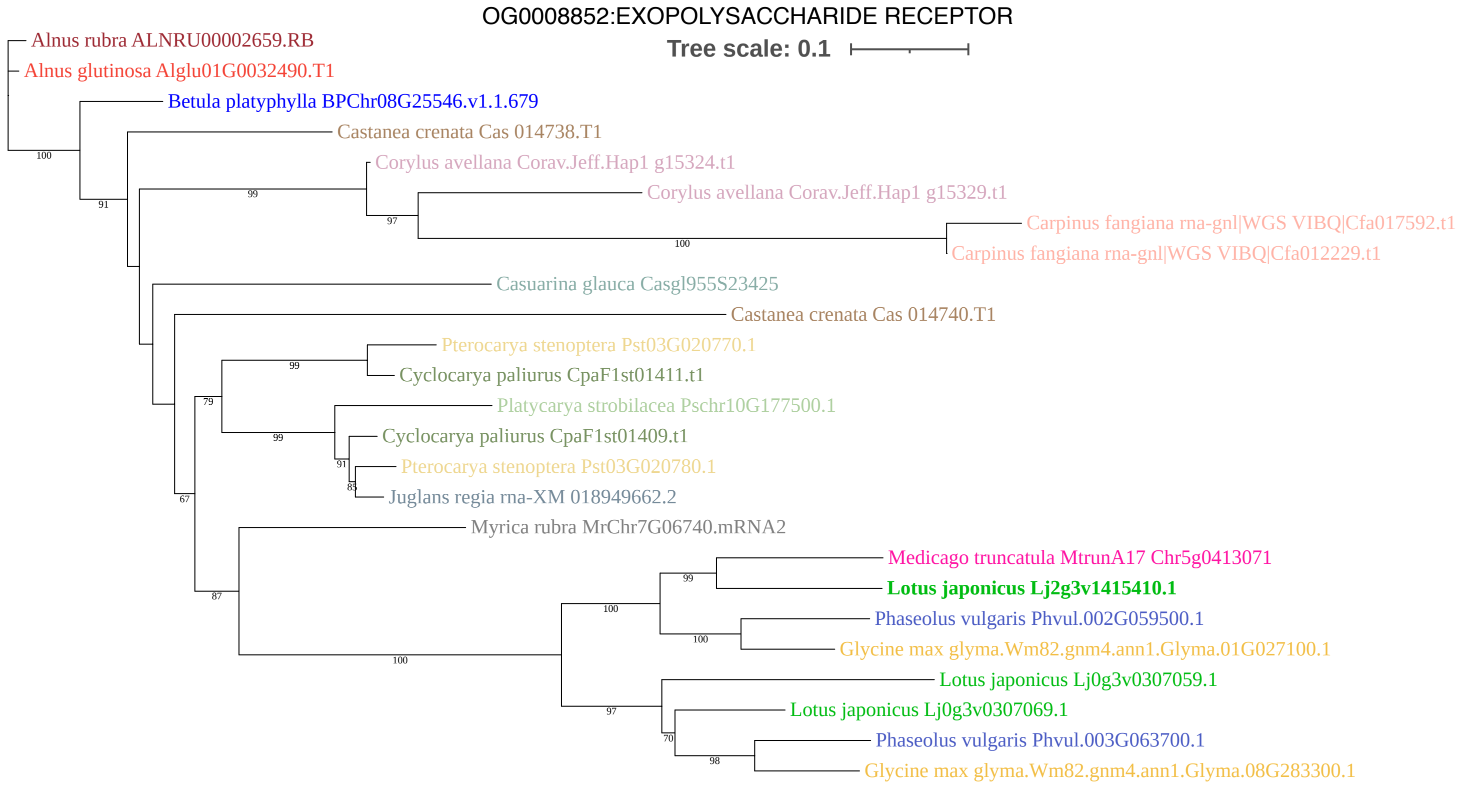
OG0008852:EXOPOLYSACCHARIDE RECEPTOR
Tree scale: 0.1
Alnus rubra ALNRU00002659.RB
Alnus glutinosa Alglu01G0032490.T1
Betula platyphylla BPChr08G25546.v1.1.679
Castanea crenata Cas 014738.T1
Corylus avellana Corav.Jeff.Hap1 g15324.t1
Corylus avellana Corav.Jeff.Hap1 g15329.t1
Carpinus fangiana rna-gnl|WGS VIBQ|Cfa017592.t1
Carpinus fangiana rna-gnl|WGS VIBQ|Cfa012229.t1
Casuarina glauca Casgl955S23425
Castanea crenata Cas 014740.T1
Pterocarya stenoptera Pst03G020770.1
Cyclocarya paliurus CpaF1st01411.t1
Platycarya strobilacea Pschr10G177500.1
Cyclocarya paliurus CpaF1st01409.t1
Pterocarya stenoptera Pst03G020780.1
Juglans regia rna-XM 018949662.2
Myrica rubra MrChr7G06740.mRNA2
Medicago truncatula MtrunA17 Chr5g0413071
Lotus japonicus Lj2g3v1415410.1
Phaseolus vulgaris Phvul.002G059500.1
Glycine max glyma.Wm82.gnm4.ann1.Glyma.01G027100.1
Lotus japonicus Lj0g3v0307059.1
Lotus japonicus Lj0g3v0307069.1
Phaseolus vulgaris Phvul.003G063700.1
Glycine max glyma.Wm82.gnm4.ann1.Glyma.08G283300.1

OG0008967:plethora ERFIBABYBOOM

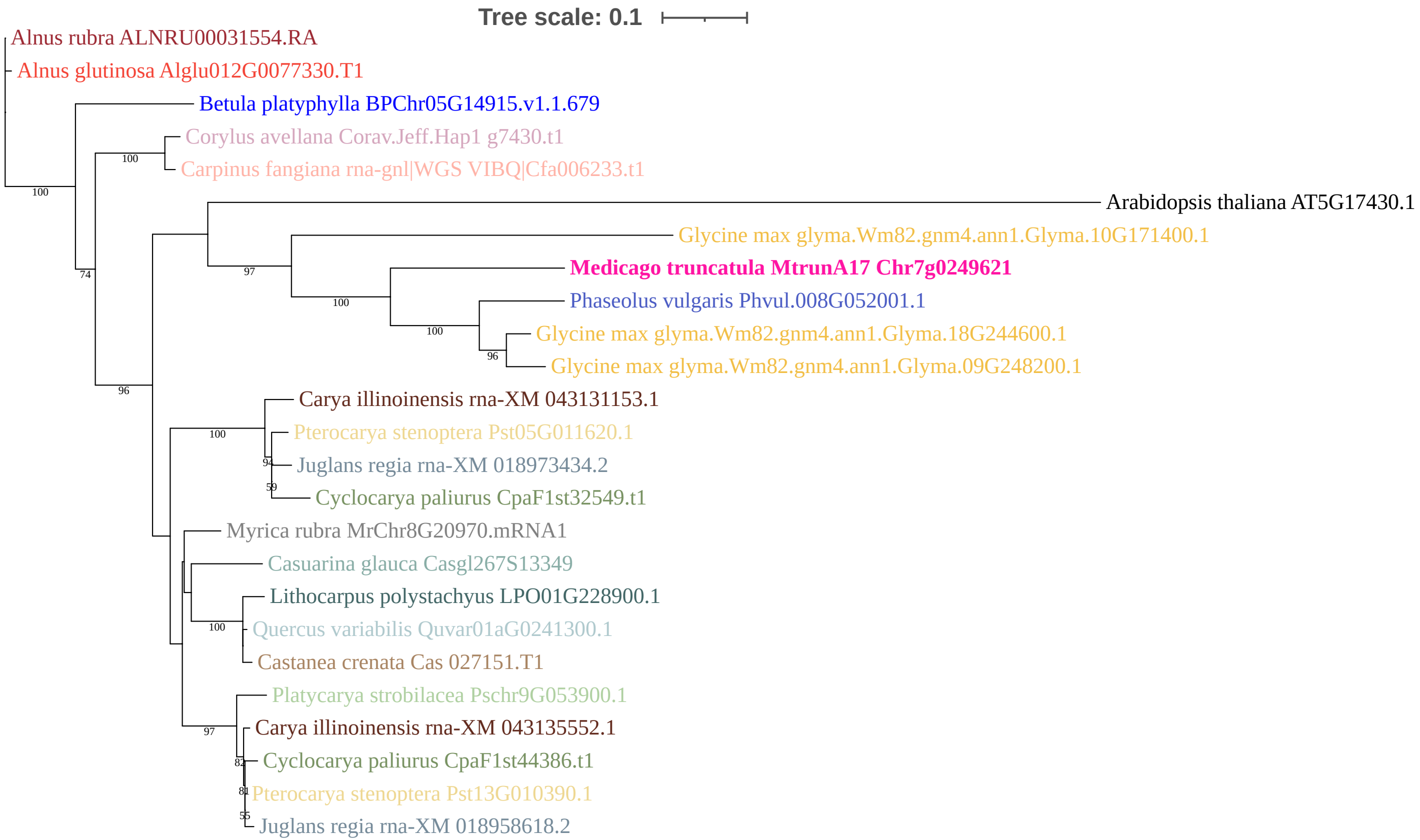

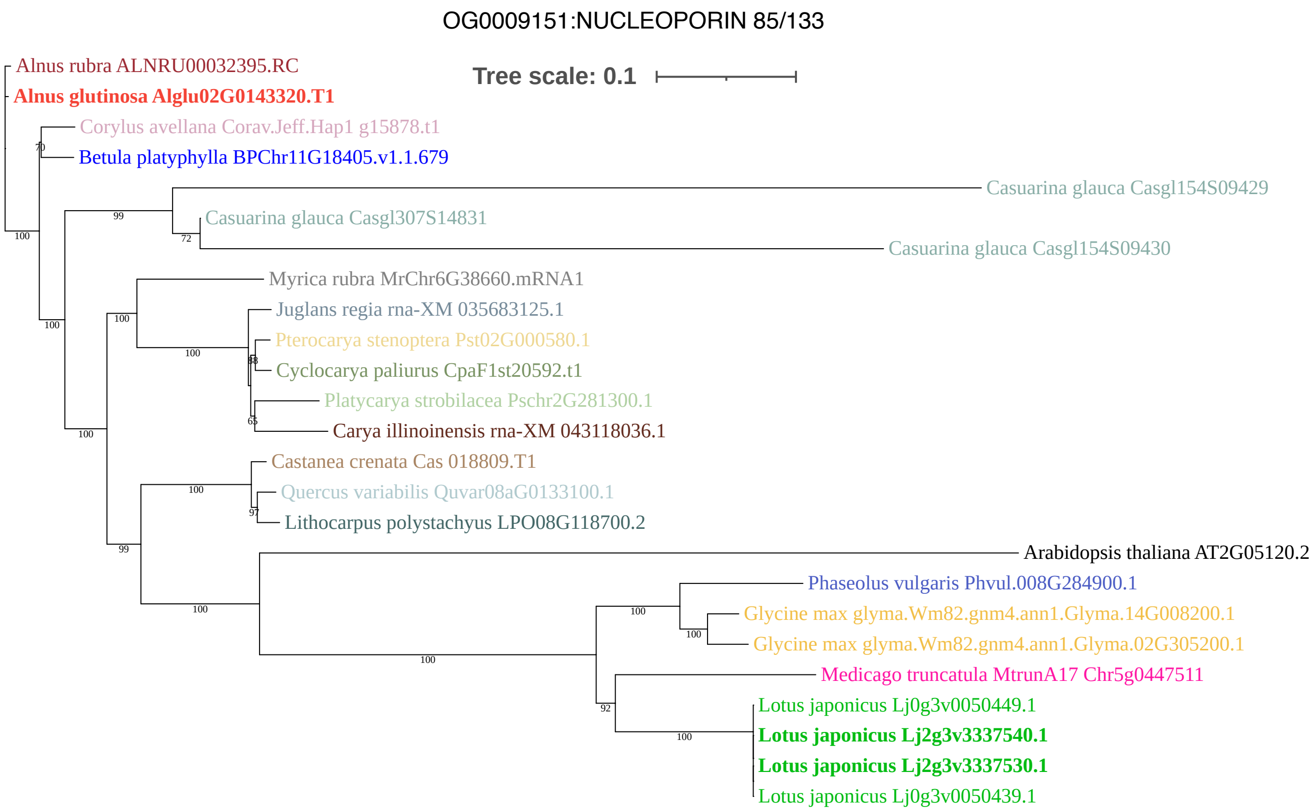
OG0009151:NUCLEOPORIN 85/133
Tree scale: 0.1
Alnus rubra ALNRU00032395.RC
Alnus glutinosa Alglu02G0143320.T1
Corylus avellana Corav.Jeff.Hap1 g15878.t1
Betula platyphylla BPChr11G18405.v1.1.679
Casuarina glauca Casgl154S09429
Casuarina glauca Casgl307S14831
Casuarina glauca Casgl154S09430
Myrica rubra MrChr6G38660.mRNA1
Juglans regia rna-XM 035683125.1
Pterocarya stenoptera Pst02G000580.1
Cyclocarya paliurus CpaF1st20592.t1
Platycarya strobilacea Pschr2G281300.1
Carya illinoinensis rna-XM 043118036.1
Castanea crenata Cas 018809.T1
Quercus variabilis Quvar08aG0133100.1
Lithocarpus polystachyus LPO08G118700.2
Arabidopsis thaliana AT2G05120.2
Phaseolus vulgaris Phvul.008G284900.1
Glycine max glyma.Wm82.gnm4.ann1.Glyma.14G008200.1
Glycine max glyma.Wm82.gnm4.ann1.Glyma.02G305200.1
Medicago truncatula MtrunA17 Chr5g0447511
Lotus japonicus Lj0g3v0050449.1
Lotus japonicus Lj2g3v3337540.1
Lotus japonicus Lj2g3v3337530.1
Lotus japonicus Lj0g3v0050439.1

OG0009172:guanine nucleotide exchange factor 2a/2b/2

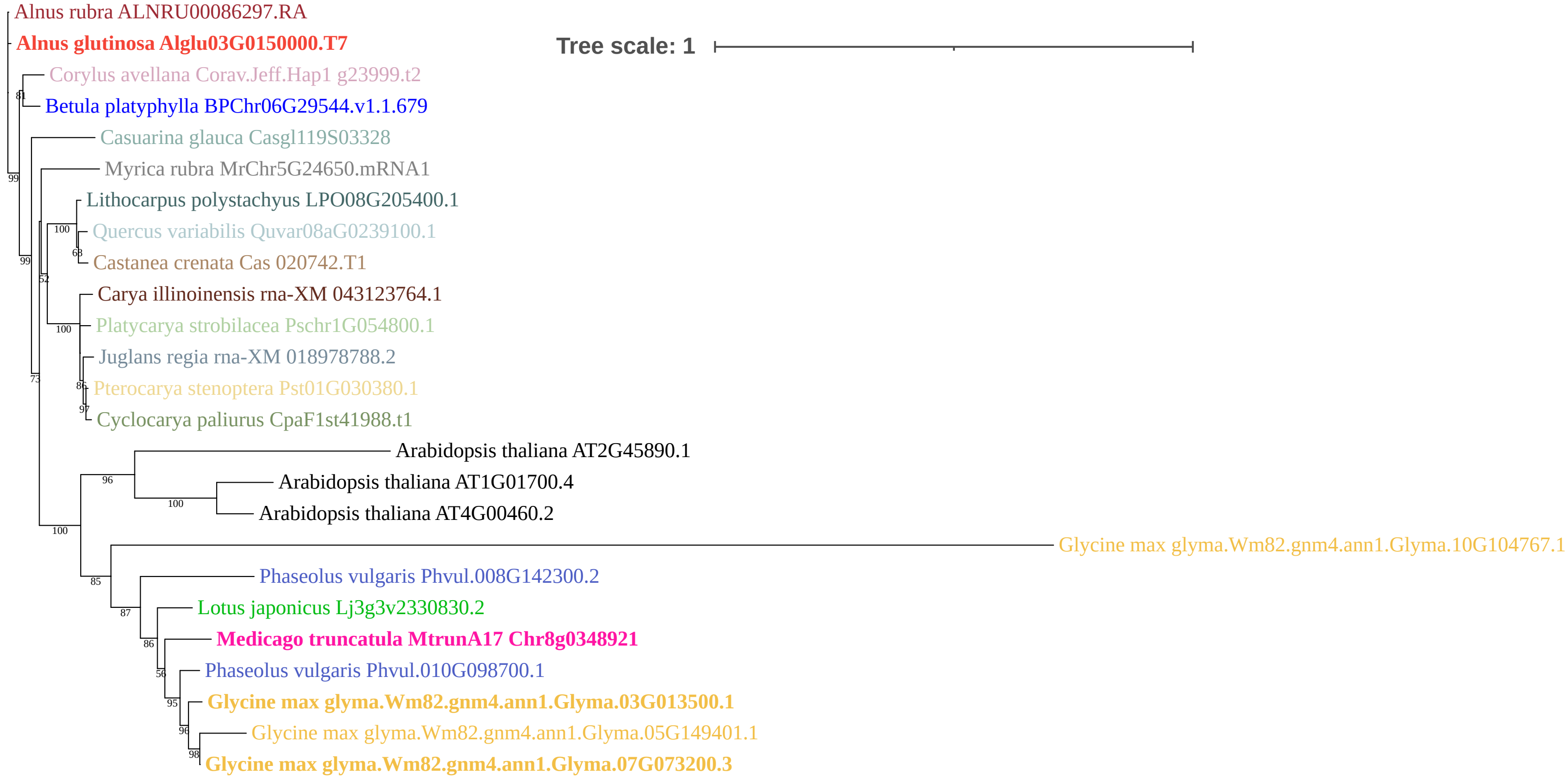

OG0009217:Wuschel-related homeobox transcription factor 5

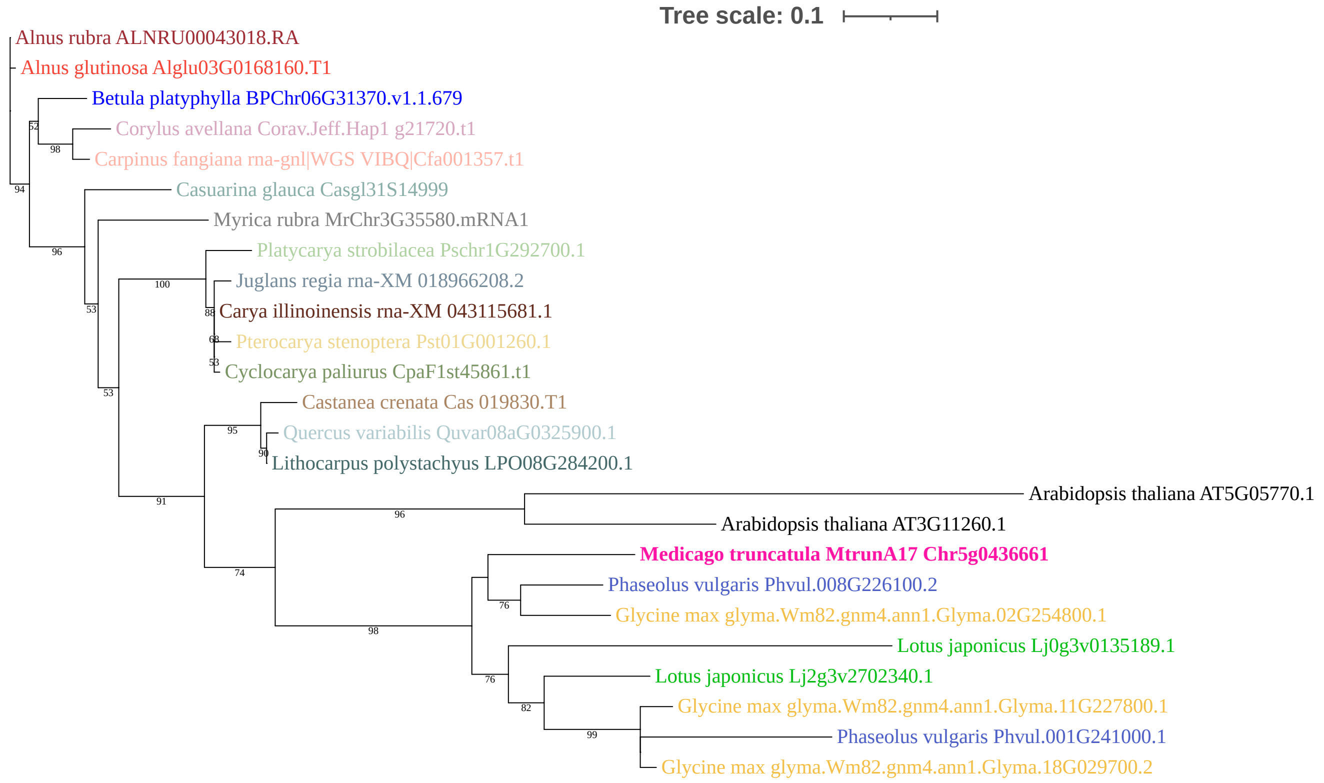

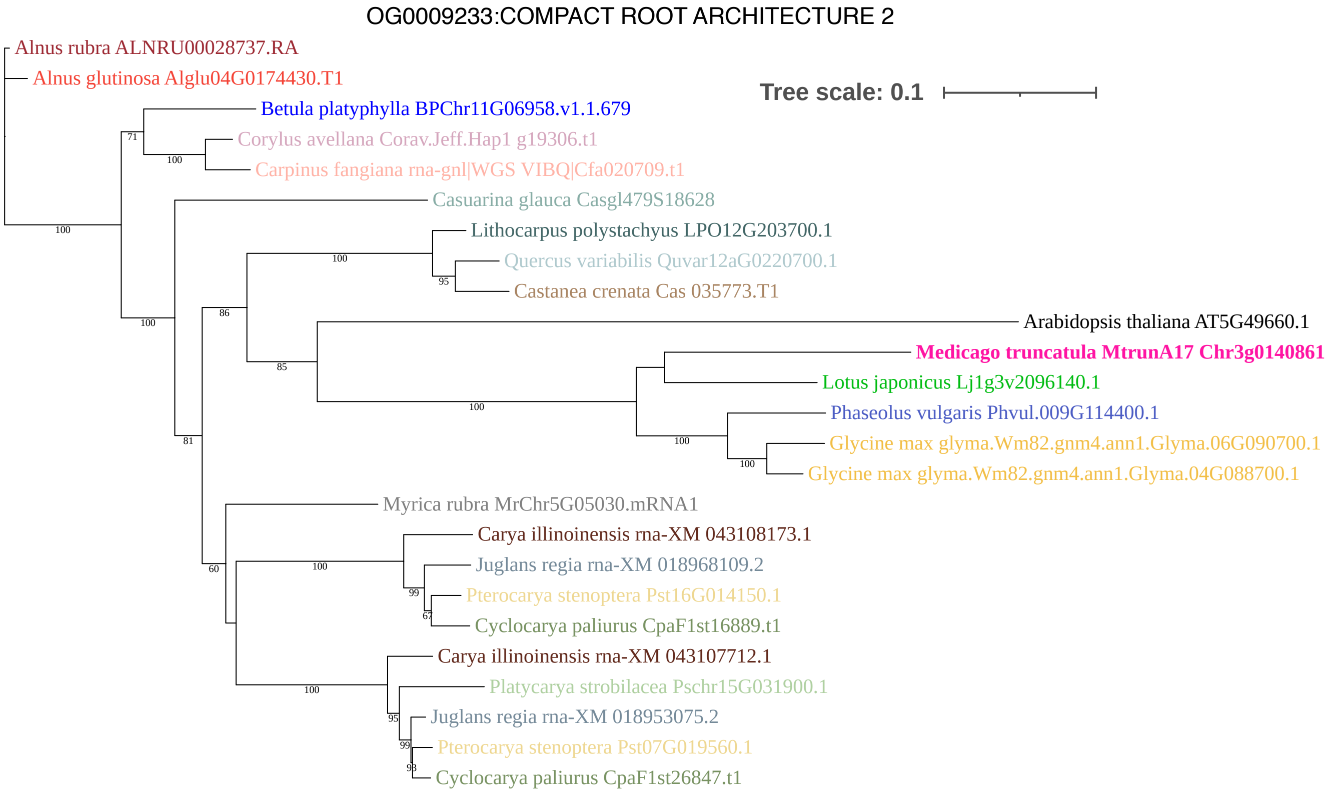
OG0009233:COMPACT ROOT ARCHITECTURE 2
Tree scale: 0.1
Alnus rubra ALNRU00028737.RA
Alnus glutinosa Alglu04G0174430.T1
Betula platyphylla BPChr11G06958.v1.1.679
Corylus avellana Corav.Jeff.Hap1 g19306.t1
Carpinus fangiana rna-gnl|WGS VIBQ|Cfa020709.t1
Casuarina glauca Casgl479S18628
Lithocarpus polystachyus LPO12G203700.1
Quercus variabilis Quvar12aG0220700.1
Castanea crenata Cas 035773.T1
Arabidopsis thaliana AT5G49660.1
Medicago truncatula MtrunA17 Chr3g0140861
Lotus japonicus Lj1g3v2096140.1
Phaseolus vulgaris Phvul.009G114400.1
Glycine max glyma.Wm82.gnm4.ann1.Glyma.06G090700.1
Glycine max glyma.Wm82.gnm4.ann1.Glyma.04G088700.1
Myrica rubra MrChr5G05030.mRNA1
Carya illinoinensis rna-XM 043108173.1
Juglans regia rna-XM 018968109.2
Pterocarya stenoptera Pst16G014150.1
Cyclocarya paliurus CpaF1st16889.t1
Carya illinoinensis rna-XM 043107712.1
Platycarya strobilacea Pschr15G031900.1
Juglans regia rna-XM 018953075.2
Pterocarya stenoptera Pst07G019560.1
Cyclocarya paliurus CpaF1st26847.t1
71
100
100
100
100
95
86
85
100
100
100
81
60
100
99
67
100
95
99
93

OG0009270:STEAROYL-ACYL CARRIER PROTEIN DESATURASE ISOFORM C

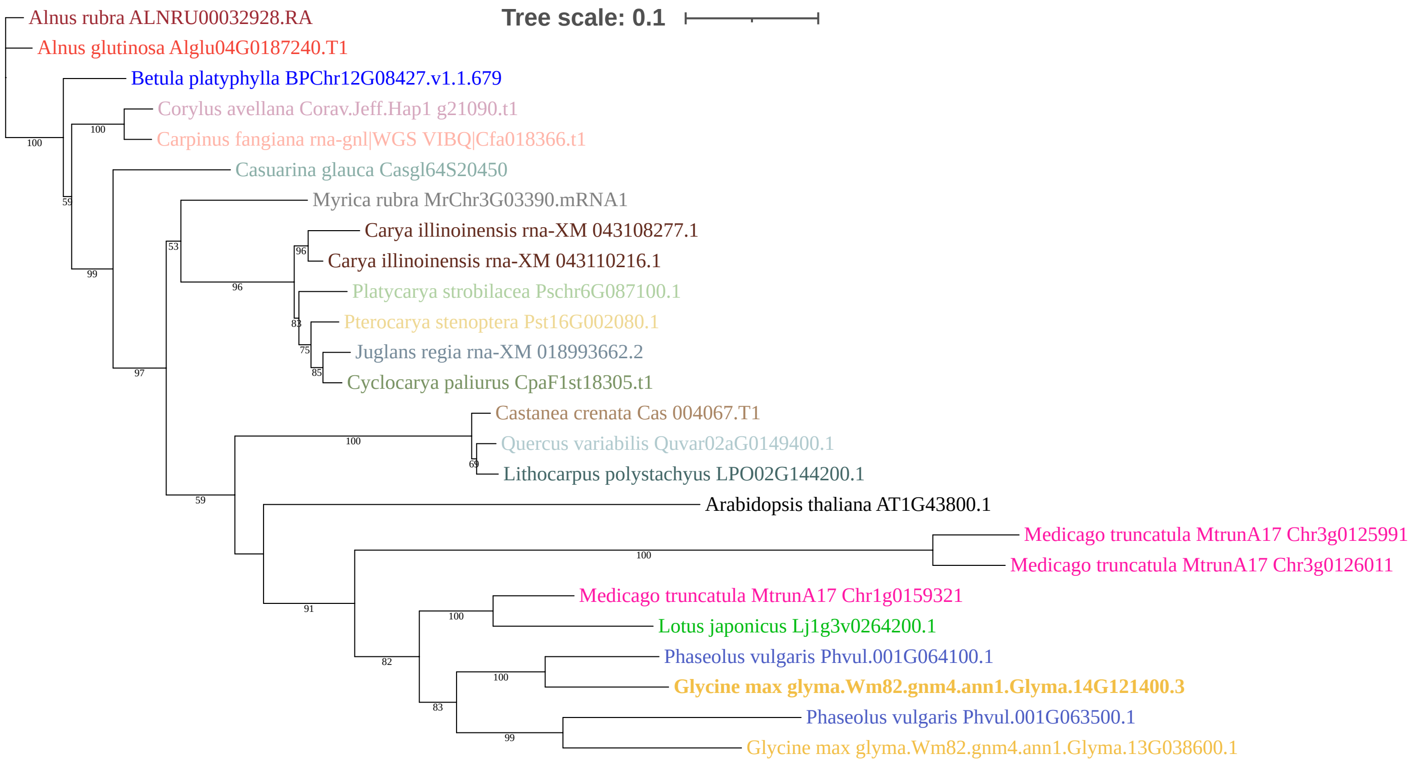

OG0009306:Mediator 16A

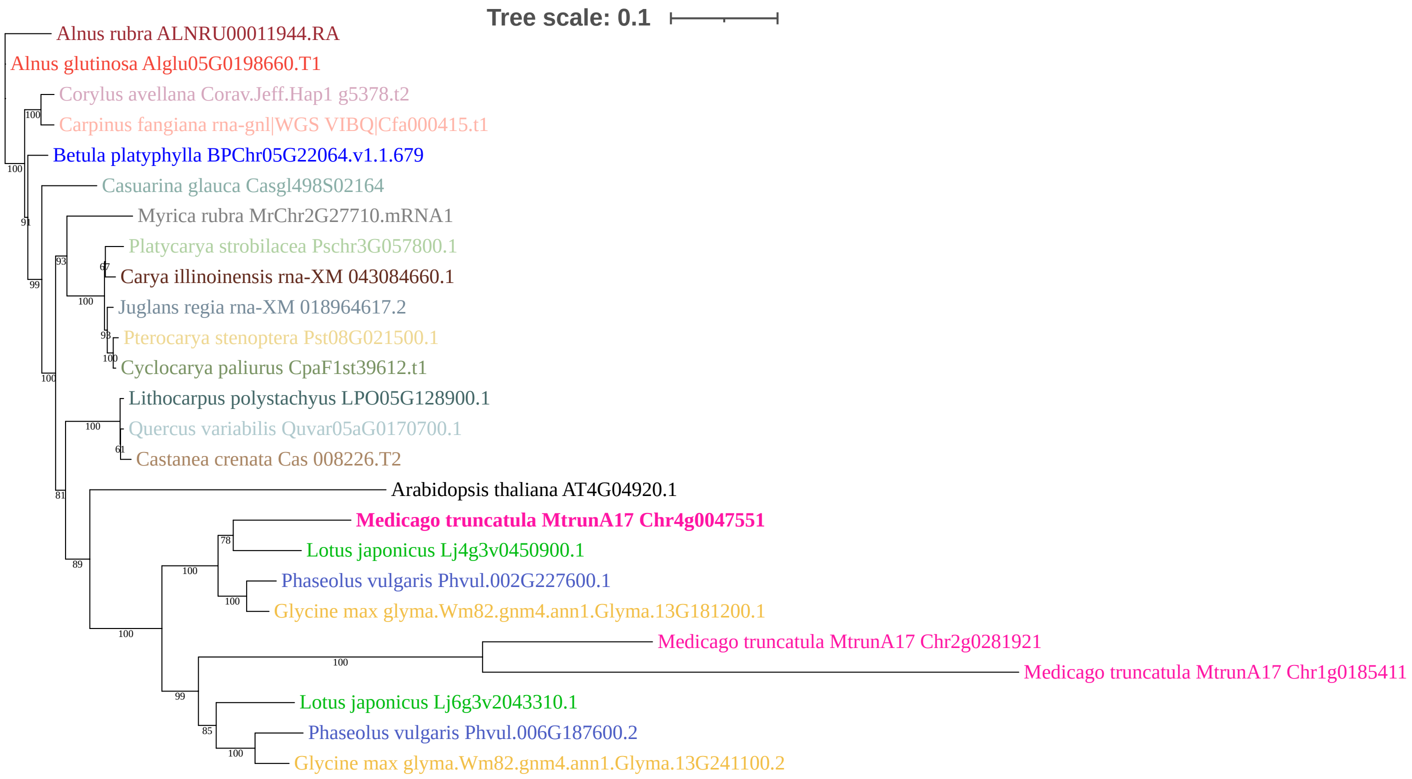

OG0009318:Nck-ASSOCIATED LIKE PROTEIN 1

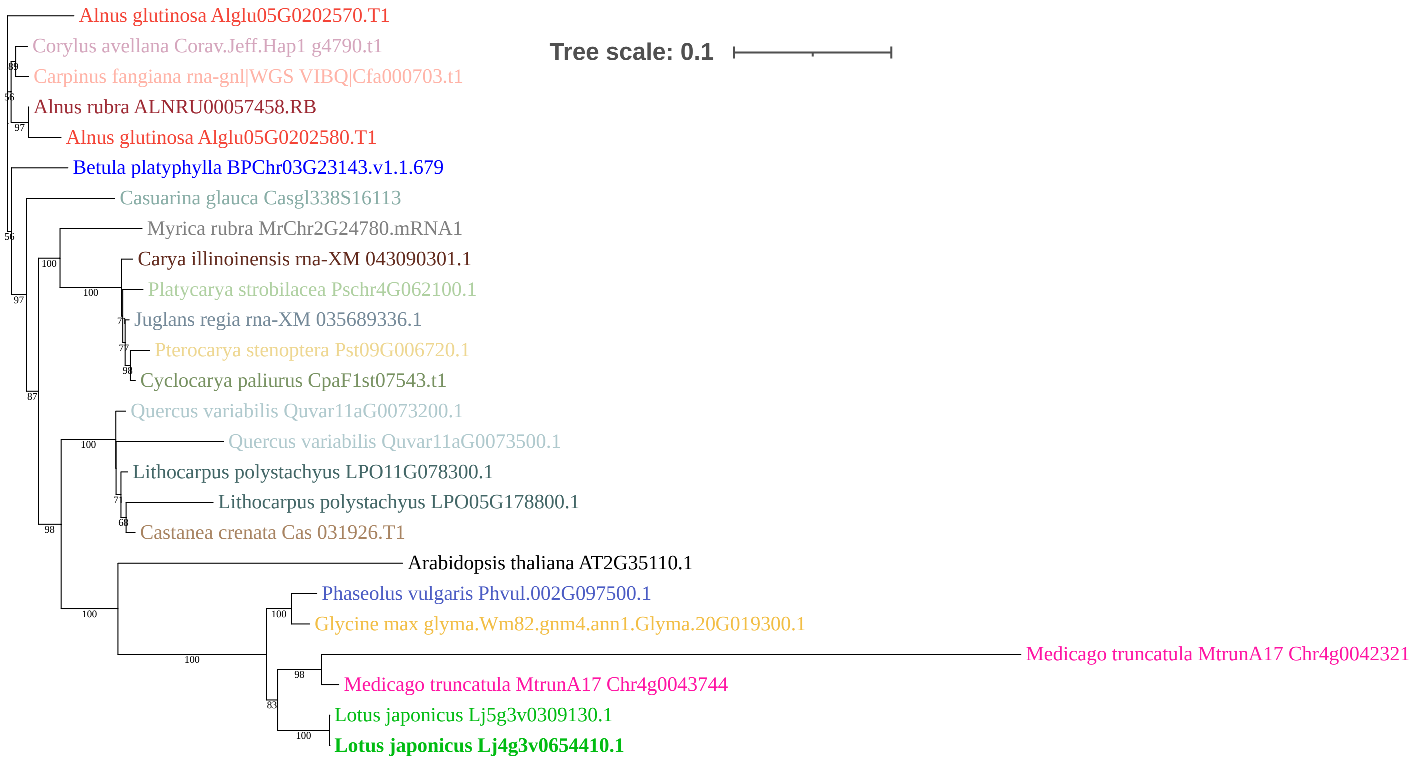

OG0009336:DEVELOPMENTALLY REGULATED PLASMA MEMBRANE POLYPEPTIDE

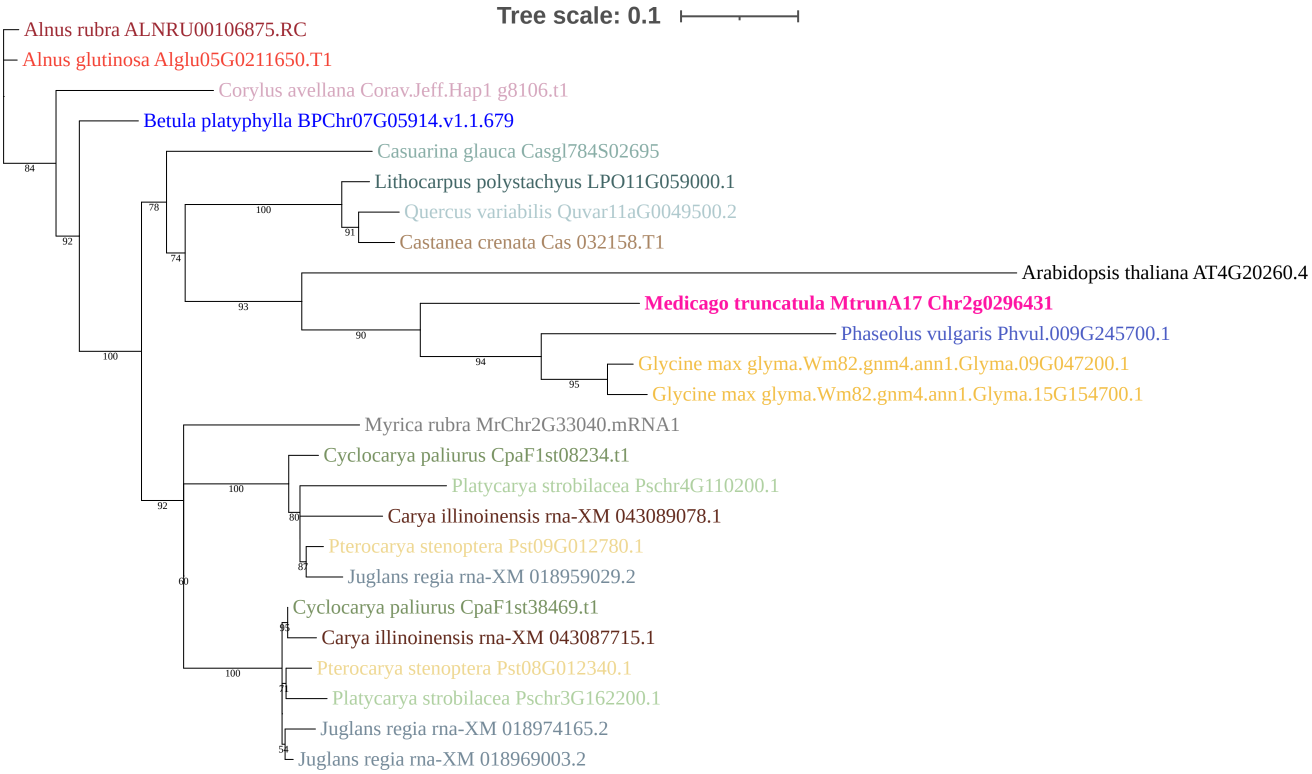

OG0009653:Rapid Alkalinization Factor

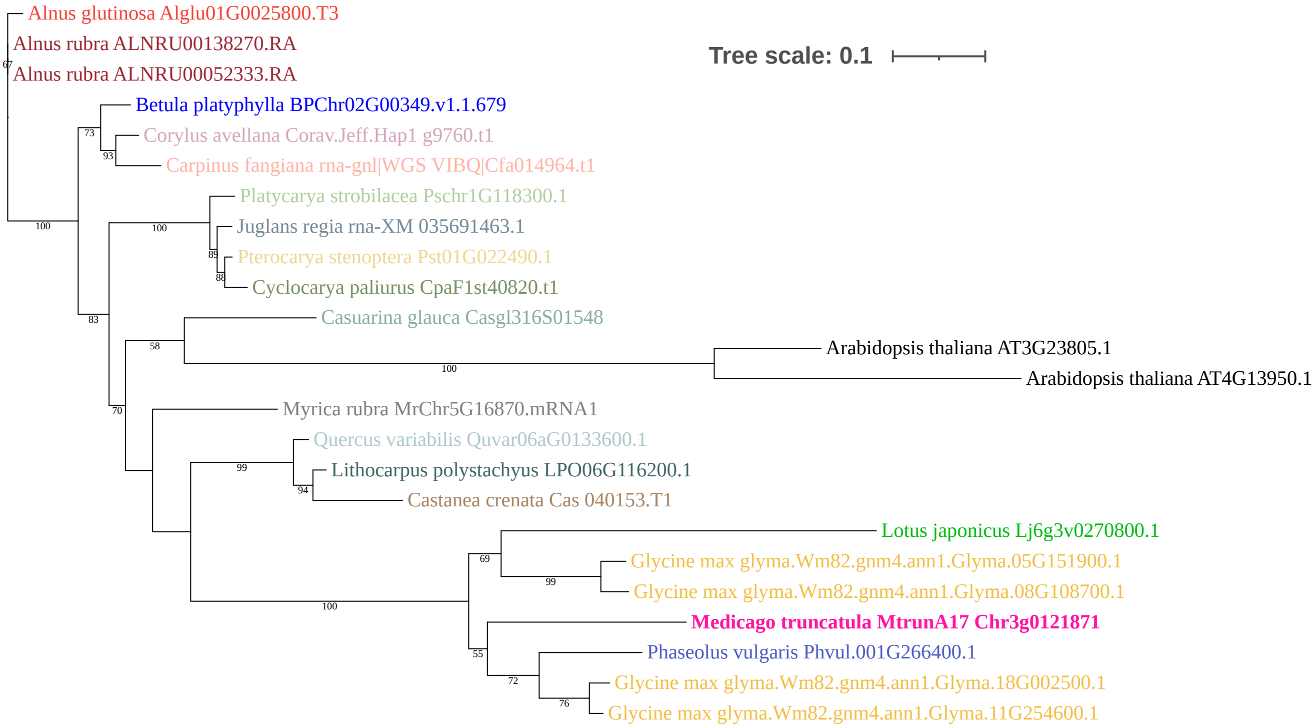

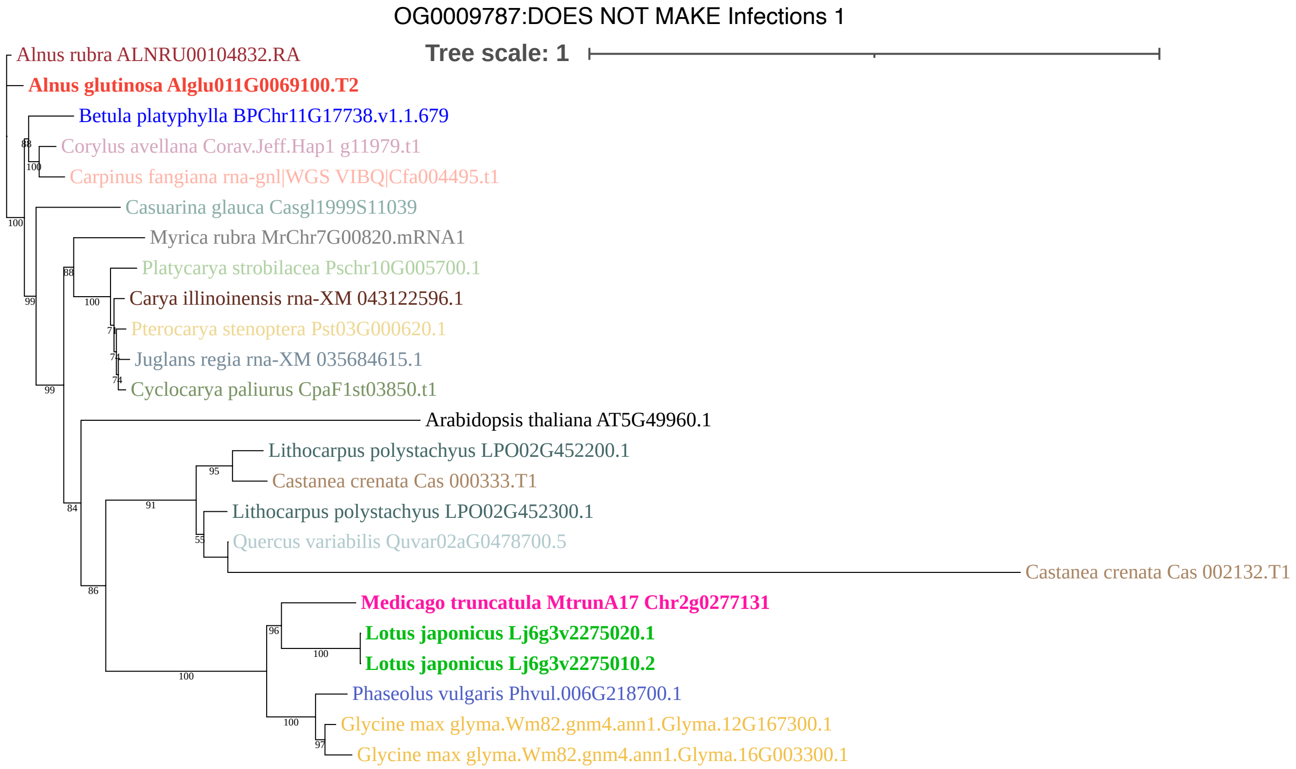
OG0009787:DOES NOT MAKE Infections 1
Tree scale: 1
Alnus rubra ALNRU00104832.RA
Alnus glutinosa Alglu011G0069100.T2
Betula platyphylla BPChr11G17738.v1.1.679
Corylus avellana Corav.Jeff.Hap1 g11979.t1
Carpinus fangiana rna-gnl|WGS VIBQ|Cfa004495.t1
Casuarina glauca Casgl1999S11039
Myrica rubra MrChr7G00820.mRNA1
Platycarya strobilacea Pschr10G005700.1
Carya illinoinensis rna-XM 043122596.1
Pterocarya stenoptera Pst03G000620.1
Juglans regia rna-XM 035684615.1
Cyclocarya paliurus CpaF1st03850.t1
Arabidopsis thaliana AT5G49960.1
Lithocarpus polystachyus LPO02G452200.1
Castanea crenata Cas 000333.T1
Lithocarpus polystachyus LPO02G452300.1
Quercus variabilis Quvar02aG0478700.5
Castanea crenata Cas 002132.T1
Medicago truncatula MtrunA17 Chr2g0277131
Lotus japonicus Lj6g3v2275020.1
Lotus japonicus Lj6g3v2275010.2
Phaseolus vulgaris Phvul.006G218700.1
Glycine max glyma.Wm82.gnm4.ann1.Glyma.12G167300.1
Glycine max glyma.Wm82.gnm4.ann1.Glyma.16G003300.1

OG0010040:NODULATION SIGNALING PATHWAY 1

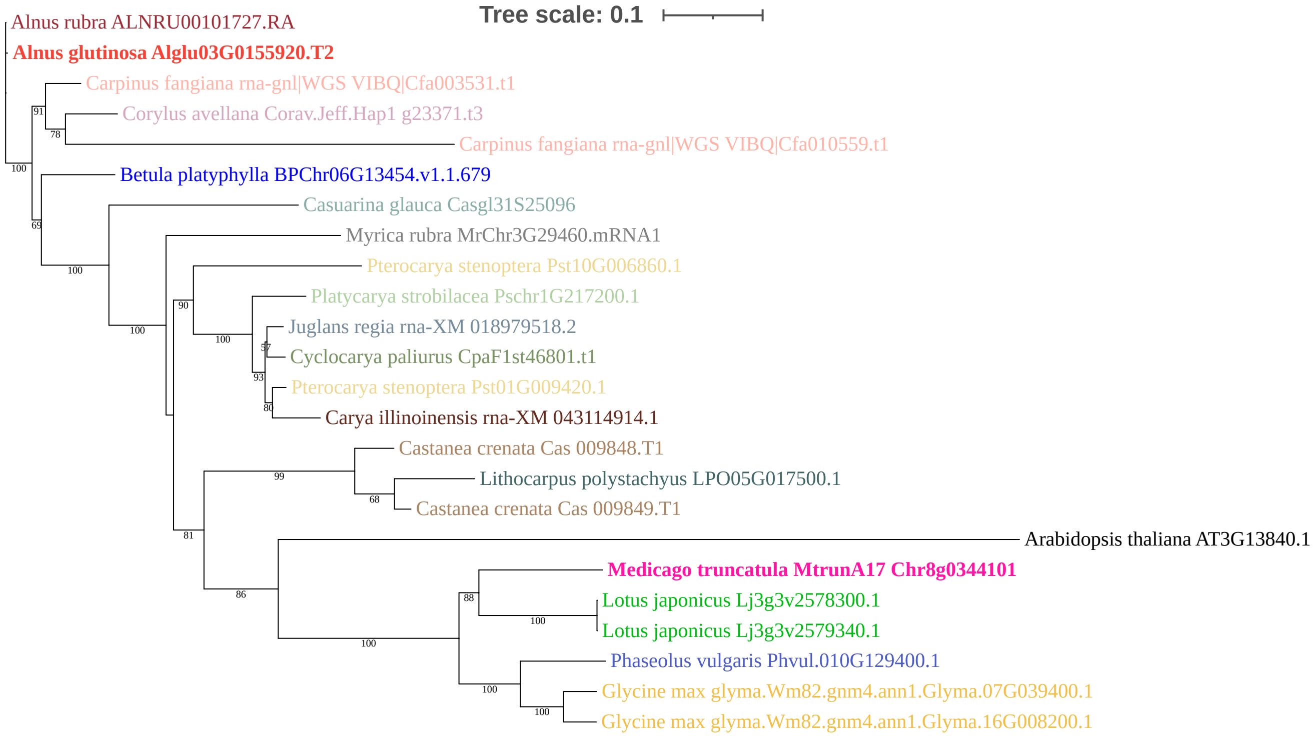

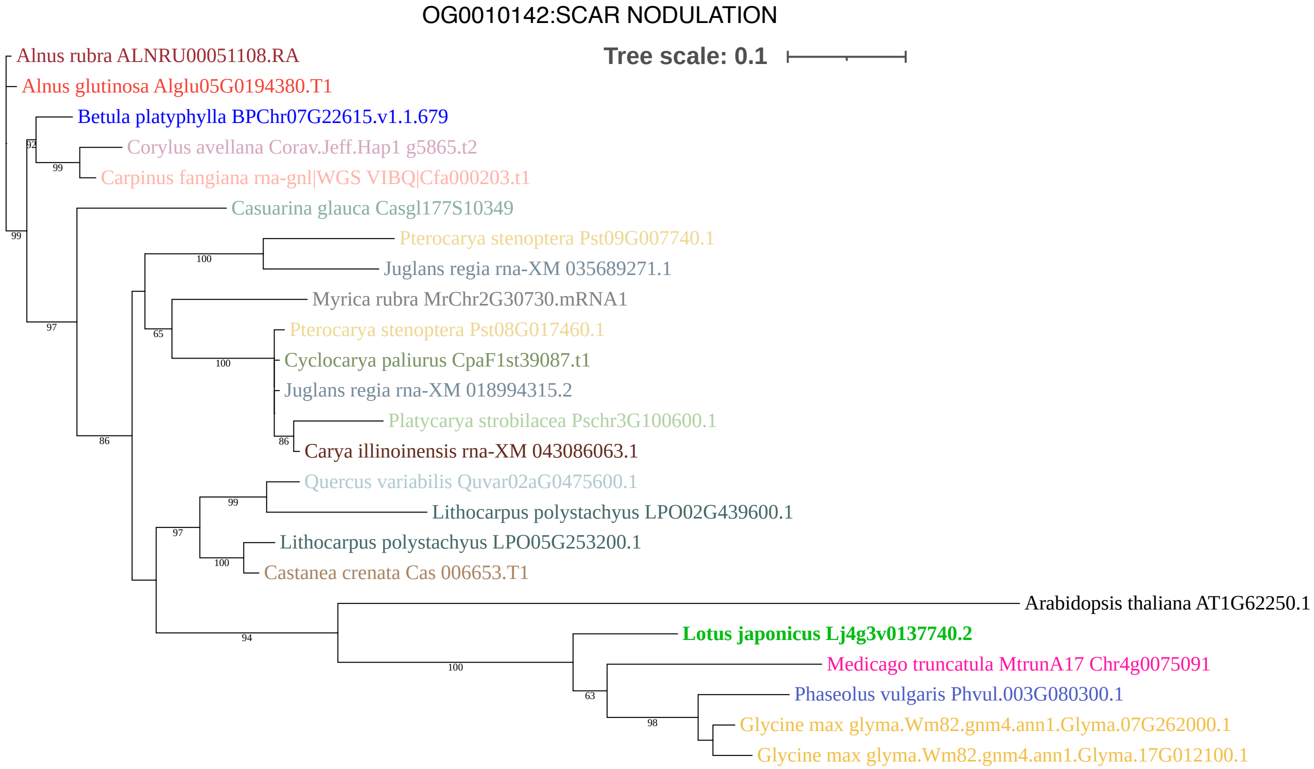
OG0010142:SCAR NODULATION
Tree scale: 0.1
Alnus rubra ALNRU00051108.RA
Alnus glutinosa Alglu05G0194380.T1
Betula platyphylla BPChr07G22615.v1.1.679
Corylus avellana Corav.Jeff.Hap1 g5865.t2
Carpinus fangiana rna-gnl|WGS VIBQ|Cfa000203.t1
Casuarina glauca Casgl177S10349
Pterocarya stenoptera Pst09G007740.1
Juglans regia rna-XM 035689271.1
Myrica rubra MrChr2G30730.mRNA1
Pterocarya stenoptera Pst08G017460.1
Cyclocarya paliurus CpaF1st39087.t1
Juglans regia rna-XM 018994315.2
Platycarya strobilacea Pschr3G100600.1
Carya illinoinensis rna-XM 043086063.1
Quercus variabilis Quvar02aG0475600.1
Lithocarpus polystachyus LPO02G439600.1
Lithocarpus polystachyus LPO05G253200.1
Castanea crenata Cas 006653.T1
Arabidopsis thaliana AT1G62250.1
Lotus japonicus Lj4g3v0137740.2
Medicago truncatula MtrunA17 Chr4g0075091
Phaseolus vulgaris Phvul.003G080300.1
Glycine max glyma.Wm82.gnm4.ann1.Glyma.07G262000.1
Glycine max glyma.Wm82.gnm4.ann1.Glyma.17G012100.1
92
99
99
97
100
65
100
86
86
99
97
100
94
100
63
98

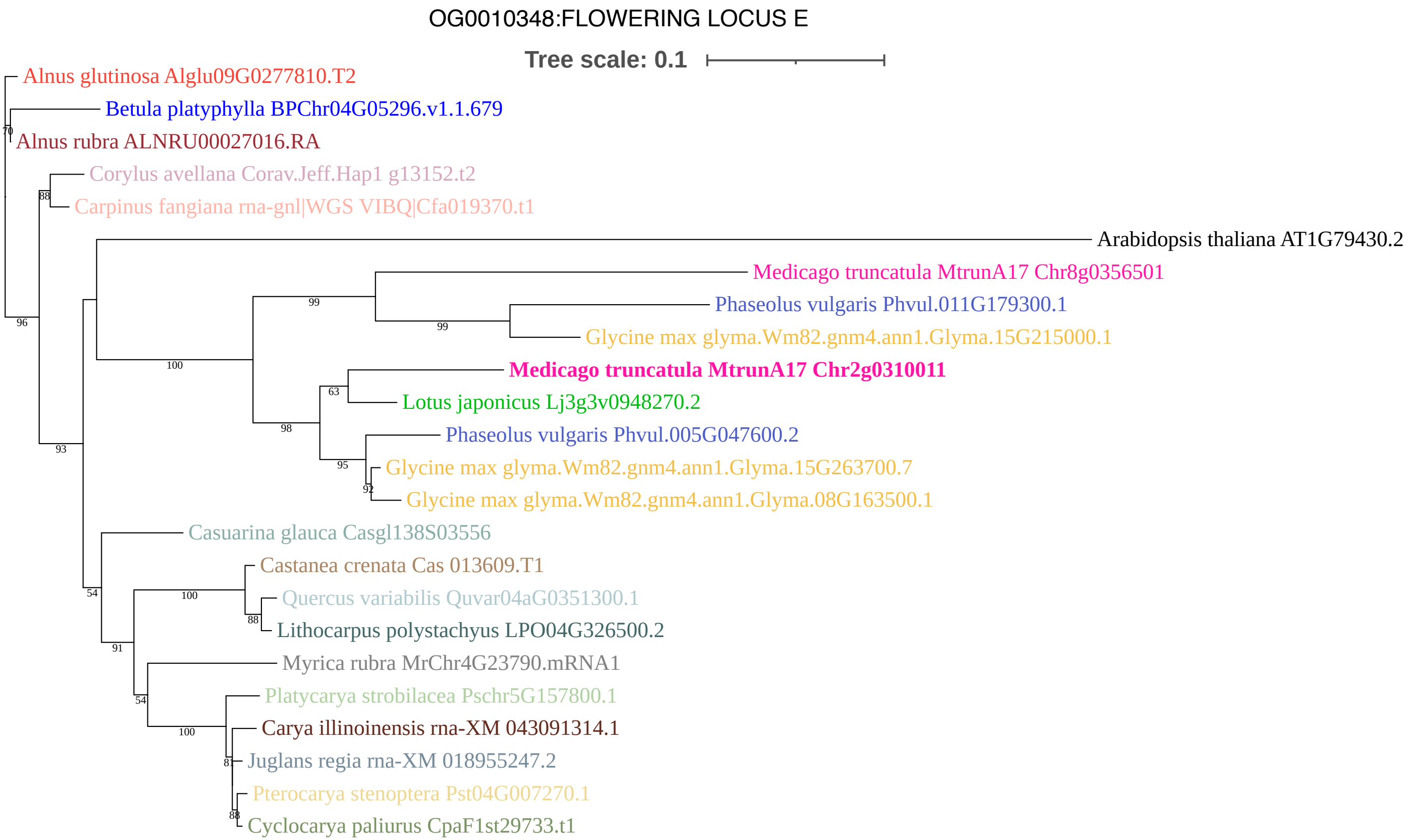
OG0010348:FLOWERING LOCUS E
Tree scale: 0.1
Alnus glutinosa Alglu09G0277810.T2
Betula platyphylla BPChr04G05296.v1.1.679
Alnus rubra ALNRU00027016.RA
Corylus avellana Corav.Jeff.Hap1 g13152.t2
Carpinus fangiana rna-gnl|WGS VIBQ|Cfa019370.t1
Arabidopsis thaliana AT1G79430.2
Medicago truncatula MtrunA17 Chr8g0356501
Phaseolus vulgaris Phvul.011G179300.1
Glycine max glyma.Wm82.gnm4.ann1.Glyma.15G215000.1
Medicago truncatula MtrunA17 Chr2g0310011
Lotus japonicus Lj3g3v0948270.2
Phaseolus vulgaris Phvul.005G047600.2
Glycine max glyma.Wm82.gnm4.ann1.Glyma.15G263700.7
Glycine max glyma.Wm82.gnm4.ann1.Glyma.08G163500.1
Casuarina glauca Casgl138S03556
Castanea crenata Cas 013609.T1
Quercus variabilis Quvar04aG0351300.1
Lithocarpus polystachyus LPO04G326500.2
Myrica rubra MrChr4G23790.mRNA1
Platycarya strobilacea Pschr5G157800.1
Carya illinoinensis rna-XM 043091314.1
Juglans regia rna-XM 018955247.2
Pterocarya stenoptera Pst04G007270.1
Cyclocarya paliurus CpaF1st29733.t1
70
88
96
99
99
100
63
98
95
92
93
54
100
88
91
54
100
81
88

OG0010448:sulfate transporter

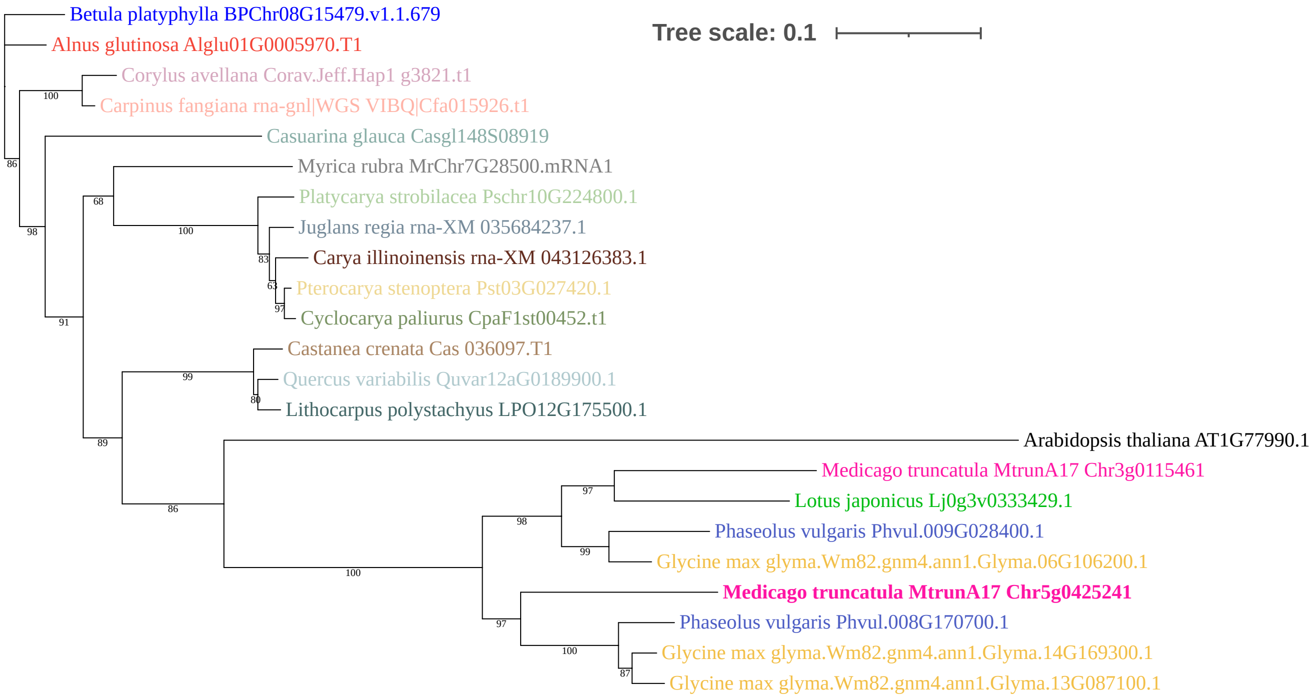

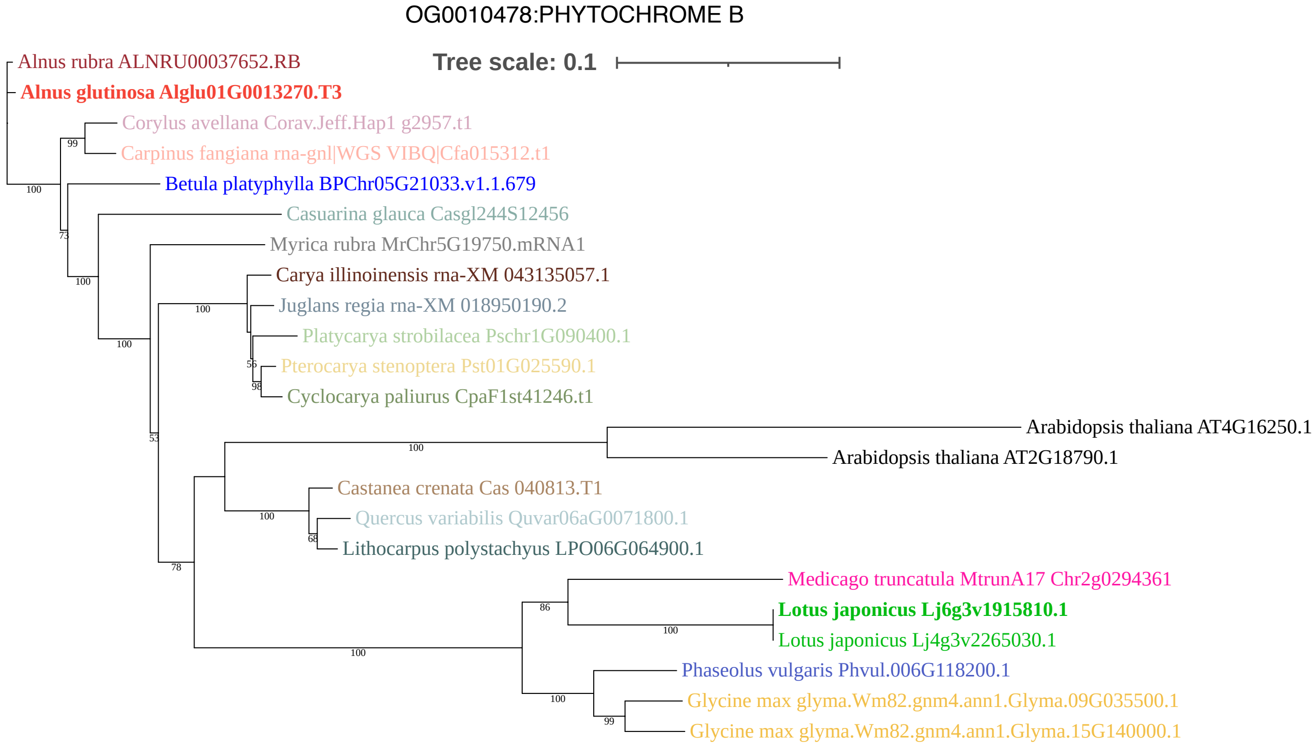
OG0010478:PHYTOCHROME B
Tree scale: 0.1
Alnus rubra ALNRU00037652.RB
Alnus glutinosa Alglu01G0013270.T3
Corylus avellana Corav.Jeff.Hap1 g2957.t1
Carpinus fangiana rna-gnl|WGS VIBQ|Cfa015312.t1
Betula platyphylla BPChr05G21033.v1.1.679
Casuarina glauca Casgl244S12456
Myrica rubra MrChr5G19750.mRNA1
Carya illinoinensis rna-XM 043135057.1
Juglans regia rna-XM 018950190.2
Platycarya strobilacea Pschr1G090400.1
Pterocarya stenoptera Pst01G025590.1
Cyclocarya paliurus CpaF1st41246.t1
Arabidopsis thaliana AT4G16250.1
Arabidopsis thaliana AT2G18790.1
Castanea crenata Cas 040813.T1
Quercus variabilis Quvar06aG0071800.1
Lithocarpus polystachyus LPO06G064900.1
Medicago truncatula MtrunA17 Chr2g0294361
Lotus japonicus Lj6g3v1915810.1
Lotus japonicus Lj4g3v2265030.1
Phaseolus vulgaris Phvul.006G118200.1
Glycine max glyma.Wm82.gnm4.ann1.Glyma.09G035500.1
Glycine max glyma.Wm82.gnm4.ann1.Glyma.15G140000.1
99
100
73
100
100
100
56
98
53
100
100
68
78
86
100
100
100
99

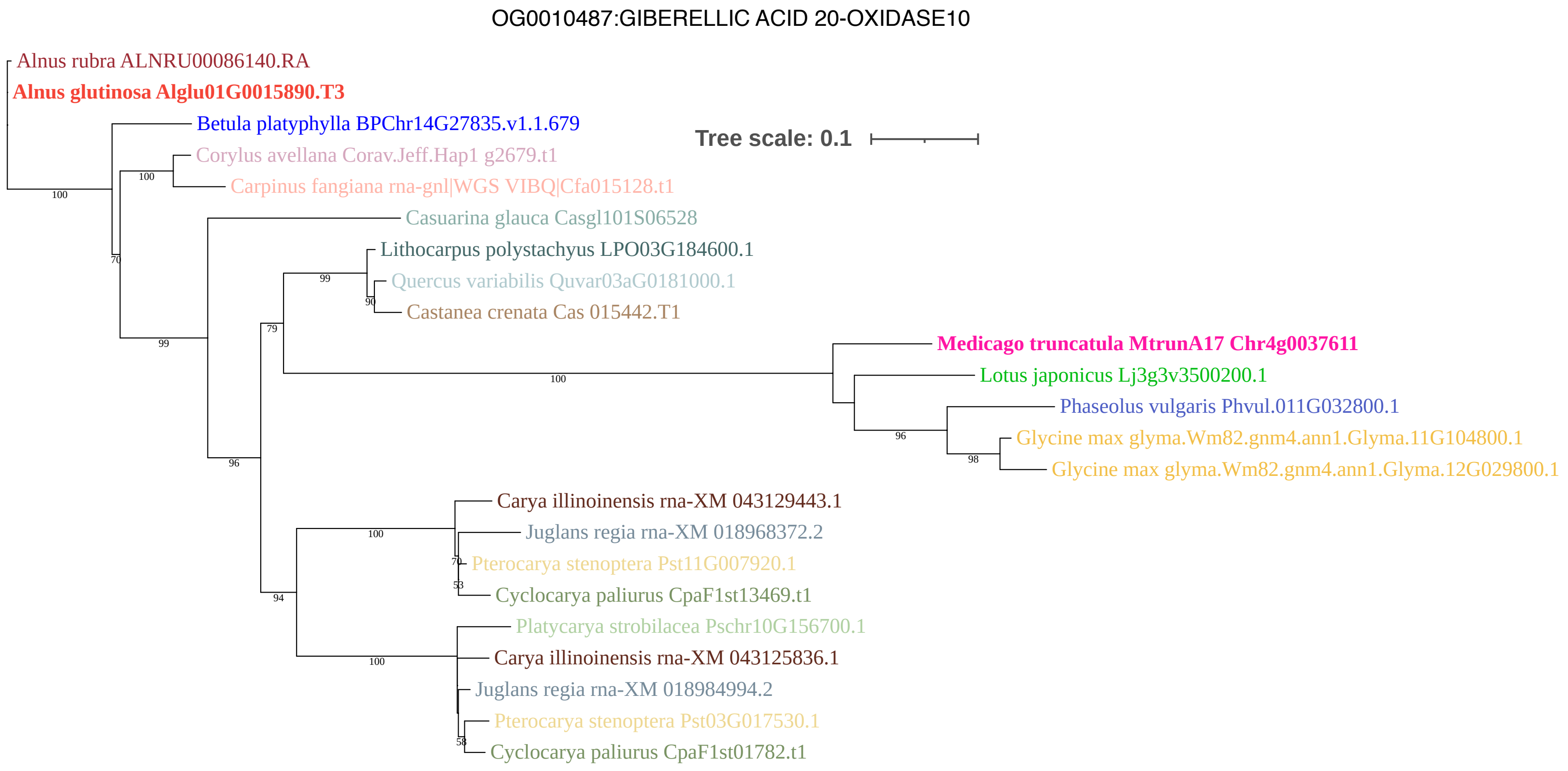
OG0010487:GIBERELLIC ACID 20-OXIDASE10
Tree scale: 0.1
Alnus rubra ALNRU00086140.RA
Alnus glutinosa Alglu01G0015890.T3
Betula platyphylla BPChr14G27835.v1.1.679
Corylus avellana Corav.Jeff.Hap1 g2679.t1
Carpinus fangiana rna-gnl|WGS VIBQ|Cfa015128.t1
Casuarina glauca Casgl101S06528
Lithocarpus polystachyus LPO03G184600.1
Quercus variabilis Quvar03aG0181000.1
Castanea crenata Cas 015442.T1
Medicago truncatula MtrunA17 Chr4g0037611
Lotus japonicus Lj3g3v3500200.1
Phaseolus vulgaris Phvul.011G032800.1
Glycine max glyma.Wm82.gnm4.ann1.Glyma.11G104800.1
Glycine max glyma.Wm82.gnm4.ann1.Glyma.12G029800.1
Carya illinoinensis rna-XM 043129443.1
Juglans regia rna-XM 018968372.2
Pterocarya stenoptera Pst11G007920.1
Cyclocarya paliurus CpaF1st13469.t1
Platycarya strobilacea Pschr10G156700.1
Carya illinoinensis rna-XM 043125836.1
Juglans regia rna-XM 018984994.2
Pterocarya stenoptera Pst03G017530.1
Cyclocarya paliurus CpaF1st01782.t1
100
100
70
99
99
90
79
100
96
98
96
100
70
53
94
100
58

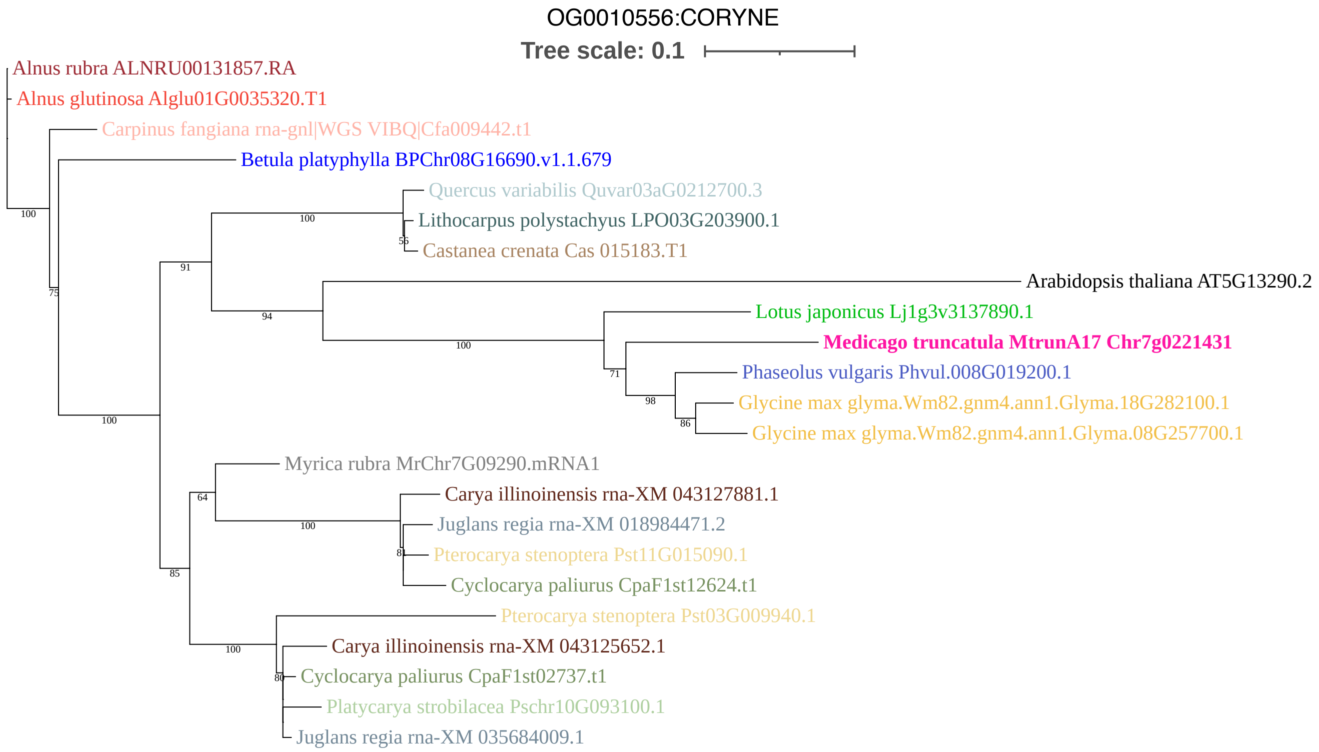
OG0010556:CORYNE
Tree scale: 0.1
Alnus rubra ALNRU00131857.RA
Alnus glutinosa Alglu01G0035320.T1
Carpinus fangiana rna-gnl|WGS VIBQ|Cfa009442.t1
Betula platyphylla BPChr08G16690.v1.1.679
Quercus variabilis Quvar03aG0212700.3
Lithocarpus polystachyus LPO03G203900.1
Castanea crenata Cas 015183.T1
Arabidopsis thaliana AT5G13290.2
Lotus japonicus Lj1g3v3137890.1
Medicago truncatula MtrunA17 Chr7g0221431
Phaseolus vulgaris Phvul.008G019200.1
Glycine max glyma.Wm82.gnm4.ann1.Glyma.18G282100.1
Glycine max glyma.Wm82.gnm4.ann1.Glyma.08G257700.1
Myrica rubra MrChr7G09290.mRNA1
Carya illinoinensis rna-XM 043127881.1
Juglans regia rna-XM 018984471.2
Pterocarya stenoptera Pst11G015090.1
Cyclocarya paliurus CpaF1st12624.t1
Pterocarya stenoptera Pst03G009940.1
Carya illinoinensis rna-XM 043125652.1
Cyclocarya paliurus CpaF1st02737.t1
Platycarya strobilacea Pschr10G093100.1
Juglans regia rna-XM 035684009.1
100
100
56
91
75
94
100
71
98
86
100
64
100
81
85
100
80

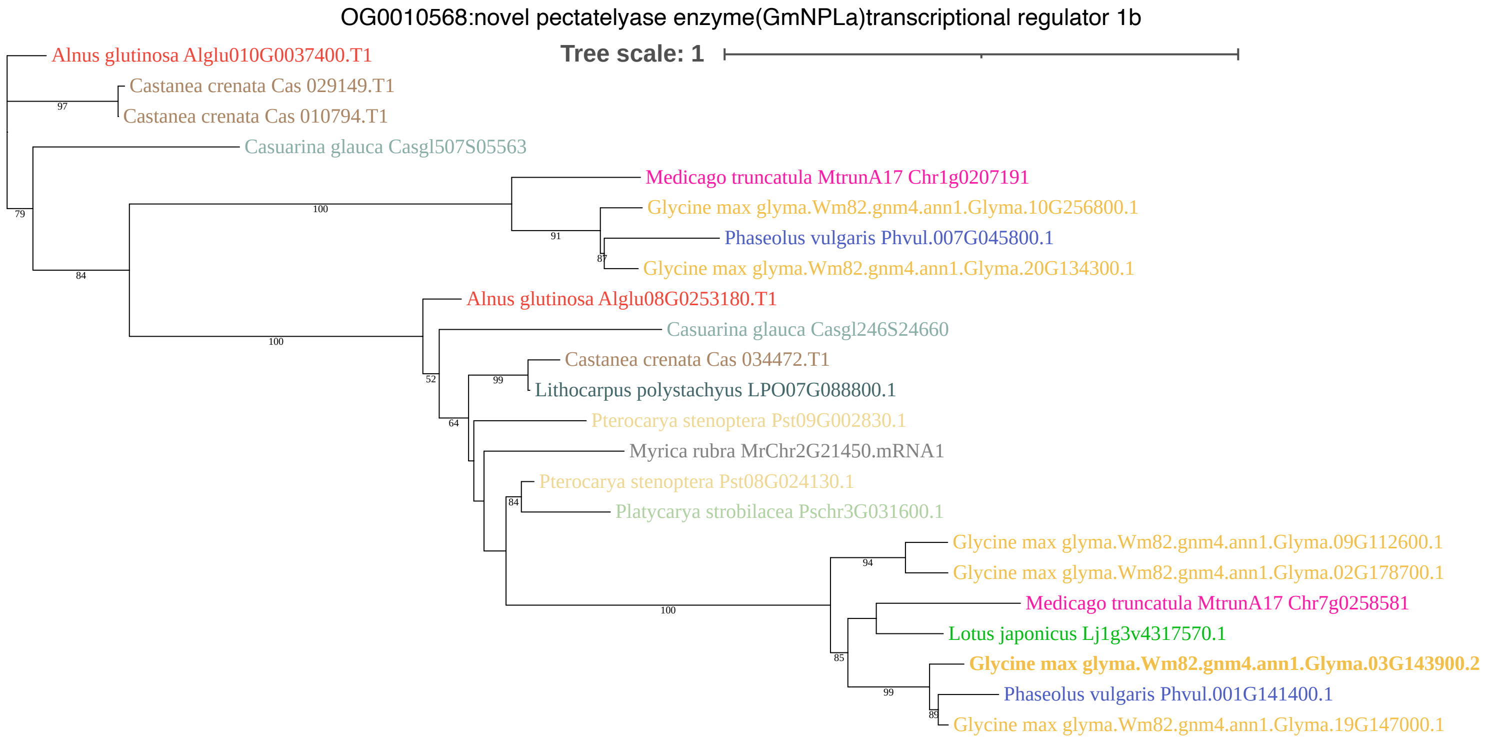
OG0010568:novel pectatelyase enzyme(GmNPLa)transcriptional regulator 1b
Tree scale: 1
Alnus glutinosa Alglu010G0037400.T1
Castanea crenata Cas 029149.T1
Castanea crenata Cas 010794.T1
Casuarina glauca Casgl507S05563
Medicago truncatula MtrunA17 Chr1g0207191
Glycine max glyma.Wm82.gnm4.ann1.Glyma.10G256800.1
Phaseolus vulgaris Phvul.007G045800.1
Glycine max glyma.Wm82.gnm4.ann1.Glyma.20G134300.1
Alnus glutinosa Alglu08G0253180.T1
Casuarina glauca Casgl246S24660
Castanea crenata Cas 034472.T1
Lithocarpus polystachyus LPO07G088800.1
Pterocarya stenoptera Pst09G002830.1
Myrica rubra MrChr2G21450.mRNA1
Pterocarya stenoptera Pst08G024130.1
Platycarya strobilacea Pschr3G031600.1
Glycine max glyma.Wm82.gnm4.ann1.Glyma.09G112600.1
Glycine max glyma.Wm82.gnm4.ann1.Glyma.02G178700.1
Medicago truncatula MtrunA17 Chr7g0258581
Lotus japonicus Lj1g3v4317570.1
Glycine max glyma.Wm82.gnm4.ann1.Glyma.03G143900.2
Phaseolus vulgaris Phvul.001G141400.1
Glycine max glyma.Wm82.gnm4.ann1.Glyma.19G147000.1
97
79
84
100
91
87
100
52
99
64
84
94
100
85
99
89

OG0010694:121F-specific p53 INDUCIBLE RNA

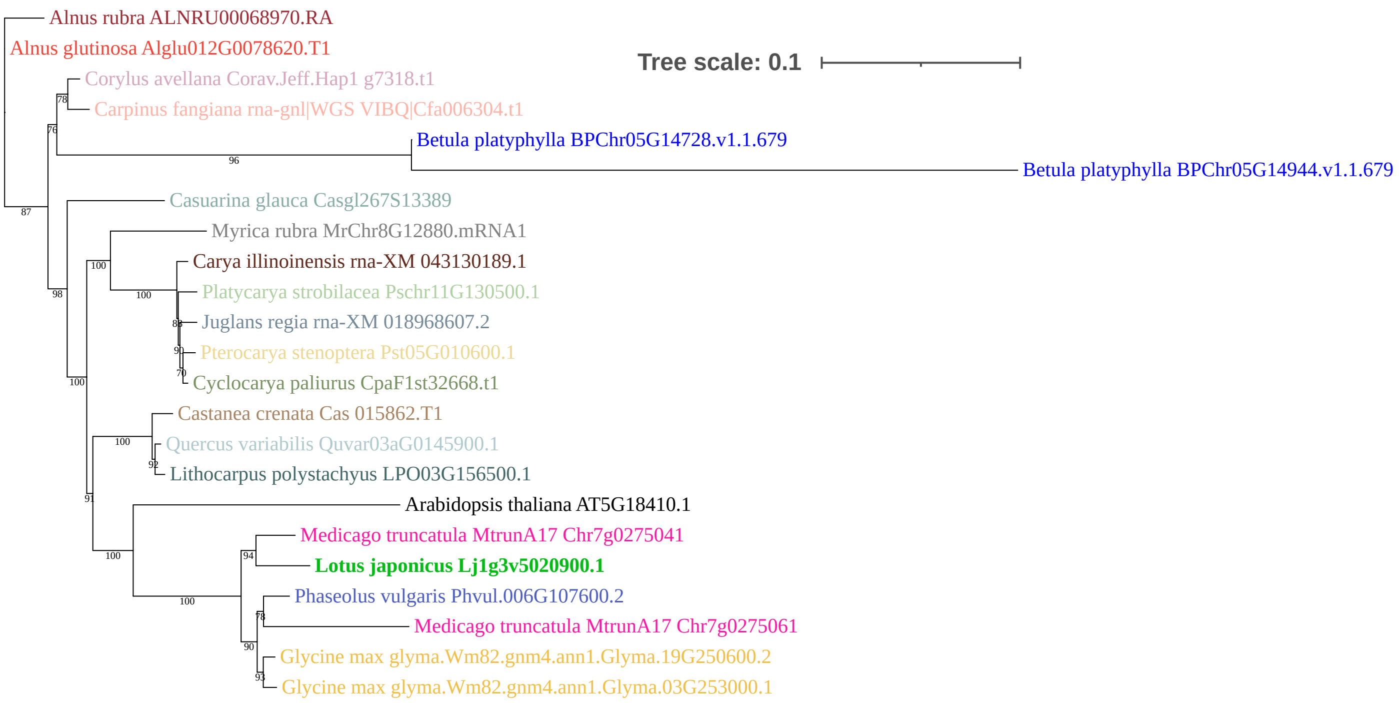

OG0011113:SYMRK INTERACTING PROTEIN 1

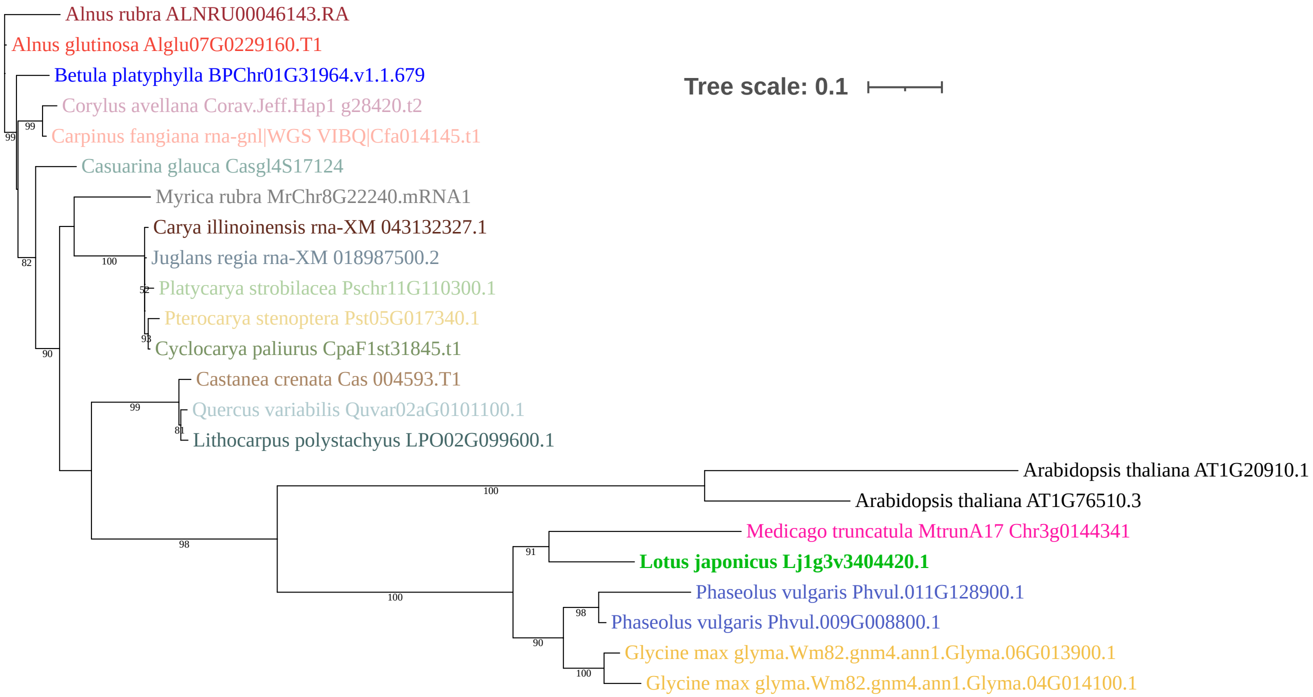

OG0011363:MADS transcription factor

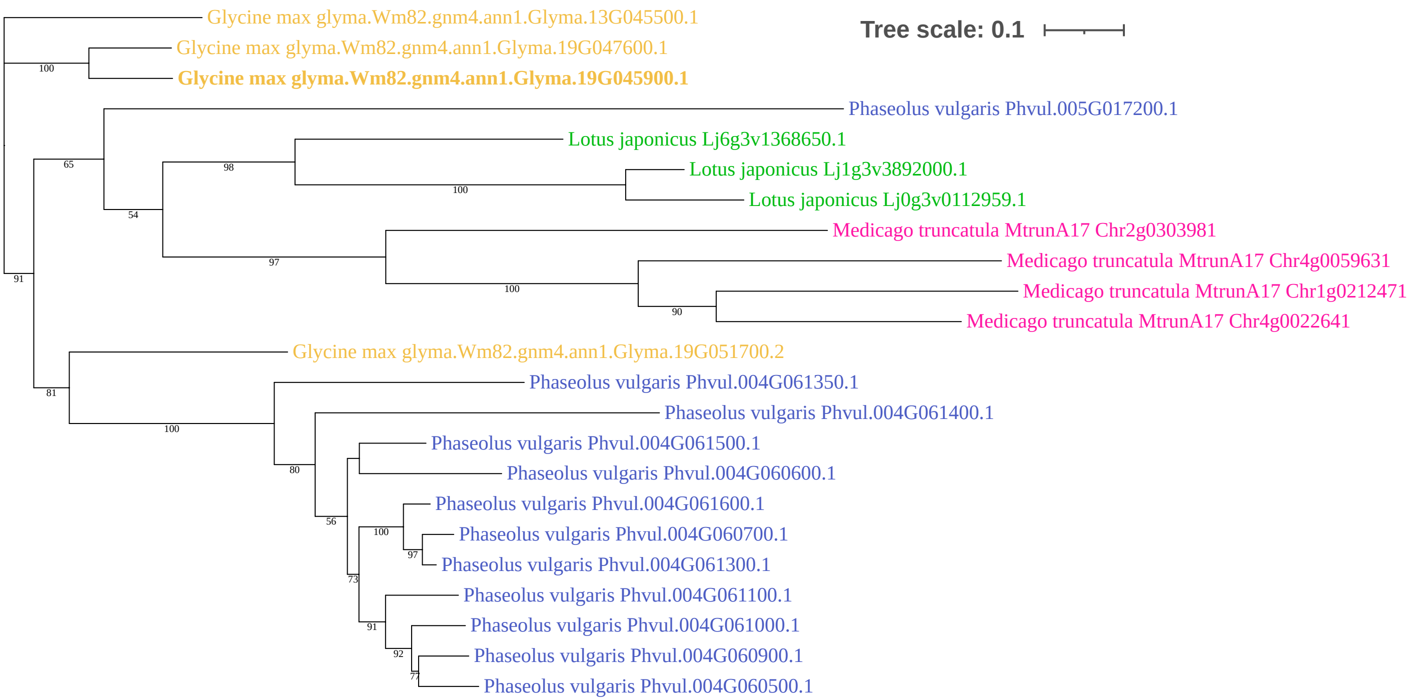

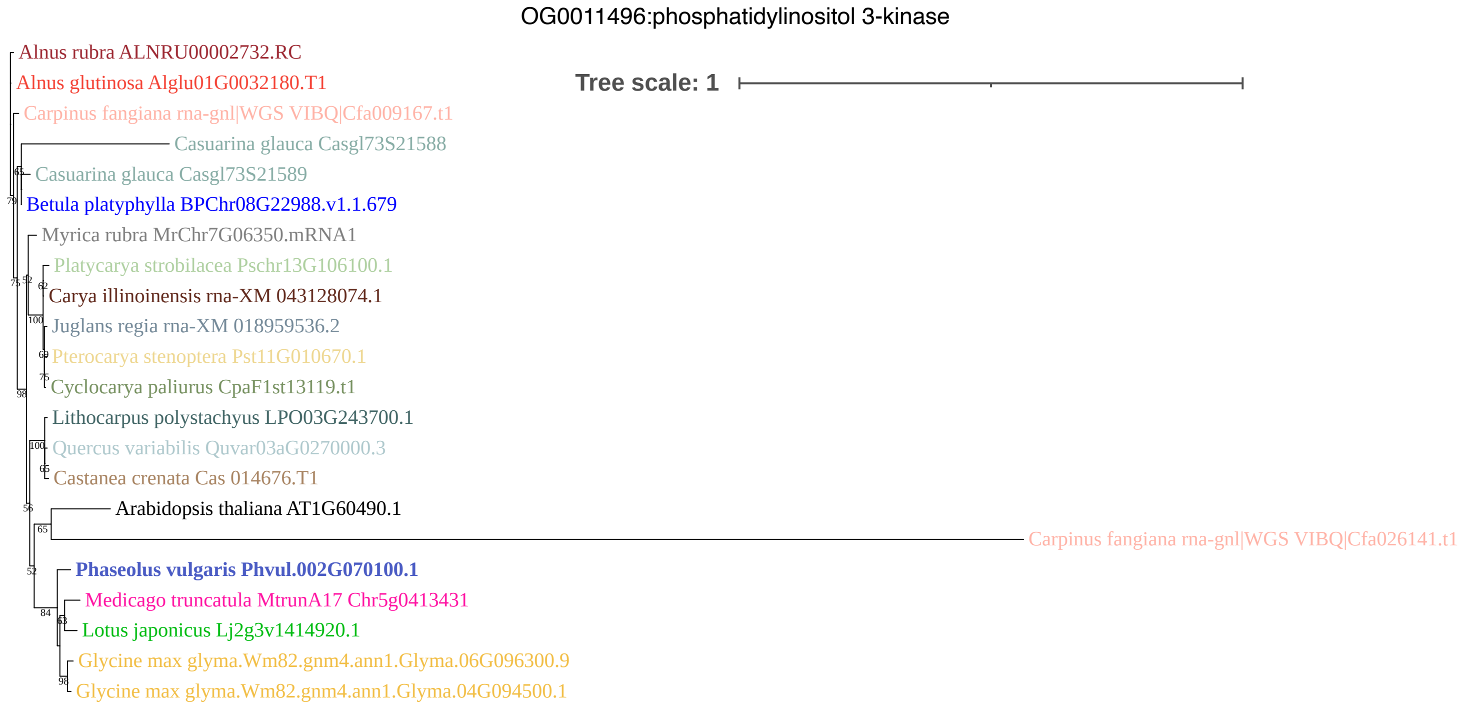
OG0011496:phosphatidylinositol 3-kinase
Tree scale: 1
Alnus rubra ALNRU00002732.RC
Alnus glutinosa Alglu01G0032180.T1
Carpinus fangiana rna-gnl|WGS VIBQ|Cfa009167.t1
Casuarina glauca Casgl73S21588
Casuarina glauca Casgl73S21589
Betula platyphylla BPChr08G22988.v1.1.679
Myrica rubra MrChr7G06350.mRNA1
Platycarya strobilacea Pschr13G106100.1
Carya illinoinensis rna-XM 043128074.1
Juglans regia rna-XM 018959536.2
Pterocarya stenoptera Pst11G010670.1
Cyclocarya paliurus CpaF1st13119.t1
Lithocarpus polystachyus LPO03G243700.1
Quercus variabilis Quvar03aG0270000.3
Castanea crenata Cas 014676.T1
Arabidopsis thaliana AT1G60490.1
Carpinus fangiana rna-gnl|WGS VIBQ|Cfa026141.t1
Phaseolus vulgaris Phvul.002G070100.1
Medicago truncatula MtrunA17 Chr5g0413431
Lotus japonicus Lj2g3v1414920.1
Glycine max glyma.Wm82.gnm4.ann1.Glyma.06G096300.9
Glycine max glyma.Wm82.gnm4.ann1.Glyma.04G094500.1

OG0011563:METAL TOLERANCE PROTEIN 2

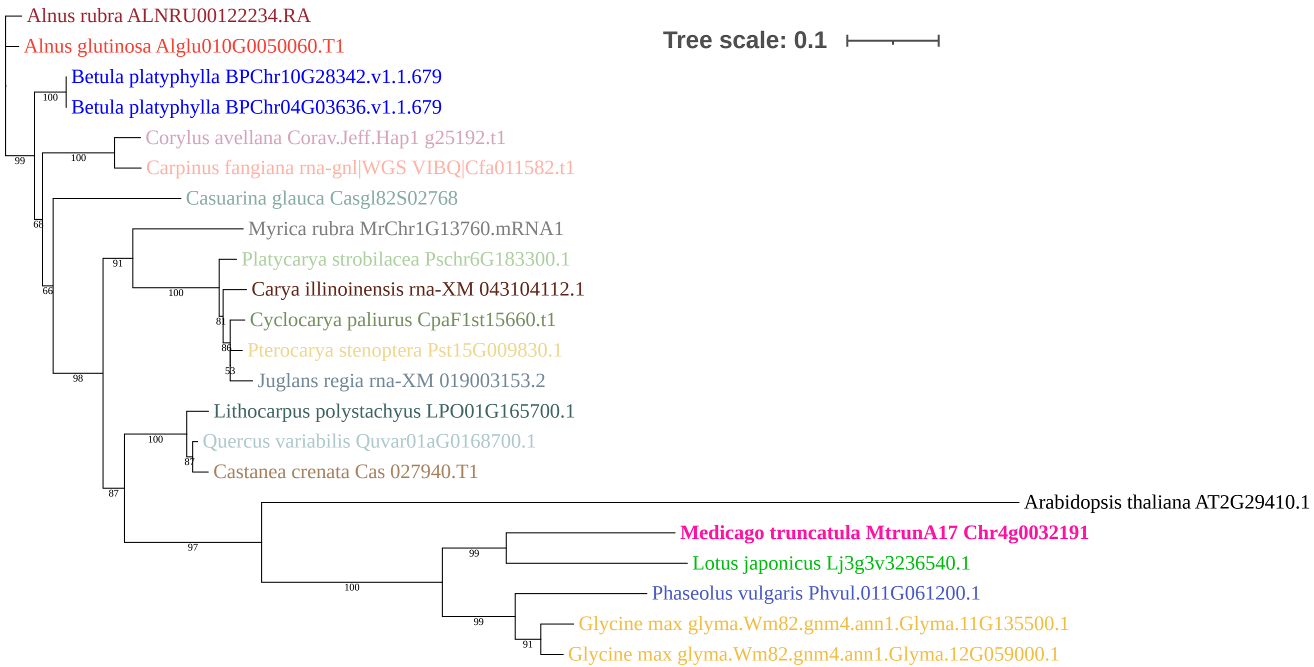

OG0011599:NOD FACTOR RECEPTOR 5

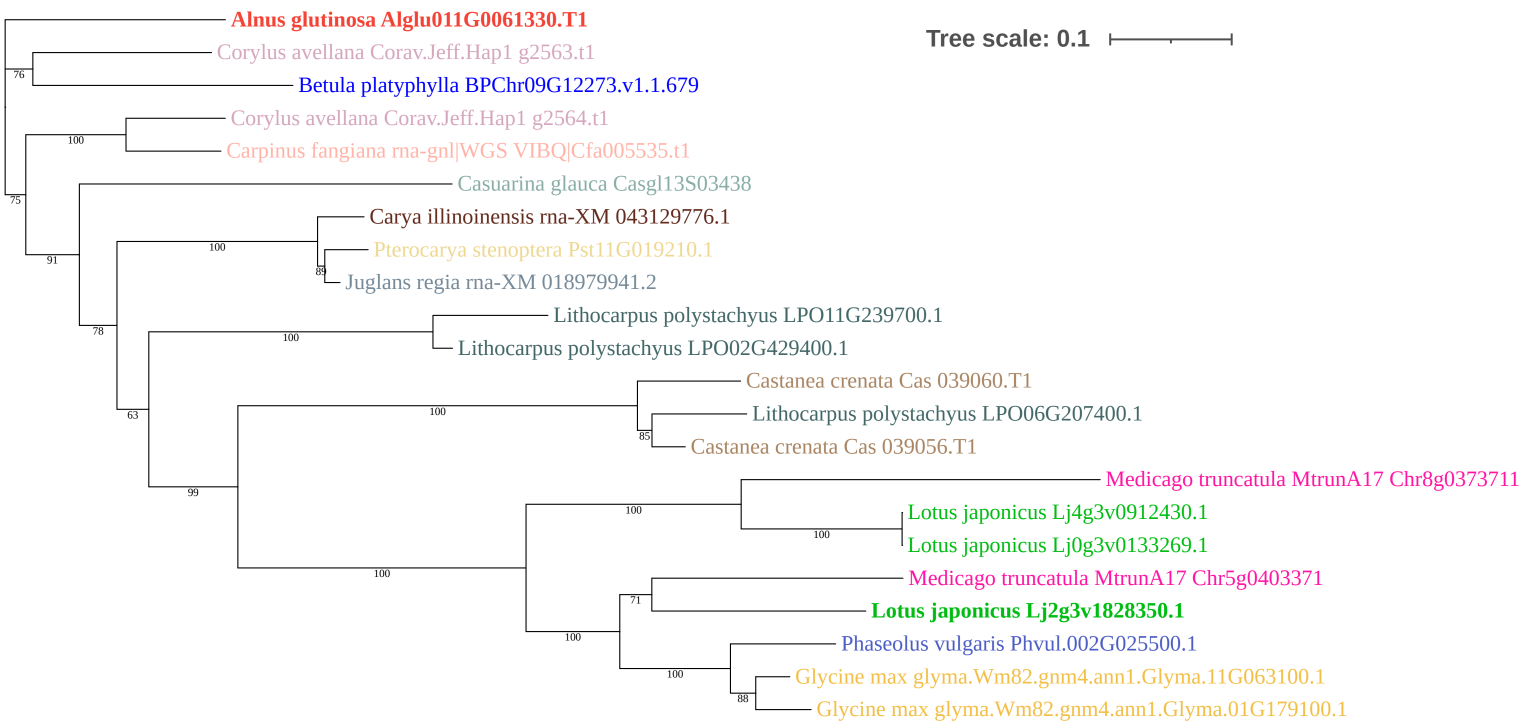

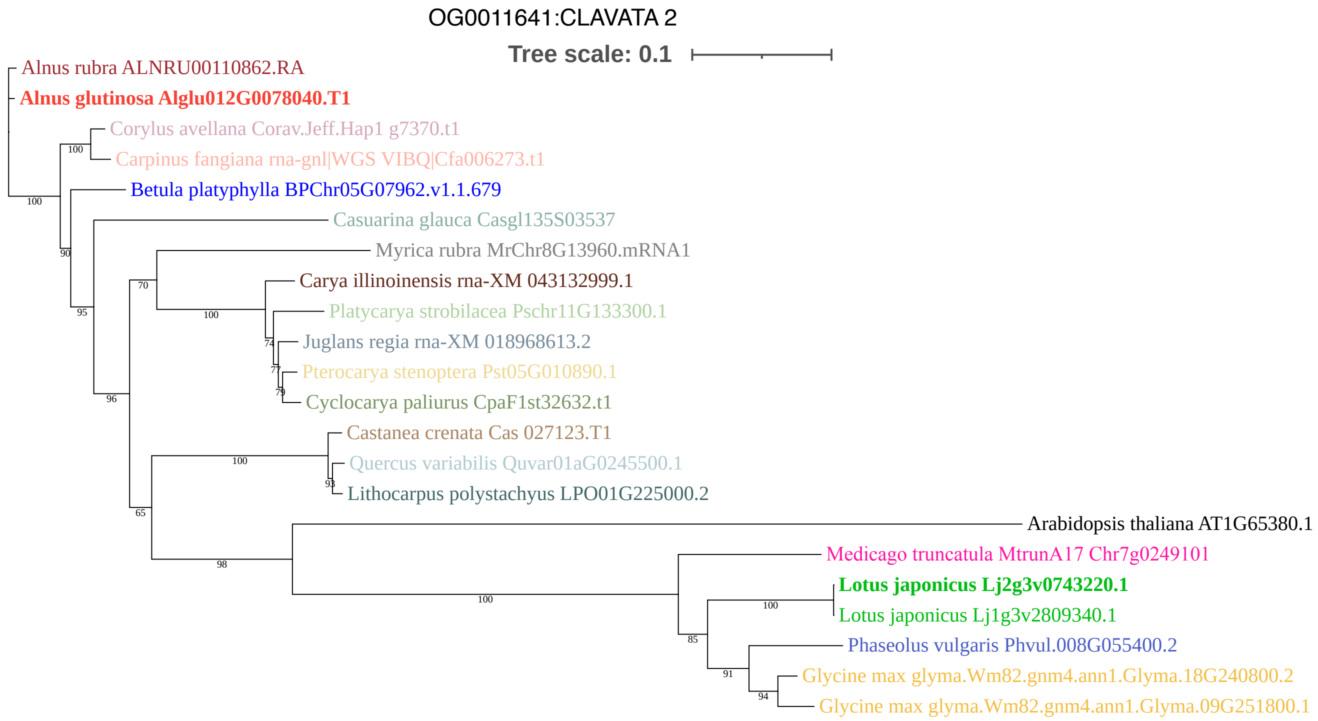

OG0011641:CLAVATA 2
Tree scale: 0.1
Alnus rubra ALNRU00110862.RA
Alnus glutinosa Alglu012G0078040.T1
Corylus avellana Corav.Jeff.Hap1 g7370.t1
Carpinus fangiana rna-gnl|WGS VIBQ|Cfa006273.t1
Betula platyphylla BPChr05G07962.v1.1.679
Casuarina glauca Casgl135S03537
Myrica rubra MrChr8G13960.mRNA1
Carya illinoinensis rna-XM 043132999.1
Platycarya strobilacea Pschr11G133300.1
Juglans regia rna-XM 018968613.2
Pterocarya stenoptera Pst05G010890.1
Cyclocarya paliurus CpaF1st32632.t1
Castanea crenata Cas 027123.T1
Quercus variabilis Quvar01aG0245500.1
Lithocarpus polystachyus LPO01G225000.2
Arabidopsis thaliana AT1G65380.1
Medicago truncatula MtrunA17 Chr7g0249101
Lotus japonicus Lj2g3v0743220.1
Lotus japonicus Lj1g3v2809340.1
Phaseolus vulgaris Phvul.008G055400.2
Glycine max glyma.Wm82.gnm4.ann1.Glyma.18G240800.2
Glycine max glyma.Wm82.gnm4.ann1.Glyma.09G251800.1

OG0011706:DELLA 3

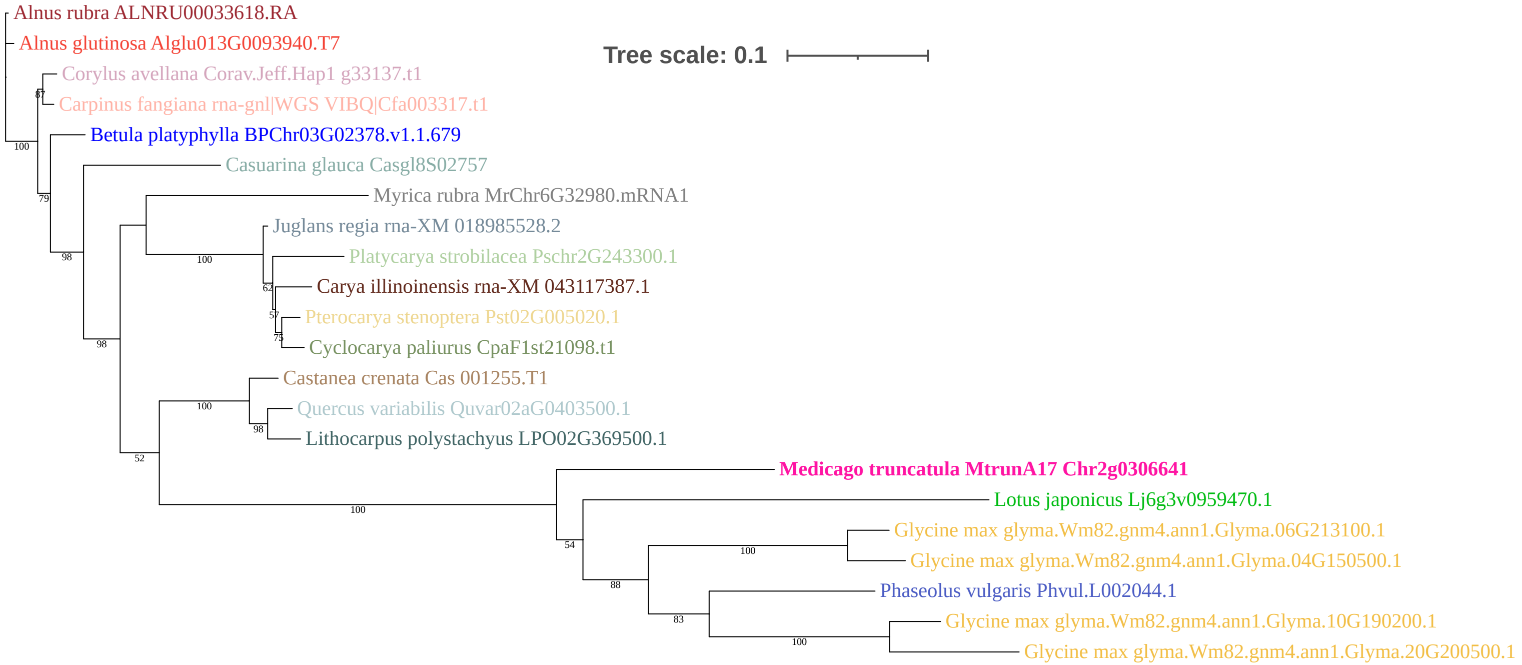

OG0011780:NENA

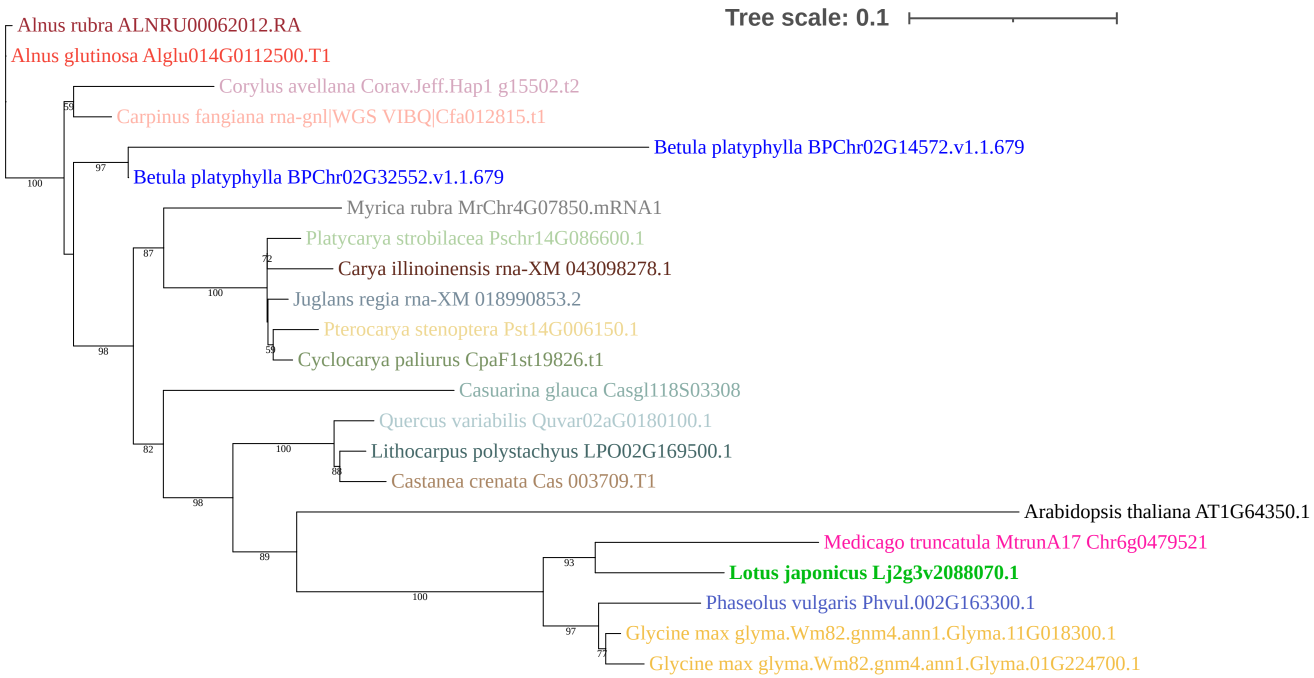

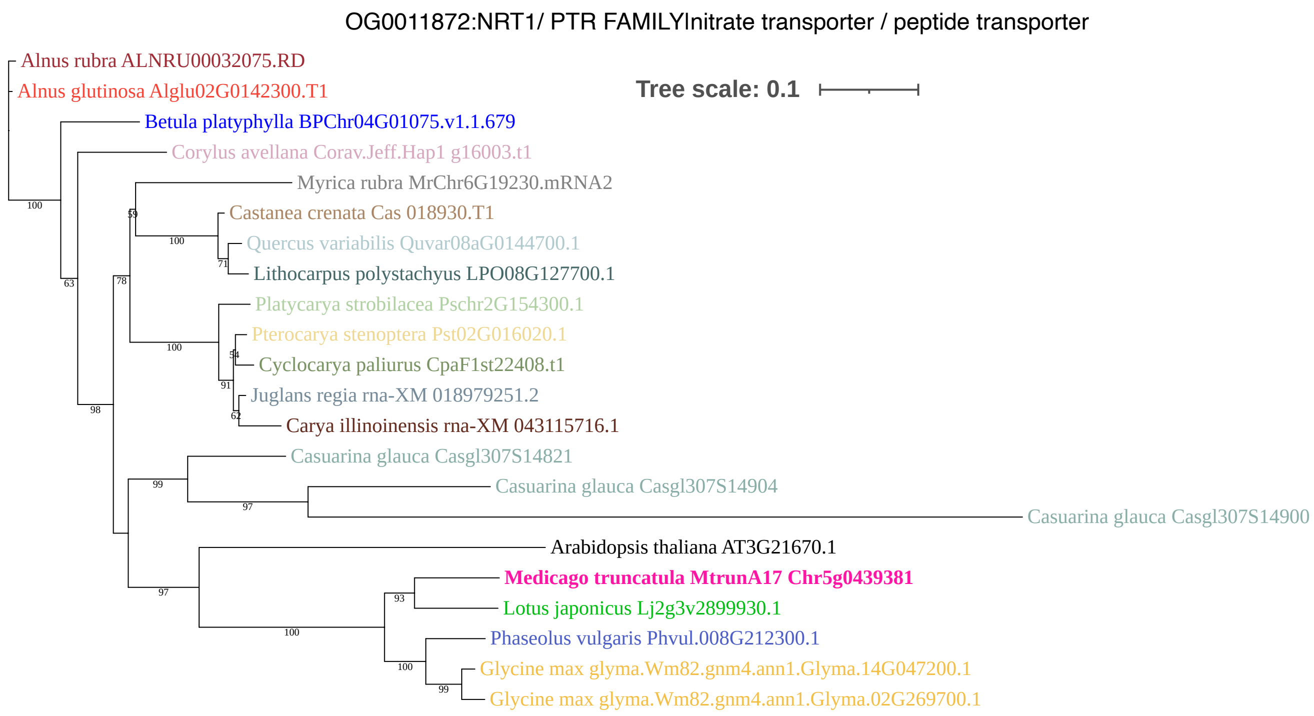

OG0011872:NRT1/ PTR FAMILY|nitrate transporter / peptide transporter
Tree scale: 0.1
Alnus rubra ALNRU00032075.RD
Alnus glutinosa Alglu02G0142300.T1
Betula platyphylla BPChr04G01075.v1.1.679
Corylus avellana Corav.Jeff.Hap1 g16003.t1
Myrica rubra MrChr6G19230.mRNA2
Castanea crenata Cas 018930.T1
Quercus variabilis Quvar08aG0144700.1
Lithocarpus polystachyus LPO08G127700.1
Platycarya strobilacea Pschr2G154300.1
Pterocarya stenoptera Pst02G016020.1
Cyclocarya paliurus CpaF1st22408.t1
Juglans regia rna-XM 018979251.2
Carya illinoinensis rna-XM 043115716.1
Casuarina glauca Casgl307S14821
Casuarina glauca Casgl307S14904
Casuarina glauca Casgl307S14900
Arabidopsis thaliana AT3G21670.1
Medicago truncatula MtrunA17 Chr5g0439381
Lotus japonicus Lj2g3v2899930.1
Phaseolus vulgaris Phvul.008G212300.1
Glycine max glyma.Wm82.gnm4.ann1.Glyma.14G047200.1
Glycine max glyma.Wm82.gnm4.ann1.Glyma.02G269700.1
100
59
100
71
63
78
100
54
91
62
98
99
97
97
93
100
100
99

OG0011909:Autophagy related gene

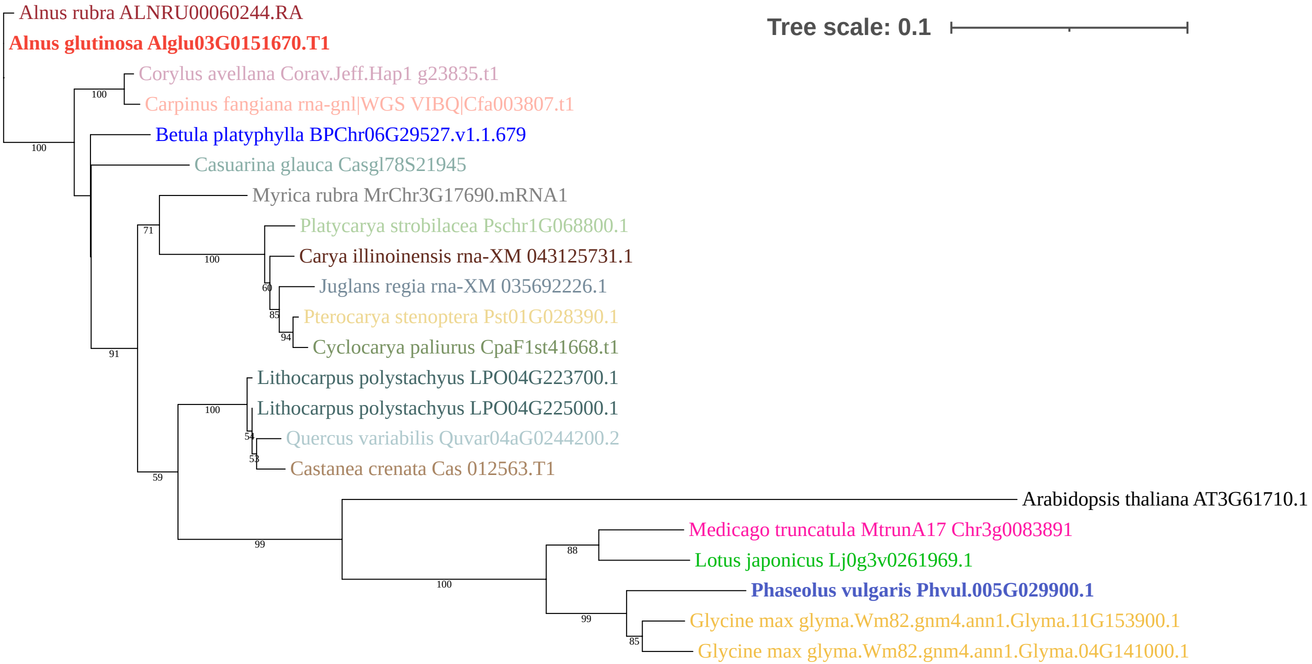

OG0012249:Digalactosyldiacylglycerol(DGDG) synthase 1

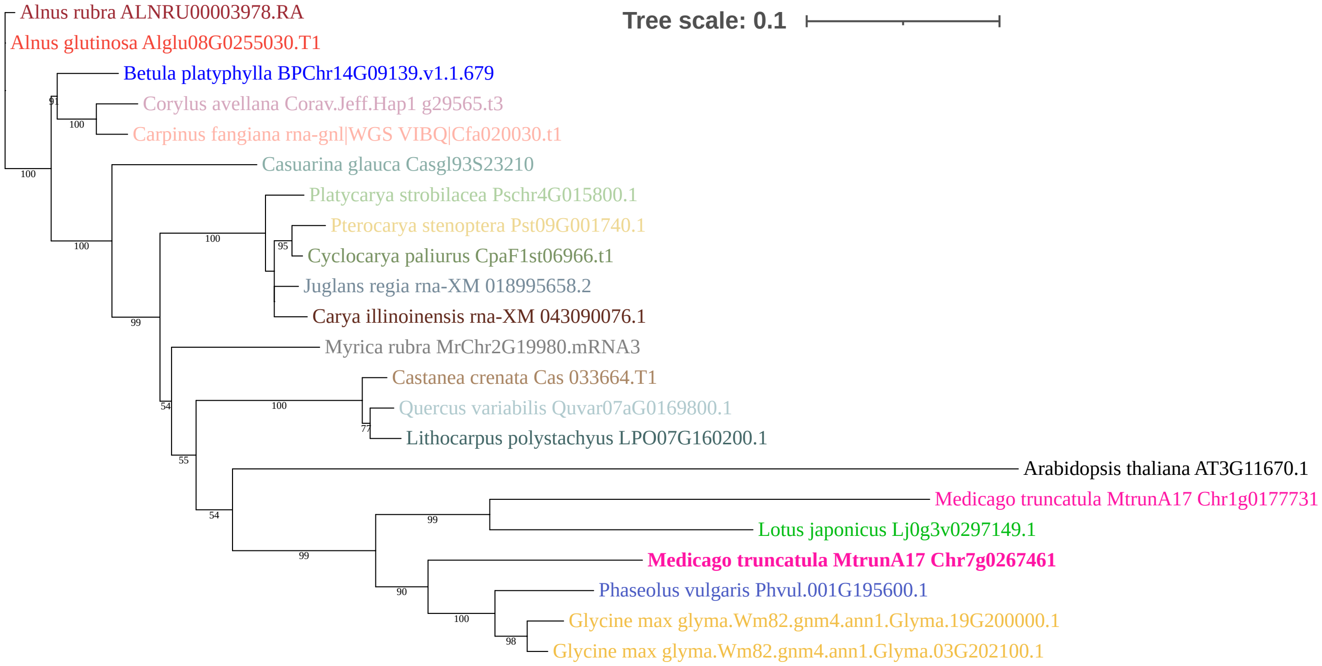

OG0012325:CELL DIVISION CYCLE16

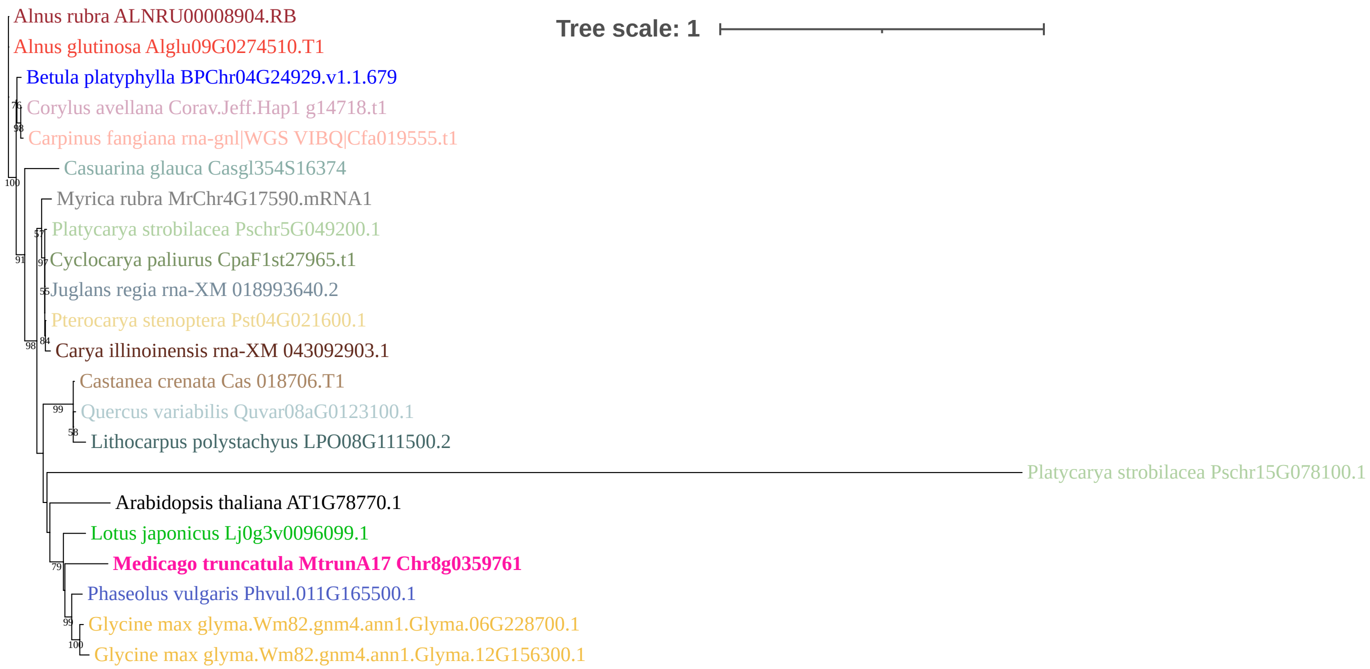

OG0012416:CHALCONE REDUCTASE

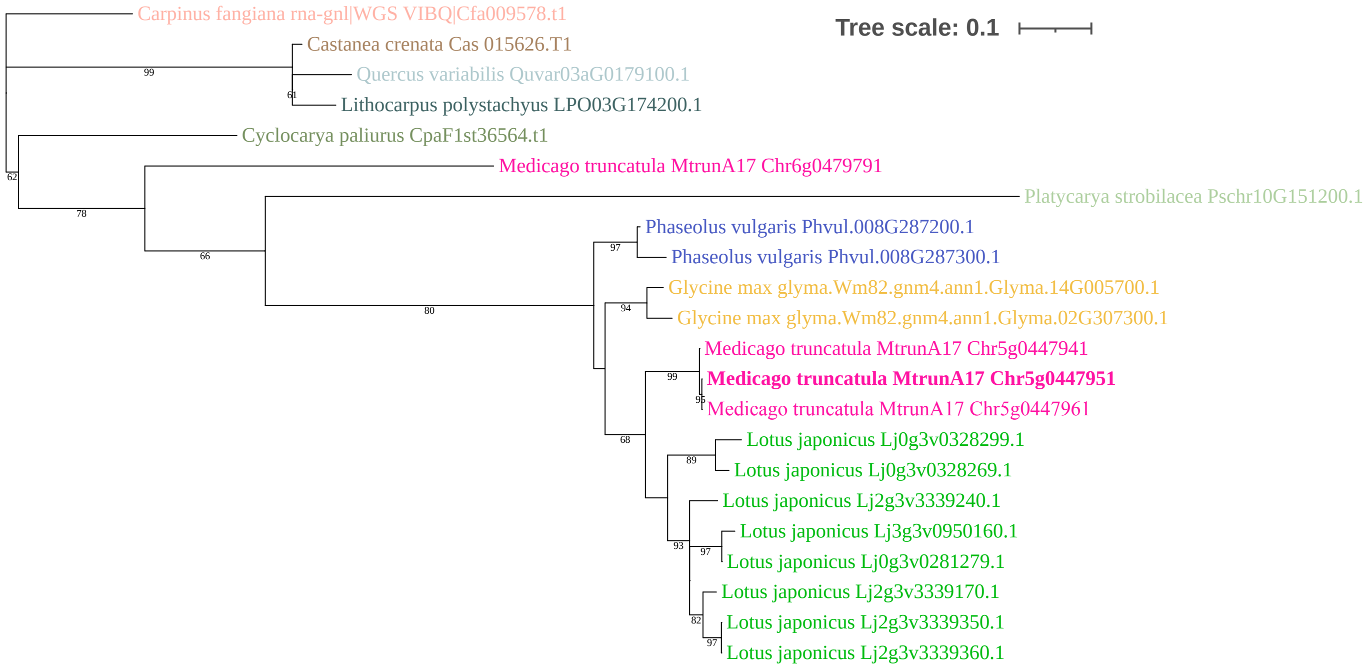

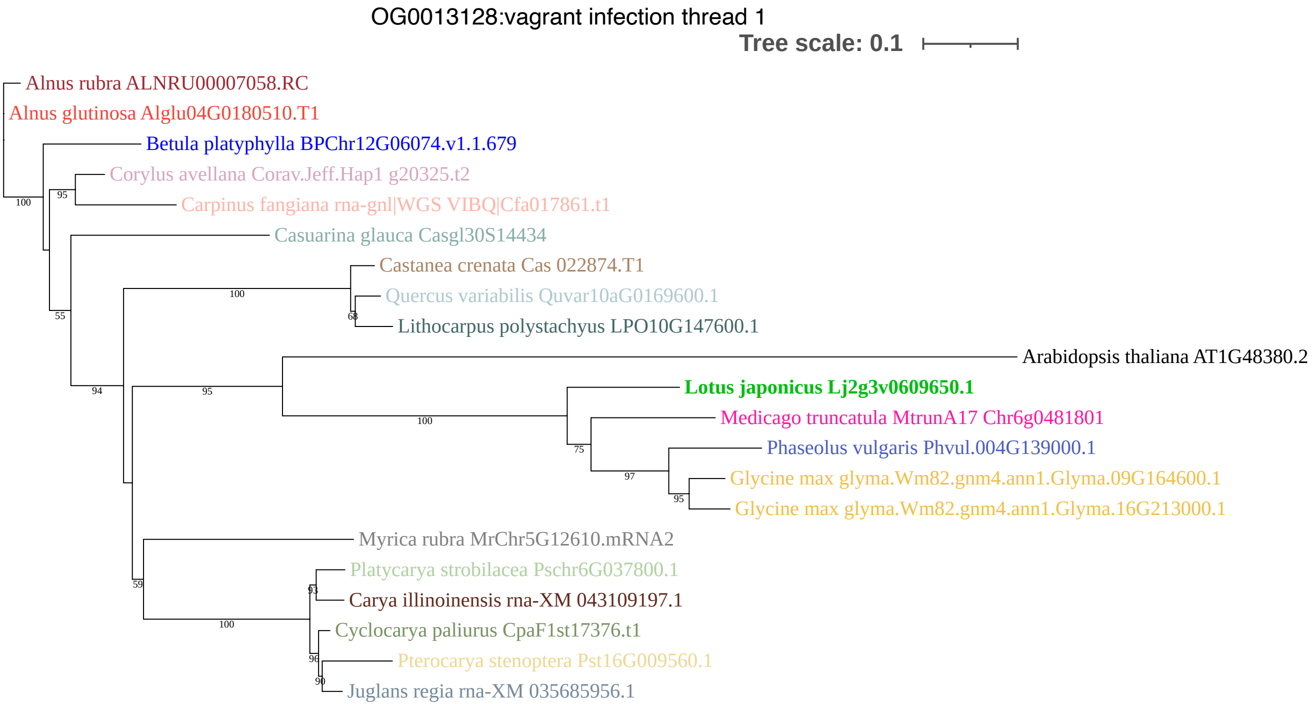
OG0013128:vagrant infection thread 1
Tree scale: 0.1
Alnus rubra ALNRU00007058.RC
Alnus glutinosa Alglu04G0180510.T1
Betula platyphylla BPChr12G06074.v1.1.679
Corylus avellana Corav.Jeff.Hap1 g20325.t2
Carpinus fangiana rna-gnl|WGS VIBQ|Cfa017861.t1
Casuarina glauca Casgl30S14434
Castanea crenata Cas 022874.T1
Quercus variabilis Quvar10aG0169600.1
Lithocarpus polystachyus LPO10G147600.1
Arabidopsis thaliana AT1G48380.2
Lotus japonicus Lj2g3v0609650.1
Medicago truncatula MtrunA17 Chr6g0481801
Phaseolus vulgaris Phvul.004G139000.1
Glycine max glyma.Wm82.gnm4.ann1.Glyma.09G164600.1
Glycine max glyma.Wm82.gnm4.ann1.Glyma.16G213000.1
Myrica rubra MrChr5G12610.mRNA2
Platycarya strobilacea Pschr6G037800.1
Carya illinoinensis rna-XM 043109197.1
Cyclocarya paliurus CpaF1st17376.t1
Pterocarya stenoptera Pst16G009560.1
Juglans regia rna-XM 035685956.1
100
95
55
94
100
68
95
100
75
97
95
59
100
93
96
90

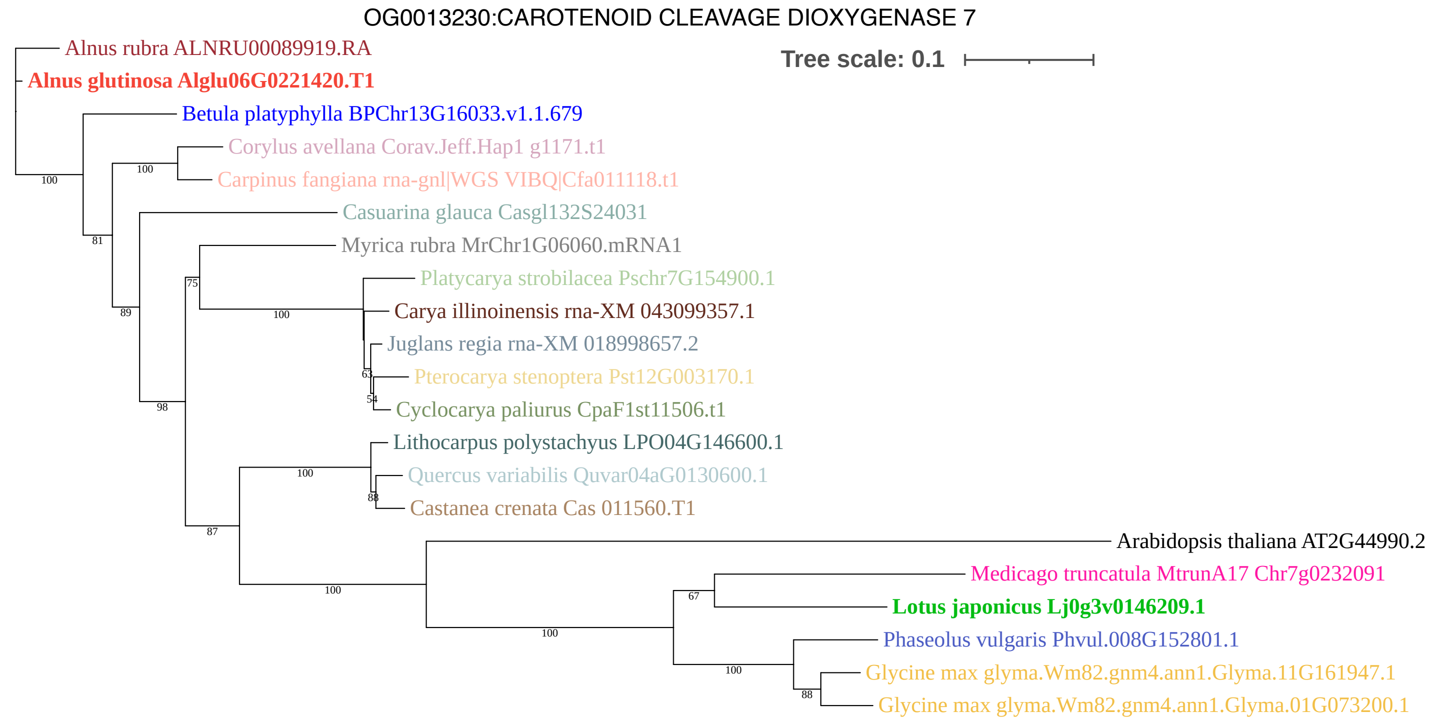
OG0013230:CAROTENOID CLEAVAGE DIOXYGENASE 7
Tree scale: 0.1
Alnus rubra ALNRU00089919.RA
Alnus glutinosa Alglu06G0221420.T1
Betula platyphylla BPChr13G16033.v1.1.679
Corylus avellana Corav.Jeff.Hap1 g1171.t1
Carpinus fangiana rna-gnl|WGS VIBQ|Cfa011118.t1
Casuarina glauca Casgl132S24031
Myrica rubra MrChr1G06060.mRNA1
Platycarya strobilacea Pschr7G154900.1
Carya illinoinensis rna-XM 043099357.1
Juglans regia rna-XM 018998657.2
Pterocarya stenoptera Pst12G003170.1
Cyclocarya paliurus CpaF1st11506.t1
Lithocarpus polystachyus LPO04G146600.1
Quercus variabilis Quvar04aG0130600.1
Castanea crenata Cas 011560.T1
Arabidopsis thaliana AT2G44990.2
Medicago truncatula MtrunA17 Chr7g0232091
Lotus japonicus Lj0g3v0146209.1
Phaseolus vulgaris Phvul.008G152801.1
Glycine max glyma.Wm82.gnm4.ann1.Glyma.11G161947.1
Glycine max glyma.Wm82.gnm4.ann1.Glyma.01G073200.1
100
100
81
89
75
100
63
54
98
100
88
87
100
67
100
100
88

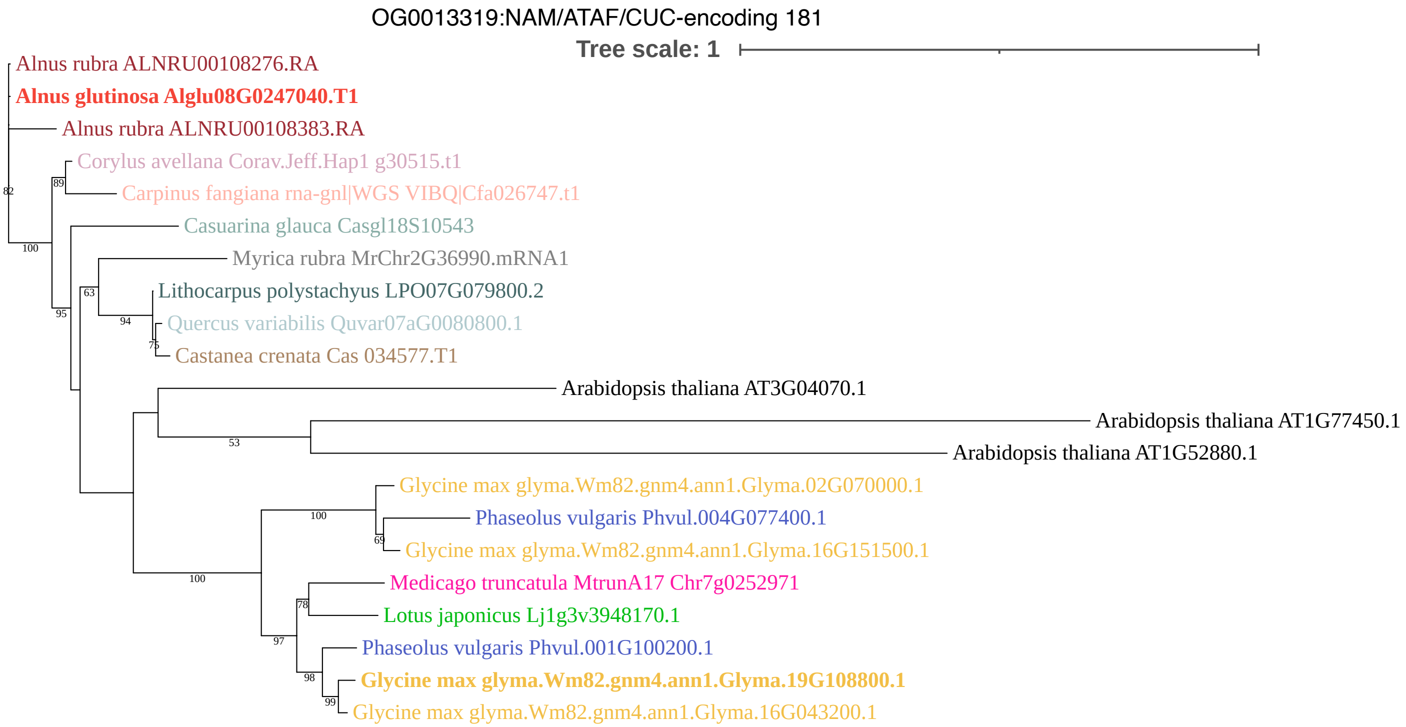
OG0013319:NAM/ATAF/CUC-encoding 181
Tree scale: 1
Alnus rubra ALNRU00108276.RA
Alnus glutinosa Alglu08G0247040.T1
Alnus rubra ALNRU00108383.RA
Corylus avellana Corav.Jeff.Hap1 g30515.t1
Carpinus fangiana rna-gnl|WGS VIBQ|Cfa026747.t1
Casuarina glauca Casgl18S10543
Myrica rubra MrChr2G36990.mRNA1
Lithocarpus polystachyus LPO07G079800.2
Quercus variabilis Quvar07aG0080800.1
Castanea crenata Cas 034577.T1
Arabidopsis thaliana AT3G04070.1
Arabidopsis thaliana AT1G77450.1
Arabidopsis thaliana AT1G52880.1
Glycine max glyma.Wm82.gnm4.ann1.Glyma.02G070000.1
Phaseolus vulgaris Phvul.004G077400.1
Glycine max glyma.Wm82.gnm4.ann1.Glyma.16G151500.1
Medicago truncatula MtrunA17 Chr7g0252971
Lotus japonicus Lj1g3v3948170.1
Phaseolus vulgaris Phvul.001G100200.1
Glycine max glyma.Wm82.gnm4.ann1.Glyma.19G108800.1
Glycine max glyma.Wm82.gnm4.ann1.Glyma.16G043200.1
82
89
100
95
63
94
75
53
100
69
100
78
97
98
99

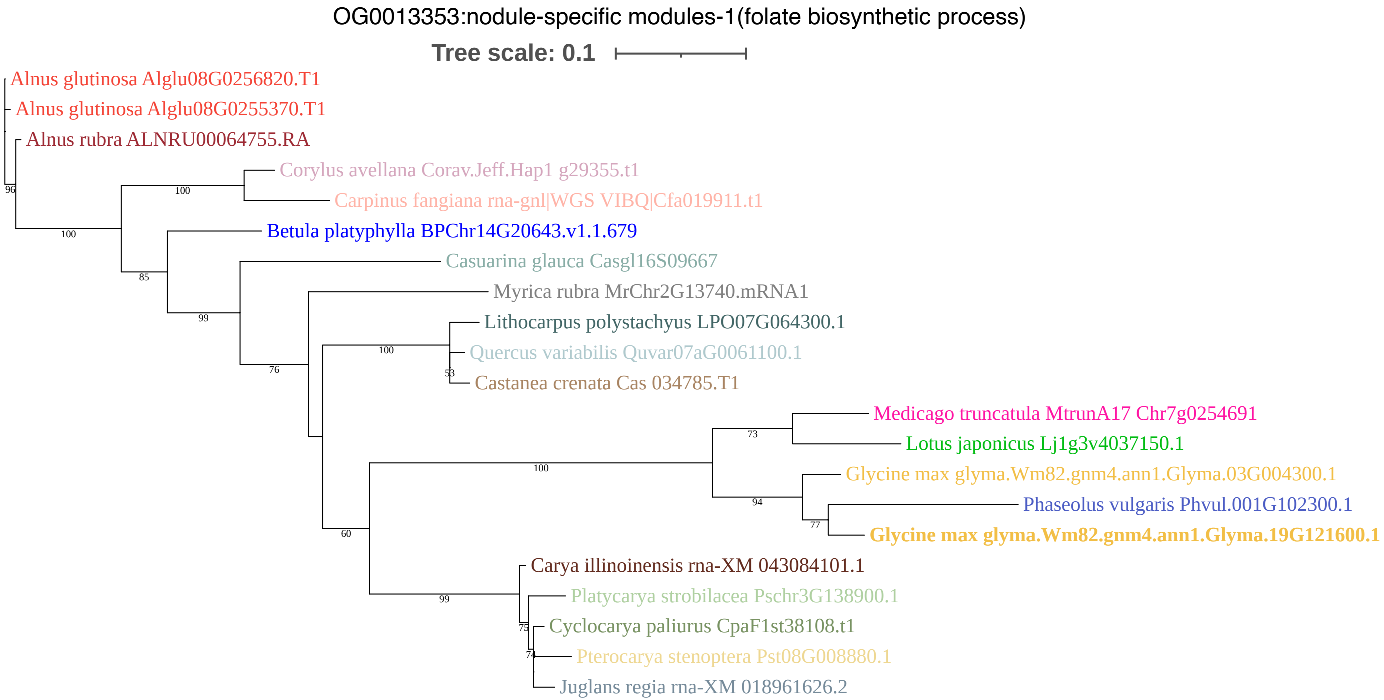

OG0013353:nodule-specific modules-1(folate biosynthetic process)
Tree scale: 0.1
Alnus glutinosa Alglu08G0256820.T1
Alnus glutinosa Alglu08G0255370.T1
Alnus rubra ALNRU00064755.RA
Corylus avellana Corav.Jeff.Hap1 g29355.t1
Carpinus fangiana rna-gnl|WGS VIBQ|Cfa019911.t1
Betula platyphylla BPChr14G20643.v1.1.679
Casuarina glauca Casgl16S09667
Myrica rubra MrChr2G13740.mRNA1
Lithocarpus polystachyus LPO07G064300.1
Quercus variabilis Quvar07aG0061100.1
Castanea crenata Cas 034785.T1
Medicago truncatula MtrunA17 Chr7g0254691
Lotus japonicus Lj1g3v4037150.1
Glycine max glyma.Wm82.gnm4.ann1.Glyma.03G004300.1
Phaseolus vulgaris Phvul.001G102300.1
Glycine max glyma.Wm82.gnm4.ann1.Glyma.19G121600.1
Carya illinoinensis rna-XM 043084101.1
Platycarya strobilacea Pschr3G138900.1
Cyclocarya paliurus CpaF1st38108.t1
Pterocarya stenoptera Pst08G008880.1
Juglans regia rna-XM 018961626.2
96
100
100
85
99
76
100
53
73
100
94
77
60
99
75
74

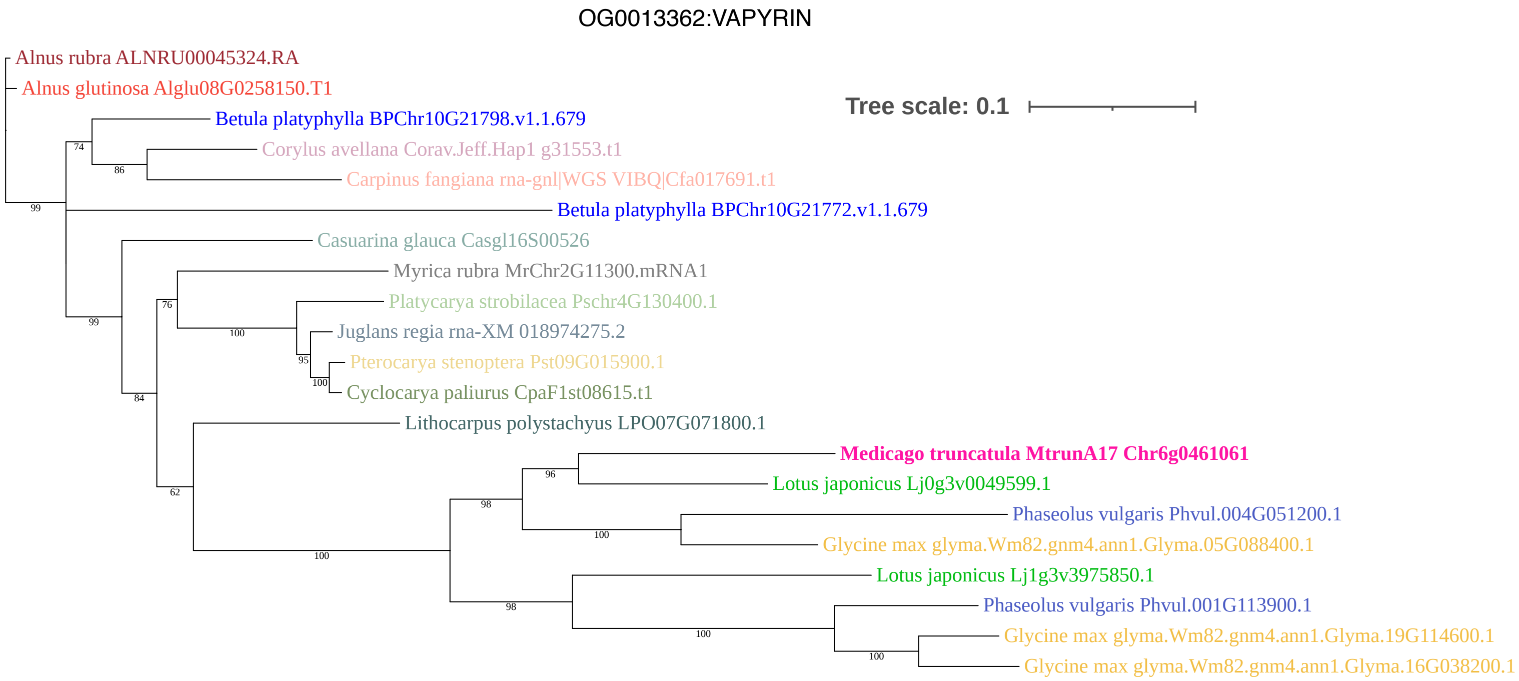
OG0013362:VAPYRIN
Tree scale: 0.1
Alnus rubra ALNRU00045324.RA
Alnus glutinosa Alglu08G0258150.T1
Betula platyphylla BPChr10G21798.v1.1.679
Corylus avellana Corav.Jeff.Hap1 g31553.t1
Carpinus fangiana rna-gnl|WGS VIBQ|Cfa017691.t1
Betula platyphylla BPChr10G21772.v1.1.679
Casuarina glauca Casgl16S00526
Myrica rubra MrChr2G11300.mRNA1
Platycarya strobilacea Pschr4G130400.1
Juglans regia rna-XM 018974275.2
Pterocarya stenoptera Pst09G015900.1
Cyclocarya paliurus CpaF1st08615.t1
Lithocarpus polystachyus LPO07G071800.1
Medicago truncatula MtrunA17 Chr6g0461061
Lotus japonicus Lj0g3v0049599.1
Phaseolus vulgaris Phvul.004G051200.1
Glycine max glyma.Wm82.gnm4.ann1.Glyma.05G088400.1
Lotus japonicus Lj1g3v3975850.1
Phaseolus vulgaris Phvul.001G113900.1
Glycine max glyma.Wm82.gnm4.ann1.Glyma.19G114600.1
Glycine max glyma.Wm82.gnm4.ann1.Glyma.16G038200.1
74
86
99
99
76
100
95
100
84
62
96
98
100
100
98
100
100

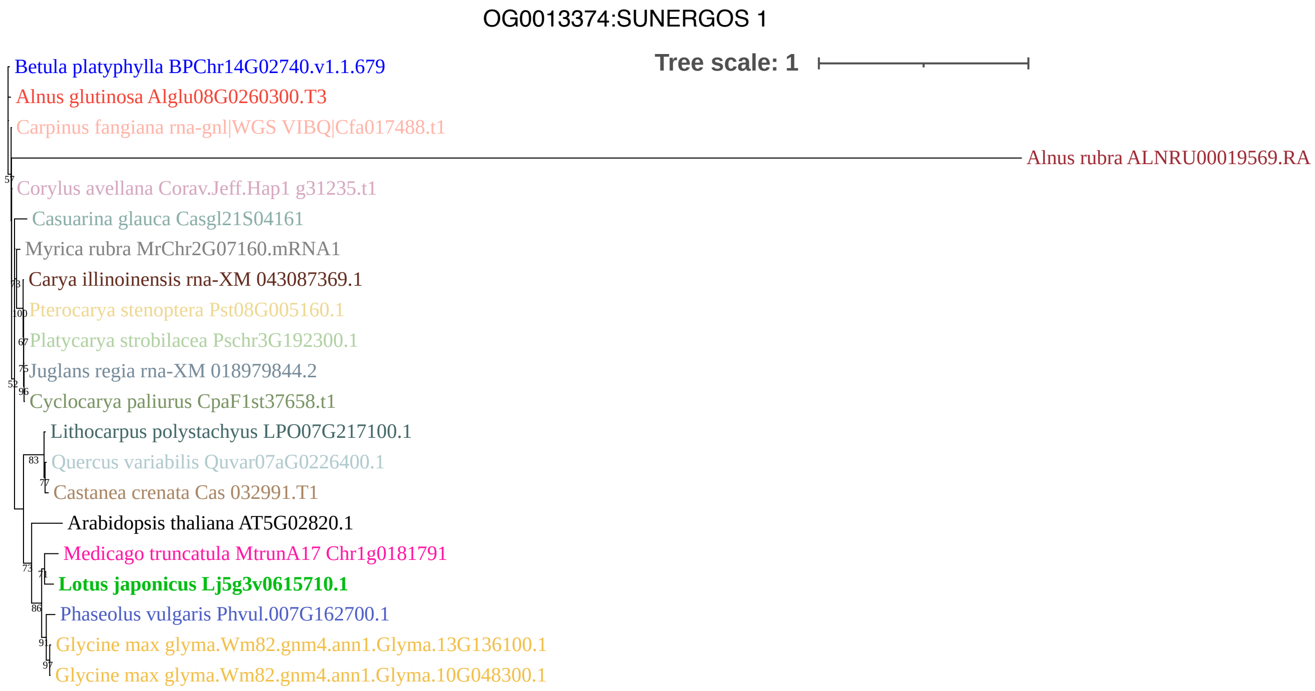
OG0013374:SUNERGOS 1
Tree scale: 1
Betula platyphylla BPChr14G02740.v1.1.679
Alnus glutinosa Alglu08G0260300.T3
Carpinus fangiana rna-gnl|WGS VIBQ|Cfa017488.t1
Alnus rubra ALNRU00019569.RA
Corylus avellana Corav.Jeff.Hap1 g31235.t1
Casuarina glauca Casgl21S04161
Myrica rubra MrChr2G07160.mRNA1
Carya illinoinensis rna-XM 043087369.1
Pterocarya stenoptera Pst08G005160.1
Platycarya strobilacea Pschr3G192300.1
Juglans regia rna-XM 018979844.2
Cyclocarya paliurus CpaF1st37658.t1
Lithocarpus polystachyus LPO07G217100.1
Quercus variabilis Quvar07aG0226400.1
Castanea crenata Cas 032991.T1
Arabidopsis thaliana AT5G02820.1
Medicago truncatula MtrunA17 Chr1g0181791
Lotus japonicus Lj5g3v0615710.1
Phaseolus vulgaris Phvul.007G162700.1
Glycine max glyma.Wm82.gnm4.ann1.Glyma.13G136100.1
Glycine max glyma.Wm82.gnm4.ann1.Glyma.10G048300.1
57
73
100
67
75
52
96
83
77
73
71
86
91
97

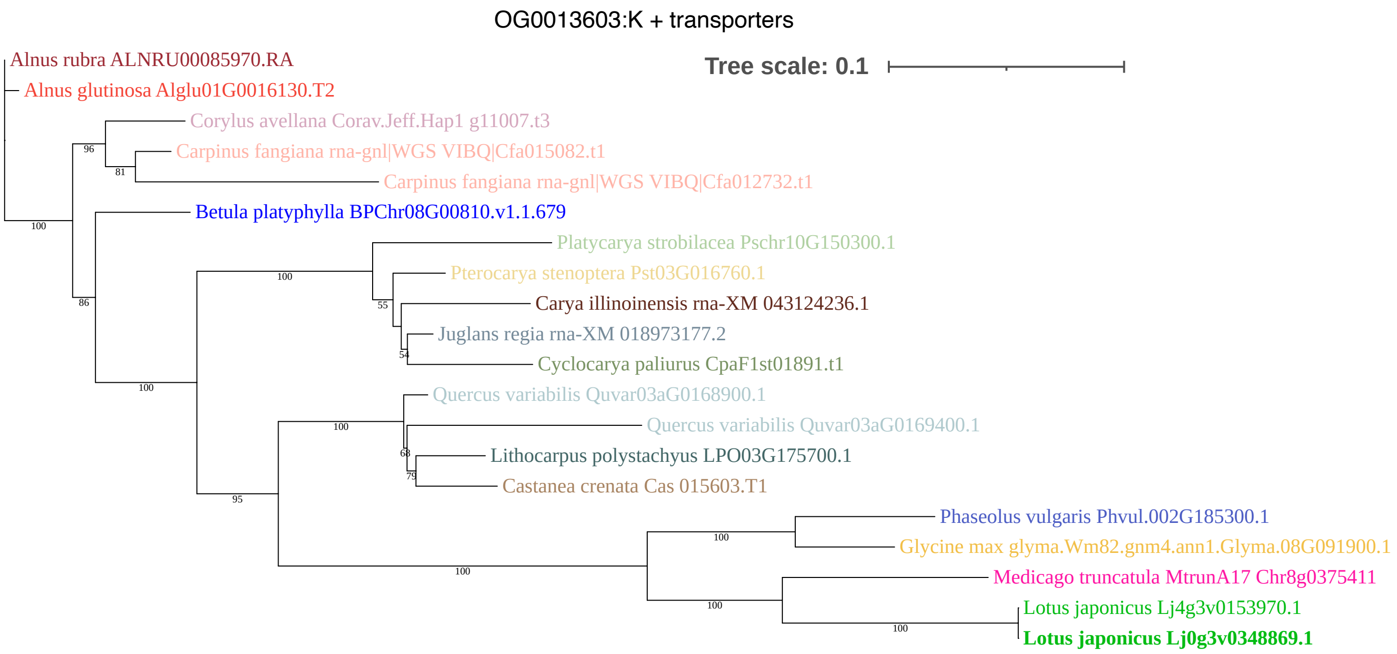
OG0013603:K + transporters
Tree scale: 0.1
Alnus rubra ALNRU00085970.RA
Alnus glutinosa Alglu01G0016130.T2
Corylus avellana Corav.Jeff.Hap1 g11007.t3
Carpinus fangiana rna-gnl|WGS VIBQ|Cfa015082.t1
Carpinus fangiana rna-gnl|WGS VIBQ|Cfa012732.t1
Betula platyphylla BPChr08G00810.v1.1.679
Platycarya strobilacea Pschr10G150300.1
Pterocarya stenoptera Pst03G016760.1
Carya illinoinensis rna-XM 043124236.1
Juglans regia rna-XM 018973177.2
Cyclocarya paliurus CpaF1st01891.t1
Quercus variabilis Quvar03aG0168900.1
Quercus variabilis Quvar03aG0169400.1
Lithocarpus polystachyus LPO03G175700.1
Castanea crenata Cas 015603.T1
Phaseolus vulgaris Phvul.002G185300.1
Glycine max glyma.Wm82.gnm4.ann1.Glyma.08G091900.1
Medicago truncatula MtrunA17 Chr8g0375411
Lotus japonicus Lj4g3v0153970.1
Lotus japonicus Lj0g3v0348869.1
96
81
100
86
100
55
54
100
68
79
95
100
100
100
100
100

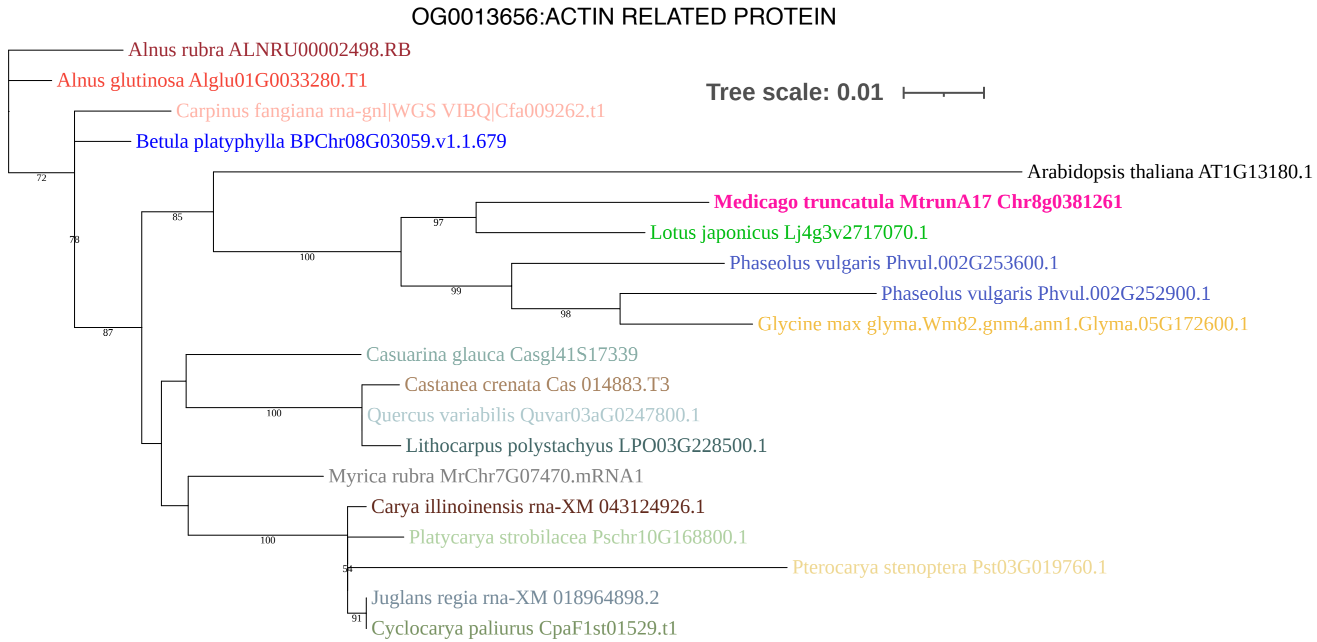
OG0013656:ACTIN RELATED PROTEIN
Tree scale: 0.01
Alnus rubra ALNRU00002498.RB
Alnus glutinosa Alglu01G0033280.T1
Carpinus fangiana rna-gnl|WGS VIBQ|Cfa009262.t1
Betula platyphylla BPChr08G03059.v1.1.679
Arabidopsis thaliana AT1G13180.1
Medicago truncatula MtrunA17 Chr8g0381261
Lotus japonicus Lj4g3v2717070.1
Phaseolus vulgaris Phvul.002G253600.1
Phaseolus vulgaris Phvul.002G252900.1
Glycine max glyma.Wm82.gnm4.ann1.Glyma.05G172600.1
Casuarina glauca Casgl41S17339
Castanea crenata Cas 014883.T3
Quercus variabilis Quvar03aG0247800.1
Lithocarpus polystachyus LPO03G228500.1
Myrica rubra MrChr7G07470.mRNA1
Carya illinoinensis rna-XM 043124926.1
Platycarya strobilacea Pschr10G168800.1
Pterocarya stenoptera Pst03G019760.1
Juglans regia rna-XM 018964898.2
Cyclocarya paliurus CpaF1st01529.t1
72
78
85
87
97
100
99
98
100
100
54
91

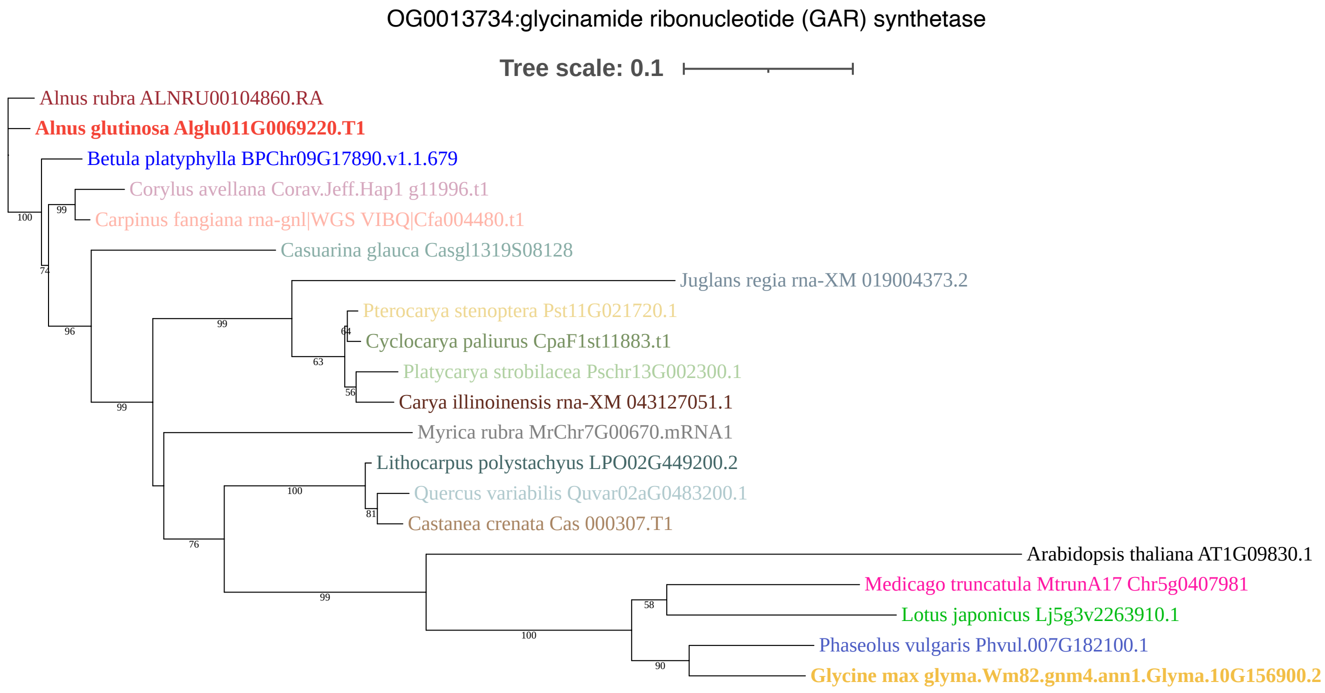
OG0013734:glycinamide ribonucleotide (GAR) synthetase
Tree scale: 0.1
Alnus rubra ALNRU00104860.RA
Alnus glutinosa Alglu011G0069220.T1
Betula platyphylla BPChr09G17890.v1.1.679
Corylus avellana Corav.Jeff.Hap1 g11996.t1
Carpinus fangiana rna-gnl|WGS VIBQ|Cfa004480.t1
Casuarina glauca Casgl1319S08128
Juglans regia rna-XM 019004373.2
Pterocarya stenoptera Pst11G021720.1
Cyclocarya paliurus CpaF1st11883.t1
Platycarya strobilacea Pschr13G002300.1
Carya illinoinensis rna-XM 043127051.1
Myrica rubra MrChr7G00670.mRNA1
Lithocarpus polystachyus LPO02G449200.2
Quercus variabilis Quvar02aG0483200.1
Castanea crenata Cas 000307.T1
Arabidopsis thaliana AT1G09830.1
Medicago truncatula MtrunA17 Chr5g0407981
Lotus japonicus Lj5g3v2263910.1
Phaseolus vulgaris Phvul.007G182100.1
Glycine max glyma.Wm82.gnm4.ann1.Glyma.10G156900.2
100
99
74
96
99
64
63
56
99
100
81
76
99
58
100
90

OG0014045:Cysteine-rich receptor-like kinases 12

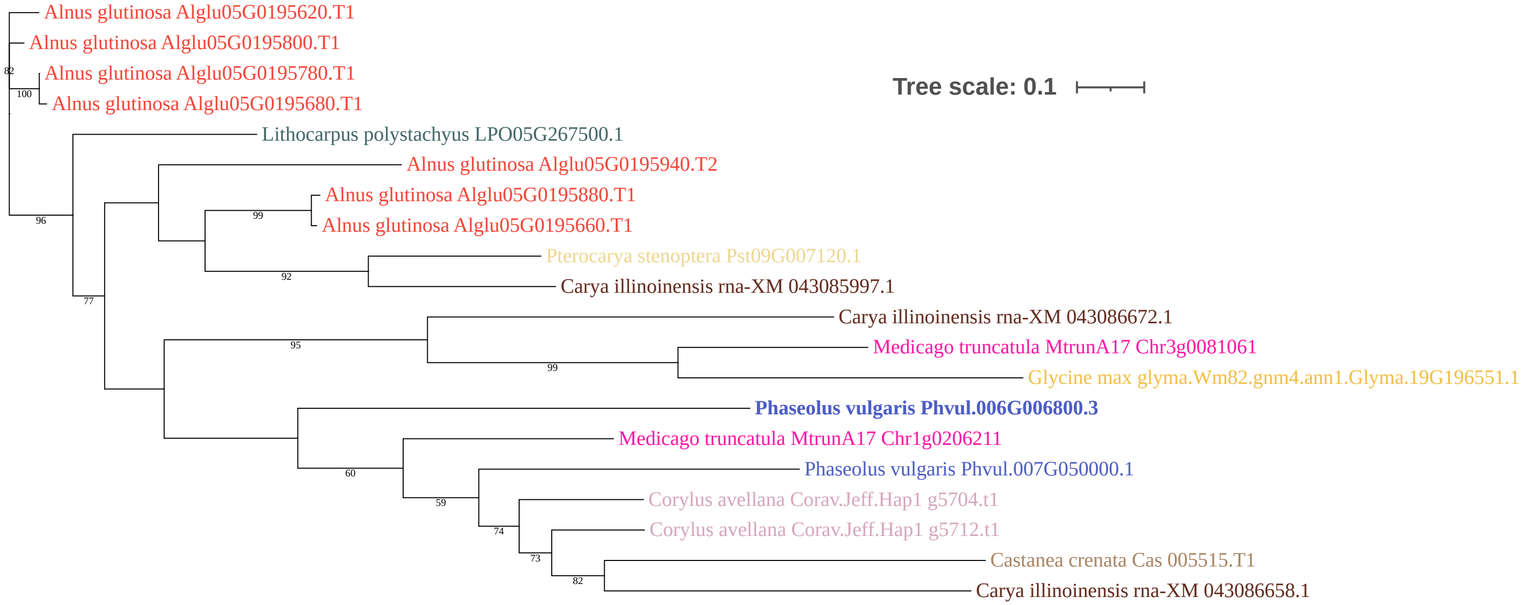

OG0014072:CALCIUM CALMODULIN KINASE

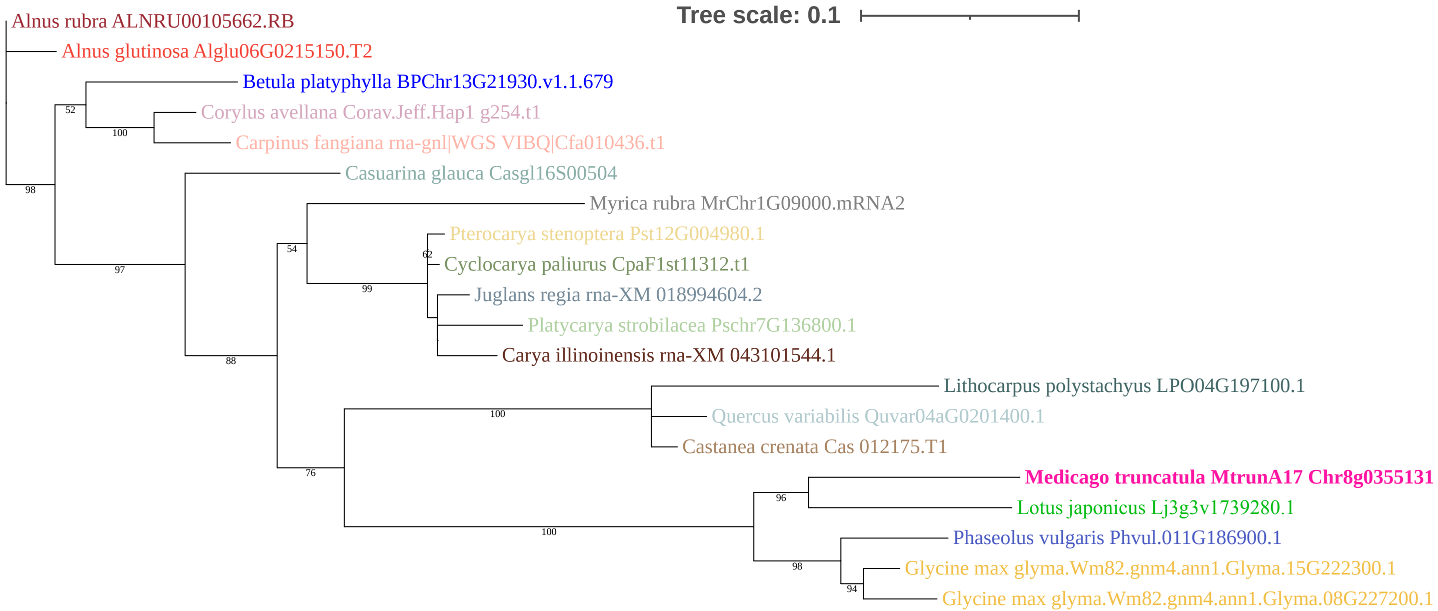

OG0014181:REGULATOR OF SYMBIOSOME DIFFERENTIATION

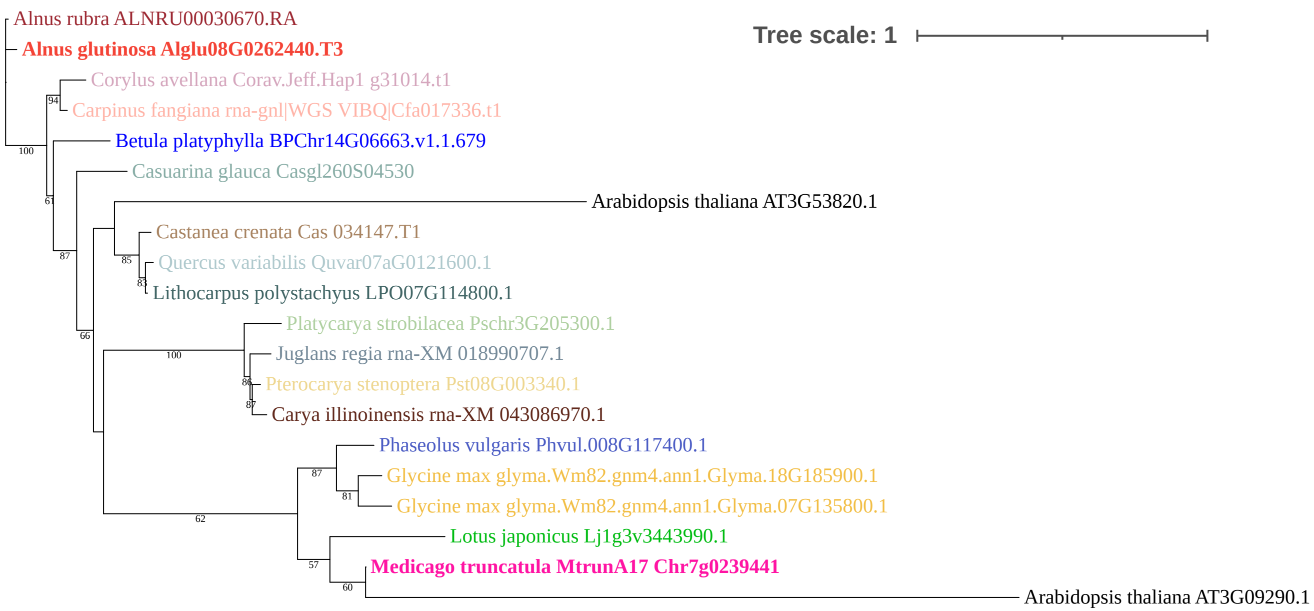

OG0014274:TGACG-motif binding factor 4/3

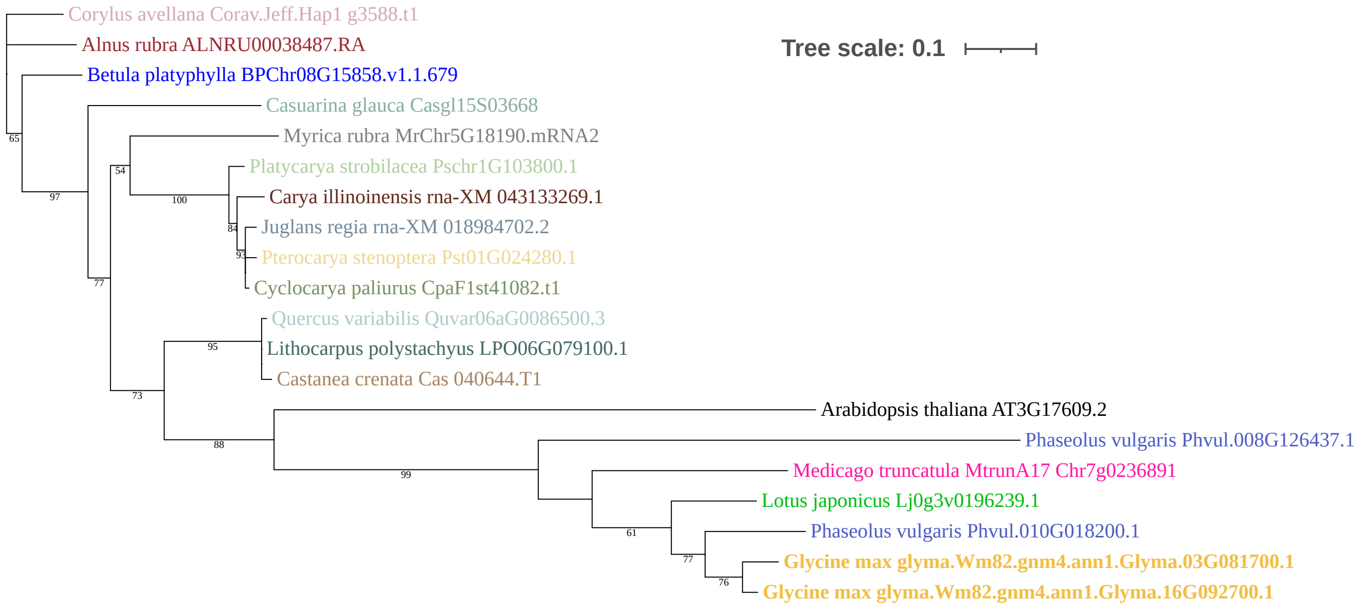

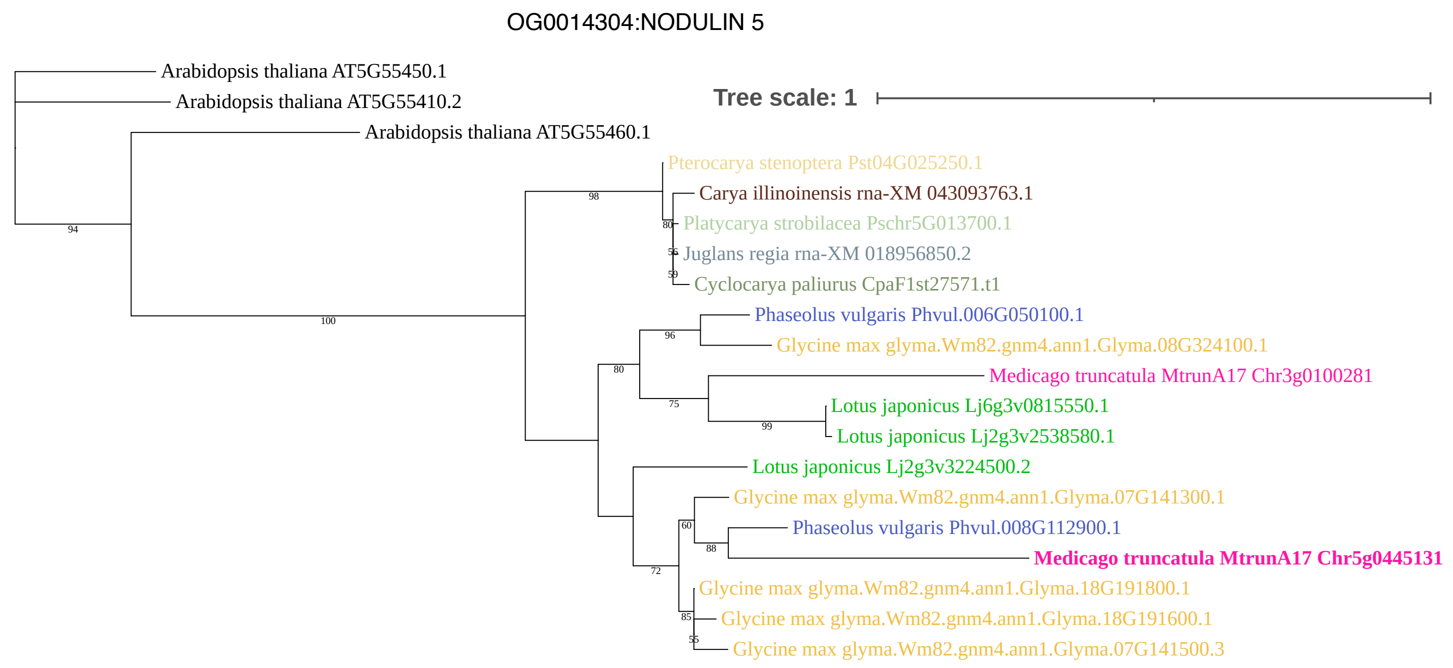

OG0014304:NODULIN 5
Tree scale: 1
Arabidopsis thaliana AT5G55450.1
Arabidopsis thaliana AT5G55410.2
Arabidopsis thaliana AT5G55460.1
Pterocarya stenoptera Pst04G025250.1
Carya illinoinensis rna-XM 043093763.1
Platycarya strobilacea Pschr5G013700.1
Juglans regia rna-XM 018956850.2
Cyclocarya paliurus CpaF1st27571.t1
Phaseolus vulgaris Phvul.006G050100.1
Glycine max glyma.Wm82.gnm4.ann1.Glyma.08G324100.1
Medicago truncatula MtrunA17 Chr3g0100281
Lotus japonicus Lj6g3v0815550.1
Lotus japonicus Lj2g3v2538580.1
Lotus japonicus Lj2g3v3224500.2
Glycine max glyma.Wm82.gnm4.ann1.Glyma.07G141300.1
Phaseolus vulgaris Phvul.008G112900.1
Medicago truncatula MtrunA17 Chr5g0445131
Glycine max glyma.Wm82.gnm4.ann1.Glyma.18G191800.1
Glycine max glyma.Wm82.gnm4.ann1.Glyma.18G191600.1
Glycine max glyma.Wm82.gnm4.ann1.Glyma.07G141500.3
94
100
98
80
56
59
96
80
75
99
60
88
72
85
55

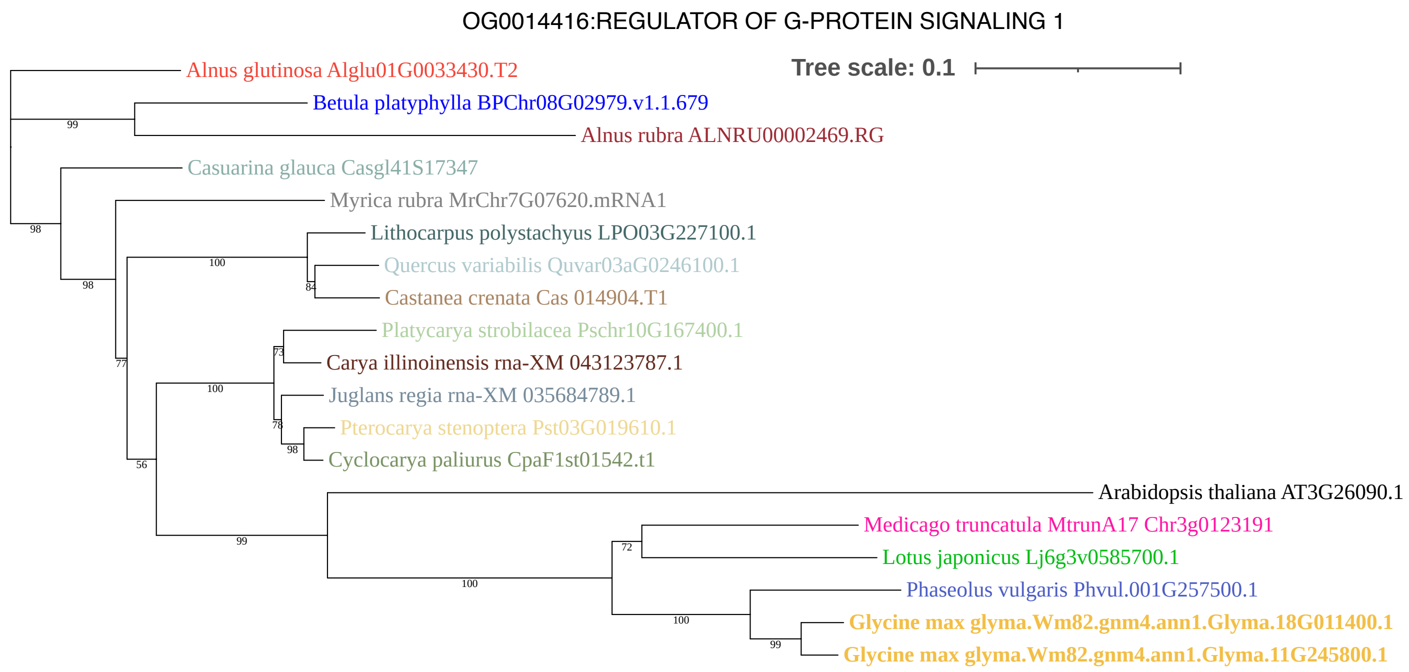
OG0014416:REGULATOR OF G-PROTEIN SIGNALING 1
Tree scale: 0.1
Alnus glutinosa Alglu01G0033430.T2
Betula platyphylla BPChr08G02979.v1.1.679
Alnus rubra ALNRU00002469.RG
Casuarina glauca Casgl41S17347
Myrica rubra MrChr7G07620.mRNA1
Lithocarpus polystachyus LPO03G227100.1
Quercus variabilis Quvar03aG0246100.1
Castanea crenata Cas 014904.T1
Platycarya strobilacea Pschr10G167400.1
Carya illinoinensis rna-XM 043123787.1
Juglans regia rna-XM 035684789.1
Pterocarya stenoptera Pst03G019610.1
Cyclocarya paliurus CpaF1st01542.t1
Arabidopsis thaliana AT3G26090.1
Medicago truncatula MtrunA17 Chr3g0123191
Lotus japonicus Lj6g3v0585700.1
Phaseolus vulgaris Phvul.001G257500.1
Glycine max glyma.Wm82.gnm4.ann1.Glyma.18G011400.1
Glycine max glyma.Wm82.gnm4.ann1.Glyma.11G245800.1
99
98
98
100
84
77
73
100
78
98
56
99
100
72
100
99

OG0014510:INTERACTING PROTEIN DMI 3

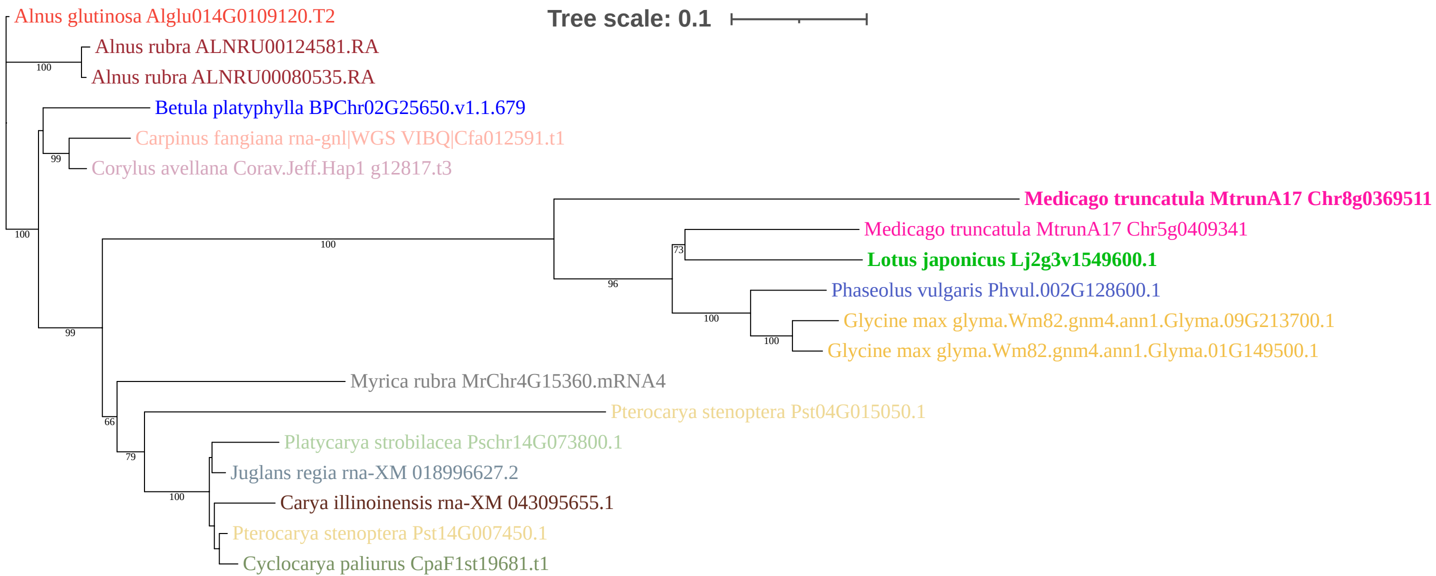

OG0014592:CCAMK INTERACTING PROTEIN OF APPROXIMATELY 73 KDa

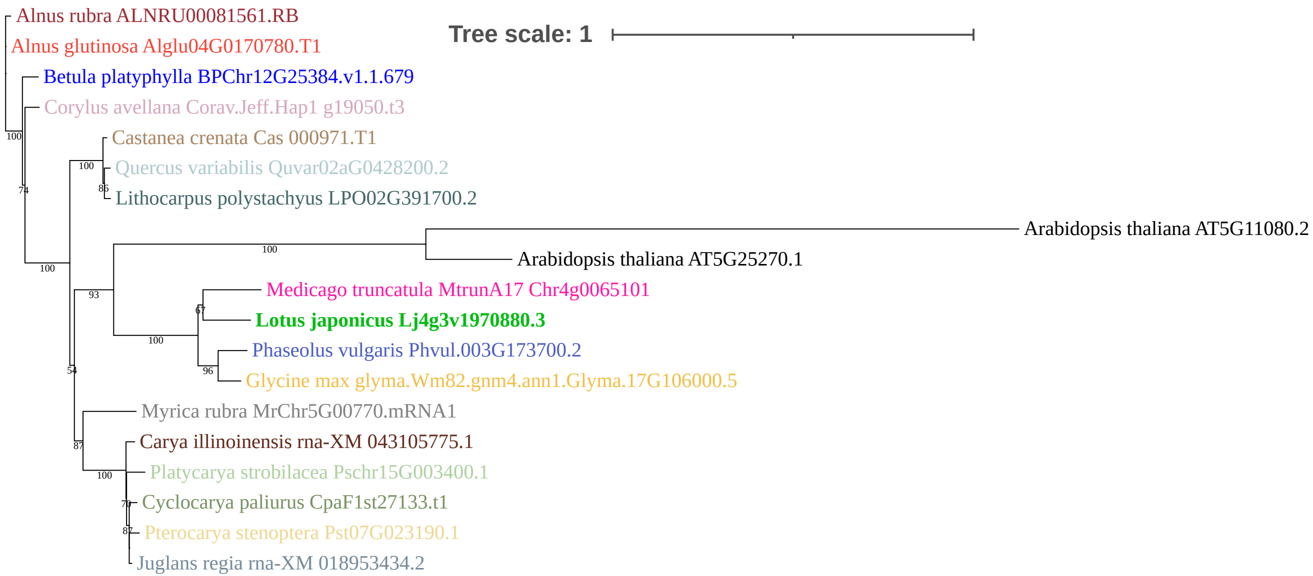

OG0014878:SYMBIOTIC RECEPTOR KINASE/DOES NOT MAKE Infections 2

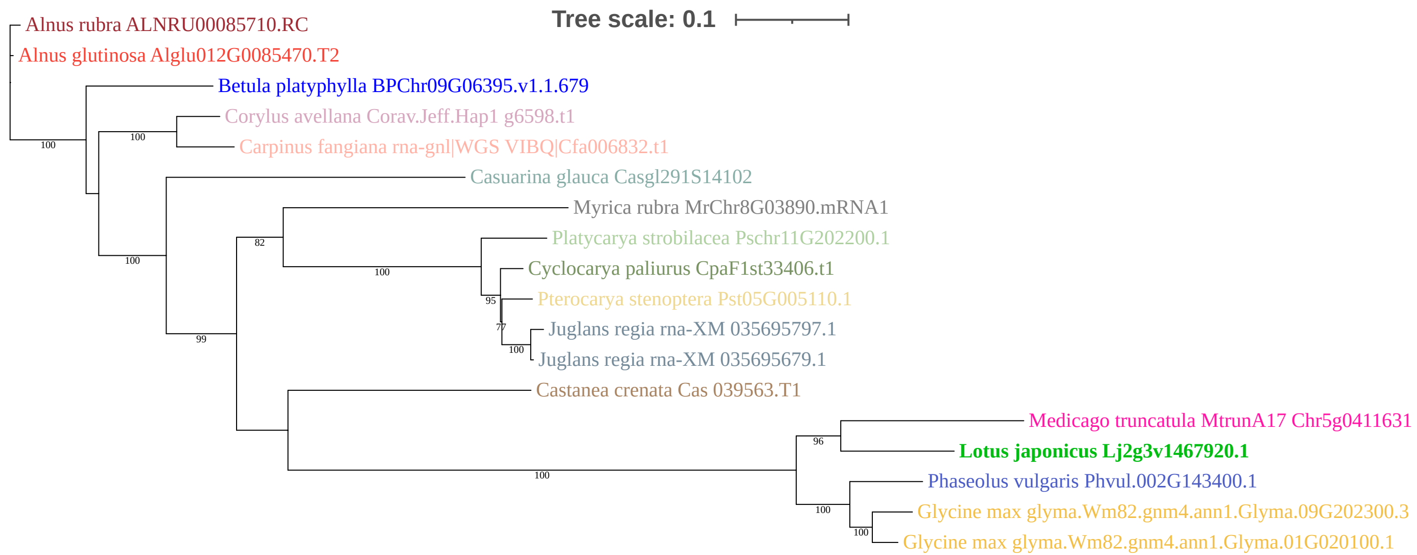

OG0015233:PLANT U BOX PROTEIN 1

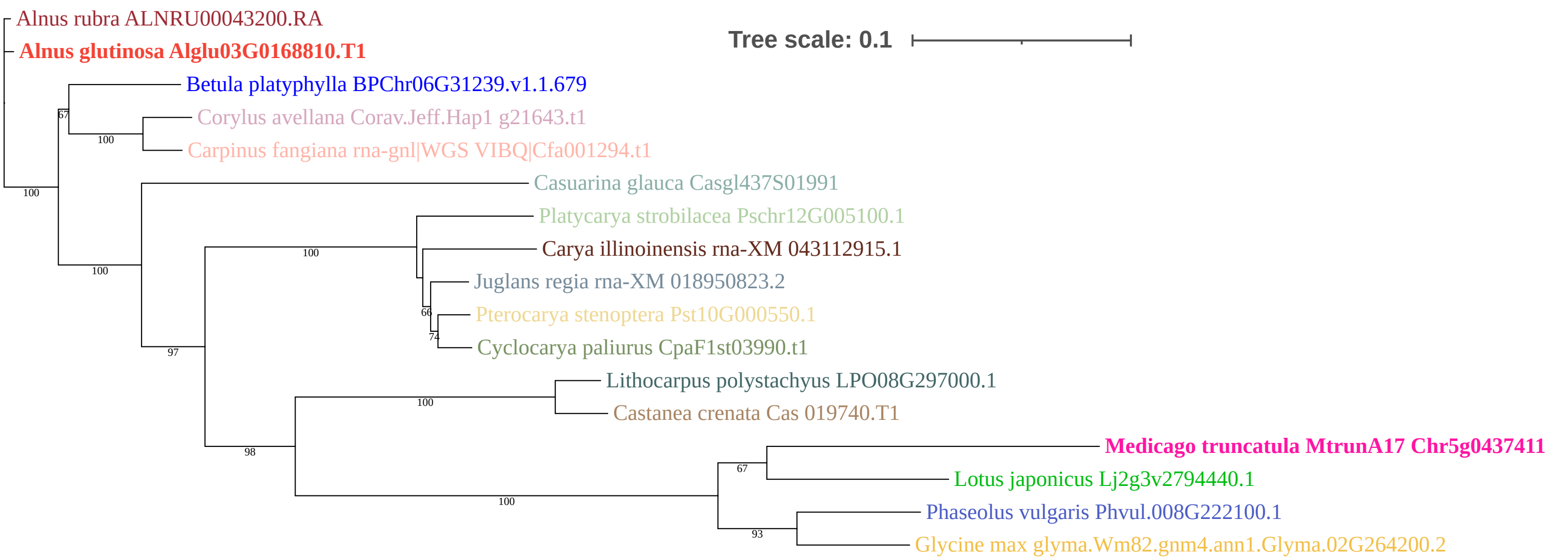

OG0015886:C-terminally encoded peptide

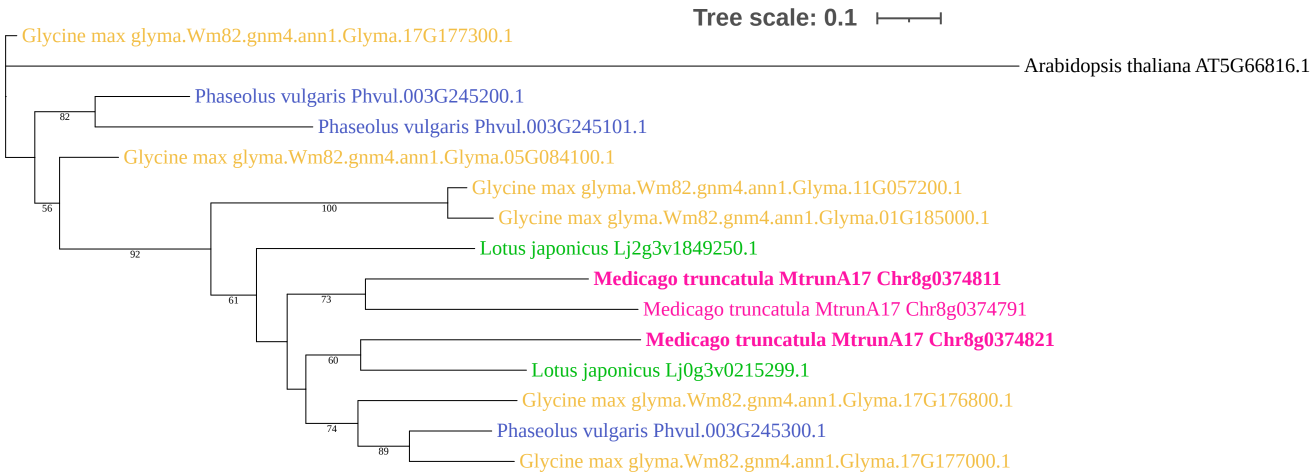

OG0015955:matrix metalloproteinase-like 1|Mt Nodulin9

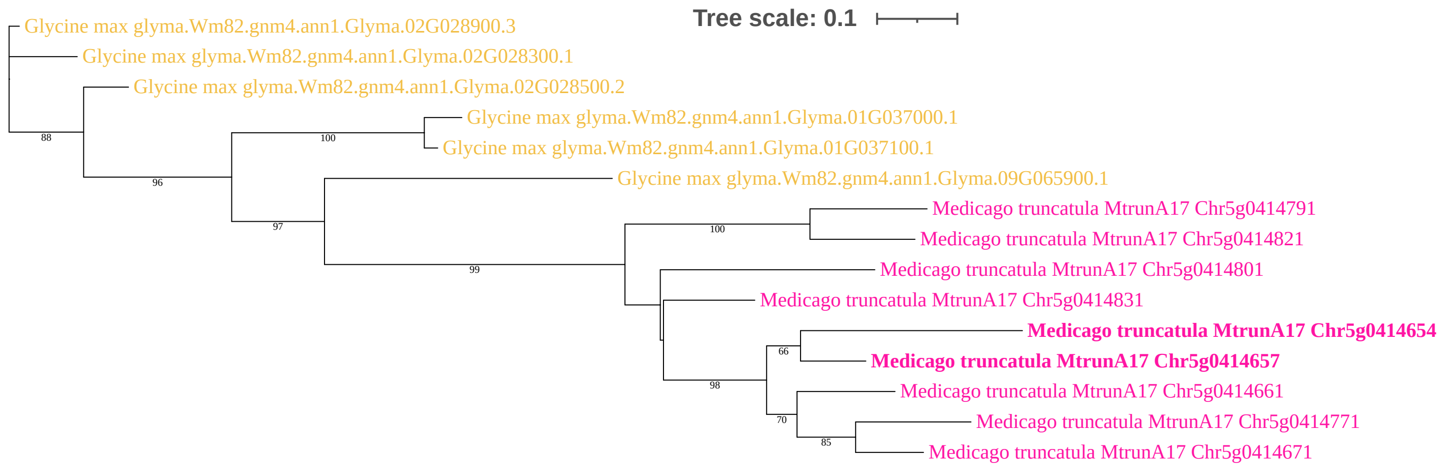

OG0016265:Nitrogen FixationSPECIFICITY 1

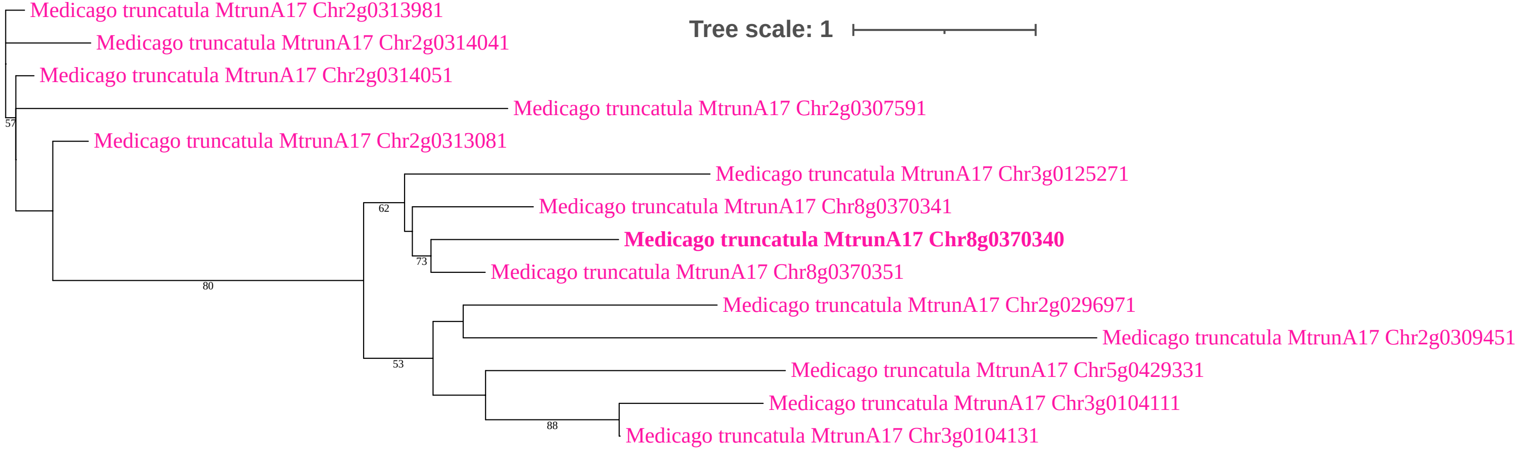

OG0016863:LEED..PEED family

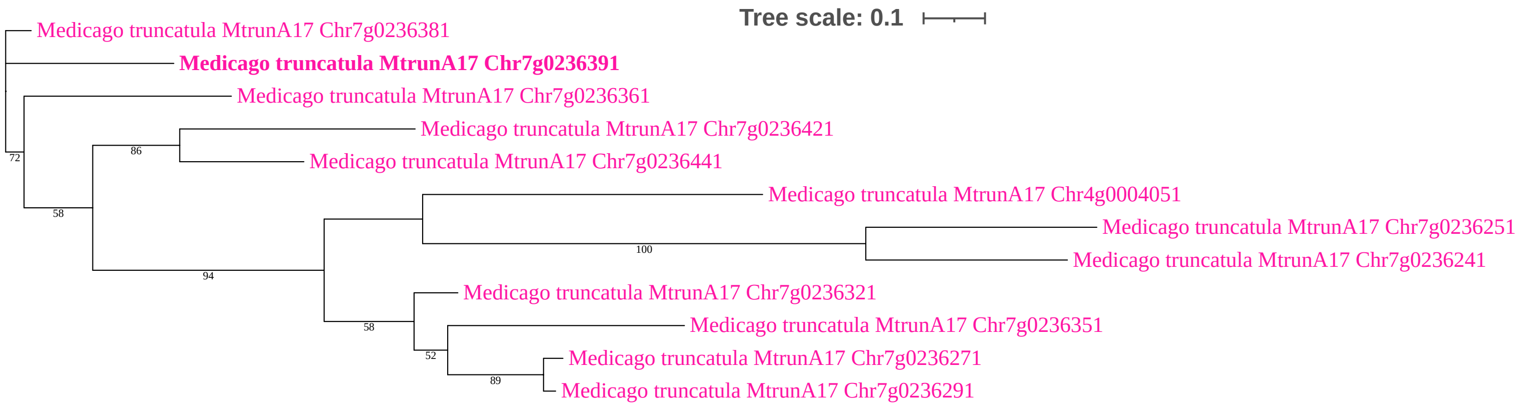

OG0017146:FIXATION UNDER NITRATE

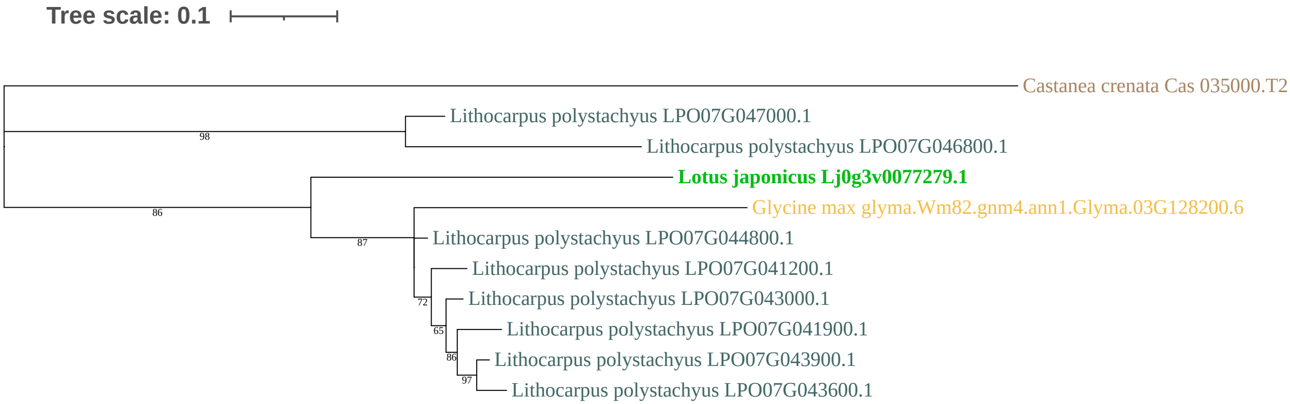

OG0017310:FAIL IN ENLARGEMENT of infection CELLS

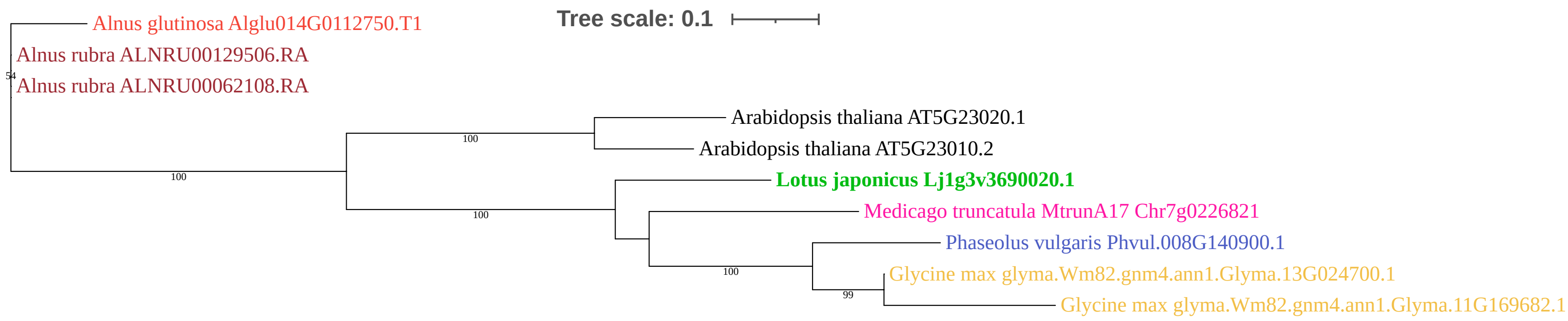

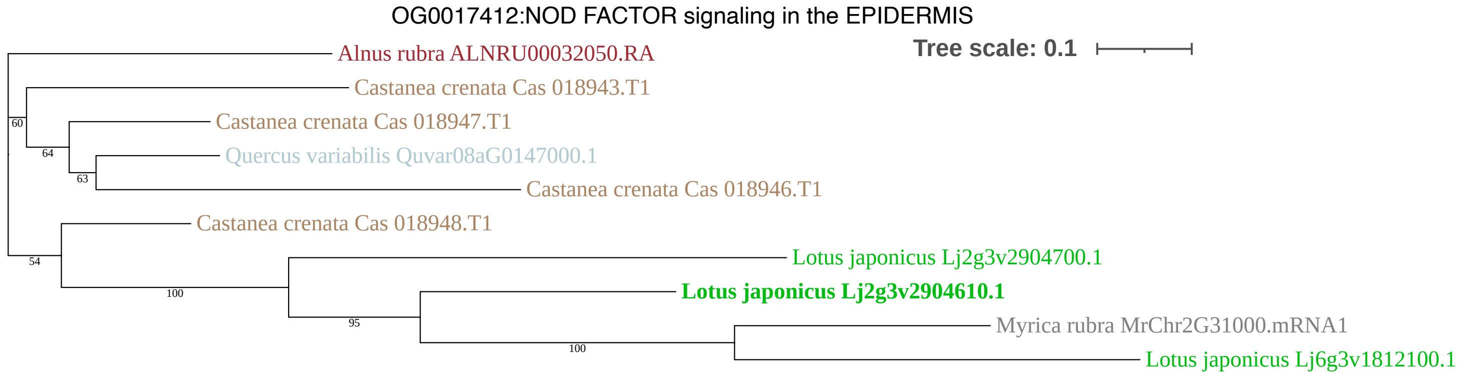
OG0017412:NOD FACTOR signaling in the EPIDERMIS
Tree scale: 0.1
Alnus rubra ALNRU00032050.RA
Castanea crenata Cas 018943.T1
Castanea crenata Cas 018947.T1
Quercus variabilis Quvar08aG0147000.1
Castanea crenata Cas 018946.T1
Castanea crenata Cas 018948.T1
Lotus japonicus Lj2g3v2904700.1
Lotus japonicus Lj2g3v2904610.1
Myrica rubra MrChr2G31000.mRNA1
Lotus japonicus Lj6g3v1812100.1
60
64
63
54
100
95
100

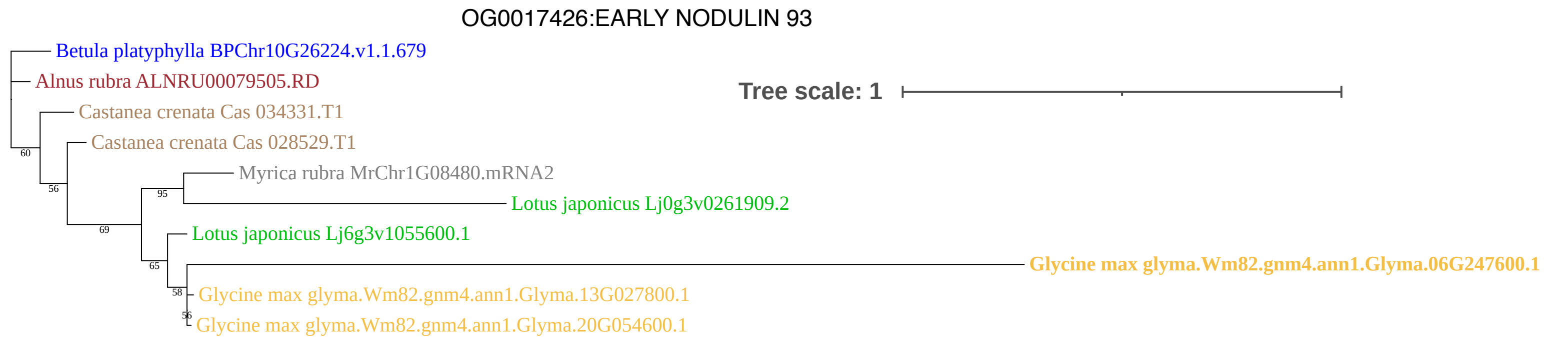
OG0017426:EARLY NODULIN 93
Tree scale: 1
Betula platyphylla BPChr10G26224.v1.1.679
Alnus rubra ALNRU00079505.RD
Castanea crenata Cas 034331.T1
Castanea crenata Cas 028529.T1
Myrica rubra MrChr1G08480.mRNA2
Lotus japonicus Lj0g3v0261909.2
Lotus japonicus Lj6g3v1055600.1
Glycine max glyma.Wm82.gnm4.ann1.Glyma.06G247600.1
Glycine max glyma.Wm82.gnm4.ann1.Glyma.13G027800.1
Glycine max glyma.Wm82.gnm4.ann1.Glyma.20G054600.1
60
56
95
69
65
58
56

OG0019197:Golven/ Root Growth Factor|Root Meristem Growth Factor(peptide)

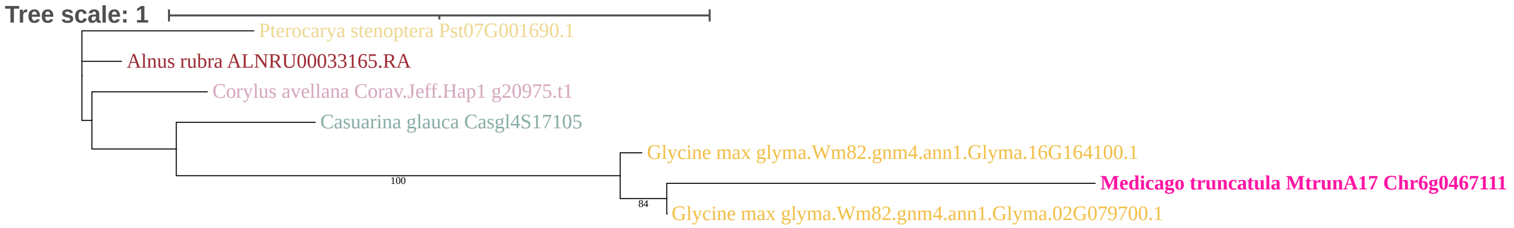

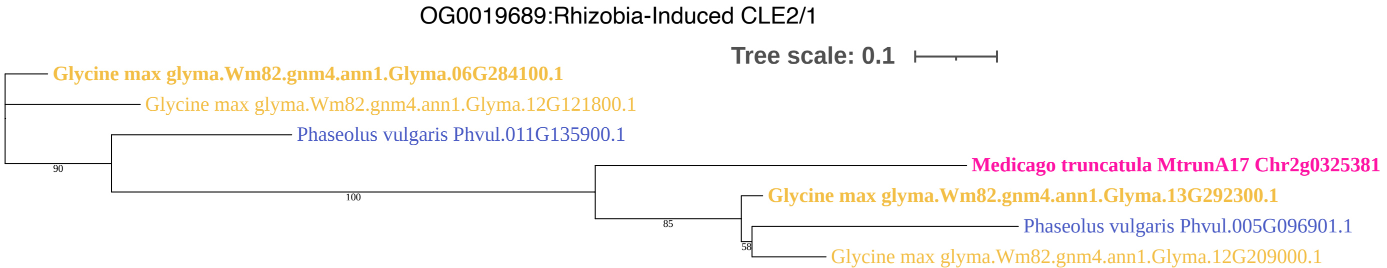
OG0019689:Rhizobia-Induced CLE2/1
Tree scale: 0.1
Glycine max glyma.Wm82.gnm4.ann1.Glyma.06G284100.1
Glycine max glyma.Wm82.gnm4.ann1.Glyma.12G121800.1
Phaseolus vulgaris Phvul.011G135900.1
90
Medicago truncatula MtrunA17 Chr2g0325381
100
Glycine max glyma.Wm82.gnm4.ann1.Glyma.13G292300.1
85
Phaseolus vulgaris Phvul.005G096901.1
58
Glycine max glyma.Wm82.gnm4.ann1.Glyma.12G209000.1

OG0021024:NODULE-SPECIFIC PLAT DOMAIN PROTEIN 2

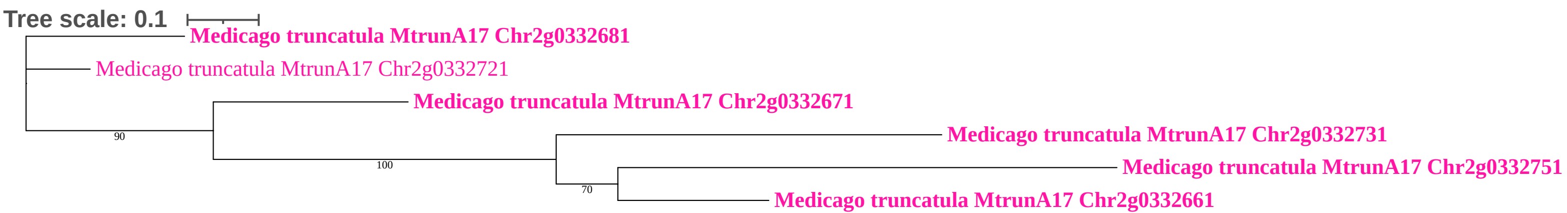

OG0022391:NODULE WITH ACTIVATED DEFENSE 1

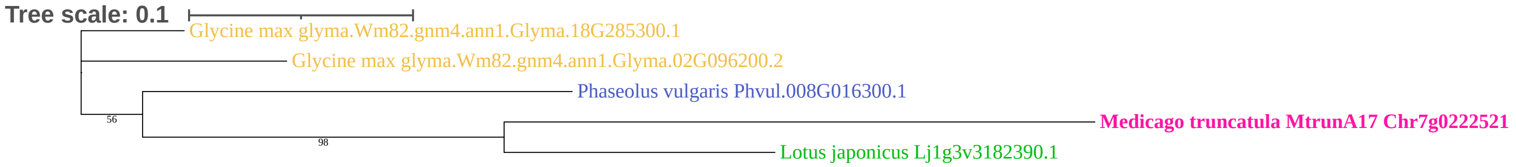

OG0023880:synaptotagmin 1

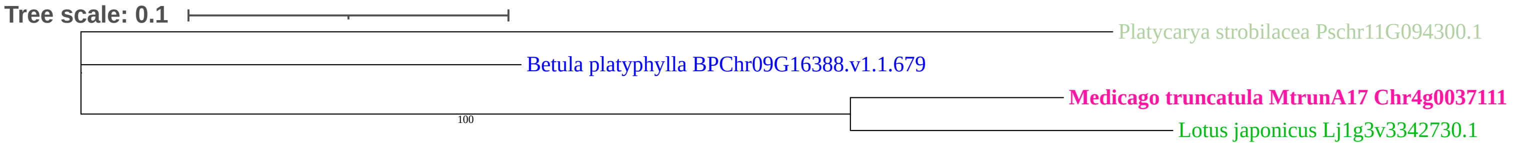

Supplement: Supplementary file 3 — Dataset S1. 275 phylogenetic trees of nitrogen‐fixing related genes from 20 species. [file PCE-49-3003-s002.pdf]
